# Spin Hyperpolarization in Modern Magnetic Resonance

**DOI:** 10.1021/acs.chemrev.2c00534

**Published:** 2023-01-26

**Authors:** James Eills, Dmitry Budker, Silvia Cavagnero, Eduard Y. Chekmenev, Stuart J. Elliott, Sami Jannin, Anne Lesage, Jörg Matysik, Thomas Meersmann, Thomas Prisner, Jeffrey A. Reimer, Hanming Yang, Igor V. Koptyug

**Affiliations:** † Institute for Bioengineering of Catalonia, Barcelona Institute of Science and Technology, 08028Barcelona, Spain; ‡ Johannes Gutenberg-Universität Mainz, 55128Mainz, Germany; § Helmholtz-Institut, GSI Helmholtzzentrum für Schwerionenforschung, 55128Mainz, Germany; ⊥ Department of Physics, UC Berkeley, Berkeley, California94720, United States; ∥ Department of Chemistry, University of Wisconsin, Madison, Madison, Wisconsin53706, United States; ¶ Department of Chemistry, Integrative Biosciences (IBio), Karmanos Cancer Institute (KCI), Wayne State University, Detroit, Michigan48202, United States; # Russian Academy of Sciences, Moscow119991, Russia; ⊗ Molecular Sciences Research Hub, Imperial College London, LondonW12 0BZ, United Kingdom; ∇ Centre de RMN à Hauts Champs de Lyon, Université de Lyon, CNRS, ENS Lyon, Université Lyon 1, 69100Villeurbanne, France; ○ Institut für Analytische Chemie, Universität Leipzig, Linnéstr. 3, 04103Leipzig, Germany; ● Sir Peter Mansfield Imaging Centre, University Park, School of Medicine, University of Nottingham, NottinghamNG7 2RD, United Kingdom; ▼ Institute of Physical and Theoretical Chemistry and Center of Biomolecular Magnetic Resonance, Goethe University Frankfurt, , 60438Frankfurt am Main, Germany; □ Department of Chemical and Biomolecular Engineering, UC Berkeley, and Materials Science Division, Lawrence Berkeley National Laboratory, Berkeley, California94720, United States; ■ International Tomography Center, Siberian Branch of the Russian Academy of Sciences, 630090Novosibirsk, Russia

## Abstract

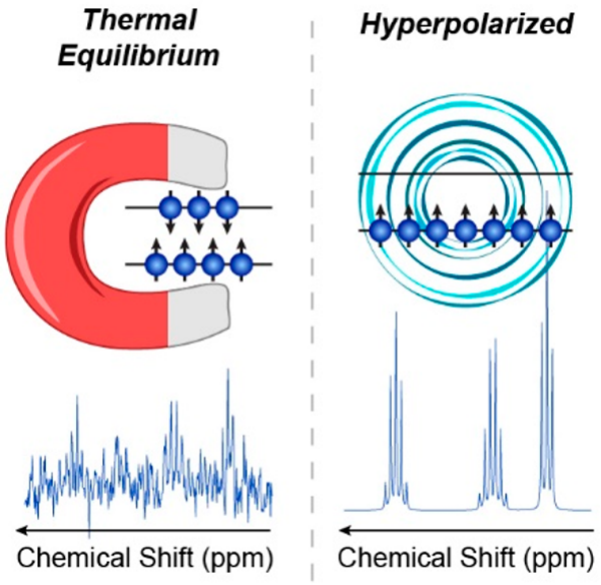

Magnetic resonance techniques are successfully utilized in a broad range of scientific disciplines and in various practical applications, with medical magnetic resonance imaging being the most widely known example. Currently, both fundamental and applied magnetic resonance are enjoying a major boost owing to the rapidly developing field of spin hyperpolarization. Hyperpolarization techniques are able to enhance signal intensities in magnetic resonance by several orders of magnitude, and thus to largely overcome its major disadvantage of relatively low sensitivity. This provides new impetus for existing applications of magnetic resonance and opens the gates to exciting new possibilities. In this review, we provide a unified picture of the many methods and techniques that fall under the umbrella term “hyperpolarization” but are currently seldom perceived as integral parts of the same field. Specifically, before delving into the individual techniques, we provide a detailed analysis of the underlying principles of spin hyperpolarization. We attempt to uncover and classify the origins of hyperpolarization, to establish its sources and the specific mechanisms that enable the flow of polarization from a source to the target spins. We then give a more detailed analysis of individual hyperpolarization techniques: the mechanisms by which they work, fundamental and technical requirements, characteristic applications, unresolved issues, and possible future directions. We are seeing a continuous growth of activity in the field of spin hyperpolarization, and we expect the field to flourish as new and improved hyperpolarization techniques are implemented. Some key areas for development are in prolonging polarization lifetimes, making hyperpolarization techniques more generally applicable to chemical/biological systems, reducing the technical and equipment requirements, and creating more efficient excitation and detection schemes. We hope this review will facilitate the sharing of knowledge between subfields within the broad topic of hyperpolarization, to help overcome existing challenges in magnetic resonance and enable novel applications.

## 1 Introduction

Nuclear magnetic resonance (NMR) spectroscopy, magnetic resonance imaging (MRI), electron paramagnetic resonance (EPR), and other magnetic resonance (MR) techniques address the interactions of nuclear or electron spins with their surroundings and thus provide rich spectroscopic information. The quantitative, real-time information that can be extracted has proven invaluable for many applications ranging from medical imaging to solid materials analysis, from protein structure elucidation to industrial process monitoring, and many others. The fields of physics, chemistry, structural biology, and medicine all bear Nobel Prizes owing to the design and application of NMR and MRI methods (see the Supporting Information for abbreviations and notation for this paper).

Past and present advances in NMR spectroscopy and imaging have had an impact on virtually every field of science and engineering. What makes NMR and related techniques so uniquely powerful is the long times for which nuclear spins produce coherent observable signals after they are excited. By observing the nuclear spins for time scales of seconds or tens of seconds, the spectral resolution that can be obtained is often on the order of hertz or better. Not only does this enable the extraction of spectral fingerprints at high resolution, but it also provides access to processes including translational and rotational diffusion, slow molecular motion, and chemical or physical transformations taking place over relatively long time scales. This advantage arises mostly from the weak interaction of nuclear spins with their surroundings. The magnetic dipole moments of nonzero-spin nuclei are quite small (e.g., compared to that of an electron); hence the molecular processes that couple to the spins to drive relaxation of NMR signals are correspondingly weak.

Unfortunately, the small interaction energies pose a significant drawback: the signals attainable in NMR experiments are notoriously weak. Many other spectroscopic methods involve the interaction of matter with electromagnetic radiation, but the observed transitions might correspond to electronic, vibrational, or rotational degrees of freedom, which are all associated with greater energy than nuclear spin flips. The higher-energy electromagnetic radiation absorbed/emitted by samples probed by spectroscopies such as ultraviolet–visible (UV–vis), Raman, infrared (IR), or microwave (MW), can be detected with orders of magnitude greater sensitivity than the radiofrequency signals produced by nuclear spins in an NMR experiment. The weak interaction of magnetic nuclei with oscillating and static magnetic fields limits the sensitivity in NMR experiments in more than one distinct way, as briefly outlined below.

**Figure 1:**
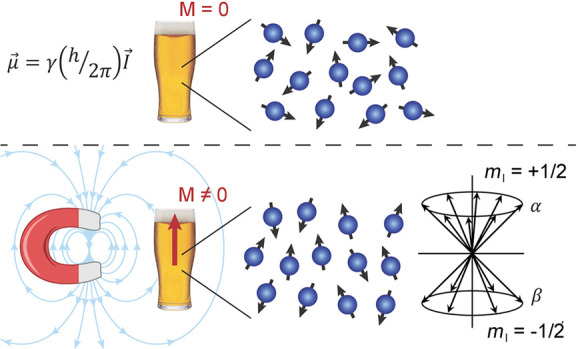
Nuclear magnetization *M* of the object is the net magnetic dipole moment (either of the entire object or of its unit volume) induced by a static magnetic field *B*_0_ as a result of the partial orientation of the magnetic moments *μ⃗* of nuclei. γ is the gyromagnetic ratio of the spin, *h* is the Planck constant.

To illustrate the underlying concepts in relatively simple terms, let us consider an ensemble of nuclei with a nonzero spin. The interaction of the spins with an applied static magnetic field gives rise to the nuclear magnetization of the object (Figure 1), which is inevitably rather small because of the aforementioned weakness of the interaction. Essentially, the magnetic field tends to orient nuclear spins along one direction but fails to efficiently compete with thermal randomization of spin orientation. As a result, the orientational preference of spins in a magnetic field is rather weak.

**Figure 2:**
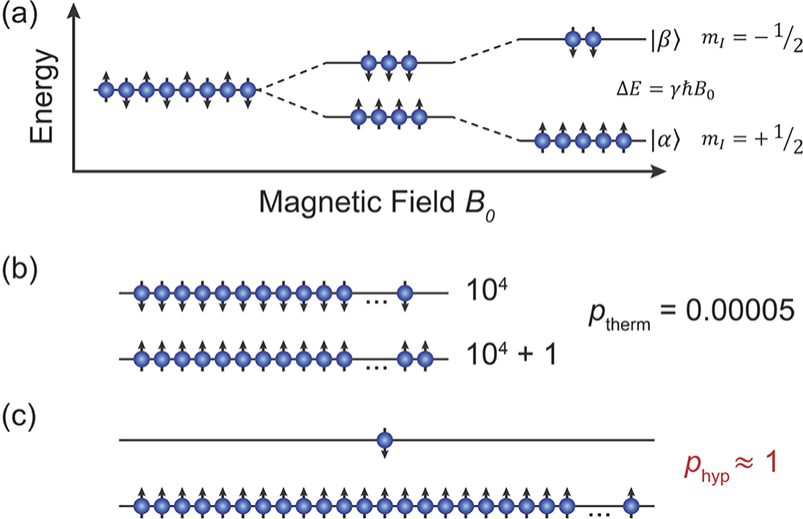
(a) Zeeman energy sublevels of spin-1/2 nuclei with positive γ (e.g., ^1^H) in a static magnetic field. (b) Thermal equilibrium polarization of ^1^H spins at 300 K and *B*_0_ = 14 T (600 MHz ^1^H NMR frequency). (c) An ultimate hyperpolarization (*p*_hyp_ ≈ 1), with almost all spins oriented in the same direction with respect to *B*_0_, which corresponds to an NMR signal enhancement of 2 × 10^4^ relative to the thermal equilibrium state in (b).

In somewhat more quantitative terms, the degree of spin orientation can be characterized by spin polarization. For an ensemble of isolated spin-1/2 species with a gyromagnetic ratio γ in a static magnetic field, the definition of polarization, *p*, is relatively straightforward:
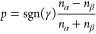
1where *n*_*i*_ is the number of species in state *i*; the states α and β are Zeeman states of the spins that correspond to orientation along or against the external magnetic field (Figure 2a), and sgn(γ) is the sign (+1 or −1) of the gyromagnetic ratio. The intensity of a signal in an NMR spectrum is directly proportional to the polarization of nuclear spins in an object.

Polarization of a thermally equilibrated spin system, *p*_therm_, depends on the strength of the applied magnetic field, the temperature, and the gyromagnetic ratio of the nuclei, γ_n_. At ambient temperatures, the spin polarization of a sample at thermal equilibrium in the magnets of modern NMR instruments is on the order of 10^–4^ to 10^–5^ for ^1^H nuclei (and even lower for other nuclei with smaller γ_n_). The spins in α and β states give opposed contributions to an NMR signal, which means that effectively only one in every 10,000–100,000 spins contributes to the observable signal (Figure 2b). Note that the factor sgn(γ) in eq 1 ensures that *p*_therm_ is always positive, both for species with a positive γ (e.g., ^1^H, ^13^C, etc.; *n*_α_ > *n*_β_) and for those with a negative γ (e.g., e^–^, ^15^N, etc.; *n*_α_ < *n*_β_).

Low spin polarization levels lead to low signal intensities in NMR, and thus to a low sensitivity achievable in the experiments. The low sensitivity precludes the exploitation of magnetic-resonance-based techniques for many applications, such as rapid analytical NMR for use in combinatorial synthesis and screening, chemical and pathogen detection, portable NMR and MRI for in-field use for chemical sensing or emergency medical diagnosis, and localized in vivo spectroscopic studies of targeted tissues. In each of these cases, the limiting factor is the limited amount or concentration of a substance available to probe, the short time scale for which the sample observation is feasible, and/or the impossibility to use a large and delicate superconducting magnet to induce sufficient polarization. Particularly demanding in terms of sensitivity are the modalities of magnetic resonance requiring spatial resolution, such as MRI and in vivo magnetic resonance spectroscopy (MRS). The volume element (voxel) that gives rise to a localized NMR spectrum or to an image pixel in MRI usually represents a tiny fraction of an entire sample/object. In such experiments, a 2-fold isotropic improvement in spatial resolution in all three dimensions decreases the voxel volume 8-fold and thus decreases the signal-to-noise ratio (SNR) in the spectrum or image by almost an order of magnitude.

Significant improvements in sensitivity of NMR-based techniques can thus largely facilitate the implementation of a broad range of novel and advanced applications. Numerous strategies to improve the SNR in magnetic resonance are known and are being explored and exploited, from the implementation of pulsed Fourier-transform NMR with its multiplex advantage enabling faster signal averaging, to the introduction of more efficient inductive detectors, detector miniaturization, noise reduction through the use of cryoprobes and cryogenically cooled signal preamplifiers, to name a few. These technological advances are important, but subsequent progress in this direction is challenging and is likely to be incremental, so that further significant SNR improvements call for different solutions. Another general strategy is to increase the commonly low equilibrium spin polarization by either cooling a sample or placing it in a stronger magnetic field. Both approaches result in a larger sample magnetization and thus provide stronger NMR signals. However, significant sample cooling is not always suitable, while designing NMR magnets with ever-increasing field strength is a slow, technologically challenging, and costly trajectory.^1^

As an alternative to increasing the intrinsically low polarization levels at thermal equilibrium, it is possible to enhance nuclear spin polarization by forcing the spin system into a nonequilibrium state; this phenomenon is known as hyperpolarization. There are a plethora of techniques through which spins can be hyperpolarized far beyond the thermal equilibrium state, and some of them can deliver spin polarization *p* approaching unity. Given that the typical thermal equilibrium polarization levels for nuclear spins do not exceed 10^–4^–10^–5^, achieving *p*_hyp_ ∼ 1 corresponds to signal enhancements of 4–5 orders of magnitude or more (Figure 2c). For all practical purposes, the signal enhancement can be defined as
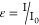
2where I and I_0_ are the intensities of the signal acquired for a hyperpolarized and thermally polarized spin system, respectively, but under otherwise identical experimental conditions. In the case of spin-1/2 species, signal intensity I is directly proportional to polarization *p*, so that I/I_0_ = *p*_hyp_/*p*_therm_, but we use eq 2 as a more general definition of NMR signal enhancement.

Signal enhancements of several orders of magnitude significantly widen the scope of applications of NMR and MRI, even on high-field instruments traditionally associated with a higher SNR and spectral resolution. In addition, there is a more recent yet powerful trend in magnetic resonance: the development of instrumentation and applications in low, ultralow, and even zero magnetic fields.^2,3^ One major incentive behind this line of research is the practical utility of having a compact instrument under a fume hood, or a mobile device that can be brought to an object or living organism under study. However, there are also more fundamental reasons for employing near-zero magnetic fields since certain studies are not feasible with modern high-field NMR instruments. Some prominent examples include NMR spectroscopy with ultrahigh spectral resolution not compromised by magnetic field inhomogeneity,^2,4^ monitoring of chemical reactions in metal containers,^4^ and exploration of the complete (untruncated) molecular spin Hamiltonian.^5^ Clearly, for magnetic resonance at low and ultralow magnetic fields, the advantages of spin hyperpolarization are even more dramatic due to a much larger difference in polarization between thermally equilibrated and hyperpolarized states, compared to high fields.

The central focus of this review is the description of hyperpolarization techniques that enhance spin polarization to overcome the sensitivity limitations of magnetic resonance and its applications. Recent years have witnessed an explosive growth in the number of available hyperpolarization techniques and approaches, in the scope of the emerging applications ranging from materials science to physics, chemistry, biology, medicine, and beyond, and in the number of research groups involved in such studies. With so many recent developments in this research area, it is often hard even for established researchers to navigate the emerging literature, let alone younger scientists and students. Moreover, while a substantial number of reviews on the subject have appeared over the years, the majority of them only cover one or a few individual techniques, either closely interrelated or considered most important and/or advanced. As a result, it may be difficult to evaluate common features, challenges, and potential solutions of existing hyperpolarization approaches. In this review, we attempt to consider the multitude of currently known hyperpolarization techniques together, in a single treatise, aiming to provide a clear unified picture of the entire field of hyperpolarization in magnetic resonance.

In section 2 we discuss in detail the underlying principles, sources, and transfer mechanisms of hyperpolarization. This way we strive to enhance the cross-fertilization between different hyperpolarization techniques, to stimulate creative thinking that will eventually lead to novel hyperpolarization approaches and techniques, and to help systematically consider the common problems and bottlenecks that are often similar across the field of hyperpolarized magnetic resonance. This approach is also expected to provide a different view of the current and emerging advanced applications of hyperpolarization techniques and the problems that they can address. The individual techniques are then discussed in section 3, with appropriate references for the in-depth technical details provided. Their relevant and illustrative applications are highlighted, which mostly cover chemistry and materials science, with a few examples from biomedicine.

In addition to the low polarization of an ensemble of nuclear spins, there are other adverse consequences of the weakness of their interactions with magnetic fields. The coupling of spins to any detector is inevitably weak as it is characterized by the corresponding magnetic moment which, for a given spin polarization *p*, is proportional to the gyromagnetic ratio of the spins. Furthermore, in the case of coil- and resonator-based inductive NMR detectors which are used in the majority of NMR and MRI probes, the detection sensitivity scales linearly with the frequency at which the spins precess in a magnetic field (i.e., the frequencies of the NMR transitions). These frequencies are relatively low, limiting the efficacy of inductive detection. Major efforts are currently devoted to the development of alternative sensors able to significantly outperform traditional inductive detectors in terms of NMR detection sensitivity. Since advanced and emerging NMR techniques involving novel sensors often utilize spin hyperpolarization, the efforts to improve detection sensitivity will also be addressed to some extent. The field of spin-based electronics (spintronics), which relies on the changes in spin states for information processing (e.g., data storage and manipulation), will be mentioned only inasmuch as the spin polarization processes in solid materials are concerned. The production of nonequilibrium and entangled spin states based on the principles of spin hyperpolarization will, without doubt, have a major impact on the development of spintronics, quantum computing, and related fields. Finally, a major application of hyperpolarization to nuclear targets^6^ for particle-accelerator experiments is briefly mentioned in this review.

The number of papers on hyperpolarization published annually is currently experiencing an exponential growth, with more than 6,400 studies published before 2023 according to Web of Science. In this review, we focus on work published in the last 10–15 years, yet key earlier publications introducing important concepts and experimental developments are included to provide a more complete picture of the field.

## 2 SPIN HYPERPOLARIZATION - PRINCIPLES, SOURCES, AND SOURCE-TO-TARGET TRANSFER MECHANISMS

### 2.1 What Is Spin Hyperpolarization?

One of the key reasons for preparing a spin system in a hyperpolarized state is to have a higher SNR in spectroscopic or imaging applications. The goal here would be to achieve the signals in a magnetic resonance experiment that are significantly stronger than those available under thermal equilibrium conditions. With that in mind, for the purpose of this review we qualitatively define a hyperpolarized system of nuclear spins as *a nuclear spin system that is not at thermal equilibrium, which can give rise to a useful enhancement of signals in an NMR experiment*.

We realize that definition of a “useful” enhancement, and thus a delineation between a hyperpolarized and a nonhyperpolarized system, can be subjective and may need further elaboration. For the simple case of an ensemble of spin-1/2 nuclei considered above, a reasonable requirement would be that the achieved increase in polarization, ***|****p*_hyp_**|** – *p*_therm_, is larger than *p*_therm_, where *p*_therm_ is the polarization *p* of spins under thermal equilibrium (*p*_therm_ > 0, see eq 1), and *p*_hyp_ is that of the hyperpolarized spin state. In other words, hyperpolarization should at least double the NMR signal compared to thermal equilibrium. In SNR-limited acquisitions, this would reduce the experiment duration by 4-fold, which is certainly useful. At the same time, an exact quantitative definition of *p*_hyp_ is not of paramount importance as the whole idea behind hyperpolarization is to deliver signal enhancements that are large enough to be useful in a magnetic resonance experiment, and preferably, enhancements by several orders of magnitude.

It is important to note that the considerations presented above apply only to an ensemble of isolated spin-1/2 nuclei; in cases involving coupled spin systems, nuclei with spin *I* > 1/2, or systems that evolve under environmental changes, varying spin dynamics, or chemical interactions, it is more difficult to define polarization. Often the terms polarization and magnetization are used interchangeably. This is notably the case for isolated spin-1/2 nuclei, for which Zeeman order (i.e., polarization) always corresponds to magnetization. However, only rank-1 polarization moments correspond to magnetization, whereas nuclei with spin >1/2 or coupled spin systems support higher-order polarization moments. For instance, for nuclei with spin *I* = 1 such as ^14^N, which have three energy sublevels, an overpopulation of the middle sublevel is not associated with any spin magnetization, and yet such a spin state can result in a major NMR signal enhancement.

Here, we briefly dwell further on the definition of polarization in more technical terms. Polarization is closely linked to the concept of “spin order”, and we define a system as polarized if the density operator that describes the state of the spin system is anisotropic, i.e., it varies upon rotation of the reference coordinate frame. The degree of polarization depends on the degree of anisotropy. “Spin order” is commonly used as a qualitative term, but represents a quantity related to the entropy of the spin system; it is zero when entropy is maximized, and tends to unity when the system is in a pure quantum state.^7,8^ There are specific types of spin order that are commonly encountered such as Zeeman order and dipolar order; Zeeman order represents the degree of polarization of the spins along the applied external magnetic field, while dipolar order describes the degree of polarization of the spins with the magnetic fields generated by neighboring spins. An interested reader can find an in-depth discussion of this matter elsewhere.^8^

Hyperpolarization has been around since the dawn of NMR, and since that time has found an ever-widening range of applications. Hyperpolarization techniques range from simple to sophisticated ones. A spin system can be hyperpolarized, for example, by allowing the sample to reach thermal equilibrium at a given magnetic field, followed by suddenly reducing the strength of the field to the value at which an actual NMR experiment is performed. Until the nuclear spins have relaxed to their new thermal equilibrium state, the polarization exceeds that at thermal equilibrium, and the spins in the sample can remain hyperpolarized. This sample “prepolarization approach” is particularly useful for NMR at zero and ultralow magnetic fields where equilibrium spin polarizations and the resulting NMR signal intensities are essentially negligible.

The strength of the interaction of spins with a static magnetic field is proportional to the gyromagnetic ratio (γ_n_) of a nucleus, and the same applies to *p*_therm_. For instance, γ(^1^H)/γ(^13^C) ≈ 3.976, so that *p*_therm_(^1^H) is ca. 4-fold larger than *p*_therm_(^13^C). This difference in polarization between nuclear spins of different isotopic species means that in many cases there is an internal source of hyperpolarization in a system. If spin state populations are redistributed among spin sublevels in such a way as to enhance the signal-to-noise ratio for the transition between a particular pair of sublevels,^9^ this would be one more example of hyperpolarization. Interestingly, this encompasses many routine NMR experiments in which signals of low-sensitivity low-γ nuclei (e.g., ^13^C, ^14^N, etc.) are enhanced by transferring polarization from a coupled nucleus with higher initial polarization, often a proton. For instance, in a ^13^C{^1^H} NMR experiment performed on a liquid with proton decoupling (either gated or not) by continuously irradiating the ^1^H spins with a radiofrequency magnetic field, such polarization transfer is induced by cross-relaxation processes, with ^13^C NMR signal enhancement of up to a theoretical maximum of I/I_0_ = 1 + 0.5γ(^1^H)/γ(^13^C) ≈ 3.^10^ There are also techniques that do not rely on relaxation processes for efficient manipulation of polarization. Such polarization transfer is ubiquitous in solid-state NMR,^11^ including nuclear quadrupole resonance (NQR),^12^ where cross-polarization (CP) from protons is used to enhance signals of lower-γ nuclei. In NMR of quadrupolar nuclei with half-integer spin in solids, the central transition in the NMR spectrum can be enhanced by selective population inversion of the sublevels corresponding to the satellite transitions. Examples from solution-state NMR include INEPT (insensitive nuclei enhanced by polarization transfer) and DEPT (distortionless enhancement by polarization transfer) and similar sequences.^13^

The polarization transfer schemes listed above are quite illustrative and useful in practice, but the signal enhancements they provide are limited to a value comparable to the ratio of the γ_n_ values of the involved nuclei. (At the same time, we note that for polarization transfer between concentrated and dilute spins, the possibility to achieve higher polarization gains has been discussed in the literature.^14^) Fortunately, diverse hyperpolarization techniques capable of providing dramatically larger sensitivity improvements are currently available. Dynamic nuclear polarization (DNP) replies on electron–nuclear polarization transfer, to utilize the significantly higher polarization of electron spins. It is used, often in combination with magic angle spinning (MAS), to probe protein structure and dynamics, as well as to study biological assemblies such as membrane proteins, ribosomes and viral capsids.^15,16^ DNP is also employed to enhance signals for surface characterization, a method known as dynamic nuclear polarization surface-enhanced NMR spectroscopy (DNP SENS), to investigate hybrid materials, organometallic surface species, and metal–organic frameworks.^17^ Yet another DNP technique known as dissolution DNP (*d*DNP) has been used to study protein structure and interactions in the solution state, for real-time monitoring of rapid chemical processes, and for producing hyperpolarized biomolecules for in vivo imaging.^18−20^ Human lung imaging has been performed using inhaled noble gases (e.g., ^129^Xe and ^3^He) hyperpolarized via optical pumping.^21^ Metabolites have also been hyperpolarized for preclinical biomedical imaging using parahydrogen-induced polarization (PHIP),^22,23^ a technique based on the use of H_2_ in its singlet nuclear spin state which is called parahydrogen. In addition, PHIP has been employed to determine hydrogenation reaction mechanisms of homogeneous^24^ and heterogeneous^25^ catalytic processes. The scope of molecules that can be polarized using parahydrogen is further broadened by the technique known as signal amplification by reversible exchange (SABRE).^26^ A hyperpolarization technique that has been used to extract information on reaction mechanisms involving free radicals is chemically induced dynamic nuclear polarization (CIDNP). The photochemically triggered version of this method (photo-CIDNP) is also employed to characterize the surface structure of peptides and proteins by hyperpolarizing amino acid residues.^27,28^ These and other powerful hyperpolarization techniques are presented in greater detail in subsequent sections of this review. Hyperpolarization techniques have also been used for applications beyond chemistry and biology, such as spin-polarized targets for solid-state physics and particle accelerator experiments.^6,29,30^ Exciting possibilities such as nuclear spintronics^31^ and searching for exotic spin-dependent interactions^32^ are on the horizon.

The advantages of spin hyperpolarization for achieving dramatic signal enhancement in magnetic resonance are vast and impressive; however, utilization of hyperpolarized spin systems in magnetic resonance experiments presents a number of challenges as well. The biggest limitation in the use of hyperpolarization is in its inherently transient nature: once a hyperpolarized state is produced it begins to decay due to spin relaxation. The entropy increase over time drives the highly ordered system back toward its more disordered thermal equilibrium state. The time scale of this process can vary greatly and depends on many factors such as the type of nuclei, temperature, the state of matter, molecular structure, magnetic field, etc. The topic of nuclear spin relaxation is covered in much greater detail elsewhere.^33−35^ When we speak about nuclear spin relaxation, more often than not we refer to the spin–lattice relaxation time constant, *T*_1n_, or the time constant describing the loss of signal after radiofrequency (RF) excitation, *T*_2n_ or *T*_2n_* (the true relaxation rate of an ensemble of spins, and the observed relaxation rate caused by a distribution of magnetic fields experienced by the spins), although coupled spins can support states with vastly different relaxation times. Usually high-spin-order states of many correlated spins have reduced relaxation times, since the relaxation rates of the individual spins contribute to the overall state relaxation,^36^ but this is not always the case.

In highly symmetric molecules, specific configurations of nuclear spins can support long-lived spin states (LLSSs) which are immune to certain relaxation mechanisms, and can have relaxation times that greatly exceed *T*_1n_.^37,38^ There are many examples of LLSSs in coupled spins systems, and understanding the molecular symmetry is crucial to predicting them.^39^ Not only do LLSSs provide a means by which spin polarization can be stored for times longer than *T*_1n_,^40,41^ but, as discussed in section 2.2.3, for some hyperpolarization techniques they are a crucial and necessary consequence of the hyperpolarization process. One rather remarkable example of a LLSS is associated with molecular hydrogen, H_2_. Its pair of coupled ^1^H spin-1/2 nuclei give rise to four eigenstates of the nuclear spin wave function: one singlet (*I* = 0) and three triplet (*I* = 1) spin states. An entire subfield of hyperpolarization experiments relies on enriching and utilizing parahydrogen. This state of enriched singlet order and depleted triplet order, known as a singlet–triplet imbalance, is a highly nonequilibrium spin state. The spin state of parahydrogen is a long-lived spin state which is immune to the key intrapair relaxation mechanisms (dipole–dipole, chemical shift anisotropy, and spin rotation). Parahydrogen is thus stable in the gas phase for long times (from hours to weeks and possibly longer, depending on conditions^42^) and is a very versatile source of hyperpolarization (section 3.11). However, it cannot provide signal enhancement in an NMR experiment without additional chemical manipulations. Therefore, unlike LLSS of molecules with an imperfect symmetry, the *I* = 0 singlet spin state of parahydrogen is not itself hyperpolarized according to our definition of hyperpolarization as it cannot give rise to NMR signals without first breaking the equivalence of its two protons, and it cannot be used for temporarily and reversibly storing hyperpolarization in an NMR experiment.

It is evident that an intrinsically transient hyperpolarized spin state can be useful only while it lasts. Therefore, prolonging the lifetime of hyperpolarization and its efficient utilization within the available time window are the issues of paramount importance in this context. The impact of relaxation on hyperpolarization-enhanced NMR can be considered in three distinct stages of the experiment:

(1) *Preparation* – Many hyperpolarization procedures are designed to avoid relaxation of the sample competing with the polarization process, which otherwise would limit the final polarization. However, this is not always the case–some hyperpolarization methods rely on establishing thermal equilibrium under conditions that give high nuclear spin polarization (i.e., low temperature and high magnetic field), and in these cases it is beneficial to enhance the rate of relaxation during the hyperpolarization process to make polarization buildup faster.
(2) *Modification* – Once created, the transient nature of the polarization limits our ability to carry out procedures such as transporting the samples to a different location, chemically modifying the sample, or purifying the samples, for instance, for use in a biological system. Therefore, sufficiently rapid procedures must be developed and implemented, and this represents a significant portion of ongoing research in the field.
(3) *Utilization* – In applications one cannot study processes or dynamics with time scales much greater than the time scale on which relaxation occurs, unless the sample can be repolarized or fresh hyperpolarized material supplied to the system repeatedly or continuously.

One focus of this review is on the measures that have been taken to circumvent the challenges presented by the transient nature of spin hyperpolarization. Examples include generating hyperpolarization in situ (i.e., at the point of detection) to avoid relaxation losses during transport,^43,44^ choosing/changing the state of matter to take advantage of slower relaxation, for example, in cryogenically cooled solids,^45,46^ transferring the hyperpolarization to slower-relaxing lower-γ nuclei with the possibility to transfer back to a high-γ nucleus for a more sensitive readout,^47,48^ and temporarily storing hyperpolarization in long-lived spin states.^41,49−51^

Once at the point of detection, more tricks can be played to extend the time for which the hyperpolarized sample produces observable signals, or to extract the maximum information possible within the short time period. It is common to use a number of small and/or variable flip-angle RF pulses to only slightly deplete the hyperpolarized state for observation by each individual pulse without completely destroying it.^52^ One can also use flip-back pulses to return the magnetization to be along the field axis between scans so that it relaxes with the longitudinal relaxation time *T*_1n_ rather than *T*_2n_*.^53,54^ Another strategy for hyperpolarized samples which undergo chemical conversion or exchange between pools is frequency-selective excitation of one component to maximize signal without perturbing other components.^55^ Ultrafast acquisition methods have been introduced to allow two-dimensional NMR spectra to be obtained with hyperpolarized samples.^56,57^ There is also a great wealth of advanced imaging strategies to extract the maximum amount of information from hyperpolarized samples before the signal is lost, which are covered in greater detail elsewhere.^58^

### 2.2 Sources of Hyperpolarization

**Figure 3:**
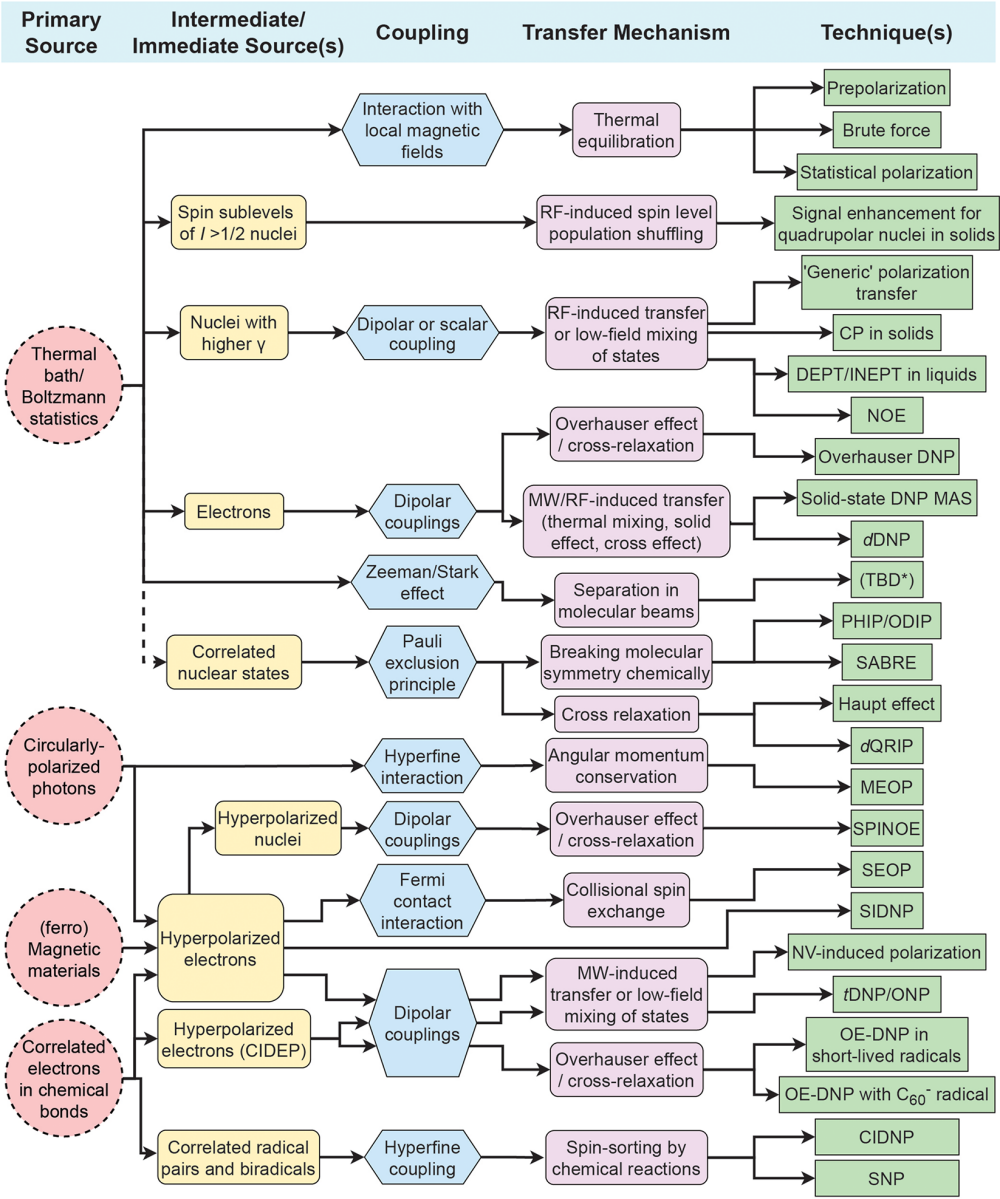
Diagram categorizing hyperpolarization techniques into a hierarchy: (1) the primary source of polarization; (2) the intermediate source which provides a bridge from the primary source to the nuclear spins; (3) the type of coupling that allows polarization transfer to the nuclear spins; (4) the mechanism that allows polarization transfer to occur; and (5) the resulting technique.

Most hyperpolarization techniques that have been demonstrated to date rely on a polarization source that can act as a reservoir of low entropy with which the nuclear spins can exchange energy to lower the nuclear spin temperature. The common sources of polarization (Figure 3) are the internal degrees of freedom in molecules and materials such as rotational, vibrational, electronic, or nuclear spin states. These sources can be replenished directly by establishing thermal equilibrium characterized by Boltzmann statistics under the experimental conditions, by thermally induced or photoinduced processes in molecular species or materials, or by literally sorting the species in different spin states by chemical reactions or physical interactions. Another powerful source of hyperpolarization is provided by circularly polarized photons^59^ which can induce electronic transitions that lead to electron or nuclear spin polarization upon light absorption.

The polarization sources directly couple to the target nuclear spins, yet in some cases polarization is passed sequentially via one or even several intermediate sources until it reaches the target spins. The exchange of entropic energy, and hence polarization transfer, is mediated by these couplings. For prepolarization of nuclear spins under high *B*_0_/*T* conditions (where *B*_0_ is the magnetic field and *T* is temperature), the spins are coupled to the thermal bath through the interaction with local magnetic fields. When electron spins are the polarization source, the coupling to nuclei is via the hyperfine interaction, either dipole–dipole or Fermi contact. When other nuclear spins are the polarization source, the coupling is via dipole–dipole or scalar (J) couplings. Quantum-rotor-induced polarization and hyperpolarization processes involving nuclear spin isomers of molecules (NSIM) are based on the coupling of nuclear spin states to rotational states due to the Pauli exclusion principle.

Different mechanisms can be used to allow the polarization transfer between the source and the target nuclei. Incoherent (i.e., stochastically fluctuating) processes can lead to polarization exchange between the source and the target nuclei, such as relaxation and cross-relaxation of spins. Alternatively, this can be done coherently (i.e., in a controlled manner) by adiabatically exchanging spin-state populations via irradiating the sample with a microwave/radiofrequency field or bringing it to a relatively low magnetic field in which spins become strongly coupled.^60^ Other methods include spin-selective chemical reactions, reactions/interactions that break the symmetry of an otherwise unobservable spin state, and collisional polarization transfer by spin exchange. Polarization transfer can also be mediated, due to angular momentum conservation, upon absorption of polarized photons and upon interaction between spins and chiral environments.

These concepts are illustrated in Figure 3 that shows the polarization sources, coupling type, and polarization transfer mechanisms, and links these to specific hyperpolarization techniques. While this picture is useful, it is not possible to make it exhaustive and to reflect every possible scenario. One of the exceptions not included in the figure is algorithmic cooling,^61^ which makes use of tailored polarization transfer schemes between nuclear spin states with different relaxation times to achieve *p* > *p*_therm_. Another example is enrichment of nuclear spin isomers of symmetric molecules through NSIM-dependent physicochemical interactions such as light-induced drift, adsorption onto surfaces, exchange reactions, selective photolysis, etc.^62^ Enriched NSIM of symmetric polyatomic molecules are yet to become a useful source of hyperpolarization, with only one demonstration of this exciting possibility reported to date.^63^

#### 2.2.1 Thermal Bath of the Surrounding Lattice

The hyperpolarization methods described in this section rely on building up polarization via Boltzmann statistics, and there are four distinct cases: (1) polarizing the target nuclear spins directly at low temperature and/or high magnetic field, and detecting under different conditions; (2) using pulse sequences to manipulate spin state populations of a single nucleus type species to enhance a specific transition; (3) transferring spin order to the target nuclei from coupled nuclei with higher polarization; (4) transferring spin order to the target nuclei from unpaired electrons.

##### 2.2.1.1 Polarizing the Target Nuclear Spins Directly at Low Temperature and/or High Magnetic Field

**Figure 4:**
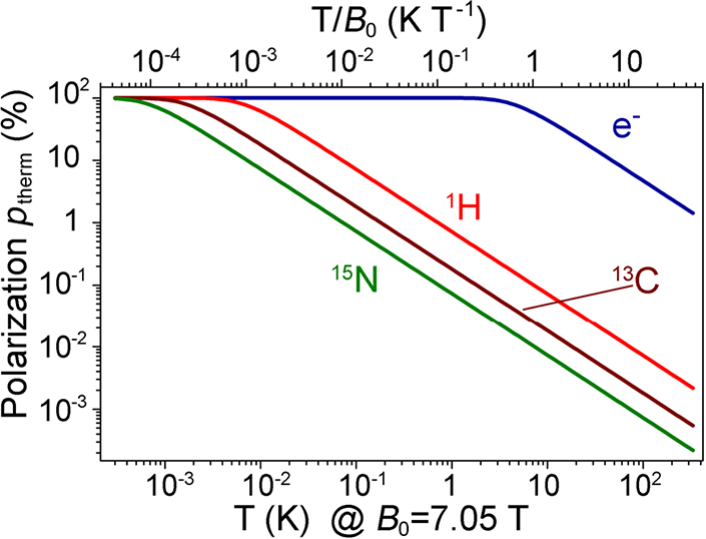
Polarization of an ensemble of the named spin-1/2 particles at thermal equilibrium (*p*_therm_) as a function of *T*/*B*_0_. Horizontal scale is additionally provided in temperature units for *B*_0_ = 7.05 T (ω_n_(^1^H)/2π = 300 MHz, where ω_n_ is the nuclear Larmor frequency).

As described in section 1, polarization of spins with a gyromagnetic ratio γ depends on magnetic field (*B*_0_) and temperature (*T*). The thermal equilibrium polarization of an ensemble of spin-1/2 particles is given by
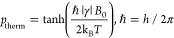
3where *k*_B_ is the Boltzmann constant and *h* is the Planck constant. The polarization of four different types of spin-1/2 particles is shown as a function of *T*/*B*_0_ in Figure 4. High levels of polarization can be generated by allowing the system to reach thermal equilibrium under high *B*_0_/*T* conditions, but the system is not hyperpolarized as long as it remains at thermal equilibrium. However, if the sample is rapidly warmed, or the magnetic field is reduced for detection before the spins relax, then the spin system is hyperpolarized. For low- or zero-field experiments^64,65^ this is called “prepolarization”, and the spins can be polarized in a permanent magnet followed by physical shuttling to low field,^66^ or polarized in situ using an electromagnet that can be switched off prior to detection.^67^

A more extreme case of prepolarization is “brute force” hyperpolarization wherein a sample is cryogenically cooled at high field, and is then given time to reach thermal equilibrium polarization before being rapidly warmed for detection.^68^ The challenge is that, under the cryogenic polarizing conditions, the relaxation times and thus the time it takes the spins to become thermally equilibrated can become quite long, but this method can lead to proton polarization approaching 1% at high field and liquid helium temperature. This topic is discussed in greater detail in section 3.1. We note that the concept of cooling the sample to lower the entropy of the nuclear spins is the reverse of the magnetocaloric effect (or nuclear demagnetization refrigeration).^69,70^

##### 2.2.1.2 Using Pulse Sequences to Manipulate Spin-State Populations of a Single Nucleus Type Species to Enhance a Specific Transition

**Figure 5:**
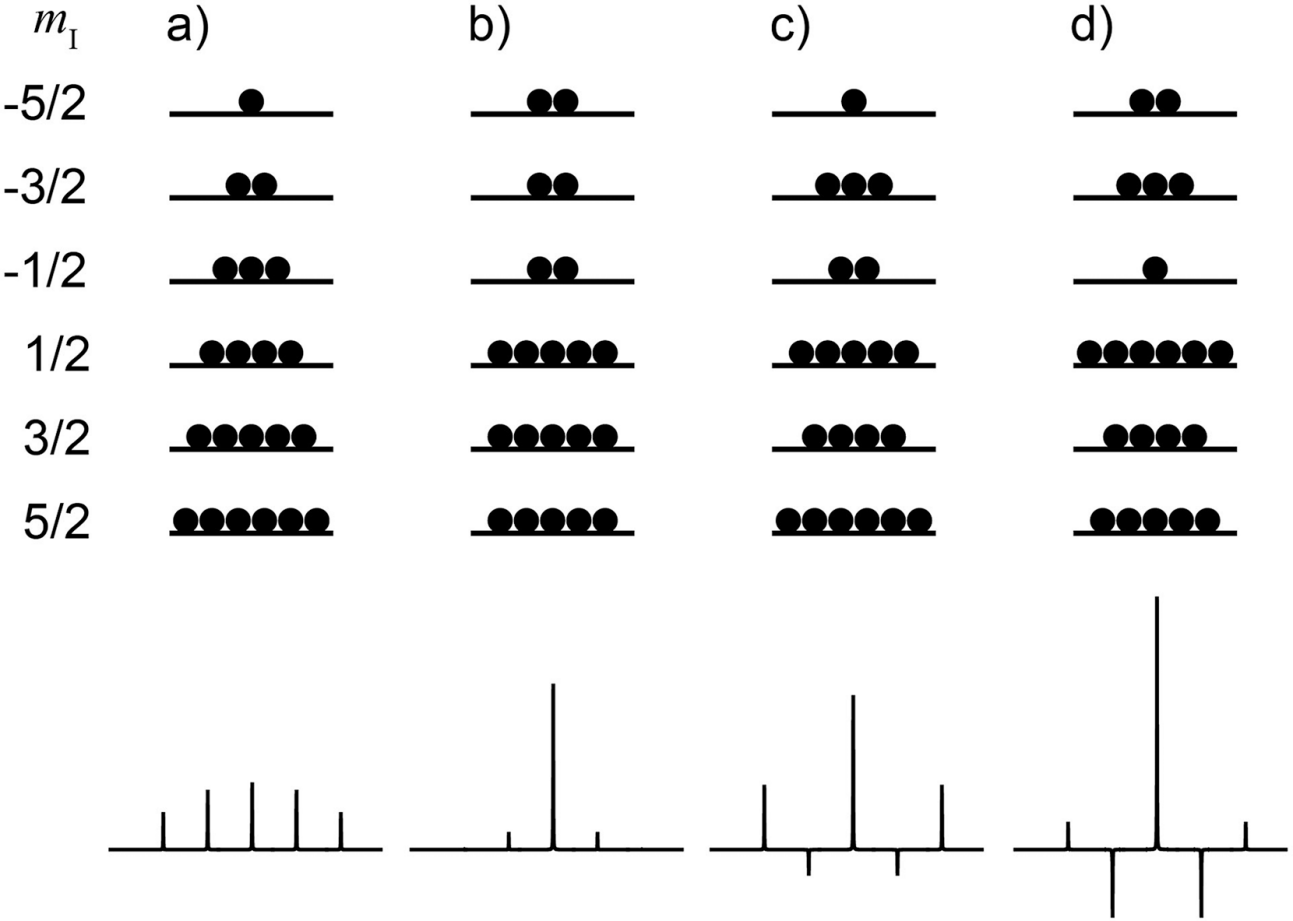
Illustration of spin state populations (exaggerated) of an ensemble of spin-5/2 nuclei, with black circles representing populations of the *m*_I_ magnetic sublevels, respectively. Beneath the energy level diagrams, NMR stick spectra simulated using the SpinDynamica^74^ software package for Mathematica are shown. The four cases correspond to (a) thermal equilibrium, (b) saturation of just the outer transitions, (c) inversion of the populations for the states involved in the ±3/2 ↔ ±1/2 transitions, and (d) inversion of the populations of the ±5/2 ↔ ±3/2 transitions, followed by inversion of the populations of the ±3/2 ↔ ± 1/2 transitions. The line intensities are shown without taking into account that satellite transitions are usually much broader than the central transition.

Hyperpolarization may also result from population transfer between spin states of quadrupolar nuclei to increase the intensity of a transition between certain magnetic sublevels.^9,71,72^ This method requires restricted molecular motion (e.g., a solid-state sample) so that the quadrupolar coupling is not isotropically averaged by rapid molecular reorientation,^73^ and hence the transition frequencies between magnetic sublevels with magnetic quantum number *m*_I_ are different, and can be addressed individually using “soft” RF pulses or adiabatic fast passage through corresponding resonances for level population inversion. The populations may be spread over a number of energy levels giving rise to multiple spectral lines, but it is possible to shuttle populations between the states to selectively enhance specific transitions. The concept^72^ is illustrated in Figure 5 which shows relative populations of spin sublevels and the corresponding NMR line intensities calculated using SpinDynamica.^74^ Signal enhancements of up to 2*I* (e.g., 5 for a spin-5/2 nucleus) can be achieved this way, provided that the outer (satellite) transitions are completely inverted adiabatically and in the proper order and that the central transition is then probed with a selective 90° flip-angle pulse. This is a somewhat unique type of hyperpolarization experiment since the entropy of the nuclear spin system is not reduced, but the spin temperature of specific transitions is lowered.

##### 2.2.1.3 Transferring Spin Order to the Target Nuclei from Coupled Nuclei with Higher Polarization

A common type of signal enhancement scheme for both solid- and solution-state NMR is polarization transfer experiments in which the polarization source is coupled spins of another kind that have higher polarization. In solid-state NMR, cross-polarization^75^ (CP) pulse sequences can be employed to transfer polarization via dipolar couplings from protons to lower-γ X nuclei, giving, in principle, an enhancement factor of γ(^1^H)/γ(X), where γ(X) is the gyromagnetic ratio of the X nucleus.^76^ This has the additional benefit that the experiment can be repeated at a rate governed by the higher-γ nuclear *T*_1n_ (e.g., that of protons) rather than the *T*_1n_ of the X nucleus, which is often favorable for faster signal averaging. Similar experiments exist for solution-state samples, although rapid molecular tumbling leads to averaging of the dipolar couplings, hence scalar J-couplings are used to mediate the polarization transfer. The INEPT^77^ and DEPT^13^ pulse sequences are examples of coherent transfer schemes which rely on using RF pulses to drive polarization transfer between the nuclei of different types.

An alternative is to use the nuclear Overhauser effect (NOE), which is a change in intensity of a nuclear spin transition when another nucleus is saturated or perturbed.^10^ This differs from coherent polarization transfer since the effect is mediated by dipole–dipole cross-relaxation processes between the coupled spin pairs. More specifically, in the simplest case of a two-spin system the effect relies on a difference between the relaxation-driven zero-, single-, and double-quantum transition probabilities, which depend strongly on the distance between the coupled nuclei. This distance dependence gives rise to the widespread application of NOEs for molecular structure determination in, for example, NOE spectroscopy (NOESY) experiments.^78^ Decoupling of a coupled nucleus from another that is being detected can also involve saturating nuclear transitions which can give rise to an NOE of the nucleus being detected. We note, however, that application of continuous RF irradiation during signal acquisition only (inverse gated decoupling) does not itself generate hyperpolarization on the detected nuclei, despite having the appearance of enhancing the intensity of an NMR line. In this case the transitions are not enhanced beyond their thermal equilibrium level, and the increase in intensity is brought about by the collapse of a multiplet into a single line. At the same time, gated (RF irradiation prior to signal acquisition) and regular decoupling do induce the NOE-based spin hyperpolarization.

Experiments which use thermal polarization of coupled protons (or any higher-γ nucleus) are fairly ubiquitously employed in NMR experiments and are not usually referred to as hyperpolarization. This is probably because these methods essentially give enhanced NMR signals “for free”, with little to no additional equipment or experimental intervention required, and because the achievable signal enhancements are modest. Hence, these techniques will not be discussed in detail in this review since they are already well-established; many commercial NMR instruments allow this type of hyperpolarization to be implemented at the touch of a button, and there is little ongoing development or possibility for cross-fertilization with other hyperpolarization techniques.

##### 2.2.1.4 Transferring Spin Order to the Target Nuclei from Unpaired Electrons

Significantly higher thermal polarization levels can be achieved for electrons: they are polarized ∼658-fold more than protons under the same typical conditions (Figure 4). Therefore, the “brute force” approach described above for nuclei requires less severe conditions for polarizing electron spins. Thermally polarized electron spins often serve as a useful and powerful source of hyperpolarization for nuclear spins. The Overhauser effect discussed above in the context of nuclear–nuclear interactions was, in fact, first predicted^79^ and demonstrated^80^ for electron–nuclear interactions, and was later extended to nuclear–nuclear systems.^81^ Hyperpolarizing nuclear spins in the solution state by saturating electronic transitions of unpaired electrons to transfer the large thermal polarization from electron spins via the Overhauser effect is called Overhauser DNP, and is covered in more detail in section 3.2.

Overhauser DNP is a somewhat unique DNP technique since it relies on cross-relaxation processes to generate nuclear spin polarization. By contrast, the majority of solid-state DNP experiments now use coherently driven electron–nuclear polarization transfer, which is somewhat analogous to the coherent nuclear–nuclear polarization transfer experiments discussed above (e.g., cross-polarization). For polarization transfer, the EPR line of an unpaired electron needs to be saturated, and this requires high-power microwave sources which were not originally available for high-field/high-frequency experiments. In a breakthrough that has since been referred to as “the renaissance of DNP”,^82^ a gyrotron was employed as a microwave source to allow DNP to be performed at 5 T.^83^ Before that, DNP was performed at 1.4 T, which limited the available electron polarization. The unpaired electrons in paramagnetic species which are crucial for these experiments are provided by doping the samples with free radicals or paramagnetic metal ions or, alternatively, by generating free radicals in situ.^84^ Electron spin angular momentum and nuclear spin angular momentum interact in materials via electron–nuclear hyperfine interaction, either static or dynamic; this coupling enables the transfer of energy between the electron and nuclear spin baths via several specific mechanisms.^85^ Solid-state DNP experiments are usually carried out at high field under cryogenic conditions and can lead to some of the highest NMR signal enhancements achievable via hyperpolarization, since near-unity thermal polarization of electron spins can be readily established near liquid helium temperatures in magnetic fields available with commercial instrumentation (Figure 4). These DNP techniques are discussed in more detail in section 3.3 and section 3.4.

Dynamic nuclear polarization is a general term that encompasses many techniques in which the polarization of unpaired electron spins is transferred to coupled nuclear spins.^82^ Note that this does not include CIDNP (sections 3.6 and 3.8), and the name is a misnomer that stemmed from an initial misinterpretation of the mechanism that gives rise to CIDNP signals that was later corrected.^86^

The techniques mentioned above use thermal equilibrium polarization of electron spins, either at ambient (Overhauser DNP) or cryogenic (solid-state DNP and dissolution DNP) conditions as the hyperpolarization source for nuclei, but it is not difficult to surmise that one could also use hyperpolarized electrons for this as well. Such existing possibilities are introduced below in section 2.2.2.

##### 2.2.1.5 Statistical Polarization

Finally, we mention a somewhat separate case of “statistical polarization.” Consider for example an ensemble of *N* independent spin-1/2 nuclei. If this ensemble is prepared in a random fashion, by randomly preparing each nucleus in either spin-up or spin-down state with respect to an arbitrarily chosen quantization axis, then an excess of either spin-up or spin-down nuclei on the order of *N*^0.5^ is expected in each realization of the ensemble. This kind of random polarization enables magnetic resonance noise spectroscopy and even imaging,^87^ which becomes particularly advantageous for small-*N* samples. This “do nothing” polarization approach is especially effective in the case of single-spin NMR (see, for example, ref (88) and references therein), where the signal size in the case of stochastic polarization is essentially equal to that of a fully polarized sample. Interestingly, since stochastic polarization makes use of a transient nonequilibrium state, it fits our heuristic definition of hyperpolarization given above.

#### 2.2.2 Correlated and Hyperpolarized States of Electron Spins in Molecules and Materials

As mentioned above, large thermal polarization of electron spins is much easier to achieve compared to nuclei (Figure 4). At the same time, it may be advantageous in practice to use hyperpolarized electron spins since, unlike achieving high equilibrium polarization levels, major hyperpolarization of electron spins is available at significantly milder or even ambient conditions, i.e., without cryogenic sample cooling and application of high magnetic fields.

Similar to nuclear spin hyperpolarization, creating hyperpolarization of electron spins also requires a suitable source. As shown below, in many (albeit not all) cases, the source of electron hyperpolarization can be traced back to the correlated states of electron spins. Indeed, the state of electron spins that participate in the chemical bonds in molecules and materials is governed by the Pauli exclusion principle. As a result, a chemical bond in a molecule is usually associated with an overall singlet (spin-zero) state of the paired electrons shared by two bonded atoms.

As electronic energies associated with chemical bonds are much larger than the energies associated with other molecular degrees of freedom, the energy separation of different electronic spin states (e.g., the singlet and the triplet states of a molecule) is significantly larger than the energy of the thermal bath. Stable molecules residing in a ground electronic state are thus ultimately enriched with the corresponding spin state of the electrons under any realistic conditions. The correlated electron spin states of molecules and materials are thus omnipresent in nature and can serve as a useful primary source of spin hyperpolarization for developing various experiments. This can be achieved in a number of different ways:

(1) Photoexcitation of an electronic transition in a molecule or material results in a number of spin-selective interconversions within and between the electronic singlet and triplet manifolds of molecular states (see sections 3.5, 3.6, 3.9, and 3.10). For instance, photoexcitation of a molecule possessing a singlet ground state S_0_ is most efficient if the singlet electronic state is retained, with the molecule ending up in an excited singlet state S_n_ (n > 0). Subsequent internal conversion within the S_n_ manifold and intersystem crossing (ISC) to the manifold of triplet states, T_n_, can proceed efficiently in a solid, liquid or gas phase. ISC can be spin-state-selective and thus can produce hyperpolarization of electron spins in paramagnetic (e.g., triplet) molecules. As a result, EPR spectra of photoexcited triplet molecules in molecular glasses and sometimes in solution show dramatically enhanced signal intensities (sections 3.5 and 3.9). Chemical transformations of hyperpolarized triplet molecules in solution can yield free radicals that inherit hyperpolarization in the spin states of their unpaired electrons (section 3.5).
(2) The same principles apply for other multiplicities of the ground state of molecules and materials as well. A prominent example is the ground electronic triplet state of nitrogen-vacancy (NV) centers in the diamond (section 3.10). Upon a photoinduced transition to an excited triplet state followed by spin-state-selective ISC between the triplet and the singlet manifolds, the NV center efficiently ends up in one out of the three electron spin sublevels of the triplet ground state. This highly overpopulated state of the electron spins at the NV center can serve as the source of hyperpolarization for magnetic nuclei in the diamond^51^ and, potentially, in molecules in contact with the diamond surface (section 3.10).
(3) In thermal or photochemical transformations of molecules in solution or in a solid material, free radical species can be often produced. As mentioned in (1) above, the latter can inherit hyperpolarization from their precursors if it is available. There is, however, a different mechanism that can produce hyperpolarized free radicals from a nonhyperpolarized precursor (e.g., a triplet, a doublet or a singlet) molecule. Such effects are addressed and utilized by a family of techniques collectively known as chemically induced dynamic electron polarization (CIDEP), which is related to CIDNP fundamentally and terminologically, and is equally unrelated to DNP. CIDEP can provide high levels of electron spin hyperpolarization via a number of different mechanisms. CIDEP effects (section 3.5) are observed in photoinduced (or sometimes thermal) chemical reactions which generate paramagnetic species such as molecular triplet states, radical pairs, radicals or biradicals, which often carry a significant load of electron spin hyperpolarization at easily attainable experimental conditions. As mentioned above, the primary source of electron hyperpolarization in these cases is the correlation of electron spins in the starting molecule or material, which is converted to hyperpolarization of unpaired electron spins either directly or via intermediate nonequilibrium spin states.

Hyperpolarized spins of unpaired electrons in paramagnetic species can in their turn become a major source of hyperpolarization for nuclear spins. One prominent example involving hyperpolarization transfer from paramagnetic triplet species to nuclei are the techniques known as triplet-DNP (*t*DNP) and optical nuclear polarization (ONP). The transfer of polarization from electrons to nuclei is spontaneous in ONP, while it is induced by microwave or radiofrequency irradiation in *t*DNP (section 3.9). Overhauser-type DNP driven by electron–nuclear cross-relaxation can transfer polarization from hyperpolarized electron spins of short-lived^89^ or stable^90,91^ free radicals to nearby nuclei.

Paramagnetic species produced in chemical and photochemical reactions are also responsible for nuclear spin polarization generated by various mechanisms of CIDNP. However, the underlying mechanisms may be different in that they rely on spin sorting by a chemical reaction rather than on direct hyperpolarization transfer from unpaired electrons. The CIDNP techniques (primarily, photo-CIDNP) are considered in detail in sections 3.6 and 3.8.

Hyperpolarization of electron spins is also possible using a source that is different from the correlated spin states of electrons in a chemical bond. In particular, spin-polarized photons can be used as a powerful primary source of hyperpolarization for other types of spins. Indeed, in the early days of magnetic resonance it was realized that transient excited-state electrons created by light absorption could provide for high levels of electron spin polarization.^92^ This is widely utilized for hyperpolarizing the unpaired electron spins of alkali-metal atoms in a vapor, which is useful for many practical applications When combined with cross-relaxation to nuclei, “optical pumping” of nuclear spins was born, in which hyperpolarized electrons of Rb vapor are utilized as a source of nuclear hyperpolarization of noble gases^93^ (section 3.13). This and related methods are discussed further in section 2.2.4. Besides, in 1968 it was reported that light absorption in semiconductors can produce hyperpolarized electrons via the optical absorption selection rules associated with polarized light excitation.^94^ A host of condensed matter physics phenomena resulted from such studies^95,96^ and are discussed further in section 3.14.

The examples above do not exhaust the list of possible ways to produce hyperpolarized electrons. In the late 1950s and early 1960s, “hot” conduction electrons^97,98^ were invoked to provide for Overhauser-type cross-relaxation processes. Here cross-relaxation driven by, e.g., the electron–nuclear hyperfine interaction, connects the nuclear spin temperature to the difference between the electron spin temperature and the temperature corresponding to their mean kinetic energy under applied DC current. This effect induced via the application of a static electric field was employed to polarize ^115^In, ^121^Sb, and ^123^Sb nuclei in indium antimonide.^98^ An electric current flowing through an oriented ferromagnetic domain can also inject polarized electrons across its interface with a nonmagnetic metal or semiconductor, and lead to nuclear hyperpolarization via the (Overhauser type) DNP driven by cross-relaxation. This effect, spin-injected DNP (SIDNP), has been proposed^99^ and demonstrated^100,101^ via electroluminescence in quantum heterostructures. Further optimization of this approach, including the use of higher-temperature ferromagnetic semiconductors, suggests the tantalizing possibility of using high electron polarizations to provide significant nuclear hyperpolarization in bulk at modest temperatures.

#### 2.2.3 Correlated States Involving Nuclear Spins in Molecules

There exist many kinds of nonequilibrium spin states in molecules and materials that do not immediately lead to an observation of an enhanced NMR signal, and are thus not classified in this review as hyperpolarized. Some such states can even give no observable NMR signal at all. However, such states can still be very useful in the context of signal enhancement in magnetic resonance because they can be employed as hyperpolarization sources if appropriate additional chemical or physical manipulations with the system or object are performed. One example is the correlated state of electron spins in chemical bonds considered in the preceding section. Another possibility considered below involves correlated states of nuclear spins and their coupling with molecular rotational wave functions, a topic frequently studied in the context of vibrational–rotational spectra of homonuclear diatomic molecules. While the energies associated with nuclear states are small compared to rotational energies, the rotational state restricts what kind of nuclear spin state the molecule can occupy. This results in equilibrium nuclear spin order governed by Boltzmann factors containing rotational energy. A representative example which is recurringly encountered throughout this review is the hydrogen molecule, H_2_. Because of its molecular symmetry, only certain combinations of its rotational and nuclear spin wave functions are allowed.

Molecular hydrogen (H_2_) is the most renowned and remarkable example of a molecule existing as a mixture of its different modifications (NSIM), namely orthohydrogen (*I* = 1) and parahydrogen (*I* = 0).^42^ Parahydrogen has proven to be a versatile source of strong hyperpolarization (section 3.11). Interestingly, the highest polarization is achieved when pure parahydrogen is used, which itself has no nuclear spin and thus gives no NMR signal. According to our definition of hyperpolarization, we do not consider parahydrogen a hyperpolarized system. Yet, it possesses a highly correlated spin state that can be exploited to produce hyperpolarization. This requires breaking the symmetry of an H_2_ molecule in a suitable chemical (catalytic) process.

The nonmagnetic but highly ordered spin state of parahydrogen can be used as a source of hyperpolarization of nuclear spins in two different ways. Both require breaking the symmetry of the H_2_ molecule, which is essentially equivalent to a chemical activation of the H_2_ molecule by breaking the H–H chemical bond. One approach^102^ (PHIP) operates via incorporation of both H atoms of a parahydrogen (p-H_2_) molecule into one product molecule by catalytically hydrogenating a suitable unsaturated precursor. This way the initial singlet nuclear spin state of p-H_2_ is inherited by the product molecule and evolves there into a hyperpolarized spin state. Another^26^ (SABRE), sometimes referred to as non-hydrogenative PHIP, is based on bringing a suitable substrate and an activated p-H_2_ into temporary contact on the metal atom of an Ir-based metal complex in solution, with spin evolution taking place in this temporary assembly to generate hyperpolarization. Continuous exchange of coordinated H_2_ and substrate with their free counterparts in solution replenishes the polarization source and leads to a buildup of polarization on the free substrate. These techniques are detailed further in section 3.11.

Importantly, in addition to H_2_ and its heavy isotopologue D_2_, other symmetric molecular gases also have two or more NSIM. They attract significant interest, both in fundamental science and for some practical applications. First and foremost, the existence of NSIM (formerly referred to as allotropes in the case of H_2_) is one of the key cornerstones and the early predictive triumphs of quantum mechanics.^103^ In modern science, of significant interest are the properties of NSIM, including NSIM interconversion processes^62^ and their behavior upon phase transitions,^104,105^ the NSIM-related selection rules in molecular spectroscopy^106,107^ and upon chemical transformations,^108^ and more. Notably, the reactivity of H_2_O with trapped diazenylium ions (N_2_H^+^) was reported to be different for para- (*I* = 0) and orthowater (*I* = 1).^109^ In addition, NSIM of polyatomic molecules (e.g., H_2_O, NH_3_, H_2_CO, CH_4_) attract significant attention in astrophysics and astrochemistry research.^110,111^ It is often assumed that the ortho–para ratio (OPR) of these molecules in outer space can remain unchanged for millions and even billions of years, so that spin temperature could reflect the conditions of the formation of comets, dark molecular clouds and protostars. This issue, however, remains controversial at present, awaiting more detailed studies of the properties of nuclear spin isomers of symmetric polyatomic molecules.

**Figure 6:**
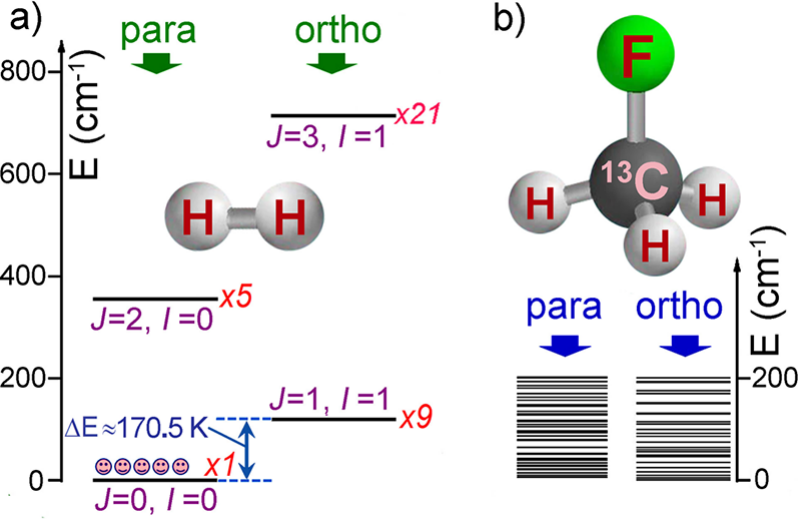
(a) Energy level diagram for parahydrogen and orthohydrogen (0–800 cm^–1^ range). The values of nuclear spin (*I*), rotational quantum number (*J*), and degeneracy (*xN*) are shown for each level. At 20.3 K, the lowest state of p-H_2_ is populated almost exclusively (pink circles). (b) Energy level diagram for ^13^CH_3_F (0–200 cm^–1^ range) shown on the same vertical scale for comparison. Reproduced from ref (112). Copyright 2017 The Authors. Published by the Royal Society of Chemistry.

**Figure 7:**
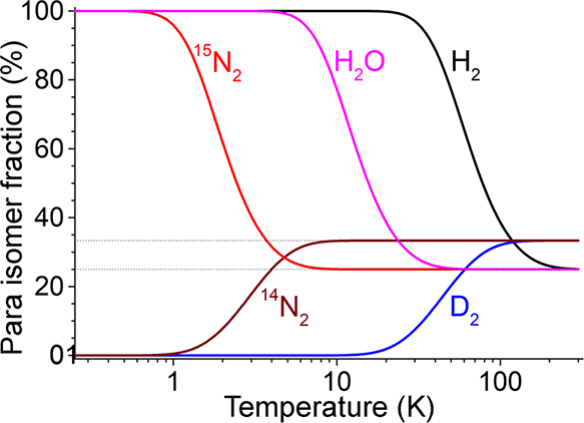
Temperature dependence of the equilibrium fraction of para NSIM for several small symmetric molecules: H_2_ (*I* = 0), D_2_ (*I* = 1), H_2_O (*I* = 0), ^14^N_2_ (*I* = 1), ^15^N_2_ (*I* = 0). Note, however, that these dependences were calculated assuming that molecular rotation is not hindered significantly in the entire temperature range presented, which for molecules other than hydrogen isotopologues would require special conditions (e.g., molecular beams, cryogenic matrices, endofullerenes).

Of interest in the context of this review is that the highly correlated nuclear spin states of NSIM of symmetric polyatomic molecules, much like that of parahydrogen mentioned above, can potentially yield major NMR signal enhancements. However, rotational energy quanta of polyatomic molecules are significantly smaller compared to that of a much smaller and lighter H_2_ molecule (Figure 6). The smaller energy separations between the rotational states of polyatomic molecules can decrease even further when molecular rotations become restricted upon sample condensation/freezing (except for certain molecules trapped in inert cryomatrices and in part for solid CH_4_). As a result, while cryoenrichment of p-H_2_ is facile and also works reasonably well for orthodeuterium (o-D_2_, *I* = 0,2), it is not generally applicable to NSIM enrichment of polyatomic molecules (Figure 7).

**Figure 8:**
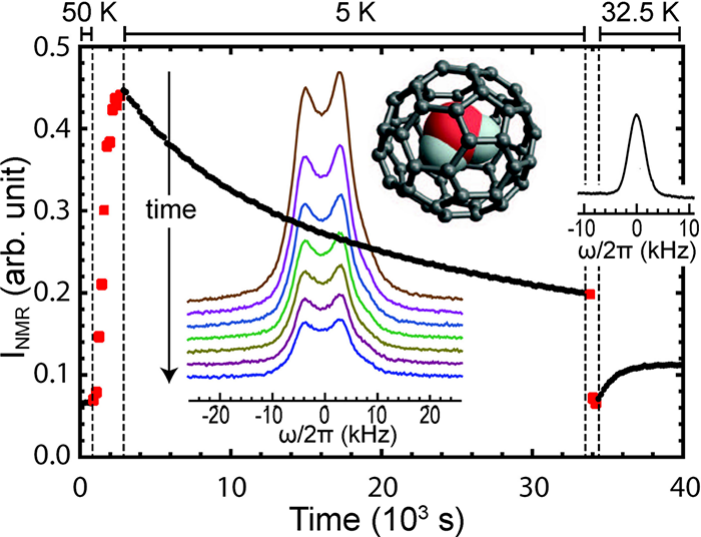
Integrated ^1^H NMR signal intensity as a function of time during an experiment involving two temperature jumps, on a sample of H_2_O@C_60_ (molecular structure is shown in the inset). The sample temperature is reported at the top of the graph. Values corresponding to a constant temperature are shown as black dots, while those obtained during a temperature change are reported as red squares; the measurements were performed at time intervals of 180 s. The sequence of colored ^1^H NMR spectra in the inset were recorded at 5 ± 0.1 K, taken at intervals of 2.25 h after cooling from 60 K. The first spectrum (top) was taken after waiting for 30 min in order to allow thermal equilibration of the equipment. A characteristic spectrum at 32.5 K is also included in the right-hand side of the figure. Reproduced from ref (113) with the permission of AIP Publishing.

Therefore, NSIM research with polyatomic molecules, including NSIM-based hyperpolarization, requires alternative techniques of enrichment. While several such techniques are known today (see also section 2.2.5),^62^ most of them are unable to produce the quantities and enrichment levels sufficient for any NMR experiment. One successful approach for water NSIM enrichment and exploration by NMR is based on an individual H_2_O molecule incarcerated in a C_60_ fullerene cage, H_2_O@C_60_ (Figure 8).^113^ The useful property of this system is that the C_60_ host can be used both as a solid powder and dissolved in a liquid, while the entrapped H_2_O molecule essentially remains under gas-phase conditions. The first NSIM interconversion studies with endofullerenes were in fact done with H_2_@C_60_.^114^ However, the enriched NSIM of endofullerenes with symmetric guest molecules are impossible to use in a chemical reaction required to break the symmetry of the incarcerated guest, and thus cannot be widely used for hyperpolarization purposes.

**Figure 9:**
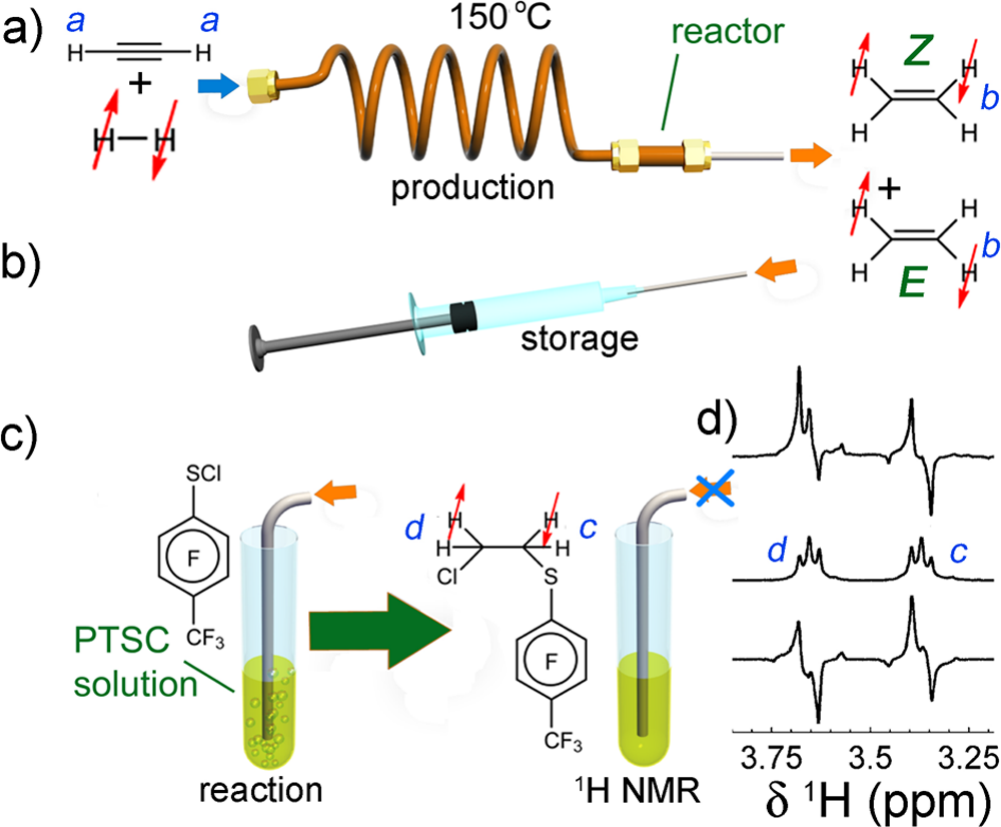
Schematics of the experimental setup and the sequence of events during chemical enrichment of ethylene NSIM and their use for NMR signal enhancement. (a) Acetylene is hydrogenated with parahydrogen by passing their mixture heated to 150 °C through the packed bed of Pd/TiO_2_ solid catalyst. The Z and E labels indicate different mutual positions of the two p-H_2_-derived H atoms in the ethylene molecules. (b) The product ethylene gas is collected and optionally stored for various time periods in a gastight syringe. (c) The ethylene gas from the syringe is bubbled through a solution of perfluoro(*p*-tolylsulfenyl) chloride (PTSC) in the NMR tube residing inside a 7 T NMR magnet, thus leading to the formation of the ethylene-PTSC adduct and breaking the symmetry of ethylene. (d) ^1^H NMR spectra observed immediately after bubbling the produced ethylene through the PTSC solution (top and bottom spectra) and 20 s after interrupting the bubbling (middle spectrum). The bottom spectrum was acquired with double-quantum filtering. Reproduced with permission from ref (63). Copyright 2013 WILEY-VCH Verlag GmbH & Co. KGaA, Weinheim.

In contrast, enrichment of ethylene (C_2_H_4_) NSIM was achieved by hydrogenating acetylene (C_2_H_2_) with parahydrogen over a Pd/TiO_2_ catalyst (Figure 9).^63^ Unlike in studies where the strong hyperpolarization is observed upon catalytic addition of parahydrogen to an unsaturated nonsymmetric substrate (sections 3.11.2 and 3.11.3), ethylene is a symmetric molecule itself, and no NMR signal enhancement can be observed. However, the spins of the parahydrogen-derived H atoms can remain partially correlated after they are incorporated in ethylene, yielding NSIM enrichment. To reveal this, the ethylene was used in a subsequent reaction to produce an asymmetric product which broke the equivalence of the pH_2_-derived protons, thereby converting the correlated spin state of ethylene into enhanced NMR signals. To date, this is still a rather rare example of a study which demonstrates hyperpolarization induced by NSIM of a freely rotating symmetric polyatomic molecule. Hopefully, further advances in NSIM enrichment protocols will change this in the near future.

Similar correlated states may arise for molecules with no overall symmetry if they possess a fragment with a local symmetry such as the methyl CH_3_ rotor. CH_3_ groups in molecules possess the necessary symmetry properties for the three coupled protons to support long-lived spin states, and to act as a source of hyperpolarization. Analogous to the case of the H_2_ molecule, the methyl group symmetry leads to a coupling of the rotational eigenstates to the spin eigenstates, and if the methyl rotor is relatively sterically unhindered, the energy separation between rotational states can be sufficient to allow for overpopulation of the lowest-energy rotational/spin state by cooling.^115^ In this way the nuclear spin polarization can be increased far beyond what would be achievable without the coupling of the spatial and spin wave functions, although this method is limited in applications to just a handful of molecules with the necessary steric properties of the methyl rotor. Hyperpolarization via cooling of a methyl moiety was originally known as the Haupt effect, and is now referred to as quantum-rotor induced polarization (QRIP). The possibilities provided by the correlated states of the CH_3_ rotor are considered further in section 3.12.

#### 2.2.4 Circularly Polarized Photons

Photons, the quanta of light, are spin-1 entities. Polarized spin angular momenta of light are available if a light beam has circular polarization. Circularly polarized photons are easily produced by passing linearly polarized light through a quarter-wave plate if the axis of the birefringent material (i.e., a perpendicular crystal axis system, each with different index of refraction) is at 45° to the polarization axis of the incident light. The resulting phase difference between the two perpendicular components of the light creates either left-handed or right-handed polarized light. Light emitted from a laser is usually (but not always) linearly polarized; however, diode-array lasers and laser light passing through fiber optics may have to be passed through an optical cube first to ensure only linear polarized light enters the quarter-wave plate. Furthermore, optical lenses may have to be used to increase the beam diameter and thereby to reduce the power density to ensure the quality of the resultant circular polarization. Photons are thus extremely easy to spin-polarize, and when such a photon is absorbed, conservation of the angular momentum may result in a change of the spin state of the light-absorbing substance: an atom, a molecule, or a material. This effect is used in several important practical and emerging applications (section 3.14).

One example is the metastability-exchange optical pumping (MEOP) technique suitable for hyperpolarizing nuclear spins of ^3^He gas (section 3.13). A weak RF discharge is used to promote ^3^He atoms from the ground to the metastable excited state, followed by photoexcitation with circularly polarized light. Angular momentum transfer from a photon to spin degrees of freedom of a He atom upon light absorption creates nuclear spin polarization for the metastable ^3^He states which is subsequently passed to the pool of ^3^He in the ground state in collisional spin exchange processes.^116^

Polarized photons can similarly polarize electrons of paramagnetic species (section 2.2.2). An illustrative example is an optically pumped atomic magnetometer, in which circularly polarized light is used to excite the D_1_ spectral transition line of atoms in alkali metal (e.g., ^87^Rb) vapor, thereby creating a hyperpolarized state of their electron spins.^117^ A laser beam orthogonal to the pumping beam then probes the optical rotation of linearly polarized light to measure the magnitude of a magnetic field experienced by the electrons. The same hyperpolarization principle is employed in atomic clocks widely used as precise frequency standards based on the accurately known frequencies of hyperfine transitions in ^133^Cs and ^87^Rb.

One of the practical applications of circularly polarized light as a source of hyperpolarization of electron spins important here is the optical pumping of rubidium vapor for hyperpolarization of nuclear spins of noble gases such as ^3^He, ^83^Kr, ^129^Xe. Hyperpolarization of rubidium electrons is transferred to the noble gas nuclei in briefly formed collisional complexes in the gas phase by means of spin exchange driven by the electron–nuclear hyperfine interaction (section 3.13). This spin-exchange optical pumping (SEOP) technique provides a remarkable and illustrative example of combining several polarization sources in succession to achieve the final goal. Indeed, first, the spins of photons are polarized by imposing circular polarization on a laser beam; this hyperpolarization is then transferred to unpaired electrons in Rb atoms upon light absorption, followed by the gas-phase-collision-mediated transfer to nuclear spins of a noble gas (e.g., ^129^Xe), and the sequence can be continued even further, e.g., by dissolution of hyperpolarized ^129^Xe gas and hyperpolarization transfer to the ^1^H nuclei of a solvent^118^ or by admitting hyperpolarized ^129^Xe gas to porous materials with subsequent hyperpolarization transfer to various nuclei on a solid surface.^119,120^ Such transfer can be mediated by the spin-polarization-induced nuclear Overhauser effect (SPINOE) for both cases, while for solids other transfer mechanisms (e.g., cross-polarization, thermal mixing) are also possible.^119,121^ The resulting polarization of, e.g., ^1^H nuclei of a solvent or a solid material surface can be passed further on to other types of nuclei using various polarization transfer mechanisms.

Dissociation of molecules under circularly polarized light is used to produce photofragments with polarized electron and nuclear spins, which can be used for a number of applications, from magnetometry to experiments in atomic and nuclear physics.^122^

#### 2.2.5 Spin-Sorting Phenomena

The populations of different nuclear spin states in thermal equilibrium under most realistic conditions are close to being statistical; for instance, there is an essentially equal number of spin-1/2 nuclei in α and β states in an applied magnetic field at room temperature (Figure 4). An attractive possibility would be to separate the ensemble of species (e.g., atoms or molecules) into groups based on the spin states of the species involved, e.g., with species carrying spins in the α state collected in the first group, and species with β spin state in the second one. Here, Maxwell’s demon certainly comes to mind, but unlike the separation of hot and cold atoms or molecules in that thought experiment, spin sorting is a reality.

One possibility stems from the classical Stern–Gerlach-type experiments^123^ in atomic and molecular beams which were used in the early days of quantum mechanics to prove that spatial orientation of angular momentum is quantized. Particles with a nonzero magnetic moment passing through a strong magnetic field gradient are deflected from a straight path in different directions depending on the orientation of their nuclear spin with respect to the applied field. This leads to a separation of a beam into 2*I*+1 separate beams with different *I*_*z*_ quantum numbers, achieving a clean sorting of particles based on their spin state, i.e., a decrease in entropy.

**Figure 10:**
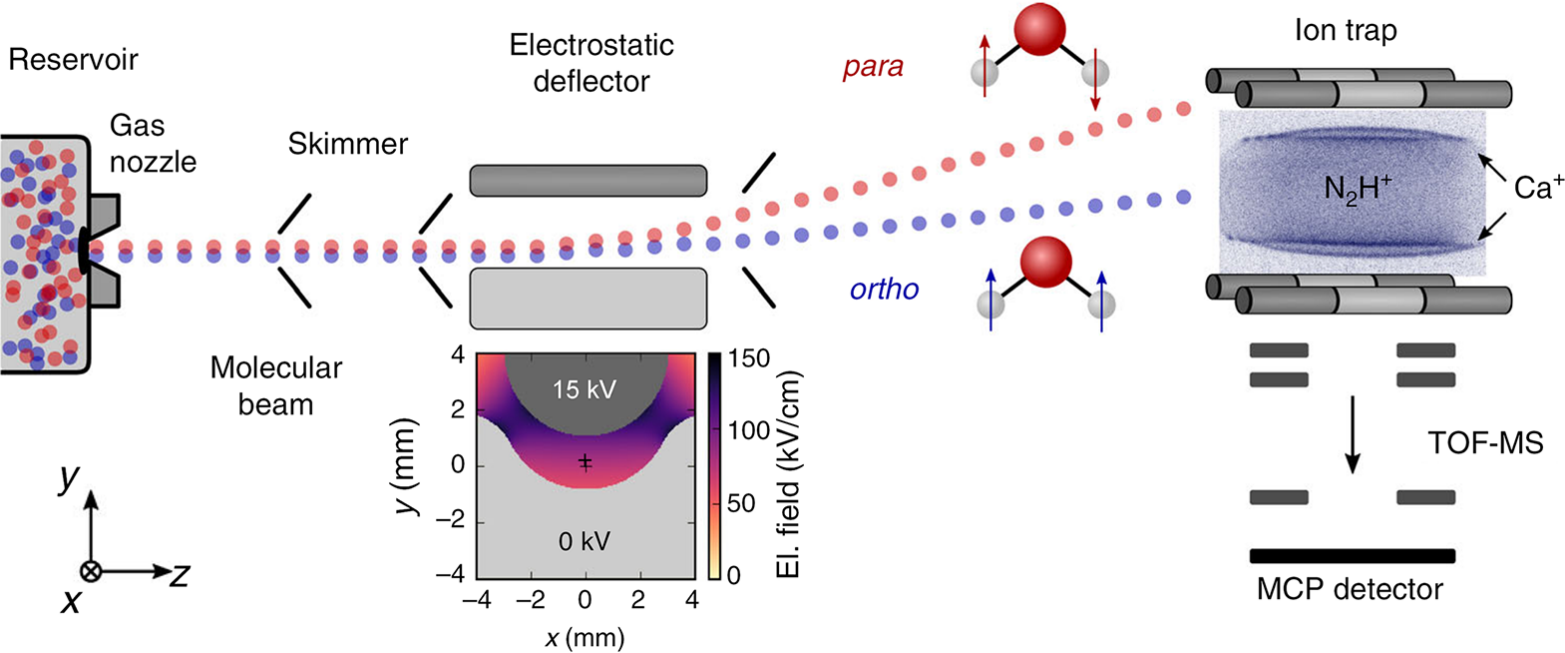
Schematics of the experimental setup for NSIM separation using Stark effect. A pulsed molecular beam of water molecules seeded in argon emanates from a room-temperature reservoir through a pulsed gas nozzle and passes an electrostatic deflector. The inhomogeneous electric field inside the deflector (shown in the inset) spatially separates parawater and orthowater molecules. After the deflector, the beam is directed at an ion trap containing a Coulomb crystal of Ca^+^ and sympathetically cooled N_2_H^+^ reactant ions. The products and kinetics of reactive collisions between N_2_H^+^ and H_2_O are probed using time-of-flight mass spectrometry (TOF-MS). Reproduced from ref (109). Copyright 2018 The Authors. Published by Springer Nature under CC BY license.

Furthermore, the beam separation approach was used relatively recently to produce beams of H_2_O and CH_4_ with hyperpolarized nuclear spins. Because of the necessarily low densities of molecular beams under collision-free conditions in such experiments and a fast relaxation of nuclear spins in the gas phase, these experiments are not immediately suitable for NMR. However, there is more to these experiments than just nuclear spin polarization when symmetric molecules such as H_2_O and CH_4_ are involved: the separation of their NSIM is achieved as well. For instance, ortho- and parahydrogen mentioned above could be separated in such an experiment, but this would be impractical as nowadays enrichment of parahydrogen can be achieved far more readily (section 3.11.1). However, in contrast to NSIM of H_2_, NSIM of symmetric polyatomic molecules are not easily accessible, but could be of major interest for expanding the range of available sources of nuclear hyperpolarization in the experiments similar to those performed with parahydrogen (sections 3.11.2, 3.11.3, and 3.11.4). Successful separation of ortho- and parawater has been achieved in cold molecular beams using the gradient of a magnetic field (the Zeeman effect),^124^ and was later extended to acetylene and methane.^125^ Ortho- and parawater separation was also successfully achieved in cold molecular beams using the gradient of an electric field^109^ (the Stark effect) (Figure 10). However, due to the low density of molecular beams required for such separation in either technique, the quantities produced (∼10^8^–10^10^ molecules/s) are rather low, and the use of NSIM of polyatomic molecules in the NMR context would require further improvement of the technique to provide much larger quantities of enriched NSIM than it is possible today.

An alternative and rather advanced approach for sorting NSIM of symmetric molecules is the so-called light-induced drift (LID) technique.^62^ It is based on a velocity-selective rovibrational photoexcitation within a Doppler-broadened absorption line of one of the NSIM, while other one or more NSIM serve as the buffer gas. A small (ca. 0.1–1%) change in the collisional cross-section upon photoexcitation produces a slow directional drift of the photoexcited NSIM, leading to a spatial separation of different NSIM in an illuminated cell. LID has been successfully used to enrich NSIM of CH_3_OH^126^ and isotopologues of CH_3_F^62^ and C_2_H_4_.^127^ The achieved enrichments (e.g., 1–3% at 1 Torr^127^) are sufficient for detection and characterization with laser spectroscopy, including the measurement of NSIM interconversion rates. However, it remains to be seen whether or not this can be sufficient for signal enhancement in NMR experiments.

Hyperpolarization by spin sorting is also encountered in the broad field of CIDNP and related phenomena (sections 3.6, 3.7, and 3.8) where nuclear spins are sorted by a chemical reaction. For instance, when photoexcitation of a suitable chromophore molecule results in the formation of, for instance, a spin-correlated pair of neutral radicals in the triplet electronic state, recombination or disproportionation of the radical pair is not possible unless/until the pair converts to a singlet spin state. The dynamic singlet–triplet (S-T) interconversion of the radical pair is driven by the local magnetic fields experienced by its unpaired electrons, which includes hyperfine interactions with nuclear spins of the participating radicals. Thus, the rate of S-T interconversion can depend on the orientation of nuclear spins, and those nuclear spin orientations that cause faster T → S conversion will end up in the product molecule faster, while radical pairs with other nuclear spin orientations will take longer to interconvert, so that nuclear polarization of the opposite sign can be lost in radicals due to relaxation or end up in different products produced by secondary reactions of the radicals escaping from the initial radical pair. For reversible reactions such as electron transfer processes between electron donor and acceptor molecular species, significant levels of hyperpolarization may be accumulated this way on the starting species without much chemical conversion or degradation. These and other possibilities are discussed in more detail in sections 3.6 and 3.8. The spin-sorting process by a chemical reaction can be further assisted externally; in the technique of stimulated nuclear polarization (SNP)^128^ the T → S interconversion in a transient radical pair or a biradical is accelerated by flipping one of the electron spins with an applied microwave magnetic field, which is possible if the EPR spectra of the two paramagnetic species are sufficiently different owing to the difference in their g-factors or nuclear hyperfine patterns. Furthermore, the interconversion can be accelerated selectively for a chosen orientation of nuclear spins by selectively exciting the respective component of an EPR spectrum with a resolved hyperfine pattern. This leads to acceleration of the chemical reaction for that particular nuclear spin state selectively, and hence leads to spin polarization in the reaction product. This possibility is discussed in section 3.7.

Electron spins are also polarizable by spin sorting or spin filtering processes. This is used in particular for producing polarized targets in atomic and nuclear physics, for example, using an atomic beam source to produce a beam of hydrogen atoms which is then separated into two beams with opposite electron spin orientations after passing through a strongly inhomogeneous magnetic field.^122^ Spin sorting or spin filtering is also widely employed in the field of spintronics. This includes (but is far from being limited to) the injection of polarized electrons across an interface between a ferromagnet and a metal or semiconductor and the SIDNP effect mentioned earlier.

Yet another way to polarize electrons is to pass them through chiral environments. According to the current understanding of this phenomenon termed chirality-induced spin selectivity (CISS),^129−133^ no spin flipping is involved in the process. Instead, this approach relies on a more efficient transmission of the electron with the favored spin orientation (either parallel or antiparallel to its velocity depending on the helicity of the medium) as a consequence of reduction in backscattering, i.e., represents spin sorting or spin filtering. We note, however, that currently the origin of this effect is not fully understood, and alternative explanations are being advanced in the literature.^134^ CISS effect has been observed for both individual chiral organic molecules and their self-assembled monolayers (SAM) on a suitable substrate, for instance oligopeptides, proteins and double-stranded DNA oligonucleotides, as well as for chiral inorganic thin films. Spin filtering has been reported for electron transport through chiral media over distances from a few to a few tens of nanometers. CISS was shown capable of achieving high polarization levels at room temperature; for instance, spin filtering in excess of 85% was reported with helical π-conjugated materials based on supramolecular nanofibers.^135^

There are a number of important implications and potential applications of CISS. For one, it may play an important role in electron transfer in biomolecules and biological systems. In particular, substantial spin polarizations were reported for electron transfer through the helical protein bacteriorhodopsin embedded in its native membrane environment (*p*_e_ ∼ 15%)^136^ and for photoinduced electron transfer in Photosystem I (PSI) (*p*_e_ ∼ 40–80%).^137^ The CISS effect may also provide access to the separation of enantiomers by adsorption on magnetized surfaces^130,138^ as well as enantiomer-selective chemistry in an adsorbed molecular adlayer induced by electron transfer through the underlying CISS-active chiral monolayer.^139^ CISS effect was demonstrated to affect redox chemistry, with an interesting example of spin control of multielectron-transfer reactions is the (photo)electrochemical splitting of water into hydrogen and oxygen. Polarization of electron spins achieved by coating the anode of an electrochemical cell with a chiral layer (e.g., a molecular monolayer, a polymer film, or an inorganic oxide) can facilitate formation of triplet O_2_ (*S* = 1) and suppress recombination of hydroxyl radicals into the side product H_2_O_2_; this results in the decrease of reaction overpotential and an increase in the overall efficiency of H_2_O splitting. A combination of CISS with an artificial molecular motor was implemented to reversibly switch chirality and achieve the associated inversion of spin polarization of transmitted electrons by applying external stimuli,^140^ paving the way to novel spintronic devices.

## 3 HYPERPOLARIZATION TECHNIQUES

In this part of the review, we discuss individual hyperpolarization techniques in more detail, starting with the general underlying principle and the hyperpolarization source(s) involved, and the mechanisms that link the source to the actual spin hyperpolarization. This is followed by a brief description of the instrumentation required to implement the technique and the practical realization of the hyperpolarization procedure. Typical applications of the technique are then illustrated with representative examples, including the common molecular species and/or materials targeted and hyperpolarization levels achieved. Finally, potential promising extensions are described, and the primary unsolved issues that could advance the technique further and broaden the range of its applications are pointed out.

### 3.1 Brute Force

As described in section 2.2.1.1 (eq 3), nuclear spin polarization in thermal equilibrium depends on temperature *T* and the strength of the external magnetic field *B*_0_. A nuclear spin system can be highly polarized by allowing the sample to equilibrate to the thermal (Boltzmann) polarization under cryogenic conditions and/or in a high magnetic field. In many experiments, this brute force approach is used to hyperpolarize samples for low-, ultralow-, or zero-field detection.^65,66,141^ The sample is prepolarized in a large magnetic field supplied either by a permanent magnet array, or an electromagnet, and after shuttling the sample to the lower-field region or switching off the polarizing field, the signal is detected. We will not discuss this straightforward implementation of the brute force approach further, since in these cases the nuclear spin polarization does not exceed that attainable in a regular high-field NMR experiment. To exceed the polarization achievable in a high-field experiment, the sample can be cryogenically cooled. Figure 4 shows the polarization of different spin-1/2 particles as a function of *T*/*B*_0_. After the polarization step, the sample can be extracted from the polarization instrumentation and returned to ambient temperature on a time scale less than the nuclear spin relaxation.

Early experiments originated in the field of condensed matter physics, with the goal of producing large quantities of hyperpolarized ^3^He and ^129^Xe.^142,143^ The advent of medical lung imaging using hyperpolarized noble gases sparked further interest in brute force as a method to produce large quantities of hyperpolarized ^3^He and ^129^Xe.^144,145^ In recent years, experimental efforts have been focused on polarizing small organic molecules, with a particular focus on metabolites.^68,146^ The brute force approach has a number of advantages over other hyperpolarization methods: (1) there is no need for microwave/radiofrequency irradiation of the sample, which reduces equipment complexity and means the sample need not be confined to a cavity; (2) extraneous molecules such as free radicals or catalysts are not required, which circumvents the need for downstream purification and quality assurance; (3) the method is in principle general and applicable to any sample; and (4) homogeneity of the polarizing field is not critical, which means large sample volumes can be used. However, at low temperatures most molecular motion is frozen out, and nuclear spin-relaxation times can become very long. Indeed, long nuclear spin *T*_1n_ times (often tens or hundreds of hours) under the high *B*_0_/*T* conditions have precluded widespread use of the brute force approach.

In contrast to all other methods discussed in section 3 (see Figure 3), brute force does not require any specific mechanism to convert an external source of polarization into nuclear spin polarization; with this approach the nuclear spins are polarized directly.

The instrumentation required for brute force experiments can be relatively simple. A cryostat is needed to cool the samples, and this should be mounted in a high-field magnet. Most experimental demonstrations have employed liquid helium baths vacuum-pumped to lower the temperature to 1–2 K, or dilution refrigerators that can operate down to millikelvin temperatures. After the cooling process and relaxation of the spins to the low-temperature equilibrium, the sample should be warmed on a time scale much less than the nuclear spin relaxation time. This can be done after a rapid pneumatic ejection of the solid sample from the cryostat,^68^ or via rapid warming of the sample without physical extraction.^147,148^

Accepting long polarization build-up times, the polarization of nuclear spins is still only modest even under high *B*_0_/*T* conditions. Assuming a 10 T external field, the polarization of an ensemble of protons is only ∼1% at 1 K (Figure 4). To reach near-unity polarization, the temperature should be on the order of millikelvin, which requires the use of significantly more complicated cryogenic equipment and can lead to even longer *T*_1n_ times.

To date, brute force has only been widely applied in the context of prepolarizing samples in high field for low/zero-field detection. Prohibitively long nuclear spin *T*_1n_ values at low temperature have precluded widespread application of this technique for generating highly polarized samples for high-field detection.

One solution to the long polarization build-up times at cryogenic temperatures is to induce relaxation by doping in relaxation agents, but importantly the relaxation mechanism should be reduced or “switched off” during the sample warming/transport step to avoid significant polarization losses at the higher temperature. A particularly elegant demonstration of this concept was shown in the production of hyperpolarized ^129^Xe.^144^ Its *T*_1n_ at ∼1 K is many tens of hours which means brute force polarization would not be feasible, but this was lowered to just tens of minutes by adsorbing a layer of ^3^He onto the sample surface. Translational motion of ^3^He across the surface modulates the ^3^He–^129^Xe dipolar couplings, which acts as a relaxation pathway. Importantly, this pathway could be “switched off” by displacing the spin-1/2 ^3^He atoms with spin-0 ^4^He prior to the warming step. However, the relaxation induced by the ^3^He was only effective for a monolayer of ^129^Xe, and a different approach would be needed for bulk samples.

One promising route for bulk samples is to dope in paramagnetic metal ions (e.g., holmium, cerium, dysprosium) complexed with diethylenetriaminepentaacetic acid (DTPA) and freeze the sample as a glass to ensure homogeneous dispersion. These metal complexes can induce nuclear spin relaxation at low temperatures because there is spectral density overlap between the electron transitions and the nuclear Larmor frequencies, a mechanism that becomes inactive at higher temperatures.^146,149^ Unfortunately, the relaxivity of these complexes decreases rapidly below 1 K, which limits the polarization that can be achieved. In another approach, copper and platinum nanoparticles (1050 nm size range) were shown to be effective relaxation agents at subkelvin temperatures.^150^ The relaxation mechanism is not fully understood, but it relies at least in part on energy transfer between the nuclear spins and the conduction electrons of the metal. With this approach, an impressive ^13^C polarization of up to 18% in the solid state was achieved by cooling the sample to 15 mK. It should be noted that the nanoparticle relaxivity cannot be “switched off” prior to sample warming and extraction, so a dissolution approach similar to that used in *d*DNP experiments (section 3.4) would be appropriate in order to dilute the nanoparticles at the time of warming. In these experiments, the samples were [1-^13^C]sodium acetate and sodium phosphate dissolved in water–glycerol solutions, with glycerol included to aid in forming a glass upon sample freezing. The eventual goal is to polarize a range of nuclei (often ^13^C with a view toward biological application), but ^1^H spins typically have shorter relaxation times due to the stronger coupling to the magnetic environment and are in much higher abundance if the solvent is protonated. Indeed, it was demonstrated that the proton spin bath (predominantly from water and glycerol) can be polarized via brute force, and this polarization transferred to other nuclei in the sample via low-field thermal mixing (LFTM).^146^ If the external field is reduced such that the dipolar couplings dominate for a short period of time, the Zeeman polarization can be transferred to coupled spins by spin diffusion. This technique was studied in detail on samples of [1-^13^C]pyruvic acid polarized at 2 T below 20 K, and the mixing times on the order of 100 ms in a field between Earth’s field and 10 mT were found optimal for ^1^H → ^13^C polarization transfer.^151^

**Figure 11:**
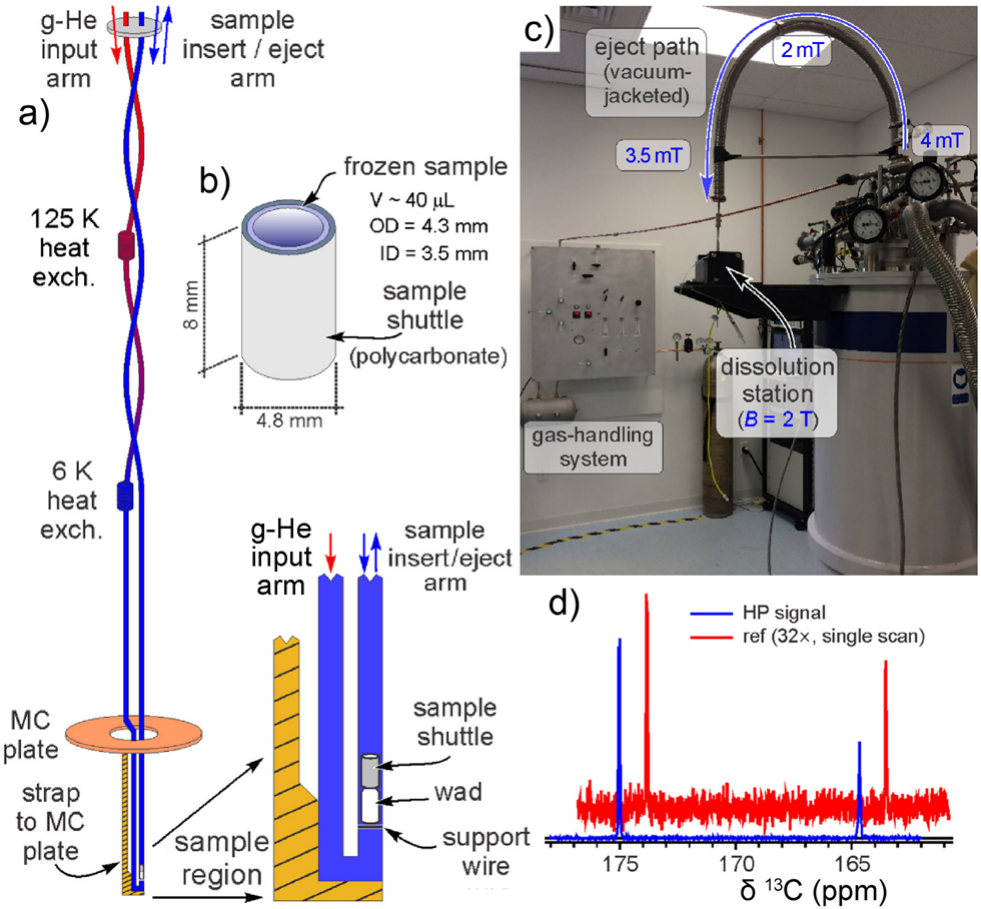
Brute force approach applied for hyperpolarizing [1-^13^C]pyruvic acid for solution-state NMR experiments. (a) The experimental apparatus. (b) The sample cup used to hold the pyruvic acid during sample transport. (c) A photograph showing the experimental apparatus, including the 2–4 mT low-field thermal mixing sample-transfer path for ^1^H → ^13^C polarization transfer. (d) Hyperpolarized and thermal equilibrium ^13^C NMR spectra of [1-^13^C]pyruvic acid showing signal enhancement from brute force polarization. Reproduced with permission from ref (68). Copyright 2015 American Chemical Society.

The previously described NMR experiments were all performed in situ on the cryogenically cooled solid-state samples. A promising extension is to pneumatically eject the solid hyperpolarized material from the polarizer and allow LFTM to occur in Earth’s magnetic field as the sample travels between the polarizing magnet and a high-field dissolution station.^68^ The experiments were performed on [1-^13^C]pyruvic acid polarized at ∼2 K in a 14 T field, which was dissolved in warm water in a 2 T field and measured at 1 or 9.4 T. The proton *T*_1n_ time in this sample was approximately 10 times shorter than the ^13^C *T*_1n_, which made it possible to leverage the faster polarization build-up on the protons, followed by transfer to ^13^C by LFTM. The experimental apparatus and results are illustrated in Figure 11. In an extension to this work, the authors showed that after polarizing in the 14 T magnet, the pyruvic acid could be ejected as a solid and stored at 60 K in a portable 1.3 T magnet.^152^ In this condition it was driven in ∼10 min to a separate location and then dissolved for solution-state NMR imaging experiments in phantoms.

In this work the ^13^C polarization measured in the solution state was approximately 0.1%, which is limited by the relatively high polarization temperature (∼2 K). Although this is not enough polarization for in vivo imaging applications of [1-^13^C]pyruvate, this is a promising demonstration of a general hyperpolarization method that can be used to improve NMR signals by orders of magnitude in a general way. One point to note is that the modest ^1^H *T*_1n_ times are likely a result of rotational motion of the methyl groups in pyruvate acting as a relaxation pathway, and other molecules not possessing similar degrees of motional freedom might exhibit longer polarization build-up times.

The greatest drawback to the brute force approach is the long times required to build-up sufficient polarization at cryogenic temperatures. However, the method does not in principle require field homogeneity of the polarizing magnet of better than a few percent, which means that it should be possible to polarize many samples at the same time in a magnet with a large bore. This would help to overcome the challenge of long sample *T*_1n_ times since many samples could be polarized together and extracted on demand.

In addition to using high-field magnets, there is another (less general) way to effectively produce high magnetic fields on nuclei.^153,154^ The idea is that the magnitude of magnetic hyperfine interactions in some paramagnetic compounds can reach into the gigahertz range, meaning the nuclei “see” a magnetic field from the electrons on the order of 100 T.

We now briefly mention a “brute-force” polarization experiment that serves as a paragon of ingenuity and, moreover, played a pivotal role in modern science. In 1956, parity nonconservation was discovered–this is a lack of “mirror symmetry” in the weak interaction.^155^ The experiment required a considerable degree of nuclear spin polarization of ^60^Co nuclei (*I* = 5), but common cryogenic techniques at the time did not permit cooling below about 1.2 K achievable by vacuum-pumping on liquid helium. Sample temperatures down to about 3 mK were achieved by using the technique of adiabatic demagnetization. A sample containing ^60^Co nuclei was deposited onto a film of a paramagnetic salt with a highly anisotropic g-factor. After the electrons were polarized in the paramagnetic substrate along the high-g direction, the magnetic field was reduced to depolarize the electrons and hence cool the sample (i.e., adiabatic demagnetization). Reintroducing the magnetic field in the same direction would lead to repolarization of the electrons, and hence reheating of the sample. However, by applying the magnetic field in the low-g direction, reheating is considerably reduced, and the nuclear spins can be highly polarized. This “trick”^156^ yielded the necessary experimental conditions: ^60^Co nuclei polarized along a magnetic field defining the axis of the experiment.

### 3.2 Overhauser Dynamic Nuclear Polarization

#### 3.2.1 The Technique

The dynamic nuclear polarization (DNP) techniques, including Overhauser-enhanced DNP (OE-DNP), rely on the fact that, owing to the larger gyromagnetic ratio γ, spins of unpaired electrons attain larger equilibrium polarizations in an external magnetic field compared to that of nuclear spins (Figure 4). Thus, upon polarization transfer from electrons to nuclei (section 2.2.1.4) the maximum possible sensitivity enhancement in a DNP NMR experiment (ε) corresponds to γ_e_/γ_n_ where γ_e_ and γ_n_ are the gyromagnetic ratios of the electron and the nucleus being polarized, respectively. OE-DNP was theoretically predicted for metals by Overhauser^79^ and shortly after that experimentally confirmed by Carver and Slichter,^80,157^ who reported that the saturation of conducting electrons in metallic lithium at their Larmor frequency led to a 100-fold enhancement of the ^7^Li NMR signal. Soon after that it was found that this mechanism is also effective in liquids: either for transferring polarization from unpaired electrons of paramagnetic species to nuclear spins of diamagnetic molecules,^158^ or between nuclear spins with different gyromagnetic ratios^159^ (nuclear Overhauser effect, NOE; section 2.2.1.3). In fact, Overhauser DNP described in this section is the only mechanism of dynamic nuclear polarization in liquids, in contrast to solids where several DNP mechanisms are known (sections 3.3 and 3.4).

**Figure 12:**
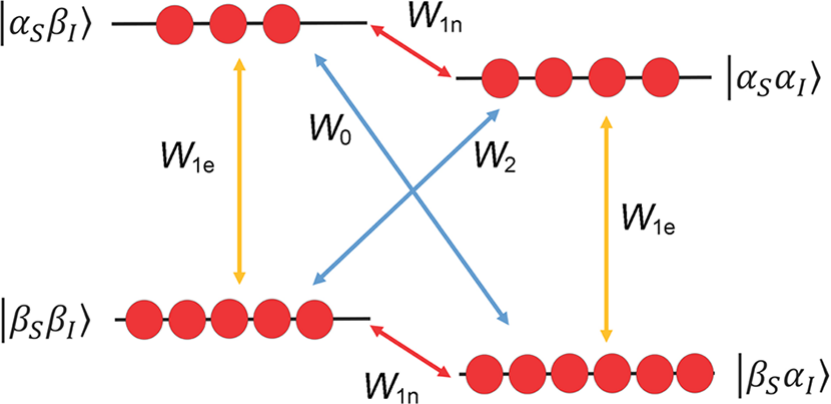
Level diagram for the coupling of an electron spin *S* = 1/2 with a nuclear spin *I* = 1/2 with Boltzmann equilibrium populations of the levels. *W*_1n_ and *W*_1e_ are the single-quantum nuclear spin relaxation rate and *W*_2_ and *W*_0_ the double- and zero-quantum electron–nuclear cross-relaxation rates, respectively. The difference between the two cross-relaxation rates *W*_0_ and *W*_2_ is the basis of OE-DNP.

**Figure 13:**
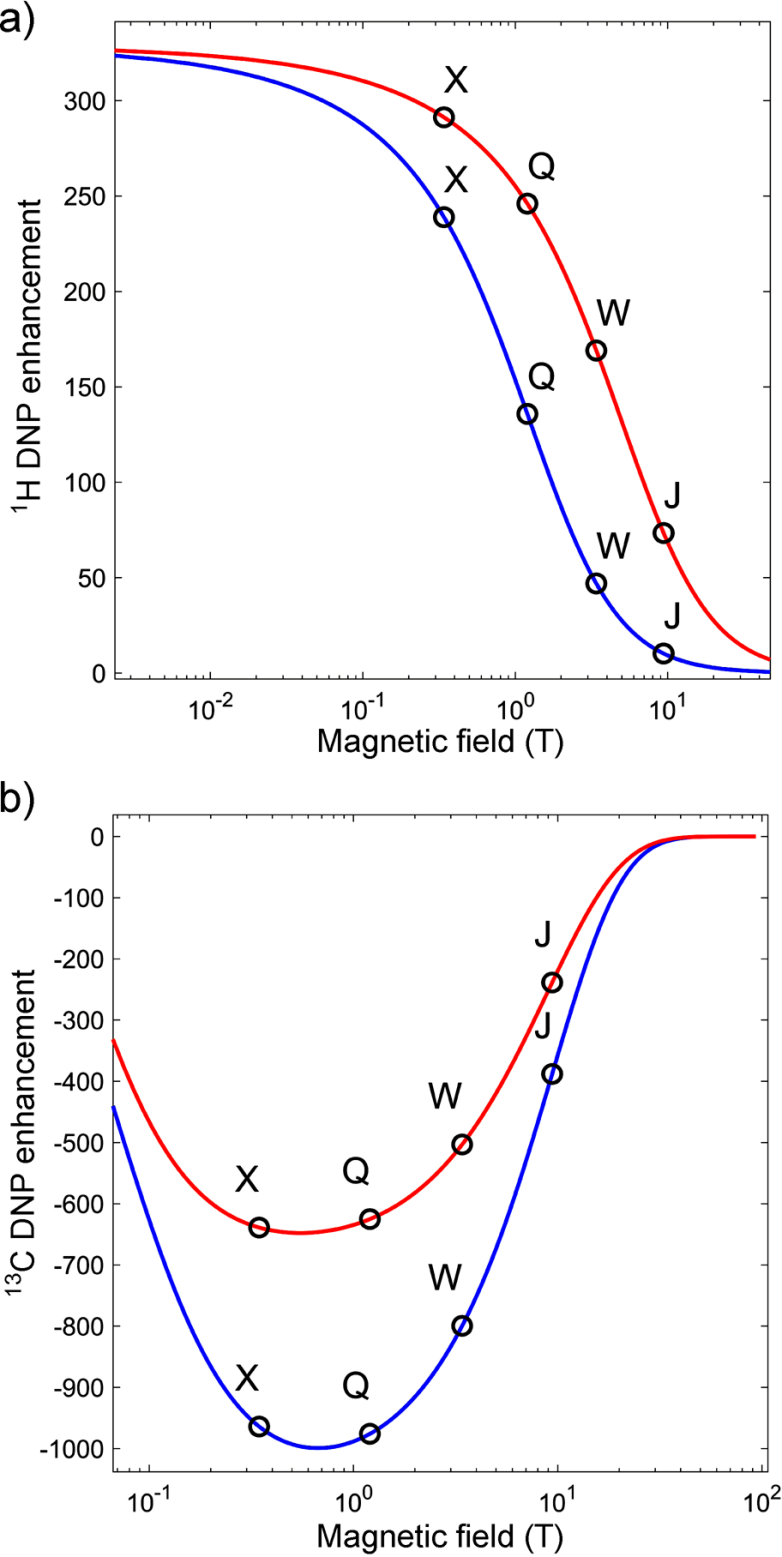
Field dependence of OE-DNP enhancement (calculated here as (*I* – *I*_0_)/*I*_0_ = ε – *1*, see eq 5). (a) Calculated ^1^H enhancement of water protons for a 40 mM aqueous TEMPOL (4-hydroxy-2,2,6,6-tetramethylpiperidin-1-oxyl) solution assuming a diffusion coefficient of *D* = 6 × 10^–9^ m^2^/s. Two different distances of closest approach were chosen (0.2 nm, red, and 0.4 nm, blue), reflecting the uncertainties caused by the nonspherical electron spin-density distribution of the nitroxide radical. Note that the enhancements arising from dipolar coupling are negative and are shown as absolute values in the diagram. (b) Calculated ^13^C enhancement for 10 mM TEMPONE (4-oxo-2,2,6,6-tetramethylpiperidine-*N*-oxyl) in ^13^C-chloroform including the dipolar contribution from translational motion and a scalar interaction with a spectral density *J*(ω_e_,τ_c_) = *F*[τ_c_exp(−ω_e_τ_c_)]^2^ with *F* = 1.7 × 10^24^ rad^2^ s^–2^ and a collision time τ_c_ = 0.5 ps.^165^ Calculations were performed for two different diffusion constants (*D* = 1.4 × 10^–10^ m^2^/s, red, and 2.8 × 10^–10^ m^2^/s, blue), reflecting different sample temperatures induced by MW heating, and a distance of closest approach of 0.4 nm. For both cases the leakage factor *f* and the saturation factor *s* were assumed to have a value of unity. Shown with circles are typically used microwave excitation frequencies (X-band: 0.35 T, Q-band: 1.2 T, W-band: 3.4 T, and J-band: 9.4 T).

OE-DNP relies on the saturation of allowed transitions of unpaired electron spins by microwaves. If the unpaired electron spin is coupled to a nuclear spin via hyperfine interaction, the processes that drive the electron spin system back to thermal equilibrium involve, inter alia, electron–nuclear cross-relaxation processes which flip both spins, thereby transferring polarization from electrons to nuclei. The efficiency of this process is largely governed by the coupling factor ξ between the electron and nuclear spins which accounts for the details of the mechanism responsible for the polarization transfer. From the Solomon equations^81^ it can be derived as
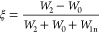
4with *W*_1n_ being the single-quantum nuclear spin relaxation rate and *W*_2_ and *W*_0_ the double- and zero-quantum electron–nuclear cross-relaxation rates, respectively (Figure 12). The cross-relaxation processes are induced by the modulation of the hyperfine coupling between the electron spin and the nuclear spin by dynamic processes. Depending on the specific model for such dynamic modulation, analytical expressions for the coupling factors have been derived. Usually, these dynamics are described by a specific correlation time τ_c_ and the related spectral density functions *J*(ω,τ_c_) for each individual process. The *J*(ω,τ_c_) values at the Larmor frequencies of the nuclear (ω = ω_n_) and (more importantly) the coupled electron–nuclear spin transitions (ω = ω_n_ ± ω_e_) are important for the cross-relaxation processes to occur and allow one to calculate the cross-relaxation rates *W*_*0*_ and *W*_*2*_. The coupling factor ξ can take values between −1 (scalar/isotropic hyperfine coupling, where only flip-flop cross-relaxation processes occur) and +1/2 (exclusively anisotropic dipolar hyperfine coupling).^158,160−164^ At high magnetic fields (and correspondingly high electron spin Larmor frequencies), where ω_e_τ_c_ > 1, all these spectral densities *J*(ω,τ_c_) approach zero, leading to a coupling factor of zero and therefore vanishing DNP enhancements (Figure 13).

In addition to the coupling factor, the efficiency of OE-DNP depends on other parameters as well, and the achievable polarization is given by the Overhauser formula:^160^
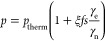
5where the gyromagnetic ratios for the unpaired electron and nuclear spins are given by γ_e_ and γ_n_, respectively, *f* is called the leakage factor, and *s* the saturation factor. For a free organic radical and a proton nuclear spin, the γ_e_/γ_n_ ratio is ca. −658 (γ_e_ is negative), leading to a maximum achievable DNP enhancement of 659 for ξ = −1 (scalar coupling) and −328 for ξ = +1/2 (dipolar coupling) at high enough microwave power (*s* = 1) and radical concentration (*f* = 1). For a ^13^C nuclear spin, the maximum achievable DNP enhancement amounts to 2618 for pure scalar coupling. The saturation factor *s* describes the level of saturation of the electron spin transition by microwave (MW) irradiation. It depends on the microwave field strength and the EPR spectral properties of the paramagnetic molecule. These are the electron spin relaxation times *T*_1e_ and *T*_2e_, the hyperfine coupling constants to the nuclei of the radical itself, and also the Heisenberg exchange rates which depend on the radical concentration. These parameters can be determined independently from EPR experiments.^166^ With large enough microwave excitation power, the saturation factor reaches unity. The leakage factor *f* describes the efficiency of the relaxation of the observed nuclear spins by the electron spins of the paramagnetic molecules. It depends on the concentration of paramagnetic molecules and their electron spin relaxation rates. The leakage factor can easily be determined by NMR *T*_1n_ measurements on samples with and without paramagnetic molecules present. For radical concentrations in the low millimolar range and higher, this factor is typically close to unity.

Proton spins of diamagnetic target molecules in solution containing paramagnetic radicals show mostly negative OE-DNP enhancements arising from dipolar hyperfine couplings between the electron spin of the radical and the nuclear spin of the target molecule, modulated by translational diffusive motion and rotational motion of short-lived solvate-radical complexes. Other nuclei, such as ^13^C, ^19^F, ^15^N, and ^31^P, can also have significant DNP enhancements arising from a fast modulation of the scalar hyperfine coupling by collision of target molecules with the radical and via formation of their short-lived complexes. Because of the opposite signs of the coupling factors arising from the scalar and dipolar hyperfine contributions, a significant reduction of the overall DNP enhancement can occur at lower magnetic fields, where both mechanisms are effective.^167−169^ This compensation effect can lead to a nonmonotonous field-dependence of OE-DNP enhancements for ^13^C nuclei^165^ as visualized in Figure 13b.

#### 3.2.2 Practical Aspects

The instrumentation required for OE-DNP depends on the magnetic field used for the microwave excitation and NMR detection.^170^ If the OE-DNP experiment is performed at low magnetic fields (0.3 T) to achieve optimal coupling factors, standard X-band EPR equipment can be used for the irradiation of the electron spin of the free radical. With NMR detection at the same field the challenge is to obtain enough spectral resolution, especially for proton spins. Recently it was shown that with improved magnetic field homogeneity and low paramagnetic concentrations line widths less than 4 Hz could be achieved at 0.3 T.^171^ Experimental setups also exist for ultralow magnetic fields for imaging applications,^172^ for detection of low-γ nuclei,^173^ monitoring of flow reactions^174^ or for magnetometry.^175^ Recently, also a miniaturized setup working at 0.5 T magnetic field was described for microfluidic chip applications.^176^

Experiments can also be performed with a subsequent detection at higher magnetic field to improve the spectral resolution. In this case, the reachable enhancement is lowered by the ratio of the polarizing and detection fields, and the challenge is to achieve a fast and efficient transfer of the hyperpolarized liquid from the low polarization field (0.3 T) to the NMR detection field to avoid polarization loss within the transfer time. This can be accomplished with flow systems,^177^ or shuttling of the sample^178^ or the entire probe.^179^

If, alternatively, the OE-DNP polarization buildup is accomplished directly (in situ) at high NMR detection fields, the experimental requirements are more stringent. First, high-frequency microwave sources in the >100 GHz range are required for the saturation of electron spin transitions. Microwaves in this range can be generated by up-converting the output of semiconductor sources, thus covering the range of up to 300 GHz (with power <1W), or by gyrotron sources which can deliver power in the range above 10 W. Second, in this case, care should be taken to avoid heating of the liquid sample by high-frequency microwave irradiation. Heating can be reduced considerably by placing the sample into a resonant MW structure. Such specific DNP (NMR/EPR) double-resonance structures limit the sample volume to a few microliters only, depending on the microwave frequency.

**Figure 14:**
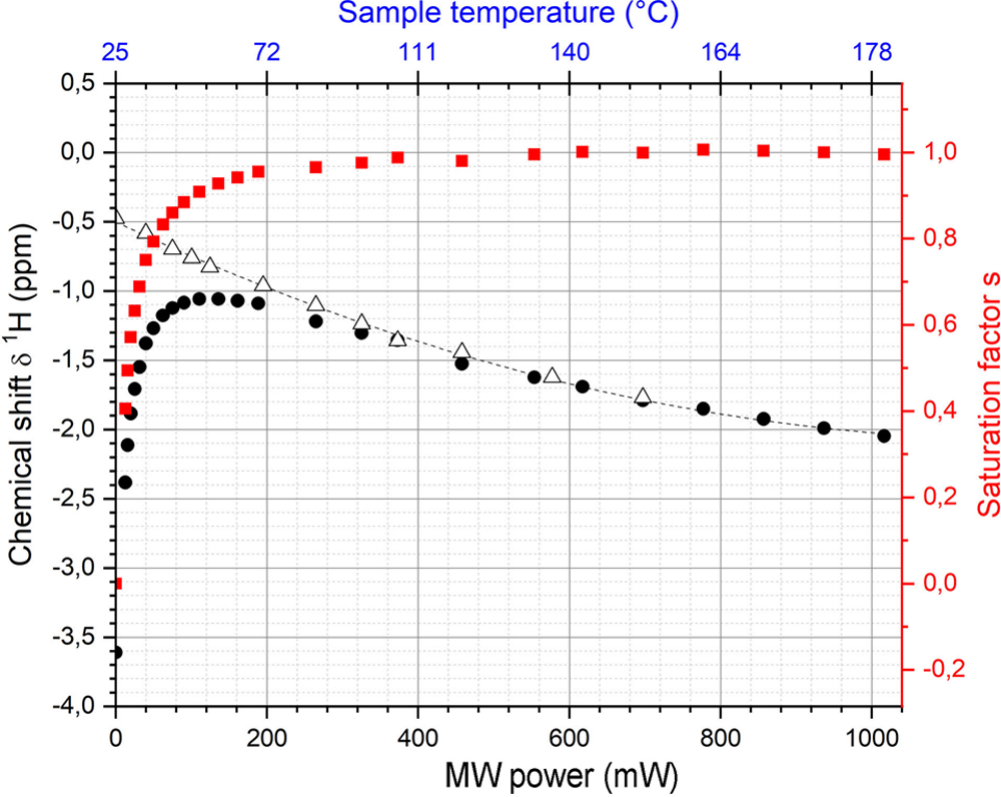
Experimental determination of saturation factor *s* at 9.4 T by observing the suppression of the water proton chemical shift of an aqueous solution of TEMPOL by MW irradiation (●). The diagram also demonstrates the chemical shift of water sample heated by the applied microwave power (△), which has to be subtracted to obtain the pure suppression of the paramagnetic shift (red ■) which reflects the saturation behavior at microwave powers larger than 200 mW. Reproduced from ref (180) with permission from the Royal Society of Chemistry.

Because the strong increase of the sample temperature is a function of applied microwave power *P*_MW_ at high microwave frequencies, it is difficult to extract the maximum achievable enhancement (for *s* = 1) from the intercept of the plot of *I*_0_/(*I* – *I*_0_) against 1/*P*_MW_, something that is possible at lower microwave frequencies. For the same reason it is hardly possible to determine the saturation factor from recording the EPR intensity as a function of the square root of MW power. Pulsed electron–electron double resonance (ELDOR) was proposed as a method to determine the effective saturation factor;^166^ however, such experiments performed at lower MW frequencies are also not easy to implement at high MW frequencies. One way to solve this problem arises from the effect of the saturation factor on the paramagnetic shift in an NMR spectrum of diamagnetic species in solution. At full saturation, the electrons become completely depolarized, and the paramagnetic shift disappears. The separation of the temperature effect into the chemical shift and the variation of the paramagnetic shift under MW irradiation is facilitated by an enhanced chemical shift resolution at high magnetic fields. Figure 14 shows how the suppression of the paramagnetic shift by saturation of the electron transitions can be observed on an aqueous solution of 4-hydroxy-2,2,6,6-tetramethylpiperidin-1-oxyl (TEMPOL) radical.^180^

For OE-DNP, nitroxide radicals are mostly used. Typically, radical concentrations in the range of 1–100 mM are used, which result in leakage factors of almost unity. NMR line broadening is only observed for high radical concentrations (>100 mM), partly because of the reduced spectral resolution obtained with the double-resonance structures. The reduced amount of sample inside the double-resonance structure limits the overall sensitivity, which can make reliable measurement of the thermal equilibrium (Boltzmann) signal demanding, for example for ^13^C signals at natural isotope abundance or low-γ nuclei. On the other hand, such MW-resonant structures increase the MW field strength at the sample, meaning that solid-state MW sources with moderate power might be sufficient to saturate the electron spin system.

All OE-DNP experiments performed directly at high magnetic fields have been performed with a continuous-wave (CW) irradiation with MW so far, mostly because a fast and efficient switching at high microwave frequencies is nontrivial to achieve. Potentially, MW irradiation with short inversion pulses could reduce sample heating and lead to efficient polarization transfer, especially in the case of inhomogeneously broadened lines, resulting from slow tumbling of a nitroxide in viscous environments. In such cases, pulsed excitation can achieve higher saturation levels compared to continuous-wave excitation.^181,182^

#### 3.2.3 Applications, Frontiers, and Challenges

OE-DNP in liquids has been studied extensively at low magnetic fields (<1.5 T) since the 1960s.^160,183,184^ DNP enhancements of ca. −180 for water proton spins in the presence of deuterated ^15^N-labeled 4-oxo-2,2,6,6-tetramethylpiperidine-*N*-oxyl radical (^15^N-TEMPONE) have been observed at X-band frequencies^166^ and could be modeled quite well by using an independent experimental determination of the saturation factor *s* and the leakage factor *f*, and the coupling factor ξ estimated by taking account of translational and rotational motion of the spin-bearing molecules in solution using the classical diffusion equation.^163^ Quantitative comparisons with the experimentally obtained DNP enhancements have been used to derive local information on the translational dynamics of the paramagnetic molecules and of the target molecules bearing the nuclear spin as well as information on their encounter complexes in liquid solutions.^184−187^ Recent applications are the investigation of polymers, ionic liquids and viscous liquids in combination with field-cycling NMR,^188^ the investigation of local water dynamics in soft materials^187^ and at the surface of biomolecules^185^ or potential applications in electrochemical cells.^189^

OE-DNP was also used to increase the sensitivity for MRI applications. OE-DNP MRI detection of free radicals, called PEDRI (proton–electron double resonance imaging) was pioneered by the Lurie group.^190^ It relies on the fact that signal enhancement in an image obtained by MRI under OE-DNP conditions reflects the local concentration of paramagnetic species. To achieve large enough sample penetration depth of the oscillating electromagnetic fields that saturate the electron spin transitions, polarization transfer is typically performed at low magnetic fields, i.e., at low resonance frequencies. Field cycling after the polarization transfer step has been developed to obtain images at higher detection fields.^191^ OE-DNP has also been used for flow imaging of hyperpolarized aqueous solutions. Again, similar to spectroscopic applications, the hyperpolarization step could be performed at lower magnetic fields^174,192^ or directly at the field of the magnet used for imaging.^193,194^ Continuous streams of radical-free liquids including water can be produced if radicals are immobilized on a suitable porous solid material^177^ or a gel.^174,192^

More recently, OE-DNP was also explored at high magnetic fields (>3 T) to achieve increased chemical shift resolution for spectrally resolved NMR applications. This is in conflict with the decreasing spectral densities (and therefore the coupling factor and the DNP enhancements) predicted for higher magnetic field values (Figure 13). DNP studies at various magnetic field strengths were performed to experimentally access and understand the dynamic processes in the terahertz regime relevant for OE-DNP processes at high magnetic fields. The first approach is to perform the OE-DNP experiment by microwave irradiation at low magnetic field and flow^177^ or shuttle^178^ the liquid subsequently to high magnetic field for NMR detection.^179^ The second approach is to perform OE-DNP experiments directly at the high magnetic field used for NMR detection (3.4 or 9.2 T).^195−198^ An impressive Overhauser ^1^H DNP enhancement of ca. −80 for water and ^13^C DNP enhancements of ca. +600 for ^13^C-tetrabromomethane in the presence of dissolved nitroxide radicals have been demonstrated at a magnetic field strength of 9.2 T.^165,199^ Because most solvents strongly absorb microwaves in this frequency range, microwave resonance structures have been used to avoid excess heating of the sample.^200,201^

**Figure 15:**
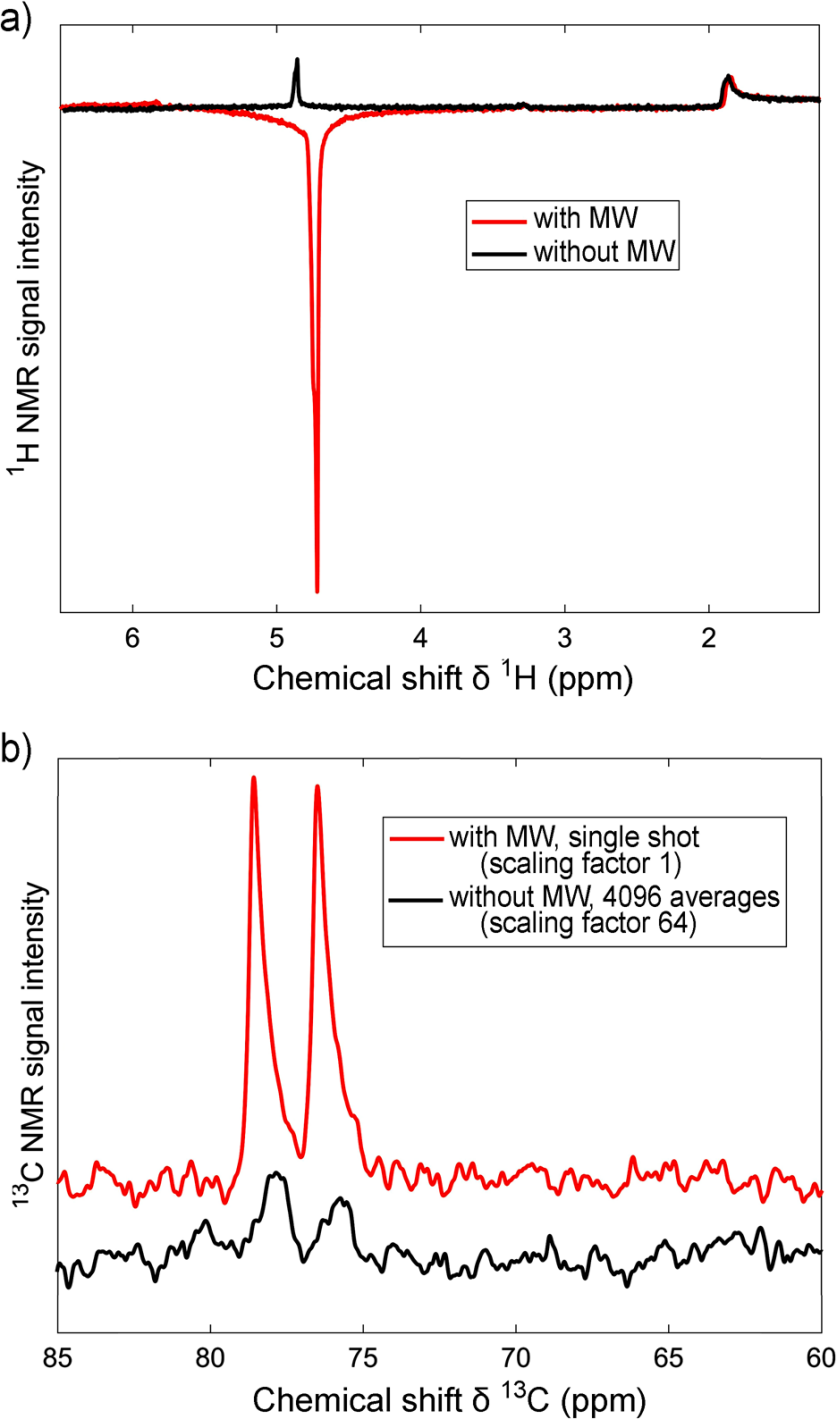
OE-DNP experiments in liquid solutions at 9.2 T. (a) ^1^H DNP enhancement of an aqueous solution of 24 mM TEMPOL. The sample volume is 90 nL, placed on the flat mirror of a Fabry–Pérot/stripline double-resonance structure. The temperature rise of the sample at 3 W applied microwave power is 30 °C. Shown is the signal without microwaves (black) and the negatively enhanced signal with microwaves (red). The small peak at 2 ppm arises from some liquid outside of the microwave excitation zone. (b) ^13^C DNP enhancement for ^13^C-chloroform with 100 mM of dissolved TEMPONE. The sample volume was 35 nL inside a 100 μm diameter capillary placed in the center of a cylindrical/helical double-resonance structure. The increase in sample temperature in this case was about 50 °C above room temperature with 1 W of microwave power. (a) Reprinted with permission from ref (199). Copyright 2015 Elsevier Inc.

In Figure 15, examples of proton and carbon OE-DNP enhancements at a magnetic field of 9.4 T are illustrated. Figure 15a shows the OE-DNP enhancement for water protons in an aqueous TEMPOL solution. A Fabry–Pérot/stripline 260 GHz/400 MHz double-resonance structure was used to saturate the electron spin transitions and to detect the proton NMR free induction decay (FID) signal.^165^ This structure can accommodate a 90 nl sample with only a moderate sample heating (by 30 °C) by the high-frequency excitation with 3 W of microwave power. The sample temperature can be further raised by applying more microwave power, increasing the diffusion constant *D* and therefore also increasing the coupling factor and the DNP enhancements.^199^ More recently this probe has been further improved to a triple-resonance probe including a ^13^C channel and increased temperature stability of the sample under MW irradiation conditions.^202^ The ^13^C DNP enhancement of ^13^C-labeled chloroform with 100 mM of deuterated ^15^N-TEMPONE is illustrated in Figure 15b. In this example, the DNP experiments at 9.2 T have been performed using a cylindrical/helical double-resonance structure tuned to the electron spin/^13^C resonance frequencies.^165^ For this type of cavity, the sample volume was restricted to 35 nL. Also, because a higher mode along the capillary length was used for the microwave cavity, more of the electric field component of the microwave was absorbed by the sample, leading to the liquid sample temperature rising by 50 °C. Recently, it was demonstrated that large ^13^C scalar enhancements can also be obtained in aqueous solutions at room temperature, which is interesting for potential biological applications.^203^

**Figure 16:**
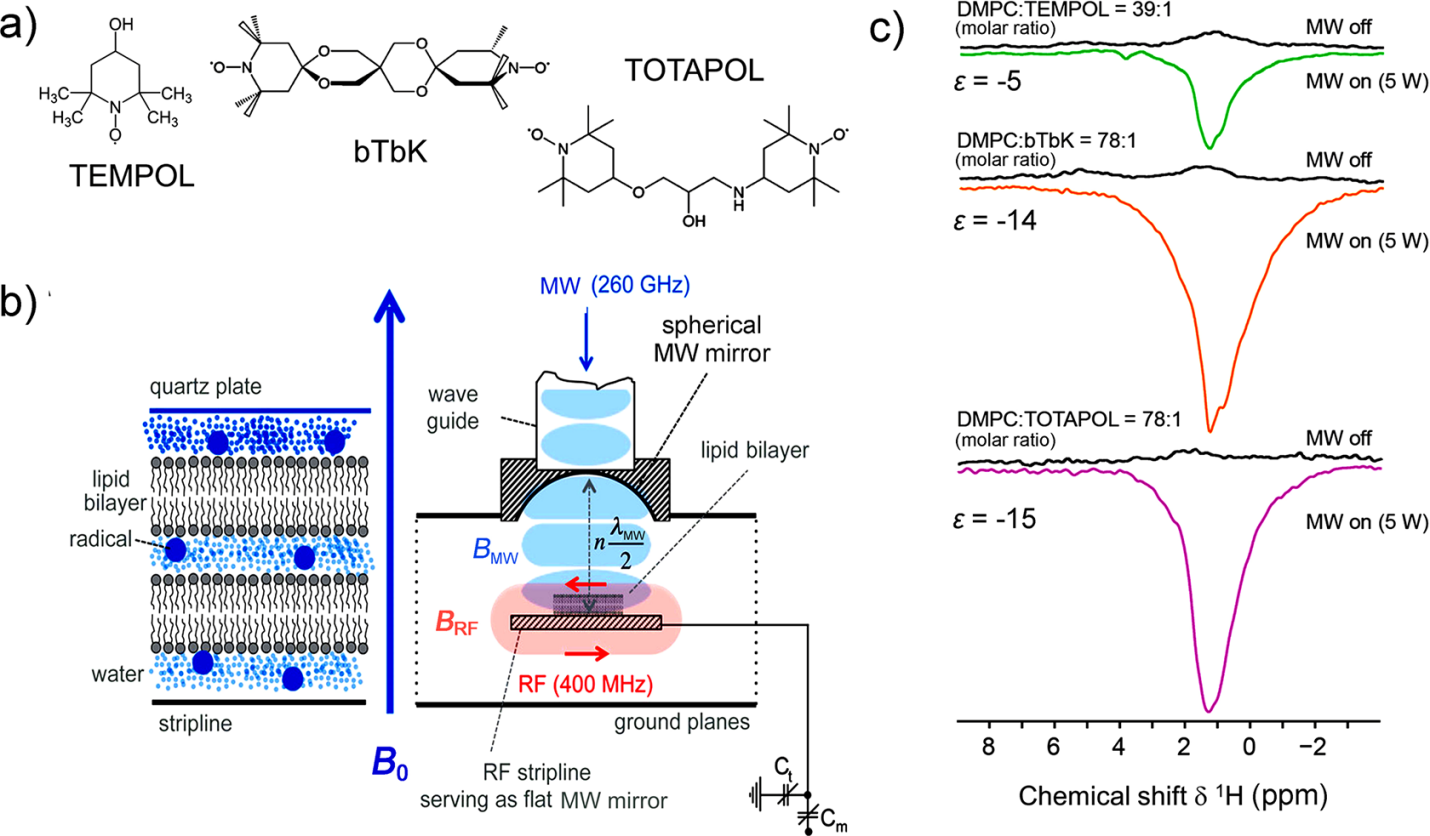
OE-DNP enhancement in the liquid phase of lipid bilayers at 9.4 T. (a) Different nitroxide mono- and biradicals mixed with the lipid. (b) Sample preparation and incorporation of the sample in the Fabry-Pérot double-resonance structure. The lipid bilayers are placed and partially aligned on the flat mirror of the Fabry–Pérot DNP double-resonance structure (400 MHz/260 GHz). *B*_MW_ and *B*_RF_ are the amplitudes of the oscillating MW and RF magnetic fields, respectively; λ_MW_ is the wavelength of microwave radiation; C_t_ and C_m_ are tuning and matching capacitors, respectively. (c) ^1^H DNP enhancements at 9.4 T magnetic field and room temperature of the acyl chain protons of dimyristoylphosphatidylcholine (DMPC) lipid bilayers doped with the different nitroxide mono- and biradicals as DNP agents. The values of ε were recalculated based on the integrated intensities of the ^1^H NMR signal according to eq 2. Adapted with permission from ref (210). Copyright 2014 American Chemical Society.

Recently, it was shown that even without microwave resonant cavities, decent ^13^C (ε ≈ + 70) and ^1^H (ε ≈ −10) OE-DNP enhancements could be reached at high magnetic fields.^204,205^ This was achieved by supplying high microwave power from a gyrotron directly to the sample and trying to avoid heating by placing the sample on a substrate with high thermal conductivity^204^ or by solvents characterized by low microwave absorption.^205^ This facilitates obtaining high-resolution NMR spectra, but strong local heating of the sample by microwave irradiation might also lead to degradation of some radicals commonly used for DNP. NMR relaxation dispersion measurements are useful to obtain information on the relaxation rates and spectral density profiles necessary to predict DNP coupling factors.^164,179,206^ At high magnetic fields, small contributions of fast fluctuations, which are difficult to detect by NMR dispersion curves, might contribute significantly to OE-DNP enhancements.^164,207^ Molecular dynamics simulations of the radical in its liquid environment have proven to be very useful to predict the coupling factors quantitatively in such cases.^165,197−201,204−209^ Fast local dynamics were also postulated to be the reason for the observed acyl chain proton DNP enhancements of up to −15 at a magnetic field of 9.4 T in ordered lipid bilayers doped with nitroxide radicals (Figure 16).^210^ (Note that, in some papers, signal enhancement is defined as (*I* – *I*_0_)/*I*_0_, which is different from that given by eq 2 and used throughout this review.) Surprisingly, OE-DNP enhancements were also observed at high magnetic fields in solid ortho-terphenyl doped with BDPA (1,3-bis(diphenylene)-2-phenylallyl) radicals at low temperatures.^211^ Again, molecular dynamics simulations and quantum chemical calculations revealed fast local dynamics of BDPA, rationalizing the size and field dependence of the OE-DNP enhancements observed at high magnetic fields.^212,213^

It has been demonstrated by several groups that sufficient OE-DNP enhancements can be indeed achieved on liquid samples even at high magnetic fields. The main restriction to real applications is due to the strong sample heating by microwave excitation and/or the strongly restricted sizes of the sample that can be put into a MW resonance structure. This reduces the typical NMR sample size by a larger factor than the achieved OE-DNP enhancement. Therefore, the method can so far not be used to enhance NMR signals of large volume samples at high magnetic fields. Thus, most studies at high magnetic fields have been centered on the investigation of the fundamental mechanism responsible for the OE-DNP efficiency and the optimization of the experimental conditions. Because OE-DNP at high magnetic fields relies on a short-distance encounter complex between the DNP agent and the target molecule, it could also be interesting for studies of specific interactions and complexes in biomolecular systems. Applications of OE-DNP at high magnetic fields might be of interest for samples which are naturally limited in size, as the output from a high-performance liquid chromatography (HPLC) analysis or a microstructured chemical reactor, or which are restricted to 2D-surfaces as, for example, ordered lipid bilayers or catalyst surfaces. In such cases, the small amount of available sample could be enhanced by OE-DNP and observed with high enough chemical shift resolution. Further development of probe structures with efficient MW excitation of samples much larger that the fundamental MW wavelength and sensitive NMR detection with high spectral resolution might be possible to achieve and would open up many avenues to new and interesting applications.

**Figure 17:**
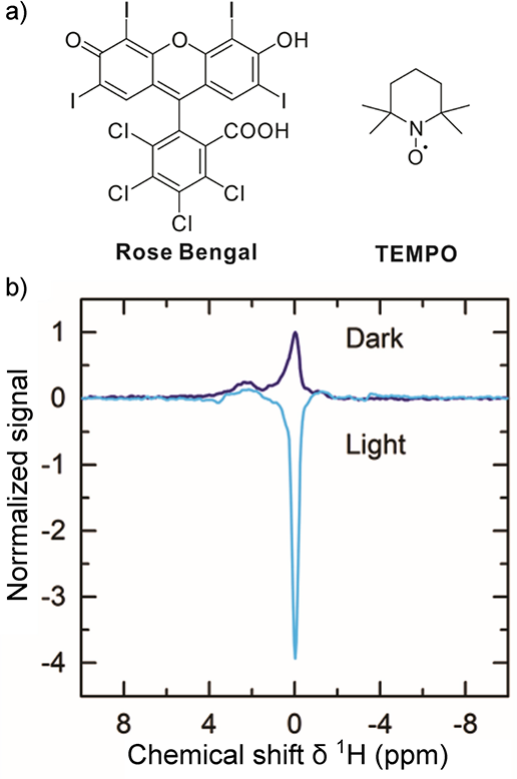
Example of application of RTPM-CIDEP-mediated OE-DNP for NMR signal enhancement. (a) Structure of the chromophore and free radical. (b) 1D ^1^H pulse-acquire NMR spectra of water solvent under dark (laser off) and light (laser on) conditions. Reproduced from ref (214) with permission from the Royal Society of Chemistry.

Conventionally, OE-DNP utilizes thermal equilibrium polarization of unpaired electron spins to enhance polarization of nuclear spins, as discussed above. At the same time, an attractive yet challenging possibility mentioned in section 2.2.2 is to use hyperpolarized electron spins in OE-DNP experiments, thereby increasing the maximum achievable nuclear spin polarization enhancements beyond the γ_e_/γ_n_ ratio. One example of the implementation of such approach^214^ is the use of chemically induced dynamic electron polarization (CIDEP; section 3.5) generated via the radical-triplet pair mechanism (RTPM); this possibility is illustrated in Figure 17. Several other examples of various DNP effects in short-lived radicals in solution can be found in section 3.7.

### 3.3 Solid-State Dynamic Nuclear Polarization with Magic Angle Sample Spinning

#### 3.3.1 The Technique,

Similar to DNP based on the Overhauser effect (OE-DNP, section 3.2), DNP enhancement in solid-state NMR spectroscopy relies on polarization transfer from unpaired electrons to the coupled neighboring nuclear spins (section 2.2.1.4) upon MW irradiation near the electron Larmor frequency, providing the maximum sensitivity enhancement ε = γ_e_/γ_n_ of, e.g., |ε| ≈ 658 for protons. In fact, OE-DNP, which is currently mainly exploited for DNP in liquids, was originally demonstrated in metallic lithium.^80^ This pioneering experiment stimulated research into the spin physics of DNP, and a number of other solid-state DNP mechanisms that are discussed below were discovered in the late 1950s and early 1960s.^215−219^ The potential of DNP to increase the NMR sensitivity for rare or low-γ nuclear spins in the solid state was soon realized,^220^ and with the introduction of suitable probe hardware,^221^ the DNP technique became compatible with modern solid-state NMR approaches, i.e., with cross-polarization (CP) and magic angle spinning (MAS). This in turn led to the first high-resolution DNP-enhanced solid-state NMR studies in the 1980s, reported on materials such as coal, doped polymers or diamond thin films.^222,223^ These experiments were carried out at room temperature and low magnetic fields, typically 1.4 T, in a handful of laboratories worldwide. Thus, DNP-enhanced solid-state NMR under MAS remained little used before experiencing a renaissance in the 90s, thanks to the work done by Griffin and co-workers who, with the introduction of gyrotron sources capable of delivering high-power high-frequency continuous microwaves, made it compatible with high magnetic fields (>5 T)^83^ and demonstrated that high sensitivity gains could be accomplished at cryogenic temperatures (<100 K) using nitroxide dopants as the source of electron polarization.^224^ The first application on a biomolecule was reported in 1997 with the DNP-enhanced ^15^N NMR spectrum of the T4 lysozyme protein in glycerol–water solutions frozen at 40 K, doped with the nitroxide free radical 4-amino-TEMPO.^225^ The data were acquired at a magnetic field of 5 T and at a spinning frequency of 3.2 kHz, and enhancement factors of up to 100 were reported. Over the years, the efficiency of DNP MAS NMR has been improved steadily, notably with the introduction of ever-more efficient polarizing agents and optimized sample formulations (detailed below). Today, DNP signal enhancements of ∼100 to 300 can be typically achieved on commercial instruments operating at ∼100 K. Enhancement factors of 200 were recently reported at the highest magnetic field available today for DNP MAS NMR, 21.1 T, at a spinning rate of 65 kHz using a 0.7 mm cryogenic MAS probe.^226^

**Figure 18:**
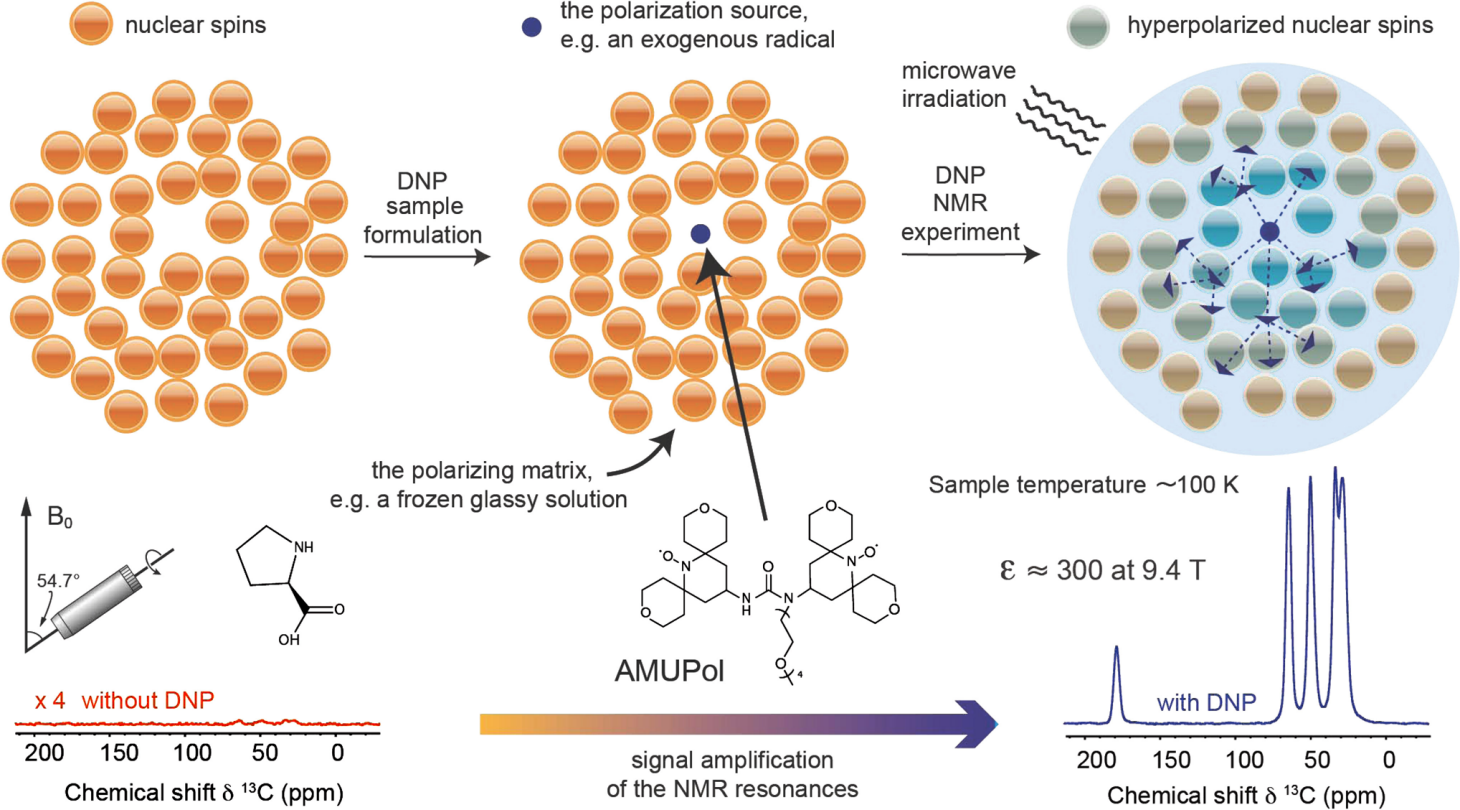
Principle of DNP-enhanced solid-state MAS NMR spectroscopy. The amplification power of DNP is illustrated for ^13^C-labeled proline in a bulk water/glycerol (glycerol-*d*_8_/D_2_O/H_2_O; 60:30:10) frozen solution containing 10 mM AMUPol stable biradical (chemical structure is shown in the figure) as the source of unpaired electrons. The ^13^C cross-polarization NMR spectra were recorded at 9.4 T (400 MHz proton frequency, 263 GHz microwave frequency) in a 1.3 mm rotor spinning at 40 kHz. The signal enhancement factor ε corresponds to the ratio of resonance intensity with and without microwave irradiation of the sample.

The general principle of DNP-enhanced solid-state NMR under MAS is illustrated schematically in Figure 18. In the case of a radical-free substrate, the polarizing agent (the source of unpaired electrons) is typically introduced in the form of an exogenous mono- or biradical dissolved in a glass-forming solution. Depending on the system of interest, other, more appropriate ways of formulating the sample were proposed, and these are discussed below. Continuous irradiation of the cryogenically cooled sample with microwaves at or near the EPR frequency results in transfer of polarization from the highly polarized electron spins of the paramagnetic dopant to the protons of the substrate through a number of mechanisms described in the next few paragraphs. Proton–proton spin diffusion, mediated by the protons of the frozen solvent matrix, then relays the hyperpolarization among the ^1^H nuclei to the molecular solid or material under study, where it is finally transferred to low-γ nuclear spins such as ^13^C, ^29^Si, ^15^N, ^31^P, ^27^Al, ^17^O, etc., either by cross-polarization or by any other ^1^H-X dipolar-based polarization transfer scheme. This methodology is referred to as indirect DNP. We note here that indirect DNP generated by ^1^H-X cross-relaxation transfers mediated by methyl reorientation has also been described.^227^ Alternatively, the large electron polarization of the polarizing agent can be transferred directly to the low-γ nuclei of interest and observed in a single-pulse experiment, a process called direct DNP. As will be discussed later on, homonuclear spin diffusion among rare, low-γ nuclear spins can also be exploited to efficiently distribute the directly or indirectly enhanced nuclear polarization.

Several schemes have been described and implemented for DNP MAS NMR that transfer the polarization from the unpaired electrons of the dopant to adjacent nuclei. The underlying mechanisms have been reviewed quite extensively.^228,229^ Here, we briefly describe the three main mechanisms encountered in DNP-enhanced solid-state NMR: the solid effect, the cross effect and the Overhauser effect. Their relative efficiency is dictated by several factors, such as molecular structure of the polarizing agent, its concentration, its electronic properties (from relaxation rates of the electrons to the size of magnetic interactions), as well as external magnetic field strength, sample temperature, and spinning frequency.

**Figure 19:**
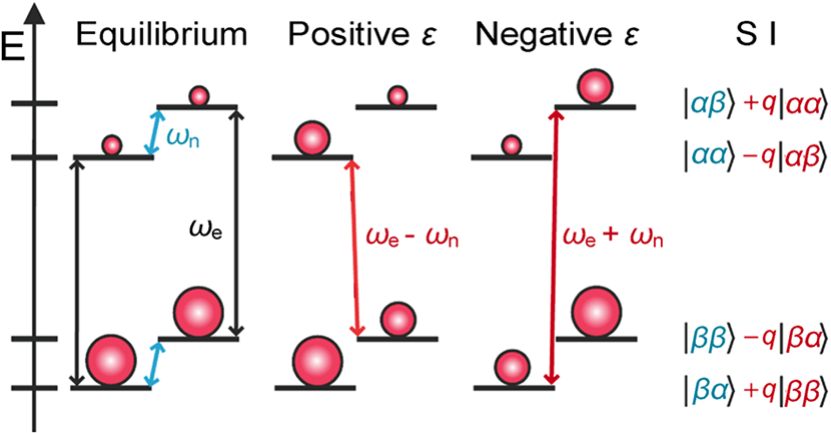
Energy level diagram illustrating DNP via the solid effect (SE). At thermal equilibrium (left), populations are governed by the Boltzmann distribution. Mixing of states in the electron spin subspaces (right) leads to partially allowed double-quantum (DQ) and zero-quantum (ZQ) transitions, and positive and negative enhancements, ε, respectively. The mixing of states is proportional to a constant *q*, which is inversely proportional to *B*_0_. Therefore, the enhancement in the solid effect DNP scales as 1/*B*_0_^2^. Adapted with permission from ref (229). Copyright 2013 American Chemical Society.

**Figure 20:**
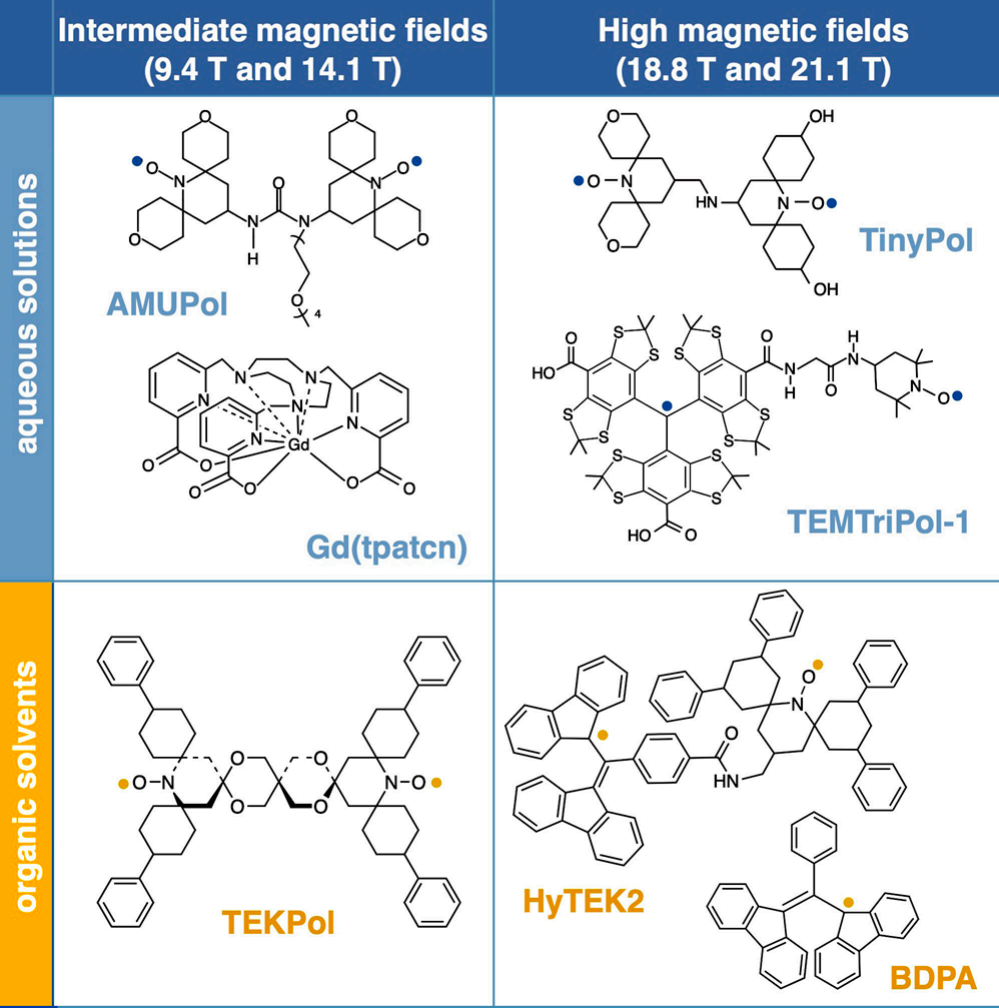
Molecular structure of some of the polarizing agents suitable for DNP MAS NMR at intermediate and high magnetic fields. Blue and orange dots denote the unpaired electrons.

**Figure 21:**
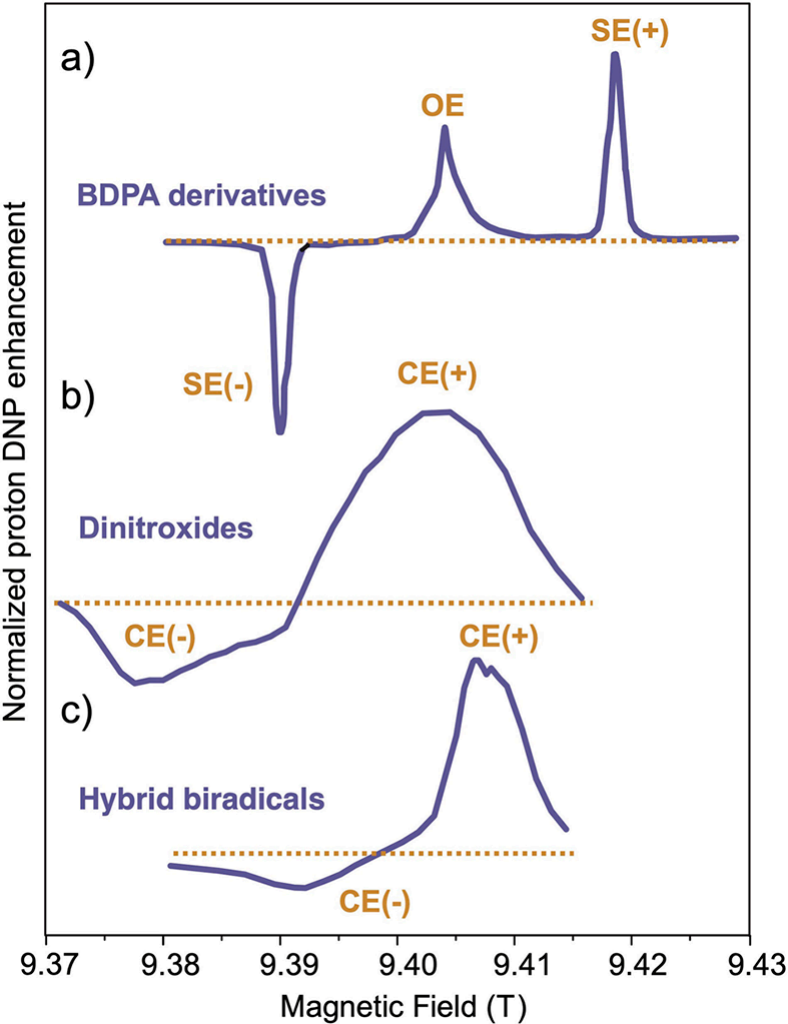
Characteristic proton DNP enhancement profiles as a function of the magnetic field observed for (a) BDPA derivatives, (b) dinitroxides, (c) hybrid biradicals. (a), (b), and (c) correspond to the experimental profiles for 32 mM BDPA in ortho-terphenyl (OTP) (95% OTP-*d*_14_, 5% OTP), 15 mM TEKPol biradical in CHCl_3_/1,1,1,2-tetrabromoethane (TBE)/CD_3_OD (65/30/5 vol %), and 16 mM HyTEK2 biradical in 1,1,2,2-tetrachloroethane (TCE), respectively. All the data were recorded with a constant microwave frequency of 263 GHz at a temperature of ∼100 K. The enhancements were measured for the NMR signal of the glassy matrix. In (b) and (c), intramolecular CE governs the DNP process and two relatively broad positive and negative lobes are observed. In (a), the SE yields two sharp positive and negative maxima, while the OE gives a positive enhancement in the middle of the field profile. (a) Adapted with permission from ref (231). Copyright 2015 American Chemical Society. (b) Adapted from ref (232) with permission from the Royal Society of Chemistry. (c) Adapted with permission from ref (233). Copyright 2018 American Chemical Society.

The solid effect (SE), discovered in the late 1950s,^215−217^ is a two-spin process in a dipolar-coupled spin system comprising one electron and one nuclear spin (Figure 19). It relies on nominally forbidden two-spin transitions upon microwave irradiation at ω_MW_ = ω_e_ + ω_n_ or ω_MW_ = ω_e_ – ω_n_, where ω_MW_, ω_e_, and ω_n_ are the microwave frequency, the electron, and the nuclear Larmor frequencies, respectively. Electron–nuclear spin flips are excited, and the nominally forbidden zero-quantum (ZQ) and double-quantum (DQ) transitions yield negative and positive enhancements, respectively. The probabilities for these ZQ and DQ transitions scale with 1/*B*_0_^2^ and therefore the efficiency of SE DNP diminishes at high fields.^230^ The SE is well-resolved for a radical with a narrow EPR spectrum, i.e., if the EPR line width Δω_e_ is smaller than the Larmor frequency ω_n_ of the nuclear spin to be polarized (Δω_e_ < ω_n_), such as trityl or BDPA (1,3-bisdiphenylene-2-phenylallyl; Figure 20), as well as for paramagnetic metal ions and metal complexes. Preferable conditions for the SE mechanism are high concentrations of slowly relaxing nuclear spins together with low quantities of rapidly relaxing electron spins. In this way, electron spins can interact with a number of dipolar-coupled nuclear partners in a short time period and participate multiple times in the electron–nuclear spin transitions. Figure 21a shows the experimental field profile for ^1^H DNP enhancement obtained with BDPA radical,^231^ where the two extrema that correspond to the two SE conditions are separated by twice the nuclear Larmor frequency.

**Figure 22:**
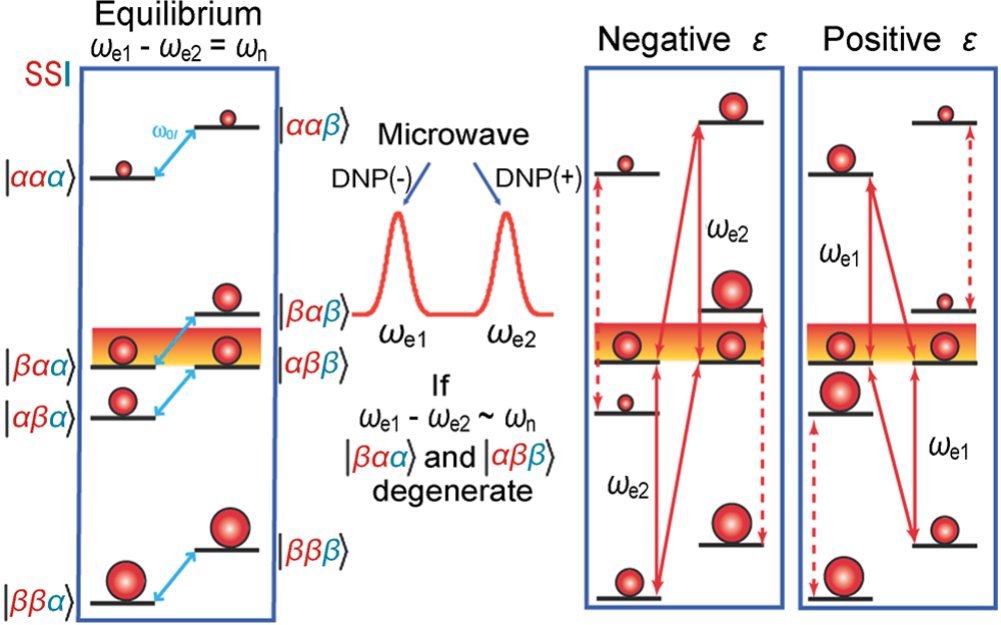
Energy diagram illustrating DNP via the cross effect (CE). At equilibrium (left), under the matching condition, there is degeneracy and 1:1 population of the two shaded levels. The EPR spectrum of an ideal biradical for CE (middle) has two narrow lines separated by the nuclear Larmor frequency. Saturation of transitions near the first (second) EPR line gives rise to a positive (negative) DNP enhancement (right). In the kets, the electron spin states are indicated in red and the nuclear spin state in blue. Reprinted with permission from ref (229). Copyright 2013 American Chemical Society.

The cross effect (CE) process, described in the 1960s,^218,219^ is today the most popular mechanism in DNP MAS NMR (Figure 22). It involves three dipolar-coupled spins: two electrons and a nucleus. When the difference of the EPR frequencies of the two electrons ω_e1_ and ω_e2_ matches the Larmor frequency of the nucleus, some of the eight energy levels of the three-spin system happen to be degenerate. Upon irradiation at one of the two electron Larmor frequencies, electron–electron flip-flops are induced that transfer the difference of electron polarization to the nucleus. The saturation of the electron with the higher EPR frequency leads to a negative enhancement while the saturation of the other electron leads to a positive enhancement. The condition ω_n_ = |ω_e1_ – ω_e2_| is the so-called CE matching condition. The rotation of the sample under MAS modulates the resonance frequencies of the electron and nuclear spins and complicates this picture. The cross effect under MAS has been recently described as a series of spin transitions of different degrees of adiabaticity at avoided crossings (a.k.a. anticrossings) of energy levels (LAC): (1) electron-microwave crossing, when the microwave frequency crosses the EPR frequency of one electron (i.e., when ω_MW_ = ω_e1_ or ω_MW_ = ω_e2_); (2) three-spin crossing, when the difference of the EPR frequencies crosses the NMR frequency (i.e., when ω_n_ = |ω_e1_ – ω_e2_|); and (3) electron–electron crossing when ω_e1_ = ω_e2_.^234−236^ This mechanism is particularly efficient for paramagnetic species having a broad inhomogeneous EPR line (with the width larger than ω_n_), such as bis-TEMPO biradicals, for which the CE matching condition between two nitroxide moieties with different orientations is permitted by the anisotropy of their *g*-tensors. Figure 21b shows the field profile of DNP enhancement obtained with TEKPol^237^ measured at 9.4 T,^232^ which is characteristic of dinitroxide biradicals. Experiments are typically recorded at the magnetic field yielding the maximum positive enhancement factor. As the EPR line width of TEMPO-based biradicals increases linearly with the external field, the efficiency of the CE mechanism is expected to scale at least with the inverse of the field. Recent theoretical work on CE DNP enhancements under MAS predicts a dependence between 1/*B*_0_ and 1/*B*_0_^3^ for dinitroxides.^235,236,238^ Such a downward dependence is, however, not expected for CE DNP when hetero- or hybrid biradicals composed of a nitroxide moiety linked to a radical with a narrow EPR line are used. Figure 21c shows the asymmetric field profile of DNP enhancement obtained with HyTEK2,^233^ measured at 9.4 T, which is characteristic of hybrid biradicals.

Under fast sample rotation, the MAS-induced modulation of the two electron frequencies leads to a sizable probability of nonadiabatic electron–electron crossings that attenuate the spin polarization differences between the electrons. This subsequently reduces nuclear spin polarization achieved via the CE mechanism, a phenomenon called nuclear depolarization, which also occurs in the absence of microwave irradiation.^236,239^

The Overhauser effect (OE) (Figure 12) is a third mechanism that has recently attracted a renewed interest in DNP MAS NMR after the discovery in 2015 that significant OE DNP enhancements could be observed in insulating solids doped with BDPA and its derivatives.^240^ Importantly, the experimental data demonstrated that the efficiency of the OE scaled favorably with increasing magnetic fields. The OE is a two-spin process. However, in contrast to the SE, the microwave irradiation is applied at the single-quantum EPR transitions of the electron and the enhancement is generated by zero-quantum and double-quantum relaxation (section 3.2, Figure 12). In insulating solids, the OE is based on stochastic modulation of scalar hyperfine couplings between electron and nuclear spins.^212^ The OE is observed on the field profile of BDPA (Figure 21a) midway between the frequencies corresponding to the two SE conditions.

Finally, we note that while all these mechanisms rely on the presence of hyperfine coupling between unpaired electrons and neighboring nuclei (usually protons), there is currently no detailed understanding of where these nuclei are located with respect to the polarizing agent, and whether there is a distance of a few tenths of a nanometer within which no DNP transfer would occur: the so-called spin-diffusion barrier in which spin diffusion from the protons nearest to the electron to the bulk is slowed down by the pseudocontact shifts.^241^ This question was recently addressed by analyzing the SE between one electron and two protons (three-spin SE) using a trityl radical in a glassy water/glycerol matrix.^242^ It was demonstrated that the size of the spin diffusion barrier is less than 0.6 nm, and that the protons involved in the initial transfer step reside on the trityl species.

#### 3.3.2 Practical Aspects

**Figure 23:**
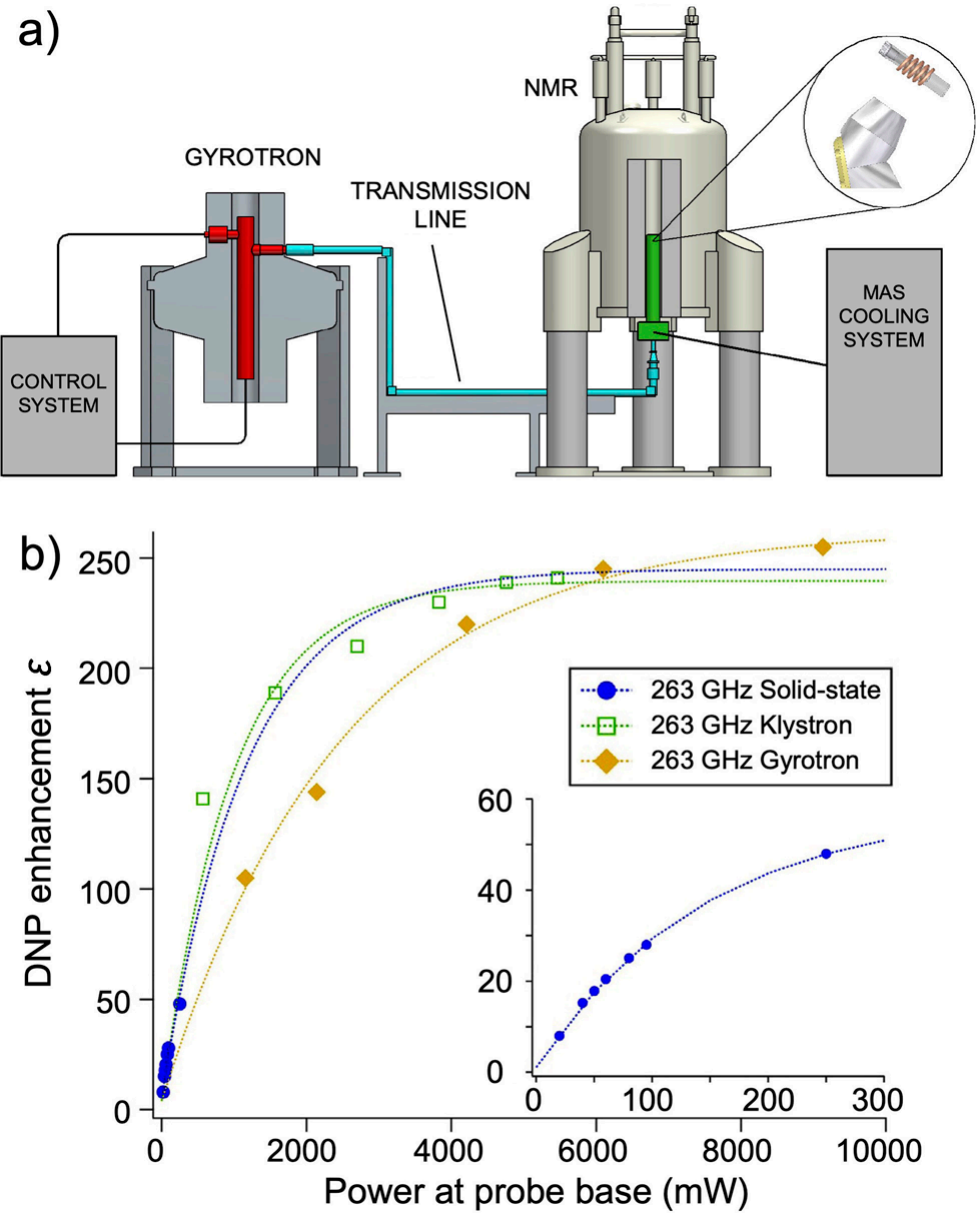
(a) Schematics of a solid-state DNP MAS NMR system with a gyrotron microwave source (gyrotron tube in red), microwave transmission line (cyan) and low-temperature NMR probe (green). (b) Proton DNP enhancement factors measured on a Proline/AMUPol standard sample in a 3.2 mm standard rotor as a function of the MW power for various microwave sources: solid-state source, klystron, and high-power gyrotron. The inset shows the 100 and 250 mW solid-state source data only. (a) Reproduced with permission from ref (244). Copyright 2016 Elsevier. (b) Reprinted with permission from ref (246). Copyright 2019 Elsevier.

DNP MAS NMR experiments require the availability of dedicated instrumentation. As mentioned previously, DNP MAS NMR was not accessible at high magnetic fields until the 1990s, when Griffin and co-workers proposed the use of high-frequency and high-power gyrotrons to generate continuous microwave irradiation. In parallel, they developed cryogenic MAS probes to carry out experiments at low temperatures where the saturation of the electron spin transitions is facilitated and their polarization is larger. Commercial DNP hardware is nowadays available from Bruker Biospin, which is built according to the design initially proposed by Griffin’s group.^243,244^ In this design, microwaves are generated by fixed-frequency gyrotrons capable of producing 50 W at 263 GHz (9.4 T) and 395 GHz (14.1 T), and 20 W at 527 GHz (18.8 T). The microwaves are supplied to the NMR probe via a transmission line that irradiates continuously a 3.2 (sapphire), 1.3, or 0.7 mm (zirconia) rotor (Figure 23a). The NMR magnet is equipped with a sweep coil, the current in which is adjusted to achieve an optimal field for DNP transfer. The probe cooling is achieved via three independent cold nitrogen gas channels (the bearing, drive, and variable temperature gas) through a liquid nitrogen heat exchanger. A sample temperature of around 100 K is typically reached, which can be measured by inserting microcrystalline KBr in the NMR rotor. The distribution of the microwaves strongly depends on the design of the probe, such as the use of converging lenses or spacing of the windings of the RF coil, as well as on the rotor diameter and the magnetic field.^245^

Furthermore, various groups have recently introduced ultralow-temperature (∼30 K) NMR probes suitable for MAS NMR experiments. Several designs have been proposed, where helium is used as cold gas for sample cooling and spinning within low-cost, closed loop systems.^247,248^ At these low temperatures, a substantial improvement in the DNP polarization transfer is expected due to prolonged electron relaxation times. A proton DNP enhancement factor as high as 677 was thus reported at a sample temperature of 55 K.^249^ Ultralow temperatures also open the possibility of exploiting compact, low-power microwave sources, such as solid-state diode sources or klystrons. These latter have been shown to be also suitable for DNP MAS NMR at around 100 K,^250^ although smaller enhancements than those achievable with high-power gyrotrons were reported. More recently, it was demonstrated that 250 mW diode sources operating at 263 GHz could be employed to get sizable proton enhancements of up to 120 at the sample temperature of 100 K.^246^

While these low-cost devices currently provide lower DNP enhancement factors than high-power sources (Figure 23b), they can be rapidly swept over a wide range of frequencies and can generate pulses of microwave radiation, thus holding promise for the near-future developments in the DNP MAS NMR instrumentation. Finally, we note the recent demonstration that electron decoupling (though not yet available on commercial instrumentation) could be beneficial for improving spectral resolution in DNP MAS NMR.^251^

A significant number of polarizing agents have been proposed as exogeneous electron sources to enable efficient solid-state DNP under MAS. They mostly belong to four main families: dinitroxides, hetero- or hybrid biradicals, derivatives of BDPA, and paramagnetic metal ions. The design of efficient polarizing agents for DNP MAS NMR is an extremely active area of research, with continuous progress over the last 20 years. Here, we briefly review the stepwise advances that have led to the paramagnetic agents in use today as well as their relative merits, notably with respect to their DNP performance at high magnetic fields and/or high MAS frequencies. Dinitroxides and heterobiradicals invoke CE DNP, whereas BDPA derivatives are suitable for SE and OE DNP, and paramagnetic metal ions for SE DNP.

While the first DNP MAS NMR experiments were performed using samples doped with high concentrations of the nitroxide monoradical 4-amino-TEMPO,^224^ it was demonstrated in the mid-2000s that dinitroxides, in which two TEMPO radicals were linked together to control the size of their electron–electron dipolar interaction, provided significantly higher DNP enhancement factors.^252^ This discovery led to the introduction of TOTAPOL dinitroxide biradical^253^ (shown in Figure 16a) that has been used by the DNP MAS NMR community for more than a decade. Rigid tethers constraining the mutual orientation of the two TEMPO moieties in a nearly orthogonal conformation were later proposed with the bis-TEMPO-bisketal (bTbK) biradical (Figure 16a).^254^ The design of bulky radicals having long electronic relaxation times facilitating the saturation of the EPR lines was another landmark achievement that led to the introduction in 2013 of TEKPol^237^ and AMUPol^255^ (Figure 20). Today, these two biradicals are among the most efficient polarizing agents for DNP MAS NMR at intermediate magnetic fields, providing proton enhancement factors of ∼200–300 at 9.4 T and 100 K in frozen organic and aqueous solutions, respectively. Several variants were proposed.^256,257^ Notably, the importance of the local (open) geometry around the unpaired electrons was recently demonstrated, and new, highly efficient, urea-based biradicals introduced.^258^

While AMUPol- or TEKPol-like systems remain the polarizing agents of choice for today’s DNP MAS NMR, their performance drastically degrades with increasing magnetic fields. This agrees with theoretical analyses of CE DNP with dinitroxides under MAS conditions, predicting that the probability for electron–electron–nucleus three-spin crossings scales down with the magnetic field.^235,236^ As a consequence, these dinitroxides become inefficient at magnetic fields approaching ∼18 T. Thus, the proton enhancements achieved with AMUPol and TEKPol typically drop from ∼200–300 at 9.4 T to ∼10–30 at 18.8 T.

One approach to obtaining larger enhancements at high fields consists in implementing CE DNP with radicals with narrow EPR lines. A direct ^13^C CE DNP enhancement at 5 T with a solution of sulfonated BDPA (SA-BDPA) and trityl radicals achieved by saturating the slowly relaxing SA-BDPA moiety was reported in 2013.^259^ In 2015, a new family of biradicals was introduced in which the trityl radical characterized by a narrow EPR line and a nitroxide are chemically tethered.^260^ In contrast to dinitroxides, these hybrid biradicals, dubbed TEMTriPols, yield DNP enhancements that increase with magnetic field. A proton signal enhancement factor of 65 at 18.8 T (two times higher than with AMUPol at this field strength) was reported with the best radical in this series, TEMTriPol-1. This work notably highlighted the key role of exchange coupling in the design of biradicals for DNP in very high fields. Several TEMTriPol derivatives were recently proposed for efficient DNP MAS NMR of biomolecules.^261^

Along similar lines, a new family of heterobiradicals consisting of a BDPA moiety linked to a nitroxide were introduced in 2018.^233^^1^H DNP enhancement factors of 180 at 18.8 T and 60 at 21.1 T were reported with HyTEK2, the best radical in the series. Importantly, hybrid or heterobiradicals show a favorable spinning-frequency dependence and no or little depolarization.^262^ In other words, the absolute sensitivity gain that they provide does not decrease with increasing MAS frequencies, in contrast to the case for AMUPol- or TEKPol-like radicals. Based on the idea that relatively high magnetic interactions led to improved DNP performance at high magnetic fields, the structure of nitroxide biradicals was recently revisited. New water-soluble dinitroxides, dubbed AsymPolPOKs^263^ and TinyPols^233^ were proposed that substantially outperform AMUPol at 18.8 T.^264^ Notably, proton DNP enhancement factors as high as 90 and 38 were reported with the TinyPol biradical in bulk frozen solutions at 18.8 and 21.1 T, respectively.

BDPA derivatives are another family of polarizing agents suitable for DNP MAS NMR. As already briefly mentioned, they recently received a renewed interest after the discovery that these radicals provided efficient OE DNP at high magnetic fields.^240^ Proton sensitivity enhancement factors of up to 100 could be achieved at 18.8 T with BDPA in OTP, a solvent that forms a rigid glass at cryogenic temperatures.^265^ However, OE DNP in insulating solids currently requires build-up times of several tens of seconds, which may compromise the gain in overall sensitivity. The molecular stochastic motions responsible for OE DNP in BDPA have been elucidated by molecular and spin dynamics simulations^212^ and mixed-valence compounds have been recently proposed as efficient polarizing agents for OE DNP at high field.^266^

Finally, paramagnetic transition metal and lanthanide complexes can also be employed as the electron source for DNP MAS NMR. High-spin Gd^3+^ and Mn^2+^ metal complexes^267^ were used to efficiently polarize low-γ nuclei such as ^13^C and ^15^N via the SE. Recently, a proton enhancement of 37 was measured at 100 K and 9.4 T in aqueous solutions for [Gd(tpatcn)] (tpatcnH_3_ = 1,4,7-tris[(6-carboxypyridin-2-yl) methyl]-1,4,7-triazacyclononane).^258^ These paramagnetic metal complexes represent promising alternative polarizing sources to dinitroxides, hybrid biradicals, or BDPA derivatives, notably in reducing environments, as discussed below.

Figure 20 shows the molecular structure of some of the most efficient polarizing agents available today for DNP MAS NMR in aqueous or organic solutions at both intermediate and high magnetic field regimes, and Table 1 summarizes their DNP performance. This latter is usually assessed from the enhancement factor ε defined as the intensity ratio in NMR spectra recorded with microwaves on and off. However, other parameters have to be considered in order to evaluate the real, overall sensitivity gain provided by DNP. In particular, the polarization build-up time as well as the magnitude of the depolarization induced by the paramagnetic agent and its propensity to quench part of the sample by paramagnetic effects are key factors that affect the efficiency of a polarizing agent. Several articles and reviews have recently described the relevance of these factors for a proper comparison of radicals’ performance under various experimental conditions.^268,269^

**Table 1:** Comparison of the Performance for the Radicals Depicted in Figure 20^a^

Radical	DNP matrix	*B*_0_, T	ω_r_/2π, kHz	ε	θ	τ_DNP_, s	Refs
AMUPol	10 mM in glycerol-*d*_8_/D_2_O/H_2_O 60/30/10 (v/v/v)	9.4	40	290	n. d.	3.8	(264)
AMUPol	10 mM in glycerol-*d*_8_/D_2_O/H_2_O 60/30/10 (v/v/v)	18.8	40	48	0.46	10	(264)
AMUPol	5 mM in glycerol-*d*_8_/D_2_O/H_2_O 60/30/10 (v/v/v)	21.1	12	18	0.64	14.4	(264)
Gd(tpatcn)	5 mM in glycerol-*d*_8_/D_2_O/H_2_O 60/30/10 (v/v/v)	9.4	8	37	n. d.	8.6	(270)
TinyPol	5 mM in glycerol-*d*_8_/D_2_O/H_2_O 60/30/10 (v/v/v)	9.4	40	225	n. d.	11.2	(264)
TinyPol	5 mM in glycerol-*d*_8_/D_2_O/H_2_O 60/30/10 (v/v/v)	18.8	40	73	0.73	15.5	(264)
TinyPol	5 mM in glycerol-*d*_8_/D_2_O/H_2_O 60/30/10 (v/v/v)	21.1	12	29	0.59	13.3	(264)
TEMTripol-1	10 mM in glycerol-*d*_8_/D_2_O/H_2_O 60/30/10 (v/v/v)	18.8	8	65	0.84	3.7	(260, 262)
TEKPol	16 mM in TCE	9.4	8	205	0.65	3	(256)
TEKPol	16 mM in TCE	18.8	40	13	0.71	4.5	(233)
HyTEK2	32 mM in TCE	9.4	40	143	n. d.	1.6	(233)
HyTEK2	32 mM in TCE	18.8	40	185	0.70	3.3	(233)
BDPA	60 mM in 95% OTP-d_14_	9.4	8	140	n. d.^b^	35.5	(231)
BDPA	60 mM in 95% OTP-*d*_14_	18.8	40	105	1.0	45	(265)

a *B*_0_ is the magnetic field, ω_r_ the sample spinning frequency, ε the enhancement factor measured from the ratio of proton signal intensity in the spectra acquired with microwaves on and off, τ_DNP_ the polarization build-up time with microwaves on, and θ the contribution factor, measured as the ratio of signal intensity in spectra of a frozen solution with and without the radical. This latter considers both depolarization and paramagnetic bleaching effects.

b n. d. - not determined.

Sample formulation is very important in DNP MAS NMR, and many efforts have been devoted to developing widely applicable sample formulation procedures providing high enhancement factors on the systems of interest. The most common procedure for intrinsically radical-free substrates consists in embedding them into a glass-forming matrix that contains the polarizing agent. Glycerol/water or DMSO/water mixtures (also known as “DNP juices”; DMSO is dimethyl sulfoxide) as well as solvents such as TCE or organic phases such as OTP are frequently employed as DNP polarizing matrices. Solutions that crystallize (i.e., do not form a glass) upon freezing lead to an inhomogeneous distribution of the radicals and to poor DNP efficiency. For powdered molecular solids and organic, inorganic and hybrid materials, samples for DNP experiments are usually prepared by incipient wetness impregnation (IWI).^271^ In this approach, the dry powders are impregnated with a minimal amount of the radical-containing solution. This latter either penetrates the pores of porous materials by capillary action, or simply wets the surface of nonporous systems. In contrast, most successful experiments on biomolecules have relied on dissolving or immersing them in the solution containing the polarizing agent before precipitation or sedimentation, while cellular preparations are typically formulated from pellets resuspended in a radical matrix. The solvent phase can possibly be removed by drying the sample after impregnation in the so-called matrix-free approach^272^ that has been demonstrated on a range of biomolecular systems including membrane proteins. Alternative matrices such as acrylamide gels of various cross-linking densities have been proposed for substrates prone to aggregation upon sample cooling such as quantum dots.^273^ Colloidal nanoparticles can also be dispersed in mesoporous silica^274^ or over a support material with favorable dielectric properties after immersion in the radical solution.^275^

The overall proton density in the DNP sample is a key parameter, and partial deuteration of the polarizing matrix is often used to channel the polarization more efficiently from the unpaired electrons to the protons of a substrate. The concentration of the polarizing paramagnetic agent is another crucial factor, as it determines not only the signal enhancement factor ε and the build-up time of the enhanced polarization, but also paramagnetic bleaching (i.e., the fraction of substrate that cannot be observed due to strong paramagnetic effects induced by the unpaired electrons of the dopant), broadening of NMR lines and reduction of nuclear spin coherence times. In practice, the optimal radical concentration is often considered as the one providing the highest overall sensitivity gain (as defined previously), or the highest signal-to-noise ratio per unit time and per unit of mass for the signals of interest. All these concentration effects have been carefully investigated in model frozen glassy matrices,^268,276−279^ as well as in real samples. While optimal concentration typically lies between 5 mM and 20 mM, a universal radical concentration does not exist, notably as the affinity of the radical for the substrate and therefore local concentrations may enter into play. Thus, the optimal radical concentration to investigate surfaces is not the same across materials but depends on their surface area as well as on interactions with functional groups.^280^

The potential reduction of the radical by the substrates of interest is another element to consider when formulating the DNP sample. Dedicated strategies have thus been designed to investigate surfaces where highly reactive species (e.g., Lewis acidic metal centers in heterogeneous catalysts) lead to the degradation of the exogenous polarizing agent and/or to a modification of the properties of the material (e.g., deactivation of the catalyst by coordination of the free radical). Polarizing agents that encapsulate the radical into hydrophobic carbosilane dendrimers have thus been proposed to inhibit a direct contact with reactive surfaces and restore sizable enhancement factors.^281^ Deleterious interactions with the exogenous dopant could also be reduced by tuning the surface hydrophobicity of nanoparticles, so as to promote their aggregation in the polarizing solution, which in turn prevents a (bulky) radical from diffusing between particles and reacting with the surface fragments.^282^ It was recently shown that adsorption of pyridine molecules on the surface of a reactive material prior to IWI was an effective approach to prevent a close proximity between radicals and reactive acid sites.^283^ Along similar lines, a sterically hindered mononitroxide, less prone to reduction than AMUPol or TOTAPol-like molecules, was proposed for DNP MAS NMR of cells.^284^ Finally, we note that metal complexes were recently suggested as alternatives to dinitroxides for DNP formulations in reducing environments.^270^

**Figure 24:**
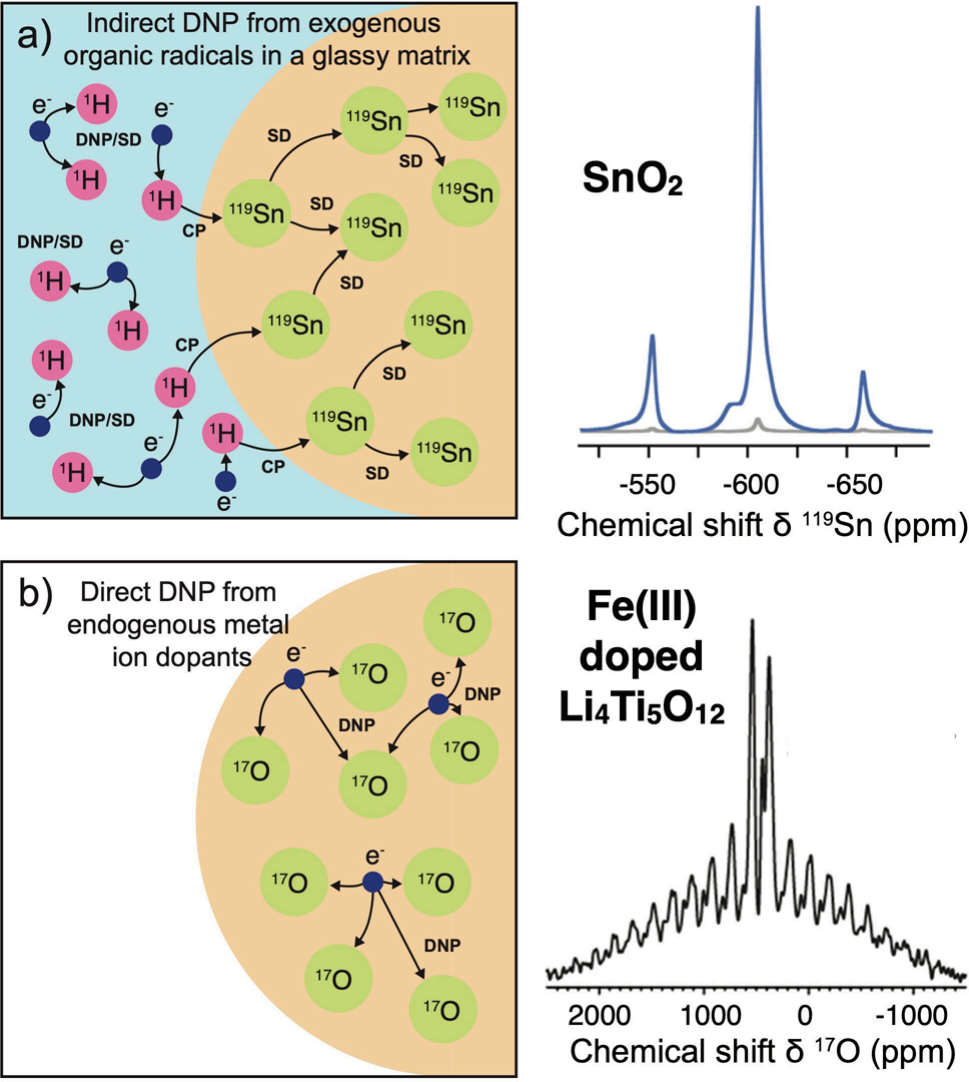
Formulations for DNP MAS NMR of proton-free inorganic solids. (a) The material is prepared by IWI with a radical-containing solution. A CP step transfers the magnetization from hyperpolarized protons in the DNP matrix to ^119^Sn nuclei at the surface. A mixing time (typically, several hundred seconds) placed between CP and signal acquisition enables the ^119^Sn hyperpolarization to be transferred from the surface to the bulk of the material by homonuclear ^119^Sn spin diffusion. Here, powdered SnO_2_ was impregnated with 16 mM TEKPol in TCE. A factor of 50 gain in overall sensitivity was reported. (b) The powdered solid contains a small quantity of metal ions that act as endogenous polarizing agents. The electron polarization is transferred directly to adjacent ^17^O nuclei in the absence of spin diffusion. Here, Li_4_Ti_5_O_12_ powders were doped with Fe(III) via solid-state synthesis. A ^17^O enhancement factor of around 280 was reported for a Fe(III) mole fraction of 0.005, enabling fast acquisition of ^17^O NMR spectra of the bulk material at natural abundance. (a) Reproduced with permission from ref (285). Copyright 2018 American Chemical Society. (b) Reproduced from ref (287). Copyright 2018 The Authors. Published by American Chemical Society under the CC BY license.

The formulations described above are especially efficient in enhancing NMR sensitivity of surface or subsurface layers of functional materials or the cores of protonated solid-state compounds (including biomolecules and microcrystalline molecular solids), where the enhanced nuclear polarization around electrons is efficiently transported from protons of the polarizing medium to the surface and then possibly to the bulk of the substrates by proton–proton spin-diffusion. As already briefly mentioned, spin diffusion among low-γ, spin-1/2 nuclei can also be exploited to relay nuclear hyperpolarization from the surface to the bulk of inorganic materials.^285^ Alternatively, specific formulation protocols have recently been proposed to polarize proton-free materials, consisting in doping inorganic solids with paramagnetic ions such as Gd(III) and Mn(II) distributed in the bulk sample that act as endogenous DNP agents.^286^ Such formulation protocols lift the requirement of efficient spin diffusion among rare nuclei and yield sizable direct DNP enhancements at the core of the inorganic substrate. Figure 24 summarizes the above-described DNP formulation efforts targeting proton-free systems.

Finally, we note that some materials or biological macromolecules have naturally occurring endogenous polarization sources such as stable organic radicals or paramagnetic metal ions^288,289^ that can be exploited as free electron sources for DNP MAS NMR. Also, it has been recently shown that γ-irradiation of inorganic materials may induce formation of stable radicals that are suitable for high-field DNP MAS NMR.^290^

#### 3.3.3 Applications

DNP-enhanced solid-state NMR under MAS is widely applied in materials science. Over the last ten years, it has developed as a unique methodology for characterizing the surface structure of organic, inorganic, and hybrid materials, in an approach called DNP SENS (DNP surface enhanced NMR spectroscopy). The large enhancement of the NMR signals provided by DNP is indeed particularly relevant to probing the structure of surfaces as the functionally relevant sites conferring their properties to functional materials are often diluted and as isotopic labeling on surfaces is often difficult, if not impossible. The first proofs of concept were reported in 2010 on model mesoporous silica matrices of high surface area.^271^ Since then, applications of DNP SENS have flourished and concern a large range of substrates such as high-performance organometallic or metal oxide catalysts, metal–organic frameworks, ligand-capped nanoparticles, quantum dots, zeolites, biominerals, and organic thin films. In these studies, the enhanced proton polarization is usually transferred to low-γ spin-1/2 nuclei (^13^C, ^31^P, ^29^Si, ^119^Sn, or ^15^N) located at the surface of the materials. The signal of surface quadrupolar nuclei, of interest in many modern inorganic or hybrid materials, can be similarly enhanced. Several ^27^Al DNP SENS NMR spectra have been reported for a variety of systems, including on-surface species having relatively large quadrupolar broadening.^291,292^ Successful DNP SENS experiments were also carried out on low-γ and/or low abundance nuclei such as ^17^O at natural abundance,^293^^43^Ca,^294^^89^Y,^295^^95^ Mo, ^47^Ti, or ^49^Ti,^296^ or on nuclei with extremely broad chemical shift anisotropy (CSA) patterns such as ^195^Pt.^297,298^ As is the case for conventional solid-state NMR, the detection of low-γ nuclei on surfaces can be facilitated by combining DNP with proton detection.^299^ Notably, a sensitivity gain by more than a factor of 8 by indirect detection for ^89^Y DNP MAS NMR was reported.^299^ For more details on the development of DNP SENS in materials science, the reader may refer to recent reviews.^300,301^ We further note that DNP MAS NMR brought new opportunities to the field of catalysis,^302^ from the characterization of the support itself to the full structure determination of single-site species or the identification of reaction intermediates.^303,304^

In the following, two selected recent case studies are described that illustrate the strong amplification power of DNP for NMR of surfaces and the unique structural insight that can be gained by this hyperpolarization technique.

**Figure 25:**
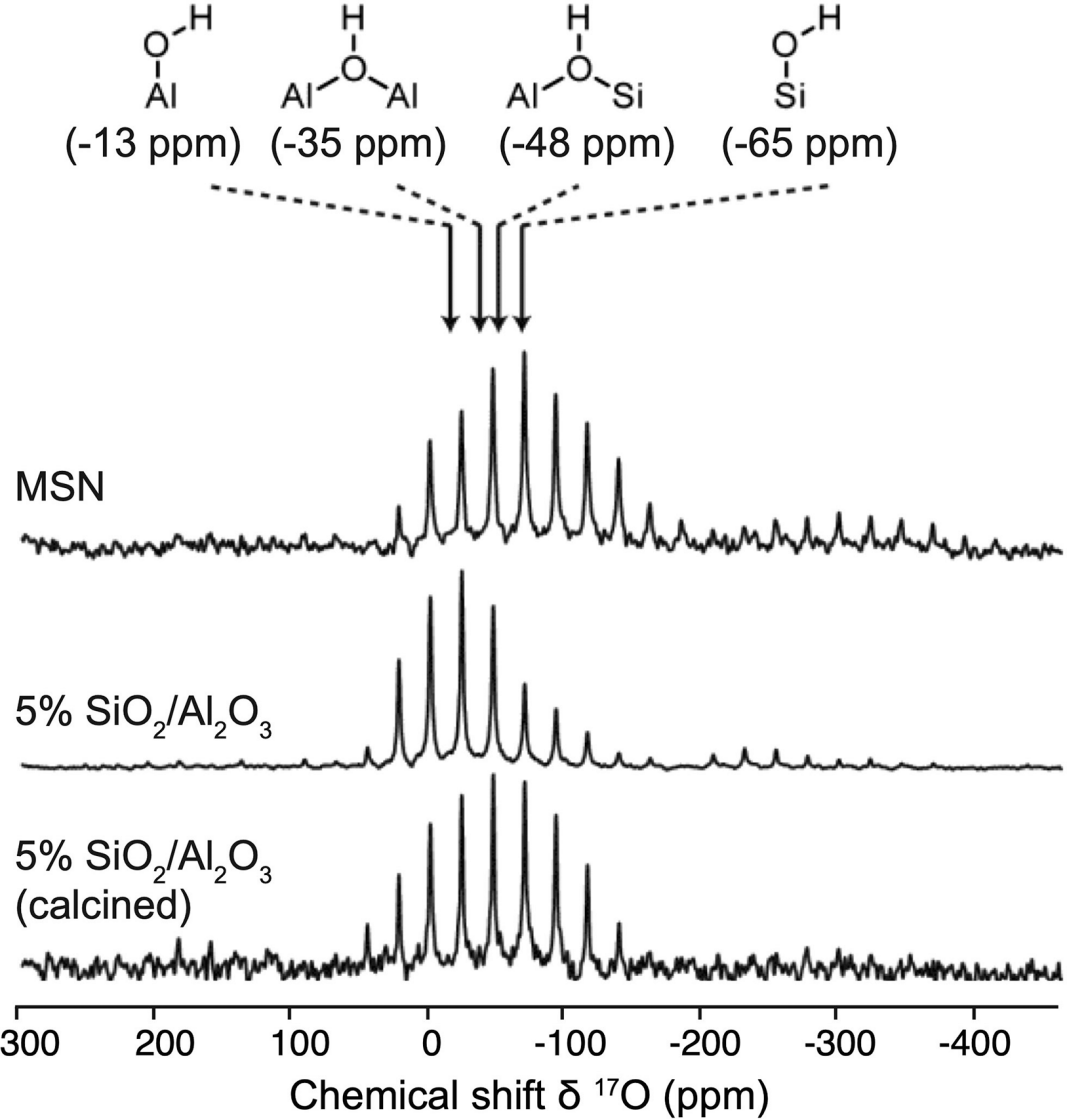
DNP-enhanced, natural-abundance, ^17^O{^1^H} PRESTO-QCPMG (phase-shifted recoupling effects a smooth transfer of order: quadrupolar Carr–Purcell–Meiboom–Gill) spectra acquired on various silica and silica–alumina samples (9.4 T, 12.5 kHz spinning frequency). The samples were impregnated with 15 to 30 mM TEKPol solutions. The centers of mass for four different hydroxyl environments are indicated at the top. The MCM-41-type mesoporous silica nanoparticle (MSN) sample presents a broad resonance centered at −65 ppm, as expected. The spectrum acquired on 5% silica–alumina features a dominant peak at around −13 ppm as well as a smaller shoulder at ca. −35 ppm, which were assigned to the μ1- and μ2-aluminols, respectively. After calcination, the μ1-aluminols are removed, and the signal is dominated by the resonance of acidic bridging hydroxyls. Adapted with permission from ref (293). Copyright 2017 Wiley-VCH Verlag GmbH & Co. KGaA, Weinheim.

The first example concerns the detailed investigation of Brønsted acidity of catalytic oxide supports by ^17^O DNP SENS.^293^ In this work, the authors were able to observe the surface ^17^O NMR signature at natural abundance for various silica and silica–alumina materials. The signals of aluminols, silanols and acidic bridging hydroxyls could be distinguished as illustrated in Figure 25. The authors were also able to measure the O–H bond length with high accuracy (subpm precision) by implementing DNP-enhanced 2D proton-detected local field experiments, which report directly on the lability of the OH protons and on the acidity of the oxide surface.

**Figure 26:**
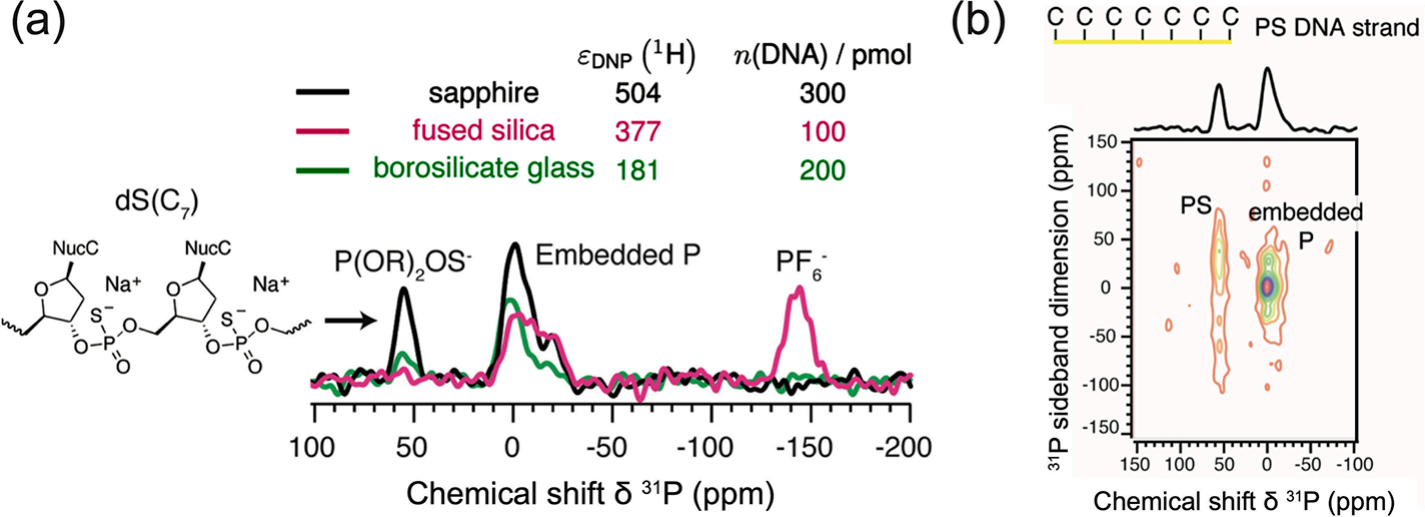
(a) One-dimensional DNP-SENS CP-CPMG ^31^P spectra from the given amounts of heptameric oligocytidine strands (dS(C_7_), C = cytidine) deposited on three different supports. The data were acquired at 9.4 T and the total acquisition time for each experiment ranged between 20 and 28 h. The polarizing agent, TEKPol or TEKPol2 dissolved in TCE, was added by IWI. The numbers indicate the proton DNP enhancement and maximum amount of functionalized DNA that was analyzed. Here, the phosphodiester (P(OR)_2_O_2_^–^) groups of DNA strands were replaced by phosphorothioester (P(OR)_2_OS^–^) functional groups, providing a unique ^31^P chemical shift signature near 55 ppm. The signal at −5 ppm corresponds to phosphate-like species embedded in the wafers. The fused silica system also exhibits a relatively strong signal around −145 ppm, corresponding to another bulk impurity. The specific surface area of the samples is <0.01 m^2^/g. (b) Two-dimensional DNP-SENS CP PASS-PIETA (phase adjusted spinning sidebands-phase incremented echo-train acquisition) ^31^P NMR spectrum from 200 pmol of dS(C_7_) strands deposited on sapphire. Vertical cross sections give spinning sideband profiles at the given isotropic shift, from which CSA parameters can be extracted. Reproduced with permission from ref (305). Copyright 2019 American Chemical Society.

The second example refers to the characterization of molecules immobilized on wafers.^305^ By combining DNP methods with Carr–Purcell–Meiboom–Gill (CPMG) acquisition and optimized sample formulations, ^31^P NMR spectra could be acquired from less than ∼100 pmol oligonucleotide functionalities deposited onto silicate glass and sapphire wafers with surface areas on the order of 0.01 m^2^/g, as illustrated in Figure 26a. Overall sensitivity enhancement factors of up to half a million and above with respect to conventional NMR experiments were reported. Such an improvement in sensitivity allowed the authors to perform 2D NMR experiments (Figure 26b), to probe conformational changes due to ion binding as well as to follow photochemical degradation reactions.

DNP-enhanced NMR spectroscopy has recently emerged as a high-sensitivity approach to probe not only the surface but also the core of inorganic materials. This has become possible thanks to recent formulation efforts discussed above (Figure 24). In particular, efficient DNP MAS NMR of bulk battery anode materials, phosphor materials, metal oxides, and quartz has been reported.^285−287,306^

Applications of DNP MAS NMR in pharmaceutical research date back to 2014 when Rossini et al. demonstrated in a landmark paper that the technique could be applied to characterize an active pharmaceutical ingredient (API) in over-the-counter drugs.^307^ By impregnating ground tablets with a polarizing solution chosen to be a nonsolvent for the solid compounds, DNP enhancement factors of between 40 and 90 were reported at 9.4 T for various commercially available formulations of the antihistamine drug cetirizine dihydrochloride, containing from 4.8 to 8.7 wt % API. The DNP-enhanced 1D and 2D ^1^H-^13^C and ^1^H-^15^N solid-state NMR spectra of the formulated APIs at natural abundance revealed direct contacts between the API and some of the polymer excipients. The experiments also enabled to measure in situ the size of the API domains within the complex superstructure of the formulated drugs from the variation of the DNP enhancement as a function of the polarization time or the interscan delay. Since then, other key developments were achieved in pharmaceutical research, with the demonstration that DNP-enhanced NMR spectroscopy could be applied to distinguish drug polymorphs,^308^ for instance from their ^35^Cl NMR signature in low wt% API-dosage forms,^309^ or to characterize the multicomponent structure of pharmaceutical salts and cocrystals,^310^ drug-delivery systems such as lipid nanoparticles,^311^ crystalline drug nanoparticles,^312^ or TEMPO-oxidized cellulose nanofibrils.^313^

In all these studies, the samples were prepared by IWI. An alternative approach was recently introduced for in situ DNP NMR investigation of pharmaceutical drugs.^314^ It was proposed to add polarizing agents directly during the preparation of the formulations, with amorphous solid dispersions (ASD) obtained by either spray drying or hot-melt extrusion, two processes widely used in pharmaceutical industry. Proton enhancement factors of up to 25 were reported for formulations doped with 1% AMUPol, which enabled rapid detection of the API NMR signature in commercialized compounds of low drug loading.

**Figure 27:**
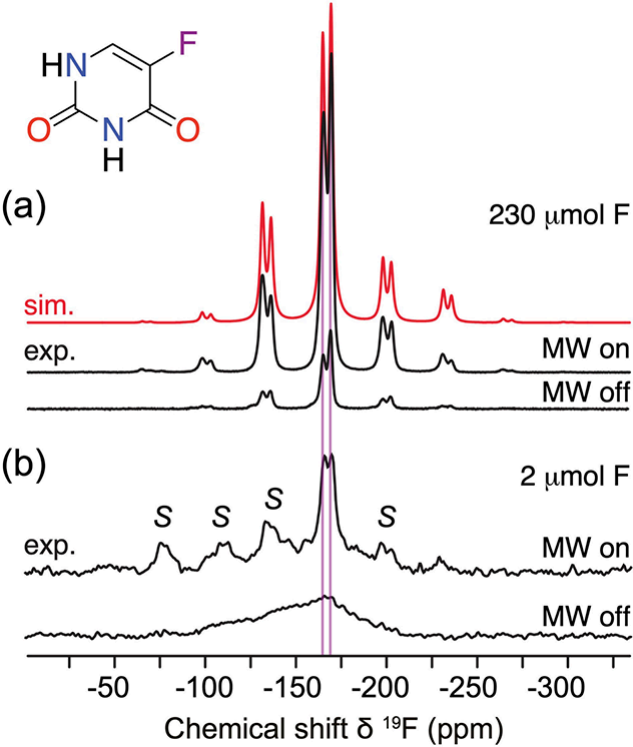
(a) ^19^F NMR spectra of 5-fluorouracil at 12 kHz spinning frequency, 9.4 T, and 110 K, acquired with (MW on) or without (MW off) microwave irradiation. Sample (a) corresponds to 30 mg of API, which contains 230 μmol of F atoms. Sample (b) is 0.293 mg of API mixed with 30.8 mg of cellulose, which contains 2 μmol of F atoms. Both samples were impregnated with AMUPol in trifluoroethanol-*d*_3_. Two crystallographically distinct sites are highlighted in purple and the other resonances in (a) represent spinning sidebands; the red trace corresponds to the simulated spectrum. In (b), the spinning sidebands are labeled with *S*, and correspond to the solvent (2,2,2-trifluoroethanol-d_3_) peaks. Reproduced with permission from ref (315). Copyright 2019 Wiley-VCH Verlag GmbH & Co. KGaA, Weinheim.

Many pharmaceutical compounds contain fluorine atoms. A combination of trifluoroethanol-*d*_3_ with 12 mM AMUPol was proposed as a versatile glassy matrix for DNP-enhanced ^19^F NMR spectroscopy, and the ^19^F resonances of the fluorouracil API diluted in an excipient matrix of cellulose were successfully detected, as illustrated in Figure 27.^315^ We finally note that DNP MAS NMR has been recently applied to provide for the first time an atomic-scale description of the interface between antigen and aluminum-based adjuvants in vaccine formulations and to identify differences in bonding strength depending on the adjuvant provider.^316^

DNP-enhanced MAS NMR has also established itself as a key spectroscopic approach in structural biology to characterize the structure of large biomolecular complexes such as amyloid fibrils, viral capsids and membrane proteins, to probe intermolecular distances in protein–ligand or protein–protein assemblies, and to elucidate structural elements of proteins directly in their cellular environments. Several recently published reviews provide an overview of the advances in biomolecular DNP NMR.^15,317^

Unlike the case of materials and microcrystalline molecular solids, the resolution of DNP-enhanced NMR spectra of biomolecules is drastically compromised. Indeed, various conformers that undergo rapid exchange at ambient temperature are frozen at the DNP cryogenic temperatures, which leads to broad chemical shift distributions. Those same cryogenic conditions, however, enable conformational ensembles to be directly examined,^318,319^ or transient species to be detected during protein folding and assembly.^320^

**Figure 28:**
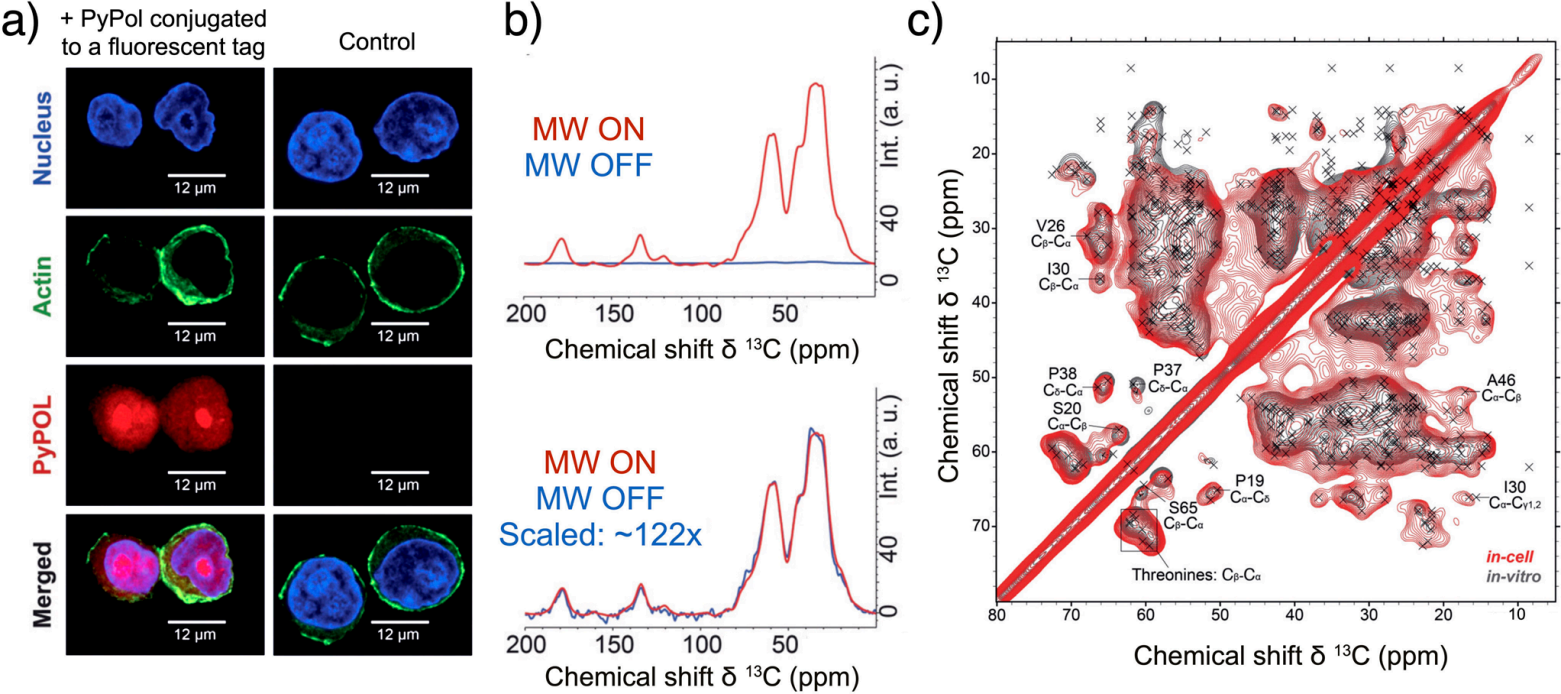
(a) Z-Slices of confocal microscopy images showing that PyPol radical is well distributed in both nuclear and cytoplasmic compartments of the cell. (b) DNP signal enhancement of approximately 122-fold was measured in ^1^H-^13^C CP-MAS experiments at 9.4 T and 8 kHz MAS frequency. (c) Aliphatic region of a 2D ^13^C-^13^C proton-driven spin diffusion (PDSD) experiment of ubiquitin in vitro (gray) and in cell (red). This comparison confirms that ubiquitin remains folded after delivery into the cells. Reproduced with permission from ref (321). Copyright 2019 The Authors. Published by Wiley-VCH Verlag GmbH & Co. KGaA.

Two representative studies are described below that highlight the outstanding potential of DNP in structural biology. The first refers to DNP MAS NMR of ubiquitin at endogenous concentrations in human cells,^321^ in which 1D and 2D in cell NMR spectra of ^13^C-labeled ubiquitin introduced in the cellular milieu by electroporation were detected with high sensitivity. The proper internalization and location of the radical was tracked by confocal microscopy using PyPol radical (a variant of AMUPol) conjugated to a fluorescent tag (Figure 28a). Proton DNP enhancement factors as high as 130 and 35 were reported at 9.4 T (Figure 28b) and 18.8 T, respectively, which enabled fast acquisition of two- and three-dimensional correlation spectra of both in vitro and in cell samples (Figure 28c). The assignment of the resonances suggested that ubiquitin remains folded after delivery into the cells.

The second study concerns time-resolved conformational changes during folding and self-assembly of melittin, a 26-residue peptide that forms a helical tetramer at high pH.^322^ Using a dedicated apparatus for rapid solution mixing, freeze-trapping and rotor filling, the sensitivity enhancement provided by DNP was exploited to monitor the NMR signature of intermediate states. It was demonstrated that helix formation in melittin after a rapid pH jump was concomitant with the appearance of intermolecular contacts characteristic of antiparallel dimers, as indicated by changes in chemical shifts as well as by the detection of intramolecular contacts in two-dimensional correlation spectra. Such an approach would be impractical without sensitivity enhancement by DNP.

#### 3.3.4 Frontiers and Challenges

DNP MAS NMR is a rapidly evolving field of research, with numerous ongoing developments in instrumentation, NMR methods, and formulation of the samples. The new directions for the 21st century have been recently reviewed.^323^ Here, we briefly mention some of these emerging frontiers.

As discussed above, while extensive research has been conducted on dinitroxides, these polarizing agents show limited efficiency at high magnetic fields and fast MAS. However, there is still much room for improvement in the chemistry of hybrid biradicals and biradicals with narrow EPR lines. The ideal polarizing agent for CE DNP at high field, which does not exist yet, would consist of two radicals with electron Larmor frequencies separated by the proton Larmor frequency. The development of metal-ion complexes for DNP remains at an early stage. The synthesis of new triradical structures may also expand the portfolio of effective DNP agents and open new windows of opportunity.^324,325^

In terms of instrumentation, the development of low-power, compact and cost-effective solid-state diode microwave sources in place of gyrotrons is expected to accelerate, and although currently limited to frequencies below 263 GHz, such sources will likely become more and more widespread. In parallel, we anticipate that microwave sources capable of phase-coherent pulses for electron decoupling and pulsed DNP methods^326,327^ will be developed. These technological advances, jointly with the introduction of sophisticated time-domain (non-CW) techniques, such as Nuclear Orientation Via Electron Spin Locking (NOVEL)^328^ (sections 3.9 and 3.10) or Time-Optimized (TOP) DNP,^329^ will open exciting experimental possibilities and will drive new applications in the future.^330^ Notably, pulsed DNP represents a potential solution to overcome the unfavorable field dependence of CW DNP as well as to reconcile this technique with applications at high temperatures.

Indeed, the requirement to perform CW DNP at cryogenic temperatures seriously restricts its applicability, notably for the study of solid biomolecules and for the investigation of dynamic and real-time events. Development of dedicated strategies suitable for elevated temperatures is thus one of the main challenges of DNP MAS NMR in the future. Rigid polarizing matrices that maintain high enhancement factors at ambient temperatures have been proposed and implemented to monitor conformational changes upon temperature increase in drug formulations.^231^ Recently, conduction electrons of lithium metal were used for room-temperature OE-DNP NMR of battery materials, demonstrating that NMR signals of molecular species at the solid-electrolyte interface could be selectively enhanced.^289^ These latter experiments open future avenues for in situ and operando DNP-enhanced NMR of working materials.

In structural biology, following the recent observations at 18.8 T and fast MAS,^331^ an increase in resolution is envisioned for DNP NMR at even higher magnetic fields, where the homogeneous component of the NMR lines will be largely reduced, a regime which should facilitate in vitro or in cell structural investigation of large protein complexes and biomolecular machines.

### 3.4 Dissolution Dynamic Nuclear Polarization

#### 3.4.1 The Technique

Similar to OE-DNP (section 3.2) and MAS DNP (section 3.3), dissolution dynamic nuclear polarization (*d*DNP) exploits the much larger polarization of electron spins as a source (section 2.2.1.4) to enhance nuclear spin polarization, theoretically, by factors of up to γ_e_/γ_n_, i.e., |ε| ≈ 658 for ^1^H and even more for lower-γ nuclear spins such as ^13^C, ^15^N, etc. (Figure 4). Microwave irradiation is applied at a frequency which is marginally nonresonant with respect to the electron spin resonance frequency, which enables a flow of electron spin polarization directly to nearby nuclear spins of interest.^332,333^ The major difference with other DNP techniques is that *d*DNP aims to enhance nuclear spin polarization at a significantly lower temperature, i.e., in experimental conditions where electron spins are substantially polarized even in a moderate magnetic field (for example, *p*_e,therm_ ≈ 99.93% at 1.2 K and 7.05 T, see Figure 4).^334^ Under microwave (MW) irradiation, the out-of-equilibrium electron–nuclear spin system is driven back toward its initial state population hierarchy via relaxation phenomena. This redistribution process is dominated by electron spin relaxation, which is orders of magnitude faster than nuclear spin relaxation. The longitudinal electron relaxation time constant (*T*_1e_) is typically of the order of ∼100 ms at liquid helium temperatures,^335^ while the nuclear spin–lattice relaxation time constant (*T*_1n_) is on the order of tens of minutes to hours. Efficient saturation of electron spin transitions results in increased nuclear spin polarizations with remarkable enhancements with respect to thermal equilibrium nuclear polarizations. Finally, as the name of the technique implies, the frozen polarized sample is converted into the liquid state with retention of a substantial fraction of nonequilibrium nuclear spin polarization.

The DNP mechanism(s) operating in the presence of microwave irradiation hinges on the nature and concentration of the polarizing agent, the nuclear spins of interest, the temperature and the static magnetic field strength. The three predominant mechanisms present under *d*DNP conditions are the solid effect (SE), the cross effect (CE) and thermal mixing (TM).

**Figure 29:**
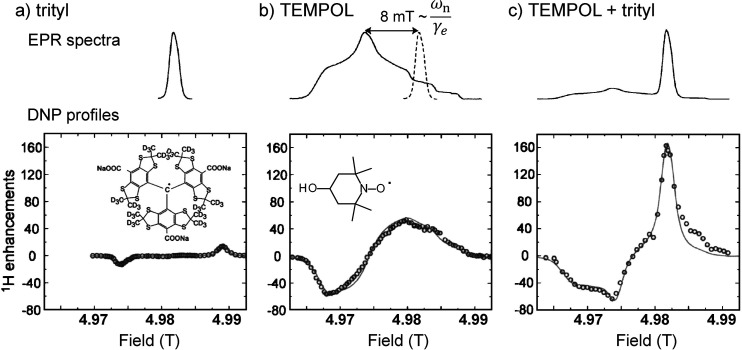
Radical species demonstrating: (a) SE; (b) CE; and (c) SE + CE, shown with the corresponding EPR spectra and DNP profiles. Adapted from ref (230) with the permission of AIP Publishing.

The two-spin SE mechanism is operative in a dipolar-coupled single-electron–single-nucleus spin system and is most efficient at the microwave irradiation frequency ω_MW_ = ω_e_ ± ω_n_ with radicals possessing a relatively narrow EPR line. The resulting NMR signal enhancement (DNP profile) at these matching conditions^230^ is seen in Figure 29. The CE is a two-electron–single-nucleus spin mechanism with the matching condition ω_e1_ – ω_e2_ = ω_n_, best implemented using radicals with a wide EPR line such as the nitroxide TEMPO(L) or biradicals.^336^ CE DNP can sometimes also be observed at reduced radical concentrations,^259,337−339^ despite the low probability of finding two radicals close enough to each other to ensure sufficient coupling of their electron spins and with such orientations that their EPR frequencies are separated by ω_n_. As both SE and CE are widely used in MAS DNP, they are described in more detail in section 3.3. In contrast, TM is only active at low temperatures (typically ≤4.2 K) where the inhomogeneously broadened EPR line is characterized by fast spectral diffusion between spin packets at different frequencies and behaves highly collectively under microwave irradiation due to a strongly coupled spin system and slow relaxation. Clearly, a large number of spin packets within a wide-line EPR spectrum do not fulfill the CE condition. Rather, at lower temperatures the TM mechanism becomes effective when *T*_1e_ is sufficiently lengthened and spectral diffusion for electron spins over the entire EPR line width is accomplished on a time scale less than *T*_1e_. The TM mechanism is particularly active in homogeneously broadened electron spin systems, i.e., at relatively high electron concentrations.

The low concentration of free electron spins compared with nuclear spins of interest in a typical *d*DNP-compatible sample implies that only clusters of nuclear spins localized in the vicinity of the polarized electron spins become hyperpolarized directly. Nuclear spin diffusion can then effectively spread the existing polarization throughout the sample if the bulk nuclear spins are in dipolar contact with a polarized nuclear spin,^340^ although with less efficiency for the nuclear spins in the immediate vicinity of the paramagnetic center (the “core”), since the unpaired electron can dramatically shift the resonance frequencies of the nearby nuclear spins.^341^ Ultimately, hyperpolarization of nuclear spins spreads by spin diffusion to nuclei in the electron-spin-depleted regions of the frozen sample which do not participate in direct hyperpolarization. It should also be noted that the NMR signals deriving from the core nuclear spins are “bleached” due to large frequency shifts and short nuclear spin–spin relaxation time constant (*T*_2n_) attributed to strong hyperfine interactions with nearby electron spins. The enhanced NMR signals therefore largely correspond to bulk nuclear spins which are hyperpolarized via nuclear spin diffusion.

#### 3.4.2 Practical Aspects

**Figure 30:**
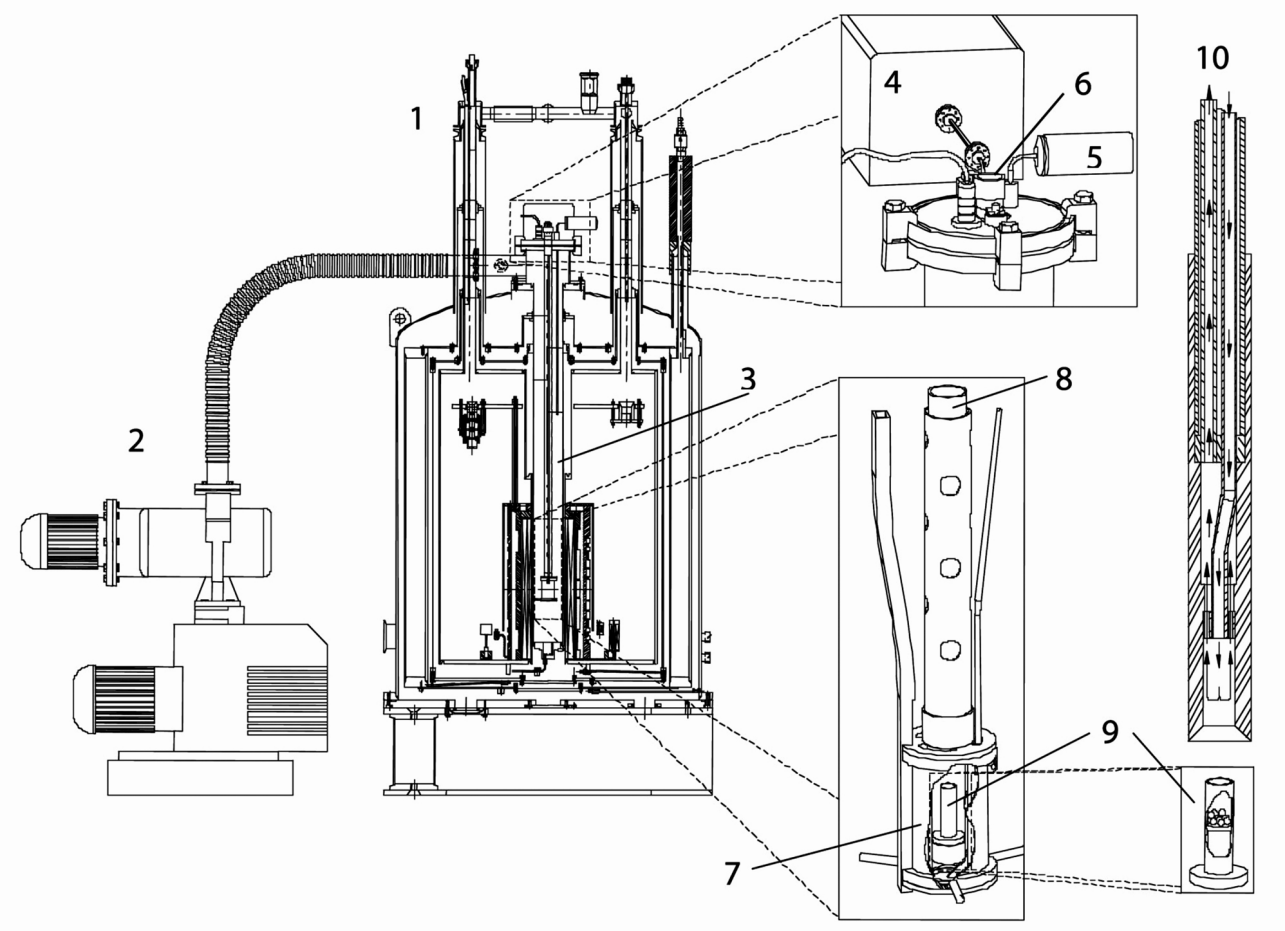
Schematic representation of the *d*DNP instrumentation used for the first successful *d*DNP experiments. 1 - polarizer; 2 - vacuum pump; 3 - variable temperature insert; 4 - microwave source; 5 - pressure transducer; 6 - sample port; 7 - microwave container; 8 - sample holder; 9 - sample container; and 10 - dissolution stick. Reproduced with permission from ref (342). Copyright 2003 National Academy of Sciences.

Ardenkjær-Larsen and co-workers first presented their *d*DNP instrumentation back in 2003^342^ (Figure 30). Two key system requirements were clearly necessary: (1) low temperatures (*T*) commonly provided by a bath of liquid helium with 1.2 K < *T* < 4.2 K; and (2) a sufficiently strong magnetic field (*B*_0_), instrumentation for state-of-the-art *d*DNP experiments provides a magnetic field *B*_0_ in the range from 3.35 to 7.05 T. Recently, *d*DNP at higher magnetic fields has also been achieved.^343,344^ A major part of a *d*DNP instrument, the “polarizer”, is composed of a superconducting NMR magnet and a cryostat with a reservoir of liquid helium to keep the sample suitably cold for extended periods of time (many hours). Cryostats specifically designed for *d*DNP experiments minimize consumption of liquid helium and have tailor-made components for sample insertion/dissolution and microwave irradiation.

Another important part of a *d*DNP instrument is a microwave source and the system for delivery of microwaves to the sample region. The microwave frequency itself is defined predominantly by the magnetic field strength of the polarizer, and minimally by the choice of target nuclear spins to be polarized. A beam of microwave radiation, although not necessarily coherent, is required. It is therefore possible to use oversized waveguides to reduce power attenuation during microwave delivery. Precisely positioned mirrors are housed within the waveguide, directing the microwave beam through the radiofrequency (RF) probe (see below) to reach the sample volume. Microwave power of a few tens of mW at the position of the sample is sufficient to saturate a specific part of the EPR spectrum, and as a result, microwave powers as low as only 25 to 100 mW are typically used.^345^

An RF probe placed within the bore of the *d*DNP polarizer fixes the position of an RF coil structure at the region with the highest and most homogeneous magnetic field *B*_0_. Running through the RF probe center is a pathway for an insertion stick to position a sample cup inside the RF coil network. The RF probe also firmly supports coaxial cables, permitting excitation RF pulses and NMR signals to propagate to and from the RF coil (sometimes inductively coupled) via a preamplifier to the NMR spectrometer. Being able to monitor the polarization build-up for a sample by detecting NMR signals throughout a *d*DNP experiment is possible with instrumentation such as the Oxford HyperSense polarizer. However, the need for accurate quantification of nuclear spin polarization and the ability to acquire quantitative thermal equilibrium NMR signals without DNP but with high levels of sensitivity and stability drives the development of highly sophisticated instrumentation, such as the prototype polarizer produced by Bruker Biospin. The majority of RF probes for *d*DNP are only suitable for one-channel RF irradiation, and typically the build-up of ^13^C polarization is primarily followed during the microwave irradiation time period.

**Figure 31:**
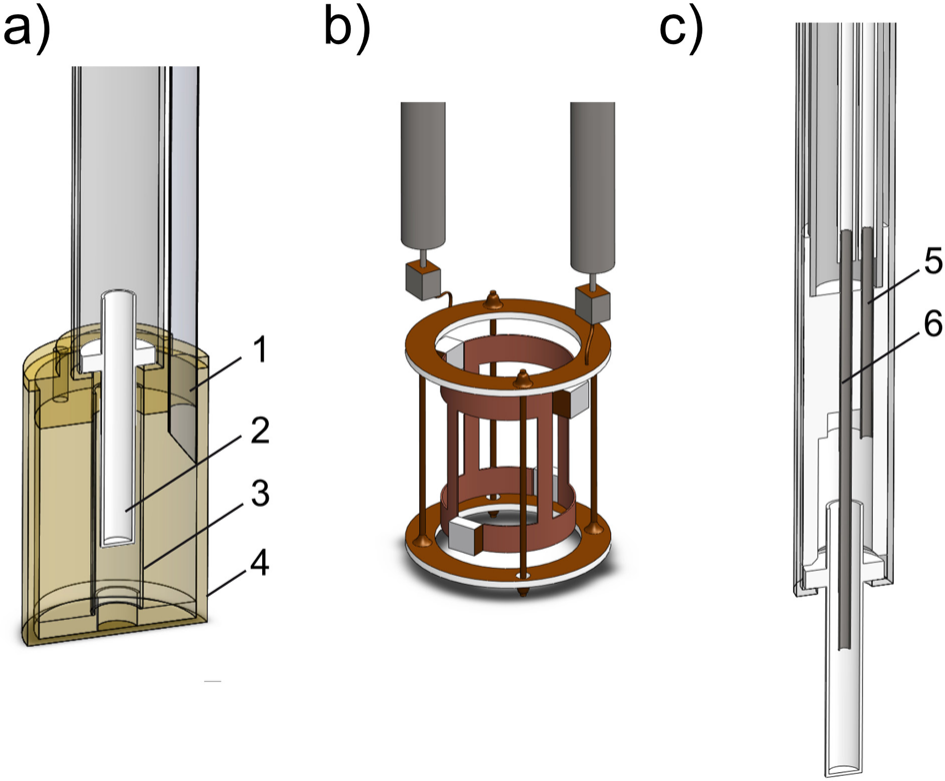
(a) DNP insert, including a waveguide (1a), a 5 mm inner diameter Vespel sample holder (2a), a glass RF coil support together with a doubly tuned NMR RF coil (3a), and a microwave cavity (4a). (b) Resonant RF circuit used for CP, composed of one pair of saddle coils tuned for ^1^H and one orthogonal pair for ^13^C, plus two saddle coils for inductive coupling. (c) Dissolution insert with the sample holder for rapid dissolution, with (1c) an outlet for the hyperpolarized solution and (2c) an inlet for the hot dissolution solvent. Adapted with permission from ref (351). Copyright 2013 American Chemical Society.

In the past, several designs for RF coils were proposed in order to monitor signal build-up during *d*DNP experiments. Initially, such designs were intended for the detection of a single type of nuclear spins (^13^C in particular) as, for example, in the Oxford HyperSense polarizer and later on in the GE SpinLab polarizer, as well as in some home-built *d*DNP equipment.^346,347^ Such a design is typically based on a saddle coil, large enough to allow sample dissolution and access from the top, connected via a coaxial line (typically, 0.5–1 m long) to a remote tuning and matching circuitry located outside of the cryostat. Such designs may provide poor sensitivity and low RF field strengths *B*_1_ but are fully adequate to monitor polarization build-up. In 2010, preliminary results were reported on the use of a doubly tuned NMR circuit in the context of *d*DNP in order to perform ^1^H to ^13^C cross-polarization (CP) experiments.^348^ This concept was developed further and resulted in a first publication of a working CP RF coil under *d*DNP conditions at 3.35 T (although incompatible with dissolution at that stage).^349^ This first design was based on a locally tuned 4 mm diameter horizontal solenoidal coil immersed in a liquid helium bath at 1.2 K together with all the tuning and matching elements. This provided a means to afford high quality factors and thus perform efficient CP experiments with *B*_1_ values corresponding to nutation frequencies exceeding 60 kHz on both channels. This first study reported sensible improvements for ^13^C DNP, both in terms of the final polarization attained and the acceleration of signal build-up. This concept was further implemented in a polarizer operating at 6.7 T, resulting in ^13^C polarizations exceeding 70% in 20 min,^350^ and then coupled with sample dissolution.^351^ The RF coil design became more sophisticated,^352^ and consisted of two separate locally tuned coils for ^1^H and ^13^C inductively coupled (no electrical contact) to the coaxial line (Figure 31). Similar techniques were subsequently implemented by other groups using the solenoidal design,^353^ and later^354^ the Alderman-Grant RF coil design with local double-tuning and matching capabilities.

One major downside of *d*DNP instrumentation is the high liquid helium consumption that working near 1 K implies, and its associated cost. This can be circumvented by the use of cryo-consumption-free instruments. Implementations of such hardware within the *d*DNP community are discussed in the literature.^343,344,355,356^ Liquid helium is replenished by recondensation of evaporated helium by a cryo-cooler integrated into the cryostat-magnet system. “Conventional” polarizers typically require 50–100 L of liquid helium per week (typ. $15–25 per liter, current pricing). Future versions of commercial equipment will inevitably feature a cryo-consumption-free technology which is already implemented in the GE SpinLab polarizer.

*d*DNP-compatible samples need to become glassy when frozen to ensure a random distribution (i.e., no aggregation) of polarizing agents upon freezing. A mixture of protonated and deuterated solvents, e.g., H_2_O, D_2_O, etc., and glass-forming compounds, e.g., glycerol, DMSO, etc., (added to avoid crystallization upon freezing) is usually used to codissolve the sample of interest and the chosen polarizing agents, e.g., TEMPO(L), trityl, etc., to optimal concentrations. The presence of a small amount of protons (typ. 10%) in a partially deuterated solvent is often beneficial in experiments tailored to exploiting CP methodologies. The sample is pipetted into a sample cup and inserted into the *d*DNP polarizer. The sample region is filled with liquid helium prior to sample injection, which establishes a homogeneous glassy sample upon contact of the *d*DNP mixture with the cryogenic liquid inside the polarizer. At this point, the molecule of interest and the paramagnetic agent are homogeneously embedded within the frozen glassy solid. Alternatively, liquid *d*DNP mixtures can be pipetted into a Dewar flask containing liquid nitrogen, and the frozen beads quickly placed in the sample cup and transferred to the polarizer before any melting occurs.

Dopants containing free electron spins in a *d*DNP sample deleteriously reduce the *T*_1n_ of liquid-state nuclear polarization after dissolution (see below). To combat such a problem, a series of porous materials were synthesized to spatially separate paramagnetic centers from the nuclear spins to be polarized in the solid state.^357^ Other sample formulation strategies that result in remarkably long values of *T*_1n_ utilize powders impregnated with DNP juice or transient radicals produced by UV irradiation of the sample. These strategies were employed in the transport of hyperpolarized metabolites^46^ and are suitable for producing radical-free solutions post dissolution. These approaches are discussed in more detail later in this section.

Once the *d*DNP-compatible sample is inserted in the polarizer and frozen, microwave irradiation is activated and, depending on the radical type, polarizing agent concentration, and choice of microwave frequency, the polarization of the nuclear spin isotope of interest accumulates at a particular rate. As a rule of thumb, a lower radical concentration gives a lower nuclear polarization build-up rate, and as a result, a paramagnetic radical concentration of 15–50 mM is used for *d*DNP experiments. This ensures that, for ^1^H nuclear spins at 3.8 K, the DNP build-up time constants are suitably short, for example, τ_DNP_ ≈ 40 s at 50 mM TEMPO(L) concentration, and that a sufficient nuclear polarization, e.g., *p*(^1^H) > 60%, is obtained within 5 × τ_DNP_. At higher radical concentrations, although faster polarization build-up rates can be achieved, this comes at the detriment of a reduced maximum polarization. For ^13^C nuclear spins, polarization build-up times are often much longer (on the order of hours) but the ^13^C polarizations achieved can be on the order of tens of percent. At lower temperatures, greater nuclear spin polarization levels are certainly achievable for all nuclear spin species but often with significantly longer polarization build-up times.

In a conventional *d*DNP setup, after a satisfactory nuclear polarization (e.g., *p*(^13^C) > 60%) is achieved, a superheated solvent (e.g., D_2_O at >150 °C) is directly injected into the sample cup through a “dissolution stick” under helium gas pressure (6–9 bar). The use of liquid-driven transfer of hyperpolarized samples has also recently been proposed.^358^ The frozen *d*DNP solution rapidly melts upon contact with the warm solvent, so long as the sample is raised above the liquid helium reservoir prior to the injection of the superheated solvent jet to prevent the dissolution medium from freezing in the liquid helium environment. Since the time taken to dissolve the *d*DNP sample (seconds) is typically much shorter than *T*_1n_, the relaxation caused by paramagnetic sources at elevated temperatures prior to dissolution is insignificant. However, the magnetic field at the point of dissolution should remain relatively high (several tesla is best) in order to maintain the hyperpolarization previously accrued in the solid state.

The RF probe (see above) must also act as a fluid pathway for the quick (in a fraction of a second) expulsion of the hyperpolarized sample from the *d*DNP polarizer. The newly liquid-state sample is flushed out of the *d*DNP polarizer under the pressure of helium gas and is propelled toward the point of use.^359^ Dissolving frozen samples polarized at liquid helium temperatures provides the greatest possible signal enhancements for solution-state NMR experiments.

Paramagnetic relaxation in the presence of free electron species in the liquid produced upon dissolution attenuates the lifetime of hyperpolarized magnetization throughout the sample transfer stage from the polarizer to the point of use. Relaxation due to paramagnetic agents is most rampant in regions of low magnetic field, which can be avoided by constructing a magnetic tunnel along the sample transfer route.^359^ A permanent magnet Halbach array with a sufficiently high magnetic field (hundreds of mT) is often used for this purpose. Paramagnetic relaxation after dissolution can be also countered by adding beads of frozen sodium ascorbate to the *d*DNP sample cup before hyperpolarization. Upon dissolution, the individual beads of sample and ascorbate are melted simultaneously, and the chemicals are intimately mixed by the hot jet of dissolution solvent. The paramagnetic centers are scavenged by the ascorbate molecules yielding ascorbyl radicals which rapidly disproportionate to diamagnetic species in the hyperpolarized solution.^360^

An approach alternative to conventional dissolution is to eject the frozen hyperpolarized sample as a solid “bullet”,^361^ with the key advantage of its scalability toward small (e.g., ≤80 μL) sample volumes suitable for high-resolution NMR while maintaining high concentrations of target molecules. In the practical implementation of this approach,^361^ the polarized frozen sample in a polytetrafluoroethylene (PTFE) cylinder is transferred over a distance of a few meters in as little as 70 ms using pressurized helium gas through a tunnel enclosed in a solenoid that provides a magnetic field of ∼75 mT. Upon arrival in the NMR magnet, the “bullet” is dissolved in a preheated solvent, and the hyperpolarized solution is drawn into a 5 mm NMR tube. A ^13^C polarization *p*(^13^C) > 30% was achieved for [1-^13^C]pyruvic acid. Further refinement of this approach resulted in the NMR line widths of the order of 3 Hz at similar levels of ^13^C polarization.^362^

#### 3.4.3 Applications

**Figure 32:**
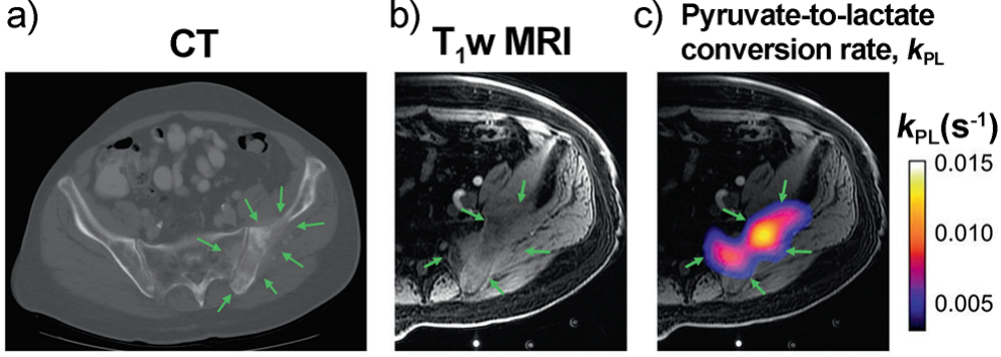
(a) A CT image of a patient with metastatic prostate cancer showing a relatively osteolytic lesion in left ilium (green arrows), which was infiltrative, causing destruction of the bone cortex and extension into the surrounding soft tissues. (b) T_1_-weighted (T_1_w) spoiled gradient-echo MR image of the same lesion. (c) The color-coded map of the pyruvate-to-lactate conversion rate (*k*_PL_) overlaid on the MR image, demonstrating the correlation of high *k*_PL_ values with the osseous lesion on CT and hypointensity on the T_1_w MR image. The value of *k*_PL_ was estimated as 0.013 s^–1^. Reproduced from ref (366). Copyright 2019 The Authors. Published by Springer Nature under CC BY license.

*d*DNP has a direct impact on in vivo research. The first successful study demonstrating the feasibility of hyperpolarized [1-^13^C]pyruvate as a noninvasive marker of tumor metabolism was completed in 2013.^363^ Hyperpolarized [1-^13^C]pyruvate was safely injected in patients with prostate cancer and real-time MRI experiments took advantage of the high intensity signals to characterize pyruvate–lactate conversion rates in localized tumors.^364^*d*DNP has also been employed to assess tumor grades^365−367^ and to continuously monitor cancer cells of living organisms in response to treatment.^363^ Today, hyperpolarized MRI is moving toward clinical applications, which is an important driving force for the whole field of *d*DNP research. A vast majority of the studies using *d*DNP are indeed focusing on such applications and are published at a high pace. As mentioned earlier, most studies focus on detecting and following prostate cancers^368,369^ and the potential metastasis to bone and liver^366^ (Figure 32). Other studies have also shown the great potential of *d*DNP for detecting breast cancer,^367^ or brain metabolism^370^ and glioblastoma.^371^ More recently, detection of renal tumors^372^ and even whole abdomen metabolism^373^ was reported.

A number of applications of *d*DNP related to NMR spectroscopy solely are also emerging, especially as the method is becoming increasingly robust and repeatable,^358,374^ getting closer to high resolution NMR spectroscopy standards.

**Figure 33:**
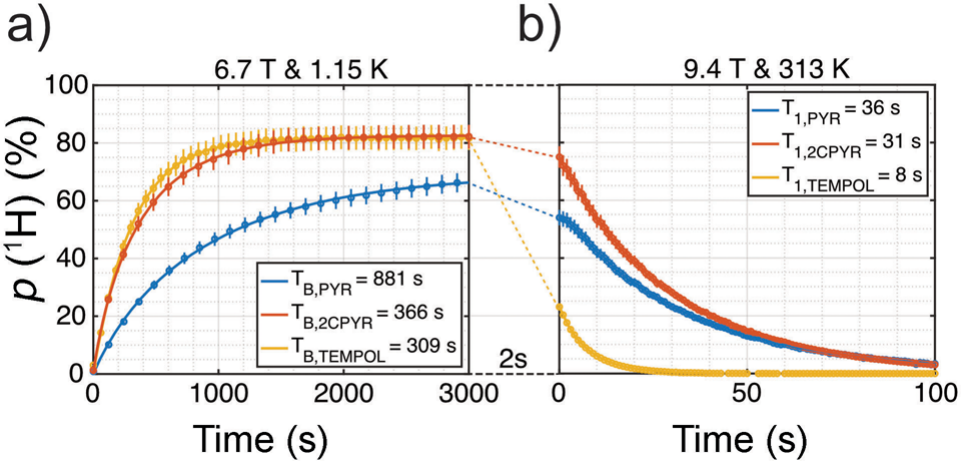
Hyperpolarization of water using DNP with radicals generated by UV light from pyruvic acid (PYR), [2-^13^C]pyruvic acid (2CPYR), and with a stable radical (TEMPO(L)) before (a) and after dissolution (b). Errors are given as the standard deviation of repeated measurements from distinct samples (*n* = 3). Adapted from ref (376). Copyright 2020 The Authors. Published by Springer Nature under CC BY license.

Hyperpolarized water has been gaining increased interest in NMR experiments as of late and could be an alternative to gadolinium-based contrast agents for MRI investigations.^375^ Recently, polarization levels of water after dissolution were reported to be in excess of 65% after using UV-generated radicals (see below) in *d*DNP experiments^376^ (Figure 33). As a result, a number of applications become possible, for example, the opportunity to rapidly acquire 2D NMR spectra which reveal the nature of ligand interactions with liposomes.^377^ Spontaneous transfer of magnetization from hyperpolarized water to natural abundance nitrogen sites in urea was also demonstrated and impressively observed with only a single ^15^N NMR signal acquisition, which may open a new route for hyperpolarization of insensitive nuclear spins.

Since *d*DNP is somewhat restricted to smaller molecular sizes which ensure *T*_1n_ times of more than a few seconds necessary for sample dissolution and transfer, most notably metabolites (including their ionic forms) have been thoroughly investigated for biological applications.^378^ In addition to the aforementioned pyruvate and water, a number of other small molecules have been screened for drug-binding studies, alongside the study of cell extracts and heteronuclear metabolomics.^374,379,380^ A number of nonenzymatic reactions have also been investigated with *d*DNP experiments, including catalytic polymerization of dissolved hyperpolarized gases,^381^ ring-closing metathesis,^382^ and the rate determination of a Diels–Alder reaction.^383^ At the same time, the high levels of nuclear polarization achieved with *d*DNP have allowed development of applications focused on larger molecular sizes, such as ^1^H-^15^N 2D NMR experiments for the study of osteopontin,^384^ probing RNA refolding,^385^ and structural elucidation at natural isotopic abundance.^386^

The concepts of *d*DNP and long-lived spin states (LLSSs)^37^ were conceived at roughly the same time, with the first publications appearing in 2003^387^ and 2004,^342^ respectively. However, the potential to have hyperpolarized substances with long storage lifetimes was not realized until the two approaches were first combined in 2009.^49^ Since then, a number of LLSSs were produced with *d*DNP.

*d*DNP was put to use for small molecules with hindered-rotor methyl groups at low temperatures, where the LLSSs are populated as a result of the strong Zeeman polarization afforded by *d*DNP,^388^ similar to the case of the chemically equivalent proton spin pair of the CH_2_ group of ethanol.^389^ Overpopulation of LLSSs for chemically inequivalent nuclear spin pairs was also achieved by their direct overpopulation in the frozen solid sample for the case of [1,2-^13^C_2_]pyruvic acid.^390^ Disappointingly, the large isotropic chemical shift difference between the ^13^C-labeled nuclear sites engendered their rapid relaxation, and no distinct advantages could be demonstrated compared to ordinary Zeeman polarization. The monodeuterated methyl group of N-CH_2_D-2-methylpiperidine displays a much smaller ^1^H chemical shift difference, and as a result, hyperpolarized long-lived NMR signals were observed more than one minute after dissolution.^391^ A more impressive result, with hyperpolarized NMR signals persisting for ca. 30 min, was demonstrated for ^13^C spin pairs by the ingenious combination of magnetic field cycling and specific RF pulse sequences.^41^

**Figure 34:**
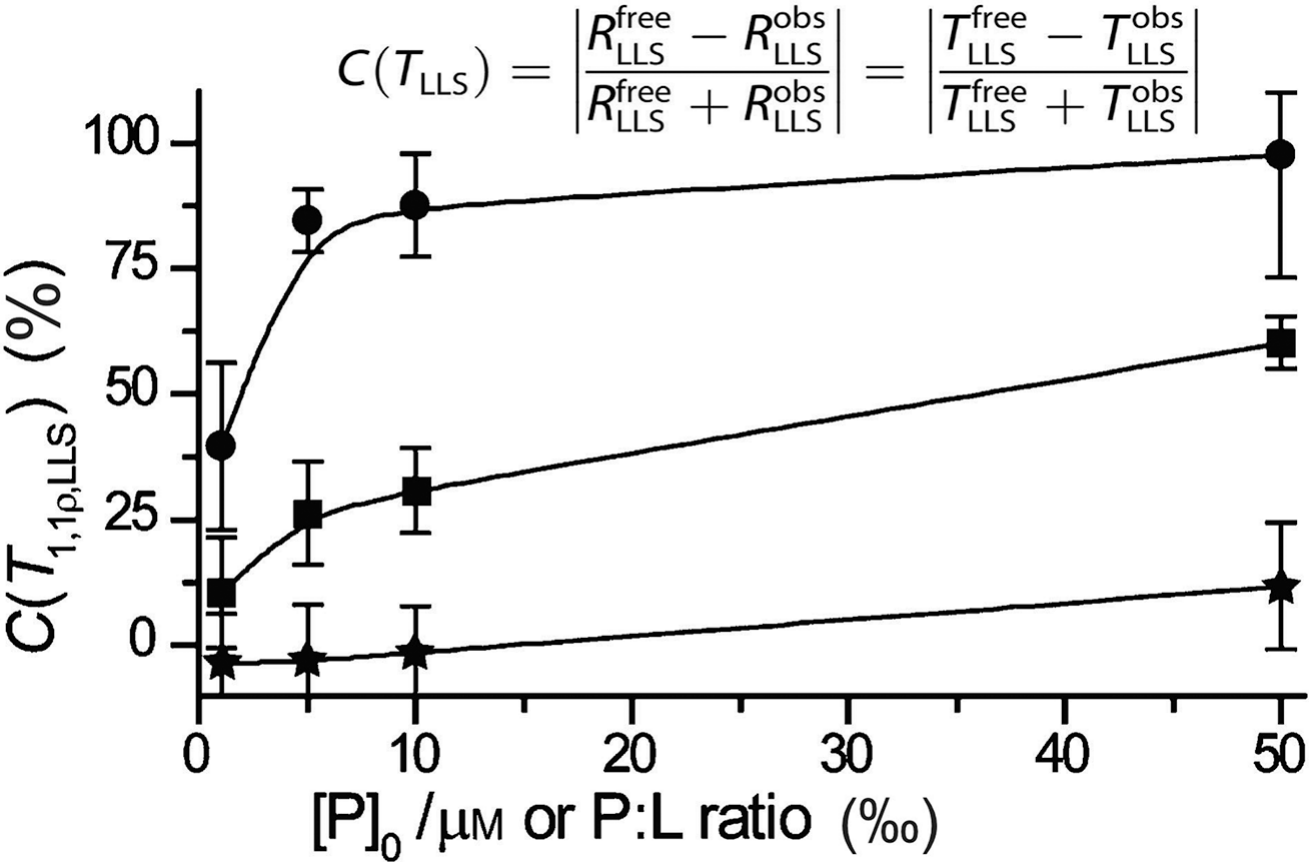
Experimental contrast *C* in *T*_1_ (star), *T*_1ρ_ (square) and *T*_LLSS_ (circle) relaxation time constants for the chemically inequivalent ^1^H spin pair in the central residue of the polypeptide Gly-Gly-Arg at 1 mM concentration in D_2_O solution at 500 MHz (11.7 T) and 8 °C as a function of protein–ligand ratio (P:L) (fixed concentration of ligand [L] = 1 mM and a variable trypsin concentration 0.5 μM < [P]_0_ < 50 μM). Reproduced with permission from ref (394). Copyright 2014 The Authors. Published by Wiley-VCH Verlag GmbH & Co. KGaA.

Biologically relevant enzymatic conversion of a chemical compound, such as fumarate into malate, was probed via *d*DNP.^392^ In this case, the magnetically equivalent fumarate proton spins were trapped in an LLSS, and the silent LLSS was then revealed as intense NMR signals via a chemical reaction. In another example, LLSSs were used to accurately determine the dissociation constants of weakly binding ligands to the protein Hsp90.^393^ The long lifetimes of the LLSSs permitted characterization of ligands with an extremely wide range of affinities in competitive binding studies. Since LLSS decay time scales are more sensitive to ligand binding than the *T*_1n_, *T*_1ρ_, and *T*_2n_ relaxation time constants (Figure 34), the scope of ligand screening can easily be extended to arbitrary ligands by employing covalent attachment of functional groups exhibiting LLSSs that can be readily overpopulated.^394^ These weakly binding spy ligands are displaced by high-affinity competitors, with the approach enabling the use of substantially decreased protein and ligand concentrations and significantly increased sample throughput.

The unusual combination of *d*DNP with zero- and ultralow-field (ZULF) NMR has recently shown promising results.^395,396^ ZULF NMR is a modality of magnetic resonance which does not require strong magnetic fields.^64^ Sufficient nuclear spin polarization is often achieved by adopting hyperpolarization techniques. ZULF NMR spectra of a handful of metabolites were enhanced by factors of ca. 10^4^ with *d*DNP employed as the hyperpolarization method.

#### 3.4.4 Frontiers and Challenges

**Figure 35:**
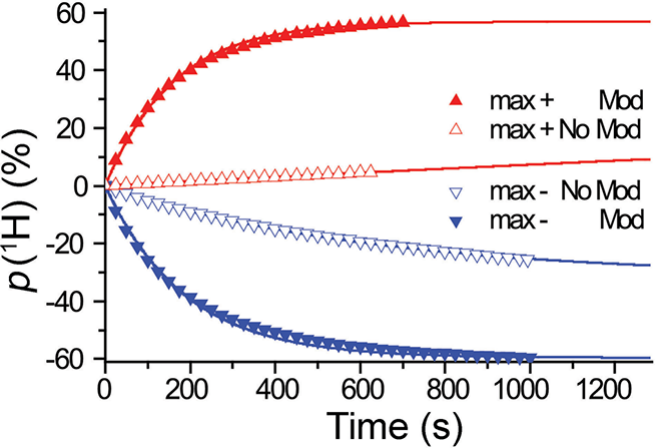
Positive and negative ^1^H DNP build-up curves measured at *T* = 1.2 K and *B*_0_ = 6.7 T, with and without frequency modulation, for a sample comprising a 10:40:50 (v/v/v) H_2_O:D_2_O:glycerol-*d*_8_ mixture with 25 mM of TEMPOL. The optimal microwave frequencies were set for positive or negative DNP (187.85 or 188.3 GHz, respectively), with a microwave power of 87.5 mW. The amplitude of the frequency modulation was set to 100 MHz with a modulation frequency of 10 kHz. An amplitude of 100 MHz was used for frequency modulation. Reprinted from ref (397). Copyright 2014 The Authors. Published by Elsevier B.V.

Numerous refinements of the *d*DNP technique were implemented over the years. Adjustment of the microwave frequency at the source is desirable in order to pursue hyperpolarization of different nuclear spins with a range of radical sources. Additionally, the capability to perform frequency modulation considerably boosts both the polarization levels and build-up rates at reduced concentrations of free radicals^397^ (Figure 35).

**Figure 36:**
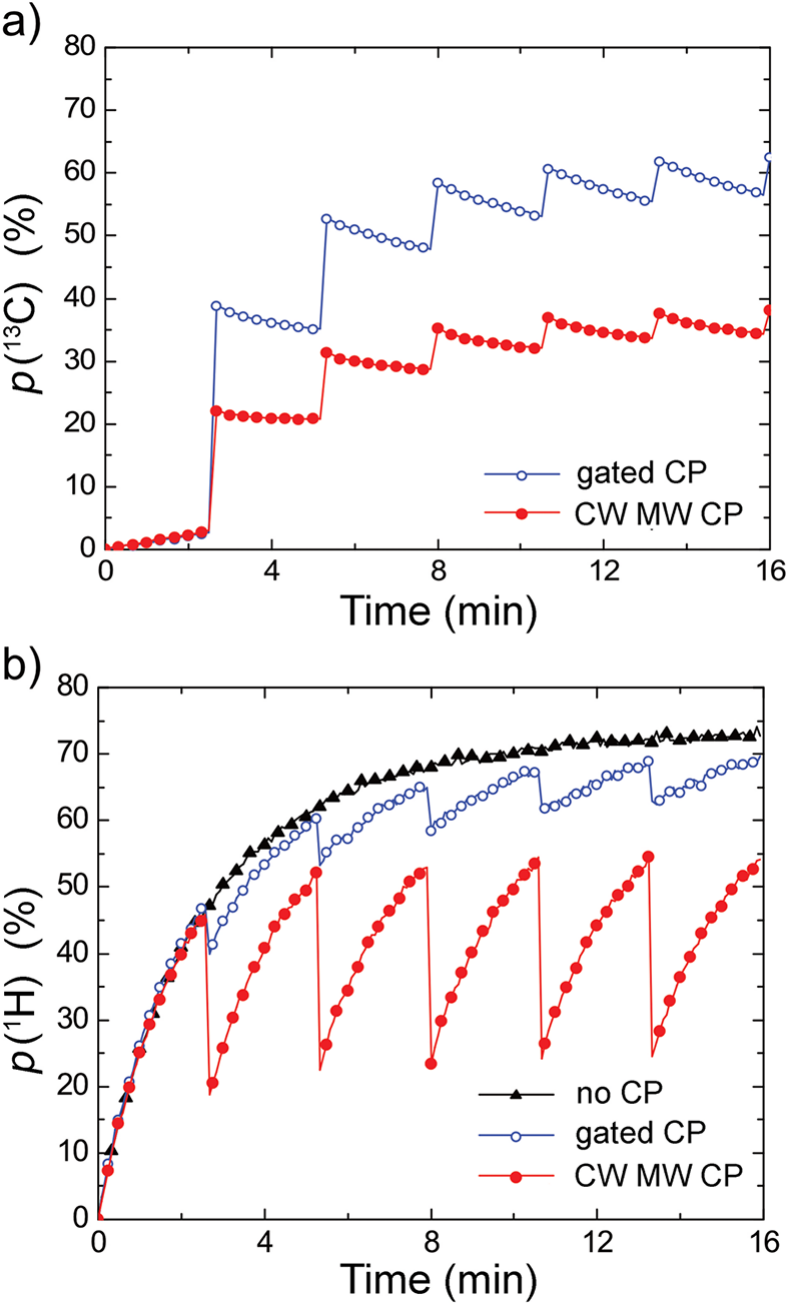
(a) Build-up of *p*(^13^C) polarization during multiple CP contacts applied every 2.5 min with continuous (red) or gated (blue) microwave irradiation at 188.3 GHz (50 MHz frequency modulation amplitude, 10 kHz modulation frequency, 87.5 mW microwave power) for a 3 M sodium [1-^13^C]acetate with 40 mM TEMPOL at 1.2 K and 6.7 T. (b) DNP build-up of proton polarization *p*(^1^H) for the same sample and conditions without any CP (black), or during multiple CP applied every 2.5 min with continuous (red) or gated microwave irradiation (blue). All lines are drawn to guide the eye. Reproduced from ref (335) with permission from the Royal Society of Chemistry.

The construction of *d*DNP RF probes with multiple RF-channel pulse and receive capabilities introduces the prospect of performing more sophisticated NMR experiments under *d*DNP conditions. One such advent which has greatly contributed to the output of *d*DNP experiments is the implementation of CP and adiabatic demagnetization RF pulse sequences.^398^ The introduction of CP has forged a route to impressively high levels of both ^13^C (*p*_hyp_ ≈ 60%) and ^15^N (*p*_hyp_ ≈ 25%) polarization, which can be achieved in a mere few tens of minutes^335,352^ (Figure 36).

**Figure 37:**
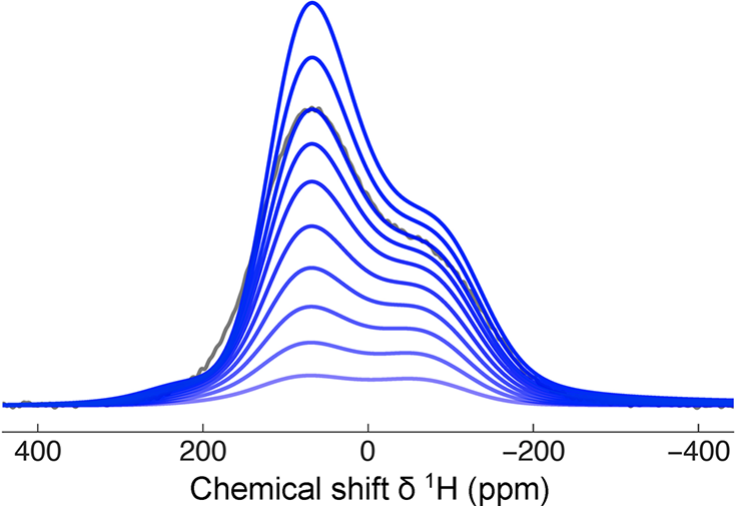
Relevant portion of the simulated (blue) and experimental (black) ^1^H echo-detected NMR spectra of water frozen at 1.2 K acquired at 7.05 T as a function of ^1^H polarization *p*(^1^H). The simulated and experimental ^1^H echo-detected NMR spectra are in agreement for the case of ^1^H polarization *p*(^1^H) = 75%. Adapted from ref (402) with permission from the Royal Society of Chemistry.

Commonly, liquid-state polarization levels are measured by comparing the hyperpolarized NMR spectrum with the thermal equilibrium NMR spectrum of the same sample, or a reference sample of known concentration, taking into account differences in acquisition parameters, e.g., RF-pulse flip angle, number of transients, receiver gain, etc. An alternative strategy, known as spin polarimetry magnetic resonance (SPY-MR), which does not rely on time-consuming acquisition of thermal equilibrium NMR signals, allows for an instant quantification of polarization since the detection of additional NMR signals is not required.^399^ For a scalar-coupled heteronuclear system of two spin-1/2 nuclei, the liquid-state thermal equilibrium NMR spectrum of either nucleus yields a doublet with NMR lines of approximately equal intensity, since the polarization of both spin types is small. Now consider the case where one spin (*i*) is hyperpolarized. The polarization of the other spin (*j*) should be sufficiently high as to allow single-shot detection of its NMR signal after sample dissolution. The polarization of spin *i* can be inferred from the NMR spectrum of spin *j*, since, in this case, the doublet line shape is asymmetric. The value of the polarization of spin *i* is directly read out from the NMR spectrum of spin *j* by using the following equation:
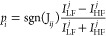
6which assumes that both nuclear species have gyromagnetic ratios of the same sign. J_ij_ is the heteronuclear scalar coupling constant, and I_LF_^*j*^ and I_HF_^*j*^ are the low-field (LF) and high-field (HF) resonances of the NMR spectrum of spin *j*, respectively. Similarly, in the solid-state prior to dissolution, indirect line shape polarimetry approaches have also recently been proposed.^400^ One important example is that of H_2_O and D_2_O. Low spin temperatures, violating the high-temperature approximation for the nuclear Zeeman interaction, lead to characteristic asymmetries in NMR powder spectra. DNP has been employed to enhance the population of the ground nuclear spin state of water molecules, which produce asymmetric ^1^H NMR spectra.^401^ This approach was recently demonstrated for ^1^H echo-detected NMR spectra of H_2_O;^402^ it avoids time-consuming comparisons with low intensity thermal equilibrium ^1^H NMR spectra acquired without microwave irradiation. After ∼34 min of DNP at 7.05 T and ∼1.2 K, a ^1^H polarization *p*(^1^H) = 75% was found by comparing the experimental spectrum with line shape simulations (Figure 37). Such an asymmetry is also present in the ^2^H echo-detected NMR line shape of highly polarized deuterium spins in D_2_O.^403^

Another notable case is that of ^15^N_2_O. This nontoxic anesthetic gas exhibits remarkably long hyperpolarization storage lifetimes in low magnetic fields,^404^ and is therefore of great interest for the imaging modality MRI. The ^15^N NMR line shape of ^15^N_2_O in a solid-state matrix (frozen mixture doped with trityl radical in an organic solvent) hyperpolarized by *d*DNP is additionally highly asymmetric.^405^

**Figure 38:**
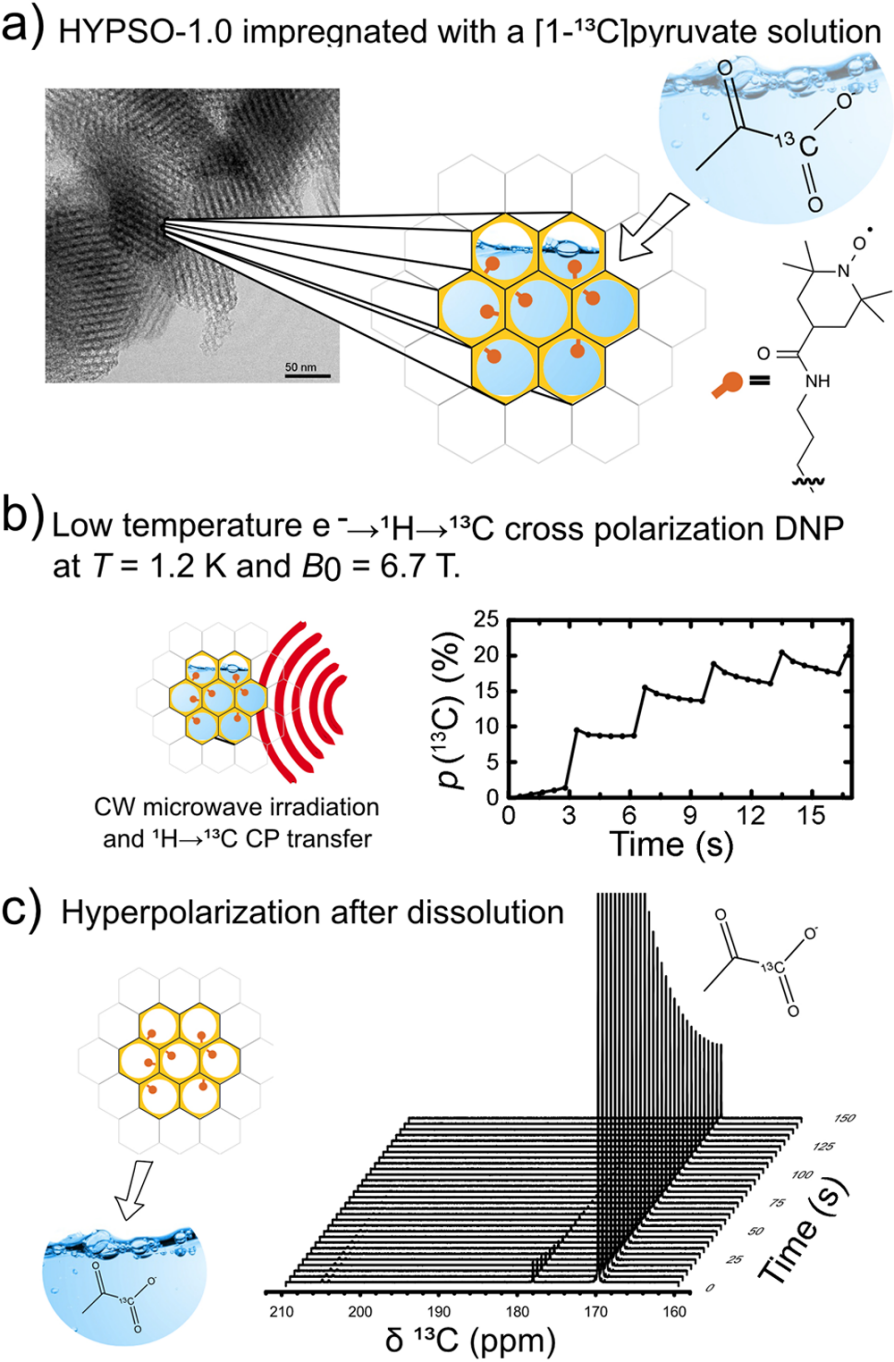
Hyperpolarization by *d*DNP with HYPSO. (a) HYPSO 1.0 is impregnated with a solution of the analyte to be polarized without addition of glass-forming agents. The transmission electron microscopy (TEM) image shows the porous structure of the material. Red dots schematically represent the polarizing agent. (b) ^1^H DNP performed on 20 mg of HYPSO 1.0 material (88 μmol/g) impregnated with 36 μL of a 3 M solution of [1-^13^C]pyruvate in D_2_O. The ^1^H polarization builds up with a time constant τ_DNP_(^1^H)=119 ± 1.5 s, and by applying ^1^H to ^13^C CP, a polarization of *p*(^13^C) > 20% is reached in 17 min. (c) The sample is dissolved and expelled from HYPSO 1.0 by injecting 5 mL of superheated D_2_O and is transferred to a 300 MHz NMR spectrometer. A series of ^13^C NMR spectra of [1-^13^C]pyruvate are acquired (one spectrum is collected every 5 s). The liquid-state polarization obtained, *p*(^13^C) = 25.3%, corresponds to an enhancement of ε > 32,000 compared with Boltzmann equilibrium at 300 K and 7 T. The polarization decays with *T*_1_(^13^C) = 49.4 ± 0.4 s, which is typical for a pure D_2_O solution of [1-^13^C]pyruvate without any free radicals. Adapted with permission from ref (408).

Early in the 1980s, research groups started developing sample formulations such as solid matrices grafted with nitroxide radicals^406^ which were later used^407^ in the context of OE-DNP for flowing solutions in and out of the DNP volume. In 2013, this concept of polarizing matrices was implemented in the contexts of MAS DNP and *d*DNP for the first time. These hybrid polarizing solids (HYPSO) were successfully used for *d*DNP^408^ thus yielding pure hyperpolarized solutions while the matrices containing paramagnetic free radicals could be filtered out inline during the dissolution process (Figure 38). HYPSO matrices are compatible with a number of molecules,^408,409^ including those of biological interest, and remove the requirement of adding glassing agents to produce *d*DNP-compatible samples. The DNP performance of such matrices was subsequently improved as a result of further development based on different pore geometries and radical localization (such as within the pore walls of the material itself rather than on its surface).^357,410,411^ In parallel to the development of silica matrices, polymer matrices were implemented for *d*DNP,^412^ first as thermoresponsive hydrogels, and then as thermoresponsive thermoplastics.^384^ The great advantage of such approaches is that these polymers tend to naturally precipitate during the dissolution process and are thus easily filterable.

**Figure 39:**
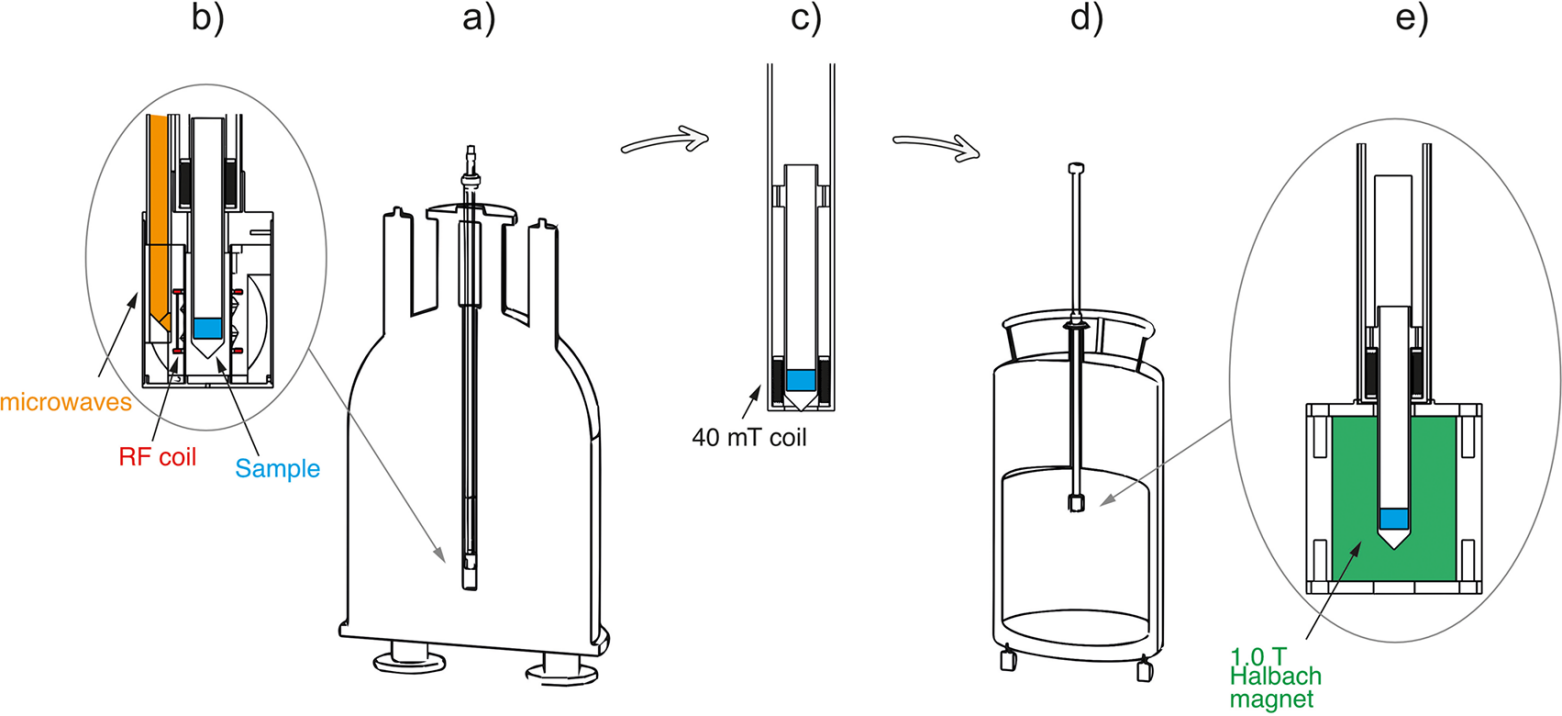
Impregnating radical-containing porous matrices with molecules of interest and storing such materials at moderate magnetic fields allows transportation of hyperpolarized media using permanent magnet systems. (a) 6.7 T wide bore magnet and 1.2 K cryostat (polarizer); (b) *d*DNP probe; (c) transfer stick; (d) liquid helium transport dewar; (e) magnetic insert. Reproduced from ref (46). Copyright 2017 The Authors. Published by Springer Nature under CC BY license.

More recently, the concept of hyperpolarizing polymers (HYPOPs) with increased pore sizes (typ. pores >100 nm in diameter) was used to generate hyperpolarization that can survive for hours.^413^ In this approach, free radicals are covalently attached within the bulk (wall) of a porous polymer matrix that can be impregnated with an arbitrary solution of molecules. Pores (typ. 0.1–1 μm in diameter) are tailored in such a way that hyperpolarization can be built up rapidly (within minutes) on ^1^H nuclear spins and then transferred via conventional CP protocols to ^13^C nuclear spins, subsequently lasting for hours or days at cryogenic temperatures. Recently, levels of polarization exceeding *p*(^13^C) > 25% were reported using this approach in HYPOP matrices. The polarization was generated within tens of minutes, while polarization lifetimes exceeded five hours, providing the possibility of transporting the hyperpolarized sample to the point of use.^413,414^ Furthermore, hyperpolarization of ^13^C nuclear spins in isotopically labeled metabolites alanine and glycine was shown to survive a 16-h storage period in a moderate magnetic field outside of a polarizer (Figure 39).^46^ The use of an impregnated micropowder with radical sources hidden away from the nuclear spins of interest was key to extending storage lifetimes and allowed an overall ^13^C polarization enhancement of up to 3 orders of magnitude to be observed almost a day later. Being able to transport hyperpolarization for such long periods would remove the requirement of the polarization process being carried out close to the point of use.

An alternative strategy is based on the use of photoinduced radical species that can be transiently generated by UV irradiation and are subsequently annihilated thermally at temperatures below the *d*DNP sample melting point. This enables removal of hyperpolarized ^13^C-bearing substances from the polarizer in the solid state, since the radicals are annihilated and no longer significantly contribute to nuclear spin–lattice relaxation, while simultaneously maintaining augmented ^13^C polarization levels; this has applications to the storage and transport of hyperpolarized products. The first *d*DNP experiment using UV-induced radicals dates back to 2013 and was conducted with neat pyruvic acid.^84^ Later on, it was shown that by warming up the *d*DNP sample without melting it, radicals could be annealed while preserving a large fraction of the hyperpolarization in a radical-free environment, therefore enhancing the hyperpolarization lifetime by orders of magnitude. Melting the solid-state sample many minutes after its extraction from the polarizer revealed liquid-state ^13^C polarizations for pyruvic acid of ∼8%.^414^

### 3.5 Chemically Induced Dynamic Electron Polarization

Chemically induced dynamic electron polarization (CIDEP) is a well-known phenomenon that leads to perturbation of the populations of electron spin energy sublevels in paramagnetic species (section 2.2.2). In most cases, spin-polarized radicals, triplet molecules or other paramagnetic species generated as a result of CIDEP in the presence of a magnetic field can be detected by EPR spectroscopy. To date, the CIDEP effect has been used for a variety of purposes, including mechanistic investigations of organic reactions^415,416^ and analysis of macromolecular solvent exposure and dynamics.^417,418^ Further, in the context of this review, CIDEP effects are important because, as stated in section 2.2.2, nonequilibrium electron spin populations can also serve as a useful source of nuclear spin hyperpolarization. Indeed, polarization of electron spins generated via CIDEP can lead to sensitivity enhancements in NMR spectroscopy.^90^ Examples of the use of polarized free radicals and triplet electronic states of molecules for signal enhancement in NMR can be found in sections 3.2 and 3.9, respectively. While CIDEP has been observed in both solids and liquids, this overview focuses on applications to the liquid state, with only brief excursions to solids.

Several CIDEP mechanisms are known to date. Most of them involve transient or stable doublet-state radicals (i.e., radicals bearing a single unpaired electron) or nonequilibrium singlet or triplet molecules, biradicals, or radical pairs.^416,419^

**Figure 40:**
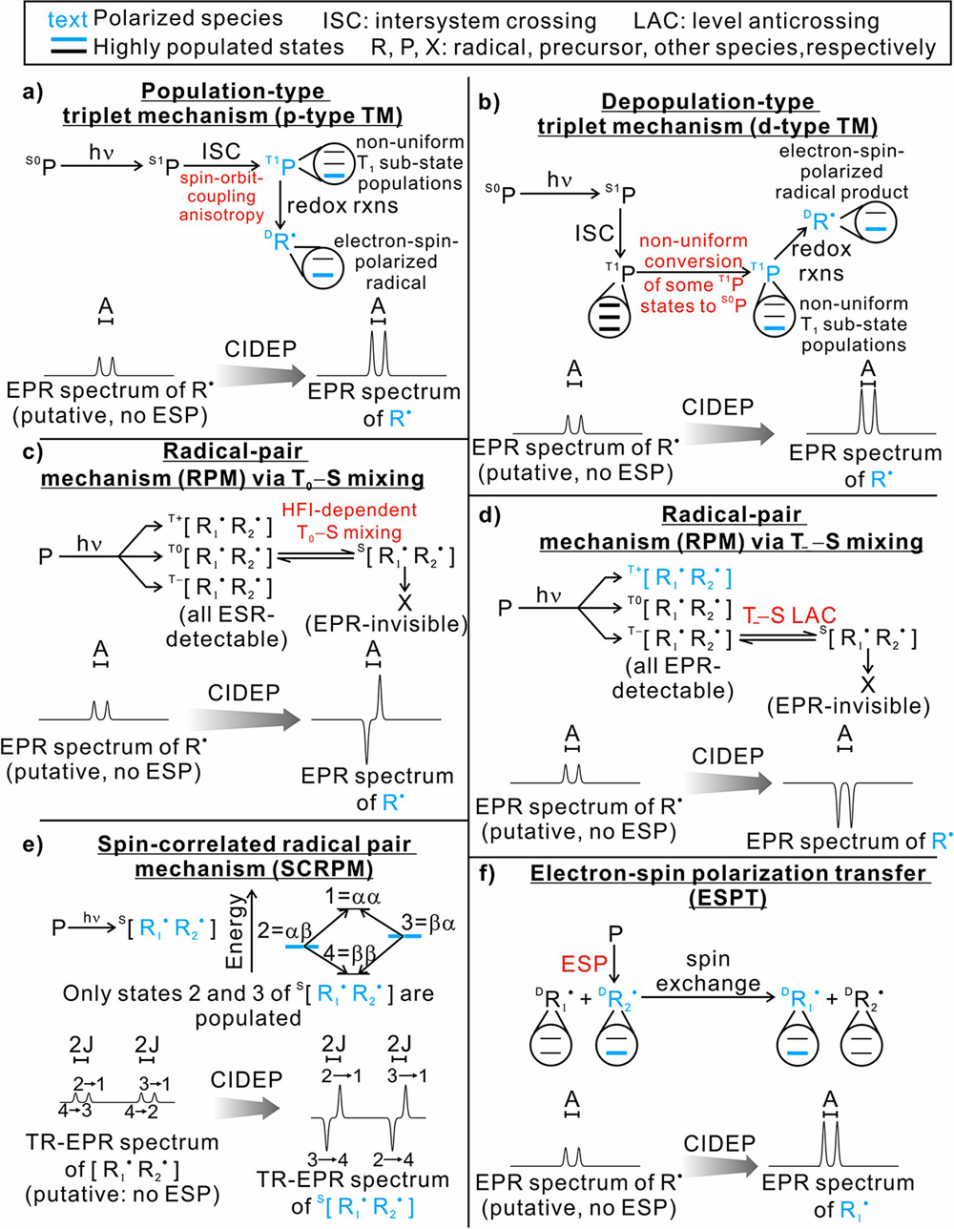
Overview of major CIDEP mechanisms. Legend: R - doublet radicals; P - precursor; ESP - electron spin polarization; X - other species; RX - covalently bound RX pair. [R^•^ X^•^] - spin-correlated R..X radical pair; S_0_ - species populating the ground-state singlet; S_1_ - species populating an excited-state singlet; T_1_ - species populating the T_1_ excited triplet state; D, T, Q: doublet, triplet, quartet electron spin states, respectively; T_+_, T_0_, T_–_ - substates of triplet electron spin states in the laboratory frame; ZFS - zero-field splitting; HFI - hyperfine interaction; LAC - level anticrossing; A - electron–nucleus hyperfine coupling; J - exchange coupling of electron spins in a radical pair or a biradical.

The major proposed mechanisms for the CIDEP effect are summarized next. We first consider the triplet mechanism^416,420^ (TM) of CIDEP, which can be further categorized into population-type (p-type TM, Figure 40a) or depopulation-type (d-type TM, Figure 40b). The CIDEP phenomena that proceed via both the p- and d-type TM involve a ground-state precursor molecule (^S0^P) that is eventually converted into a triplet-state intermediate (^T1^P). This conversion often takes place upon photoexcitation of ^S0^P within the manifold of molecular singlet states ^Sn^P (see section 3.9). In the case of p-type TM (Figure 40a), anisotropic spin–orbit coupling in P leads to spin-selective intersystem crossing (ISC) from the S_n_ to the T_n_ manifold, resulting in largely unequal populations of the three triplet sublevels in ^T1^P.^420,421^ In contrast, in the case of d-type processes (Figure 40b), nonequilibrium populations of the three triplet sublevels of ^T1^P are generated due to their unequal decay rates. Both processes can yield a strong hyperpolarization of electron spins of ^T1^P, which is observable as a major signal enhancement in the EPR spectra of molecular triplets in glassy solids (see below and also section 3.9) and in solution.^422^ For chemically reactive triplet molecules, the ^T1^P state can then yield doublet radicals (^D^R^•^) each possessing a single unpaired electron (or other paramagnetic species, e.g., ion-radicals or biradicals). These radicals can inherit strong hyperpolarization of electron spins from their triplet precursors, thus providing a major signal enhancement in their EPR spectra. Thus, a key feature shared by the p- and d-type TM processes is that they both go through a triplet intermediate bearing nonequilibrium electron spin sublevel populations. The two mechanisms ultimately give rise to similar spectral features in the EPR spectra of doublet radicals, with all resonances of each species bearing the same phase and displaying uniform initial enhancement.

Another common CIDEP mechanism proceeds via radical pairs (RPs) with a certain overall electron spin multiplicity (a triplet or a singlet),^415,423,424^ either in a photoinduced or sometimes in a thermal chemical reaction, and is commonly referred to as the radical pair mechanism (RPM). This CIDEP mechanism can be further divided into two subcategories, depending on whether the triplet-singlet mixing happens between S and T_0_ (Figure 40c) or S and T_–_ (via level anticrossing, Figure 40d) electron spin states of a radical pair or a biradical. In typical X-band EPR experiments, the dominant transition is between T_0_ and S states (Figure 40c). This transition probability depends on the g-factors, hyperfine coupling constants and nuclear spin configurations of both radicals. In the end, variable degrees of polarization levels (both positive and negative) are generated for resonances corresponding to different nuclear spin configurations. As a result of this hyperpolarization, the EPR spectrum shows absorptive and emissive enhancements for distinct spectral lines, e.g., an A/E (absorption/emission for the low/high field EPR signal component) or E/A pattern. On the other hand, if the hyperfine interaction is strong and translational diffusion is slow, mixing between the T_–_ and S states via LACs (level anticrossings) may become dominant (Figure 40d), leading to emissive enhancements.^415^ This mechanism is expected to be particularly efficient when the exchange interaction J of the two radical centers averaged over the T-S mixing time is sufficiently large to bring the T_–_ (in the usual case of J < 0) and S energy levels closer in energy (see section 3.6). A typical example is provided by transient biradicals with sufficiently short linkers between the two radical centers.^425^

Alternatively, CIDEP can be generated via the spin-correlated radical pair mechanism^426−428^ (SCRPM), whose major features are illustrated in Figure 40e. This mechanism involves chemical generation of an RP with the two electron spins correlated in such a way that only some of the possible electron spin states of the newly generated RPs are initially populated. This phenomenon leads to nonequilibrium intensities of EPR transitions associated with those states. In the example shown in Figure 40e, the spin-correlated radical pair (SCRP) originating from a singlet precursor exclusively populates the |αβ > and |βα > electron spin states. This scenario leads to two antiphase CIDEP doublets, upon detection via time-resolved EPR (TR-EPR). Importantly, to observe the SCRPM CIDEP effect in an EPR spectrum, there must be a weak but nonzero exchange coupling J between the two unpaired electrons (or dipolar coupling if molecular motion is slow enough). If this coupling is too strong, the exchange interaction can no longer be treated as a perturbation to the Zeeman Hamiltonian and a more advanced treatment of the phenomenon becomes necessary.^416^ Conversely, if J = 0, the intensity of the spectral lines with opposite phases (within each antiphase doublet) cancels out, leading to no net EPR signal. In practice, SCRPM CIDEP in liquids usually requires restricted translational diffusion of the radicals composing the RP. In this way, a significant exchange coupling persists over the course of the RP lifetime. Common approaches to achieve slow and/or restricted translational diffusion include embedding the spin-correlated RP into micelles, using highly viscous solvents, employing biradicals belonging to a rigid structural framework, or focusing on structurally constrained photosynthetic systems.^416,429^ Alternatively, CIDEP proceeding via SCRPM can be generated in the solid state.^426,430^ In contrast to RPM CIDEP, which requires some time for the S and T electronic states of an RP to mix, SCRPM CIDEP is generated as soon as the SCRP is produced.

In addition to direct polarization of multiple paramagnetic species as a result of CIDEP, the initially polarized species can then transfer their spin polarization to other paramagnetic species (Figure 40f), thereby propagating hyperpolarization throughout the sample. This process is known as electron spin polarization transfer (ESPT).^416^ A typical CIDEP proceeding via the ESPT mechanism is illustrated in Figure 40f. ESPT is possible upon both reactive and nonreactive collisions. Polarization can be transferred from a hyperpolarized triplet molecule to a doublet radical, or between two different stable radicals when one of them is polarized via an appropriate CIDEP mechanism, mediated by spin exchange. Polarization transfer via ESPT was also observed upon chemical reactions involving various radicals, biradicals, and diamagnetic and/or paramagnetic molecules.^416^

**Figure 41:**
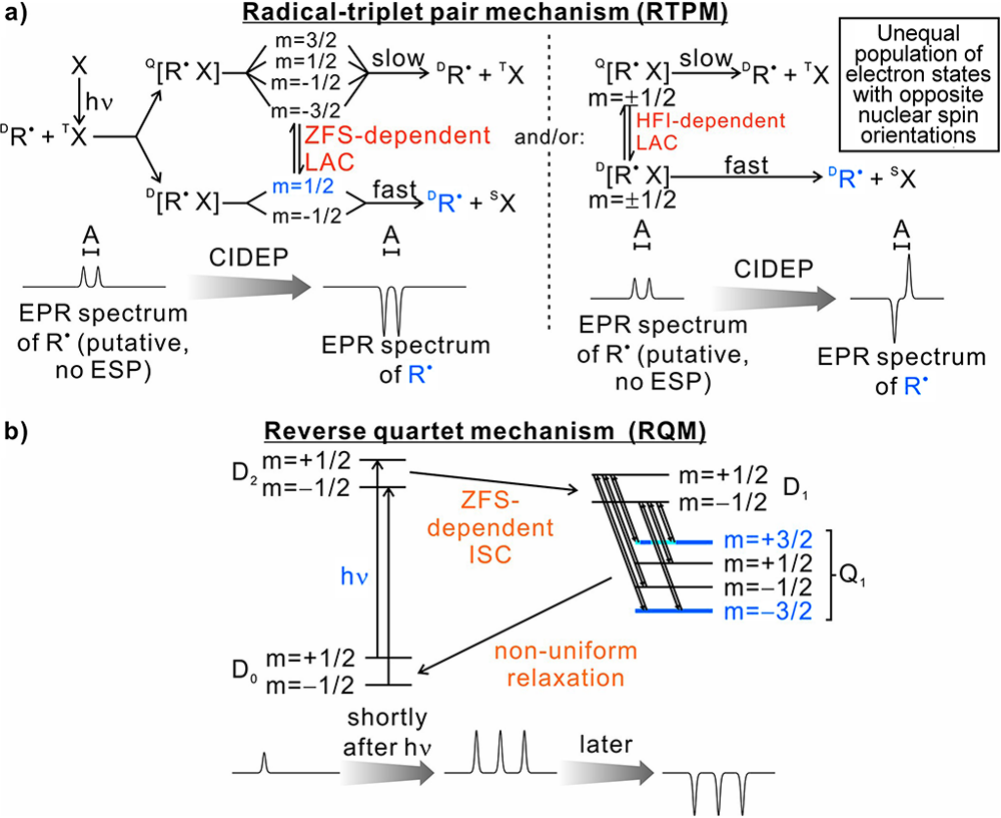
Overview of (a) radical-triplet pair mechanism (RTPM) and (b) reverse quartet mechanism (RQM) of CIDEP. Legend: ZFS - zero-field splitting; HFI - hyperfine interaction; LAC - level anticrossing; ISC - intersystem crossing.

Another mechanism involving molecular triplet states is the radical-triplet pair mechanism (RTPM, Figure 41a), which was initially proposed^431^ in 1989 and further explored later.^432−434^ CIDEP proceeding via RTPM requires a stable doublet radical (^D^R^•^) and, most typically, a chromophore photoexcited to a triplet electronic state (^T^X). The two components can be either free in solution, or tethered to each other via a flexible covalent link. Upon collision between ^D^R^•^ and ^T^X, two different types of radical-triplet pairs are produced; they are shown enclosed in square brackets in Figure 41a. Namely, the radical–triplet pair can either be in a quartet or in a doublet electron spin state, denoted as ^Q^[R^•^..X] or ^D^[R^•^..X], respectively. According to conservation of the total spin angular momentum, only the doublet pair can convert into ^D^R^•^ and ^S^X, where the triplet state of the chromophore has been converted into its singlet state. Electron spin hyperpolarization is generated because of either zero-field-splitting-dependent (ZFS-dependent) or hyperfine-interaction-dependent (HFI-dependent) LACs. These LACs enable transitions between specific quartet substates and the doublet state of the triplet radical pair. As a result, electron spin hyperpolarization is generated and the respective TR-EPR spectra bear the features illustrated in Figure 41a. In general, the observed spectral pattern is the combination of net emissive and E/A type polarization (denoted as E*/A in the literature).^434^ As a rule, the ZFS-dependent route leads to a net emissive polarization while the HFI-dependent path gives rise to E/A-type polarization.

Remarkably, the RTPM mechanism results in hyperpolarization of stable free radicals that are already present in solution before optical irradiation. In contrast, the other mechanisms usually require in situ generation of radicals. Moreover, RTPM-governed processes do not require any chemical reaction. This feature enables unique applications specifically targeting EPR or NMR sensitivity enhancement, as discussed below.

Yet another sequence of events, known as the reverse quartet mechanism^435,436^ (RQM), was proposed for CIDEP. This mechanism has some features in common with RTPM and usually requires a pair consisting of a stable radical and a chromophore that can be photoexcited to its triplet electronic state. Unlike RPTM, however, the two components must be rigidly bound to each other to prevent relative motions between the triplet chromophore and the doublet radical (see D_0_ in Figure 41b). Provided that the exchange interaction J between the triplet chromophore and the radical is much larger than the difference in their Zeeman frequencies, the radical-triplet pair must be treated as a doublet or quartet spin system. TR-EPR spectroscopy performed on RQM-polarized samples displays some characteristic features. Namely, the spectrum is first enhanced in one direction (for example, corresponding to net emission) but later in time, as data collection continues, the spectrum undergoes inversion (for example, to net absorption), as shown in Figure 41b. Shortly (ca. 1 μs) after a laser pulse, the quartet excited state Q_1_ is populated and is characterized by a nonuniform population of electron spin sublevels, resulting in the initial electron spin polarization. Later, due to nonuniform electron spin–lattice relaxation, the electron spin polarization reverses. The absorptive vs emissive nature of the hyperpolarization was shown to be governed by the sign of exchange interaction J.^437^ A detailed description of this mechanism can be found elsewhere.^436^

**Figure 42:**
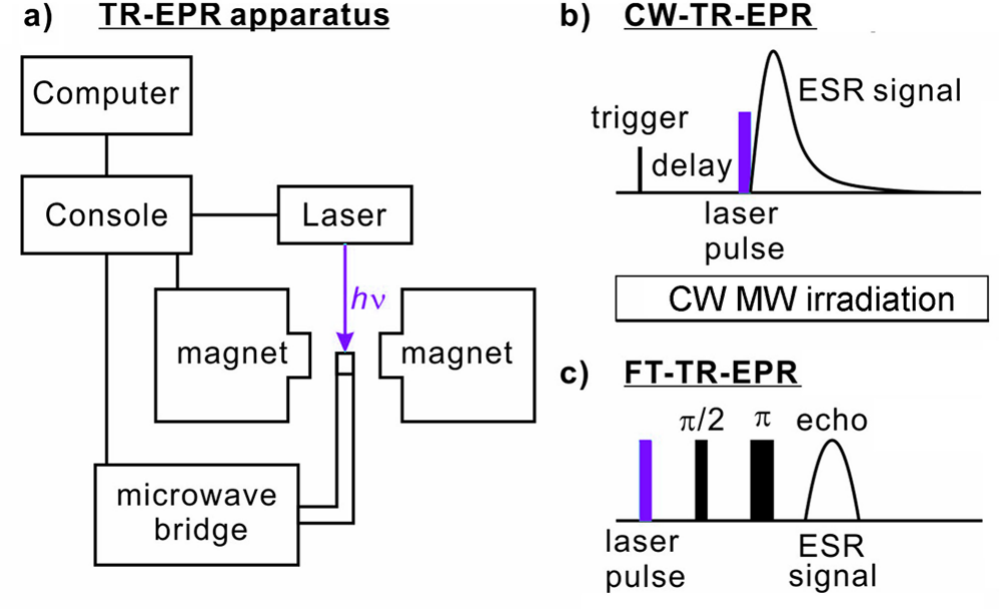
(a) Schematic illustration of experimental apparatus employed to monitor CIDEP effects by TR-EPR. A short laser pulse generates transient paramagnetic species for EPR detection. The time between laser irradiation and data collection is then tracked. (b) Data acquisition scheme for a typical CW-TR-EPR experiment. The MW irradiation is continuous and the EPR signal is recorded upon comparing MW absorption before and after laser irradiation. (c) Data collection scheme for a typical FT-TR-EPR experiment. No CW MW irradiation is present, and MW pulses serve the purpose of producing an observable signal.

**Figure 43:**
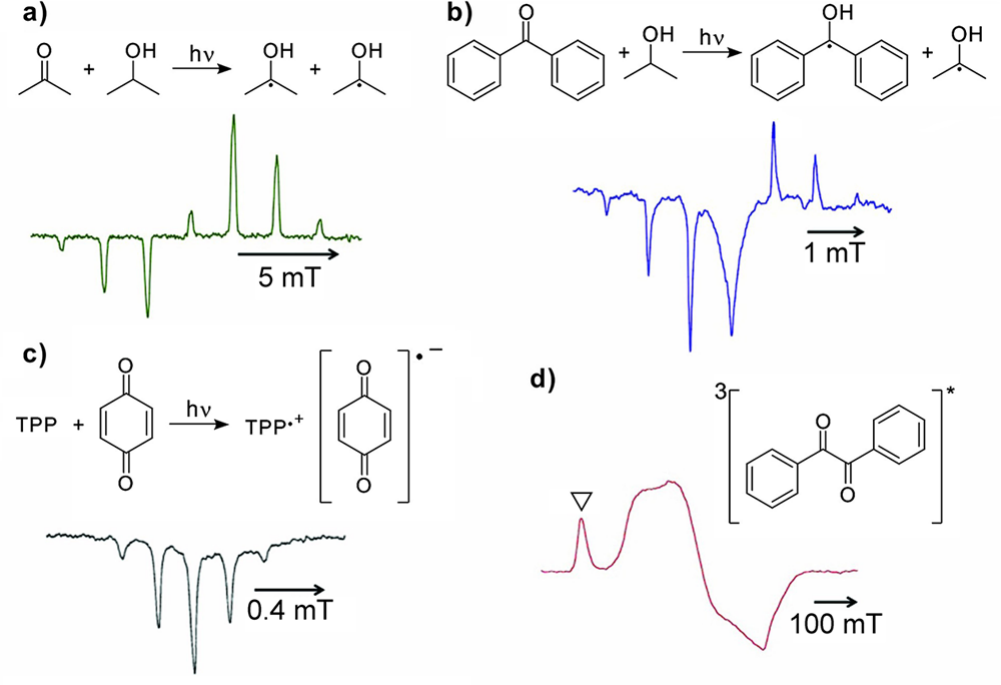
Examples of X-band EPR spectra exhibiting CIDEP effects: (a) acetone/isopropyl alcohol/water (1:1:1) photoexcited at 308 nm; (b) benzophenone (100 mM) in isopropyl alcohol photoexcited at 355 nm; (c) tetraphenyl porphyrin (TPP; 0.1 M) and benzoquinone (10 mM) in 3:1 chloroform/methanol photoexcited at 460 nm; (d) benzil (1 mM) in frozen toluene photoexcited at 308 nm. The triangle denotes the half-field (double quantum) transitions characteristic of a triplet-state molecule. Reproduced with permission from ref (439). Copyright 2013 Elsevier.

The experimental apparatus necessary to generate and observe CIDEP effects is similar to the one employed in TR-EPR.^438,439^ The main components are shown schematically in Figure 42a. Briefly, the setup consists of a microwave bridge, a tunable magnet and an excitation source. These three components are linked to the console. Usually, an excimer or a solid-state (e.g., Nd:YAG) pulsed laser serves as an excitation source to generate transient radicals and/or photoexcited molecules in triplet states. In general, TR-EPR spectra are acquired by either continuous-wave TR-EPR (CW-TR-EPR) or pulsed Fourier-transform TR-EPR (FT-TR-EPR). In the former case (Figure 42b), the CIDEP EPR signals of short-lived radicals or other transient paramagnetic species produced by a laser pulse are read as an output of a fast preamplifier under continuous MW irradiation. These are integrated with a boxcar integrator or a fast digital oscilloscope for a preselected duration after the laser pulse and averaged over a number of laser pulses at a preset value of the magnetic field *B*_0_.^438,439^ Then the magnetic field is stepped to the next value to generate the EPR spectrum. Alternatively, *B*_0_ is slowly swept continuously in some studies. The observation of evolution of CIDEP effects in time is achieved by shifting the signal integration time window and repeating the entire experiment. Several examples of the hyperpolarization-enhanced EPR spectra of paramagnetic species detected in CW-TR-EPR experiments are given in Figure 43. In the case of FT-TR-EPR (Figure 42c), after the laser pulse a short microwave pulse is applied to generate a free induction decay or a spin echo EPR signal. The time-domain data are then either Fourier-transformed to generate the EPR spectrum, or the echo signal is integrated. The temporal evolution of the EPR spectrum exhibiting CIDEP in this case is monitored by changing the delay between the laser pulse and the MW pulse.

Given that CIDEP effects lead to strong perturbations of the electron spin-state populations, they are widely applied to enhance the sensitivity of TR-EPR spectroscopy. Some representative CIDEP applications targeting the enhancement of electron spin polarization are briefly discussed next. Numerous studies exploiting RTPM CIDEP for electron spin hyperpolarization purposes are available in the literature. For instance, CIDEP effects were generated via the RTPM mechanism in the context of the stable radical TEMPO and a few photoinduced triplet states.^440^ Up to 280-fold EPR signal enhancements were observed upon using TEMPO and 1-chloronaphthalene in isopropyl alcohol at 226 K. The enhancement factors were strongly dependent on the translational diffusion coefficient. Raising the temperature from 226 to 270 K resulted in a 7.4-fold increase in translational diffusion coefficient, causing a 74% decrease in the enhancement factor. Moreover, switching the solvent to benzene, a much less viscous liquid than isopropanol, led to an additional 10.4-fold increase in diffusion coefficient and a 97% decrease in enhancement factor. Similar effects were observed in later studies.^90^ More recently, a covalently linked chromophore-radical system capable of achieving up to 320-fold polarization enhancements by effectively restricting the relative translational diffusion of the triplet chromophore and the radical was developed.^441^ Finally, CIDEP in aqueous media was demonstrated for a nitroxide radical using eosin Y or rose Bengal as the chromophore, gaining an impressive polarization enhancement of up to 150-fold.^442^ The latter development in aqueous media paves the way to future applications to biological systems.

**Figure 44:**
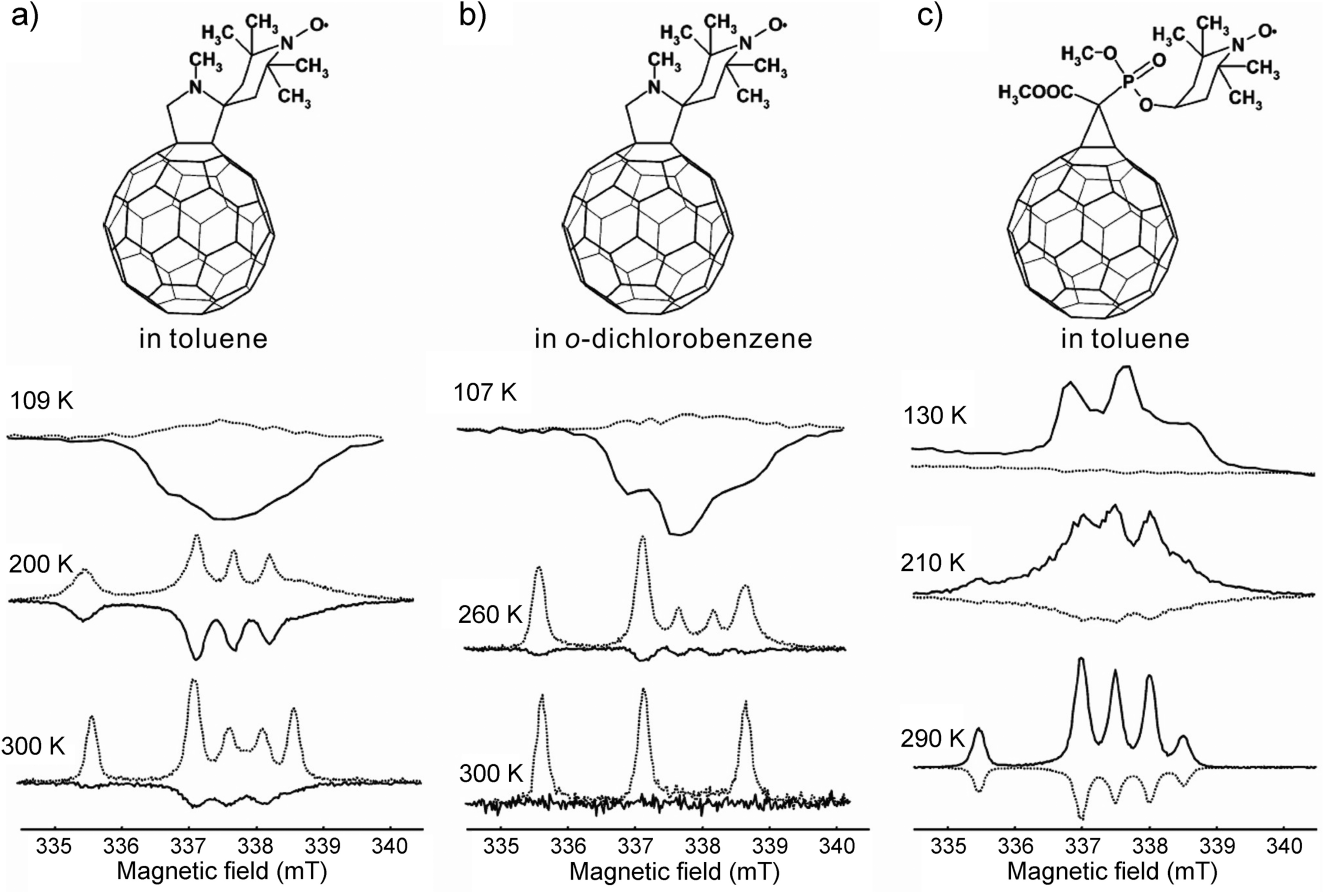
Representative example of RQM-mediated CIDEP at various temperatures and in different solvents. Spectra shown with thick and thin lines correspond to data acquired 0.3 and 1.5 μs after laser irradiation, respectively. The rather complex spectral patterns arise from the mutual coupling of the two paramagnetic species. Reproduced with permission from ref (436). Copyright 2005 American Chemical Society.

While RTPM has been the prevalent CIDEP mechanism when significant enhancements in electron spin polarization have been targeted, CIDEP proceeding via the RQM mechanism has also played a role. RQM was first proposed and developed^436^ for a complex of fullerene with the TEMPO radical, as shown in Figure 44. Later, RQM CIDEP was demonstrated for a pyrene–TEMPO complex, achieving up to a 30-fold enhancement in electron spin polarization.^435^ An additional example of RQM application is provided by recent studies exploring the CIDEP dependence on the chemical nature of photoexcitable chromophores containing a variety of metal ions.^443,444^

An overview of the major CIDEP-mediated electron spin polarization enhancements achieved to date is provided in Table 2.

**Table 2:** Overview of Electron Spin Polarization Enhancements Achieved to Date via CIDEP

CIDEP mechanism	Molecules of interest	Polarization enhancement factor^a^	Refs	Comments
RTPM	TEMPO-anthraquinone	320	(441)	In benzene
RTPM	TEMPO and rose bengal	13.6	(90)	ESP-enhanced DNP, in aqueous media
RTPM	TEMPO and 1-chloronaphthalene	280	(440)	At low temperature
RTPM	Nitroxide and eosin Y/rose bengal	150	(442)	In aqueous media
RQM	2-pyrenemethyl TEMPO	30	(435)	In ether

a Defined as the ratio *p*_e_/*p*_e,therm_, where *p*_e_ is the maximum polarization achieved in the TR-EPR experiment, and *p*_e,therm_ is the polarization at thermal equilibrium.

Thanks to the ability to strongly enhance EPR signals, CIDEP can be employed for structural and dynamic studies of macromolecules, especially when it proceeds via the RTPM mechanism. Given that RTPM requires an interaction between a stable radical and a triplet chromophore, it can be employed to monitor solvent exposure, similarly to the nuclear-spin-dependent photochemically induced dynamic nuclear polarization (photo-CIDNP) technique (section 3.6). For instance, the solvent exposure of tryptophan (Trp) amino acid in proteins can be probed by adding the stable radical TEMPO to protein solutions.^417^ Upon optical irradiation, Trp is converted to its triplet state. If this amino acid is solvent-exposed within the host protein, the triplet state can be quenched by dissolved TEMPO thereby leading to an enhancement of the EPR signal of the nitroxide via RTPM mechanism (Figure 41a). The solvent exposure of chlorophyll in samples including protein-bound chlorophylls can also be probed via the same approach.^445^

Another strategy to investigate the structure and dynamics of macromolecules relies on RTPM-mediated CIDEP for a chromophore-radical pair of the probes covalently attached to a macromolecule. The probes can be naturally occurring (e.g., Trp within a polypeptide or protein) or extrinsic (e.g., 4-benzoylphenylalanine as triplet precursor and 4-amino-1-oxyl-2,2,6,6-tetramethylpiperidine-4-carboxylic acid, a.k.a. TOAC, as a stable radical). Given that polarization generated by RPTM CIDEP is distance-dependent, this method can provide qualitative information on the distance between the probes, thus shedding light on macromolecular conformation and dynamics.^418,446,447^ Triplet states of suitable chromophores hyperpolarized via TM CIDEP were shown to be applicable to sensitive nanometer distance measurements between spin labels by EPR techniques in labeled biomolecules.^448,449^

Overall, the CIDEP phenomenon is triggered photophysically or (photo) chemically and leads to generation of strong hyperpolarization of electron spins in paramagnetic species. These include transient or even stable free radicals, radical-ions, biradicals, and triplet molecular species. Quite often, several CIDEP mechanisms can operate at the same time.^450^ This significant perturbation of the population of electron spin states can be a useful tool in organic photochemistry.^416,438^ Specifically, CIDEP is useful to explore the mechanisms of chemical reactions including the direct and sensitive detection of short-lived reaction intermediates. In addition, CIDEP can be intentionally generated, mostly upon photoirradiation, for enhancing electron spin polarization to learn about solvent exposure of macromolecules. The RTPM and RQM mechanisms are prevalent in applications targeting enhanced electron spin polarization, with the major goal of increasing the sensitivity of EPR. Finally, electron spin polarization generated via CIDEP provides yet another opportunity for increasing NMR sensitivity^90^ (see also sections 3.2, 3.7, and 2.2.2). The progress in the field achieved so far, briefly surveyed here, suggests that CIDEP will become an increasingly popular technology in the future.

### 3.6 Photochemically Induced Dynamic Nuclear Polarization in Liquids

#### 3.6.1 The Technique

In 1967, Bargon and co-workers discovered the presence of unusual emissive resonances in the NMR spectrum of reaction products resulting from the thermal decomposition of dibenzoyl peroxide and di-*p*-chlorobenzoyl peroxide.^451^ During the same year, Ward and colleagues independently discovered the surprising proton nuclear spin polarization generated during the reaction of various organometallic compounds.^452^ In both cases, free radicals were identified as key reaction intermediates, and the phenomenon was named chemically induced dynamic nuclear polarization (CIDNP) based on the (incorrect) assumption that the underlying mechanism was similar to that of OE-DNP (section 3.2). Later, it became clear that similar effects can also be generated upon photochemical triggering. For instance, optically excited photosensitizer dyes can collide in solution with oxidation-prone molecules and undergo redox reactions, giving rise to radical pairs. The latter can then rapidly undergo further processes leading to the conversion of the molecule of interest to a nuclear-spin-hyperpolarized species.^453,454^ This effect is known as photochemically induced dynamic nuclear polarization (photo-CIDNP).

A correct mechanism for the nonphotochemically triggered CIDNP was first proposed in 1969. The process was envisioned to involve formation of transient radical pairs rather than individual radicals^86,455^ and was named the radical pair mechanism (RPM; section 3.5). This process essentially relies on spin sorting by a chemical reaction (section 2.2.5). When a radical pair is produced, the spin states of its unpaired electrons are correlated. The correlated electron spin state is inherited from the electronic spin state (e.g., a singlet or a triplet) of the pertinent precursor (section 3.5). The spin-correlated radical pair then evolves to produce nuclear spin hyperpolarization.

**Figure 45:**
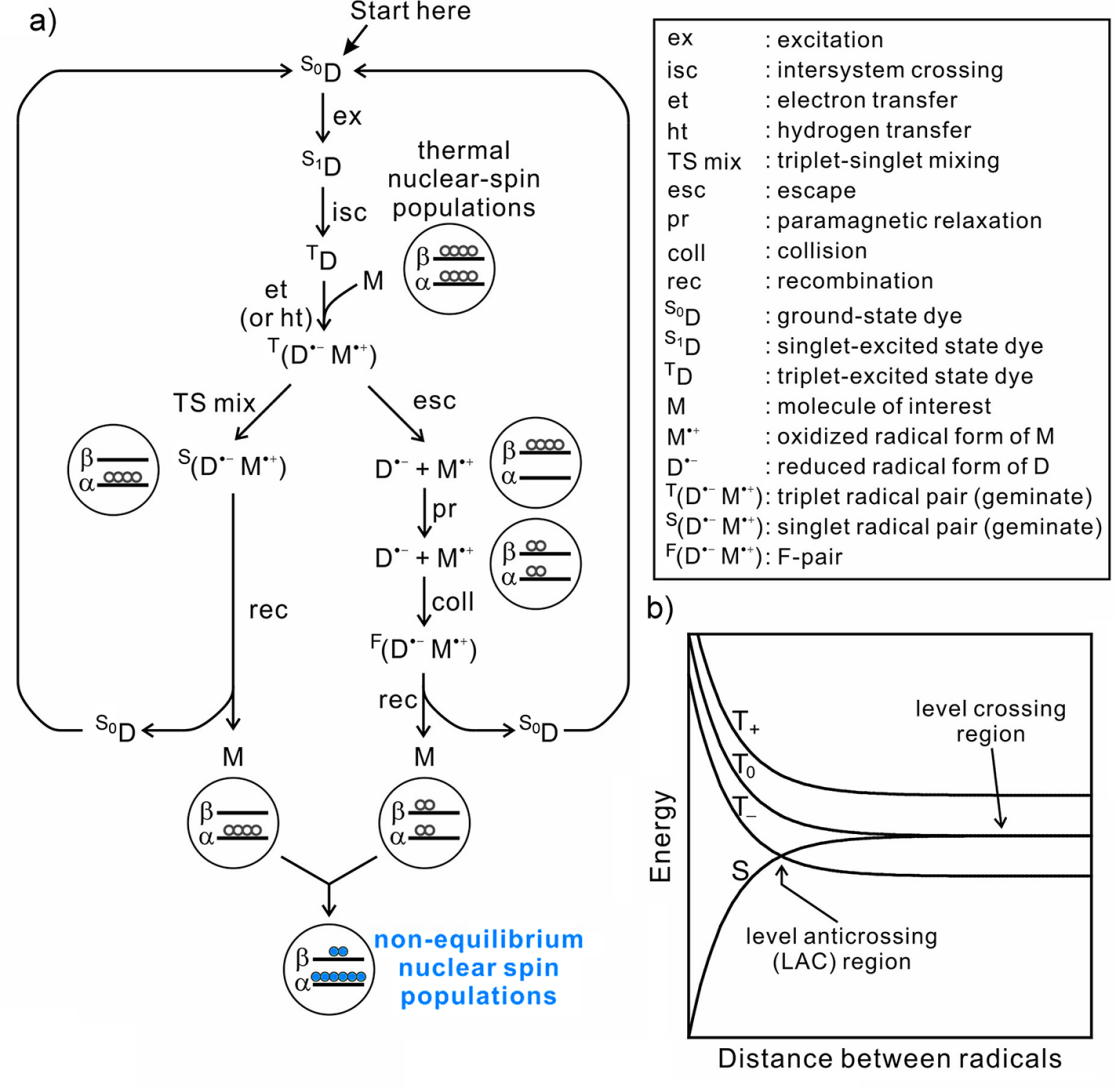
(a) Overview of the radical pair mechanism of photo-CIDNP. This simple scheme assumes a cyclic process. Note that F-pairs may also give rise to additional hyperpolarization (not shown in the image above). (b) Electron spin energies of a radical pair as a function of distance between radical pair components.

**Figure 46:**
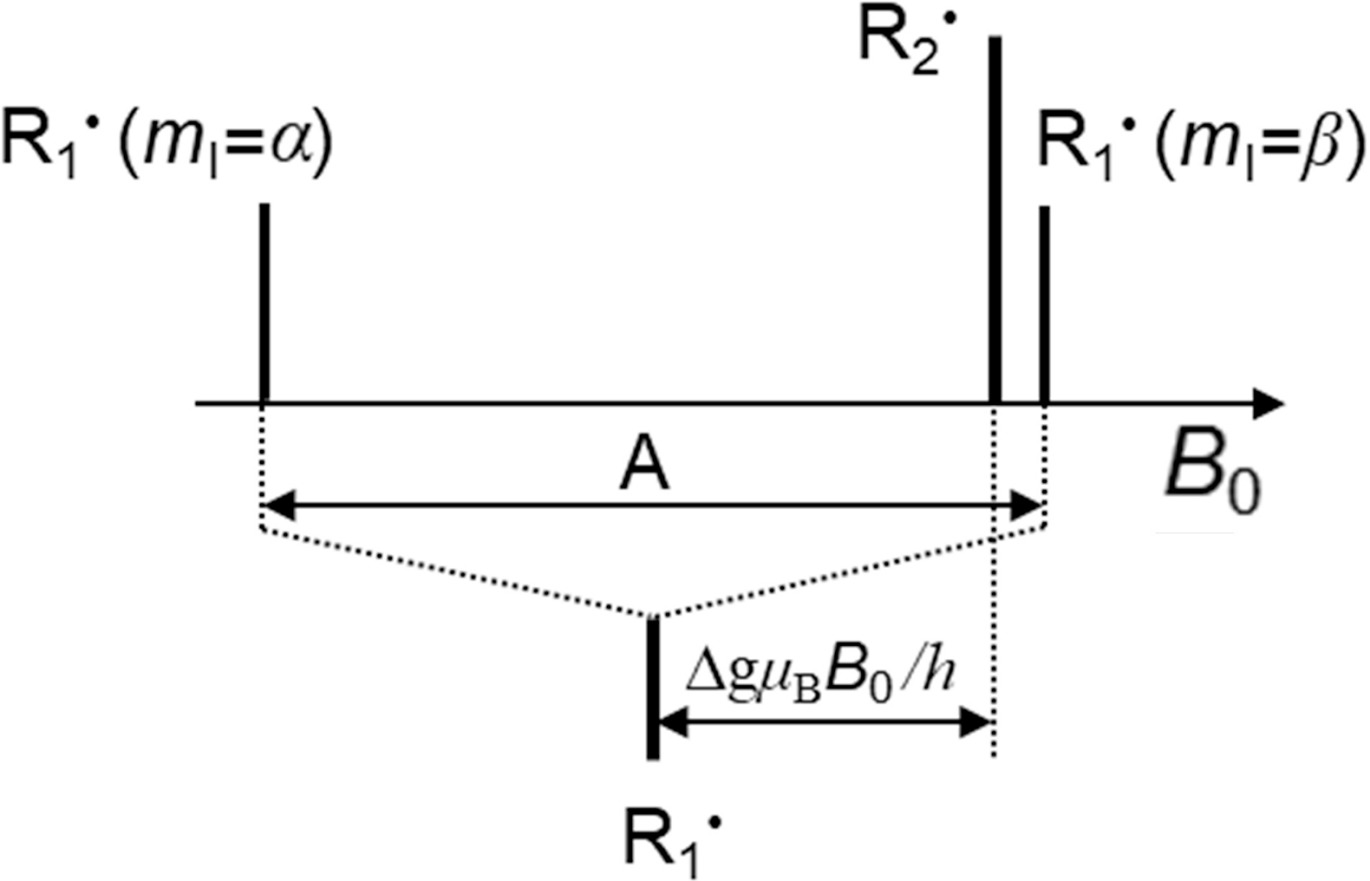
EPR stick-spectrum of a radical pair consisting of two radicals R_1_^•^ and R_2_^•^, with R_1_^•^ carrying a single spin-1/2 nucleus with the hyperfine coupling constant *A*. Larger difference in the resonance frequencies of the two radicals results in a faster singlet–triplet interconversion, which in the illustrated case corresponds to RP with the nucleus in the α spin state.

A similar mechanism was later suggested for the photochemically triggered version of the process (photo-CIDNP) in liquids, and rapidly gained supporting evidence.^453,456^ A scheme illustrating the essential features of this mechanism is shown in Figure 45. Briefly, the ground-state photosensitizer dye (^S0^D) is electronically excited to a singlet excited state (^S1^D). The latter often undergoes intersystem crossing (ISC) and populates a triplet state (^T^D). The triplet-state dye then reacts with the molecule of interest (M) transiently oxidizing it and giving rise to a radical pair (RP) preserving the triplet electron spin state. According to the Pauli exclusion principle, to regenerate the original version of the molecule of interest M, a triplet RP must first undergo triplet–singlet conversion (T-S mixing) to the singlet state before the onset of recombination. The above process is referred to as geminate radical pair recombination, which proceeds extremely rapidly (0.1–100 ns) and does not alter nuclear spin states. Importantly, the T-S mixing is driven by the hyperfine couplings (A) of electron spins of the two radicals constituting the RP and the difference in their *g*-factors (Δ*g*).^457^ Therefore, the T-S mixing frequency depends on the nuclear spin states of both radicals, and is generally more favorable for one nuclear spin state than another, as schematically illustrated in Figure 46. This important property leads to the kinetic sorting of RPs across different chemical pathways based on nuclear spin states of the radicals involved and results in hyperpolarization of the molecule of interest. Figure 45 illustrates a representative example of this phenomenon. Briefly, RPs carrying one nucleus in the α state may preferentially undergo T-S mixing and conserve this nuclear spin state upon their recombination. Under these circumstances, RPs bearing the same nucleus in the β state preferentially escape from a solvent cage and separate into their individual radical components, whose electron spins become uncorrelated.^458^ Individual radicals may then randomly collide with one another and form new radical pairs denoted as F-pairs. The subset of F-pairs bearing the singlet electron spin configuration may then recombine and give rise to the starting parent species. However, before this collision, and depending on relative concentrations, viscosity and temperature, a significant degree of paramagnetic relaxation takes place, on the microsecond time scale. This results in a Boltzmann distribution of nuclear spin states in the cage-escaped radicals and in the loss of the initial overpopulation of the nuclear β state. As a result of the overall process, the nuclear α state in M becomes enriched at the expense of the β state, resulting in positive (absorptive) nuclear spin hyperpolarization. Alternatively, it is also possible that faster T-S mixing may be experienced by radical pairs with a β nuclear spin state, in which case emissive nuclear hyperpolarization is generated in M. The mechanism highlighted above recapitulates only the essential features of the process, and more thorough descriptions are available elsewhere.^459−462^ It is worth noting that the effect of multiple nuclear–electron couplings and the role of additional potential side reactions, e.g., undesired degradation processes, are not included in Figure 45a. Some of the remaining F-pair population (not shown in Figure 45a) can also lead to additional hyperpolarization.^459^

**Figure 47:**
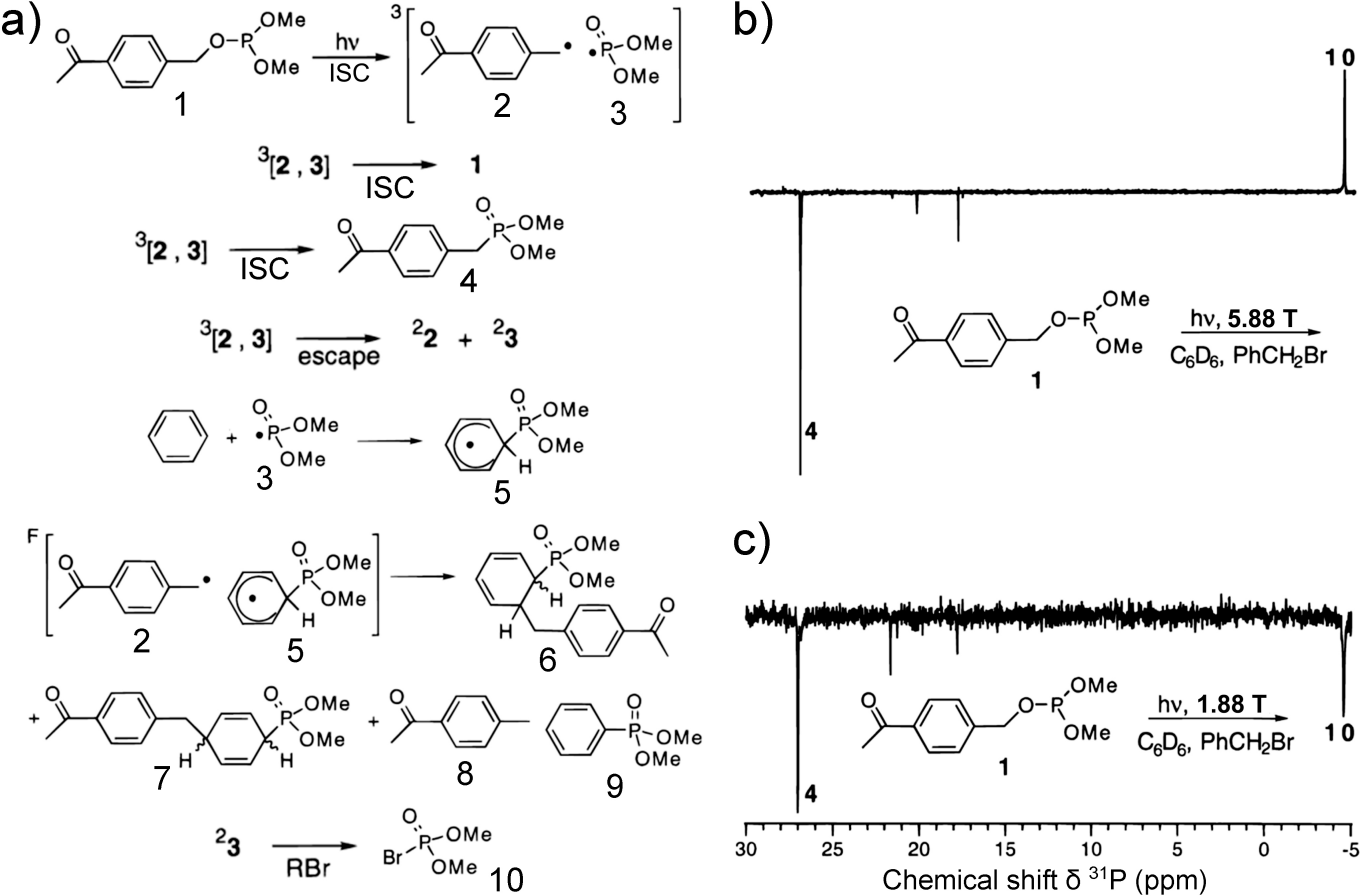
Representative example of photo-CIDNP proceeding via a noncyclic process. (a) The photolysis reaction scheme for *p*-acetylbenzyl dimethyl phosphite (1) in solution. (b,c) ^31^P CIDNP NMR spectra recorded during the photolysis of deoxygenated 0.1 M benzene-*d*_6_ solutions of 1 in the presence of benzyl bromide as a radical scavenger, at high (101.26 MHz, 5.88 T) and low (32.44 MHz, 1.88 T) magnetic fields. Note the sign change of the NMR signal of cage-escape product 10 from absorptive to emissive character at high and low field, respectively, signifying the switch from S-T_0_ to S-T_–_ mixing in the geminate radical pair ^3^[2, 3]. Adapted with permission from ref (463). Copyright 1996 American Chemical Society.

In a number of cases, photo-CIDNP proceeds via a modified mechanism. For instance, photo-CIDNP is noncyclic when the hyperpolarized reaction products differ from the starting materials. This situation is most common in organic photochemical reactions involving neutral radical intermediates.^464^ In this case, RPs are often produced as a result of hydrogen atom abstraction or chemical bond cleavage processes (Figure 47).^463^ The recombination products arising from the geminate RP are often different from the products formed by radicals that escape from the solvent cage. In the latter case, the α and β nuclear spin states, which give rise to enhanced absorptive (A) and emissive (E) NMR signals, respectively, can end up in different products, consistent with Kaptein’s rules.^465^ Another notable example is provided by intramolecular photo-CIDNP initiated by dyes in a singlet excited electronic state. In this case, a mechanism somewhat different from that of Figure 45a applies, as reported in the literature.^27,464,466^ Further, the case of macromolecules bearing a built-in dye (e.g., flavoproteins) with a rotational correlation time longer than the time scale of hyperfine coupling (i.e., the inverse of the hyperfine anisotropy) yet shorter than the chemical shift time scale (i.e., the inverse of the chemical shift anisotropy), is particularly interesting.^467^ When the above conditions are met, a solid-state-like photo-CIDNP process (section 3.8), known as the triplet mechanism, applies. This mechanism operates via nuclear-spin-dependent ISC and dominates at high applied fields. Conveniently, the averaging of chemical shift anisotropy mediated by molecular tumbling enables retention of liquid-like NMR spectral features.^467^

Here, we focus on photo-CIDNP experiments at high applied magnetic fields, where the electronic Zeeman component of the Hamiltonian largely exceeds the hyperfine interaction. Thus, the T_0_ electronic state of the radical pair ends up mixing with the singlet state S, at appropriate inter-radical distances. Conversely, under low-field conditions the electronic Zeeman interaction is comparable to the hyperfine component of the Hamiltonian, and mixing can also occur between the T_+_/T_–_ and the S states, typically referred to as level anticrossing (LAC). Whether LAC occurs between T_+_ and S or between T_–_ and S depends on the relative values and signs of the exchange interaction J between radical pair electrons and the hyperfine coupling *A*.^457,468^ A similar situation is encountered, for instance, in the case of transient biradicals or radical pairs confined in micelles when the exchange interaction of the two unpaired electrons J is nonvanishing (e.g., |J| > |*A*|) owing to their close proximity in space. As J is negative in most common cases, this brings the S and T_–_ (rather than T_+_) states close in energy (Figure 45b) and results in a dominant T_–_-S mixing.^469^

#### 3.6.2 Practical Aspects

To perform photo-CIDNP experiments, a setup comprising an NMR spectrometer and appropriate optical components is required. Conveniently, photo-CIDNP hyperpolarization develops rapidly, within less than 1 s of optical irradiation, and takes place in situ within an NMR tube. Therefore, photo-CIDNP can be carried out with commercially available NMR spectrometers. In essence, in most cases no modifications to the conventional NMR hardware and no sample transfers from a different part of an apparatus are necessary.

**Figure 48:**
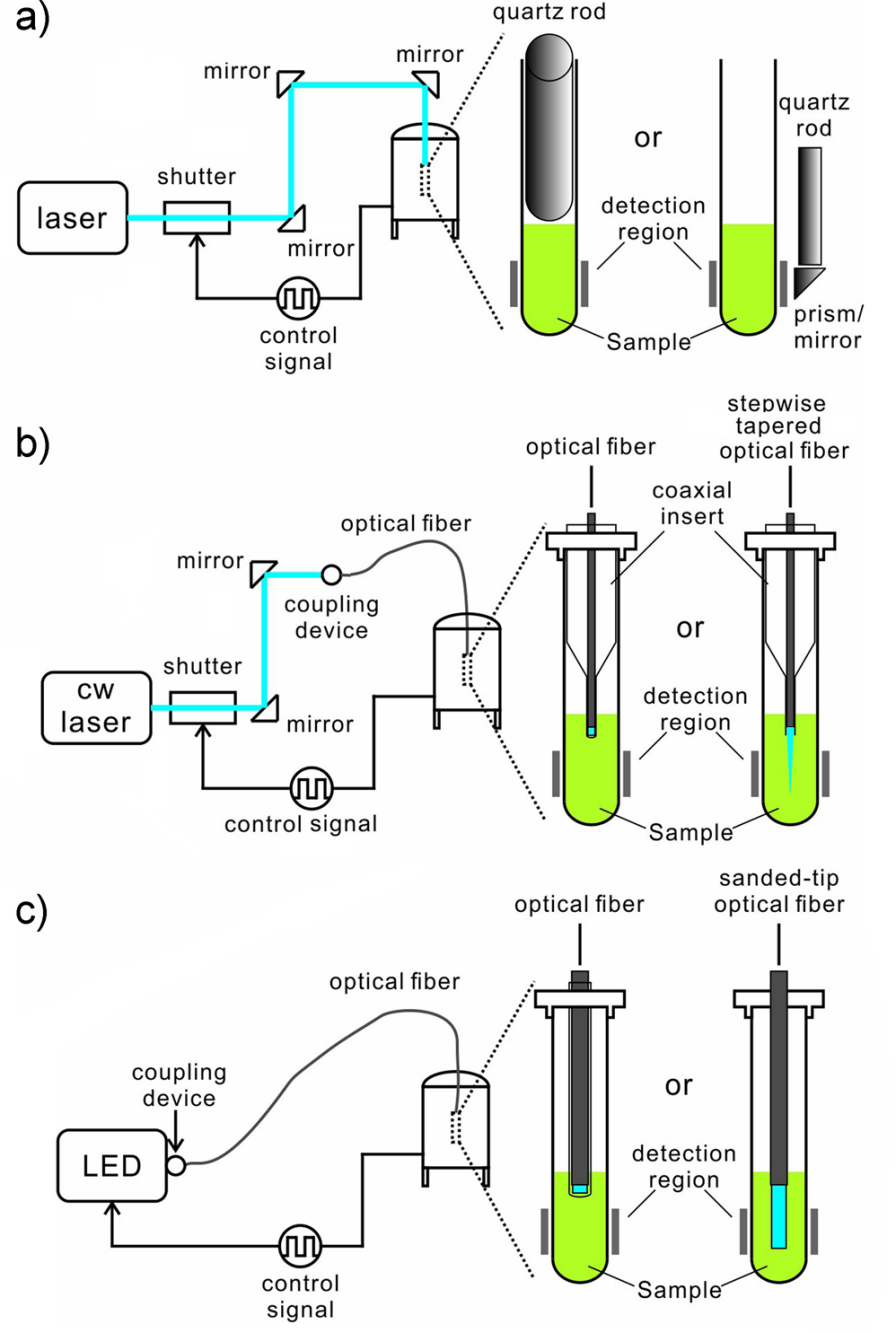
Overview of common photo-CIDNP instrumentation. (a) Experimental setup employing a laser source in conjunction with a quartz rod. (b) Apparatus based on a laser as a light source and optical fiber for light delivery inside the NMR spectrometer. (c) Setup employing a LED light source and an optical fiber.

Numerous types of optical systems of the UV and visible range have been employed in photo-CIDNP to date. While in the early days CW illumination with optically filtered light of a xenon arc or mercury-vapor UV lamps was often used, nowadays the three most representative categories include lasers coupled with quartz rods, laser/optical-fiber setups, and light-emitting diodes (LEDs) coupled with optical fibers (Figure 48).

Directing laser light onto NMR samples via a quartz rod as schematically illustrated in Figure 48a is a common choice. Quartz rods exhibit excellent light transmission in the UV–vis range and are compatible with pulsed lasers. These features are crucial for time-resolved photo-CIDNP experiments, as well as for experiments employing a wide variety of dyes, including those absorbing in the UV region. Such an apparatus is versatile but also not entirely straightforward to implement, especially in terms of installation and alignment. Namely, this setup requires accurate vertical guiding of laser light onto the sample, from either the top or the bottom of an NMR magnet. This arrangement is not always compatible with all spectrometer geometries and room dimensions, and may pose safety challenges, and in addition may lead to light beam attenuation by the sample. An alternative design based upon horizontal irradiation onto the side of the NMR tube^470^ overcomes geometrical issues, yet it requires some NMR probe modifications.

A second illumination strategy uses an optical fiber (Figure 48b) and usually involves a CW laser as a light source with irradiation timing controlled by the spectrometer through a software-controlled shutter. Laser light is guided into the optical fiber via mirrors, lenses, and a fiber coupler. The optical fiber is typically introduced into the NMR tube via a coaxial insert, and the fiber/coaxial-insert/NMR-tube setup is conveniently placed inside the magnet from above, as in conventional NMR experiments. A more sophisticated setup involves stepwise tapering of the optical fiber at the NMR tube end followed by deep insertion into the NMR tube, enabling a more uniform sample irradiation.^470^ This fiber modification is particularly convenient in the case of samples with high optical density. Optical fibers are easier to handle and align than a quartz-rod setup. In addition, unavoidable light losses can be countered upon employing high-power lasers and optional fiber tapering.^470^ A downside of this setup is that commercially available optical fibers cannot typically withstand the high transient energy density of pulsed lasers.^471^ This limitation renders this apparatus incompatible with photo-CIDNP applications requiring pulsed laser irradiation, including time-resolved photo-CIDNP. Moreover, specialized optical fibers are required if CW UV lasers are employed, for example, when irradiation of dyes like 2,2′-bipyridine is desired (irradiation wavelength is 355 nm).

Finally, LEDs have recently replaced lasers in photo-CIDNP^43,472,473^ and other light-assisted NMR technologies.^474^ LEDs are much cheaper, more portable, and easier to handle and maintain than high-power lasers. They are also capable of producing pulsed irradiation with typical rise/fall times of a few μs.^473^ Due to LED output beams being uncollimated, it is preferable to guide the light into the optical fiber directly at the LED source (Figure 48c). To optimize sample irradiation, the fiber tip reaching the NMR sample can be sanded or etched. This procedure exposes part of the fiber core, enabling more pervasive light escape.^473^

LEDs coupled to optical fibers usually deliver less optical power than high-power lasers, and this may in principle be viewed as a disadvantage. As discussed below, however, the recent realization that photo-CIDNP hyperpolarization depends only weakly on optical irradiation power at low (micromolar and submicromolar) concentrations of the molecule of interest,^475^ has actually rendered low-power irradiation preferable. As a consequence, the replacement of lasers with LEDs does not compromise sensitivity in many desirable applications targeting low sample concentrations.^472,475,476^ Overall, the use of LEDs in photo-CIDNP rendered this technique considerably safer, more affordable and easier to implement, and proved to be particularly advantageous for low-concentration photo-CIDNP (LC-photo-CIDNP; see below) experiments.

**Figure 49:**
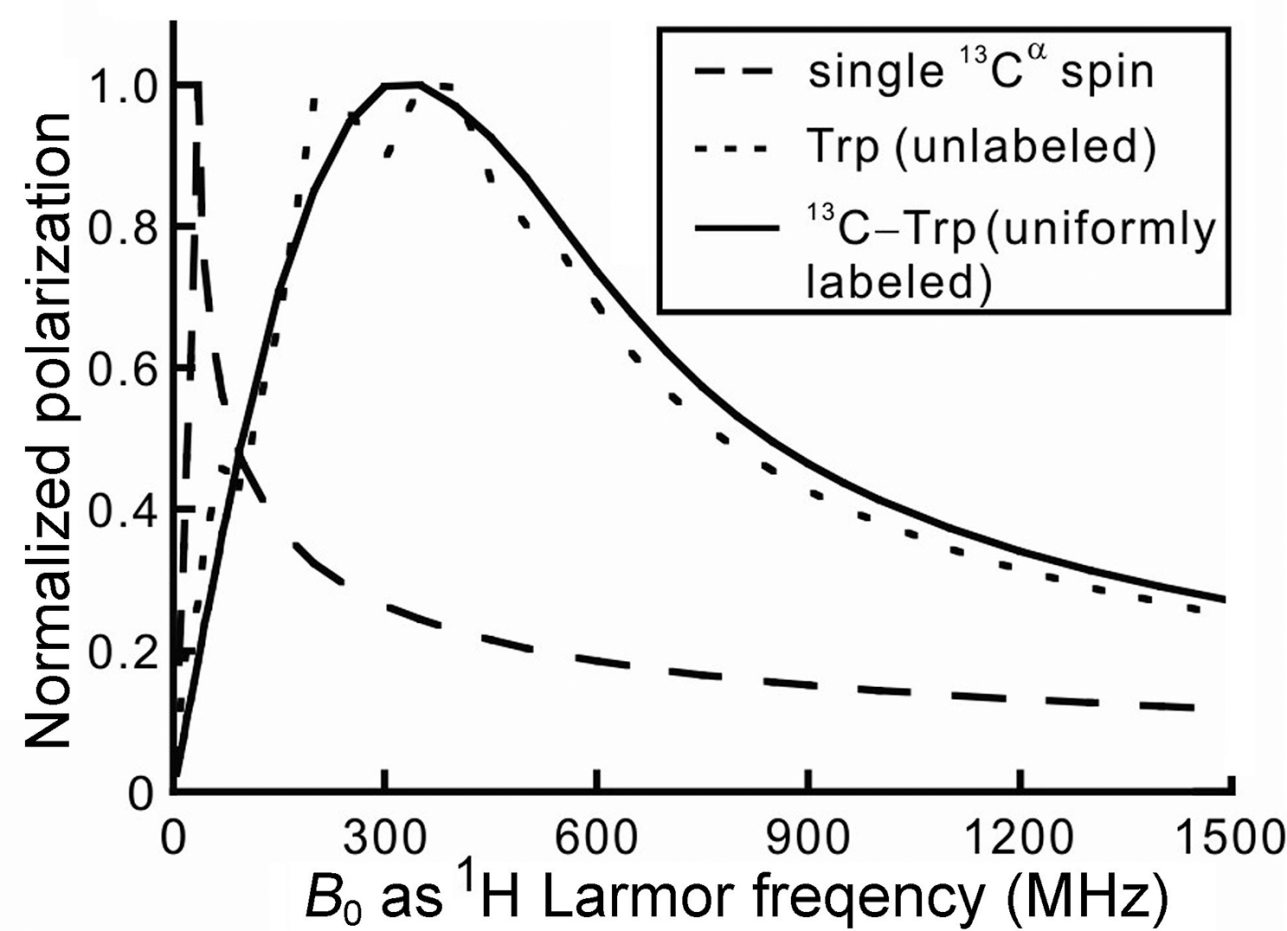
Expected magnetic field (*B*_0_) dependence of photo-CIDNP hyperpolarization of the ^13^C^α^ nucleus of tryptophan (Trp), assuming an isolated ^13^C^α^ (dashed line), a ^13^C^α^ within an otherwise unlabeled Trp (dotted line) and a ^13^C^α^ within a uniformly ^13^C-enriched Trp (solid line). Simulations were carried out for the Trp amino acid as the molecule of interest and for fluorescein as the photosensitizer dye, according to known equations and procedures. Parameters used: translational diffusion coefficients *D*_Fl_ = 4.25 × 10^–6^ cm^2^/s,^480^*D*_Trp_ = 5.40 × 10^–6^ cm^2^/s,^481^ van der Waals radii *R*_Fl_ = 0.44 nm and *R*_Trp_ = 0.38 nm,^482^ hyperfine coupling constant of ^13^C^α^*A* = 0.5643 mT.^484^ Hyperfine coupling constants of other Trp radical nuclei were as reported.^483^ The computed polarizations were normalized relative to the maximum achievable polarization of each species at optimal *B*_0_. The hyperfine coupling constants of the fluorescein hydrogens were not taken into account.

As mentioned above, most studies are performed in situ. An exception to this common practice is the case of field cycling. Briefly, photo-CIDNP polarization of any given sample depends on the strength of the applied magnetic field,^459,467^ and signal enhancements are expected to differ drastically between low fields (e.g., micro- to millitesla) and the commonly employed higher fields of 7.05 to 28.2 T (300 MHz to 1.2 GHz ^1^H frequency).^477^ In cases where greater enhancements are predicted at lower fields, one can carry out photo-CIDNP polarization under low-field conditions and then transfer the sample to a higher field for detection. This procedure has the inherent advantages of preserving the high spectral resolution of high-field NMR while exploiting the higher photo-CIDNP enhancements attainable at low magnetic fields. For this experiment, field-cycling components must be installed to enable generation of a tunable low magnetic field located well above or below the receiver coils, followed by rapid mechanical shuttling of the hyperpolarized sample from the low-field to the high-field area for detection.^478^ The optimal magnetic field for photo-CIDNP hyperpolarization depends on the g-factors and hyperfine coupling constants of the radicals produced in the reaction of interest. Therefore, the potential advantage of switching magnetic field during a photo-CIDNP experiment needs to be evaluated on a case-by-case basis.^459,467^ An example of the expected dependence of photo-CIDNP polarization on an applied magnetic field is provided in Figure 49. The simulations in this figure were carried out according to known procedures,^459,479^ and employed published values of relevant parameters.^480−484^

Another specialized application involves performing photo-CIDNP on microscale-volume samples. For instance, a microfluidic apparatus coupled with a LED and an optical fiber was developed for experiments with 1 μL sample volume.^43^ This setup enables flow-through experiments that accomplish high-sensitivity detection by continuously replenishing the NMR sample, hence bypassing challenges associated with photodegradation, and also enables experiments aimed at probing reaction kinetics via two-channel micromixing.

Given that photo-CIDNP polarization typically builds up and decays relatively fast (<1 s) and in situ, the pulse sequences employed in photo-CIDNP typically include time delays, UV–vis illumination, and radiofrequency (RF) irradiation within the same single-transient train of events. 1D ^1^H photo-CIDNP pulse sequences can be as simple as pulse-acquire preceded by laser or LED optical irradiation, and may include RF pulse schemes for solvent suppression. In addition, ^1^H, ^13^C, or ^15^N presaturation may be performed before optical irradiation to eliminate thermal polarization, so that the resulting spectrum reflects solely photo-CIDNP hyperpolarization.^485−488^

In order to increase the overall sensitivity, experiments involving pulsed lasers may feature multiple laser pulses before one single RF pulse train. The magnetization can then be temporarily stored in the transverse plane between individual laser pulses.^489^ The experiments targeting heteronuclei (i.e., photo-CIDNP effects for nuclei other than ^1^H) often involve polarization transfer to ^1^H, to further enhance sensitivity. This purpose is efficiently fulfilled by pulse sequences employing reverse-INEPT or similar RF pulse schemes.^472,475,490,491^

In many cases, 2D experiments involving heteronuclei utilize photo-CIDNP-adapted heteronuclear single- or multiquantum correlation (HSQC, HMQC) pulse schemes or simpler 2D reverse-INEPT RF schemes, and usually involve photo-CIDNP-enhanced ^13^C/^15^N coherence.^490−493^ Other more complex pulse schemes reported to date involve initial transfer of ^1^H thermal polarization to ^15^N before optical irradiation, resulting in the constructively combined ^1^H thermal and ^15^N photo-CIDNP polarizations, yielding higher sensitivity.^491^ Another fairly sophisticated approach includes the use of a second optical irradiation per transient. This procedure enables photo-CIDNP enhancement of ^1^H followed by magnetization transfer to ^15^N and another optical irradiation to enhance ^15^N polarization, so that the two photo-CIDNP enhancements add up, yielding higher sensitivity.^491^ Photo-CIDNP ^1^H-^13^C heteronuclear correlation spectroscopy has been particularly popular and has led to the development of a class of pulse sequences^490^ based on the reverse-INEPT RF scheme which share the early generation of strong ^13^C hyperpolarization followed by coherence transfer to ^1^H and detection.^490^ Standard constant-time evolution and pulsed-field-gradient sensitivity enhancement schemes can be included.^494−496^

The basic photo-CIDNP pulse sequence ^13^C PRINT^490^ (photo-CIDNP-enhanced constant time reverse INEPT) was optimized^475^ (^13^C PREPRINT, perturbation-recovered PRINT) for additional sensitivity advantage provided by cryogenic NMR probes.^497^ Selective ^13^C 180° flip-angle pulses were recently introduced in experiments targeting the photo-CIDNP enhancement of α-carbons (^13^C^α^) to enable full decoupling of carbonyl carbons (^13^C′) from ^13^C^α^. The resulting pulse sequence is known as ^13^C PRESPRINT (perturbation-recovered selective-pulse PRINT).^476^ Finally, it should be noted that photo-CIDNP pulse sequences do not usually need the conventional recycle delay (1–2 s) as most of the nuclear polarization relevant to photo-CIDNP is generated within 0.1–0.2 s of optical irradiation.^498^ The ^13^C RASPRINT (rapid-acquisition selective pulse PRINT) combines this advantage with optimal solvent suppression to further increase sensitivity by 2-fold.^472^

Table 3 provides an overview of the sensitivity advantages provided by the major photo-CIDNP pulse sequences and experiments developed to date.

**Table 3:** Summary of NMR Sensitivity Advantages Achievable via Photo-CIDNP Nuclear Polarization

Molecule of interest	Enhanced/detected nucleus	Photosensitizer dye	Pulse sequence type^a^	Percent polarization *p*, %^d^	Enhancementfactor ε^g^	Sensitivity enhancement	Lowest detected conc. (amount)^k^	Ref
Tryptophan (U-^13^C, ^15^N)^b^	^13^C^α^/^1^H^α^	FMN	^13^C-^1^H rev-INEPT (^13^C PRINT)	0.016%^e^	13.5 ± 0.6	22 ± 3^h^	1.0 mM (0.14 mg)	(490)
Tryptophan (U-^13^C, ^15^N)^b^	^1^H^ε1^/^1^H^ε1^	FMN	^1^H-^15^N HSQC	0.031%^e^	6.0 ± 0.3	6.0 ± 0.3^h^	2.0 mM (0.29 mg)	(492)
Tryptophan (U-^13^C, ^15^N)^b^	^13^C^η2^/^1^H^η2^	FMN	^13^C-^1^H rev-INEPT (^13^C PRINT)	0.033%^e^	41 ± 2	11.8 ± 20.7^h^	1.0 mM (0.14 mg)	(490)
Tryptophan (U-^13^C, ^15^N)^b^	^15^N^ε1^, ^1^H^ε1^/^1^H^ε1^	FMN	^1^H-^15^N HSQC	0.048%^e^	100 ± 15	100 ± 15^h^	2.0 mM (0.29 mg)	(492)
Tryptophan (^15^N_2_-Trp)^b^	^15^N^ε1^/^1^H^ε1^	FMN	^1^H-^15^N HSQC	0.15%^e^	30.9 ± 0.8	30.9 ± 0.8^h^	2.0 mM (0.29 mg)	(491)
Tryptophan (U-^13^C, ^15^N)^c^	^13^C^α^/^1^H^α^	Fluorescein	^13^C-^1^H rev-INEPT (^13^C PREPRINT)	0.353%^e^	292	1,800^h^, 73^i^	200 nM (28 ng)	(475)
Tryptophan (U-^13^C, ^15^N)^c^	^13^C^α^/^1^H^α^	Fluorescein	^13^C-^1^H rev-INEPT (^13^C RASPRINT)	0.375%^e^	310 ± 16	832 ± 44^h^,208 ± 11^i^	500 nM (76 ng)	(472)
Tryptophan (α-^13^C)	^13^C^α^/^1^H^α^	Fluorescein	^13^C-^1^H rev-INEPT (^13^C RASPRINT)	0.496%^e^	410 ± 2	1,168 ± 61^h^, 292 ± 15^j^	n.a. (est. <28 ng)^i^	(484)
Tryptophan (α-^13^C-β, β,2,4,5,6,7-d_7_)	^13^C^α^/^1^H^α^	Fluorescein	^13^C-^1^H rev-INEPT (^13^C RASPRINT)	0.568%^e^	470 ± 13	1,306 ± 69^h^, 327 ± 17^j^	20 nM (2.8 ng)	(484)
Tyrosine	^1^H/^1^H	Atto Thio 12	Pulse-acquire	0.30%^e^	59	59^h^	10 μM	(499)
*N*-Acetyl-l-tyrosine	^1^H/^1^H	FMN	Pulse-acquire	0.019%^f^	6	6^h^	5 mM (1.1 μg)	(43)
Nucleotide (GMP)	^1^H/^1^H	FMN	Pulse-acquire	0.023%^f^	7	7^h^	20 mM (7.3 μg)	(43)
*p*-Fluorophenol	^19^F/^19^F	FMN	Pulse-acquire	n.a.^l^	n.a. (>500)	n.a. (>500)^h^	0.8 μM (90 pg)	(43)

a Rev-INEPT denotes a reverse-INEPT pulse sequence type.

b Can be detected both in its free form or as a residue within proteins. The tyrosine (Tyr) and histidine (His) amino acids can also be detected, though the sensitivity is somewhat lower.

c Can be detected both in its free form or as a residue within proteins. The tyrosine (Tyr) amino acid can also be detected, though the sensitivity is somewhat lower.

d *p* is defined according to eq 1 and is expressed in percent. These values were not explicitly reported in the original references and were assessed here according to the reported enhancement factor, experimental methods and thermal polarization (according to the Boltzmann distribution).

e Polarization values calculated at 600 MHz, consistent with the original reference.

f Percent polarization values were assessed at 400 MHz, consistent with the original reference.

g The enhancement factor ε defined as the ratio between photo-CIDNP polarization and thermal polarization: ε = *p*/*p*_therm_, where *p*_therm_ is the polarization at thermal equilibrium.

h Sensitivity enhancement factor relative to the same pulse sequence under dark conditions (sensitivity enhancement = (SNR)_t. light_/(SNR)_t, dark_).

i Sensitivity enhancement factor relative to ^1^H-^13^C CT-SE-HSQC (constant-time sensitivity-enhanced HSQC). Sensitivity enhancement = (SNR)_t,light_/(SNR)_t,HSQC_.

j Approximate estimate from comparisons between SE-HSQC and dark-state ^13^C RASPRINT data.

k Smallest amount of detectable material according to the corresponding reference. The true detection limit is lower or equal to the reported value.

l Not available (i.e., not quantified in the original publication).

**Figure 50:**
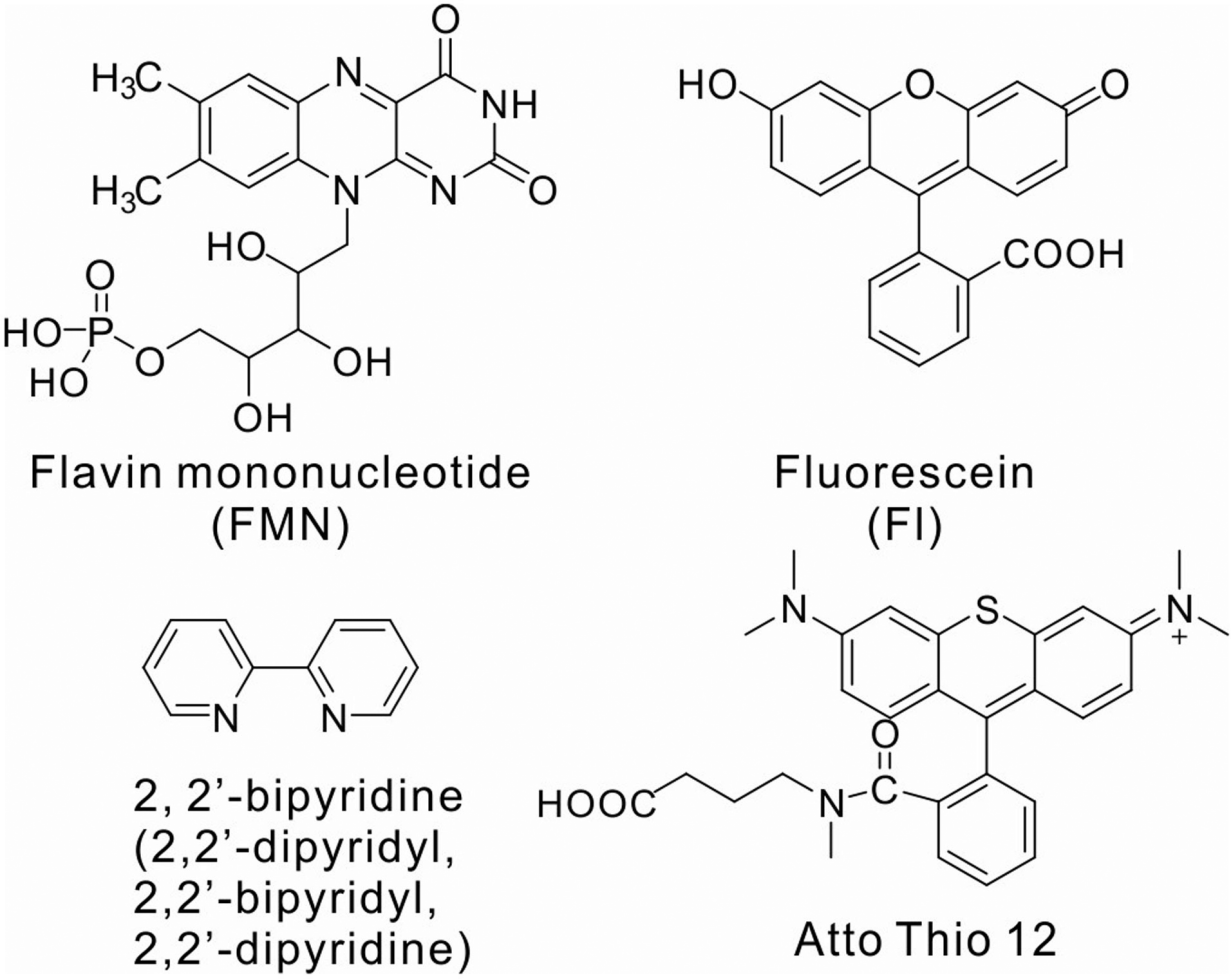
Chemical structure of common photosensitizer dyes employed in photo-CIDNP and LC-photo-CIDNP.

The basic composition of a photo-CIDNP sample in liquids can be fairly simple. In addition to the molecule of interest and the solvent, a suitable photosensitizer is required. A number of different photosensitizer dyes have been employed in photo-CIDNP to date. Some representative examples are shown in Figure 50. Historically, flavins were the most popular dyes in photo-CIDNP experiments designed to identify the solvent exposure of the amino acids tryptophan, tyrosine and histidine within proteins.^453,454^ Flavin mononucleotide (FMN) has been particularly useful for this purpose.^500^ Flavins proved to be effective at mediating photo-CIDNP in aromatic amino acids and proteins^453,454,500^ as well as in nucleotides (including oligonucleotides).^501^ Another commonly used photosensitizer is 2,2′-bipyridine (a.k.a. 2,2′-dipyridyl)^478,502^ which absorbs light in the UV region of the spectrum. Fluorescein, a well-known chromophore (and fluorophore) absorbing in the visible region of the spectrum, was introduced as an efficient dye tailored to photo-CIDNP experiments targeting low-concentration samples^503^ (see below). Finally, the recently introduced Atto Thio 12 dye leads to higher enhancements than other dyes for the specific detection of tyrosine in solution.^499^ It is worth noting that photosensitizer dye and the molecule of interest do not have to be separate entities. For instance, flavin-bound proteins can undergo intramolecular photo-CIDNP. In this case, the endogenous flavin is the dye, and aromatic residues within the protein serve the role of the molecule of interest. In addition, dyads, i.e., molecules encompassing covalently linked electron-donor-bridge-acceptor units, can also undergo intramolecular photo-CIDNP. In this case, optical irradiation leads to donor photoexcitation, followed by intramolecular transfer of electron from the donor to the acceptor, leading to the formation of a biradical.^504^ The sensitivity gains afforded by the major photo-CIDNP sensitizer dyes are summarized in Table 3.

Importantly, the concentrations of dye and the molecule of interest influence the degree of achievable polarization. Higher dye concentrations, however, do not necessarily lead to higher photo-CIDNP polarization, as overly concentrated dyes attenuate light transmission and prevent most of the sample from being effectively irradiated.^470,503^ An optimal dye concentration depends on sample tube and fiber-optic dimensions and geometry, as well as on the dye extinction coefficient and light wavelength. Tailor-shaped optical fiber tips^470^ and microscale sample volumes^452^ have enabled the use of higher dye concentrations. Another challenge is posed by the fact that for a few photosensitizer dyes (e.g., fluorescein), intrinsic fluorescence leads to partial depopulation of the excited singlet state of the dye back to the ground state S_0_. This measurably reduces the yield of ISC to the triplet state of the dye upon optical excitation, thus reducing the photo-CIDNP sensitivity advantage. To circumvent this problem, additives containing heavy atoms can be introduced to accelerate ISC^505^ via a phenomenon known as the external heavy atom effect.^506^

**Figure 51:**
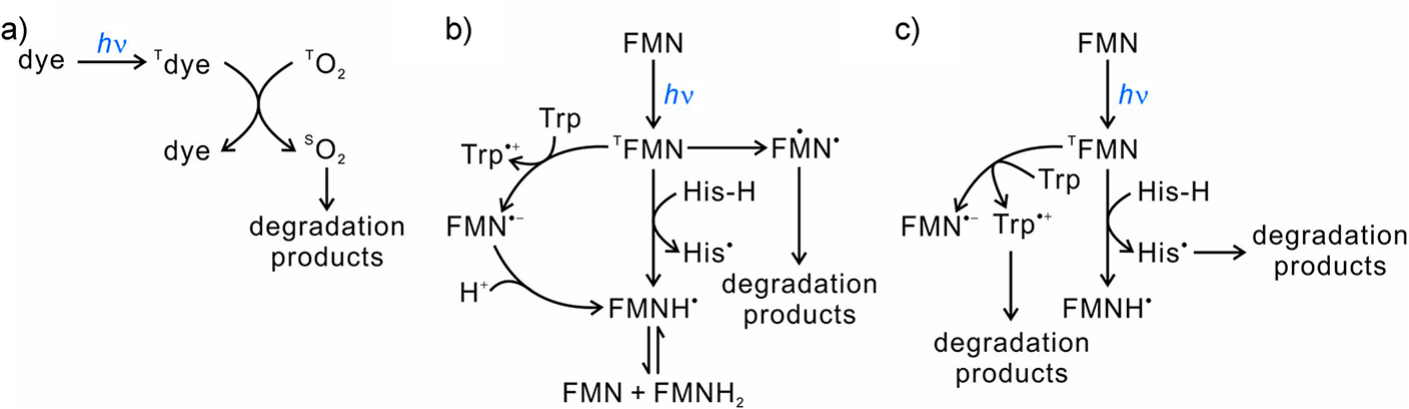
Overview of degradation pathways of dye and molecule of interest during photo-CIDNP. (a) Degradation of dye and molecule of interest mediated by singlet oxygen. (b) Degradation of FMN dye mediated by its diradical form generated as a photo-CIDNP byproduct. (c) Degradation of the molecule of interest (Trp or His) mediated by the radicals that are typically transiently generated during photo-CIDNP in solution.

The basic sample composition and preparation procedures described above, however, are far from being ideal. Optical irradiation of a sample implies that a certain extent of photodegradation is unavoidable. Photodegradation processes may affect both the photosensitizer dye and the molecule of interest, and are usually mediated by excited electronic states or radical intermediates, including the escape species in Figure 45. In particular, the transiently generated triplet state of the dye can interact with molecular oxygen present in solution,^507,508^ typically at ca. 280 μM concentration. As schematically shown in Figure 51a, collisions with molecular oxygen not only quench the triplet state of the dye, but also generate highly reactive singlet oxygen which promotes sample degradation and is known to generally take place in the presence of a wide variety of dyes.^509,510^ Other common light-induced degradation pathways, summarized in Figure 51 in the case of the FMN dye, include slow degradation of dye and molecule of interest via radicals of both species (Figure 51b,c) and singlet oxygen-mediated processes (Figure 51a).

Photodegradation prevents long-term data collection in photo-CIDNP^475,508,511^ such as signal averaging or multidimensional NMR. A number of strategies were recently adopted to circumvent the above challenges. First, singlet-oxygen-mediated degradation can be prevented by removing molecular oxygen from photo-CIDNP NMR samples. This is done by either Ar/N_2_ purging^512^ or via addition of oxygen-scavenging enzymes, typically glucose oxidase (GO) and catalase (CAT).^508,513^ Molecular oxygen concentration can be monitored in solution with commercially available devices.^472^ It is worth noting that oxygen depletion becomes increasingly important at lower concentrations of the molecule of interest.^475,508^ Second, the extent of dye degradation can be attenuated by chemically reversing the actual degradation process^508,514^ or by selecting particularly photostable dyes.^503^ Third, degradation of both components can be substantially reduced upon employing low- or sub-μM concentrations of reductive radical quenchers (e.g., ascorbic acid).^515^ Alternatively, photodegradation can be altogether bypassed by rejuvenating or replacing the sample during data collection, for instance, by stirring the sample after each signal acquisition in a 2D NMR experiment to recursively enrich the optically irradiated region with fresh intact molecules.^516^ Alternatively, a microfluidic continuous-flow apparatus was employed to replenish NMR samples in real time during data collection.^43^

Interestingly, dissolved oxygen can sometimes produce beneficial effects. When FMN is employed as a dye, 1,5-dihydroriboflavin 5′-(dihydrogen phosphate), typically denoted as FMNH_2_, is generated^517^ during a photo-CIDNP experiment (Figure 51b). In this case, part of the dissolved molecular oxygen can reoxidize FMNH_2_ back to FMN, thus enabling multiple rounds of photo-CIDNP via dye recycling. Yet, the overall unfavorable effects listed above justify the need to deplete molecular oxygen even when FMN is employed as the dye. Approaches adopted to reoxidize FMN include addition of H_2_O_2_,^514^ mechanical mixing^514,518^ and addition of catalytic amounts of the nitrate reductase (NR) enzyme, which specifically oxidizes FMNH_2_ back to FMN even in the absence of molecular oxygen.^508^

#### 3.6.3 Applications

To date, photo-CIDNP has been exploited either as a reporter of solvent exposure,^454,492,519^ as a probe of organic reactions involving radicals,^27,464,520−523^ or as an NMR sensitivity enhancement tool. For instance, time-resolved photo-CIDNP can be employed to measure the *T*_1n_ relaxation time of aromatic radicals in proteins. This parameter provides valuable information about protein global and local dynamics.^524^ Time-resolved photo-CIDNP also enables the analysis of supramolecular host–guest interactions. By acquiring photo-CIDNP NMR and EPR data, one can learn valuable information on the structure, reaction mechanism, *g*-factors and hyperfine coupling constants of radical intermediates pertaining to reactions of interest.^525^ In addition, under appropriate zero-to-low field conditions, photo-CIDNP is capable of generating long-lived nuclear spin states with relaxation times of the order of tens of seconds.^526−528^ These long-lived spin states pave the way to investigations focusing on slow processes.^527,528^ Further, theoretical arguments predict that the ratio between regular *T*_1n_ and the relaxation time of the corresponding long-lived nuclear spin state depends on molecular geometry.^529^ This property was exploited to determine torsion angles of a variety of small-molecule isotopologues.^529^

In addition to studies focusing on ^1^H, ^13^C, and ^15^N, photo-CIDNP hyperpolarization of other nuclei has also been investigated. For instance, ^19^F-labeled Tyr was hyperpolarized via ^19^F photo-CIDNP, achieving a ca. 14-fold sensitivity enhancement, and enabling ^19^F MRI of this compound.^530^ Another application involved ^31^P photo-CIDNP to study the interaction between phosphonium-iodonium ylide and *p*-methoxyphenylacetylene.^531^ This investigation yielded interesting information about the mechanism of the reaction between the two compounds.^531^

Next, a more recent class of applications that primarily target photo-CIDNP as a source of nuclear spin hyperpolarization and sensitivity enhancement in liquids is illustrated below via a number of representative examples. Table 3 summarizes the current status of photo-CDNP nuclear hyperpolarization in solution, whereas photo-CIDNP in solids falls into a different mechanistic category addressed separately in section 3.8.

In liquids, the aromatic amino acids tryptophan, tyrosine, and histidine can be hyperpolarized via photo-CIDNP and give rise to sensitivity enhancements of up to 200-fold,^472^ both as free amino acids and within proteins.^454,460,472,475,490,492,500^ In addition, other aromatic molecules including nucleotides,^460,501^*p*-fluorophenol^43^ and oligophenols^460^ can be hyperpolarized. In 1999, Hore and Dobson^492^ in their experiment on ^1^H-^15^N-tryptophan achieved 6- and 100-fold sensitivity improvement for ^1^H and ^15^N nuclei, respectively, relative to the spectra acquired without sample illumination.^492^ These authors also collected photo-CIDNP-enhanced 2D ^1^H-^15^N data on the hen lysozyme protein. Later, this approach was further developed by introducing new pulse sequences that enhance NMR sensitivity^491^ more significantly by exploiting ^13^C hyperpolarization.^490^ In addition, the use of catalytic amounts of commercially available oxygen-depleting enzymes to effectively remove molecular oxygen was exploited.^508^ The combined use of the latter two approaches resulted in a ca. 20-fold improvement in NMR sensitivity (defined as SNR_t_ = SNR/(time)^1/2^, with time denoting the total experiment time) relative to reference ^1^H-^13^C constant-time spin–echo HSQC (CT-SE-HSQC) experiments.^508^

To the best of our knowledge, the largest ^13^C polarization enhancement achieved via photo-CIDNP was observed on a variant of the light-oxygen-voltage-sensing (LOV) domain of phototropin, a blue-light receptor protein responsible for regulating the phototropic response in higher plants.^467^ This protein bears an FMN moiety that serves as an intramolecular photo-CIDNP sensitizer. An astonishing 6,000-fold polarization enhancement of the ^13^C^γ^ of this protein’s Trp_491_ was observed.^467^

**Figure 52:**
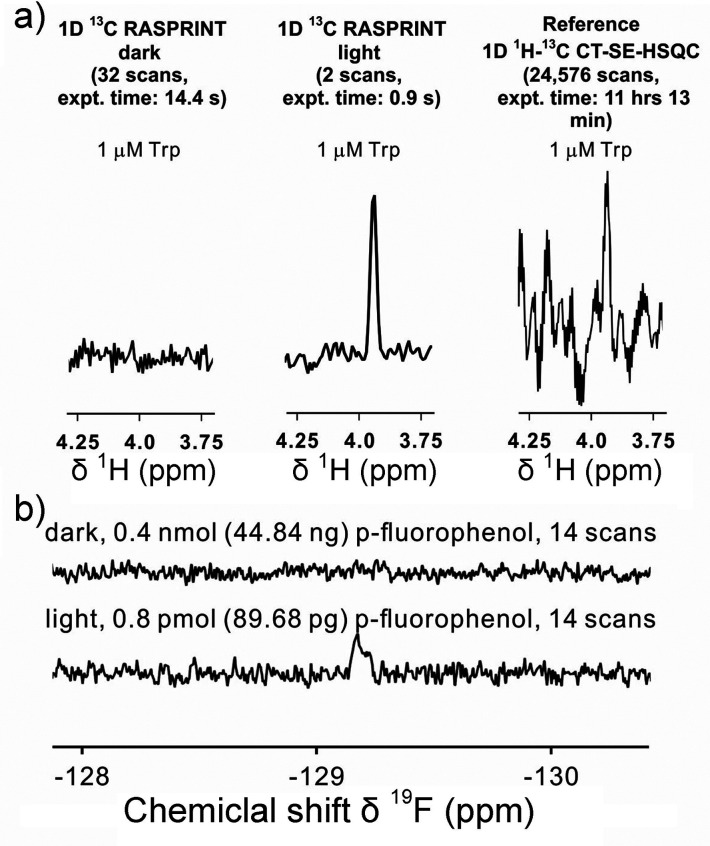
Overview of the sensitivity advantages of LC-photo-CIDNP for Trp and *p*-fluorophenol in solution. (a) Comparison between the sensitivity of LED-driven ^1^H-detected ^13^C LC-photo-CIDNP and the reference ^1^H-^13^C CT-SE-HSQC experiment. The number of scans of the reference HSQC experiment was adjusted to achieve SNR similar to LC-photo-CIDNP. Note that, due to the intense residual solvent signal, strong baseline distortions are observed in the H^α^ region. The noise level away (ca. 7–8 ppm) from the residual solvent signal was used to estimate SNR. A 208-fold sensitivity enhancement relative to reference HSQC was observed. (b) Sensitivity of microvolume (1 μL samples) ^19^F photo-CIDNP performed on *p*-fluorophenol. (a) Adapted from ref (472) with the permission of AIP Publishing. (b) Reproduced from ref (43). Copyright 2018 The Authors. Published by Springer Nature under CC BY license.

Intriguingly, the coupling of ^1^H photo-CIDNP with microfluidics enabled a low detection limit, namely 5 nanomoles (1.1 μg) of *N*-acetyl-l-tyrosine and 20 nanomoles (7.3 μg) of guanosine monophosphate (GMP). These ^1^H photo-CIDNP experiments were performed on microscale sample volumes (1 μL).^43^ The authors also performed ^19^F photo-CIDNP with the same apparatus, and were able to detect 0.8 picomoles (90 pg) of *p*-fluorophenol (Figure 52b).^43^

#### 3.6.4 Frontiers and Challenges

**Figure 53:**
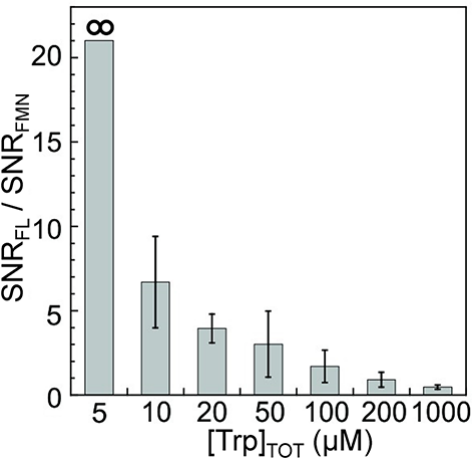
Ratio of photo-CIDNP enhancements for Trp obtained with two different photosensitizer dyes, namely fluorescein (FL) and flavin mononucleotide (FMN). The notation of infinity indicates that signal from FMN-mediated photo-CIDNP was below detection limit for 5 μM Trp. Adapted with permission from ref (503). Copyright 2016 American Chemical Society.

In 2016, it was demonstrated that the fluorescein dye significantly outperforms FMN as a photo-CIDNP sensitizer at low concentrations of the molecule of interest (low- to submicromolar). For instance, the mere replacement of FMN by fluorescein led to an additional sensitivity enhancement of more than 5-fold at a 10 μM concentration of the molecule of interest (Figure 53), enabling efficient detection of 1 μM of uniformly labeled ^13^C-^15^N-Trp within 8 scans at room temperature on a 600 MHz NMR spectrometer.^503^ This effect is primarily due to the long lifetime of the triplet excited state of this dye (20 ms)^507^ relative to other traditional photosensitizers.^532^ This property is of key importance despite the fact that the intrinsic fluorescence of this photosensitizer leads to a reduced yield of ISC to the triplet state. The long-lived triplet state of fluorescein is essential to warrant sufficient time for molecular collisions of photoexcited triplet-state dye with the molecule of interest, especially at low concentrations of the molecule of interest (low micromolar to nanomolar) and dye (low- or submicromolar). Conveniently, enhancements become much higher at lower concentrations of the molecule of interest (Figure 53).

The superior performance of fluorescein at low sample concentrations led to the trend of combining long-lived triplet-state photosensitizers with low- to submicromolar sample concentrations in an oxygen-free environment. This modus operandi resulted in the introduction of a new branch of photo-CIDNP denoted as low-concentration photo-CIDNP or LC-photo-CIDNP.^475^ This particularly convenient nuclear hyperpolarization technology enables the detection of highly dilute solutions of the molecules of interest (in the low-micromolar and nanomolar range). In contrast, all prior photo-CIDNP applications require sample concentrations of at least hundreds of micromoles per liter.

Photo-CIDNP experiments where fluorescein serves as the dye employ low-micromolar to nanomolar sample concentrations and conventional 5 mm NMR tubes and fiber-optic tips.^503^ The optimal dye concentration is within the low-micromolar to submicromolar range.^503^ This straightforward and minimally perturbative arrangement is sufficient to yield enhancement factors ε > 300, and more than 200-fold sensitivity enhancements in SNR_t_ (Table 3). Conveniently, the above setup is applicable to a variety of molecules of interest, including the amino acids tryptophan (Trp) and tyrosine (Tyr), either in isolation or within proteins. Laser-driven LC-photo-CIDNP in the presence of glucose, GO/CAT oxygen-depleting enzymes and the fluorescein photosensitizer dye led to the rapid acquisition of spectra of 200 nM Trp,^475^ with 55-fold sensitivity enhancements of aromatic resonances relative to ^1^H-^13^C SOFAST-HMQC (band-selective optimized flip-angle short-transit HMQC). LC-photo-CIDNP is also effective on dilute proteins. Namely, 500 nM and 5 μM ^13^C-labeled proteins were recently detected via 1D and 2D LC-photo-CIDNP, respectively.^475^ With the advent of LED-driven LC-photo-CIDNP, comparable detection limits were achieved at a lower cost, with a 208-fold increase in sensitivity for Trp α-protons (500 nM, 76 ng) relative to ^1^H-^13^C CT-SE-HSQC (Figure 52a).^472^ Further, the combination of LED irradiation and selective isotope labeling, in the presence of small amounts of ascorbic acid (2 μM), recently enabled the detection of 20 nM (2.8 ng) Trp in solution.^484^ In this study, LC-photo-CIDNP attenuation effects where minimized upon employing Trp-α-^13^C-β,β,β,2,4,5,6,7-*d*_7_, a Trp isotopologue bearing a ^13^C^α^-^1^H^α^ pair surrounded predominantly by non-NMR-active and low-γ nuclei.^484^

Given that LC-photo-CIDNP is tailored to very low concentrations of the molecule of interest (low- or submicromolar), dyes with a long triplet-state lifetime and oxygen depletion strategies are an absolute requirement.^475^ Further, LC-photo-CIDNP displays a dependence on key NMR parameters which is different from that of conventional photo-CIDNP. The most important difference is the nuclear spin hyperpolarization dependence on optical irradiation power.

**Figure 54:**
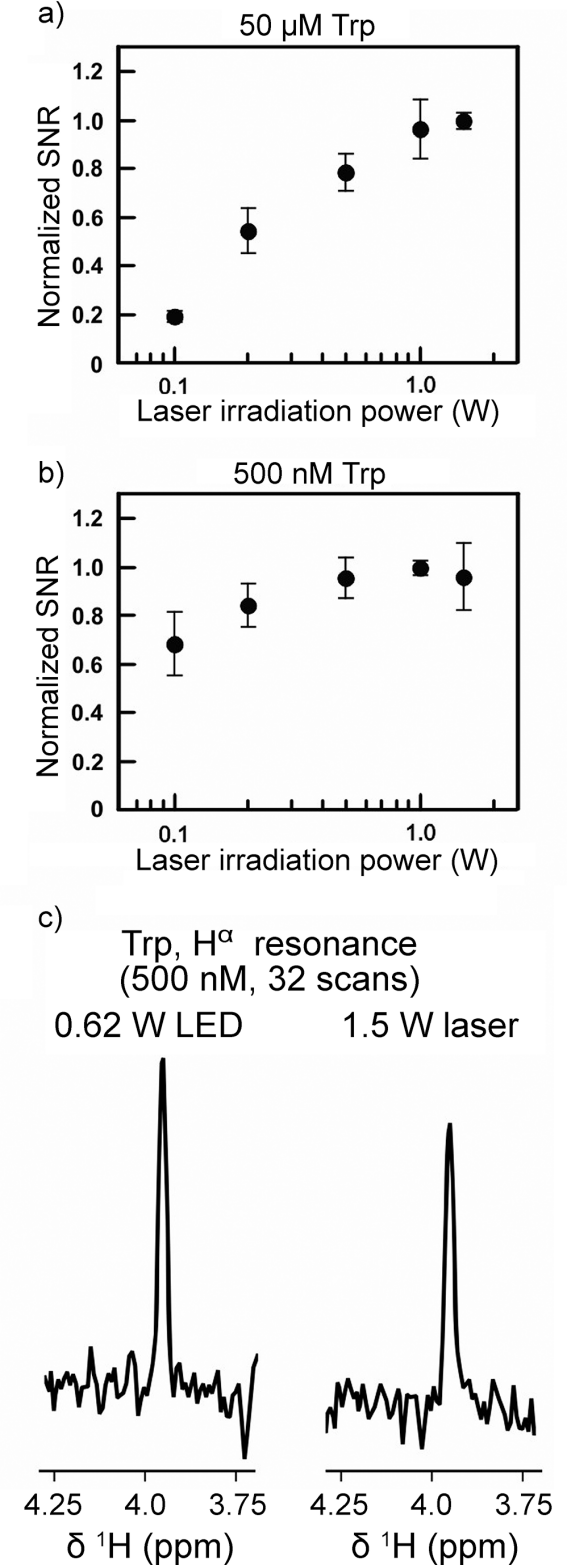
Dependence of photo-CIDNP SNR on the power of optical irradiation at low sample concentrations. All data pertain to tryptophan (Trp) in aqueous solution at room temperature and pH 7.0. (a) 50 μM Trp. (b) 500 nM Trp with optical irradiation by a 1.5 W argon ion laser at 488 nm. (c) Comparison between the SNR of LC-photo-CIDNP experiments employing a 1.5 W laser and a 0.62 W LED, respectively. (a,b) Adapted with permission from ref (475). (c) Adapted from ref (472) with the permission of AIP Publishing.

Higher optical irradiation power enhances the transient population of the triplet excited state of the dye, ^T^D. At concentrations of the molecule of interest exceeding ca. 10 μM, this effect leads to more collisions between ^T^D and the molecule of interest M, ultimately leading to a higher photo-CIDNP polarization. At lower concentrations of M, however,^475,476^ the reaction between ^T^D and M causes a more sizable depletion of ground-state M, thereby reducing the steady-state concentration [M]^SS^. Under these conditions, [M]^SS^ becomes nearly inversely proportional to [^T^D]. Thus, the product of [^T^D] and [M]^SS^ becomes roughly constant and nearly independent of optical irradiation power. As a result, the optical irradiation power dependence of LC-photo-CIDNP is much weaker than that of regular photo-CIDNP (Figure 54a,b). This feature is practically convenient because it enables the use of safer and cheaper low-power light sources, including LEDs, with no loss in sensitivity (Figure 54c).^472^

**Figure 55:**
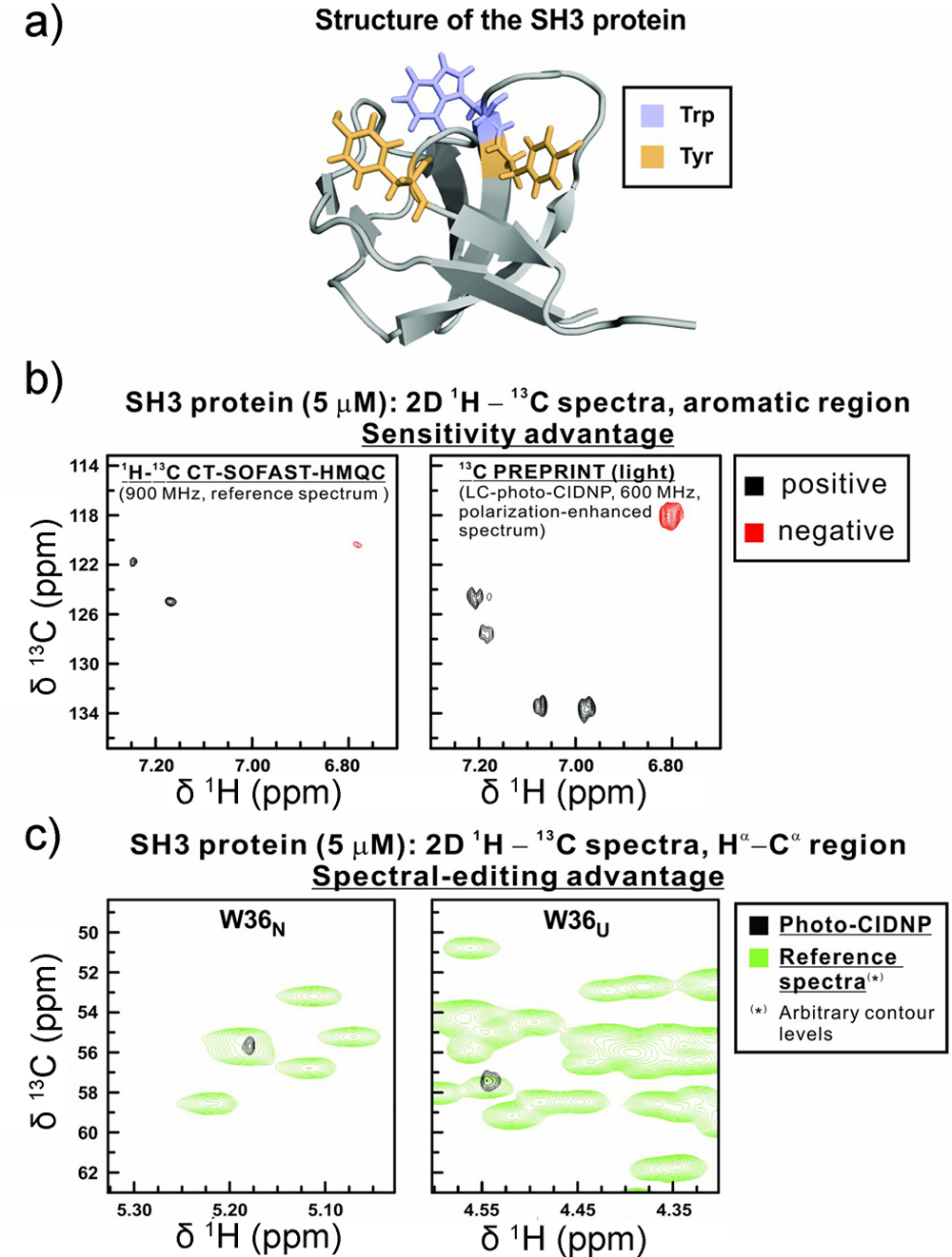
(a) 3D structure of SH3 protein. (b,c) 2D spectra of SH3 protein acquired with conventional NMR and LC-photo-CIDNP. Data were collected in aqueous solution at pH 7.2 and 24 °C. In (b), comparison between NMR spectra of 5 μM SH3 protein acquired via 2D ^1^H-^13^C SOFAST-HMQC and 2D ^1^H-^13^C LC-photo-CIDNP (^13^C PREPRINT) under dark and light conditions. The same total experimental time applies to all three experiments. In (c), spectral overlap between 2D LC-photo-CIDNP spectrum of 5 μM SH3 protein (^13^C PREPRINT pulse sequence) and reference ^1^H-^13^C CT-SE-HSQC spectra highlights the spectral editing capabilities of LC-photo-CIDNP in the H^α^-C^α^ region. Adapted with permission from ref (475).

Due to its fast in situ polarization buildup, LC-photo-CIDNP can also be readily combined with 2D NMR techniques, which is particularly useful in the case of macromolecules, e.g., proteins, bearing many NMR-active resonances (Figure 55a). For instance, an LC-photo-CIDNP 2D ^1^H-^13^C correlation spectrum was recently acquired in 2.5 min at 5 μM protein concentration on a 600 MHz NMR spectrometer (Figure 55b).^475^ Finally, it is worth noting that photo-CIDNP provides spectral editing, given that only aromatic residues are selectively enhanced, resulting in a convenient reduction in spectral complexity (Figure 55c).

Overall, photochemically induced dynamic nuclear polarization enables significant enhancements in NMR sensitivity in solution under mild conditions. This technology bears an exciting potential for additional future advances, including the extension to other biomolecules and the detection of even lower sample concentrations, down to the low- and subnanomolar concentration regimes.

### 3.7 Stimulated Nuclear Polarization

Nuclear spin sorting via the radical pair mechanism of CIDNP (section 3.6) relies on spontaneous nuclear-spin-dependent T-S interconversion in transient RPs driven by magnetic interactions of electron spins. In contrast, in the stimulated nuclear polarization (SNP) technique^128,533^ the rate of T-S interconversion is altered by applying a resonant MW field to the electron spins. At low powers, MW irradiation can affect a single EPR transition of RP, thereby accelerating T-S conversion only in a subset of RPs with a selected configuration of their nuclear spin states. Similar to CIDNP, faster T-S transition for RPs with α or β nuclear spin state followed by recombination of the RP will result in enhanced absorption or emission, respectively, in the NMR spectrum of the diamagnetic product. As both CIDNP and SNP processes act concurrently, SNP effects are usually extracted as the difference in NMR signal intensities with and without MW irradiation, plotted vs the *B*_0_ field at fixed MW frequency. This dependence is sometimes called an “SNP spectrum” of an RP. As the power of the incident MW irradiation is increased, this first leads to faster T-S transitions in RPs and larger SNP effects. However, for MW fields B_1_ comparable to the hyperfine couplings in the radicals, spin-locking effects can cause a sign change of the observed SNP effects or even their suppression.^128,534^

The equipment required to perform SNP experiments^128^ combines the elements used for CIDNP (section 3.6) and OE-DNP (section 3.2). A light source (a laser or a UV lamp) is used to produce transient radical pairs or biradicals by illuminating a solution of suitable reactants. In addition, a source of MW irradiation is required to manipulate the unpaired electron spins. The EPR and NMR parts of the experiment are commonly separated in space and time by implementing sample transfer/shuttling. The sample solution can be continuously flown, from the cell which combines sample photoexcitation and MW irradiation in a relatively low magnetic field of an auxiliary magnet, to the probe of a commercial NMR spectrometer. As the transient free radicals are no longer present during the sample transfer, the *T*_1n_ times of diamagnetic products (a few to a few tens of seconds) are sufficient for the transfer of a polarized sample. SNP experiments have been performed at different fields and MW frequencies: ca. 100, 300, and 1500 MHz (technically, the RF range) as well as ca. 9.5 GHz. In the latter case, commercial X-band EPR equipment can be used.^535^ In one implementation,^536^ both EPR irradiation and NMR detection were performed at 300 MHz by shuttling an entire probe between an electromagnet and a superconducting NMR magnet.

A time-resolved variant of SNP^536,537^ requires a pulsed laser and a pulsed MW source and is implemented by varying the delay between the light pulse and the leading or trailing edge of a MW pulse, or sometimes its duration. The achieved time resolution can be on the order of 5–30 ns, which is sufficient to, e.g., separate the contributions of geminate and F-pairs (RPs formed upon random collisions of transient radicals; section 3.6) to the observed nuclear polarization. Multiple laser pulses can be used to accumulate sufficient NMR signal in the diamagnetic products.

In an SNP experiment, the magnetic field is swept or stepped across the entire EPR spectrum while the MW frequency is kept constant, and the intensity of a chosen signal in an NMR spectrum of a reaction product is monitored. At relatively low MW powers, the resulting field dependence of the SNP effect is essentially an EPR spectrum of the transient RP. Since the RPs are short-lived (e.g., nanoseconds), the lines in such spectrum are measurably broader than one observes in conventional EPR spectra in liquids. It should be noted, however, that short-lived RPs are often not directly detectable by EPR.

In contrast to TR-EPR which attempts to detect transient radicals during their lifetime, in SNP the effect of MW irradiation on RPs is stored in the nuclear polarization of the diamagnetic reaction products. Different reaction products can be chosen for observation to reflect different chemical prehistory of the RPs in complex reactions. Furthermore, different types of nuclei (e.g., ^1^H, ^13^C, ^31^P) and different signals in the acquired NMR spectrum can be interrogated.^128^

**Figure 56:**
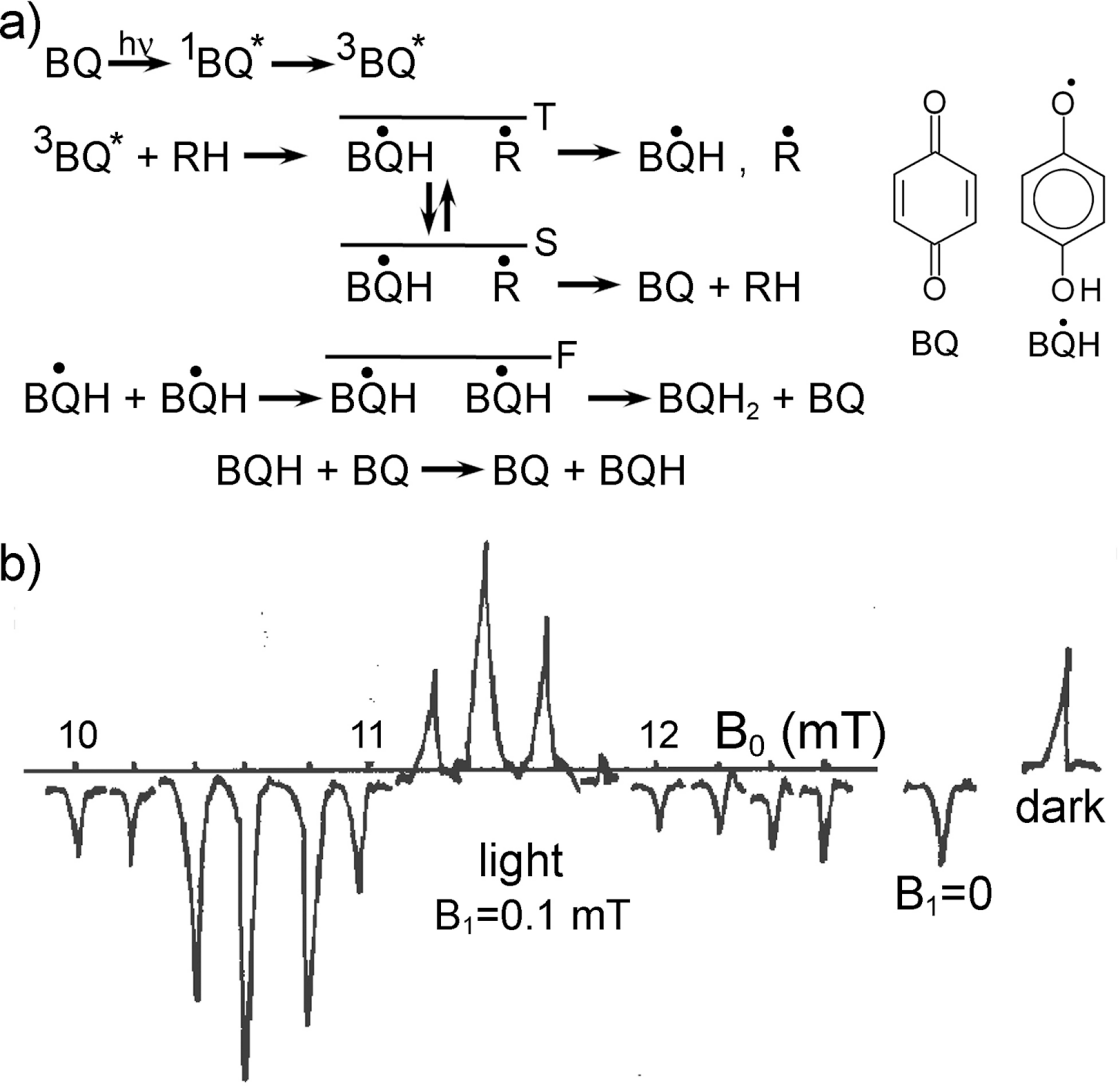
(a) The reaction scheme of *p*-benzoquinone (BQ) photolysis in solution. RH is a hydrogen atom donor (e.g., a solvent molecule); B^•^QH is the transient semiquinone radical. (b) The dependence of benzoquinone ^1^H NMR signal on the magnetic field *B*_0_ during its photolysis in CD_3_CN with a UV lamp. The electron spin transitions are irradiated in an auxiliary electromagnet at 310 MHz (*B*_1_ = 0.1 mT) while the sample is continuously flowing from the reaction cell to an NMR tube in the probe of a 200 MHz NMR spectrometer. The signal of BQ is extracted from the spectrum and is shown centered at the corresponding *B*_0_ value. The signal labeled “dark” is acquired in thermal equilibrium; the one labeled “*B*_1_ = 0” is the signal showing CIDNP effect (without MW). Reprinted with permission from ref (537). Copyright 1989 Elsevier B.V.

SNP effects for RPs composed of neutral radicals have been addressed in photochemical reactions involving ketones, aldehydes, peroxides or quinones.^128^ An example of an SNP spectrum obtained upon photolysis of *p*-benzoquinone is shown in Figure 56. SNP effects were also studied^128,538^ in radical-ion pairs produced in photoinduced electron transfer reactions.

**Figure 57:**
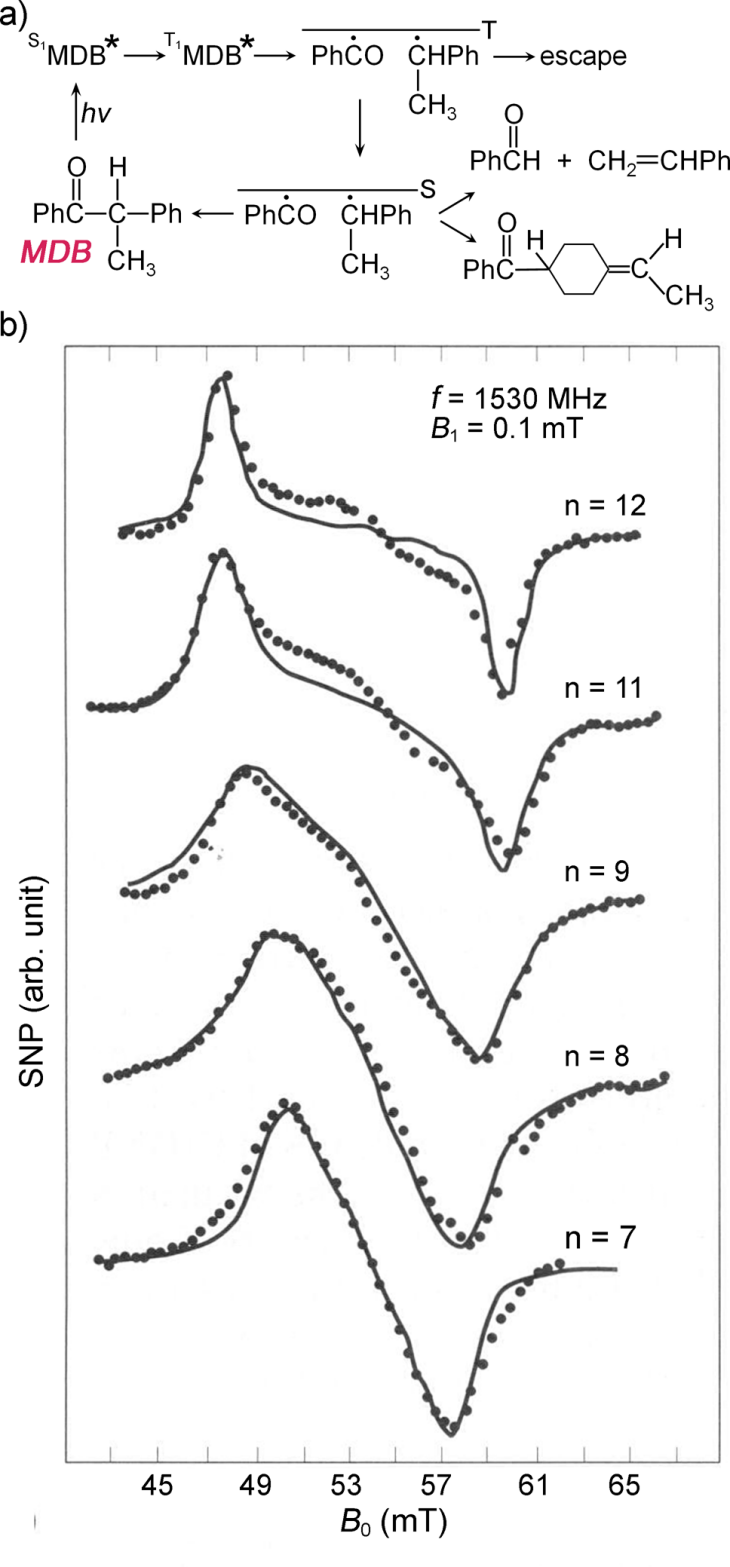
(a) The reaction scheme of α-methyldeoxybenzoin (MDB) photolysis. (b) SNP spectra detected at 1530 MHz MW frequency via carbonyl ^13^C NMR signal of MDB during its photolysis in aqueous micellar solutions (detergent molecule CH_3_(CH_2_)_*n*−1_SO_4_Na; *n* = 7, 8, 9, 11, 12). Solid lines show results of model calculations. Republished from ref (533) with permission of Walter de Gruyter and Co.

A number of SNP studies addressed RPs in micelles composed of alkyl sulfate surfactants in an aqueous medium produced via chemical bond cleavage or hydrogen atom abstraction upon photolysis of ketones.^539−541^ The lifetimes of micellized RPs (100–1000 ns) are sufficient to monitor their kinetics in time-resolved SNP experiments. Furthermore, since two radicals of an RP are forced to stay close to each other, the exchange interaction (J) of the unpaired electrons significantly affects the spin dynamics and the shape of SNP spectra (similar to CIDEP effects, section 3.5). In particular, it was reported^539,541,542^ that with decreasing micelle size by using surfactants with a shorter alkyl chain, the separation of the extrema in SNP spectra tends to decrease to one-half of the corresponding hyperfine coupling (HFC) value (Figure 57), the feature which is well-known from the EPR spectra of, for example, stable biradicals in the limit of strong/fast spin exchange.

An even more rigorous restriction on the separation of the radical centers is imposed in transient biradicals produced, for instance, upon photoinduced cleavage of cyclic ketones.^536,543−545^ This yields a biradical with a flexible polymethylene chain which links the two paramagnetic centers. Similar to RPs in micelles, the conformation-averaged value of the exchange interaction J can be manipulated by changing the polymethylene chain length in the parent ketone. For shorter chains, this leads to a switch in the predominant T-S mixing mechanism from T_0_-S to T_–_-S (for negative J),^544,545^ and an associated change in the SNP patterns from E/A or A/E type to the entirely emissive SNP spectra as the result of T_–_α-Sβ spin transitions. The separation of the lines in the SNP spectra can be reduced to half the HFC value not only for large values of |J|, but also by fast recombination of RPs and biradicals even when J = 0.^543^ Time-resolved SNP studies of biradicals addressing kinetic information were also reported.^536^

**Figure 58:**
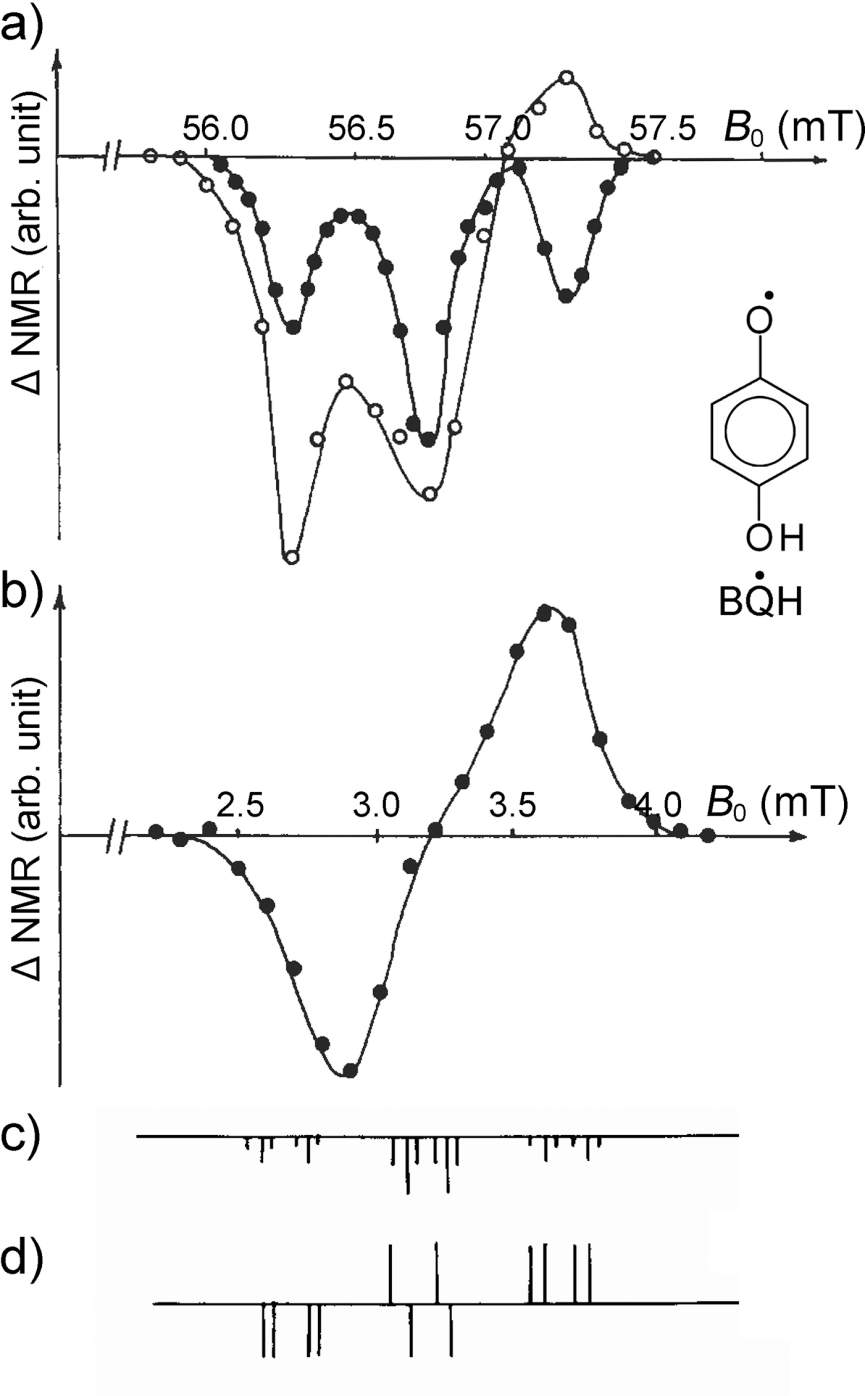
(a,b) The *B*_0_ dependence of benzoquinone NMR signal upon its photolysis and MW irradiation at (a) 1590 MHz and (b) 94.5 MHz; *B*_1_ = 0.01 mT (solid circles) or 0.1 mT (open circles). (c) The predicted DNP stick-spectrum for semiquinone radical (B^•^QH). (d) The predicted SNP stick-spectrum for RP containing B^•^QH radical. Reprinted with permission from ref (546). Copyright 1986 Elsevier B.V.

In addition to radical pairs, microwaves inevitably affect radicals that, for instance, escape from the geminate RP. As a result, DNP-type effects are often observed,^89,535,546,547^ either alone or together with SNP. In the OE-DNP experiments in solution (section 3.2), stable paramagnetic species are used, and nuclear polarization is created on the nearby diamagnetic molecules. In contrast, MW irradiation of transient paramagnetic radicals produces polarization of their own nuclear spins which is preserved upon their chemical transformation to diamagnetic products. In fact, the cage-escape and F-pair products often show the combination of SNP and DNP effects^546^ (Figure 58).

**Figure 59:**
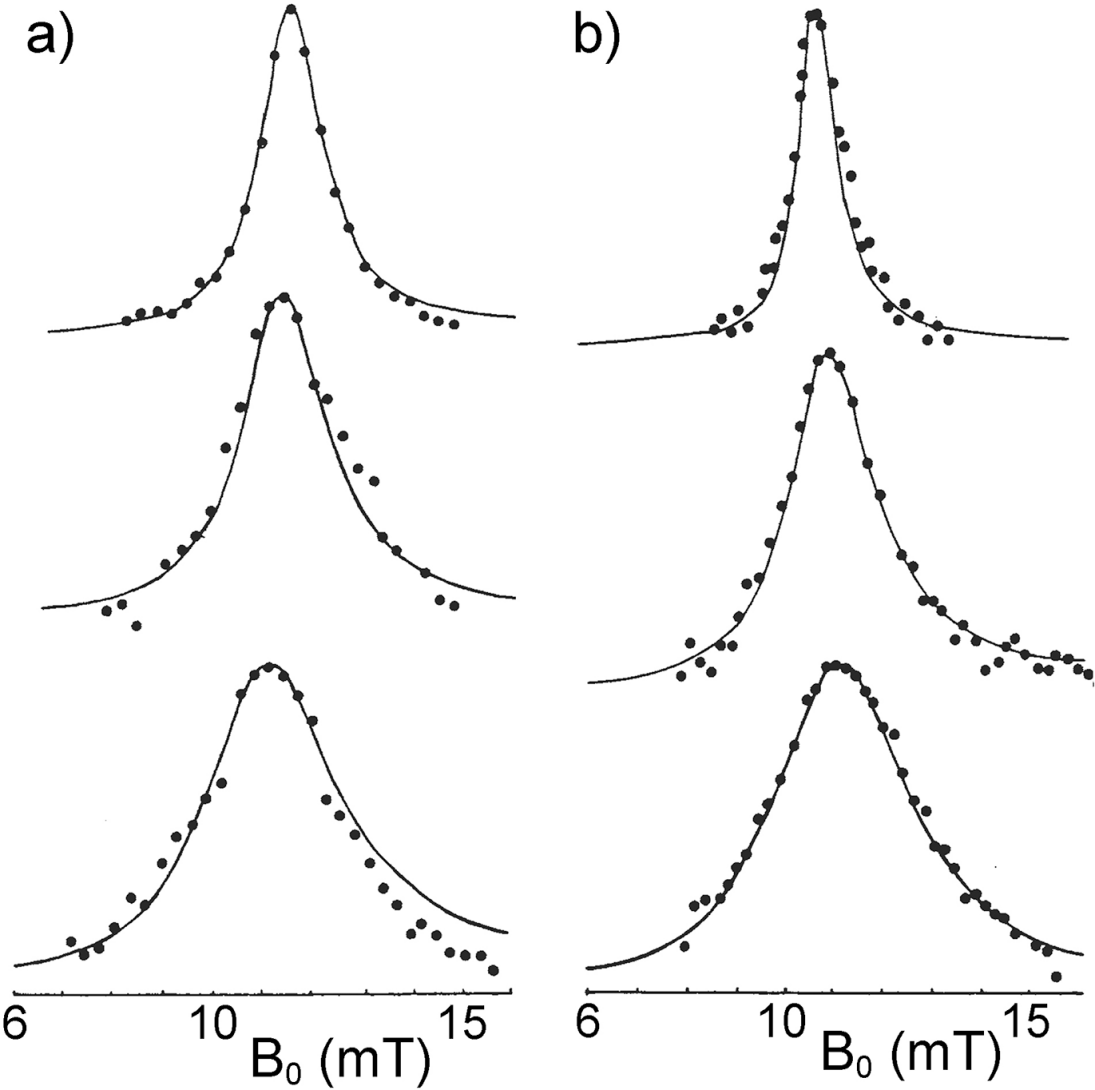
Dependence of DNP effects on *B*_0_ detected by monitoring the ^1^H NMR signal of dimethylaniline (DMA) aromatic protons upon photolysis of anthracene–DMA solutions using MW irradiation at 310 MHz. (a) The effect of DMA concentration at *B*_1_ = 0.3 mT (top: [DMA] = 0.5 M; middle: [DMA] = 0.22 M; bottom: [DMA] = 0.17 M). (b) The effect of MW field amplitude *B*_1_ at [DMA] = 0.8 M (top: *B*_1_ = 0.3 mT; middle: *B*_1_ = 0.7 mT; bottom: *B*_1_ = 1.2 mT). Reprinted with permission from ref (89), Springer Nature Customer Service Centre GmbH. Copyright 1990 Springer Nature.

DNP effects in radical-ions produced by photoinduced electron transfer between a donor (D) and an acceptor (A) were addressed in several studies.^89,547^ In such case, an essential role is played by the degenerate electron exchange processes of the type **D**^•+^ + D → **D** + D^•+^ and **A**^•–^ + A → **A** + A^•–^, which transfer the DNP-derived nuclear polarization (indicated by bold face) from radical-ions to diamagnetic species as well as govern the appearance of the observed dependence of DNP effect on *B*_0_ at constant MW frequency. A representative example demonstrating DNP effects in dimethylaniline radical cation is shown in Figure 59.

**Figure 60:**
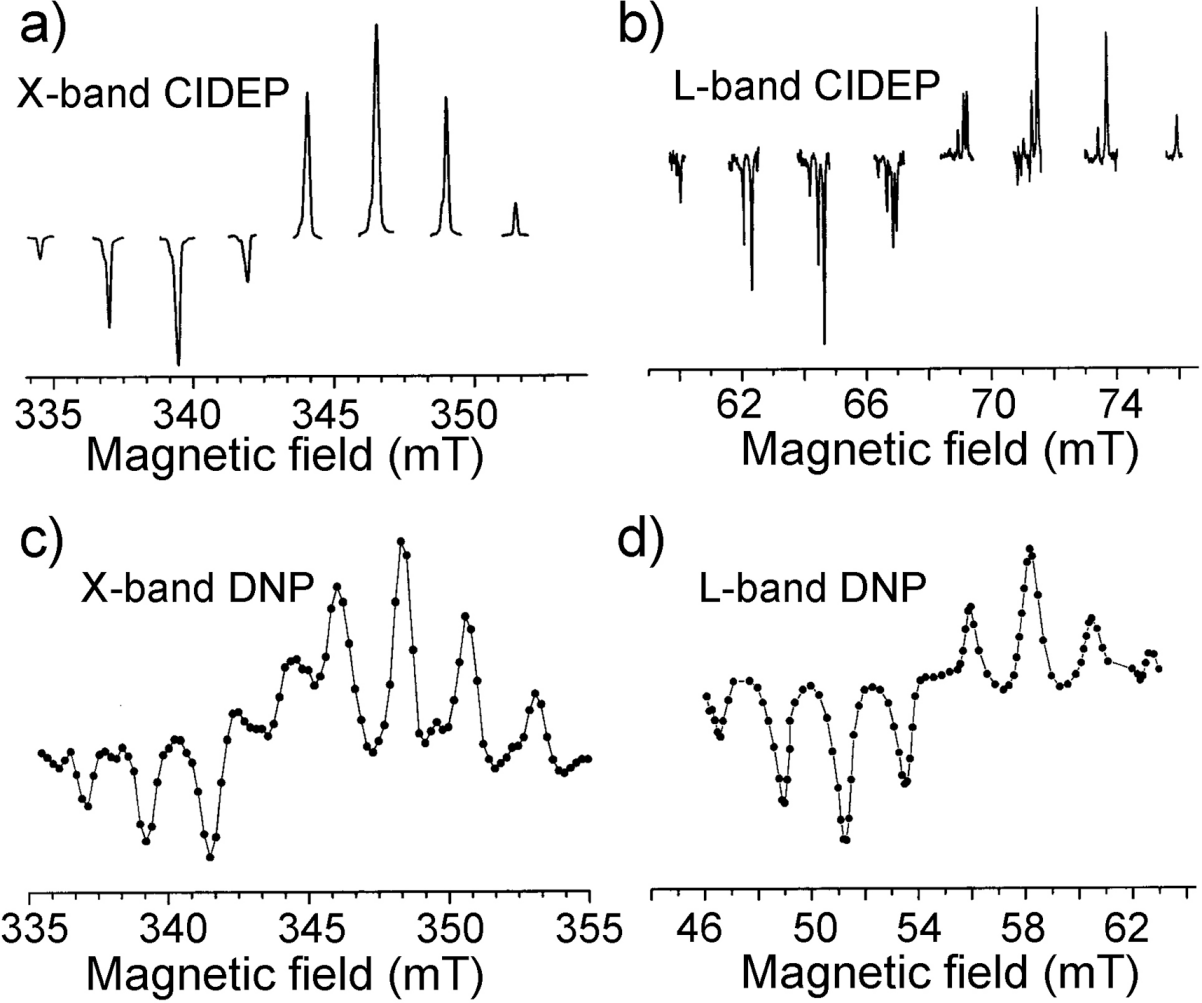
TR-EPR spectra and DNP effects detected upon photolysis of di-*t*-butyl ketone ((CH_3_)_3_C)_2_CO in benzene. (a) X-band (9.4 GHz) TR-EPR spectrum of *t*-butyl radical (CH_3_)_3_C^•^ integrated from 0.5 to 1.5 μs after the laser flash. (b) L-band (1.9 GHz) TR-EPR spectrum integrated from 1.5 to 2 μs. (c) X-band (9.33 GHz) and (d) L-band (1.53 GHz) DNP effects detected by monitoring the ^1^H NMR signal of the recombination product 2,2,3,3-tetramethylbutane (CH_3_)_3_C–C(CH_3_)_3_ with *B*_1_ = 0.2 mT. Reprinted with permission from ref (548). Copyright 1999 American Chemical Society.

Finally, several other effects should be mentioned in this context. The electron spins of radicals that escape from the primary RPs can be hyperpolarized owing to CIDEP effects (section 3.5). In such cases, electron–nuclear cross-relaxation can convert the hyperpolarization of electron spins to nuclear polarization even in the absence of any MW irradiation. Application of MW irradiation to such radicals before they relax to thermal equilibrium perturbs the polarized state of their electrons and thus affects the cross-relaxation process, leading to a decrease or even an increase of the resulting nuclear polarization.^548^ The sign and magnitude of such DNP effects reflect the corresponding CIDEP effects observed in TR-EPR spectra (Figure 60). Yet another interesting possibility considered earlier^549^ was the perturbation of nuclear spin state populations in transient radicals to alter the resulting nuclear polarization of the diamagnetic products, thereby accessing the NMR-detected nuclear resonance spectra of transient radicals. It is further noted that at magnetic fields comparable to HFC values of the radicals involved, an RF field induces mixed electron–nuclear spin transitions, which can also result in nuclear polarization of diamagnetic products.^550^

### 3.8 Photochemically Induced Dynamic Nuclear Polarization in the Solid Phase

Chemically induced dynamic nuclear polarization (CIDNP) can be induced thermally, as originally discovered,^451,551^ or photochemically^552^ (photo-CIDNP). CIDNP in the fluid phase was addressed in section 3.6, with one notable example being CIDNP in a cyclic photochemical reaction of a flavin dye and an aromatic amino acid in aqueous solution. In this section, we discuss the occurrence of CIDNP effects in the solid state, in combination with magic angle spinning NMR (photo-CIDNP MAS NMR), which has been recently reviewed.^553^

While DNP (sections 3.2, 3.3, and 3.4) relies on the presence of radicals, and ONP (section 3.9) is based on triplet states, CIDNP always requires the presence of radical pairs (RPs) possessing correlated electron spins. The concept of a spin-correlated RP was introduced earlier (sections 3.5 and 3.6).^428,429^ It is based on two organic radicals, R_1_ and R_2_, which form a common spin state: either a singlet or a triplet state. If these states of the radical pair are not its eigenstates, the RP might oscillate between these two collective spin states via their coherent superposition. As it can be rationalized in vector representation,^554^ the frequency of this oscillation depends on the difference in the g-factors of these two radicals (Δ*g*), with a large value of Δ*g* allowing for fast oscillations. In the radical pair mechanism (RPM)^86,555^ (sections 3.5 and 3.6), the oscillation between the spin states of a spin-correlated RP is modulated by the hyperfine interactions of unpaired electrons with magnetic nuclei such as protons. Depending on the nuclear spin state, such oscillation of the RP can be accelerated or slowed down.

Let us take the case that only one of the two radicals possesses one spin-1/2 nucleus, with the hyperfine splitting (given by the coupling constant A) being roughly twice the difference in the Zeeman frequencies of the two radicals (determined by Δ*g*) (see Figure 46). There are two possibilities, depending on the orientation of the nuclear spin. Assuming the Boltzmann-weighted distribution of the two nuclear spin orientations (i.e., n_α_:n_β_ ≈ 50:50), there is a 50% chance that the spin evolution of the RP is essentially stopped and the electron spin state of the RP is maintained. There is also a 50% chance that the oscillation of the electron spin state accelerates. Thus, if a spin-correlated RP is born in a triplet state, in one case it will stay in the triplet state, while in the other it will interconvert into a singlet state. The RP can only recombine in its singlet state, while in the triplet state its radicals might diffuse apart. This spin-sorting process (section 2.2.5) implies that nuclear spin states “decide” on the fate of a chemical reaction. This is an amazing result: nuclear spins, for which the energies of magnetic interactions are tiny compared to the enthalpies of chemical reactions, are able to control the kinetics of chemical transformations.^556^

In solid state, in contrast to most liquids, dipolar interactions are not averaged to zero and anisotropies occur. Thus, two new mechanisms that combine spin evolution and chemical reaction were proposed: *(1)* a fully coherent transformation of nuclear coherences to nuclear polarization called three-spin mixing (TSM).^557^ Here, during the free spin-evolution of the RP under the influence of the pseudosecular part of the anisotropic hyperfine interaction, electron coherence is converted into electron hyperpolarization which is transformed into nuclear hyperpolarization if a triple matching condition is fulfilled; *(2)* a coherent mechanism relying on the difference of the decay rates of the two spin states of the RP, called differential decay (DD).^558^ Since for the singlet-born RP in photosynthesis, the fraction of singlet RPs will exceed the fraction of triplet RPs (because the decay of the RP is much faster from its triplet state than from its singlet state), selective enrichment of particular nuclear spin states occurs. Both mechanisms exclusively occur in solid state and follow certain sign rules.^559^ Recently, in the context of explaining field-cycling MAS NMR data,^560^ the theory was reformulated and extended within the framework of level crossings and level anticrossings,^561,562^ which is also successfully applied to other hyperpolarization methods.

**Figure 61:**
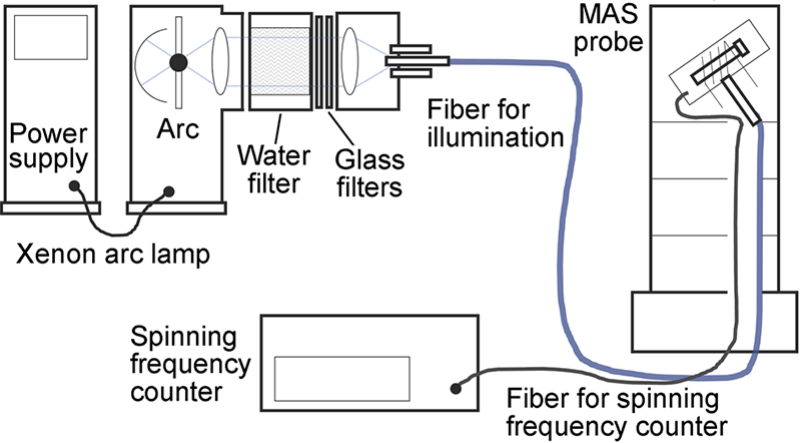
Setup for sample illumination under MAS NMR conditions. A 1000-W xenon lamp is used as a light source. A water filter and Schott glass filters remove infrared and ultraviolet radiation. A fiber bundle guides the light into the MAS NMR probe. The sample is located within a sapphire rotor and is laterally illuminated. Interference with the MAS frequency optical sensor has to be avoided.

Observation of the solid-state photo-CIDNP effect requires a solid-state NMR experiment. Therefore, magic angle spinning (MAS) of the sample is demanded. The sample is placed in an optically transparent rotor made from sapphire. Such sapphire rotors are presently available with 3.2, 4.0, and 7.0 mm diameters. Sapphire rotors can be used from cryogenic to ambient temperatures and are chemically inert. The bottom half is massive sapphire, while the sample occupies the upper half. Sapphire rotors are rather fragile. To avoid microscratches on their surface, the pneumatic sample lift should be avoided and the MAS frequency limited to, for example, 8 kHz for 4 mm rotors. Furthermore, an illumination setup is required for CIDNP experiments (Figure 61). In a typical setup, a 1000 W xenon lamp, emitting the full white spectrum from ultraviolet to near-infrared similar to sunlight, is used. Depending on the sensitivity of the sample, it might be necessary to block ultraviolet radiation, for example, using Schott glass filters. Against infrared radiation, a water filter might be a good choice. Alternatively, lasers can be used for sample illumination. Selecting the wavelength for monochromatic illumination, one should keep in mind that, for samples with high optical density, it is not the Lambert–Beer law that applies, but rather the Kubelka–Munk–Schrader theory.^563^ Therefore, it is advisable to choose a wavelength so that the light is able to penetrate deep into the sample. The MAS rotation allows illumination of the sample from all sides. One can expect that soon laser diodes will provide sufficient power, which may offer new illumination strategies. For the delivery of light into the MAS probe, fiber bundles with a heat-conducting metal husk at one end and a porcelain husk which does not affect the NMR experiments at the other end, are recommended. The stator has to have a hole which can be drilled into the tile carrying the coil. The coil might have to be modified so that it allows for sufficient irradiation of the rotor.

**Figure 62:**
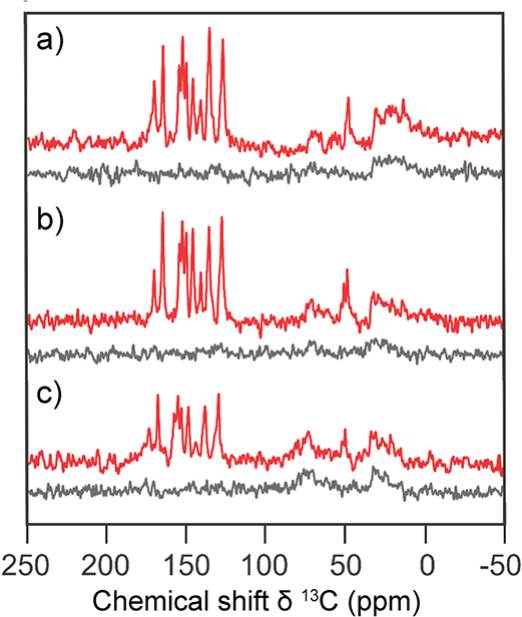
^13^C MAS NMR spectra of selectively ^13^C-labeled (a) photosystem II particle preparation, the so-called BBY preparation, (b) thylakoid membranes, and (c) entire plants of the aquatic plant *Spirodela oligorrhiza*, obtained under continuous illumination (red traces); also shown are the corresponding spectra obtained under dark conditions (gray traces). All samples were selectively labeled by feeding with 4-^13^C_1_-aminolevulinic acid (4-ALA). All spectra were obtained at a magnetic field of 4.7 T and a temperature of 235 K with a MAS frequency of 8 kHz and a recycle delay of 4 s. Reprinted from ref (565). Copyright 2018 The Authors. Published by Springer Nature under CC BY license.

Until now, two types of systems have been demonstrated to show the solid-state photo-CIDNP effect: photosynthetic reaction center (RC) proteins as well as some flavoproteins. The discovery of the solid-state photo-CIDNP effect in 1994 by Zysmilich and McDermott succeeded with a purple bacterial RC protein studied by ^15^N MAS NMR.^564^ To this end, the RC was isolated from the membrane, the electron-accepting quinones were chemically prereduced to allow for induction of cyclic electron transfer, and the sample was uniformly labeled with ^15^N isotope. While studying and optimizing the effect, it turned out that the NMR signal enhancement is so strong that enhanced NMR signals could be obtained also from photosynthetic membranes, cells and entire plants (Figure 62).^565^ The possibility to study such large units is very useful since, for many organisms, no procedure to isolate RC proteins is known. Therefore, sample preparation can be rather straightforward; however, cyclic electron transfer always needs to be introduced, for example by prereduction of the acceptor site. Isotope labeling, for example ^15^N labeling of bacteria, is rather straightforward, while introduction of ^13^C labels into plants is very demanding. Interestingly, also with a photosynthetic membrane under liquid-phase conditions, ^13^C photo-CIDNP signals were obtained, and even a ^13^C-^13^C COSY experiment was possible.^566^ In the slow-tumbling regime, the large anisotropic electron–nuclear interactions are preserved over the time period required for polarization transfer, as in solids, and therefore the “solid-state” photo-CIDNP effect can occur.

The other systems showing the solid-state photo-CIDNP effect, as discovered in 2010,^567^ are specific flavoproteins such as light-oxygen-voltage-sensing (LOV) domains. These proteins need to be mutated since the photochemically active cysteine, forming a covalent bond to flavin, has to be removed to allow for cyclic electron transfer and formation of an RP.^568^

**Figure 63:**
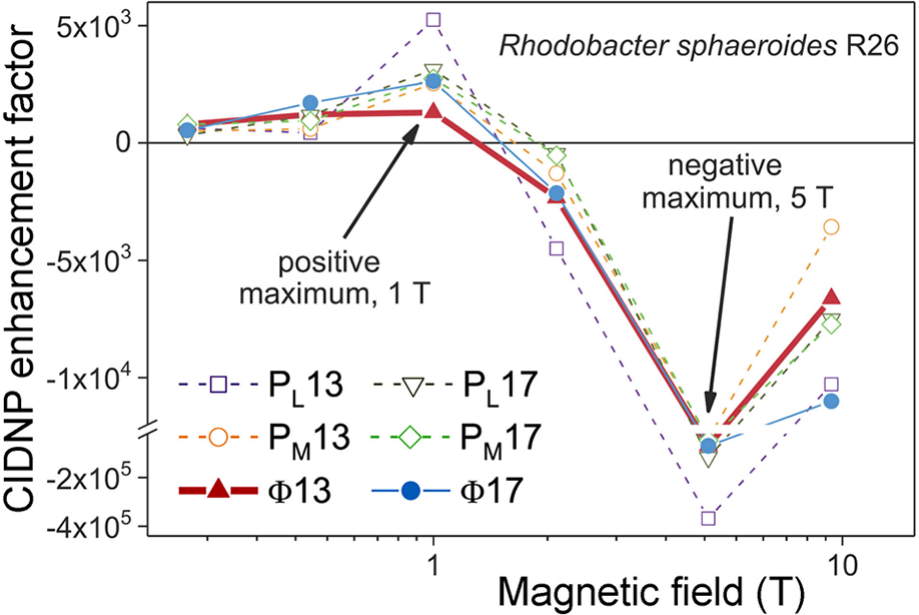
Experimental field dependence of the solid-state photo-CIDNP effect in the photosynthetic reaction center of the purple bacterium *Rhodobacter sphaeroides* R26. The field dependence of the effect for selected carbons ^13^C of the “special pair” donor cofactors P_L_ and P_M_ as well as of the bacteriopheophytin acceptor Φ is shown. The notation “P_L_13”, e.g., refers to carbon 13 on cofactor P_L_ according to the IUPAC numbering. Reprinted from ref (562) with the permission of AIP.

The achievable polarization depends strongly on the strength of the external magnetic field (Figure 63).^560−562,569^ There is apparently an enhancement maximum in the range of typical NMR fields. A low-field maximum, attributed to S-T_–_ mixing in liquid-state NMR (section 3.6),^570^ is predicted by theory but has not been demonstrated experimentally.

**Figure 64:**
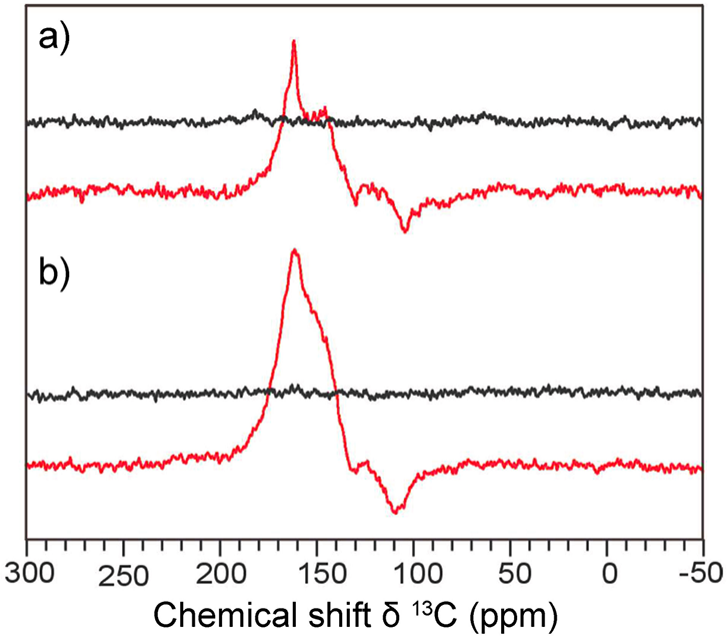
Solid-state photo-CIDNP effect in the photosynthetic reaction center of the purple bacterium *Rhodobacter sphaeroides* R26. The ^13^C MAS NMR spectra are obtained at (a) 2.4 T (100 MHz ^1^H frequency), and (b) 1.4 T (60 MHz ^1^H frequency). Photo-CIDNP MAS NMR spectra (red traces) are acquired under continuous illumination with a white light of xenon lamp and without proton decoupling. The corresponding spectra obtained without illumination are shown for comparison (black traces). Enhancement factors are (a) 20,000 and (b) 80,000. Adapted with permission from ref (569). Copyright 2012 American Chemical Society.

Photo-CIDNP MAS NMR experiments allow for direct measurement of the enhancement factor by comparing the spectra obtained with and without illumination under otherwise the same conditions. Often the “dark” signal of the methyl groups of the protein is taken to obtain an absolute scale. These experiments have shown enhancement factors of more than 80,000 for ^13^C MAS NMR under continuous illumination (Figure 64).^569^ Such nuclear spin order close to unity is obtained by building up hyperpolarization during several photocycles.

As mentioned above, samples are either photosynthetic RC proteins or flavoproteins. Despite several trials, photo-CIDNP experiments with artificial photosynthetic systems did not succeed. On the other hand, all natural photosynthetic systems that have been tested show the CIDNP effect.

Photo-CIDNP MAS NMR has been done with two motivations. On one hand, the aim was to resolve the exact mechanism of the solid-state photo-CIDNP effect. To this end, mainly time-resolved laser-flash experiments^571^ as well as magnetic field dependence studies^560−562^ were performed. On the basis of the understanding of the effect, photo-CIDNP MAS NMR was developed into an analytical method allowing one to study electronic structures, kinetics and molecular dynamics of RPs.^572^

On the other hand, photo-CIDNP MAS NMR has always been used to learn about the fundamental processes in natural photosynthesis and efficient electron transfer in nature. Even without understanding the origin of the solid-state photo-CIDNP effect, NMR chemical shifts are directly available to obtain information on the ground electronic state of electron-pumping photochemical machinery of RCs. Of particular interest is the RC of photosystem II of plants, having the highest oxidizing force in living nature, able to oxidize water to molecular oxygen. Here, a monomeric chlorophyll cofactor appears as donor, not a dimer as in various other RCs. It turned out that the donor chlorophyll carries a strongly asymmetric electron spin density^573^ which is spread to the axial histidine leading to the so-called hinge-model.^574,575^

Another long-standing question is the difference in the symmetry of the light-induced electron transfer between bacterial RCs of the purple bacterium *Rhodobacter sphaeroides* and photosystem I of plants. In purple bacterial RCs, both cofactors are forming the electron donor, the so-called “special pair”, however, the electron is transferred selectively into one of the two cofactor branches.^576^ On the other hand, in photosystem I, no functional symmetry breaking occurs and both branches of cofactors are active. Chemical shifts of the two cofactors in the “special pair” are rather different;^577^ however, the differences are not much larger than those observed in the donor dimer of photosystem I.^578^ Therefore, the ground-state electronic structure does not appear to be the decisive factor. Interestingly, there is a large asymmetry in the electronically excited state of the donor molecules in bacterial RCs, as shown by the analysis of the signature of the donor triplet state on the solid-state photo-CIDNP effect,^569^ implying that orbital factors related to orbital overlap and therefore electron-transfer kinetics are very different between the two cofactors. Hence, it appears that orbital overlap is a key factor deciding on the electron transfer pathway.

Two forms of the ancient heliobacteria, which might be the oldest survivors of the period of early photosynthesis, have been studied. The protein structure of heliobacterial RCs is highly symmetrical;^579^ however, an oxygenated product can be formed showing a dramatic color change of the sample from brown to green, and no intermediate is reported. In both forms, called Braunstoff and Grünstoff, the solid-state photo-CIDNP effect was observed and the transformation of both forms studied, providing evidence that spin-correlated RPs occur in both forms of preparation.^580^

Diatoms (Greek: dia = through, tomos = knife: i.e., “the cuttable” because cell proliferation has been studied on these systems), a major form of algae living in the ocean, also have two RCs like plants, photosystems I and II, and were studied with photo-CIDNP MAS NMR.^581^ Comparing photosynthetic RCs of three types of diatoms, chemical shifts similar to those in RCs of plants were observed, suggesting a similar ground-state electronic structure. It remains puzzling that the magnetic field dependence of the ^13^C solid-state photo-CIDNP effect in photosystem II of the diatom *Phaeodactylum tricornutum* is different from that in plants.

**Figure 65:**
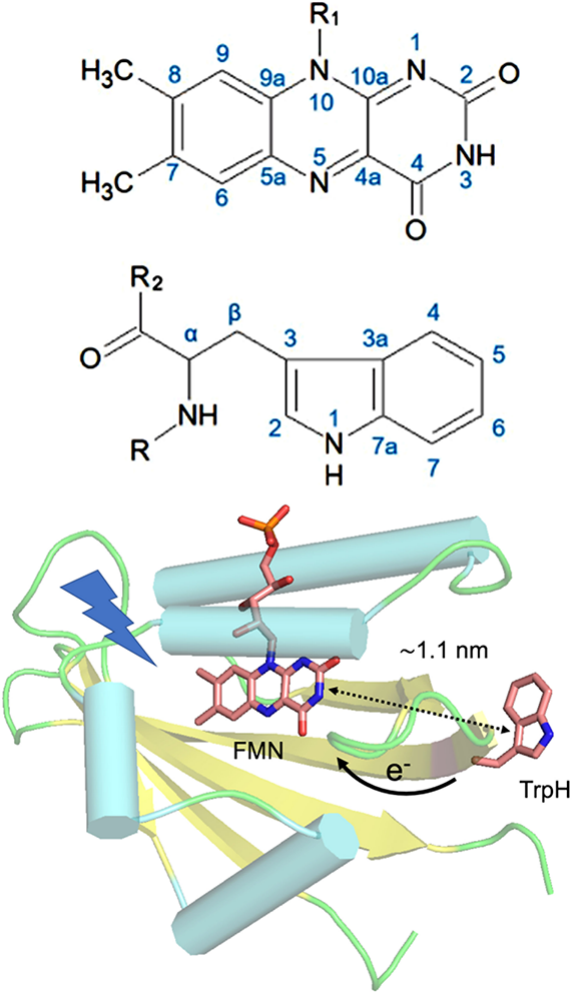
Crystal structure of the flavoprotein phototropin-LOV1 wild type in the dark with 0.19 nm resolution (Protein Data Bank reference: 1N9L). The edge-to-edge distance between the flavin mononucleotide (FMN) cofactor and tryptophan (Trp) is around 1.1 nm. Note that electron transfer from tryptophan (at position 98) to FMN after photoexcitation occurs only when the conserved cysteine close to FMN is mutated to serine or alanine. The IUPAC numbering of FMN and tryptophan is included. Reprinted from ref (568). Copyright 2019 The Authors. Published by Springer Nature under CC BY license.

The discovery of the solid-state photo-CIDNP effect in flavoproteins^567^ opened a new world of experiments, in particular because in these systems the electron donor is an aromatic amino acid which, using side-directed mutagenesis, can be shifted to other positions in the protein, varying distance, orientation and strength of couplings (Figure 65).^568,582^ Presently, understanding of the photo-CIDNP mechanism in flavoproteins is required. It must be distinguished from that in photosynthetic RCs, in which the RP is born in a singlet state, while in flavoproteins the electron transfer is initiated by the triplet state of the flavin cofactor.

**Figure 66:**
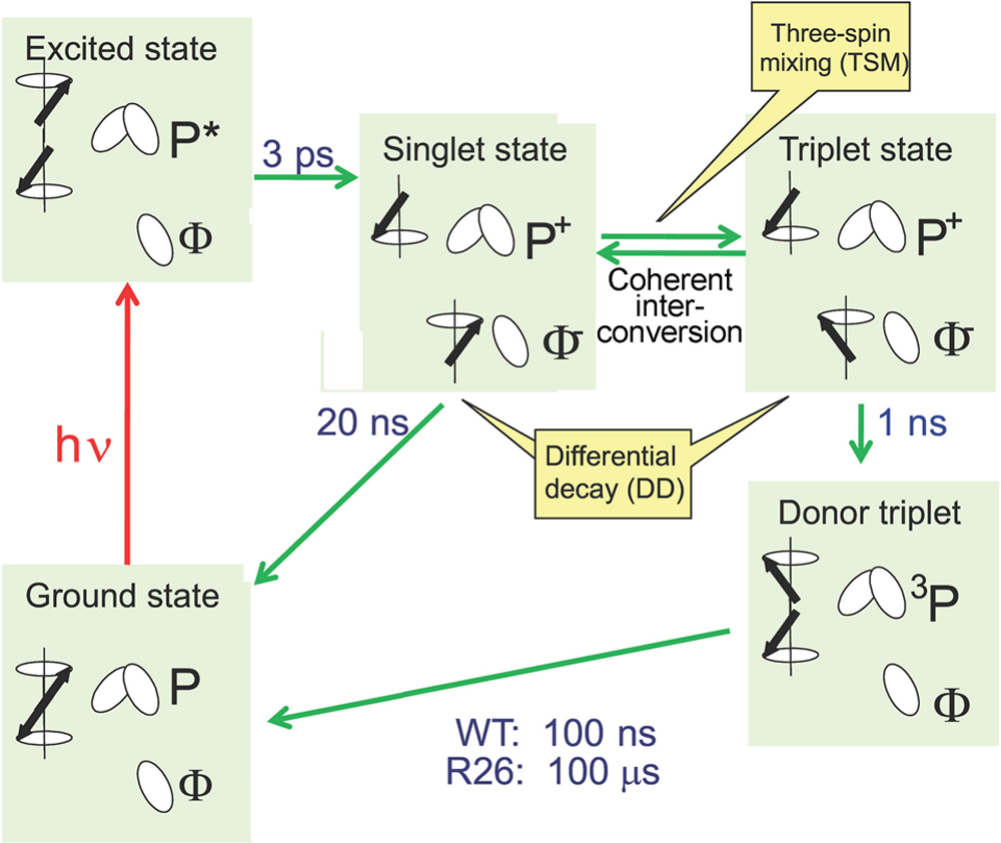
Photocycle of quinone-blocked bacterial reaction center of *Rhodobacter sphaeroides*. Upon photon absorption, the primary electron donor P, bacteriochlorophyll dimer (the “special pair”) is excited to its first excited electronic singlet state. In the excited state, fast electron transfer to the bacteriopheophytin acceptor Φ forms the spin-correlated RP in its singlet state. Since the forward reaction is prevented, the RP can recombine to its electronic ground state. Alternatively, in a coherent interconversion of the electron spin multiplicity, the triplet state is formed. The triplet state of the RP can either convert back to the singlet state or form a donor triplet state, from which a slow back-reaction to the ground-state is possible. The kinetics of this process is given for wild type (WT) and for R26 reaction centers. R26 is a mutant without the carotenoid cofactor.

The photocycle of a standard photo-CIDNP experiment is shown in Figure 66. Using nanosecond laser pulses, time-resolved photo-CIDNP MAS NMR experiments allowed to elucidate the kinetics and mechanism occurring in photosynthetic RCs.^571^ In addition, the initial signal intensity in time-resolved photo-CIDNP MAS NMR refers to the enhancement due to the RPM and can be related to the isotropic hyperfine coupling constants. While the RP recombines and the sample becomes diamagnetic, the nuclear hyperpolarization remains and decays with the normal nuclear spin relaxation kinetics governed by *T*_1n_. Hence, during NMR detection, there are no unpaired electron spins remaining, and thus no paramagnetic broadening of the NMR lines. Furthermore, in selectively ^13^C-labeled samples, spin diffusion between different nuclear positions can nicely be followed. Recently, it was possible to transfer the ^13^C and ^15^N hyperpolarization of the cofactor to the nearby protons within the protein pocket.^583^ These “spin-torch” experiments are opening the possibility to study not only the molecules forming the RP but also their direct environment.

Presently, there are efforts toward developing photo-CIDNP MRI. Ideally, one would install solid-state photo-CIDNP agents, such as proteins or artificial dyads, capable to bind to selective surfaces and observe these selectively labeled surfaces with MRI methodology. It would be useful to incorporate the know-how used in fluorescence spectroscopy by using agents such as green fluorescent protein (GFP) to label particular biological surfaces.

Finally, there is no fundamental reason why artificial systems could not show the photo-CIDNP effect. Hence, one would expect that future attempts will eventually lead to success in such studies. Furthermore, the occurrence of the solid-state photo-CIDNP effect is apparently affected by the isotopic labeling pattern. Here, a systematic study would be desirable.

### 3.9 Optical Nuclear Polarization and Triplet Dynamic Nuclear Polarization in Molecular Crystals

**Figure 67:**
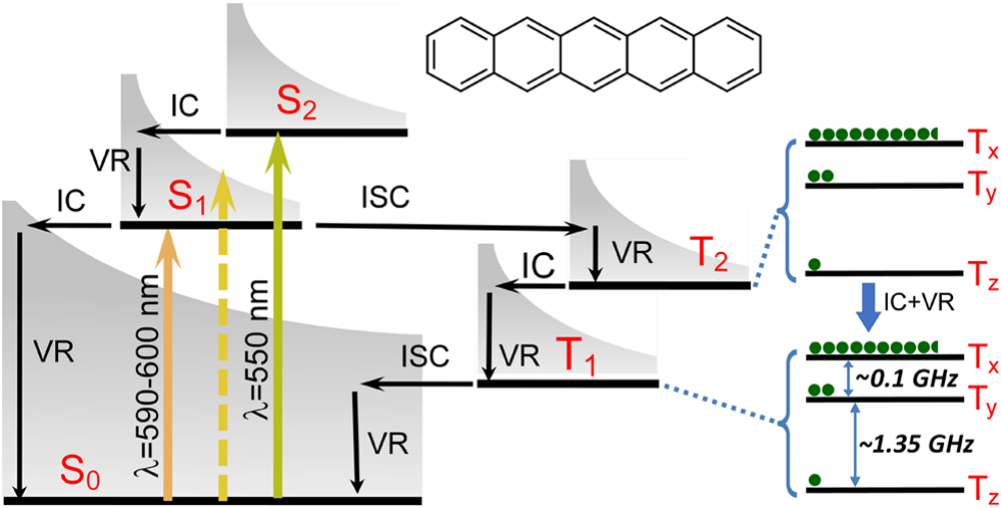
Jablonski diagram for pentacene. S_0_ is the ground electronic state. Excited singlet states S_n_ can be reached by photoexcitaiton at an appropriate wavelength; other radiative transitions are not shown. Shaded patterns schematically show vibrational energy sublevels. Isoenergetic ISC processes populate and depopulate the three spin sublevels T_*x*_, T_*y*_, T_*z*_ of the molecular triplet states T_n_ at different rates. IC - internal conversion; VR - vibrational relaxation.

The optical nuclear polarization (ONP) and triplet dynamic nuclear polarization (*t*DNP; a.k.a. MW-induced ONP, MI-ONP; RF-induced ONP, RF-ONP; Hartmann–Hahn ONP, HH-ONP) techniques^584−586^ utilize strong polarization of electron spins of the lowest triplet state T_1_ of a suitable guest chromophore molecule (or sometimes a triplet radical pair) in a host matrix produced upon photoexcitation of the guest by spin-selective intersystem crossing (ISC) between its singlet and triplet manifolds (section 2.2.2) (Figure 67). The subsequent transfer of polarization from electrons to nuclei during the lifetime of the polarized T_1_ state can be either spontaneous (in ONP) or induced by a microwave (MW) or radiofrequency (RF) field (in *t*DNP). As the ISC from T_1_ back to the ground diamagnetic S_0_ state does not change the nuclear spin states, the initial nuclear polarization of the guests or their immediate neighbors is preserved and then spreads over the network of nuclei in a solid sample by spin diffusion. This is facilitated by the absence of a diffusion barrier (section 3.3) and the long nuclear spin relaxation times *T*_1n_ in a diamagnetic solid since the photogenerated paramagnetic species are short-lived. In contrast to *d*DNP (section 3.4) and MAS DNP NMR (section 3.3) techniques, *t*DNP can be achieved without resorting to cryogenic temperatures and/or high magnetic fields.

**Figure 68:**
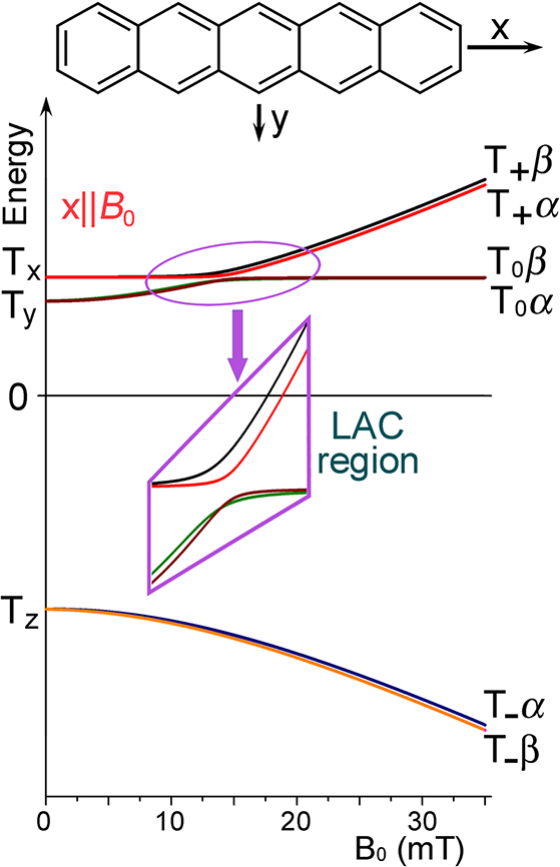
Energy levels for triplet pentacene with its molecular *x* axis oriented parallel to magnetic field *B*_0_ are shown schematically (only one nuclear spin is taken into account).

In most cases, coherent polarization transfer from electrons to nuclei is mediated by mixing of states by the static hyperfine interaction (HFI) between electron and nuclear spins. In ONP, this is efficient only in the vicinity of an avoided energy level crossing (LAC) (Figure 68). At other *B*_0_ fields, application of a MW or RF field is essential. In the solid effect (sections 3.3 and 3.4), the oscillating (*B*_1e_) field drives the nominally “forbidden” flip-flip (Δ*S*_*z*_ + Δ*I*_*z*_ = ±2) and flip-flop (Δ*S*_*z*_ + Δ*I*_*z*_ = 0) mixed electron–nuclear spin transitions which are partially allowed by HFI. However, when the EPR line width exceeds nuclear Larmor frequency, the polarization transfer is inefficient as concurrent flip-flip and flip-flop transitions lead to opposing nuclear polarization (differential solid effect). More efficient schemes are based on driving the allowed electron spin transitions in combination with electron–nuclear cross-polarization under condition similar to Hartmann–Hahn matching (see below).

The Overhauser-type incoherent transfer driven by cross-relaxation is also possible, for instance when energy transfer from a photoexcited guest to a host matrix produces mobile triplet excitons with modulated HFI. Several transfer mechanisms can be operative simultaneously and often destructively. For instance, all three mechanisms (Overhauser, ONP, and *t*DNP) were observed in doped anthracene crystals.^587^ In the case of coherent polarization transfer, oscillations in nuclear polarization can occur under appropriate conditions, particularly for high *B*_1_ and moderate *B*_0_ fields.^588−590^ Coherence transfer pathways have been analyzed in some detail.^589^

Photoexcitation of a chromophore molecule requires a suitable light source, preferably a pulsed laser. For instance, the most efficient photoexcitation of the pentacene guest in naphthalene or *p*-terphenyl host crystals is from the S_0_ to S_1_ state at 600 and 590 nm, respectively (Figure 67). Higher excitation energies can be used if the host crystal is transparent at the chosen wavelength (e.g., naphthalene at 337 nm of a pulsed N_2_ laser), but light absorption may be less efficient, and the excess energy is released as heat, changing the sample temperature and damaging its crystalline structure. While electron spin polarization is independent of magnetic field, in low magnetic fields the anisotropic HFI of the triplet electronic spin state required for polarization transfer is greatly reduced in first order when the electron Zeeman energy is significantly below those of the zero-field transitions.^591^ Therefore, a suitable magnet is required. The *t*DNP experiments additionally require a (pulsed) MW or RF source. Many experiments use the widely available X-band (9–12 GHz) EPR equipment,^592^ but several studies were performed either at much lower frequencies or at ∼18 GHz.^593−595^ Experiments have been performed from cryogenic (4 K and above) to room temperature (RT),^592^ with lower temperatures often providing higher polarization levels. NMR signal detection is performed either in situ or by shuttling the sample to a dedicated NMR probe either within the same or in a separate magnet. Attaining the highest polarizations requires capabilities to sweep magnetic field or MW/RF frequency (see below), and to carefully control crystal orientation with respect to the *B*_0_ field.

**Figure 69:**
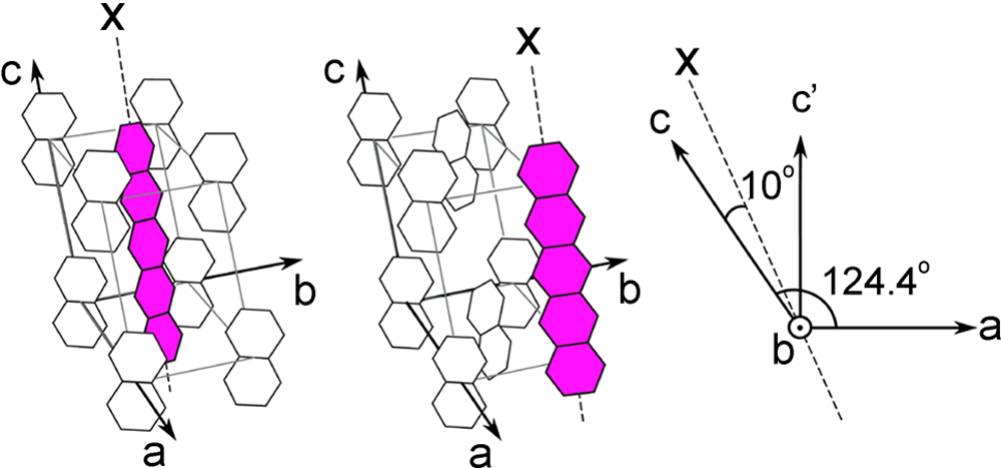
Crystal structure of pure naphthalene is monoclinic with a unit cell with axes *a* ≈ 0.81 nm, *b* ≈ 0.59 nm, *c* ≈ 0.86 nm, and β = 124.4°. The a–b plane is the cleavage plane. Two naphthalene molecules can be replaced at two different positions in the crystal by one pentacene molecule whose *x*-axis lies in the *a*–*c* plane at an angle of 10° to the *c*-axis. Reprinted with permission from ref (592). Copyright 2013 Elsevier.

The *t*DNP technique has so far been demonstrated with a rather limited range of chromophore guest molecules and host matrices. A single crystal of naphthalene doped with pentacene is one of the most efficient and widely used systems for ONP/*t*DNP, with pentacene substituting two naphthalene molecules in the lattice without perturbing the rest of the crystal (Figure 69). For room temperature experiments, *p*-terphenyl is a better host as it allows higher dopant concentrations facilitating polarization buildup, and is much less prone to sublimation. Dopant concentration (∼10–1000 ppm) determines the uniformity of light absorption in the sample; clustering of chromophore molecules can, for instance, cause quenching of the triplet state. Good-quality doped single crystals are often grown using the conventional Bridgman method. A number of experiments were also reported in which, contrary to the initial misinterpretation, the guest (e.g., anthracene, acridine, phenazine) photochemically abstracts an H atom from the host (e.g., fluorene, anthracene) to produce a triplet radical pair.^587,596^

A highly spin-selective ISC from the S_1_ state to the triplet manifold of pentacene upon its photoexcitation in *p*-terphenyl host (Figure 67) populates the three triplet sublevels T_*x*_, T_*y*_, T_*z*_ in the reference frame of the zero-field-splitting (ZFS) tensor as *P*_*x*_:*P*_*y*_:*P*_*z*_ = 0.76:0.16:0.08.^586^ The magnitude and sign of the observable electron polarization in the lab frame of reference largely depends on the orientation of the chromophore molecule with respect to *B*_0_. For pentacene in the x||*B*_0_ orientation (Figure 68), the populations of the T_+_, T_0_, and T_–_ states are *P*_+_:*P*_0_:*P*_–_ = 0.12:0.76:0.12, maximizing the population difference for the T_+_-T_0_ pair at 73%. Even larger electron polarizations can be achieved for pentacene in naphthalene host,^597^ whereas more air-tolerant 6,13-diazapentacene and 5,12-diazatetracene in *p*-terphenyl matrix yielded 49% and 66% electron polarization, respectively, at RT and *B*_0_ ∼ 0.676 T.^598^ Other orientations give lower usable polarizations. For pentacene, *T*_1e_ ∼ 100 μs is longer than the lifetime of its T_1_ state (∼20 μs for T_0_, ∼ 80 μs for T_+_ and T_–_) and thus has little effect on electron polarization. However, because the reverse ISC from T_1_ to S_0_ is also spin-selective, photoexcitation with a continuous-wave (CW) source or long laser pulses leads to considerably lower time-averaged electron polarizations (e.g., max. ∼ 12% for pentacene/naphthalene^599^).

In ONP, spontaneous polarization transfer to nuclei is efficient when the *B*_0_ field is set to achieve conditions close to a LAC (Figure 68). The optimum field value thus depends on the exact ZFS parameters and crystalline sample orientation with respect to *B*_0_. Rapid *B*_0_ switching away from the LAC after a laser pulse can increase polarization levels.^600^

**Figure 70:**
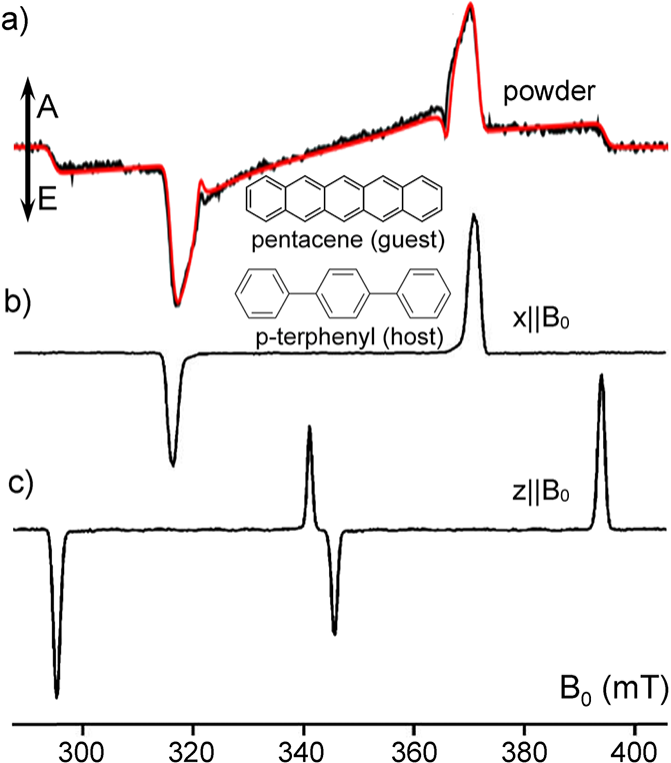
(a) An experimental time-resolved EPR spectrum (black trace) of a powder sample of pentacene in *p*-terphenyl host at room temperature after photoexcitation at 590 nm, and its simulation (red trace). A - enhanced absorption, E - emission. (b,c) Corresponding EPR spectra of a single crystal for *x*||*B*_0_ (b) and *z*||*B*_0_ (c) orientations. Two pairs of EPR lines observed in (c) correspond to two different lattice sites occupied by pentacene in the host crystal. Spectra (a–c) are not shown on the same vertical scale. (a) Adapted with permission from ref (601). Copyright 2016 American Chemical Society. (b,c) Reprinted from ref (602) with the permission of AIP Publishing.

In *t*DNP, *B*_0_ is usually chosen to set the system away from a LAC, and the transfer of electron polarization to nuclei is mediated by application of a MW (or RF) field. Since EPR spectra are broadened inhomogeneously^601,602^ (Figure 70), CW MW irradiation affects only a small fraction of electron spins and is thus less efficient compared to pulsed MW irradiation. In the pulsed scheme termed NOVEL (nuclear orientation via electron spin locking),^603,604^ the electron spin magnetization is locked in the rotating frame. The value of the locking field *B*_1e_ is chosen to match the Rabi (nutation) frequency of an electron in the rotating frame to the Larmor frequency of the nuclei in the lab frame^604,605^ (modified Hartmann–Hahn condition, √2γ_e_*B*_1e_ = γ_n_*B*_0_). We note that NOVEL is also applied in some studies to generate nuclear spin hyperpolarization using color centers in diamond (section 3.10). An even more efficient scheme termed integrated solid effect (ISE) implements the matching condition for a much larger fraction of electron spins by an adiabatic fast passage through the inhomogeneously broadened EPR line. This is achieved by sweeping the *B*_0_ field or MW frequency during a single MW pulse, e.g., an 8–20 μs sweep over a 5–20 mT range for pentacene with 20–90 μs lifetime of its T_1_ state.^606^ The polarization transfer from electrons to nuclei at the matching condition is fast, on the order of a few hundred ns.

**Figure 71:**
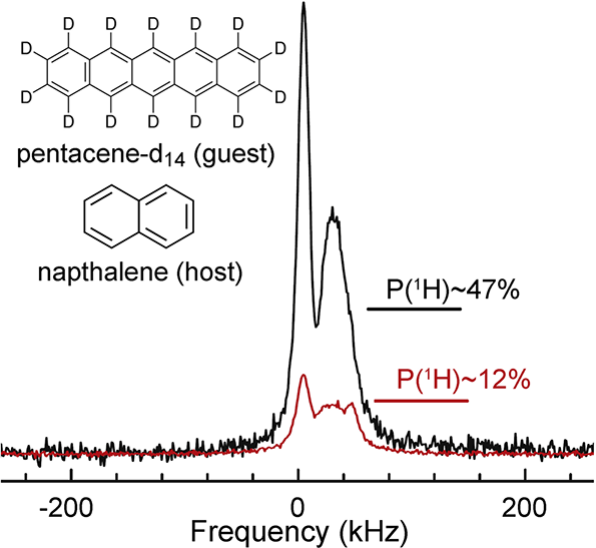
Enhanced ^1^H NMR signals of a naphthalene crystal doped with pentacene-*d*_14_ taken at different times during the ISE polarization build-up at 0.3 T and *T* ∼ 100 K. Reprinted from ref (592), Copyright (2013), with permission from Elsevier.

Nuclear polarization produced by a single polarization cycle is rather low, in particular because the yield of S → T ISC can be rather low, e.g., 2–3% for pentacene at low temperatures (but possibly larger at RT^603^). A laser pulse much longer than the S_1_ state lifetime achieves repetitive S_0_ → S_1_ re-excitations, accumulating electron population in the T_1_ state. For pentacene, the S_1_ state lifetime is ∼20 ns, and 800–1000 ns laser pulses were shown to be efficient.^607^ However, illumination should be shorter than the relaxation and decay times of the T_1_ state, otherwise its polarization is degraded. Furthermore, multiple repetitions of the entire polarization cycle at a rate of up to several kHz^592^ can be used to accumulate much higher polarization levels within the *T*_1n_ time of a diamagnetic sample (Figure 71). For naphthalene host at ∼0.3 T, *T*_1_(^1^H) ∼ 40 min at RT in the absence of light, and ∼1000 min at 77 K even under pulsed illumination.^608^

**Figure 72:**
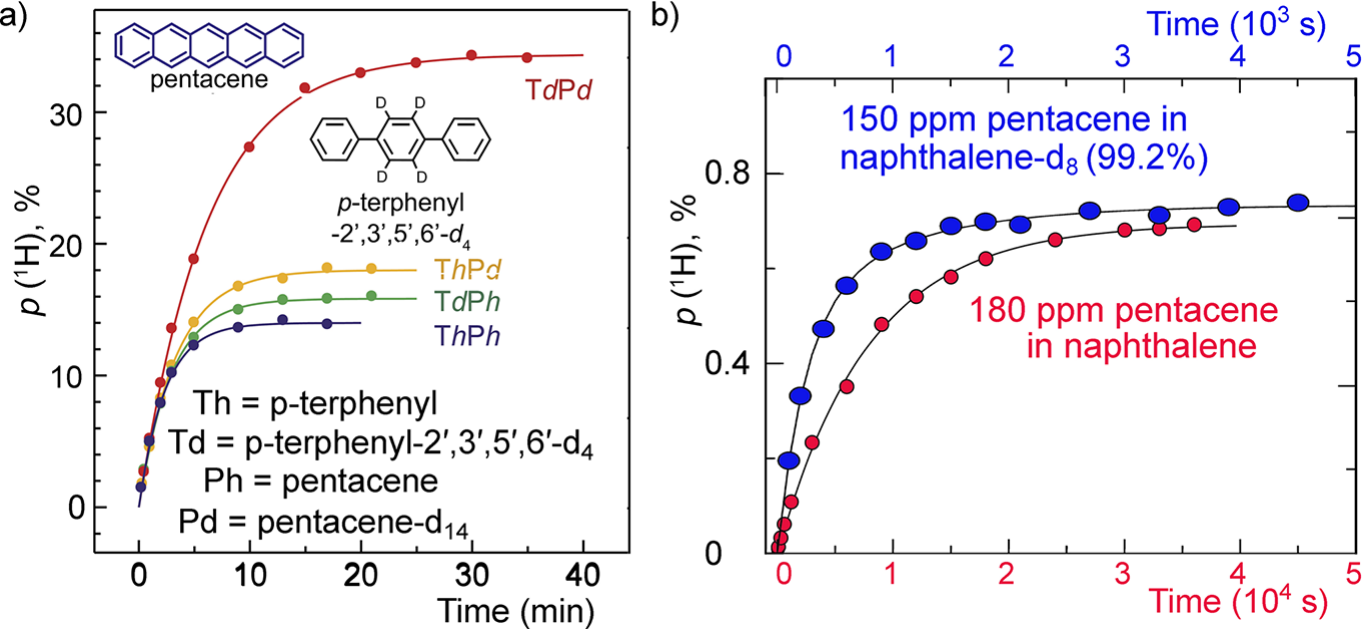
(a) Effect of deuteration of components on polarization for pentacene in *p*-terphenyl; experiments at RT and *B*_0_ = 0.4 T. (b) Effect of matrix deuteration on the polarization buildup times (357 s vs 7890 s) for pentacene in a naphthalene crystal; note the difference in time scales in the graph. Experiments were performed at 105 K and *B*_0_ = 0.3187 T. (a) Reproduced with permission from ref (610). (b) Adapted with permission from ref (607) and ref (609). Copyright 2004 The Physical Society of Japan.

The nuclear polarization build-up time depends on the sample and the experimental conditions; values ranging from a few minutes to several hours have been observed. It can be shortened dramatically using partial deuteration of the sample to reduce the size of the pool of polarizable nuclei. This also tends to increase *T*_1n_ and usually yields higher polarization levels. For instance, for pentacene-naphthalene at 105 K, deuteration of the host (∼99%) resulted in *T*_1n_ ∼ 10^5^ s for residual protons and a dramatic shortening of the build-up time from ∼(1–2) × 10^4^ s down to 357 s^609^ (Figure 72).

Achieving maximum efficiency of nuclear polarization thus requires a careful optimization of numerous parameters. For the pentacene/naphthalene single crystal, the highest bulk ^1^H spin polarizations achieved were ∼70% at 105 K^607,609,611^ and ∼80% at 25 K;^612^ for a doped *p*-terphenyl crystal at RT, polarization of ∼34% was achieved.^610^ Cross-polarization (CP) and field cycling can be used to transfer polarization from ^1^H to other nuclei such as ^13^C^610,613^ and ^19^F.^611^

Besides the studies of their principles and practical aspects, ONP and *t*DNP were used to perform time-resolved spectroscopy of excited triplet states in molecular solids and enhanced NMR of molecular crystals. For instance, the ONP/CP combination was applied to evaluate the ^13^C CSA tensors for all carbon atoms of fluorene doped with acridine.^614^*t*DNP scheme can be also used for optically detected NMR and nuclear quadrupole resonance (NQR) experiments.^615,616^

In addition, application of *t*DNP in nuclear physics for experiments with radioactive nuclear beams and for spin filtering/polarization and small-angle scattering of polarized neutron beams was pursued systematically. For naphthalene doped with pentacene-*d*_14_, *T*_1n_ ∼ 920 h at 0.5 T and 25 K, and ∼800 h at 20 mT and 6 K, and a polarization level of ∼80% were achieved.^612^ Such extremely long relaxation times allow one to polarize the sample under optimum conditions and then transport it to a neutron beamline and use it for several days with little polarization loss. As an example of applications which are potentially interesting for quantum information processing, a multiqubit entanglement in the system of 14 strongly polarized ^1^H spins at 295 K was demonstrated with the pentacene/*p*-terphenyl single crystal.^617^

Compared to oriented single crystals, the EPR spectra of polycrystalline samples are broad superpositions of contributions from differently oriented triplet molecules (Figure 70). Polarization transfer to nuclei is still possible by limiting the field/frequency sweep to the most intense parts of an EPR spectrum corresponding to the most favorable orientations. However, the efficiency is significantly reduced as all triplet guests shorten *T*_1n_ while only their fraction contribute to nuclear polarization. For polycrystalline sample of pentacene/naphthalene-*d*_8_, this yielded 1–3% polarization^618^ as opposed to ∼70% achieved with a single crystal. Mechanical grinding of single crystals can measurably reduce *T*_1n_, from ∼29,000 to 4000 s, likely by creating free radicals. For pentacene in benzophenone or *o*-terphenyl glass, polarization achieved at 120 K was 1.45%.^611^ Modest ^1^H NMR signal enhancements were achieved for some other polycrystalline^595^ and glassy^593^ samples

**Figure 73:**
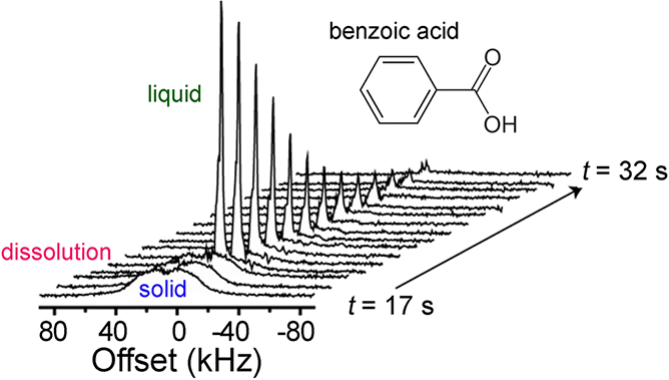
Dissolution *t*DNP performed by injecting a hot aqueous solution to the sample tube with a polarized pentacene/benzoic acid sample. The injection was performed 20 s after polarizing the powdered sample by *t*DNP for 10 min. The ^1^H magnetization was measured with 15° flip-angle pulses repeatedly at intervals of 1 s; *t* = 0 corresponds to the end of *t*DNP procedure. Reprinted with permission from ref (619). Copyright 2018 American Chemical Society.

Recent efforts include attempts to utilize *t*DNP in the liquid phase. For instance, polycrystalline benzoic acid doped with pentacene (with one or both components isotopically labeled) polarized at *B*_0_ = 0.38 T and RT and then dissolved with a hot solvent at high field yielded polarization of up to 0.88% for ^1^H (Figure 73)^619^ and 0.22% for ^13^C after CP.^620^ In a more recent study, *t*DNP was used to transfer polarization to several organic molecules in solution.^621^ To this end, a single crystal of naphthalene doped with deuterated pentacene with *p*_hyp_(^1^H) ≈ 20–25% was transferred at RT in a hand-held magnet, crushed, and dissolved to produce a concentrated (1.8 M) solution of hyperpolarized naphthalene in CDCl_3_ containing low concentrations of several organic molecules. As a result of intermolecular NOE-mediated polarization transfer, this procedure demonstrated signal enhancements beyond ε = −200 at 1.45 T, with the highest polarization (*p*_hyp_∼ 0.86%; ε = −1730 ± 60) achieved for CH protons of propargyl acetate.

**Figure 74:**
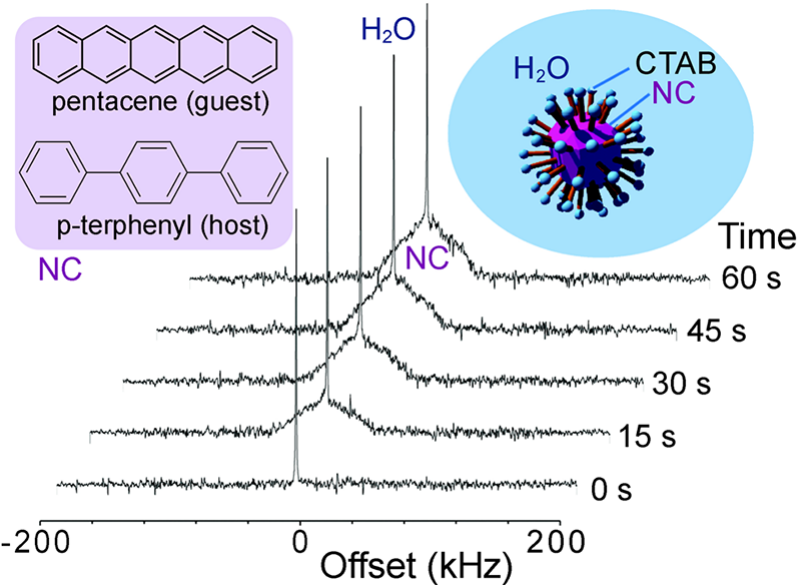
^1^H NMR spectra (0.676 T) of an aqueous dispersion of nanocrystals (NC) of *p*-terphenyl doped with 0.5 mol % pentacene at thermal equilibrium (0 s) and during *t*DNP process at RT. CTAB - cetyltrimethylammonium bromide surfactant. Reproduced from ref (594) with permission from the Royal Society of Chemistry.

Another approach used a stable aqueous dispersion of 100 nm nanocrystals of *p*-terphenyl doped with pentacene obtained by ball-milling of the bulk crystals in a surfactant solution, producing 0.083% ^1^H polarization at RT for nanocrystals but not for water^594^ (Figure 74). Transfer of ^1^H polarization from the host nanocrystal to bulk water was achieved recently, albeit with ε = 2.4 or lower at 0.66 T.^622^ Polarization of ^31^P and ^1^H nuclei in a derivatized C_60_ fullerene in frozen toluene-*d*_8_ was demonstrated in the context of qubit manipulations for quantum computing.^623^

The major limitations of *t*DNP are a limited range of known suitable guest chromophores, sample preparation restrictions, and suitable experimental conditions. In particular, pentacene solubility and perturbation of a host crystal limit the range of potential host compounds. Alternative sample preparation strategies were demonstrated for introducing pentacene in films of *p*-terphenyl or *trans*-stilbene (*p*_hyp_(^1^H) ≈ 4–13%)^624^ and eutectic mixtures of target compounds (e.g., aromatic carboxylic acids) with benzoic acid-*d*_6_ (*p*_hyp_(^1^H) ≈ 0.3–1.2%).^613^ Pentacene functionalized with metal-coordinating carboxylate moieties (4,4′-(pentacene-6,13-diyl) dibenzoic acid) and incorporated in a diamagnetic metal–organic framework (MOF) [Zn(MeIM)_2_]_n_ (ZIF-8; MeIM = 2-methylimidazolate) demonstrated ε ∼ 58 (at RT) and up to 100 (at 220 K) for the host ^1^H nuclei at *B*_0_ = 9.67 T.^625^ This approach could be potentially used to hyperpolarize molecules in solution via their reversible association with such functionalized MOFs.

Significant aggregation and phase separation of hydrophobic acenes in biologically relevant environments severely degrades their performance as polarizing agents. A recent study^626^ introduced a water-soluble porphyrin derivative, sodium salt of tetrakis(4-carboxyphenyl) porphyrin (TCPPNa), which was shown to generate up to 60% electron polarization in several crystalline and amorphous sugars and sugar alcohols. Moreover, for crystalline erythritol doped with TCPPNa, ε > 120 was observed at 0.65 T.

Aggregation of guest molecules and perturbation of host crystal structure limit guest concentrations in *t*DNP experiments. One approach to overcome this limitation is the use of charge-transfer materials comprising molecular cocrystals, in which the fraction of triplet-generating molecules is 50%, i.e., 2 orders of magnitude higher than in conventional *t*DNP samples. Electron spin polarization produced upon photoexcitation of a 1:1 cocrystal of phenazine and 1,2,4,5-tetracyanobenzene (PNZ/TCNB) at 445 nm was sufficient to generate maser oscillations at 2412 MHz in a high-Q MW cavity.^627^

Hyperpolarization techniques based on the same principles as ONP and *t*DNP are also applied to paramagnetic color centers associated with lattice vacancies in certain materials. In particular, in silicon carbide (SiC) a silicon vacancy and an adjacent carbon vacancy constitute a defect with a triplet electronic ground state. Polarization of ^13^C and ^29^Si nuclei in SiC can be achieved by near-IR broadband illumination in a magnetic field of 30–50 mT to bring the electron–nuclear spin states to a LAC either in the ground state (GSLAC) or in the excited state (ESLAC). For the 4H polymorph SiC wafer, these two conditions were revealed as two prominent maxima in the *B*_0_ field dependence of ^29^Si polarization at *B*_0_ = 30.0–33.5 mT and 46.5–49.0 mT, respectively, with the highest polarization reaching 99 ± 1%.^628^ Potential application of hyperpolarized SiC in MRI was demonstrated.^629^ Similar electronic spin states are associated with nitrogen-vacancy defects in diamond. These states can be interrogated and manipulated at either ensemble or single-spin level, making them useful for a range of fundamental and practical applications. Given the importance of this field of research and its rapid progress, the use of color centers in diamond for hyperpolarization purposes is addressed separately in section 3.10.

### 3.10 Nuclear Polarization with Nitrogen-Vacancy Color Centers in Diamond

**Figure 75:**
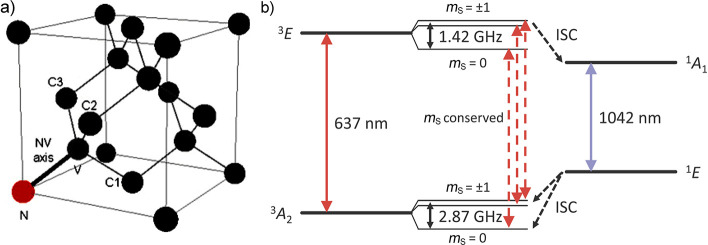
(a) The NV center consists of a nitrogen atom and a vacancy substituting for two adjacent carbon atoms in the diamond lattice. The NV axis may lie along any of the four main diagonals of the crystal, accounting for the total of eight possible orientations of the center. The molecular symmetry of the center is C_3v_. (b) Lowest electronic energy levels of the negatively charged NV center. The colored solid double-sided arrows indicate zero-phonon lines. Spin selectivity of the ISC transitions results in preferential optical pumping of the NV center into the *m*_S_ = 0 ground state. Reprinted with permission from ref (630), Springer Nature Customer Service Centre GmbH. Copyright 2017 Springer Cham.

The negatively charged nitrogen-vacancy (NV) color center in diamond^630^ (Figure 75) holds a special place among the plethora of paramagnetic centers both in diamond and other crystals due to a unique combination of its properties^631^ that makes it uniquely suitable for a multitude of applications, including sensing magnetic and electric fields, mechanical rotation, temperature and strain, generation of single photons on demand, performing quantum operations, and, as relevant to this review, for nuclear hyperpolarization. The basic idea of nuclear polarization using NV centers (sometimes referred to as NV-DNP) is the same as in other DNP techniques: electron polarization of the paramagnetic center, that can be generated in NV centers by application of an optical field followed by spin-selective ISC (section 2.2.2), is transferred to that of nuclear spins. Electron polarization of NV centers in the form of preferential population of the *m*_S_ = 0 sublevel (*m*_S_ is the projection of the electron spin on the NV axis) of the ground electronic ^3^A_2_ state is achieved when the center is optically excited into the ^3^E state (see Figure 75b). While the centers can radiatively decay back to the ground state, there is also spin-dependent intersystem crossing (ISC) into singlet states that provides a pathway for the *m*_S_= ± 1 states to decay into *m*_S_ = 0, responsible for the population accumulation in this state. Significant hyperpolarization of ^14^N (*I* = 1) or ^15^N (*I* = 1/2) nuclei intrinsic to the NV center was demonstrated, as well as that of ^13^C in the vicinity of NV centers and in the bulk of the diamond crystal. At the same time, efficient transfer of polarization outside of the diamond remains an open challenge that researchers have begun to tackle only recently (see below).

DNP of ^13^C in bulk diamond was initially implemented at cryogenic temperatures.^632−634^ The transfer of the electron polarization to ^14^N/^15^N and ^13^C nuclei proximal to the NV centers at room temperature was first discovered in the work involving single NV centers^635,636^ and later demonstrated with ensembles.^637^

One approach for transferring electron polarization to nuclei involves bringing magnetic sublevels of the NV center to near crossing via application of an external magnetic field. If the near-degenerate states show an avoided crossing, a.k.a. level anticrossing (LAC), due to hyperfine or superhyperfine interactions, this typically creates favorable conditions for polarization transfer to nuclei. Using NV centers in diamond, nuclear hyperpolarization is achieved in the vicinity of both the ^3^E excited-state level anticrossings^637,638^ (ESLAC, occurring around 50 mT for a field collinear with the NV axis) and the ground-state level anticrossings^639^ (GSLAC, occurring around 100 mT).

Complementary to the techniques relying on LAC are nuclear polarization techniques operating away from anticrossings that employ sequences of selective hyperfine-structure-resolved microwave transitions changing the electron spin projection *m*_S_ for a system in a selected |*m*_S_, *m*_I_⟩ state, radiofrequency transitions changing the nuclear projection *m*_I_, and green laser pulses resetting NV centers to the *m*_S_ = 0 state while preserving the nuclear spin. With proper choices of the pulses, such an operation results in enhanced population of the target nuclear state and can be used recursively to increase the polarization.^640^

For bulk NMR applications, of particular interest is using the relatively dilute NV centers in order to hyperpolarize the ^13^C nuclei everywhere in the diamond samples, including the regions relatively far from the color centers. Such “polarization spreading” relies on spin diffusion, that turns out to be efficient in diamond.^638^ In this experiment, a diamond single crystal containing NV centers was placed in a ≈50 mT fringe field of an NMR magnet and irradiated with green light. After a period of polarization, the sample was shuttled into the NMR magnet, where ^13^C NMR spectra were recorded, demonstrating bulk polarization levels averaged over the volume of the crystal in excess of 0.5%. The sample remained at room temperature for the duration of the experiment, and the sign of the ^13^C magnetization could be controlled by finely tuning the polarization field in the vicinity of the ESLAC.^638^ Similar experiments were later carried out and described elsewhere.^641,642^ The authors identified an additional bulk hyperpolarization mechanism that is efficient in the same range of magnetic fields as ESLAC but is related to optically induced cross-relaxation in the NV center ground state and involves the electron spin of a substitutional nitrogen impurity (the P1 center) with its ^14^N nucleus. According to the authors’ analyses, this mechanism dominates the bulk polarization.

Similar efficiency and control of nuclear hyperpolarization as demonstrated with bias fields of around ≈50 mT are also obtained in the vicinity of the GSLAC.^639^

The instrumentation needed for NV-based hyperpolarization largely depends on the specific technique used. For example, experiments utilizing the fringe field of a high-field NMR magnet used for detection require a sample shuttling system, while experiments using a static magnetic field or microwave sweeps require corresponding electromagnets and microwave sources. A common element is intense (milliwatts to tens of watts of power) sources of green light, which could be continuous-wave diode-pumped solid-state lasers or light-emitting diodes.

Bulk hyperpolarization intrinsic to diamond is often carried out with commercially available high-pressure high-temperature (HPHT) or chemical-vapor-deposition (CVD) single-crystal diamond with natural abundance of ^13^C isotope (1.1%), although ^13^C-depleted or ^13^C-enriched (up to essentially 100%) material has been produced as well. It is important to note that whether enrichment leads to an increase in the density of polarized nuclear spins in the bulk or in overall polarization is a nontrivial matter^643,644^ and may depend on the specific hyperpolarization strategy employed.

The nitrogen content of HPHT diamond typically ranges up to ≈200 ppm. To create the NV centers in the bulk, crystals are irradiated with relativistic charged particles, most often electrons with energies 1–15 MeV, that penetrate the material and produce crystal vacancies due to ionization losses. The samples are subsequently annealed to immobilize the vacancies and form the NV centers with a typical relative concentration of several ppm. Some experiments use ultrapure CVD diamond with low defect concentration, so single NV centers can be isolated. In some cases, a layer of NV centers several nm below the surface of the diamond is desired. Such layers are created by ion implantation or, more recently, with CVD overgrowth techniques, allowing enhanced coherence and optical properties of shallow NV centers. Some of the work described below employs micro- and nanodiamond.

Early work on bulk hyperpolarization of ^13^C nuclei in diamond showed that efficient hyperpolarization could only be achieved when the bias magnetic field was aligned within a small angle (less than a degree) to an NV axis which is collinear with one of the four main diagonals of the diamond cubic lattice. However, since many applications call for polarization in randomly oriented polycrystalline and powdered samples, this was a serious drawback.

**Figure 76:**
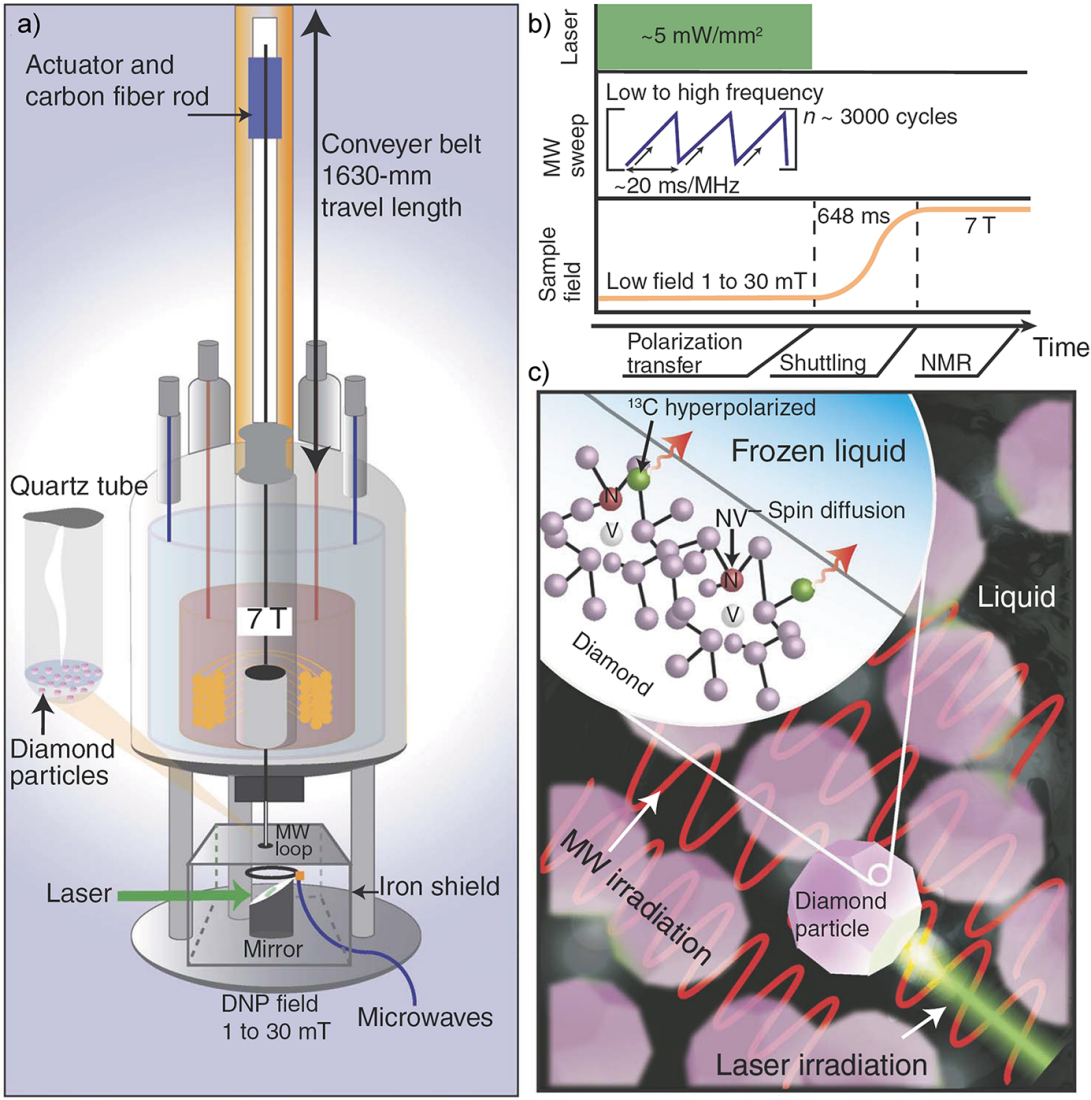
Hyperpolarization of ^13^C nuclei in nanodiamond with high-field NMR detection. Hyperpolarization occurs at a low (fringe) magnetic field, after which the sample is shuttled into the high-field NMR spectrometer for detection. Reprinted from ref (645). Copyright 2018 The Authors. Published by AAAS under CC BY-NC 4.0 license.

Luckily, it was subsequently shown^645,646^ that bulk ^13^C polarization can be achieved also for randomly oriented diamond particles with efficiencies comparable to those achieved for well-aligned single crystals. For instance, >0.25% bulk ^13^C polarization was demonstrated with ≈200 μm diamond particles.^645^ The experimental arrangement used in this work^645^ resembles that of the earlier study^638^ (Figure 76): hyperpolarization occurs in a low field, and the sample is shuttled into a high field for NMR detection. However, in this approach, polarization transfer from electrons to nuclei is accomplished by application of frequency-swept microwaves. As usual, the electron spins of the NV centers are optically pumped into the *m*_S_ = 0 state, a process that is largely independent of the NV orientation or magnetic field, as long as it is sufficiently weak (≲30 mT^645^). The transfer of polarization to nuclei occurs as a result of the microwave frequency sweep over a sequence of rotating-frame LACs, where the parameters are carefully tuned to ensure adiabatic passage through “strong” LACs (i.e., anticrossings with relatively large minimum separation of the eigenstates), with partial failure of adiabaticity at “weak” LACs. Moreover, light intensity should be low enough to keep low the probability of an optical transition during the passage through a LAC. An interesting feature of the technique is that the sign of hyperpolarization can be reversed by reversing the direction of the microwave frequency sweep. A detailed theory developed to describe the hyperpolarization dynamics corresponding to this method is reported elsewhere.^647^

The hyperpolarization technique used by Ajoy et al.^645^ has many attractive features for practical applications, while not requiring an excessively expensive apparatus. Indeed, hyperpolarization occurs at room temperature, microwave equipment in the 2–4 GHz range and green-light lasers employed in this work are readily available, and, most importantly, polycrystalline and powder samples can be used because the hyperpolarization mechanism works for an arbitrarily oriented sample. A microwave-free technique based on similar ideas and utilizing a bias field sweep across LAC has been recently demonstrated as well.^648^

If NMR measurements are to be performed at high field, a viable approach is in situ dynamic nuclear polarization (DNP) achieved by optically pumping NV centers (to the *m*_S_ = 0 state) and driving a microwave transition to one of the *m*_S_= ±1 states. Electron polarization is transferred to the bulk ^13^C nuclei via dipole–dipole interactions, although the details of the mechanism are not fully understood.^649^ The experiment was performed in a 420 mT field and the bulk ^13^C polarization of a 2 mm × 2 mm × 0.3 mm natural isotopic abundance single-crystal diamond was measured to be 6%. The polarization was also found to be tolerant to a crystal misalignment (i.e., the angle between the NV axis and magnetic field) of several degrees. A disadvantage of this method is that it requires microwaves of relatively high frequency (e.g., 8.9 and 14.6 GHz^649^).

While the thrust of much of the ongoing work is toward transferring hyperpolarization outside of the diamond or using diamond itself as an imaging contrast agent, there are also important applications making use of hyperpolarization within diamond. For instance, low-field hyperpolarization was used^650^ to study many-body thermalization of the spin system and the mechanisms of spin diffusion, itself responsible for the spread of hyperpolarization in the bulk of the crystal. Another future application is ^13^C-nuclei-based diamond rotation sensors.^651^

In addition to polarization transfer due to cross-relaxation in the laboratory or rotating frame, NV-based hyperpolarization is also possible using Hartmann–Hahn techniques where microwaves are used to drive the NV electron spins and the Rabi frequency of the electrons is matched to the Larmor frequency of the target nuclei.^652^ This approach is known as nuclear orientation via electron-spin locking (NOVEL; section 3.9).

**Figure 77:**
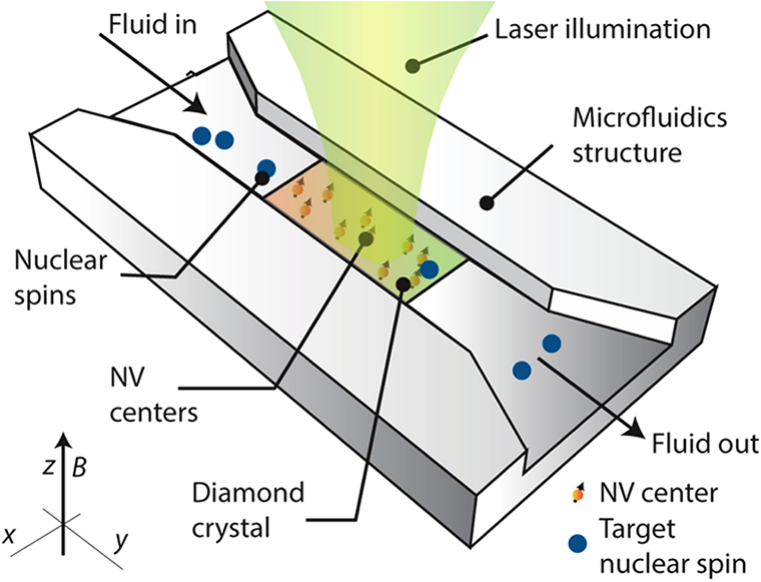
Concept of polarization transfer from hyperpolarized bulk diamond to a fluidic analyte. Adapted with permission from ref (653). Copyright 2014 American Chemical Society.

As mentioned above, it has been a major goal in the field of NV-diamond-based nuclear hyperpolarization to somehow transfer the polarization to nuclei outside the diamond, and in particular, to polarize liquids or gases relevant to bioanalytical applications (Figure 77). Two scenarios can be considered: (1) direct transfer of the NV electron polarization to nuclei outside the diamond crystal, and (2) transfer of polarization between NV-hyperpolarized nuclei inside the diamond and external nuclei. In both cases, one seeks to have large surface area of contact between diamond an external fluid. This suggests using microstructured bulk diamond or, if possible, micro- or nanodiamond. In the former case, it was theoretically found that it is best to use surfaces with micrometer-scale roughness.^653^ A variety of protocols for polarization transfer have been considered, for example, field-sweep “spin-ratchet” techniques;^654^ however, we are not aware of any published reports of direct experimental observations of polarization transfer to external nuclei.

On the other hand, several groups have reported impressive results based on indirect observation of such polarization transfer.^655−657^ In particular, polarization transfer from the NV centers to external nuclei using a cross-relaxation-induced polarization technique was described recently,^658^ in which a magnetic field applied along the NV axis with a magnitude of about 100 mT brings the magnetic sublevels of the electron spins in the color center close together, so that the residual separation matches the Zeeman frequency of the target nucleus (on the order of a MHz), facilitating cross-relaxation. Repeated optical pumping cycle on an NV center results in considerable transfer of polarization to the surrounding nuclei. In another study,^658^ single NV centers some 10 nm deep from the diamond surface (which is a trade-off for sufficiently strong coupling to the outside nuclei and maintaining good relaxation properties of the NV center) were used and the polarization of the target nuclei, the protons in poly(methyl methacrylate) (PMMA) applied to the surface of the diamond, was measured indirectly via the disappearance of the target-ensemble cross-relaxation feature as observed with the NV center itself. The attractive features of this approach include microwave-free, room-temperature operation, the possibility to tune to a specific target nucleus (with tuning accomplished by minute changes of the bias magnetic field) and high (50%) polarization levels inferred. In addition, the method does not require any chemical reactions, which is a feature of all NV-diamond-based hyperpolarization techniques. The projections for this approach are optimistic: for example, it is envisioned^658^ that NV arrays with surface density of 4 × 10^11^ cm^–2^ over a 4 mm × 4 mm diamond surface can achieve a polarization transfer rate of 4 μL s^–1^ at a polarization level of 80% for a concentrated 1 M solution of MRI contrast agent hydroxyethyl propionate (HEP) enriched with a ^13^C isotope, ^13^C_5_H_10_O_3_.

Experiments on NV-based hyperpolarization of nuclei outside the diamond are beginning to go beyond proof of principle and method development toward scientific applications. As an example,^659^ the protons in oil were polarized via NOVEL and the diffusion coefficient was measured in a layer of oil adjacent to the diamond surface and compared to that in the bulk, a direct probe of nanoscale hydrodynamics.

Finally, we remark that paramagnetic defects in diamonds other than NV centers can also be used to hyperpolarize external nuclei. An example is the report of enhanced MRI with nuclei proximal to nanodiamond hyperpolarized with an Overhauser technique.^660^

### 3.11 Parahydrogen-Based Hyperpolarization Techniques

In section 2.2.3 the H_2_ molecule was introduced as a leading example of a molecular system with the necessary symmetry properties to act as a source of nuclear spin hyperpolarization and support long-lived spin states. Before we discuss the parahydrogen-based hyperpolarization techniques, we briefly describe the properties of parahydrogen that make it uniquely suitable for a range of such techniques and their promising applications.

#### 3.11.1 Parahydrogen, Its Basic Properties and Enrichment

Molecular H_2_ exists as two different (ortho and para) modifications because the Pauli principle imposes strict requirements on the combined symmetry of the spin and rotational (in general, rovibronic) parts of the total molecular wave function. Hydrogen nuclei are identical fermions, thus in the ground vibronic state parahydrogen (*I* = 0) is allowed to have rotational states with even rotational quantum numbers (*J* = 0, 2, 4, ...) only, while orthohydrogen (*I* = 1) has odd values of *J* (*J* = 1, 3, 5, ...).^42^ Other combinations are strictly forbidden, and thus the nuclear spin state of the H_2_ molecule is impossible to change without an associated change in the rotational state. Therefore, nuclear spin conversion (NSC) inevitably involves a significant change in molecular energy. For H_2_ gas, the smallest energy difference is between the lowest ortho (*I* = 1, *J* = 1) and para (*I* = 0, *J* = 0) states of H_2_ which amounts to ca. 170.5 K (Figure 6a),^112^ i.e., is in the terahertz range. For comparison, an energy change associated with flipping a nuclear spin in molecules with no molecular symmetry in an NMR experiment does not normally exceed hundreds of MHz.

Normal H_2_ is available in high-pressure cylinders or can be produced with a hydrogen generator by water electrolysis. As H_2_ is a highly flammable gas, it is advisable to use an H_2_ sensor in the lab when such experiments are performed to warn of accidental leaks.

**Figure 78:**
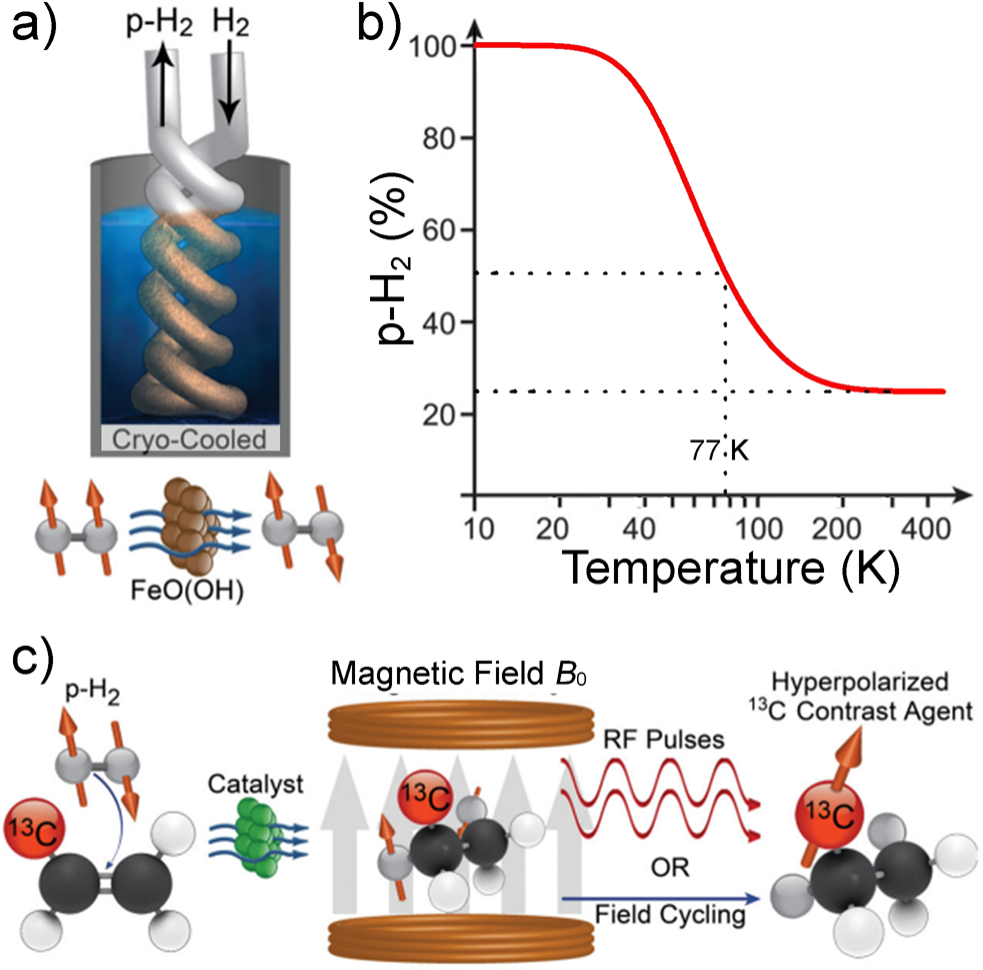
(a) Molecular hydrogen can be enriched in the para nuclear spin isomer by passing it over a magnetic material, e.g., FeO(OH), at cryogenic temperatures. (b) The para enrichment fraction as a function of temperature. (c) Parahydrogen can be chemically reacted with another molecule in solution to produce a hyperpolarized product molecule, and RF pulses or magnetic field manipulations can be used to transfer the hyperpolarization to chosen nuclear spins in the molecule. Note that ortho- and parahydrogen molecules are often depicted as having parallel and antiparallel orientations of their nuclear spins, respectively, which is a pictorial oversimplification of their true (triplet and singlet, respectively) nuclear spin states. Adapted with permission from ref (661). Copyright 2015 WILEY-VCH Verlag GmbH & Co. KGaA, Weinheim.

At room temperature and above, the equilibrium composition of H_2_ approaches the statistical ortho–para ratio (OPR) of 3:1, referred to as normal hydrogen (n-H_2_) (Figure 78b). H_2_ can be a source of hyperpolarization only if the OPR deviates from this value. The problem of enrichment of H_2_ in the para spin state (p-H_2_) has been solved for industrial H_2_ liquefaction processes,^662^ and is easy to perform on a small scale as well (see below).^663,664^ Since the rotational energies are much larger than spin interaction energies (e.g., Zeeman, dipolar, etc.), the energy separation of different spin states of H_2_ is relatively large (Figure 6a). Therefore, at cryogenic temperatures, the thermal equilibrium is shifted from n-H_2_ toward p-H_2_ fractions above the 25% p-H_2_ equilibrium content at room temperature. In practice, this is often done by flowing H_2_ gas through a cryostat containing a paramagnetic solid material such as charcoal or iron(III) oxide to accelerate equilibration by promoting ortho–para H_2_ conversion. The conversion process is schematically illustrated in Figure 78a.^661^

As can be seen in Figure 78b, the p-H_2_ enrichment that can be achieved depends on the temperature at which ortho–para conversion is performed, which in turn governs the design of the converter. Inexpensive immersion-type systems that use a bath of liquid nitrogen operate at 77 K and yield H_2_ mixtures with a ca. 50:50 ortho–para ratio.^664−666^ Higher enrichments require the use of lower conversion temperatures; for instance, ∼99.8% parahydrogen can be produced at H_2_ boiling point (ca. 20.3 K). The implementation of an immersion-type converter in combination with a liquid helium dewar has been reported which operates at 30 K (an incorporated heater is used to prevent condensation of H_2_ to avoid potential safety issues) and yields ∼97% p-H_2_ at 4.5 bar and 300 mL/min flow rate.^667^ A more popular option is based on the use of closed-cycle cryostats^668,669^ which can easily reach para enrichments close to 100%. Such equipment is substantially more expensive, and carries the cost of ∼50,000–125,000 USD (ca. 2021). A stand-alone helium-free generator is offered by Bruker with production capacity of ∼0.15 standard liters per minute (SLM) at ∼6 bar pressure of ∼92% p-H_2_ fraction. More advanced setups have been developed around ARS closed-cycle helium cold-heads with production capacity of up to 4 SLM at up to 50 bar pressure and over 98% p-H_2_ fraction.^670,671^ Note that 50% para enrichment yields a maximum polarization level of 1/3 of that obtained with pure p-H_2_, which is already sufficient for many applications. A potentially simpler alternative is to use bottles of liquid hydrogen, which is transported and stored in the para spin state.

It is also advantageous that parahydrogen is the most long-lived nuclear spin state (section 2.1) known to-date, as both radiative and nonradiative spontaneous ortho–para transitions are forbidden for an isolated H_2_ molecule due to the symmetry of the hyperfine Hamiltonian. Thus, once enriched, after the catalyst is removed parahydrogen can be stored under ambient conditions for days/weeks.^42^ The NSC to n-H_2_ is induced by intermolecular interactions and by collisions with container walls so that the actual storage time depends on storage conditions (container quality, admixture of oxygen and other gases, etc.).

Below we describe in detail how hyperpolarization of nuclear spins can be achieved by using p-H_2_ in homogeneous (section 3.11.2) or heterogeneous (section 3.11.3) catalytic hydrogenation reactions or in reversible chemical exchange processes (section 3.11.4).

#### 3.11.2 Homogeneous Parahydrogen-Induced Polarization

This section covers parahydrogen-induced polarization (PHIP) effects achieved by catalytic hydrogenation reactions in homogeneous solutions (sometimes referred to as hydrogenative PHIP) whereby the p-H_2_ molecules react irreversibly with unsaturated moieties of other molecules, and the product molecules are hyperpolarized (Figure 78c).

##### 3.11.2.1 The Technique

Homogeneous hydrogenation with p-H_2_ is almost always performed using transition metal catalysts.^672^ There are a few important considerations: (1) the time scale of the chemical reaction should be similar to or shorter than the time scale of the nuclear spin relaxation, which is typically seconds or tens of seconds in the products but may be even shorter in reaction intermediates; (2) a suitable unsaturated moiety (often, a double or a triple carbon–carbon bond) in a precursor molecule is required so that hydrogen atoms of an H_2_ molecule can be incorporated in the reaction product via a hydrogenation reaction; (3) usually, the reaction must be carried out in a pairwise manner, meaning that the protons from one p-H_2_ molecule are transferred together to the same product molecule, and not scrambled. In this section, we review how these limitations affect the design of PHIP experiments, how they can be partially overcome, and how in some cases they can be leveraged to enable applications of PHIP.

Parahydrogen itself cannot produce observable NMR signals, because the two protons are in a spin-0 singlet state. A chemical interaction is required to temporarily or permanently break the magnetic or chemical equivalence between the two protons; this can lead to hyperpolarization and enhanced NMR signals. The size of the interaction breaking the proton equivalence is key. If the interaction energy is larger than the proton–proton J-coupling, the singlet state is no longer an eigenstate, and enhanced NMR signals can be directly excited and observed. In this case the protons are described as being “far from magnetic equivalence” (also referred to as weak coupling of nuclear spins). Typical examples are when there is a large chemical shift difference at high field, or if one of the protons is directly bound to a heteronuclear spin (e.g., ^13^C) to which it has a large J-coupling. If the interaction energy that breaks the equivalence is smaller than the proton–proton J-coupling, the singlet state remains close to an eigenstate, and a suitable pulse sequence or magnetic field manipulation is required to convert the singlet order into observable magnetization. This is referred to as the near-equivalence regime (strong coupling of nuclear spins). The arctangent of twice the ratio between the proton–proton J-coupling and the equivalence-breaking interaction strength is known as the “Goldman angle”.^673^ There are techniques to generate enhanced signals in both cases, on the protons or on J-coupled heteronuclei. However, if the chemical reaction produces a molecule in which the hydrogen atoms remain magnetically equivalent, the spin order remains “locked” in the unobservable singlet state, and a further reaction is required to break the equivalence of the two protons.^63,674,675^

##### 3.11.2.2 Practical Aspects

The instrumentation for a variety of PHIP experiments was reviewed recently.^676^ The first requirement for PHIP is a way of generating enriched parahydrogen, as described in detail in section 3.11.1. The next requirement is an apparatus in which to carry out the chemical reactions. This importantly requires dissolving hydrogen gas into solution, which is often the rate-limiting step which needs to be performed rapidly on the time scale of nuclear spin relaxation to avoid excessive polarization losses. In many cases it is sufficient, and often even expedient, to perform the hydrogenation in an NMR tube within an NMR instrument, so that the signal can be acquired during the hyperpolarization process or immediately afterward. A pressurizable NMR tube containing a catalyst and an unsaturated precursor in the reaction solution under an atmosphere of parahydrogen at a pressure of 1–10 bar can be shaken to dissolve the hydrogen into solution and initiate the chemical reaction.^677^ For applications (e.g., in biomedicine) that require a single bolus of hyperpolarized material at high concentration or volume (∼100 μL or more) of polarized molecules, more sophisticated setups exist that involve bubbling of p-H_2_ into a pressurized reaction vessel,^678^ or spraying the reaction solution into an atmosphere of parahydrogen.^679,680^ For other applications such as pulse sequence optimization^681^ or acquiring 2D NMR spectra,^44^ it is often beneficial to have PHIP signals at steady state. For this purpose it is problematic to bubble or mechanically mix the parahydrogen into solution, because: (1) this is inherently irreproducible; (2) it is impractical to do NMR on an inhomogeneous sample; and (3) the solutions need to be periodically replaced since the reactions are irreversible. Microfluidic implementation of PHIP can be advantageous;^44^ parahydrogen can be brought into solution nondisruptively through a membrane,^682^ with the solution flowing at a constant rate into the detection volume.

To implement a pairwise hydrogenation required for PHIP, a precursor molecule that can receive a pair of hydrogen atoms and a suitable catalyst are required. Currently, transition metal complexes are used as catalysts to produce PHIP in homogeneous (liquid-phase) hydrogenations, with rhodium and ruthenium making up the majority. Some PHIP catalysts (e.g., Rh(dppb)(COD)BF_4_; dppb = 1,4-bis(diphenylphosphino)butane, COD = cyclooctadiene) require an activation step in which one of the ligands (COD in this case) is hydrogenated and leaves the metal center, yielding free coordination sites; this can be done before addition of the precursor to the solution.^683^

In a typical experiment, a solution of the unsaturated precursor and hydrogenation catalyst is prepared, usually in a deuterated solvent since this extends the relaxation times of the hyperpolarized nuclei throughout the chemical reaction. A solvent is usually chosen that gives a high rate of reaction and/or allows for further steps to be carried out (such as purification or rapid sample transport). In most cases, the solvent is degassed to remove residual oxygen, which is paramagnetic and leads to faster conversion of p-H_2_ back to n-H_2_, as well as relaxation of the hyperpolarized molecules of interest. Also, it is common for the solution to be heated to increase the rate of reaction.

**Figure 79:**
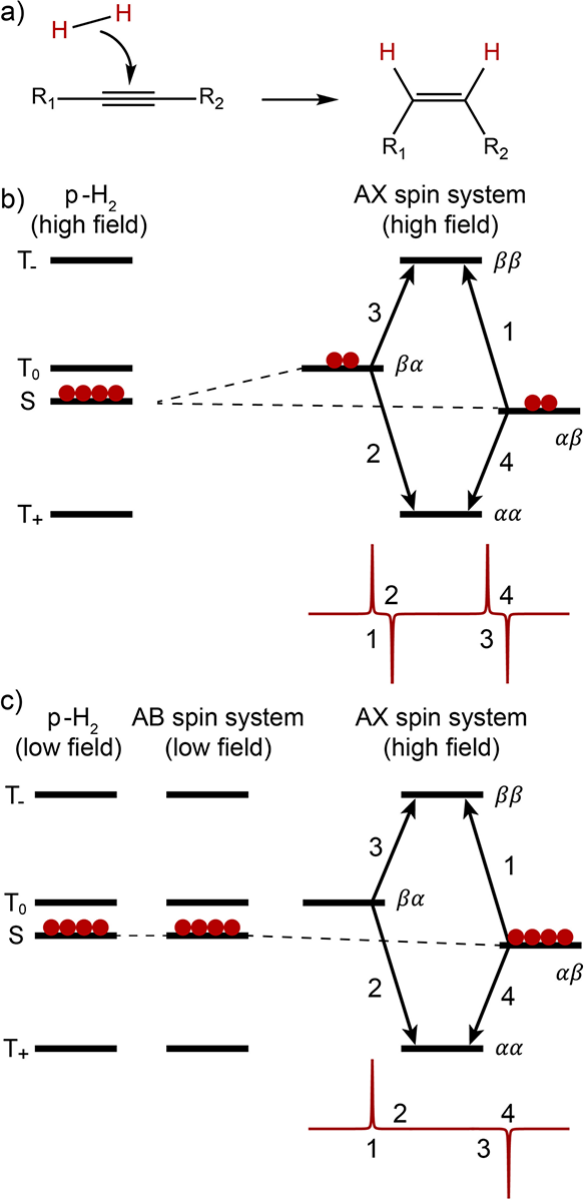
(a) A catalytic reaction of an unsaturated substrate with parahydrogen produces a product molecule in which the protons have a chemical shift difference. (b) In a PASADENA experiment the reaction is performed at a high field, and so the transformation of p-H_2_ into a weakly coupled (AX) spin system is nonadiabatic. This leads to a characteristic antiphase PASADENA peak pattern shown on the right. (c) In an ALTADENA experiment the reaction is performed at a low field, and the sample is then transported to a high field. The transformation of the p-H_2_-derived strongly coupled (AB) spin system of the product into an AX spin system is often adiabatic and leads to an ALTADENA peak pattern (shown for a RF pulse with a flip angle α ≪ π/2).

PHIP signals can be generated in simple pulse-acquire experiments. In a PASADENA (parahydrogen and synthesis allow dramatically enhanced nuclear alignment) experiment,^102^ the hydrogenation reaction (Figure 79a) is carried out at a high field, and there should be a chemical shift difference between the protons in the product molecule. Hyperpolarization-enhanced signals are excited by applying an RF pulse, with maximum enhancement given by a 45° flip-angle pulse (not 90° as is the case with thermal equilibrium polarization).^684^ In PASADENA experiments, nuclear spins of the parahydrogen-derived protons (*I*_1_ and *I*_2_) are in a correlated state of *I*_1*z*_*I*_2*z*_ spin order, which differs from thermal equilibrium spin order of the form *I*_1*z*_ + *I*_2*z*_. The resulting spectra thus show a characteristic antiphase (e.g., absorption/emission) pattern, with individual resonances enhanced beyond their thermal equilibrium intensity, but with no or little net increase in magnetization (for a weakly coupled spin system, see Figure 79b). Such antiphase NMR signals are not amenable to imaging or to spectroscopy in inhomogeneous fields, since magnetic field gradients (applied, or intrinsic) cause the enhanced antiphase peaks to destructively interfere, which results in partial or complete cancelation of the hyperpolarization-enhanced NMR signals. To overcome this, pulse schemes have been developed known as out-of-phase echoes^685,686^ which can be applied prior to imaging^675^ or as part of the imaging sequence.^687^ At the same time, the properties of the *I*_1*z*_*I*_2*z*_ spin order produced in PASADENA experiments can be leveraged to selectively observe PHIP signals while suppressing background signals of the solvent, the unreacted precursor, etc., which might be present in far higher concentration. For this purpose, pulse sequences termed “only parahydrogen spectroscopy” (OPSY) have been developed.^688,689^

In an alternative experiment known as ALTADENA (adiabatic longitudinal transport after dissociation engenders nuclear alignment),^690^ the hydrogenation reaction is carried out at a magnetic field low enough that the chemical shift difference between the parahydrogen-derived protons in the product molecule is suppressed, i.e., in the strong coupling regime. After the hydrogenation step, the sample is brought to a high field for NMR signal acquisition. The patterns of the multiplets in the NMR spectra of ALTADENA experiments (Figure 79c) differ from those in PASADENA in both sign and intensity, with the largest enhancement observed for a 90° flip-angle pulse.

An experiment in a sense similar to PASADENA exists for molecules in which the protons have a large difference in their J-coupling constants to a third spin, since this interaction breaks the proton equivalence in place of the chemical shift difference.^691^ This is particularly relevant at zero- to low-field, where chemical shift differences are suppressed. It is possible to hydrogenate a molecule at zero field, and if a third nuclear spin in the molecule breaks the proton equivalence, hyperpolarization-enhanced signals can be observed after applying a magnetic field pulse.^692^

In cases where the protons are in the near-equivalence regime, more sophisticated pulse sequences are required to convert the singlet order into magnetization, and for this purpose hard-pulse sequences such as singlet-to-magnetization (S2M) and its improved generalized version (gS2M) have been developed.^40,693^ Another option is spin-lock induced crossing^694^ (SLIC) which involves applying a constant-amplitude weak RF field to the protons, and alternative methods exist in which the weak RF field is adiabatically ramped.^695−697^ Polarization can be also transferred to another proton in the product molecule, either by using these RF-pulse-based methods that exploit the difference in its J-couplings to the two p-H_2_-derived hydrogens, or by achieving the near-equivalence regime by placing the sample in a low static magnetic field of an electromagnet or a permanent magnet.

Because heteronuclei relax much slower than protons in liquid-phase experiments, it is often desirable to transform the proton singlet order into magnetization of a heteronucleus (e.g., ^13^C, ^15^N) via intramolecular J-couplings. To this end, the S2M sequence was modified to achieve singlet-to-heteronuclear-magnetization (S2hM) conversion,^698,699^ and a more general form was developed (gS2hM).^700^ The SLIC sequence was also modified for this purpose,^698^ and many methods that use constant^50^ or adiabatically ramped RF fields are in use, such as adiabatic passage spin order conversion (APSOC) and adiabatic SLIC (aSLIC).^701^ Many other hard-pulse-based methods exist for polarization transfer to heteronuclei, and a more thorough review of this topic can be found elsewhere.^37^

In high-field experiments, a chemical shift difference between the protons in the reaction intermediate (often, a dihydride metal complex) can lead to singlet–triplet mixing^702^ and a loss of spin polarization. This can be partially alleviated by spin-locking the protons during the hydrogenation^703^ to suppress the chemical shift difference, or by applying a purge pulse prior to signal excitation.^681^

Alternatively, it is possible to work at a low field, and achieve spin order conversions by varying the applied static magnetic field. One of the most common methods is known as magnetic field cycling; after the hydrogenation step, the magnetic field is diabatically (rapidly) reduced to a near-zero field, and then adiabatically (slowly) increased back to an intermediate/high field.^679^ An alternative approach is to adiabatically invert the magnetic field (i.e., pass from negative to positive field values, or vice versa),^704^ and in the simplest case it is possible to spontaneously hyperpolarize a heteronucleus by performing the hydrogenation with parahydrogen at an ultralow field.^701^ The magnetic fields required for these polarization transfer experiments are typically on the order of hundreds of nanotesla to microtesla. The constraints on field homogeneity are usually weak (on the order of a hundred nanotesla or more) since the polarization transfer is close to optimal for a relatively broad range of magnetic field values.

Adiabatic ramping of the RF field or external magnetic field is necessarily slow, and fast spin relaxation can reduce the efficiency of the process. To overcome this problem, constant-adiabaticity schemes have been implemented at both a high field^701^ and an ultralow field,^705^ which are designed to yield the highest degree of adiabaticity for a given field-ramp duration. Another problem that can be encountered during spin order conversion at ultralow field is quadrupolar nuclei in the molecules becoming strongly coupled to the hyperpolarized spins. For the majority of quadrupolar nuclei, *T*_1n_ is so short they “self-decouple” from the spin-1/2 nuclei and do not act as a polarization sink, but this is not the case for deuterium or ^14^N nuclei.^706^ For this reason, a low-field scheme known as WOLF (weak oscillating low field) was developed where polarization transfer is completed at low field without entering the problematic ultralow-field regime.^707^

Polarization levels in PHIP experiments range from only a few times higher than that at thermal equilibrium to tens of percent, reflecting the wide range of chemical systems and optimizations of the specific cases. Spin relaxation during the reaction leads to a trade-off between the produced amount of hyperpolarized product and its polarization level. Because the rate of polarization transfer to other spins is approximately given by the size of the J-couplings between the p-H_2_ protons and the target nucleus, the final polarization level is further reduced if this or other additional steps such as sample transport or purification are required. Despite that, in a few cases, often those that are used to produce hyperpolarized metabolites for imaging applications, optimization of experimental protocols yielded polarization levels over 10% for ^13^C nuclei.^23^

Some key questions can be answered to estimate the likely outcome of a PHIP experiment: (1) How fast does the chemical reaction go to completion? (2) Does the hyperpolarized molecule contain additional spins that might induce faster relaxation of the p-H_2_-derived protons? (3) If polarization transfer is required, are the J-couplings large enough to facilitate fast transfer? (4) Are there additional spins in the molecule to which polarization can spread and be lost from the nuclei of interest?

**Figure 80:**
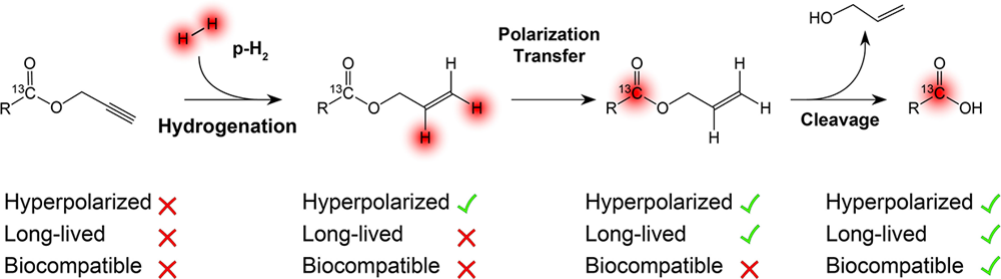
Illustration of the side arm hydrogenation (SAH) procedure to produce hyperpolarized R^13^COOH (the “target”) molecule. The RCOOH is initially chemically modified to incorporate a side arm that can be hydrogenated with parahydrogen. After hydrogenation, the polarization is transferred to the ^13^C nucleus of the target. An aqueous base is added to the solution to cleave off the side arm, and the target ends up in the aqueous phase, with the other molecules from the reaction remaining in the organic phase. Adapted with permission from ref (709). Copyright 2021 Elsevier B.V.

To extend the range of molecules polarizable via PHIP, the side arm hydrogenation (SAH) procedure was developed.^708^ A molecule of interest (e.g., [1-^13^C]pyruvate or [1-^13^C]acetate) is functionalized with a side arm moiety (e.g., a vinyl or propargyl group) which can be hydrogenated. After hydrogenation in an organic solvent, the polarization is transferred to the ^13^C spin, and an aqueous basic solution is added to cleave off the side arm through a saponification reaction. The chemical system is chosen such that the molecule of interest ends up in the aqueous phase, and the side arm remains in the organic (hydrophobic) phase along with the catalyst. This method is illustrated in Figure 80.^709^

Another important practical issue is the presence of the catalyst and other toxic components in the hyperpolarized solution which, for some specific applications such as generating PHIP-polarized metabolites for preclinical imaging, need to be removed. There are currently a few options for achieving this. The first is to avoid bringing the catalyst into solution in the first place, either through the use of heterogeneous catalysis, or homogeneous catalysts tethered to an insoluble support. This possibility is covered in more detail in section 3.11.3. If a homogeneous catalyst is used and needs to be removed, catalyst scavenging with commercially available metal scavengers^710^ or with a cation-exchange filter^711^ has been shown to be effective. However, a more thorough purification is often needed to additionally remove reaction side-products and unreacted starting material, and for this purpose two methods have been demonstrated.

One is the liquid–liquid phase extraction used in the SAH procedure described above. Another successful method used for purifying PHIP solutions demonstrated for [1-^13^C]fumarate is precipitation.^678^ After the polarization process, the pH of the solution is lowered; this causes fumarate to rapidly (in less than a second) precipitate out as solid fumaric acid, leaving the remainder of the reaction solution behind to be vacuum-filtered away. The solid fumaric acid can then be redissolved into a buffered solution for applications. Both methods involve relatively simple chemical purification procedures that rely on solubility differences between the desired product molecule and other species in solution, and we expect many similar methods to emerge to purify other PHIP-polarized molecules.

##### 3.11.2.3 Applications

The PHIP approach is rather diverse in terms of current and potential applications. While the major incentive for developing hyperpolarization techniques today is related to current and future biomedical applications, in the early days of PHIP its main application was in the mechanistic studies of homogeneous catalytic processes in solution that involve activation of H_2_.^712−716^ Note, however, that the initial experimental observations of PHIP were originally interpreted in terms of a different hyperpolarization mechanism (CIDNP; section 3.6), which inevitably led to incorrect mechanistic conclusions.^717^

As already mentioned, observation of PHIP effects reveals that addition of H_2_ is pairwise, which is an important mechanistic detail in itself.^718,719^ Furthermore, PHIP is generated as soon as the symmetry of p-H_2_ is broken, which often takes place already upon activation of p-H_2_ by a metal complex. This makes the direct detection and unambiguous structural identification of the key low-concentrated intermediates feasible. For many catalysts comprising neutral and cationic transition metal complexes, previously unobserved mono- and binuclear dihydrides have been successfully detected, and their detailed transformation pathways and the roles in the reaction mechanism established. Similar studies were performed in the absence of unsaturated substrates to focus in more detail on the H_2_ activation by metal complexes and clusters, on the formation of dihydride and polyhydride complexes, their structure and dynamics including isomerization and ligand exchange.^712,713,720^ We note that for detection of short-lived reaction intermediates, the experiments have to be performed in situ (PASADENA).

**Figure 81:**
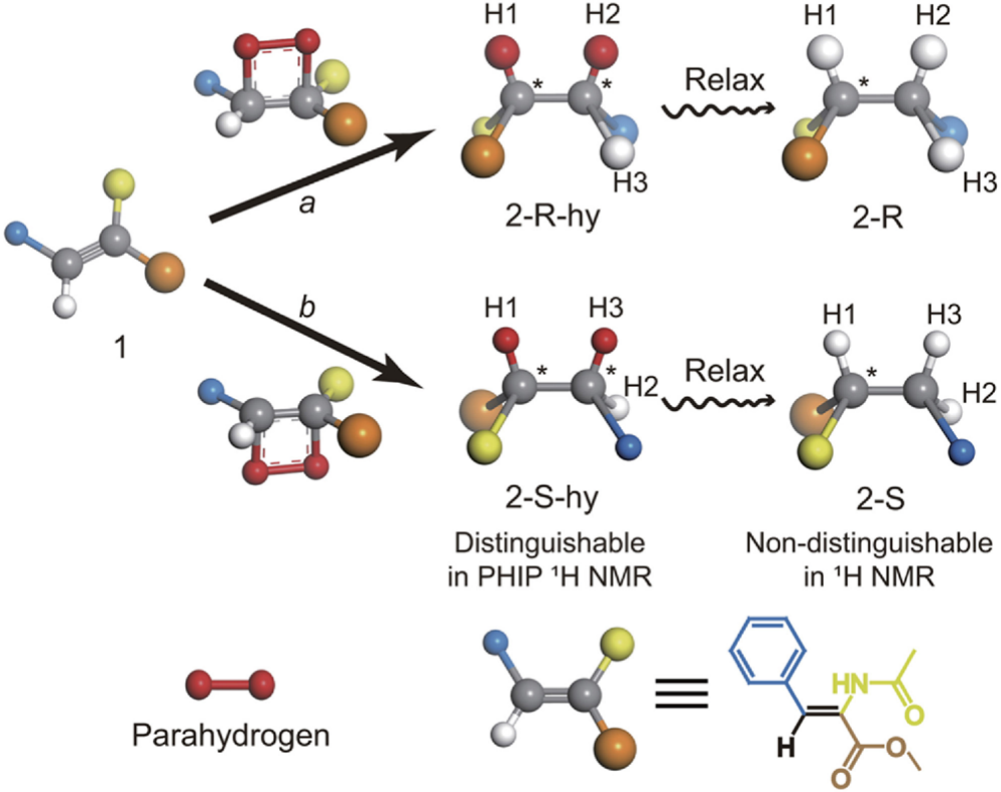
Use of PHIP to determine the enantioselectivity of asymmetric hydrogenation reactions, made possible by the fact that the hyperpolarized protons are distinguishable from thermally polarized protons. The PHIP-polarized molecules (labeled “hy”) are distinguishable in ^1^H NMR spectra since they are diastereomeric, but once relaxed they are chiral enantiomers and indistinguishable. Adapted from ref (722) with the permission of AIP Publishing.

PHIP can also be used to determine the enantioselectivity of asymmetric hydrogenation reactions;^721,722^ this is illustrated in Figure 81 for hydrogenation of methyl-α-acetamido cinnamate over a rhodium-based asymmetric hydrogenation catalyst. Determination of the enantioselectivity of this reaction using parahydrogen is possible because the PHIP-derived protons are distinguishable from the proton with thermal-equilibrium nuclear spin polarization. While the protons are hyperpolarized, the product molecule contains two chiral centers, and this diastereomerism means the chirality can be directly revealed in 1D ^1^H NMR spectra; something that is no longer possible after the protons relax and the molecules contain only one chiral center (and are hence enantiomers).

A significant sensitivity boost provided by PHIP is also useful for chemical reaction monitoring,^4,723^ enhancing NMR signals for low-^724^ or zero-field^725^ NMR (where thermal-equilibrium polarization levels are prohibitively low), and metabolomics and trace analysis^726^ (the latter application is discussed in more detail in section 3.11.4).

**Figure 82:**
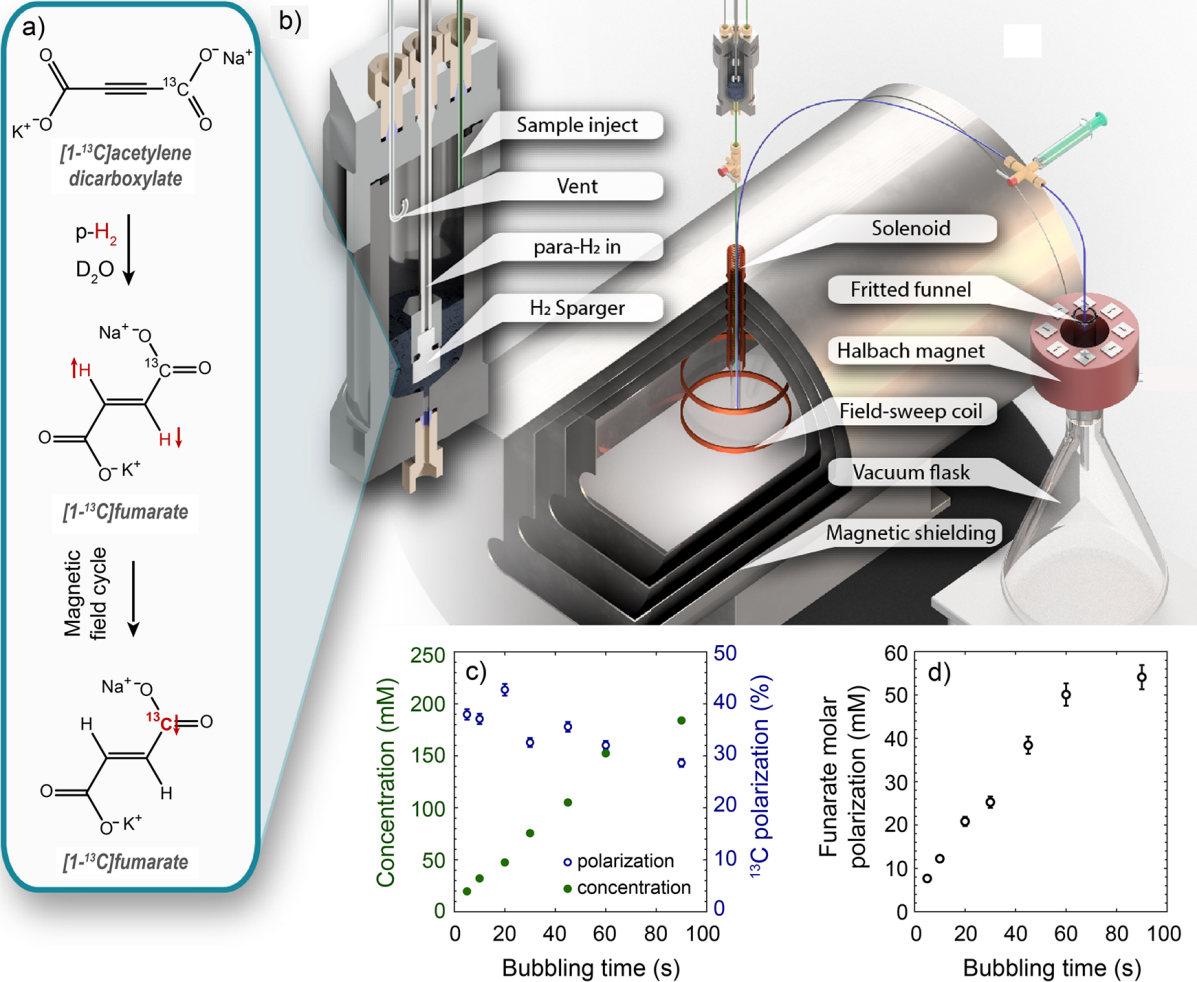
Hyperpolarization and purification (via precipitation) of [1-^13^C]fumarate. (a) Catalytic trans hydrogenation of a [1-^13^C]acetylene dicarboxylate precursor with parahydrogen over the Ru-based catalyst to generate [1-^13^C]fumarate with enhanced proton singlet spin order, followed by a magnetic field cycle to transform this singlet order into ^13^C magnetization. (b) The apparatus used for the experiment. A zoom of the chemical reactor is shown on the left, including a sparger to dissolve p-H_2_ into solution more effectively during bubbling. The magnetic shield and electromagnetic coils are used for the magnetic field cycle. On the right, the precipitation stage is shown; a Halbach permanent magnet array provides a field in which the precipitation is carried out. (c) [1-^13^C]Fumarate concentration and polarization for different p-H_2_ bubbling durations. (d) Molar polarization (i.e., the product of [1-^13^C]fumarate polarization and concentration) for different bubbling durations. Adapted with permission from ref (678).

Currently, PHIP is widely applied to produce hyperpolarized molecules for MRI, both for perfusion imaging^711^ and for metabolic imaging.^677^ In general, dissolution DNP (section 3.4) can produce solutions with higher polarization and a wider range of hyperpolarized molecules, but PHIP has an important role to play since it is comparatively inexpensive and has a higher turnover rate (on the order of one sample per minute). For in vivo imaging, a key requirement is that the hyperpolarized solution be pure from toxic substances. Particularly promising in this context are the production of hyperpolarized [1-^13^C]pyruvate by side arm hydrogenation^708^ and of hyperpolarized [1-^13^C]fumarate produced via an uncommon trans hydrogenation over a Ru-based catalyst^678^ (Figure 82) outlined above. Both methods have been applied for in vivo metabolic imaging.^677,710^ Other PHIP-polarized molecules have been used for in vivo imaging in mice, including succinate and phospholactate,^727^ and hydroxyethyl propionate.^728^ Nevertheless, because of the specific requirements of the PHIP technique. its applications in vivo remain rather challenging at present. However, given the high solution turnover rates and ease with which PHIP polarizers can be installed, this hyperpolarization technique might be more suited for in vitro studies, especially for high-throughput applications in e.g., drug discovery. Indeed, there are already a number of examples of in vitro studies using PHIP-polarized molecules.^683,729,730^

**Figure 83:**
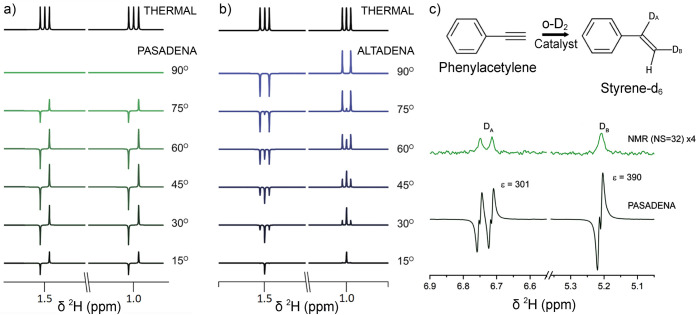
(a,b) ODIP experiments can be carried out to yield PASADENA (a) and ALTADENA (b) ^2^H NMR spectra; the spectral pattern dependence on excitation pulse flip angle is shown. (c) ^2^H NMR spectra of styrene-d_2_ after chemical reaction of a precursor molecule (phenylacetylene) at high field with o-D_2_. The resulting PASADENA spectrum is shown below, and a thermal equilibrium spectrum obtained with NS = 32 transients and vertically expanded by a factor of 4 is shown above. The two deuterium atoms and the corresponding NMR signals are labeled D_A_ and D_B_. Also shown are the signal enhancements ε evaluated for the two multiplets. Adapted from ref (732) with the permission of AIP Publishing.

A technique similar to PHIP called orthodeuterium-induced polarization (ODIP) exists that uses molecular deuterium (D_2_) in place of molecular hydrogen.^731^ Since deuterium nuclei are bosons with nuclear spin *I* = 1, the overall wave function of D_2_ must be symmetric with respect to the permutation of the two nuclei, and this constrains symmetric and antisymmetric spin states to symmetric and antisymmetric rotational states, respectively. Although the energy separation between rotational states of D_2_ is smaller than that of H_2_ due to its larger moment of inertia, it is still possible to achieve relatively high ortho enrichment by cooling (Figure 7). The room temperature fraction of orthodeuterium (o-D_2_) is 66.7%, but this rises to 92.5% at 30 K.^732^ Conventional PHIP experiments such as PASADENA and ALTADENA can then be performed using o-D_2_ in place of p-H_2_: simulated and experimental spectra are shown in Figure 83. ODIP is a promising route to hyperpolarizing deuterium nuclei in solution, which is usually challenging because of the short *T*_1n_ due to quadrupolar relaxation.

##### 3.11.2.4 Frontiers and Challenges

A combination of factors including typically lower polarization levels, the limited range of molecules that can be hyperpolarized via PHIP, and the necessity to develop dedicated procedures to purify the PHIP-polarized solutions for in vivo use have hindered such applications to date.

The vast majority of PHIP experiments in liquid phase involve stereoselective cis hydrogenation of an unsaturated substrate. At the same time, examples exist of alternative hydrogenation pathways such as trans hydrogenation (anti addition of the two H atoms),^733^ gem-hydrogenation (addition to the same carbon atom),^703^ hydroformylation,^734^ and methoxycarbonylation.^735^ The latter two reactions provide notable examples of PHIP effects observed in reaction products and intermediates which inherit only one of the two H atoms from p-H_2_ molecule–the so-called one-H PHIP. We note, however, that observation of one-H PHIP still requires that the initial activation of p-H_2_ molecule which breaks its symmetry is pairwise and is followed by spin evolution in a pair of nonequivalent hydrides.

Finding hydrogenation catalysts that can yield alternative product molecules is key to widening the scope of hydrogenative PHIP. The majority of PHIP reactions are carried out using rhodium catalysts, but other metal catalysts are used such as ruthenium,^736^ palladium,^735^ platinum–tin,^734^ iridium,^734^ osmium,^737^ and cobalt,^738^ and it is even possible to observe PHIP and SABRE effects with metal-free catalysts.^739,740^

In some cases, the parahydrogen protons end up occupying chemically and magnetically equivalent positions in the product molecule, and the spin order of the proton spin singlet state remains locked in this nonmagnetic state until a further chemical reaction breaks the equivalence, releasing hyperpolarized NMR signals. These demonstrations are promising since the singlet order can persist for times much longer than the proton *T*_1n_ time,^37,63^ meaning the enhanced spin order can be stored in this manner and revealed as hyperpolarization by a chemical reaction. This has been used as a way of measuring the lifetime of proton spin singlet order,^674^ and as a method for performing metabolic imaging with protons rather than the more-commonly used ^13^C nuclei.^675^

Another extension to PHIP is the use of light-activated catalysts.^741^ The addition of fresh parahydrogen to a metal center can be initiated by a laser pulse, which removes H_2_ from the metal center, leaving free coordination sites for fresh p-H_2_ to add across.

**Figure 84:**
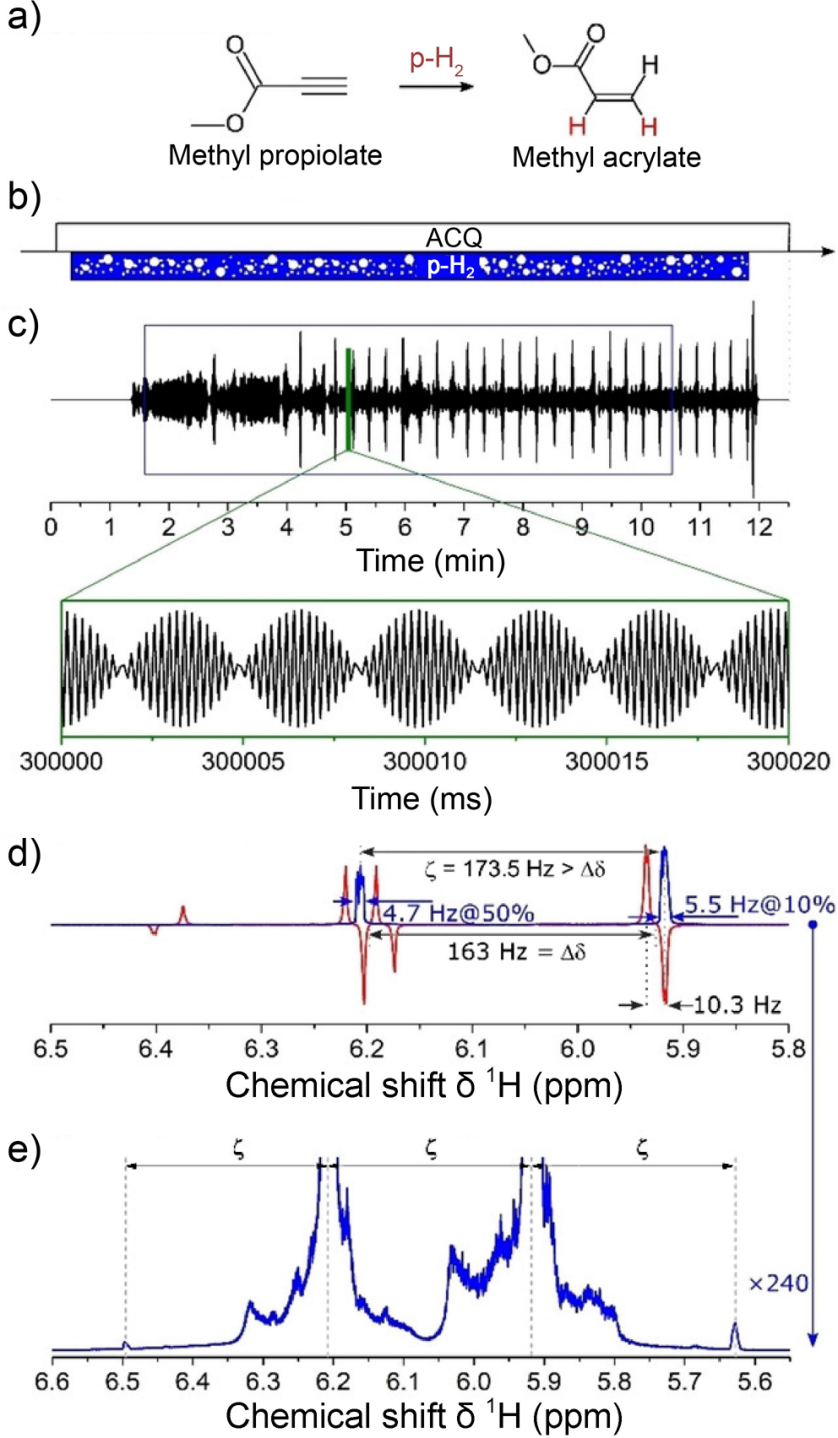
(a,b) The chemical reaction that forms the basis of the PHIP RASER and the experimental protocol. A 100 mM solution of methyl propiolate in acetone-*d*_6_ was used; p-H_2_ was bubbled into the solution at 0.2 bar gauge pressure continually for the duration of the experiment, such that hyperpolarized methyl acrylate was generated throughout. (c) The NMR signal acquired during the experiment. (d) A Fourier transform of the RASER signal (blue trace) and comparison with PASADENA signals from the same chemical system (red trace). ζ is the frequency difference between RASER lines. (e) An expansion of the NMR spectrum in (d). ACQ = NMR signal acquisition. Reproduced with permission from ref (744). Copyright 2020 The Authors. Published by Wiley-VCH Verlag GmbH & Co. KGaA.

One interesting phenomenon is using parahydrogen as a polarization source to induce a radiowave maser (RASER).^742^ The RASER is based on a well-known effect in NMR, namely “radiation damping”^743^ (in fact, like many terms in NMR, this is also a misnomer as there is essentially no radiation involved in NMR). Essentially, when the magnetization of a sample that is efficiently coupled to a tuned coil is large enough, it can induce a current in the coil, which acts back on the sample as a time-dependent additional magnetic field. If the magnetization starts out inverted (oriented along −*z*) and is weakly perturbed, the radiation-damping field pulls the magnetization back along *+z*, and the trajectory taken brings the magnetization vector temporarily into the xy-plane. This is the basis of parahydrogen RASER effects; when part of the NMR signal is inverted (e.g., some of the resonances in a PASADENA spectrum), the inverted magnetization can self-excite to yield transverse magnetization. If the experiment is set up in a way to produce continuous PASADENA signals, this self-excitation can in principle persist forever. Hydrogenative-PHIP-based RASER has been demonstrated at high field (14 T)^744^ and in a benchtop NMR device (1.4 T);^745^ the high-field RASER is illustrated in Figure 84.

PHIP-X is a recent development with the potential to make PHIP a more general hyperpolarization method.^746^ In this experiment, a compound with an exchanging proton (e.g., an unsaturated alcohol) is hydrogenated at a low field, and since the protons are strongly coupled, the exchangeable proton becomes polarized. It can then carry the polarization into other molecules with which it undergoes chemical exchange. This has been used to hyperpolarize protons in pyruvate and lactate, and ^13^C spins in glucose. Although the polarization levels are currently much lower than 1%, this method holds promise as a more general method for creating PHIP-based hyperpolarization.

**Figure 85:**
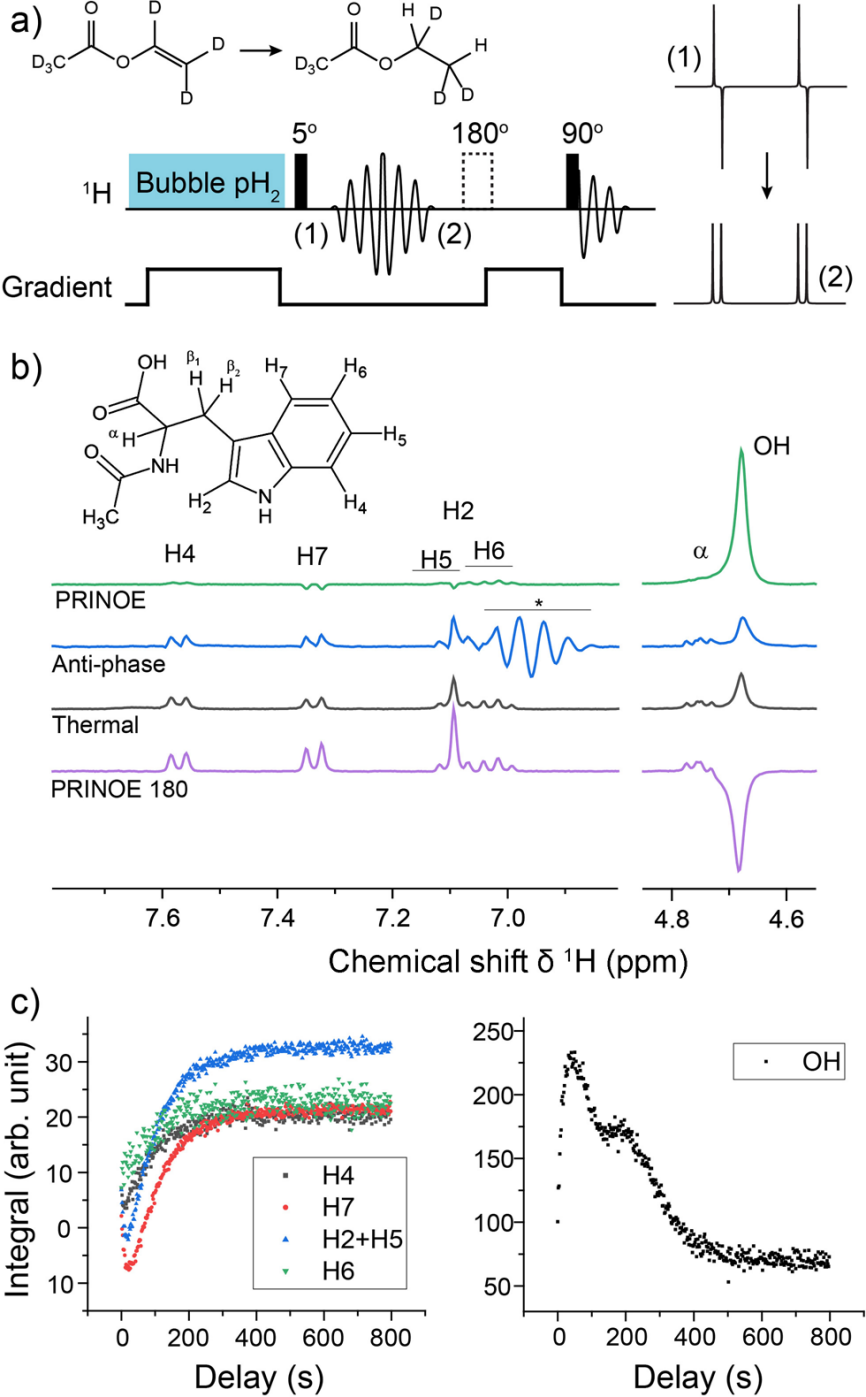
A PRINOE experiment in which 100 mM ethyl acetate-*d*_6_ is polarized via PHIP and then used to hyperpolarize 100 mM *N*-acetyl-l-tryptophan in [D_4_]MeOH. (a) A simplified scheme showing the principle of the PRINOE experiment. Parahydrogen reacts with vinyl acetate-*d*_6_ at a high field to form ethyl acetate-*d*_6_, with a gradient applied to suppress radiation damping. The proton spin order on ethyl acetate-*d*_6_ is *I*_1*z*_*I*_2*z*_, which would give rise to an antiphase peak pattern illustrated in (1). A 5° flip-angle pulse is applied to initiate radiation damping, which leads to *I*_1*z*_ + *I*_2*z*_ spin order which exhibits an absorptive peak pattern illustrated in (2). Since the hyperpolarized spin order of the source molecule now carries a net magnetic moment, it can lead to NMR signal enhancement of a target molecule in solution via the intermolecular NOE effect. (b) Molecular structure of the target *N*-acetyl-l-tryptophan, and the resulting NMR spectra from performing the PRINOE experiment without and with a 180° flip-angle pulse applied during the pulse sequence. (c) The PRINOE-enhanced signals from *N*-acetyl-l-tryptophan acquired using 5° flip-angle pulses every 2 s. Adapted with permission from ref (749). Copyright 2021 The Authors. Published by Wiley-VCH GmbH.

Another route to developing a more general hyperpolarization method using PHIP is based on the spin-polarization-induced nuclear Overhauser effect (SPINOE).^747^ Molecules in solution that experience fluctuating dipolar couplings to a hyperpolarized source molecule can become hyperpolarized through the intermolecular nuclear Overhauser effect. This was originally demonstrated using laser-polarized ^129^Xe,^747^ and has also been shown using *d*DNP-polarized [1,4-^13^C_2_]fumarate.^748^ Recently, it was shown that molecules polarized by PHIP can also be used as the polarization source in a parahydrogen- and RASER-induced NOE (PRINOE) experiment.^749,750^ Hydrogenation of a suitable precursor such as (perdeuterated) vinyl acetate with parahydrogen under PASADENA^749^ or ALTADENA^750^ conditions was used to produce hyperpolarized ethyl acetate. In both types of experiments, no net magnetization is produced on the protons, since the enhanced NMR signals are in equal part positive and negative. Upon application of a 2–5° flip-angle pulse, the positive and negative NMR signals experience strong radiation damping, causing the magnetization vectors to reorient along the magnetic field axis. The result is net magnetization, which can then lead to polarization of other molecules in solution via an intermolecular NOE. Enhancements of up to −3 were obtained on the protons of 100 mM *N*-acetyl-l-tryptophan in solution.^749^ Similar results were demonstrated^750^ for several other substrates and target molecules, including hyperpolarization of heteronuclei (^19^F, ^31^P). The procedure and some results are illustrated in Figure 85.

The biggest limitation of PHIP is that it is not a general technique: specific reaction pathways must be designed, and there is great scope for extending PHIP to chemical reactions beyond producing alkenes and alkanes via cis hydrogenation. There is already some precedent for this as mentioned above, but there is much room for innovation.

Even when a suitable synthetic route is found for hyperpolarizing a desired molecule, the solutions are contaminated with the catalyst, reaction side products, and unreacted precursor. Some specific methods have been designed to purify PHIP-polarized molecules from solution, and a few examples of PHIP-catalysts being filtered out of solution exist, but there is clearly ample room for more physicochemical purification methods to be employed to yield clean hyperpolarized solutions.

#### 3.11.3 Parahydrogen-Induced Polarization in Heterogeneous Catalytic Processes

Similar to PHIP produced in homogeneous catalytic processes (section 3.11.2), its heterogeneous version (HET-PHIP)^25,112,751,752^ uses the correlated nuclear spin state of parahydrogen (p-H_2_) as hyperpolarization source and usually requires pairwise H_2_ addition to a substrate. This implies incorporation of both hydrogen atoms of a p-H_2_ molecule into the same product molecule where they become chemically or magnetically inequivalent. The requirement of pairwise H_2_ addition, however, is much more difficult to meet with typical heterogeneous (solid) catalysts, so much so that HET-PHIP was considered impossible.^753^

For hydrogenations in a liquid phase (slurry), unsaturated substrates are usually dissolved in a suitable solvent. For gas-phase hydrogenations, gases can be premixed with p-H_2_ before the reaction. Another approach is to bubble p-H_2_ through a volatile liquid and supply the p-H_2_/vapor mixture to the solid catalyst for hydrogenation. In either case, it is required that a substrate possesses an unsaturated moiety (e.g., a double or a triple carbon–carbon bond) which can be catalytically hydrogenated.

**Figure 86:**
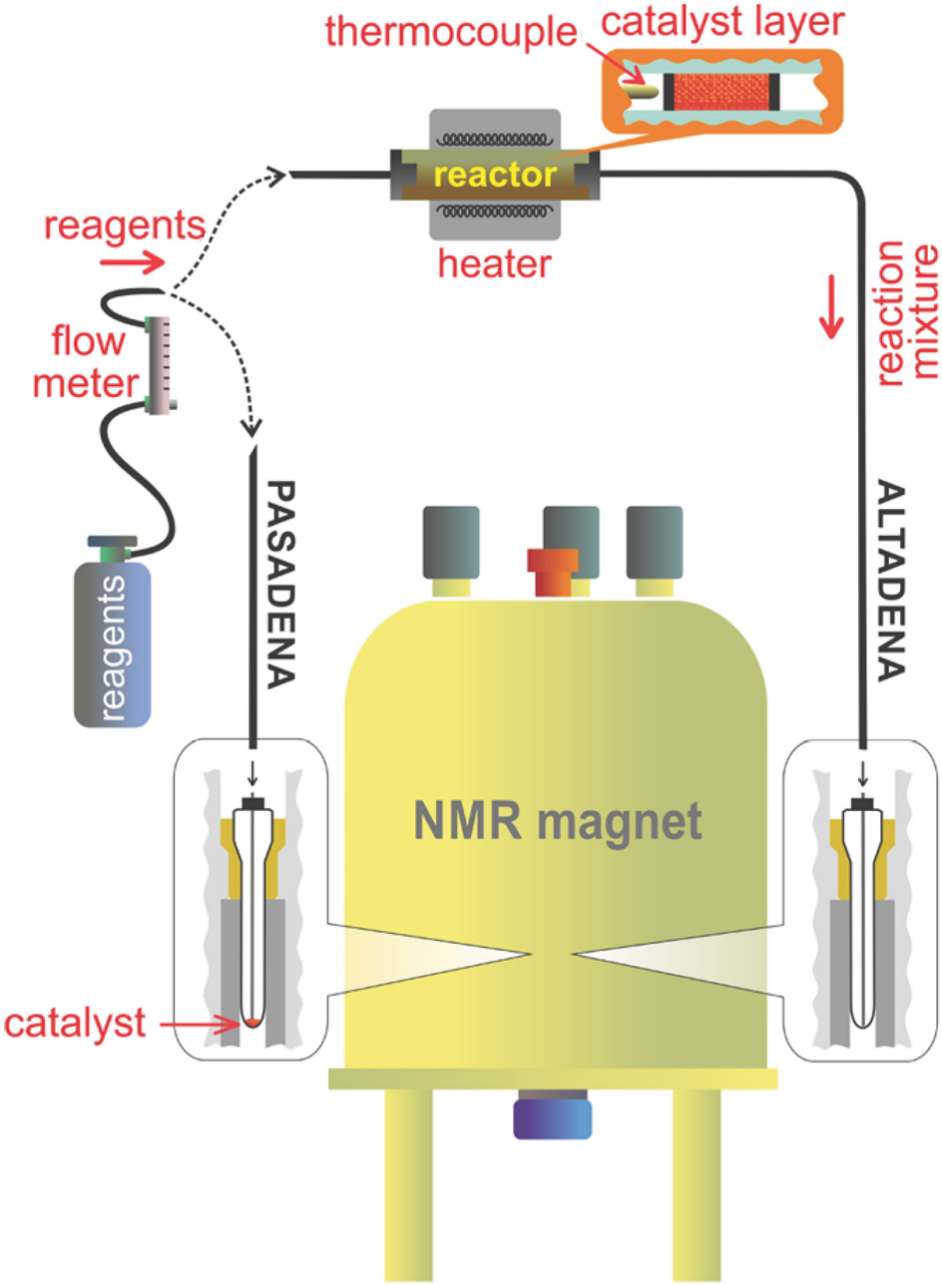
Experimental setup and procedure for HET-PHIP in gas–solid processes. The reagent gas flow controlled by a flow meter is supplied either to the packed-bed reactor positioned in the Earth’s field and then to the empty sample tube inside the NMR magnet (right branch, ALTADENA experiment), or directly into the sample tube with the catalyst inside the NMR magnet (left branch, PASADENA experiment). Reproduced with permission from ref (754). Copyright 2018 American Chemical Society.

In situ hydrogenations (PASADENA) can be performed directly in an NMR tube loaded with catalyst and, if required, with substrate solution (for gas–liquid–solid hydrogenations), while p-H_2_ (or its mixture with other gases or vapors for gas–solid hydrogenations, Figure 86) is supplied and then vented via the connected tubing/capillary.^754^ For elevated gas pressures, medium- or thick-walled NMR tubes may be required. Solid catalyst particles are agitated when p-H_2_ is bubbled through the catalyst slurry or when the gas is supplied at high flow rates to a dry catalyst powder; in both cases, this can significantly accelerate the reaction by reducing mass transport limitations. For a continuous-flow hydrogenation outside an NMR magnet (ALTADENA), the solid catalyst can be placed between glass wool plugs in a flow-through reactor. For catalyst pretreatment and/or to facilitate the reaction, the reactor can be heated from outside.

**Figure 87:**
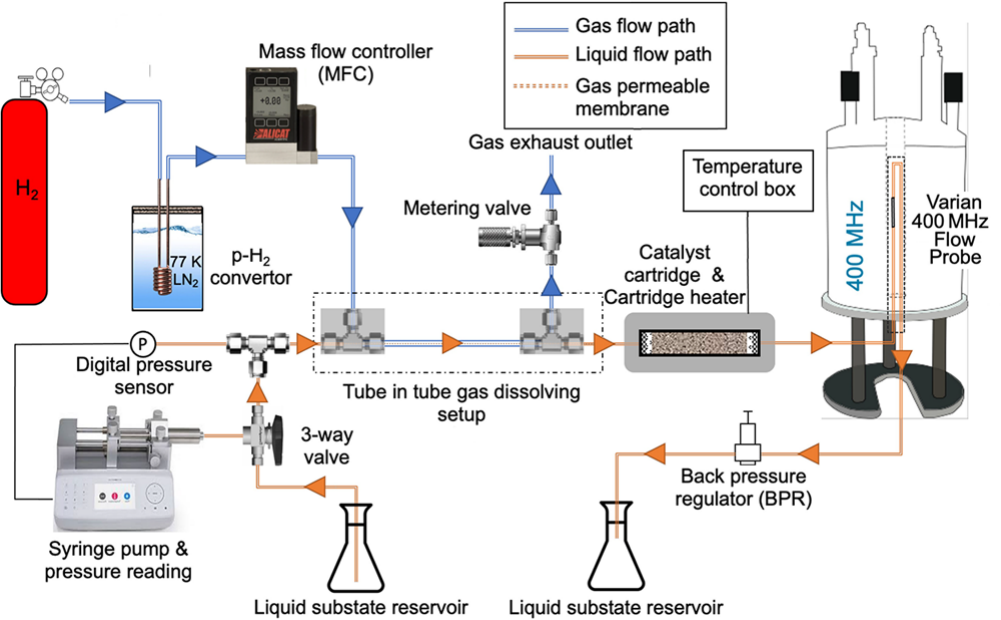
Experimental setup and procedure for HET-PHIP in gas–liquid–solid processes. The liquid is drawn into the syringe from the left liquid reservoir and the three-way valve is then switched to allow the liquid to flow through the tube-in-tube device for membrane dissolution of p-H_2_ and then into the heated catalyst cartridge. After that, the liquid continues to flow into the magnet for detection and is then collected in a separate reservoir. Adapted with permission from ref (755). Copyright 2021 Wiley-VCH GmbH.

After the reaction is performed in a solid–liquid slurry, the solid catalyst can be filtered out in most cases. An alternative scheme involves a continuous stream of a reacting fluid constantly supplied to the reactor and collected downstream. Continuous production of hyperpolarized allyl and propyl acetates in methanol-*d*_4_ was recently achieved^755^ using a setup combining membrane dissolution of p-H_2_ and a packed-bed catalytic reactor for flow-through hydrogenation of propargyl acetate (Figure 87). A modified setup for achieving a steady-state polarization level upon recirculation of a reacting solution at low per-pass conversion levels was reported later.^756^ The continuous hydrogenation approach is particularly easy to implement for producing continuous streams of hyperpolarized gases. Transfer of the polarized gas to an NMR instrument should be fast to avoid significant polarization losses caused by relaxation.

The first demonstration of HET-PHIP effects^757^ was achieved with rhodium complexes immobilized on solid supports (silica, polymers). The expectation that such catalysts would inherit the pairwise reaction mechanism from their homogeneous counterparts was indeed confirmed experimentally in both liquid- and gas-phase hydrogenations. Furthermore, soon after, the industrial-type catalysts comprising metal nanoparticles (MNP) deposited on oxide supports were also shown to produce HET-PHIP effects.^758^ Extensions to metal oxides, sulfides and carbides and other types of solid catalysts followed,^25,751^ with some of them being of significant importance for modern industrial catalytic processes.

Performance of different catalysts is usually compared in terms of the percentage of pairwise selectivity (PS) of H_2_ addition instead of polarization level *p*_hyp_ (*p*_hyp_ = PS × (4*f* – 1)/3 in ALTADENA and 0.5 × PS × (4*f* – 1)/3 in PASADENA experiments, where *f* = [p-H_2_]/([p-H_2_] + [o-H_2_]) is the parahydrogen fraction in H_2_). Another essential factor to consider is the amount of the hyperpolarized product (i.e., the conversion of reactants to products, *X*) accumulated before relaxation becomes significant.

**Figure 88:**
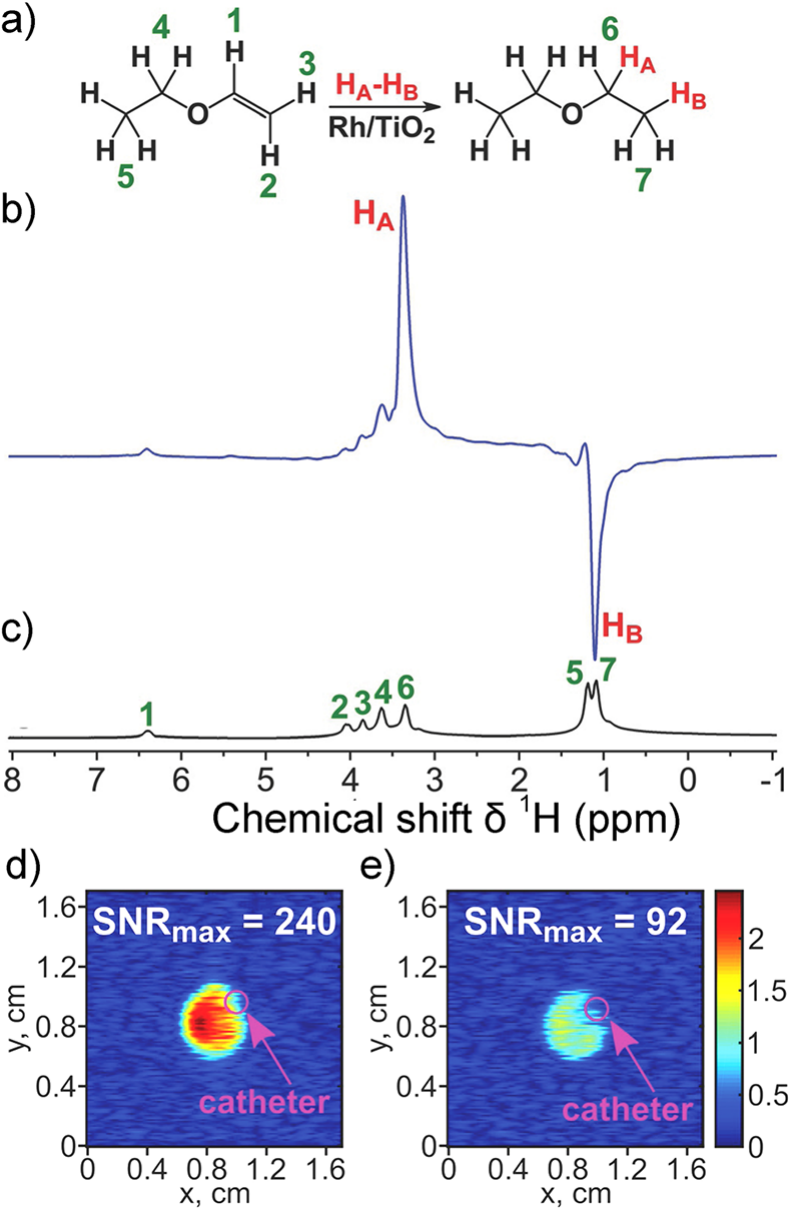
(a) The scheme of pairwise addition of p-H_2_ to ethyl vinyl ether (EVE) with the formation of hyperpolarized diethyl ether (DE) over Rh/TiO_2_ catalyst. (b) ^1^H NMR spectrum of gaseous hyperpolarized DE acquired while the gas mixture was flowing at 4.3 mL/s gas flow rate. (c) ^1^H NMR spectrum of thermally polarized gaseous DE scaled by a factor of 16. The spectra were acquired with eight signal accumulations. Hydrogenation of EVE with 6.5-fold excess of p-H_2_ was performed at 200 °C and 2.7 bar. Signal enhancement calculated using the signal of DE CH_3_ group was ε = 570, which corresponds to *p*_hyp_(^1^H) = 1.3%. (d,e) ^1^H FLASH (fast low-angle shot) MRI of diethyl ether vapor in a 5 mm NMR tube (axial view): (d) continuously flowing (5.1 mL/s gas flow rate) hyperpolarized DE and (e) thermally polarized DE under stopped-flow conditions. The gas pressure was 3.9 bar. The images were acquired at 9.4 T. Frequency offset was adjusted to the signal of CH_3_ group of DE. The FLASH imaging parameters: flip angle, 6°; number of averages, 2; acquisition time, 120 ms; matrix size, 128 × 16 (zero-filled to 128 × 128), field of view (FOV), 1.7 cm × 1.7 cm; spatial resolution, 0.1 mm × 1.1 mm. Reproduced with permission from ref (760). Copyright 2020 Wiley-VCH GmbH.

HET-PHIP has a clear potential to produce catalyst-free hyperpolarized liquids and gases for magnetic resonance applications, potentially including biomedical research and practice. For instance, rapid continuous production and imaging of substantial quantities of catalyst-free hyperpolarized gas^759^ is of interest in the context of MRI of lungs. A promising recent result is the hydrogenation of ethyl vinyl ether with p-H_2_ to produce and image hyperpolarized diethyl ether, a well-known anesthetic^760^ (Figure 88).

Polarization transfer from ^1^H to heteronuclei (section 3.11.2) is a viable strategy to extend the hyperpolarization lifetime in the liquid phase. For instance, 1.2% polarization of ^13^C nuclei was obtained for hydroxyethyl propionate in water,^761^ while heterogeneous hydrogenation of neurine^762,763^ was used to polarize ^15^N nuclei of a choline derivative up to 12.2%. In contrast, for hyperpolarized gases, this approach is impractical because for gases the *T*_1n_ times of heteronuclei are often significantly shorter than those of protons because of the dominating spin-rotation-induced relaxation. Despite that, ^1^H → ^13^C polarization transfer was successfully demonstrated for propane.^764^

**Figure 89:**
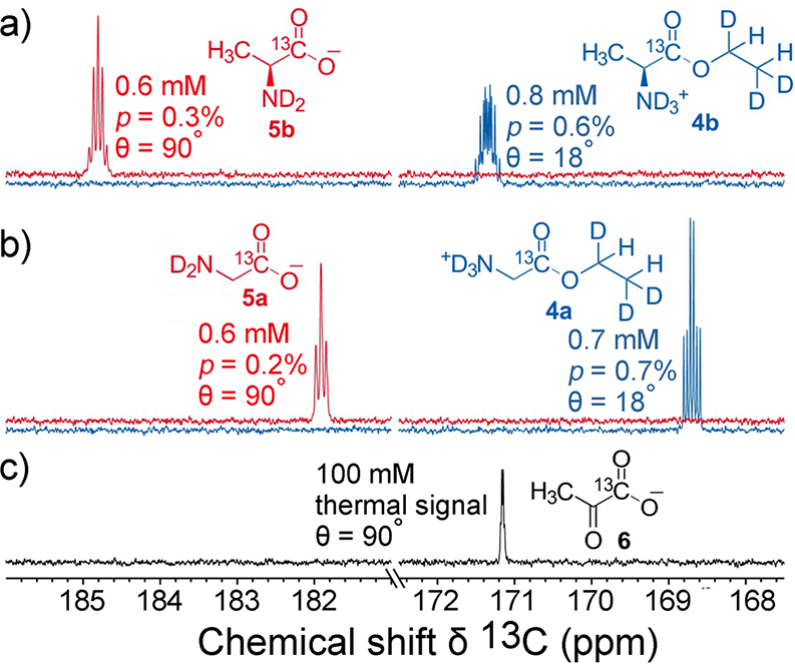
(a,b) Single-scan ^13^C{^2^H} spectra for the ^13^C-hyperpolarized compounds in the production of (a) alanine-1-^13^C and (b) glycine-1-^13^C. Spectra collected before ester cleavage (blue traces) were acquired with a 18° flip-angle pulse, whereas spectra after cleavage (red traces) were collected with a 90° flip-angle pulse. (c) The reference ^13^C NMR spectrum of pyruvate-1-^13^C in thermal equilibrium is shown for comparison. Reproduced with permission from ref (766). Copyright 2019 Wiley-VCH Verlag GmbH & Co. KGaA, Weinheim.

A rather powerful approach for extending the scope of substrates for PHIP is side arm hydrogenation (PHIP-SAH; section 3.11.2). The HET-PHIP-SAH approach was demonstrated for the production of a catalyst-free aqueous solution of hyperpolarized ethanol and acetate by hydrogenating vinyl acetate vapor and then hydrolyzing the hyperpolarized ethyl acetate by dissolving it in a basic aqueous medium.^765^ Aqueous solutions of hyperpolarized glycine and alanine amino acids were produced by hydrogenation of corresponding vinyl esters followed by polarization transfer to carbonyl ^13^C and subsequent ester hydrolysis.^766^ The ^13^C polarization levels achieved this way for the unprotected amino acids were ca. 0.3% (Figure 89).

Interestingly, hyperpolarization of water and OH protons of ethanol and methanol was observed when p-H_2_ was bubbled through a suspension of silica-encapsulated Pt_3_Sn intermetallic nanoparticles in the corresponding liquid.^767^

Importantly, HET-PHIP is not merely an extension of PHIP studies based on homogeneous hydrogenations, but is an important field of research in its own right. In fact, the overwhelming majority of industrial catalytic processes are heterogeneous. In particular, heterogeneous hydrogenation and hydrogenolysis processes are the key elements in the chemical and petrochemical industries, for instance, pharma and hydrotreating of oil. PHIP effects have already proven useful in the studies of mechanisms of catalytic processes involving metal complexes in homogeneous solution (section 3.11.2). Thus, successful demonstration of HET-PHIP effects^25,751,758^ opens a gateway toward the studies of mechanisms of industrially important processes.

Indeed, a mere observation of the PHIP effect already reveals an important mechanistic detail: an involvement of pairwise H_2_ addition to a catalytic center and to a substrate. There is thus an apparent disagreement with a fundamentally nonpairwise nature of the broadly accepted hydrogenation mechanism for MNP catalysts, attributed to Horiuti and Polanyi,^768^ via dissociative chemisorption of H_2_ on the metal surface followed by sequential addition of random H atoms to an unsaturated compound. Therefore, successful observation of HET-PHIP effects over a variety of MNP-based catalysts indicates that the relevant mechanistic details for such catalysts are far from being fully understood.

A lot of efforts are devoted to establishing the nature of catalytically active sites responsible for pairwise H_2_ addition on supported metal catalysts. To this end, various metals (e.g., Pt, Pd, Ir, Rh, Cu, Co, Ni) on a variety of solid supports were prepared and studied under a broad range of experimental conditions in both liquid- and gas-phase hydrogenations with p-H_2_.^25,751^ Nevertheless, the possible nature of the active sites involved in pairwise H_2_ addition on supported metal catalysts essentially remains an open question. One recent study^769^ discusses correlation of pairwise selectivity with preferential syn vs anti addition of H_2_ to triple C–C bonds; however, cis–trans isomerization of the double bond in the hydrogenation product may significantly affect the interpretation of such experiments.

**Figure 90:**
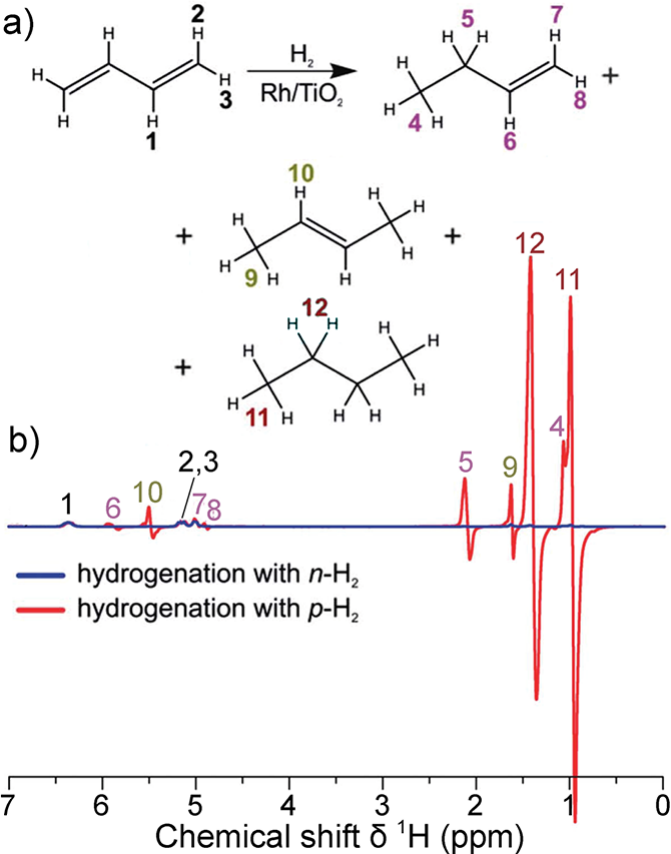
(a) Reaction scheme of 1,3-butadiene hydrogenation. (b) ^1^H NMR spectra acquired during 1,3-butadiene hydrogenation with normal hydrogen (blue trace) and parahydrogen (red trace) over a Rh/TiO_2_ catalyst prepared from rhodium nitrate and calcined at 600 °C. Reproduced from ref (770) with permission from the Royal Society of Chemistry.

At present, ^1^H polarization levels commonly obtained with HET-PHIP are in general lower than those in homogeneous hydrogenations (section 3.11.2). The highest catalytic activity is achieved with supported MNPs of platinum group metals (Pt, Pd, Ir, Rh), but their PS values usually do not exceed 2–3% and vary significantly depending on the catalyst and the experimental conditions used. Nevertheless, higher PS values are sometimes achieved; for instance, in hydrogenation of 1,3-butadiene (Figure 90) and propylene over optimized Rh/TiO_2_ catalyst, the PS values were 4.5 (*X* ∼ 15%) and 7% (*X* ∼ 22%), respectively^770^ Capping the surface of MNPs with ligands possessing a thiol functionality^761,771,772^ was reported to increase polarization levels, but at the expense of significantly reduced conversion (e.g., *p*_hyp_ up to 60% at *X* < 1% for 4-mercaptobenzoic acid^771^).

Apart from supported metals, HET-PHIP effects were also successfully demonstrated with other catalyst types.^25,751^ Among metal oxides, CeO_2_ demonstrated remarkable selectivity in semihydrogenation of alkynes to alkenes and an interesting dependence of behavior on the shape of its nanocrystals.^773^ Molybdenum carbides are extensively studied in catalysis in the context of the search for replacing noble-metal catalysts. For Mo_2_C catalysts, the presence of hexagonal close-packed (HCP) phase was essential to achieve selectivities to pairwise H_2_ addition comparable to those of supported MNPs.^774^

In addition to hydrogenation of various unsaturated compounds, extending the HET-PHIP approach to other catalytic processes involving H_2_ is also of major interest.^25,751^ In particular, it was used to reveal the differences in the mechanisms of thiophene hydrodesulfurization over MoS_2_/γ-Al_2_O_3_ and Pt/TiO_2_ catalysts.^775^ Thus, similar processes (e.g., hydrodenitrogenation, hydrodeoxygenation) may be potentially addressed as well. Pronounced HET-PHIP effects were also reported in the oligomerization of acetylene over Pd MNPs of different shapes and sizes supported on SiO_2_.^776^ At the same time, attempts to observe HET-PHIP effects for higher hydrocarbons and oxygenates produced from CO and H_2_ via Fischer–Tropsch synthesis (FTS) were so far unsuccessful.

**Figure 91:**
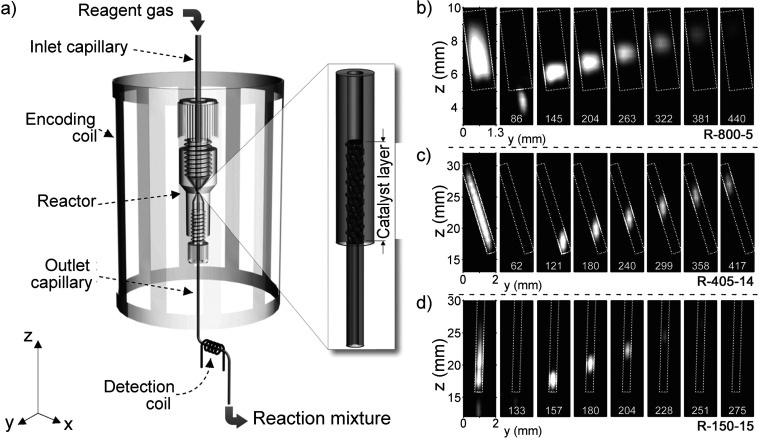
(a) Schematics of the remote detection MRI experiment. Catalyst bed (reactor) is placed inside the encoding RF coil which, in combination with the gradient coils, is used to perform spatial encoding of the ^1^H NMR signal of the continuously flowing gaseous mixture of p-H_2_ and propylene. The ^1^H NMR signal detection is performed with a detection RF coil located downstream. (b–d) Remote detection time-of-flight images encoded in the yz plane for Rh/SiO_2_ catalyst beds (b) 800 μm in diameter and 5 mm long (R-800-5); (c) 405 μm in diameter and 14 mm long (R-405-14); (d) 150 μm in diameter and 15 mm long (R-150-15). The images visualize the ^1^H NMR signal of hyperpolarized propane. The experiments were performed at 60 °C. Gas travel times between the encoding and detecting RF coils are indicated in the panels in milliseconds. The leftmost images are obtained by coadding all images obtained for various travel times. The catalyst bed regions are outlined with white dashed lines. The complete data set for each catalyst bed was acquired in 13 min with a time resolution of 12 ms and a spatial resolution of 160–250 μm in the y direction and 0.62–2.2 mm in the z direction. Reproduced with permission from ref (779). Copyright 2012 WILEY-VCH Verlag GmbH & Co. KGaA, Weinheim.

Another direction of HET-PHIP application is its use in the spatially resolved studies of operating model reactors.^25,777,778^ Sensitivity enhancement is particularly advantageous for imaging of gases with their low spin density compared to liquids. HET-PHIP has proven useful for MRI studies of operating (micro) reactors, including small packed catalyst beds with diameters ranging from several mm down to 150 μm (Figure 91),^779^ and catalyst layers deposited on the walls of cylindrical glass structures.^25,780^ The resulting images can successfully visualize mass transport and product distribution in operating model reactors.

**Figure 92:**
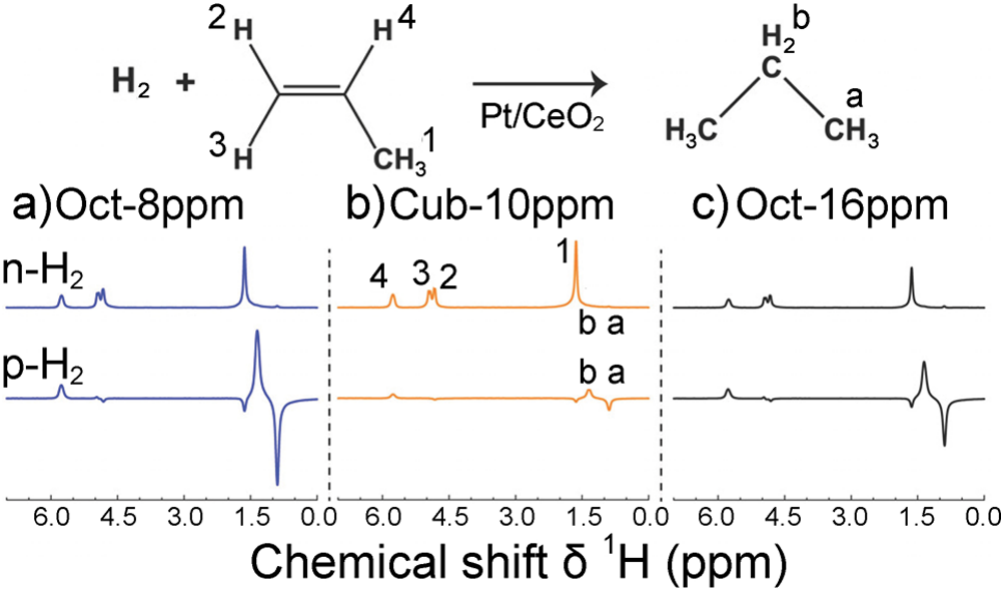
(a–c) ^1^H NMR spectra of thermally polarized (n-H_2_, top) and hyperpolarized (p-H_2_, bottom) samples in the hydrogenation of propylene to propane over catalysts (10 mg) comprising (a) 8 ppm Pt on CeO_2_ octahedra (Oct-8 ppm), (b) 10 ppm Pt on CeO_2_ cubes (Cub-10 ppm), and (c) 16 ppm Pt on CeO_2_ octahedra (Oct-16 ppm) at 300 °C. The flow rates were 365/30/105 mL/min N_2_/H_2_/propene. All spectra are shown on the same vertical scale. The reaction scheme is shown above the spectra. Adapted with permission from ref (782). Copyright 2020 Wiley-VCH GmbH.

One of the important trends in modern heterogeneous catalysis is the exploration of catalysts with well-defined isolated metal centers, for instance single-atom catalysts (SAC) which comprise individual metal atoms (or sometimes small clusters) stabilized on a suitable porous support. The perceived advantages of SACs include the ultimate utilization of expensive noble metals and the possibility to combine the advantages of homogeneous and heterogeneous catalysts. The semblance between SAC and homogeneous catalysts implies that they may be expected to provide high levels of pairwise H_2_ addition selectivity. Indeed, gold-based SAC supported on multiwalled carbon nanotubes, Au_*n*_/MWCNT (*n* = 1 or a few), demonstrated rather significant selectivity in pairwise H_2_ addition to 1,3-butadiene (PS = 7–11% and PS = 3–6% for butane and 1-butene, respectively; *X* ∼ 0.5%).^781^ Single Pt-atom catalysts, Pt_1_/CeO_2_, containing only 8–16 ppm of Pt were shown to provide pairwise selectivity of up to ca. 6% (Figure 92).^782^

**Figure 93:**
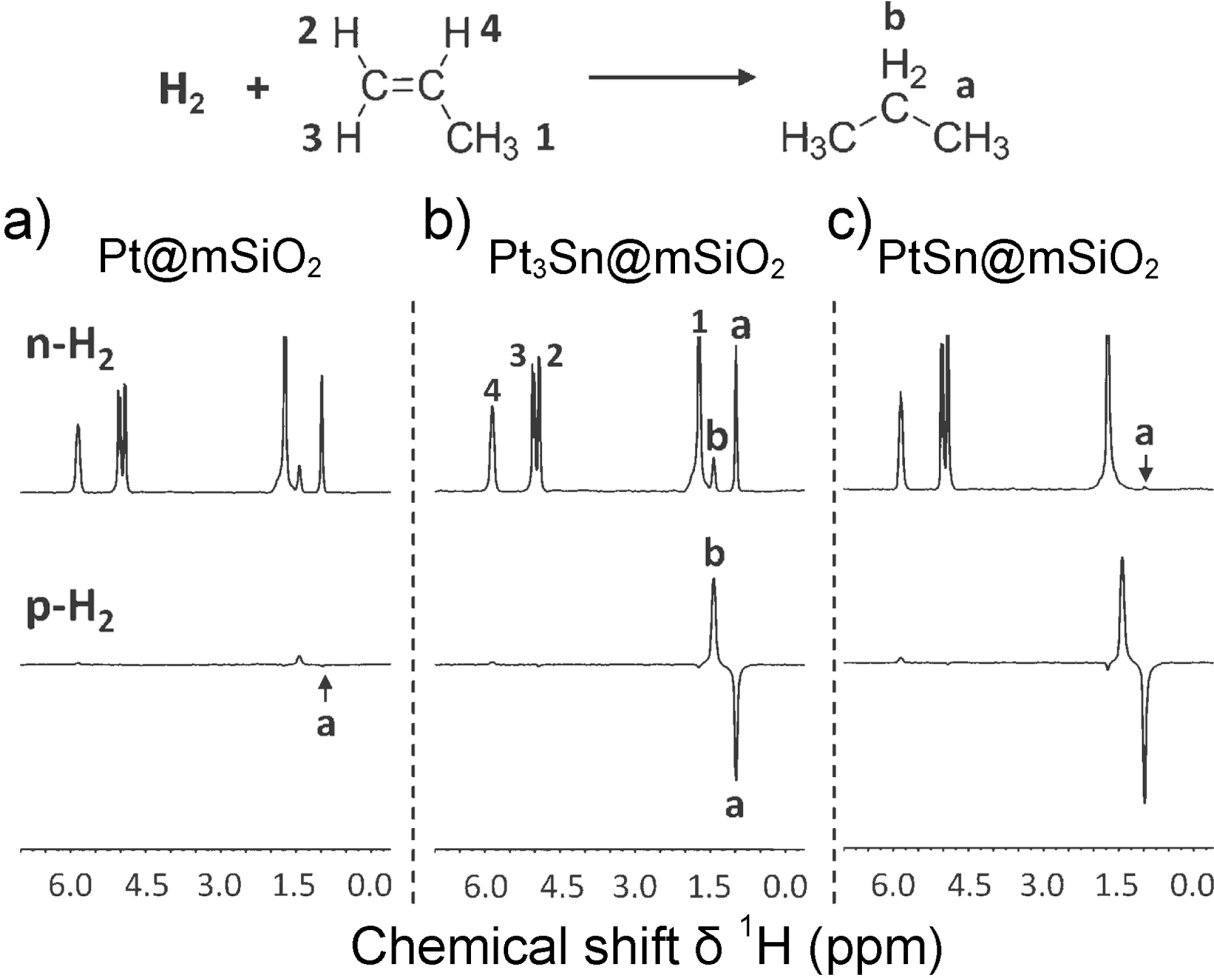
^1^H NMR spectra of thermally polarized (top) and hyperpolarized (bottom) reactor effluent obtained using 10 mg of (a) Pt@mSiO_2_, (b) Pt_3_Sn@mSiO_2_, and (c) PtSn@mSiO_2_ at 300 °C. The reactant flow rates were 120 mL/min H_2_, 210 mL/min propylene, and 70 mL/min N_2_. All spectra are displayed on the same vertical scale. Reproduced with permission from ref (783). Copyright 2017 Wiley-VCH Verlag GmbH & Co. KGaA, Weinheim.

**Figure 94:**
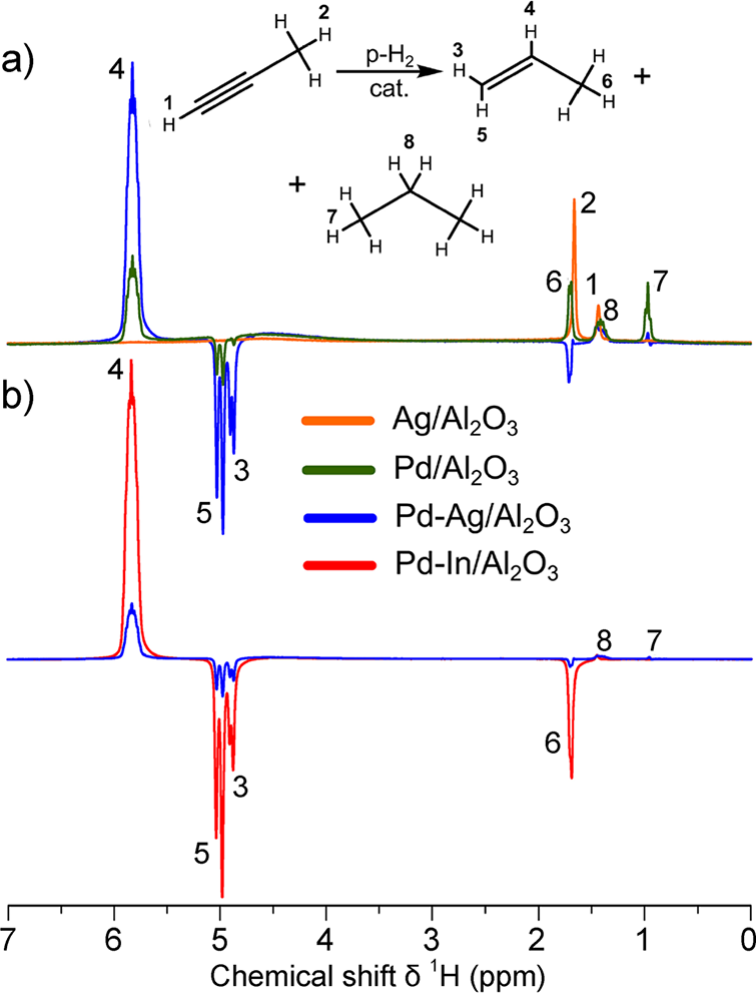
(a) ^1^H NMR ALTADENA spectra acquired during propyne hydrogenation with parahydrogen over Ag/Al_2_O_3_ (orange trace), Pd/Al_2_O_3_ (green trace), and Pd-Ag/Al_2_O_3_ (blue trace) catalysts. The reaction temperature was 200 °C, and the total gas flow rate was 3.8 mL/s. All spectra were acquired with eight signal accumulations and are presented on the same vertical scale. (b) Comparison of Pd-Ag/Al_2_O_3_ (blue trace; the same spectrum as in (a)) and Pd-In/Al_2_O_3_ (red trace) catalysts. Reaction scheme of propyne hydrogenation is shown above the spectra. Reproduced from ref (785). Copyright 2021 The Authors.

An alternative strategy is the dilution of an active metal in order to both localize the catalytically active site and prevent diffusive migration of activated hydrogen away from it. In particular, in bimetallic catalysts such as single-atom alloys and intermetallic compounds, an active metal atom is surrounded by another metal which is far less active in the reaction. Examples include propylene hydrogenation (PS ∼ 11%, *X* < 1%) over Pt–Sn intermetallic NPs with a mesoporous silica shell (Figure 93),^769,783^ Pd–Au core–shell systems,^784^ and propyne hydrogenation (PS ∼ 9.3%, *X* ∼ 20%) over intermetallic Pd–In/Al_2_O_3_ catalyst (Figure 94).^785^

**Figure 95:**
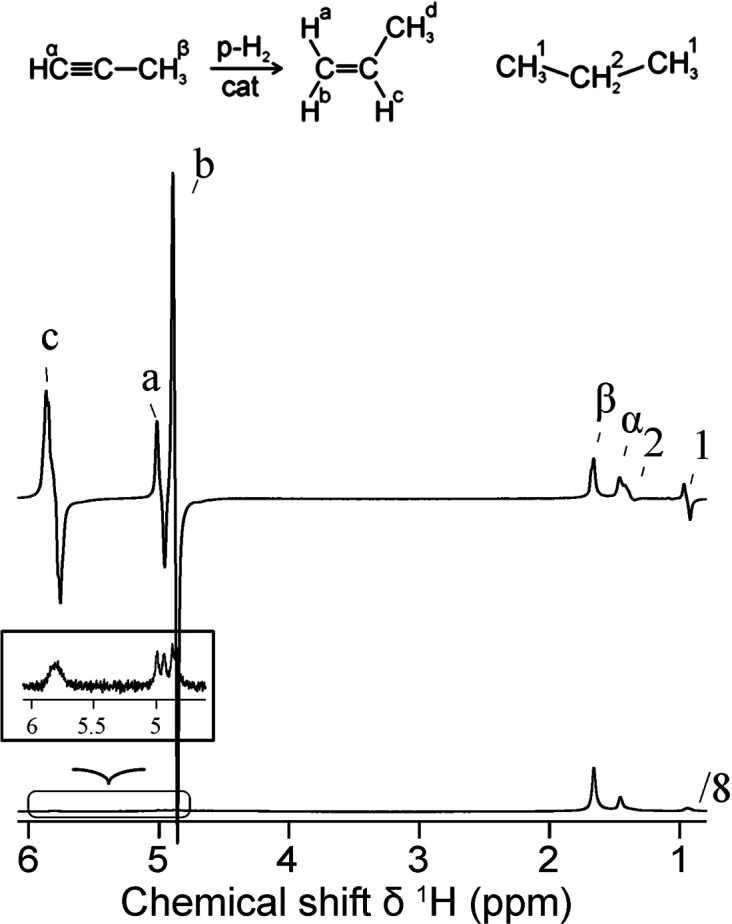
^1^H NMR spectra detected during the hydrogenation of propyne with p-H_2_ at 120 °C over an immobilized Ir metal complex synthesized from [Ir(COD) Cl]_2_ (COD = 1,5-cyclooctadiene) by covalently binding an Ir metal center of the complex to PPh_2_– functional groups of the linker chains on the surface of functionalized silica gel. The spectrum detected in a PASADENA experiment (top trace; 16 signal accumulations) shows characteristic enhanced antiphase multiplets. ^1^H NMR spectrum at thermal equilibrium (bottom trace) was recorded for the same reaction mixture after relaxation of the hyperpolarized products with 128 accumulations. The spectra are scaled accordingly and are presented on the same vertical scale; the inset shows the vertically expanded part of the spectrum acquired at thermal equilibrium to make signals of the product propylene visible. The reaction scheme is shown in the top part of the figure. Adapted from ref (789) with permission from the Royal Society of Chemistry.

Exploration of immobilized transition metal complexes, the approach that was used in the initial demonstration of HET-PHIP feasibility,^757^ is also worth pursuing further. So far, they demonstrate propensity to metal leaching^786^ and significant structural transformations^787^ in liquid-phase reactions and chemical degradation in gas-phase processes.^788^ However, recent results demonstrated^789^ that Ir-based catalysts may be more efficient (PS ∼ 9%, *X* ∼ 10%) and stable (Figure 95). Silica-supported vanadium oxo organometallic complex demonstrated signal enhancements of 200–300- and 1300-fold in the gas-phase hydrogenation of propylene and propyne, respectively.^754^ Instead of tethering a metal complex to a support, it may be more efficient to design a catalytic center on an oxide surface using surface organometallic synthesis. Interestingly, even Co(II)^790^ and Cr(III)^791^ surface sites showed HET-PHIP effects despite their paramagnetic nature.

Curiously enough, and despite the expectations, even the advanced types of heterogeneous catalysts mentioned above cannot so far achieve polarization levels demonstrated with transition metal complexes in homogeneous PHIP. It is possible that, in addition to pairwise selectivity, the observed polarization levels are also largely governed by the relaxation-induced losses in reaction products and intermediates - an issue which calls for an in-depth study.

**Figure 96:**
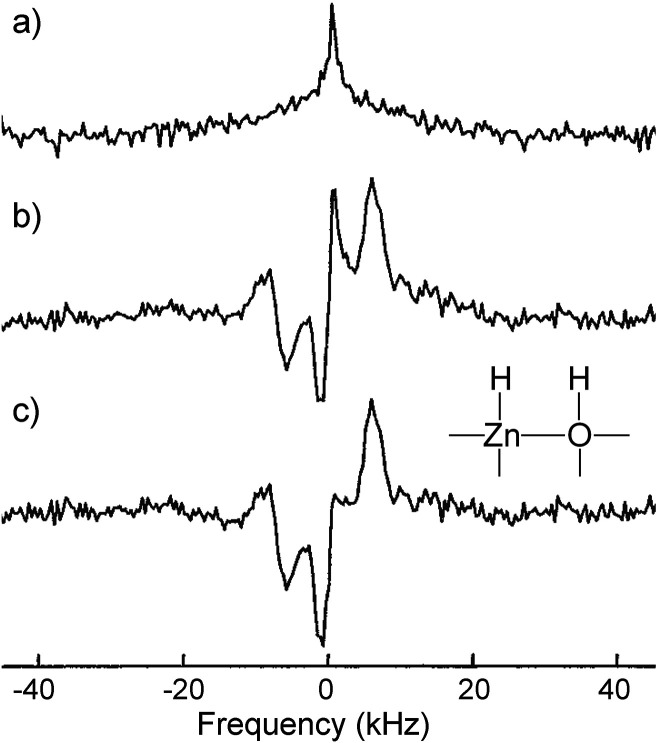
(a) ^1^H NMR spectrum acquired immediately after a nominal 50 ms burst of n-H_2_ delivered to polycrystalline ZnO. (b) Spectrum taken under the same conditions as in (a) but using p-H_2_ instead of n-H_2_. Strong PASADENA enhancement is seen for sites not detected in the n-H_2_ experiment. (c) Pure PASADENA spectrum obtained by taking the weighted difference of (a) and (b). The inset depicts the suggested structure of the detected surface species. Reproduced with permission from ref (795). Copyright 2001 American Chemical Society.

For a detailed mechanistic understanding of catalytic processes, direct detection of short-lived reaction intermediates at low concentrations can be a major advance. NMR signal enhancement provided by PHIP can make this a reality, as demonstrated convincingly with metal complexes in solution (section 3.11.2). However, for heterogeneous processes, this is still an unaccomplished and challenging task. To this end, MAS NMR with a continuous flow of gaseous reactants was attempted in hydrogenation of unsaturated gaseous hydrocarbons over several heterogeneous catalysts.^792−794^ While polarized products adsorbed on the porous support were observed, no reaction intermediates could be detected in such studies so far. At the same time, heterolytic activation of p-H_2_ on the surface of ZnO was reported to exhibit an antiphase PHIP pattern (Figure 96) in the wide-line ^1^H NMR spectra provisionally attributed to the neighboring Zn–H and O–H moieties.^795^

The key practical problem to solve in HET-PHIP is the relatively low level of polarization achieved with the majority of heterogeneous catalysts compared to their homogeneous counterparts, often combined with low amounts of product produced. Development of more efficient catalysts is hampered largely because an understanding of the mechanisms of pairwise H_2_ addition on MNP-based catalysts is lacking. A major complicating issue well-known for such catalysts is the existence of a variety of different active sites on their surfaces, both inherent to the initial catalyst structure and possibly produced in the course of reaction. Further studies are thus required to establish the nature of active sites on MNPs responsible for pairwise H_2_ addition.

As mentioned in section 2.2.3, nuclear spin isomers (NSIMs) of polyatomic molecules could be used similarly to p-H_2_ for spin hyperpolarization, and could dramatically expand the range of applications of the technique. In particular, reactions not involving H_2_ (e.g., oligomerization, metathesis and other key industrial processes) could become accessible, and higher hyperpolarization levels may be achievable. Currently, practical NSIM enrichment procedures for polyatomic molecules do not exist. Nevertheless, the feasibility of NSIM enrichment for ethylene by hydrogenating acetylene with parahydrogen and its use for NMR signal enhancement in a subsequent chemical reaction has been demonstrated,^63^ providing a glimpse of what the future may hold.

#### 3.11.4 Signal Amplification by Reversible Exchange

##### 3.11.4.1 The Technique

**Figure 97:**
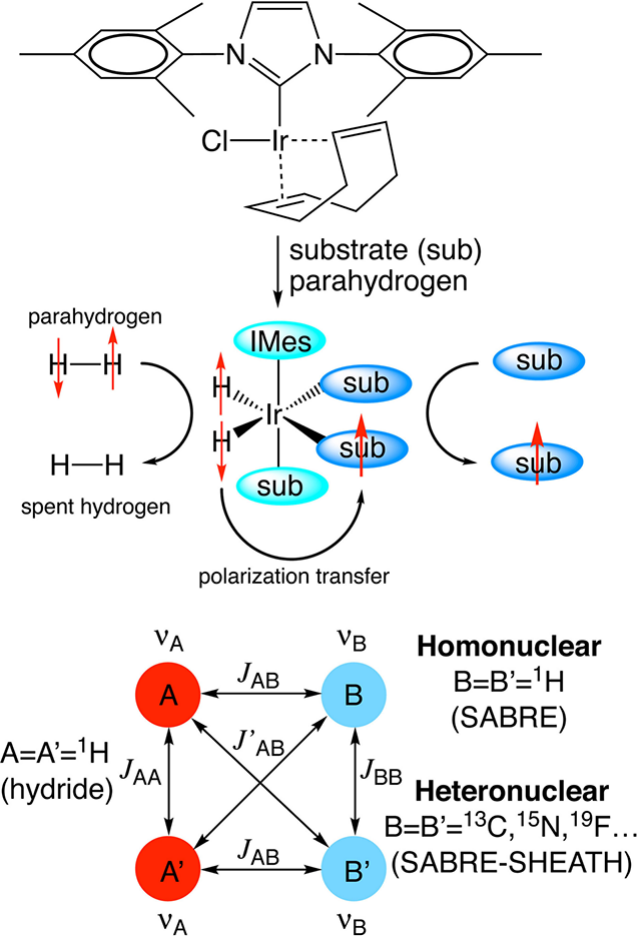
Overall schematic representation of SABRE: simultaneous chemical exchange of parahydrogen and to-be-hyperpolarized substrate (sub) leads to formation of “free” hyperpolarized substrate. Note that the axial ligand positions are not exchangeable. Once the catalyst is activated using parahydrogen and substrate, the hexacoordinate complex facilitates polarization transfer in the equatorial plane. The AA′BB′ spin system and relevant spin–spin couplings of the four ligands in the equatorial plane are shown at the bottom (note that spins A and A′ are chemically equivalent, and so are spins B and B′; the magnetic equivalence is broken by the difference in the corresponding A–B spin–spin couplings: J_AB_ and J′_AB_). The corresponding spin–spin couplings with axial ligands are negligible. The *N*-heterocyclic carbene ligand, IMes, is shown explicitly in the precatalyst structure at the top.

**Figure 98:**
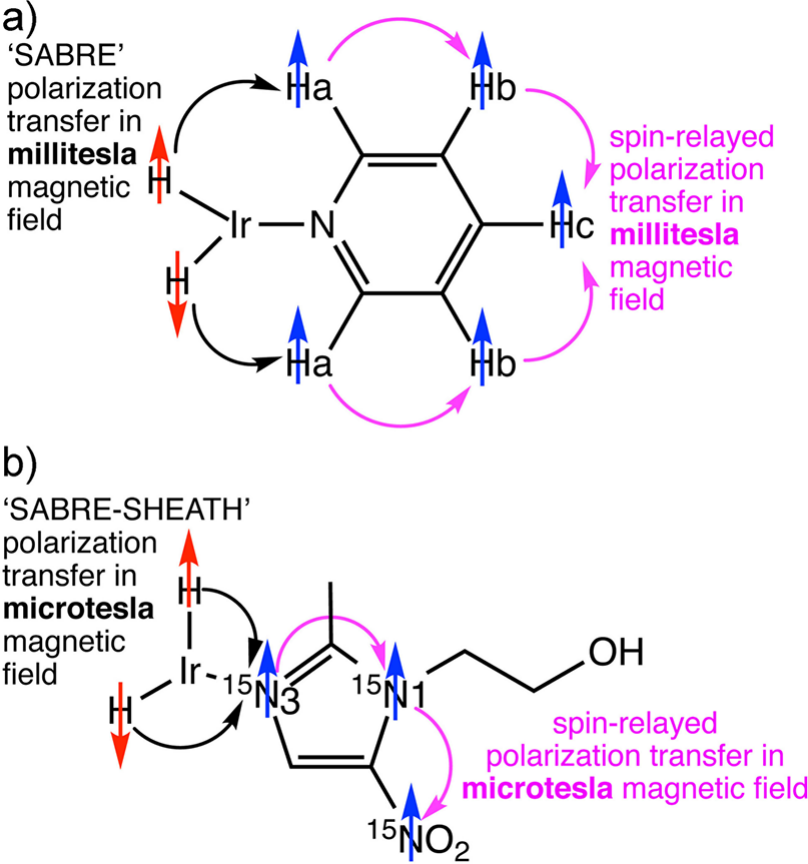
(a) Schematic representation of SABRE polarization transfer via ^1^H-^1^H spin-relays in a millitesla magnetic field to enable spontaneous polarization transfer from p-H_2_-derived hydrides through all proton sites of pyridine substrate molecule via 3- and 4-bond ^1^H-^1^H spin–spin couplings. (b) Schematic representation of SABRE-SHEATH polarization transfer via ^15^N–^15^N spin-relays in a microtesla magnetic field to enable spontaneous polarization transfer of p-H_2_-derived hyperpolarization through all ^15^N sites of [^15^N_3_]metronidazole molecule via 2-bond ^15^N–^15^N spin–spin couplings. In (a,b), not all ligands are shown to simplify the structures (cf. Figure 97). (a) Adapted with permission from ref (805). Copyright 2021 Wiley-VCH GmbH.

Signal amplification by reversible exchange (SABRE) relies on simultaneous reversible binding of p-H_2_ and to-be-hyperpolarized substrate to a metal complex, Figure 97.^26,796^ The SABRE approach, pioneered by Duckett and co-workers, does not require an actual hydrogenation of unsaturated compound to produce hyperpolarization, and is therefore sometimes referred to as a non-hydrogenative variant of PHIP (section 3.11.2).^796−799^ It is proposed and commonly accepted that polarization transfer from p-H_2_-derived hydrides to the nuclear spins of substrate in a transient complex is accomplished via spin–spin couplings when the condition for spin level anticrossing (LAC) is met.^800^ However, a more recent work shows that efficient SABRE polarization transfer can happen using pulsed magnetic fields that put a spin system far away from the LAC condition.^801^ In the original pioneering demonstration, the LACs were conveniently generated at a magnetic field of 2–10 mT for efficient transfer of p-H_2_-based singlet spin order to protons of an exchangeable substrate molecule.^796,802^ The substrate protons that are coupled to p-H_2_-derived hydrides via spin–spin interactions are hyperpolarized first, and the network of ^1^H-^1^H spin–spin couplings in the substrate molecule propagates the hyperpolarized state to other substrate protons, Figure 98a.^797,803^ Proton polarization values *p*_hyp_(^1^H) in excess of 50% have been achieved.^804^

The SABRE approach enjoyed a number of substantial extensions since its invention, which significantly expanded the range of amenable substrates. The first class of compounds hyperpolarized with SABRE was based on N-binding to an Ir-based complex: most notably nitrogen heterocycles on hexacoordinate Ir-based catalyst with an *N*-heterocyclic carbene ligand (Figure 97).^802^ Sulfur-,^806^ phosphorus-,^807^ and oxygen-containing^808,809^ transiently binding molecules have been shown to successfully undergo SABRE hyperpolarization. Recently Co-based complexes have been shown to facilitate SABRE process, too.^810^

Moreover, SABRE of substrates possessing exchangeable protons leads to net hyperpolarization of bulk protons via the effect termed SABRE-Relay.^811^ This way, polarization can be propagated to molecules which do not directly bind to a SABRE catalyst, demonstrating the utility of SABRE to hyperpolarize a wide range of targets that can participate in proton exchange processes.^812^

Furthermore, it was also demonstrated^807,813,814^ that LACs between p-H_2_-derived hydrides and a heteronucleus of a substrate can be successfully established at magnetic fields below 1 μT.^813,815^ This approach, termed SABRE-SHEATH (SABRE in shield enables alignment transfer to heteronuclei),^813^ was extended to ^15^N,^813^^13^C,^816^^19^F,^817^^31^P,^807^ and other spin-1/2 nuclei. Polarization of ^15^N nuclei *p*_hyp_(^15^N) > 50% was demonstrated.^818^ The use of microtesla magnetic fields also allows establishing intramolecular spin–spin relays^803^ between ^15^N–^15^N and ^15^N–^13^C coupled spin pairs that enable spontaneous propagation of p-H_2_-derived hyperpolarization throughout a substrate molecule via the network of heteronuclear spin–spin couplings, Figure 98b.^819^

The field requirement for “canonical” homonuclear SABRE and heteronuclear SABRE-SHEATH is provided by the analysis of requirements for LACs matching conditions in a AA′BB′ four-spin system (Figure 97) formed in the equatorial plane of the activated SABRE catalyst by the two hydrides and a pair of substrate spins experiencing spin–spin couplings with the hydrides:^813^
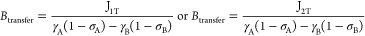
7where J_1T_ = ±(J_AA_ – J_BB_), J_2T_ = ±(J_AA_ + J_BB_ – (J_AB_ + J′_AB_)/2), and σ_A_ and σ_B_ are chemical shifts of the two nuclei. The matching conditions for a three-spin system are similar.^815^

**Figure 99:**
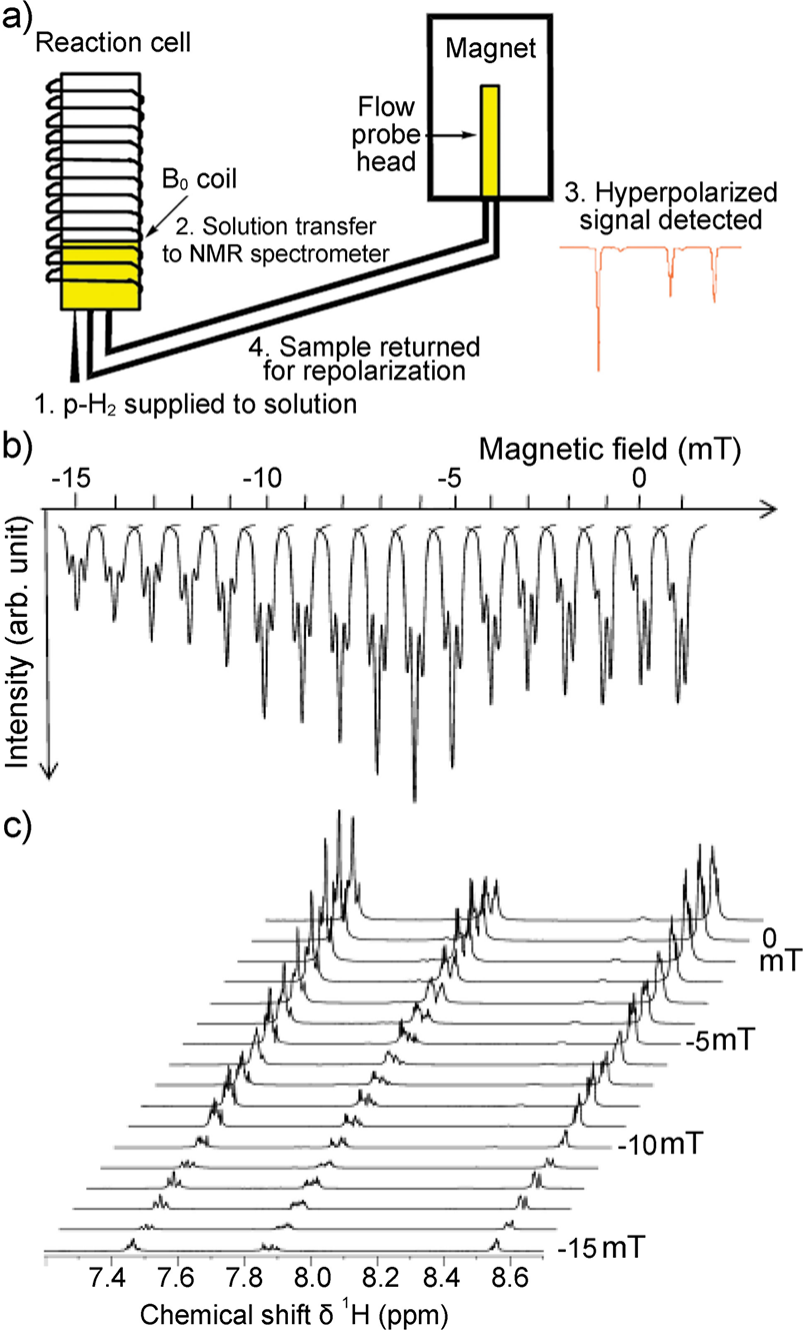
(a) Schematic diagram of the automated hyperpolarized sample preparation process featuring an external reaction cell, an NMR flow probe, and a *B*_0_ polarization coil. (b,c) ^1^H NMR signal response profiles of pyridine measured as a function of the external polarization field for (b) the hydrogen atom in the para position of the pyridine ring, which shows longitudinal magnetization, and (c) longitudinal two-spin order terms spanning the three sites. Reproduced with permission from ref (802). Copyright 2011 American Chemical Society.

It follows that, for example, for a homonuclear spin system of ^1^H nuclei (γ_A_ = γ_B_ = γ(^1^H) = 42.6 MHz/T), and for J_1T_ ∼ 9 Hz and (σ_B_ – σ_A_) = 32 × 10^–6^ (32 ppm), an optimum *B*_transfer_ is ∼6.6 mT.^813^ For a heteronuclear case, e.g., when nucleus B is ^15^N, the optimum transfer field is ∼0.4–0.6 μT.^813^Figure 99 demonstrates an example of ^15^N SABRE-SHEATH for [^15^N_3_]metronidazole with a clear maximum in polarization at *B*_0_ ∼ 0.6 μT.

It was also shown that singlet spin order of p-H_2_-derived hydrides can be employed to create singlet states on the nuclear spin pairs in a substrate;^820−822^ as a result, it was possible to extend the lifetime of ^15^N–^15^N singlet states to over 20 min.^821^

The account of SABRE at low magnetic fields would be incomplete without mentioning the studies performed at subnanotesla fields.^823^ A particular feature of this experiment is that NMR detection of polarized pyridine was accomplished in situ and at zero- to ultralow-field (ZULF) conditions with an optically pumped atomic magnetometer inside a magnetic shield. An interesting recent application of SABRE-ZULF is a continuous production of relatively large quantities of hyperpolarized material, on the order of several cm^3^/min of hyperpolarized solution with >10^17^ polarized ^15^N nuclei of a labeled material per cm^3^, with prospects of increasing this much further in the future. This holds great promise for applications in chemistry, biomedicine, and fundamental physics.^824^ Currently investigated applications of in situ SABRE-ZULF include the ZULF NMR study of systems undergoing chemical exchange^395^ as well as biomolecular analysis.^825^ The polarization method used in SABRE-ZULF experiments is essentially the SABRE-SHEATH approach described above.

In addition to the use of millitesla, microtesla and subnanotesla fields, SABRE has been demonstrated at high magnetic fields too, where the polarization transfer is accomplished via cross-relaxation.^826^

Besides the use of static magnetic fields to establish LACs, radiofrequency (RF) pulses can also be employed to create LACs between p-H_2_-derived hydrides and ^1^H, ^15^N,^827^ or ^19^F^828^ spins of an exchangeable substrate. Several RF pulse sequences were developed including LIGHT-SABRE (low-irradiation generation of high-tesla SABRE),^827^ SLIC-SABRE (spin-lock induced crossing SABRE),^829^ RF-SABRE,^830^ QUASR-SABRE (quasi-resonance SABRE),^831^ etc. All in all, SABRE can be induced by a wide range of approaches involving static and alternating magnetic fields, clearly demonstrating the versatility of this technique.

##### 3.11.4.2 Practical Aspects

**Figure 100:**
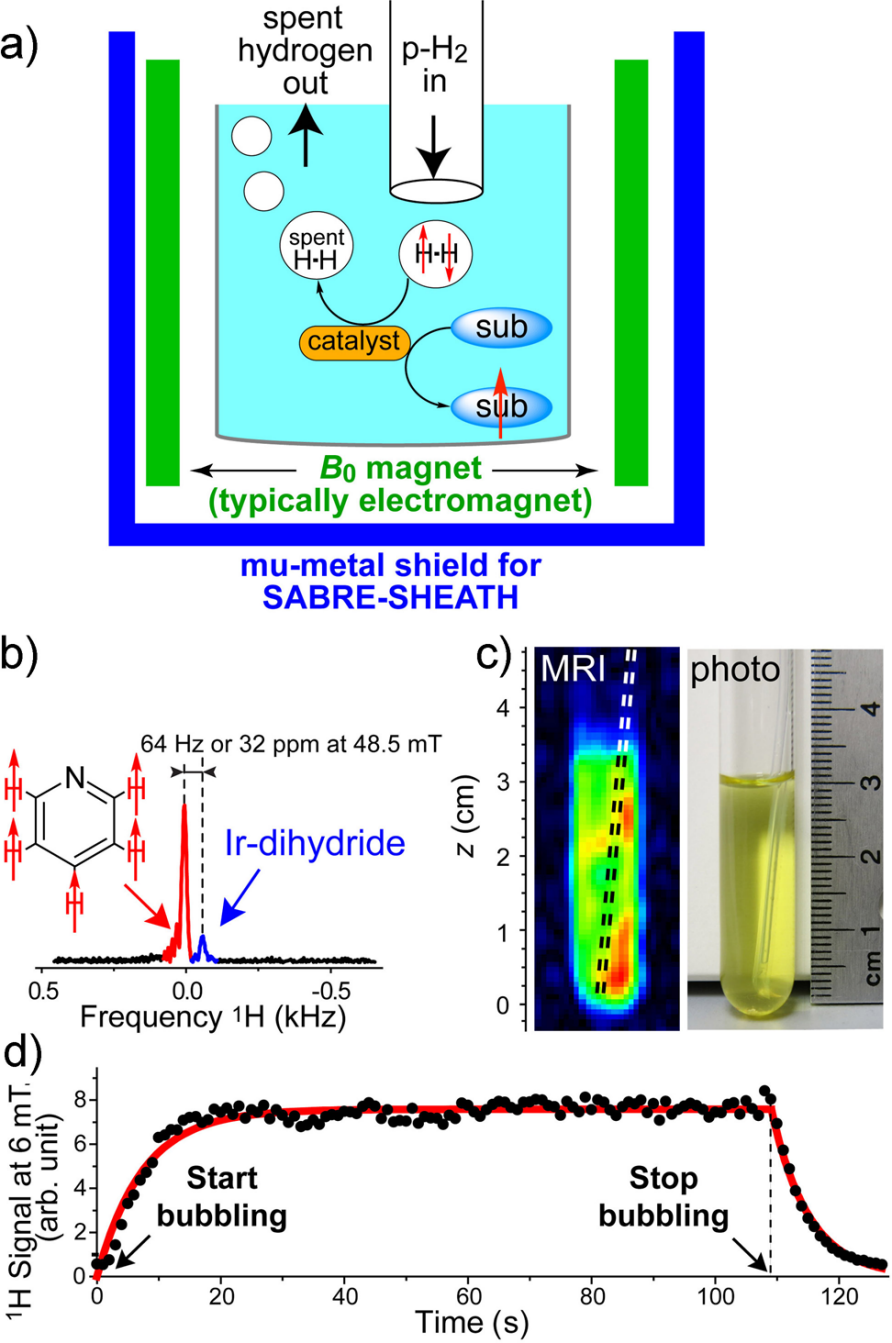
(a) Typical experimental setup showing delivery of p-H_2_ gas to the sample (the bubbles delivered by a catheter as shown, or sample shaking). The exchange at the catalyst leads to production of spent H_2_ (i.e., H_2_ with a reduced p-H_2_ enrichment level) exiting the solution (the nominal conversion of the para state, denoted as ↑↓, to the “spent” state in the figure is shown to emphasize that parahydrogen excess in solution is depleted upon the exchange; note, however, that an excess ortho state cannot be created via this process). An electromagnet is typically employed to create a static magnetic field for optimum polarization transfer via SABRE; a magnetic shield is required for SABRE-SHEATH experiments in microtesla magnetic fields. The produced hyperpolarization can be detected in situ or ex situ at higher magnetic fields using sample transfer. (b) In situ NMR spectroscopy of proton-hyperpolarized pyridine by SABRE at 48.5 mT. (c) Corresponding MR image (acquired at 48.5 mT) and sample photograph, demonstrating that the entire sample is indeed hyperpolarized. (d) In situ monitoring of SABRE process polarization build-up and decay corresponding to starting and stopping of p-H_2_ delivery (via bubbling) performed at 6 mT. (b–d) Adapted with permission from ref (833). Copyright 2014 WILEY-VCH Verlag GmbH & Co. KGaA, Weinheim.

Similar to PHIP experiments based on homogeneous (section 3.11.2) and heterogeneous (section 3.11.3) hydrogenation processes, the key requirement for SABRE hyperpolarization technique is access to p-H_2_, which is typically produced at cryogenic temperatures using a p-H_2_ generator,^22^ as described in more detail in section 3.11.1. In addition, SABRE instrumentation must: (1) provide efficient contact between SABRE catalyst and p-H_2_, and (2) establish LACs during simultaneous chemical exchange of p-H_2_ and to-be-hyperpolarized substrate to achieve polarization transfer (Figure 99, Figure 100).^832,833^ Therefore, the core component of any SABRE polarizer is the reaction vessel, where simultaneous chemical exchange of p-H_2_ and to-be-hyperpolarized substrate occurs on the metal atom of a suitable metal complex.

To date, p-H_2_ delivery has been accomplished via three approaches.^832^ In one approach, an outgassed solution of catalyst and the to-be-hyperpolarized substrate is loaded in a container (e.g., a 5 mm NMR tube), which is pressurized with p-H_2_ gas. The sample is next shaken vigorously to establish good mixing between the gas and liquid phases.^834^ The key advantage of this approach is fast (on the time scale of a few seconds) and efficient saturation of the solution with p-H_2_. To repeat the SABRE experiment, the sample needs to be outgassed and repressurized with p-H_2_, thereby representing a shortcoming of this approach.^796,799^

In the second approach, a catheter is placed inside the solution to provide a flow of p-H_2_ (Figure 100) regulated by mass-flow controllers (MFCs)^819^ or needle valves.^835^ Parahydrogen is bubbled through the solution continuously for replenishment with fresh p-H_2_ during the polarization buildup of the SABRE process, while the spent hydrogen is exhausted. The gas flow is ceased after the polarization process is finished, and the flow can be restarted later. As a result, this approach can enable quick (in less than a second) rehyperpolarization of a SABRE sample for systematic studies of relaxation dynamics and optimization of polarization efficiency.^819^ A variant of this approach employs a reaction cell through which p-H_2_ is blown. This cell is placed in the *B*_0_ coil to produce LACs,^836^ and the hyperpolarized solution is continuously circulated between the reaction cell and the NMR detector (Figure 99a);^802^ as a result, it becomes possible to optimize experimental parameters, e.g., the magnetic field *B*_0_ (Figure 99c).^802^ The disadvantage of this approach is the relatively large size of bubbles delivered by catheters resulting in suboptimal dissolution of fresh p-H_2_ and its transfer to the catalyst.

The third approach employs hollow fibers for p-H_2_ dissolution in the liquid phase.^837^ Parahydrogen is infused continuously while used H_2_ is removed at the same time.^838^ This approach mitigates the shortcomings of the above-mentioned bubbling technique.

The hardware required to create LACs can be divided in three categories. The first kind employs an electromagnet or a permanent magnet capable of creating static magnetic fields of a few millitesla to establish LACs required for polarization transfer.^802^ Second, in the SABRE-SHEATH approach which requires submicrotesla fields (i.e., fields lower than the Earth’s magnetic field by at least 2 orders of magnitude), the use of a μ-metal magnetic shield is typically required.^814^ The desired field can then be created by the use of a calibrated electromagnet placed inside the shield (Figure 100).^819^ Third, for creating LACs using RF pulses, the RF irradiation capabilities of NMR spectrometers and MRI scanners can be employed.^827,830^

Of note, in some applications of SABRE (see below), sensing may be performed using the NMR signatures of hyperpolarized metal hydride complexes which can be efficiently hyperpolarized via the exchange of p-H_2_ on the metal complex irrespective of the applied magnetic field. As a result, for this group of applications, there is no need to induce LACs, mitigating the requirement for the instrumentation.

**Figure 101:**
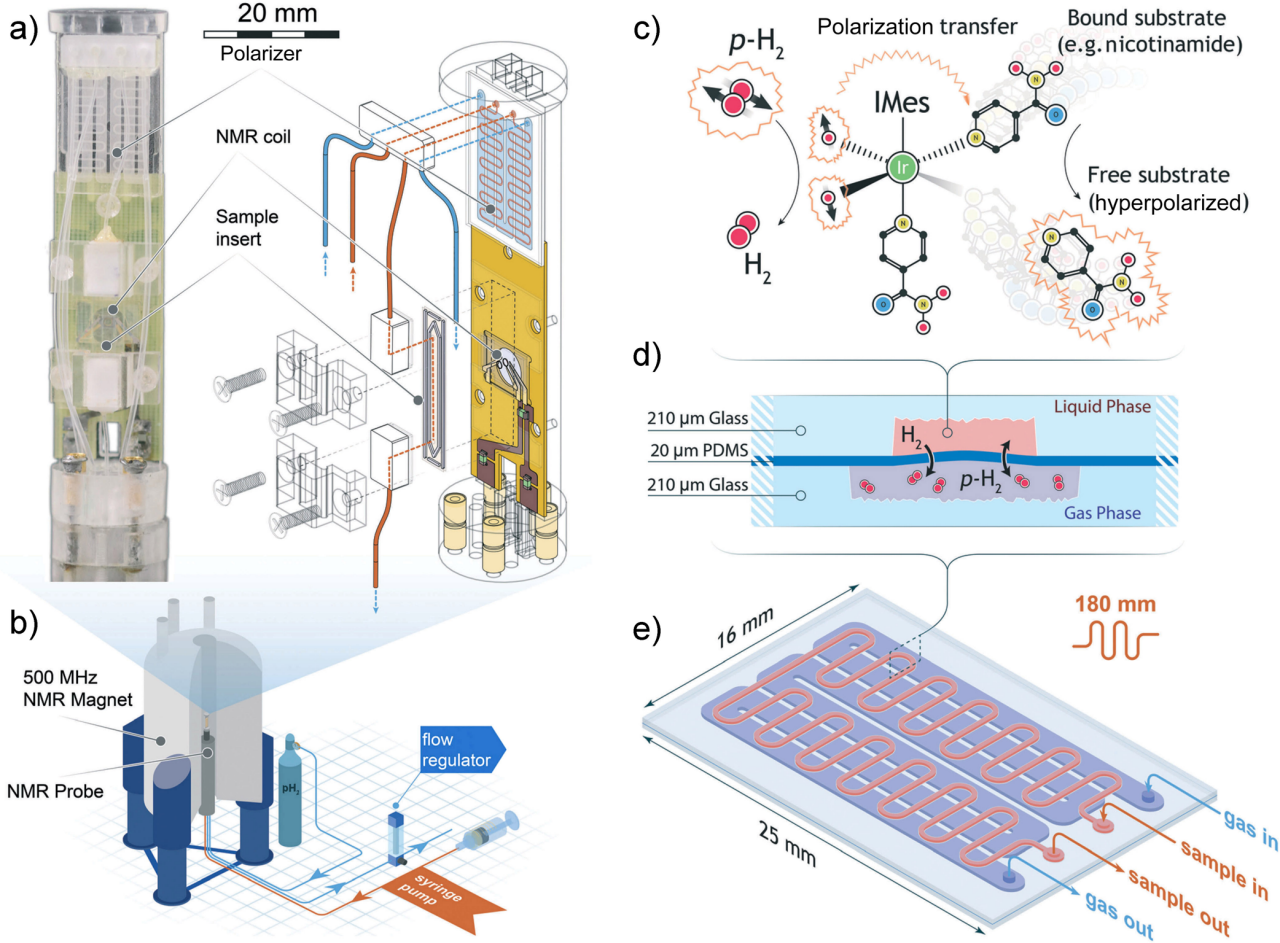
(a) Photograph of the micro-SABRE platform mounted on the head of the NMR probe (left), and schematic drawing displaying the main components of the micro-NMR platform (right). Red lines indicate gas flow paths, blue lines are fluidic flow paths. The meandering channel gas–liquid contactor visible at the upper extremity of the platform is depicted in (d). (b) An overview of the main components of the experimental layout. The gas and solution are pneumatically transported through the NMR probe mounted in an 11.74 T NMR magnet, where the custom probehead insert performs the SABRE experiment. Parahydrogen gas flow is regulated outside of the NMR probe to ensure proper pressure equilibration throughout the system. The sample solutions are injected with a standard syringe pump; any excess fluid running out of the detection area is collected in a spill-out chamber inside the NMR probe body. The area of the Helmholtz pair is 1.13 mm^2^, the detection volume enclosed by the coil is 0.56 μL. (c) Schematic representation of the SABRE process. A p-H_2_ molecule coordinates to an iridium-centered catalyst. Each hydride then has a distinct, enhanced signal in the ^1^H NMR spectrum. In the presence of a coordinated substrate, the chemical shift of the hydride is slightly modified. Simultaneously, spin-order is transferred through the coupling network from the p-H_2_ to the target molecule. A hyperpolarized substrate and a used p-H_2_ molecule are released from the complex. (d) Schematic diagram of the gas–liquid contact channel. (e) Schematic drawing of the meandering channel for improved contact area. On the gas side, the total channel length is 180 mm while the enclosed volume is 4.8 μL. On the gas side, the fluidic path is 120 mm long and the channel volume is 20.2 μL. The total area available for gas exchange is 45 mm^2^. Reproduced from ref (839) with permission from the Royal Society of Chemistry.

Microfluidic devices are being adapted for the development of advanced SABRE instrumentation for detection of small amounts of an analyte. Detection sensitivity is frequently a challenge for microfluidics applications. The SABRE hyperpolarization technique is well suited to address this limitation, because it can provide selective (i.e., only for compounds amenable to SABRE polarization) NMR signal enhancement at room temperature without chemical modification of the analyte.^839^Figure 101a shows the overall design of a microfluidic NMR probe that enables mixing of p-H_2_ with the incoming solution and is interfaced with an NMR spectrometer (Figure 101b). Fresh p-H_2_ gas flows into the RF probe and enters the solution through a gas-permeable polydimethylsiloxane (PDMS) membrane (Figure 101c).^839^ The arrangement of in and out ports for p-H_2_ gas and nicotinamide solution is shown in Figure 101d,e. NMR spectroscopic chemosensing of microliter sample volumes, nanoliter detection limits, and micromolar concentrations corresponding to picomole molecular sensitivity were achieved.^839^

In a typical sample preparation, SABRE precatalyst and to-be-hyperpolarized substrate compound are mixed in a suitable solvent. Dissolved O_2_ is removed by purging the resulting solution with inert gas (e.g., argon) or, in some applications, by several freeze–pump–thaw cycles.^802^ The presence of molecular oxygen causes several deleterious effects. First, oxygen can coordinate to the SABRE catalyst thus hindering the exchange of p-H_2_ and substrate. Second, paramagnetic O_2_ molecule may cause accelerated undesirable spin–lattice relaxation of the hyperpolarized state.^802,814^

**Figure 102:**
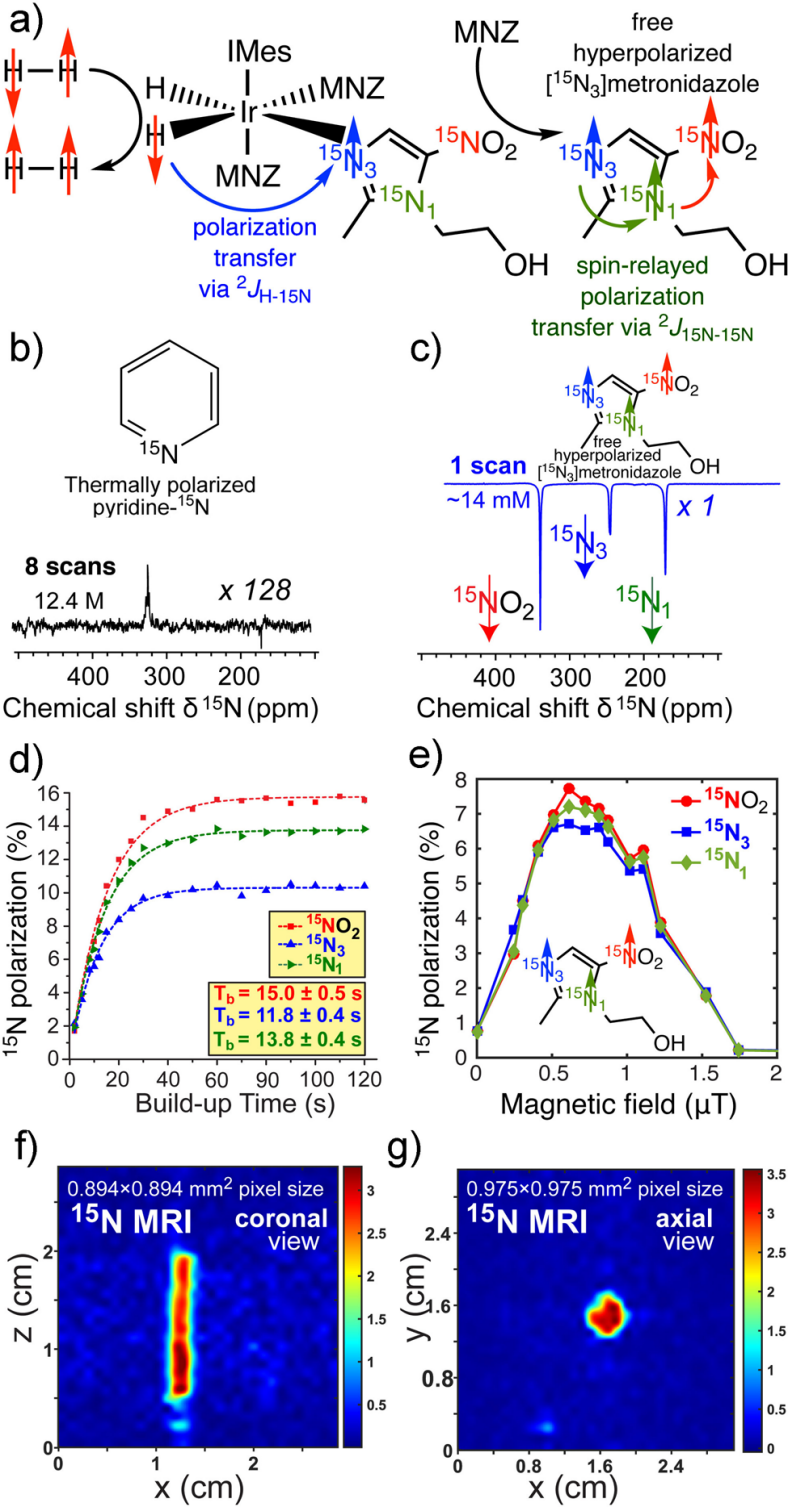
^15^N SABRE-SHEATH studies of [^15^N_3_]metronidazole. (a) Schematic representation of SABRE-SHEATH chemical exchange process and spin-relayed polarization transfer. (b) ^15^N NMR spectrum of thermally polarized signal reference compound (neat pyridine-^15^N) at 1.4 T; the sample is employed as an external polarization reference to measure polarization levels in (c). (c) ^15^N NMR spectrum of hyperpolarized [^15^N_3_]metronidazole (MNZ-^15^N_3_) hyperpolarized via SABRE-SHEATH and recorded at 1.4 T. (d) ^15^N polarization buildup of [^15^N_3_]metronidazole during SABRE-SHEATH process at ∼0.6 μT. (e) SABRE-SHEATH field optimization. (f,g) ^15^N MR images of hyperpolarized [^15^N_3_]metronidazole. (a,e) Adapted with permission from ref (840). Copyright 2021 John Wiley & Sons, Ltd. (b–d, f, g) Adapted from ref (841) with permission from the Royal Society of Chemistry.

The next important step is precatalyst activation (Figure 97). The most widely used precatalyst as of ca. 2022 is IrCl(COD)(IMes), where IMes is 1,3-bis(2,4,6-trimethylphenyl) imidazol-2-ylidene and COD is cyclooctadiene.^802^ Its activation with H_2_ leads to hydrogenation of the COD ligand, its removal, and formation of a hexacoordinate complex (Figure 97, Figure 102a).^802,835,840^ The two axial nonexchangeable positions are occupied by the IMes ligand and a substrate molecule.^802^ The four exchangeable equatorial positions are occupied by two hydrides and the substrate molecules (and sometimes by coligands or solvent molecules).^802^ It has been shown that substrate molecules (e.g., most notably pyridine) in the equatorial plane in this complex can both undergo exchange.^802^ Alternatively, a substrate molecule can occupy two binding sites of the Ir-IMes complex, e.g., most notably pyruvate, representing a bidentate versus monodentate ligand coordination to the metal center.^808^

Complete catalyst activation takes anywhere from a few minutes^835^ to a few hours depending on the experimental procedures. The activated complex can be stable for many hours and potentially days.^842^ Following the catalyst activation, SABRE hyperpolarization process is performed via introduction of p-H_2_. As p-H_2_ and substrate exchange happen on the time scale ranging from milliseconds to hundreds of milliseconds,^843^ multiple sequential exchange events lead to polarization buildup of bulk free substrate over time (Figure 100d, Figure 102d).^819^ Spin–lattice relaxation acts as a limiting factor for achieving the steady state. In the vast majority of cases, the steady-state equilibrium is thus achieved over the course of a few seconds to 1–2 min. The detailed chemical kinetics and spin dynamics of the formation of SABRE hyperpolarization have been thoroughly described elsewhere.^844,845^

In the case of metabolomics and other analytical applications, SABRE offers a tremendous utility because the hyperpolarization process can be conveniently performed and repeated in a conventional 5 mm NMR tube.^846^ The rehyperpolarization reproducibility enables 2D NMR spectroscopy.^847−849^ However, biomedical applications demand preparation of biocompatible solutions.^22^ In SABRE, alcoholic solutions of the Ir-IMes catalyst are typically employed, whereas biomedical applications require preparation of a solution of hyperpolarized substrates that is free from Ir metal and organic solvent(s).^22^

**Figure 103:**
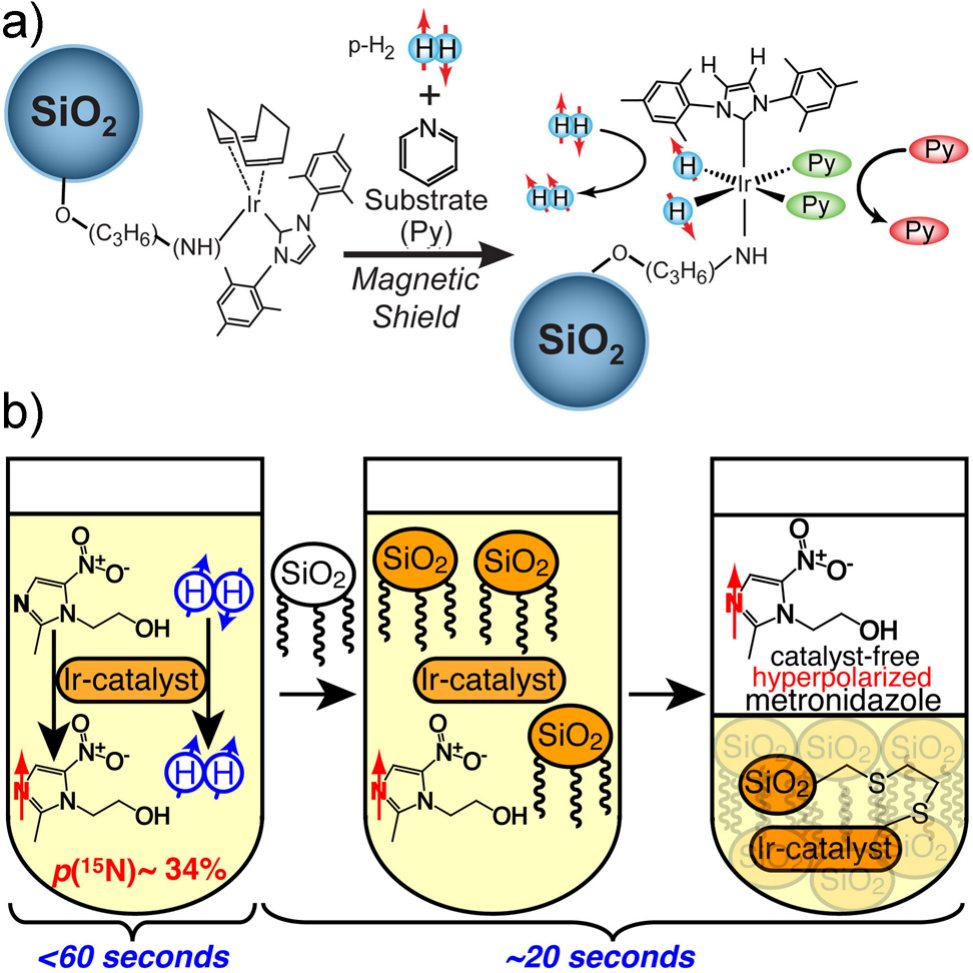
Two approaches for preparation of catalyst-free SABRE-hyperpolarized solutions. (a) Schematic representation of heterogeneous (HET) ^15^N SABRE-SHEATH process to produce ^15^N-hyperpolarized pyridine (Py). (b) Schematic representation of SABRE hyperpolarization of metronidazole drug followed by catalyst capture by functionalized silica beads resulting in transparent catalyst-free solution of hyperpolarized metronidazole. (a) Adapted with permission from ref (850). Copyright 2017 Wiley-VCH Verlag GmbH & Co. KGaA, Weinheim. (b) Reproduced with permission from ref (851). Copyright 2018 American Chemical Society.

There are a number of ways to obtain such solutions. The first group of methods rely on heterogeneous (HET) catalysis, where the SABRE catalyst is immobilized on a solid support. As a result, once hyperpolarization process is completed, the solution can be easily separated from the solid phase (Figure 103a).^852^ Although substantial progress has been made in this direction, the achieved ^1^H^852^ and ^15^N^850^ polarization levels are approximately an order of magnitude lower than those reported for homogeneous catalysts. Future catalyst optimization may allow obtaining substantially higher polarization levels using HET-SABRE.^850^ The alternative solution for catalyst removal is the use of materials that can irreversibly bind the SABRE catalyst (Figure 103b).^853^ Indeed, a number of functionalized solid materials were demonstrated to bind the SABRE catalyst after the hyperpolarization process.^851,854^ For example, commercial functionalized silica beads (mercaptopropyl silica, QuadraSil MP, Sigma-Aldrich 679526, and 2-mercaptoethyl ethyl sulfide silica, Sigma-Aldrich 745111) bind the catalyst, and the resulting hyperpolarized solution can then be separated from the solid material together with the captured Ir-containing species.^851,854^ Another alternative is the phase extraction approach, where the catalyst is retained in organic layer, while hyperpolarized substrate migrates to an aqueous layer after the SABRE hyperpolarization procedure.^855^ This procedure is similar to the one used in combination with the PHIP-SAH approach (section 3.11.2).

Similarly, an organic solvent use can be obviated by performing SABRE hyperpolarization in aqueous media.^835,856,857^ Indeed, a number of reports have shown its feasibility for ^1^H and ^15^N spins in biologically relevant molecules. To date, these pioneering efforts yielded polarization values approximately an order of magnitude lower than the corresponding values in organic solvents.^858,859^

##### 3.11.4.3 Applications

Biomedical applications are the key driver behind the development of hyperpolarization techniques.^860^ Although SABRE-hyperpolarized biocompatible compounds have not been demonstrated as contrast agents in vivo yet (ca. 2022), a number of such compounds are now in development for potential bioimaging applications of the SABRE hyperpolarization techniques. Here, we briefly summarize most notable substrates under development.^22^ Both [1,2-^13^C]pyruvate^808^ and [1-^13^C]acetate^809^ have been successfully hyperpolarized with the vision that SABRE can potentially provide a substantially cheaper and faster gateway to these hyperpolarized contrast agents that have already been validated in vivo using the *d*DNP technique (section 3.4). Of note, [1-^13^C]pyruvate hyperpolarized by *d*DNP is under evaluation in over 30 clinical trials and numerous clinical studies (ca. 2022).^861^ This molecule undergoes metabolic conversion to hyperpolarized [1-^13^C]lactate, ^13^C-bicarbonate, and [1-^13^C]alanine, which can be mapped in vivo using the magnetic resonance spectroscopic imaging (MRSI) technique.^862^ As a result, the abnormal metabolism of many diseases can be detected in real time, paving the way to future diagnostic application of MRSI using hyperpolarized [1-^13^C]pyruvate for detection, staging^863^ and monitoring response to treatment of cancer^864^ and other diseases.^865^

A number of drugs have been successfully hyperpolarized with SABRE as well, including pyrazinamide, isoniazid,^866^ and dalfampridine.^843,867^ The key rationale for SABRE hyperpolarization of such targets is to employ them as xenobiotics for potential imaging applications.

**Figure 104:**
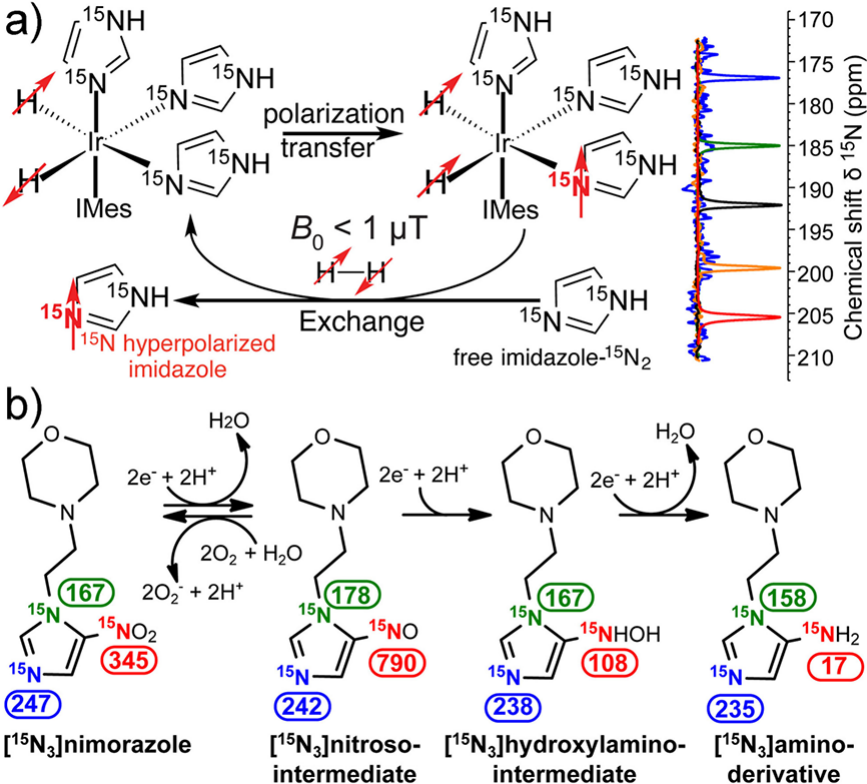
(a) Schematic representation of ^15^N SABRE-SHEATH hyperpolarization of [^15^N_2_]imidazole with p*K*_a_ of ∼7.0. Note the change in chemical shift of hyperpolarized ^15^N resonance in a broad dynamic range as a function of pH shown on the right: pH 12.0 (red), 8.0 (orange), 7.0 (black), 6.2 (green), and 4.6 (blue). (b) Metabolism of nitroimidazole compounds on the example of nimorazole in hypoxic environment; color-coded values of ^15^N chemical shifts (in ppm) were computed for aqueous media using Gaussian’09 ab initio calculations. (a) Reproduced with permission from ref (870). Copyright 2016 American Chemical Society. (b) Reproduced with permission from ref (842). Copyright 2020 Wiley-VCH GmbH.

Because in vivo proton *T*_1n_ times are rather short (ca. several seconds in the best-case scenario), substantial efforts are focused on developing SABRE for hyperpolarizing ^15^N and ^13^C nuclei in compounds that, in addition to biomedical relevance, satisfy three key conditions: high polarization levels, long hyperpolarization lifetimes (typically, for heteroatoms without directly attached protons), and safe profile at a dose of at least a few hundred milligrams. To that end, both [1-^15^N]nicotinamide^868^ and nicotinamide with natural ^15^N isotope abundance have been successfully hyperpolarized;^869^ this can be potentially employed for metabolic sensing of this key metabolite. ^15^N nuclei of [^15^N_2_]imidazole have been hyperpolarized for the purpose of pH sensing using ^15^N NMR, which was successfully demonstrated in vitro (Figure 104a).^870^ Furthermore, a nitroimidazole class of compounds have been hyperpolarized by SABRE-SHEATH with the vision that they can be employed as hypoxia-sensing probes.^805,842^ The radioactive ^18^F variants of structurally similar compounds (most notably FMISO, or fluoromisonidazole^871^) have already been successfully employed in positron emission tomography (PET) for hypoxia sensing in cancer. However, they require long clearance time (over 2 h), which is a substantial drawback of this imaging technique in addition to the use of radioactivity. Hyperpolarized ^15^N-labeled nitroimidazoles can potentially address this shortcoming, because, during metabolism, the ^15^N NMR signals of the labeled sites experience substantial changes in chemical shifts^872^ (up to a few hundred ppm, Figure 104b).^842^ Several representative examples from this class of compounds have been hyperpolarized via SABRE-SHEATH with ^15^N polarization exceeding 50%: metronidazole (including [^15^N_3_]metronidazole, Figure 102), ornidazole,^873^ and nimorazole.^842^ Given that these compounds are already approved for medical use for treatment of anaerobic infections, rapid clinical translation may be envisioned. Moreover, nimorazole is in phase 3 clinical trials as a radiosensitizing agent, and theragnostic applications of this hyperpolarized biomolecule have been proposed.^842^ The *T*_1_(^15^N) values of up to 10 min have been demonstrated in this class of compounds, making them suitable for biomedical translation.^819^ The development of many other biocompatible compounds is expected to expand the repertoire of hyperpolarized drugs.

MR imaging of SABRE-hyperpolarized compounds was demonstrated successfully, including ^1^H,^804^^13^C,^808,869^ and ^15^N MRI^841,842,874^ (see, for example, Figure 102f,g). Although ^15^N NMR detection sensitivity is generally regarded to be low (∼1% of that of protons even for ^15^N-enriched samples in a hyperpolarized state), it is envisioned that indirect detection can mitigate the sensitivity challenge. For instance, production and prolonged storage of hyperpolarization on ^15^N nuclei with its subsequent transfer to ^1^H nuclei for NMR signal detection were successfully demonstrated with SABRE for ^15^N-labeled diazirines^875^ in addition to an earlier demonstration for ^15^N-labeled choline hyperpolarized with *d*DNP.^47^

Several other applications of SABRE (besides biomedical ones) have also emerged, and they are succinctly discussed below.

The field of SABRE-hyperpolarized biochemicals is evolving rapidly, including amino acids,^876^ peptides,^877^ and water.^878^ New applications are emerging quickly, e.g., SABRE-enhanced characterization of protein–ligand interactions.^879^ Moreover, the SABRE-Relay^812^ approach described above opens up an opportunity to hyperpolarize molecular targets that do not directly interact with the polarization transfer complex.

**Figure 105:**
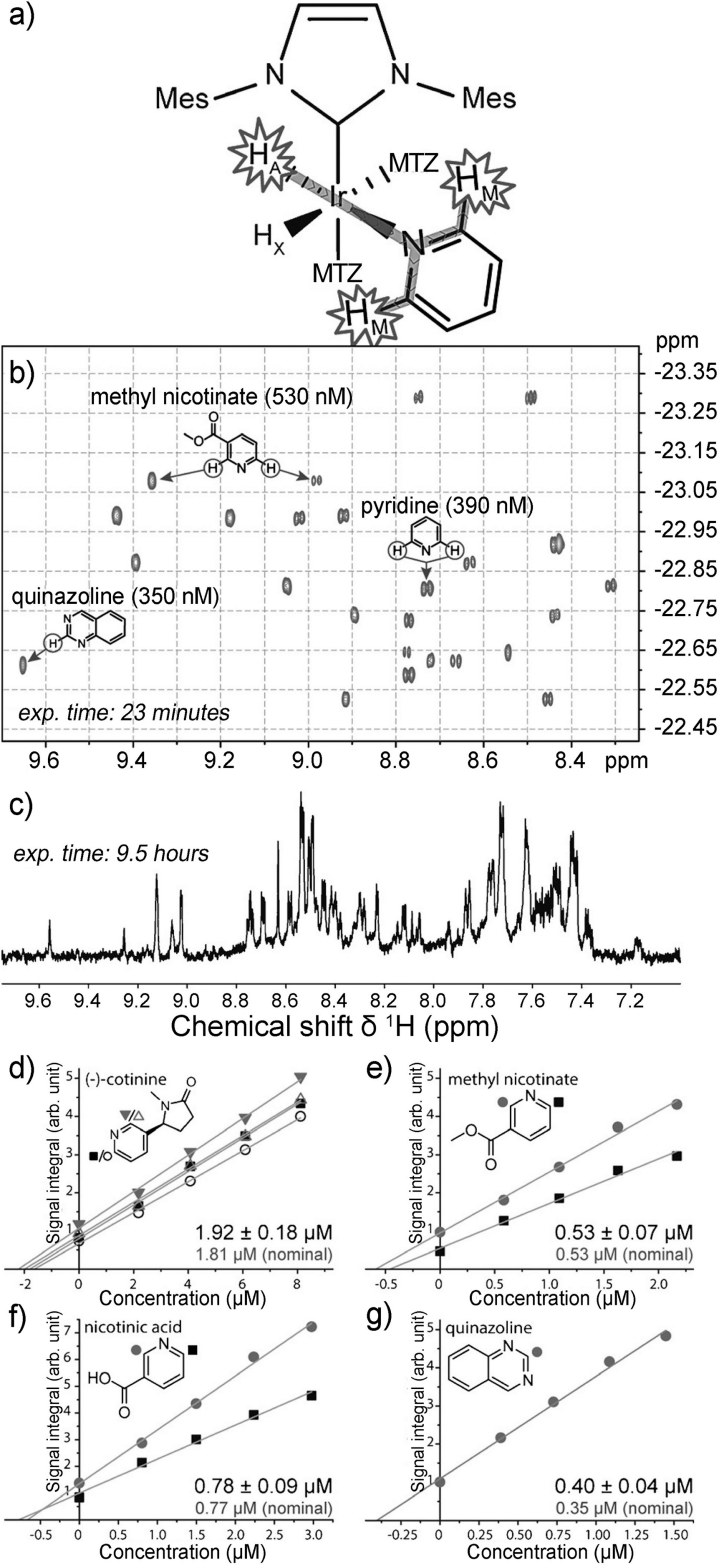
(a) Schematic representation of the asymmetric [Ir(IMes)(H)_2_(MTZ)_2_(sub)]Cl complex for a pyridine-like substrate (sub) in the presence of a large excess of 1-methyl-1,2,3-triazole (MTZ) as cosubstrate. Only hydride H_A_, in the trans position with respect to the substrate, displays an appreciable scalar coupling interaction with substrate protons H_M_. (b) A 2D ^1^H-^1^H correlation spectrum between enhanced hydrides and aromatic protons of a mixture of 13 SABRE substrates with concentrations between 250 nM and 2 mM. The spectrum was recorded in 23 min at 258 °C in the presence of 2 mM metal complex, 30 mM MTZ, and 5 bar of 51%-enriched p-H_2_. (c) 1D spectrum of the same substrates mixture, in the absence of metal complex, MTZ, and p-H_2_. This spectrum was acquired with 32,768 scans in 9.5 h using a 30° flip-angle pulse and a recovery delay of 1 s. All spectra were acquired at 500 MHz ^1^H resonance frequency. (d–g) Standard addition curves for (−)-cotinine (d), methyl nicotinate (e), nicotinic acid (f), and quinazoline (g). The symbols used in the graphs refer to different protons, as indicated next to the molecular structures. Note that each (−)-cotinine proton results in two resonances when bound to iridium because of formation of diastereomeric complexes. Reproduced with permission from ref (847). Copyright 2015 WILEY-VCH Verlag GmbH & Co. KGaA, Weinheim.

SABRE has been shown to improve the detection limit of high-field NMR spectroscopy down to the nanomolar range.^846^ In addition to direct detection of SABRE-hyperpolarized substrates, the hydrides of the iridium complex can themselves serve as the indirect sensing moieties of the exchanging substrate (Figure 105).^847^ This indirect sensing becomes possible because the chemical shift of the hydride resonance changes when the exchanging substrate coordinates to the complex.^847^ It has been demonstrated that multiple resonances corresponding to multiple exchanging substrate species can be detected when the catalyst is activated in the presence of 1-methyl-1,2,3-triazole (MTZ), Figure 105b,c.^847^ MTZ binds strongly to the iridium complex, rendering a competitive ligand binding in the presence of other substrate molecules in the solution. This approach was successfully extended to 2D NMR spectroscopy for simultaneous detection of multiple substrates in the solution in the presence of MTZ,^847^ and quantitative trace analysis of complex mixtures^880^ with applications related to analysis of biofluids and extracts (Figure 105d–g).^881−883^ Another study reports on an approach for the detection and quantification of α-amino acids down to submicromolar concentrations in complex mixtures based on the propensity of these compounds for strong axial–equatorial bidentate binding to the Ir center.^884^ One attractive feature of this approach for practical applications is that it can be used for complex biofluids such as urine with essentially no sample pretreatment apart from dilution of an aqueous mixture with methanol.

SABRE is also currently utilized for radiofrequency amplification by stimulated emission of radiation (RASER), a variant of maser operating in the radiofrequency range. A sufficiently high magnetization (usually inverted; see section 3.11.2 for more details) required for RASER conditions can be easily achieved^885^ with canonical SABRE of protons performed in the millitesla magnetic field range.^796^ Moreover, the sample can be continuously hyperpolarized with SABRE for extended periods of time, allowing one to sustain RASER activity for minutes (and potentially much longer). The pioneering demonstration of RASER was achieved in the millitesla range.^885,886^ More recent work employed a compact membrane reactor capable of continuous supply of parahydrogen to the SABRE solution that is circulated continuously between polarization transfer field (e.g., 6 mT for proton polarization) to the detection field (e.g., 1.1 or 9.4 T).^838^ This recent advance allows one to create SABRE-induced RASER at an arbitrary frequency, with up to 400 MHz RASER demonstrated so far.^838^

SABRE is also well-suited for signal enhancement of the reactants and products in chemical reaction monitoring. For example, benchtop NMR spectroscopy was employed in conjunction with SABRE to monitor the conversion of [IrCl(COD)(IMes)] in the presence of an excess of p-H_2_ and a substrate (4-aminopyridine or 4-methylpyridine) into [Ir(H)_2_(IMes)(substrate)_3_]Cl.^887^ Moreover, the authors employed this approach for monitoring the substrate deuteration.^887^ In another study, SABRE was employed to monitor chemical synthesis of methyl 2-(nicotinamide) acetate,^888^ providing signal enhancements over 130-fold with 50%-enriched p-H_2_. Most recently, SABRE was utilized^889^ to produce high levels of ^15^N hyperpolarization of a range of important nitrogen-containing synthons, such as NO_2_^–^, PhCH_2_NH_2_, ND_3_, NaN_3_, and NO_3_^–^, all exhibiting sufficiently long T_1n_ times to permit detection of hyperpolarized products and intermediates upon their chemical transformations to a range of potentially useful nitrogen-rich products.

SABRE can be also used to generate long-lived states of nuclear spins. The nascent singlet spin state of parahydrogen-derived hydrides is naturally well-suited to induce singlet state on the substrate molecules that undergo exchange on the metal center. Some representative examples are the creation of the singlet state of pyridine protons,^890^^15^N-^15^N singlet state in diazirine^821^ and ^13^C-^13^C singlet state in 1-phenyl-2-(4-pyridyl) acetylene.^822^ The substantially prolonged lifetime of singlet or pseudo singlet states of the substrate is beneficial for hyperpolarization applications. For example, the time constant of exponential decay for the ^15^N-^15^N singlet state can reach 20 min in favorable cases. The utility of the long-lived singlet spin states has been expanded to other substrates^891,892^ including the most notable case of hyperpolarized [1,2-^13^C]pyruvate.^808^

##### 3.11.4.4 Frontiers and Challenges

The SABRE technique, introduced in 2009,^796^ is still undergoing the phase of rapid development as described above. The key SABRE requirement is the simultaneous chemical exchange of p-H_2_ and to-be-hyperpolarized substrate on a metal complex. The metal complex and its structural, chemical, and electronic properties are key to maximizing SABRE efficiency, and were explored in detail.^893−895^ However, so far [IrCl(COD)(IMes)] remains the most widely used precatalyst in both fundamental studies of the SABRE effect and its applications. Many substrates naturally bind to the iridium-based hexacoordinate complex: most notably N-containing heterocycles,^799^ diazirines,^821^ Schiff bases,^896^ and others.^799^ In cases when the substrate direct binding with a metal complex is either too weak or too strong (i.e., the exchange is too fast for spin dynamics to establish polarization transfer, or too slow for a hyperpolarized substrate to be released), various strategies have been developed including the addition of a coligand to modulate the substrate binding.^897^ Addition of coligands has led to development of the SABRE-Relay variant mentioned earlier.^812^ To conclude, the strategic ongoing experimental efforts are focused on expanding the scope of substrates, development of more efficient and robust hyperpolarization schemes, preparation of biocompatible hyperpolarized solutions, validation in cellular and animal models of various diseases, developing new applications and expanding the scope of already established ones. These advances become fundamentally possible because the SABRE technique is relatively straightforward with respect to chemistry and instrumentation.^799^

The theoretical basis for SABRE^797^ was developed shortly after the seminal feasibility publication.^796^ Theoretical modeling and description of SABRE effects have been expanding and evolving hand in hand with the experimental efforts. In particular, the role of LACs^800^ and other polarization transfer mechanisms^898^ in SABRE was analyzed, and a simple analytical model of SABRE developed,^844^ which was later expanded to include spin dynamics.^845^ Because SABRE can occur in multiple regimes with respect to the application of static fields and RF pulses, the theoretical modeling of each SABRE “flavor” requires special consideration, for example, high-field SABRE^899^ or SABRE-Relay.^900^

### 3.12 The Haupt Effect and Quantum-Rotor-Induced Polarization

In section 2.2.3 the CH_3_ methyl rotor was highlighted as an example of a molecular system with the necessary symmetry properties to support long-lived spin states and act as a source of nuclear spin hyperpolarization. We now examine more closely how nuclear spin polarization can be generated by equilibration of the system at low temperature.

The molecular symmetry group of the methyl moiety is *C*_3_, which has 3 irreducible representations, *A*, *E*_a_, and *E*_b_; note that this is not the spatial point symmetry group, which would be *C*_3v_, but *C*_3_ is used because the reflection planes of *C*_3v_ are not experienced by the methyl group since this would require bond-breaking. The nuclear spin states are grouped such that their transformations belong to one of the three irreducible representations, and these are shown in Table 4.

**Table 4:** Nuclear Spin Eigenfunctions |*X*,*m*⟩ of the CH_3_ Moiety Grouped by Their Symmetry, where *X* Is the Symmetry of the State and *m* Is the Angular Momentum Quantum Number; *c* = e^2π*i*/3^^a^

|*A*, 3/2⟩ = |ααα⟩
|*A*, 1/2⟩ = 1/3(|ααβ⟩ + |αβα⟩ + |βαα⟩)
|*A*, 1/2⟩ = 1/3(|ββα⟩ + |βαβ⟩ + |αββ⟩)
|*A*, −3/2⟩ = |βββ⟩
|*E*_a_, 1/2⟩ = 1/3(|ααβ⟩ + *c*|αβα⟩ + c*|βαα⟩)
|*E*_a_, −1/2 = 1/3(|ββα⟩ + *c*|βαβ⟩ + c*|αββ⟩)
|*E*_b_, 1/2⟩ = 1/3(|ααβ⟩ + c*|αβα⟩ + *c*|βαα⟩)
|*E*_b_, −1/2⟩ = 1/3(|ββα⟩ + c*|βαβ⟩ + *c*|αββ⟩)

a Cyclic permutation of the spins leaves the *A* states unchanged, and the *E* states acquire a phase.

**Figure 106:**
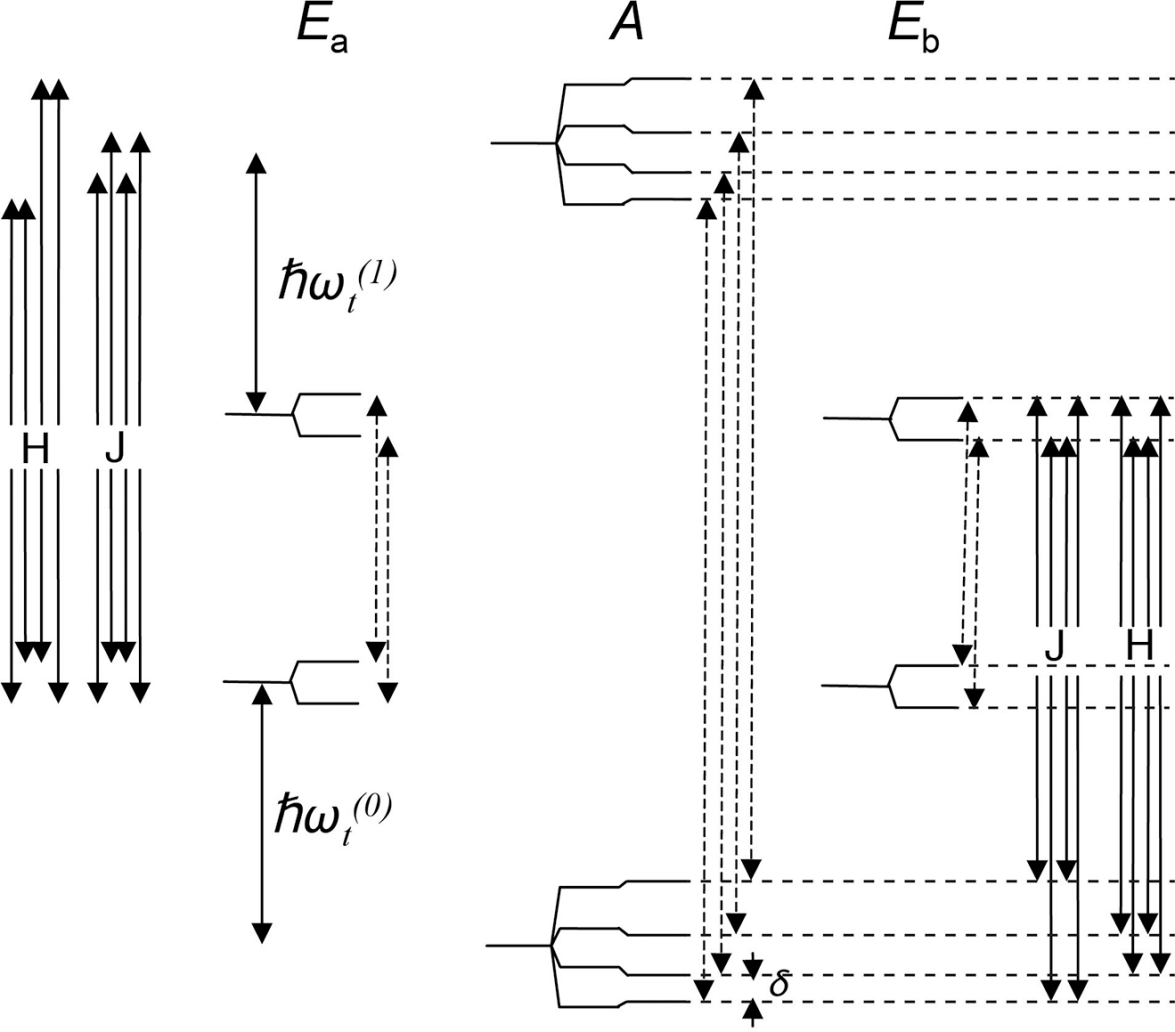
Energy level structure of the methyl rotor. The tunnel splitting is given by ℏω_t_^(i)^ where i indicates the librational state. The energy levels are labeled by their spatial symmetry, and the Zeeman interaction splits the rotational energy levels. Proton–proton dipolar coupling shifts the *A* states (δ). The dashed lines represent rapid transitions that do not require a change of symmetry species, and the solid lines represent slow transitions between *A* and *E* rotational states. H and J indicate *A*/*E* transitions that involve states in the *A* manifold shifted down or up in energy by the dipolar interaction, respectively. Reproduced with permission from ref (115). Copyright 1999 Elsevier Science B.V.

The spatial eigenstates of the methyl rotor are shown in Figure 106. The rotational states are labeled (i), and each rotational state is split further into states which possess *A*, *E*_a_, or *E*_b_ symmetry. The *E* states are degenerate, and their energy separation to the *A* state is called the tunnel splitting, or tunnel frequency, and depends crucially on the hindering barrier to methyl rotation. Lowering the hindering barrier leads to an increased tunnel splitting.

For the case of two coupled indistinguishable spin-1/2 particles (e.g., the nuclei in an H_2_ molecule), the Pauli principle dictates that the combined spatial (*ψ*_*R*_) and spin (*ψ*_*S*_) wave function must be antisymmetric with respect to particle exchange. In the case of the methyl rotor with three coupled and indistinguishable spin-1/2 particles, the Pauli principle dictates that the combined wave function must be of *A* symmetry, which means that the allowed combinations are ψ_*R*_^*A*^ψ_*S*_^*A*^, ψ_*R*_^*E*_a_^ψ_*S*_^*E*_b_^, and ψ_*R*_^*E*_b_^ψ_*S*_^*E*_a_^. Hence, by cooling such a system and selectively populating the rotational eigenstates of *A* symmetry, the nuclear spin eigenstates of *A* symmetry are also populated.

In practice, this is only possible for molecules which exhibit relatively unhindered methyl rotation in the solid state (i.e., while frozen), and hence have a large tunnel splitting, on the order of (or larger than) 0.1 meV, corresponding to the temperatures achievable in the cooling process. In typical quantum-rotor-induced polarization (QRIP) experiments, temperatures down to ∼1 K are used, since this can be readily achieved using liquid helium. This cooling process engenders an *A*/*E* population imbalance of the nuclear spin states, although note from the spin states given in Table 4 that this does not correspond to net Zeeman polarization (or magnetization), similar to the case of enriched parahydrogen (section 2.1). In contrast to parahydrogen, however, the *A*/*E* imbalance of a methyl rotor can be converted into dipolar or Zeeman observable nuclear spin polarization by the application of a sudden temperature jump. Transitions between nuclear spin states belonging to different irreducible representations of the *C*_3_ symmetry group are symmetry forbidden, which means the nuclear spins do not become polarized immediately following the temperature jump. A symmetry-breaking interaction/mechanism is required to allow transitions. Two different varieties of QRIP experiment have been demonstrated, known as the Haupt effect and dissolution QRIP (*d*QRIP).

**Figure 107:**
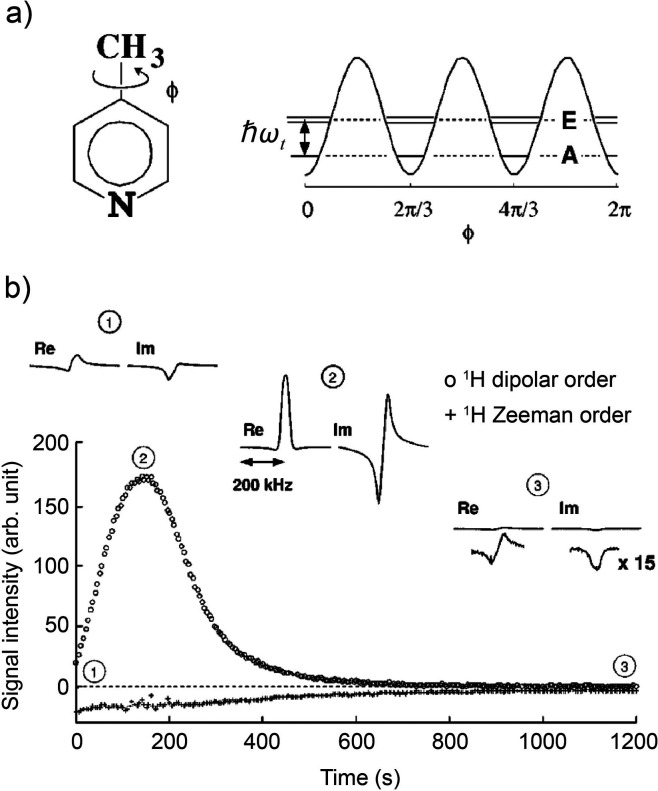
Haupt effect in a solid sample of γ-picoline. (a) Molecular structure of γ-picoline with a simplified diagram showing the potential energy of the CH_3_ group, with the lowest librational levels shown, and the *A*/*E* tunnel splitting labeled ℏω_t_. (b) The dipolar and Zeeman signals arising from the sample after a temperature jump from 4 to 55 K are shown in the plot. Spectra were acquired by applying 0.05° flip-angle pulses, and the plotted points were obtained by integrating the real and imaginary components of the spectra, respectively. This is possible because the signal originating from dipolar order after applying a single RF pulse is 90° out of phase with respect to the signal originating from Zeeman order. The *A*/*E* transitions initially lead to a build-up of dipolar order, which subsequently decays due to spin relaxation. The Zeeman order is not affected by the temperature jump, beyond reequilibrating at a lower polarization level at the higher temperature. Representative spectra at three time points are shown in the insets. Reproduced from ref (903) with the permission of AIP Publishing.

The Haupt effect was first shown in 1972 by Haupt^901,902^ using polycrystalline solid γ-picoline, cooled to low temperature. This molecule has a relatively large tunnel splitting in the solid state of approximately 0.5 meV (6 K), and hence can be polarized efficiently by QRIP. After a build-up of the *A*/*E* imbalance at low temperature, the sample is subjected to a sudden temperature jump which alters the equilibrium population distribution. Transitions between states belonging to the same symmetry species are relatively rapid, but *A*–*E* transitions are slow in comparison, since they require a change in both the spin and spatial symmetry. *A*–*E* transitions are weakly allowed by spin–spin dipolar couplings, since this interaction involves both spin and spatial components. The dipolar couplings shift the energies of the *A* states either up or down, and the resulting *A*–*E* transitions are either up-shifted or down-shifted in energy. Importantly, the difference in transition probabilities weighted by the transition energies for the up- and down-shifted energy levels is nonzero. This leads to a build-up of dipolar order in the solid, but this process alone does not lead directly to nuclear Zeeman polarization. However, the dipolar order can be observed directly in the solid state as was done in the original experiments,^902^ or transformed into Zeeman polarization with resonant radiofrequency (RF) pulses. The temperature jump can be either in a positive or negative direction, which leads to positive or negative dipolar polarization. The Haupt effect is illustrated in Figure 107. A more rigorous description of the Haupt effect can be found elsewhere.^115^

**Figure 108:**
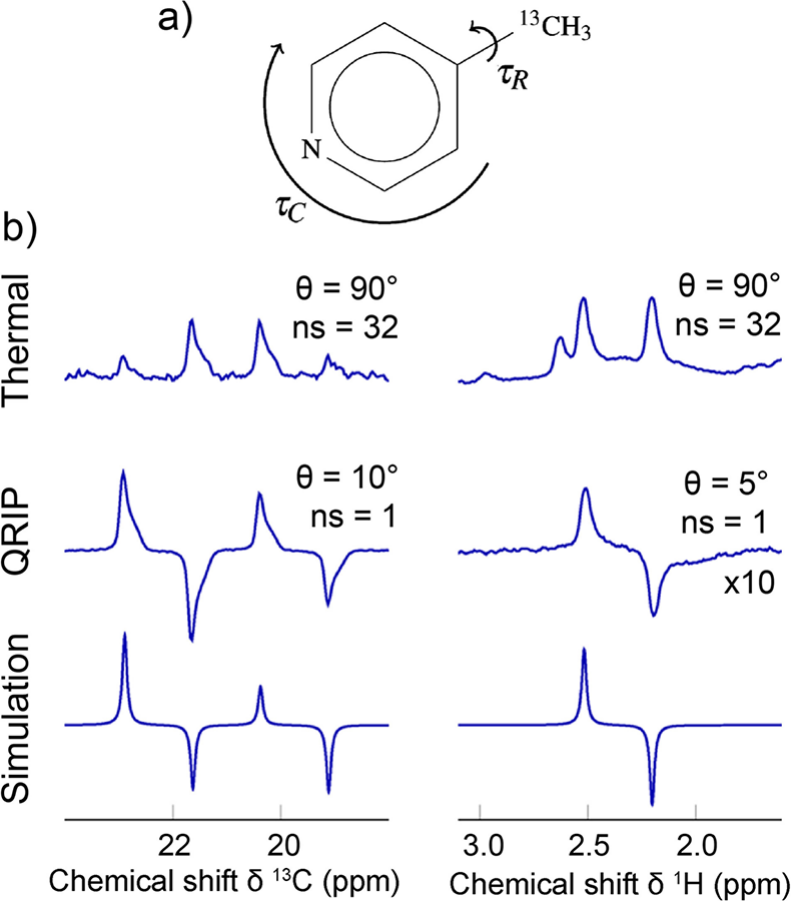
(a) A molecule of γ-picoline shown with the methyl rotational correlation time τ_R_ and the overall molecular tumbling correlation time τ_C_ labeled. (b) ^1^H and ^13^C spectra of the methyl moiety showing the *d*QRIP effect in comparison with thermal equilibrium signals acquired at 9.4 T. The spectral lines enhanced by *d*QRIP show a characteristic mixed absorption/emission line shape, which is simulated beneath. The RF pulse flip angle (θ) and number of transients (ns) are shown by each experimental spectrum. Adapted with permission from ref (905). Copyright 2013 American Chemical Society.

The first *d*QRIP experiment was shown in 2012,^904^ also on γ-picoline. In a *d*QRIP experiment, the nuclear spin polarization generated in the solid state is observed in the solution state after dissolution of the sample in a warm solvent. In this case, since the dipolar couplings are time-averaged to zero by the rapid isotropic molecular tumbling in solution, the *A*/*E* imbalance is substantially preserved in the solution state. However, although the dipolar couplings no longer produce any observable splittings in the NMR spectra, they appear in the fluctuating interactions and can cause spin relaxation. The protons in the methyl rotor are magnetically equivalent, and fluctuating dipolar couplings between the protons cannot induce *A*/*E* transitions. However, the presence of a ^13^C spin in the methyl group allows ^1^H-^13^C cross-relaxation processes to convert the long-lived *A*/*E* imbalance into observable hyperpolarized nuclear spin order on both ^1^H and ^13^C nuclei.^905,906^ The cross-relaxation rate, and hence the time constant describing the build-up of observable nuclear polarization, is determined by a combination of the methyl group rotational correlation time τ_R_ and the overall molecular tumbling correlation time τ_c_. Neglecting extraneous relaxation effects, if τ_R_ were zero the *A*/*E* imbalance would be infinitely long-lived, but not observable. There is hence a trade-off between the long-lived nature of the *A*/*E* imbalance and its conversion into observable magnetization. This experiment was originally called a QRIP experiment, but is now more appropriately referred to as *d*QRIP to better distinguish it from a Haupt effect experiment, since both rely on the broad topic of QRIP. In Figure 108 an experimental manifestation of the *d*QRIP effect is shown. A more detailed description of *d*QRIP is given elsewhere.^907^

An appealing feature of QRIP experiments is the simplicity of the required instrumentation. To generate polarization, the material is cooled to cryogenic temperatures with liquid helium. Increased polarization is available if the temperature is further lowered by vacuum pumping of the helium.^905^ A temperature jump is then used, which can either be controlled with a heater next to a sample in the solid state as in Haupt-effect experiments, or by dissolution of the sample in a warm solvent as in *d*QRIP experiments. Note that the temperature jump does not need to be to higher temperature; the Haupt effect has also been shown for jumps to a lower temperature.^902^ For *d*QRIP experiments, the cryogenic and dissolution equipment is similar to that required for *d*DNP (section 3.4) but there are two important differences: (1) no electromagnetic (MW or RF) irradiation of the sample is required since nuclear/electronic transitions do not need to be driven; and (2) it was demonstrated that QRIP is independent of magnetic field,^908^ which means no high-field magnet is required for the polarization step which can be conveniently performed in Earth’s field.

The polarization levels in QRIP experiments are typically low, since the tunnel splittings are generally not much larger than the sample temperatures attained in experiments (∼1 K). However, a ^1^H polarization level of 3% has been reported for Haupt-effect experiments on γ-picoline.^908^ For *d*QRIP, the highest reported signal enhancement was also on a sample of γ-picoline: a ^13^C enhancement of 530 was seen at 9.4 T, corresponding to ∼0.4% polarization.^909^ This was achieved using an automated dissolution setup. A dedicated dissolution setup is not required; it was shown that the polarization step can be carried out on a sample in an NMR tube submerged in liquid helium, the sample can then be extracted and dissolved by injection of a warm solvent, and finally placed in a high-field NMR magnet for signal acquisition.^910^ This manual procedure leads to polarization levels lower by close to an order of magnitude due to the additional time delay between polarization and signal observation, but requires minimal apparatus. In this work the authors showed enhancements of between 2 and 30 at 9.4 T for a number of other molecules such as 2,4-dibromo-1,3,5-trimethylbenzene, lithium acetate, and toluene. The achievable polarization level of a given molecule is approximately predicted by the tunnel splitting in the solid state, and QRIP has not been observed- on methyl rotors with a tunnel splitting of <1 μeV (∼10 mK). Although ^1^H-^13^C cross-relaxation can lead to hyperpolarized NMR signals after the sample cooling and dissolution steps, it has been rigorously shown that these signals can only be attributed to a QRIP mechanism if the methyl rotor has sufficiently free rotation.^911^

To date, QRIP has been studied at a fundamental level to understand the mechanisms and to investigate possible extensions of the technique, but it has not been applied as a hyperpolarization method to overcome challenges in magnetic resonance caused by limited sensitivity.

It was shown that ^15^N-acetonitrile, which itself does not exhibit QRIP effects due to the small methyl tunnel splitting, can be weakly hyperpolarized by inclusion in a frozen γ-picoline solid matrix.^903^ The γ-picoline proton dipolar polarization generated via the Haupt effect was transferred into ^15^N Zeeman polarization in the acetonitrile by adiabatically ramped RF fields.

In a different strategy, molecules of acetonitrile were trapped in a solid Kr matrix (0.5 mol %) to reduce the barrier for rotation of the methyl group.^912^ The authors were able to demonstrate the Haupt effect in the methyl rotor, and estimated the tunnel splitting to be approximately 50 K. This large enhancement in the tunnel splitting by incorporation of a molecule in a noble gas matrix is a promising technique that might allow QRIP to be used to polarize a much wider range of molecules, and improve the attainable polarization levels to near-unity.

As is the case with PHIP (section 3.11.2), the spin polarization attained in a QRIP experiment is independent of the external magnetic field,^908^ which means that it is particularly suited to low- or zero-field NMR detection. A nuclear quadrupole resonance (NQR) experiment was shown on a sample of γ-picoline using the following procedure: (1) generate dipolar order on the protons by a temperature jump from 7.5 to 55 K at zero field; (2) adiabatically ramp the field to 17.7 mT to convert the dipolar order to Zeeman order and allow ^1^H-^14^N spin mixing; (3) adiabatically return to zero field and acquire a hyperpolarized-^14^N NQR spectrum. The authors showed an impressive 2 × 10^3^ enhancement of the signals over the thermal equilibrium NQR signals at 7.5 K; unfortunately, the number of molecular targets this type of experiment is applicable to is limited.

Despite the simplicity of the required instrumentation, there are some significant limitations in the generality of QRIP. The method is limited to molecules containing a quantum rotor moiety; to date QRIP has only been demonstrated on molecules containing a CH_3_ group, and the specific case of H_2_^17^O@C_60_ (^17^O-enriched water trapped in a fullerene cage).^913^ In addition to this, the quantum rotor should possess a tunnel splitting in the solid state of at least on the order of one hundred millikelvin (or around 10 μeV) so that Boltzmann polarization leads to a significant *A*/*E* population imbalance. For this reason, QRIP has been found to be limited to only a handful of molecular systems.^914^ Another limitation is in the ability to utilize the engendered hyperpolarization: in Haupt-effect experiments the sample remains in the solid state in the cryostat; in a *d*QRIP experiment the sample is extracted from the polarizer as a solution, but in this case no permanent dipolar couplings exist to allow for coherent polarization transfer, and instead, Zeeman polarization is produced by ^1^H-^13^C cross-relaxation caused by the fluctuating dipolar couplings. One future direction might be to use a chemical reaction or physical interaction of a molecule polarized via *d*QRIP to render the methyl protons magnetically inequivalent, and allow for coherent transfer of the *A*/*E* imbalance into spin hyperpolarization, which could lead to much larger signal enhancements. This is a particularly exciting prospect since the *A*/*E* imbalance in a ^13^CH_3_ group corresponds to a symmetry-protected state which is long-lived in comparison to the nuclear spin *T*_1n_ times.^905,906^

### 3.13 Optical Pumping of Noble Gas Isotopes

The terminology “optical pumping” has been used in a variety of processes; however, for spin-exchange optical pumping (SEOP)^93^ and metastability-exchange optical pumping (MEOP)^915,916^ as described in this review, the goal of optical pumping (OP) is to hyperpolarize the nuclear spins of a gas-phase system, typically, noble gas atoms.

#### 3.13.1 The Technique

There are five stable (i.e., nonradioactive) noble gas isotopes with a nonzero nuclear spin. Historically, the most commonly used noble gas isotope for pulmonary MRI is ^3^He (nuclear spin *I* = 1/2) that can be hyperpolarized via SEOP, and it is the only isotope that has been successfully pumped through MEOP. Unfortunately, ^3^He is currently exclusively produced from tritium decay by the nuclear weapons industry leading to a waning stockpile as it is used for many further applications beyond MRI and NMR spectroscopy. It is increasingly being replaced by ^129^Xe (*I* = 1/2) that is a renewable resource obtained from air liquefaction; it is found at high natural abundance, and advancement in SEOP methods enables high polarization of this isotope. There are three additional NMR-active noble gas isotopes that are stable, but all of them have nuclear spin *I* > 1/2 and, therefore, possess nuclear electric quadrupole moments that cause fast *T*_1n_ relaxation. The three isotopes are ^21^Ne (*I* = 3/2), ^83^Kr (*I* = 9/2), and ^131^Xe (*I* = 3/2). All three are used to a much lesser extent than ^129^Xe but show some interesting properties due to the high nuclear spin. Due to the very low natural abundance of ^21^Ne, this isotope has found few applications. Beyond noble gas isotopes, SEOP of gas-phase molecules, such as molecular hydrogen or hydrocarbons, is also possible in principle;^917^ however, fast relaxation driven by spin-rotation interaction typically counteracts the build-up of hyperpolarization and therefore limits the polarization *p* that can be achieved. This relaxation also leads to rapid loss of the modest polarization in these molecules, and practical applications remain largely unexplored for this reason.

The high nuclear spin *I* > 1/2 for three of the five NMR-active noble gas isotopes requires a closer look at the definition of spin polarization *p*. Commonly, for spin*-*1/2 systems, the polarization *p* is introduced in a semiclassical description using the difference in the populations *n*_α_ and *n*_β_ of the α and β spin states, normalized by the total number of spins (see eq 1). Populations under thermal equilibrium are governed by the Boltzmann distribution, and at temperatures where the high-temperature approximation is applicable, for *I* = 1/2 the polarization can be expressed as
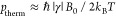
8(cf. eq 3). For an arbitrary spin *I*, the high-temperature approximation gives^918^
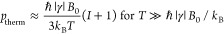
9

Note that for *I* = 1/2, eq 9 gives the same result as eq 8. The polarization of a hyperpolarized system is typically determined from the thermal equilibrium polarization of eq 9 multiplied by the experimentally obtained enhancement factor. Table 5 displays thermal equilibrium polarization values for the various noble gas isotopes at 3 T magnetic field strength. Note that eq 9 provides the polarization as a fraction of unity, i.e., the maximum polarization possible. Alternatively, polarization values are reported as a percentile with the maximum possible polarization of 100%.

**Table 5:** Complete List of Stable Noble Gas Isotopes with Nuclear Spin *I* > 0^a^

Isotope	Nuclear spin *I*	Natural abundance, %	NMR frequency at 3 T for pure gas interpolated to zero density, MHz	Thermal equilibrium polarization at 3 T and 300 K	Nuclear electric quadrupole moment (*Q*)/10^–28^ m^2^
^3^He	1/2	<2 × 10^–4^	97.30	7.78 × 10^–6^	–
^21^Ne	3/2	0.27	10.08	1.34 × 10^–6^	0.102
^83^Kr	9/2	11.49	4.915	1.44 × 10^–6^	0.259
^129^Xe	1/2	26.44	35.33	2.83 × 10^–6^	–
^131^Xe	3/2	21.18	10.47	1.40 × 10^–6^	–0.114

a The noble gas resonance frequencies are for the pure gas (oxidation state 0), interpolated to zero gas pressure as the resonance frequency is pressure-dependent, in particular for Xe and Kr (note that some manufactures of spectrometers use XeOF_4_ for frequency reference, leading to more than 5000 ppm higher resonance frequencies than that of the gas). The thermal equilibrium spin polarization *p*_therm_ for 3 T magnetic field strength and 300 K is provided based on eq 9 (i.e., based on a theoretical maximum polarization of *p* = 1).

Both SEOP^93^ and MEOP^915,916^ use circularly polarized photons (section 2.2.4) as a primary source for the hyperpolarization of electron spins of an atomic system in the gas phase (i.e., alkali metals in SEOP, and metastable ^3^He* in MEOP, see below) which then serves as an intermediary source to obtain the final goal of noble gas nuclear spin hyperpolarization (Figure 3).

The hyperpolarization of noble gases is a two-step process. The first step is optical pumping of electrons of an atomic system to produce electron spin polarization. In the second step, described further below, this polarization is transferred to the nuclear spin of a noble gas.

**Figure 109:**
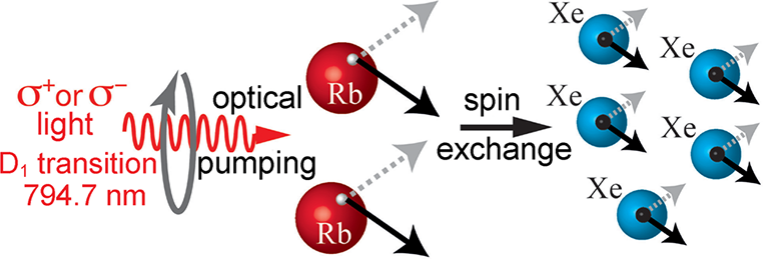
SEOP is a two-step process. Angular momentum from circularly polarized light (σ^+^ or σ^–^ photon) is transferred to the electron spin during absorption by the rubidium electronic structure in the optical pumping process that leads to electron spin polarization. Spin exchange transfers the electron spin hyperpolarization to the nuclear spin of a noble gas (here, the isotope ^129^Xe with spin *I* = 1/2).

SEOP (in contrast to MEOP, see below) requires alkali metal atoms, and optical pumping of their electron spins is used to produce hyperpolarization intermediaries (Figure 109). Alkali metals have low melting points and high vapor pressures sufficient for performing SEOP at a fairly low temperature. Depending on the noble gas isotope to be hyperpolarized, the temperature for SEOP with rubidium, the most commonly used alkali metal for this process, is typically in the range of 373–453 K. Rubidium has a melting point of 312.4 K (39.3 °C), although cesium is a promising additional contender.^919^ Rubidium density in the gas phase as a function of temperature can be calculated according to the following empirical equation:^920^
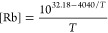
10where [Rb] is expressed in the units of atoms per m^3^ and *T* is temperature in kelvin. At a temperature of 373 K (100 °C), close to that commonly used for ^129^Xe SEOP, one obtains [Rb] = 6.0 × 10^18^ m^–3^. Like all alkali metals, Rb has a single electron in its outer electron shell, i.e., the 5s orbital in the case of Rb. If the laser is tuned in resonance with the D_1_ transition, the outer electron will be pumped from the 5s level to the 5p energy level. Using term symbols, the transition is 5 ^2^S_1/2_ → 5 ^2^P_1/2_ and this requires photons in the near-infrared with a wavelength of 794.7 nm, where high laser power is readily available. Note that other transitions can be used as well, for instance, the D_2_ transition.^919^

**Figure 110:**
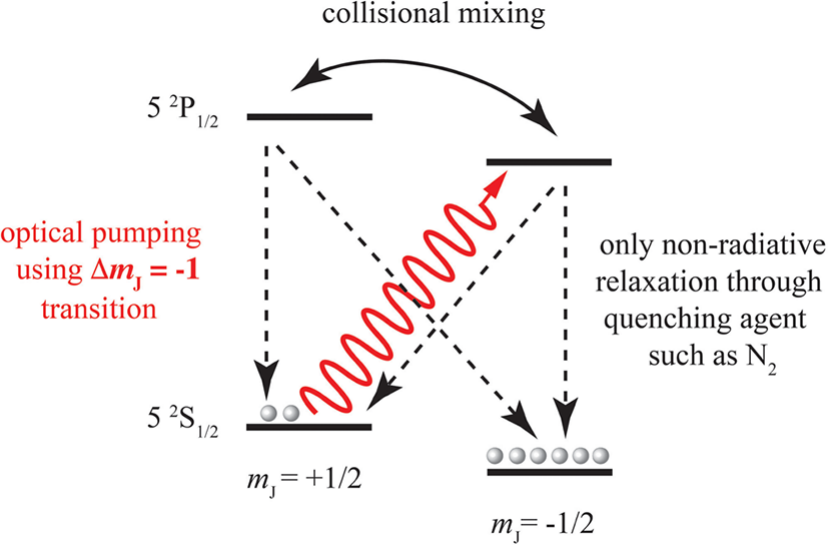
Concept of optical pumping using the D_1_ transition, with Δ*m*_J_ = −1 transitions illustrated here. Note that the conservation of angular momentum dictates the selection rules for circular photon absorption to be either Δ*m*_J_ = −1 or +1, depending on circularity of the photons (σ^+^ or σ^–^) and the direction of the magnetic field (i.e., parallel or antiparallel to the laser beam). The D_1_ transition wavelength for Rb is 794.7 nm. Although optical pumping shown here transfers the electrons into the *m*_S_ = −1/2 sublevel of the excited state (P-term), collisional mixing in the excited state and relaxation in both sublevels mixes the spin states again. Nevertheless, due to continuous optical pumping, the +1/2 sublevel gets depleted while the −1/2 sublevel gets increasingly populated.

Figure 110 depicts the energy levels if a small magnetic field is present that lifts the 2-fold degeneracy of the ^2^S_1/2_ and ^2^P_1/2_ levels into two sublevels each due to the z-quantization of the total electron angular momentum *J* = 1/2 of the electron shell that is dictated by the electron spin quantization (i.e., *m*_S_ = +1/2 and −1/2).

Upon absorption of circularly polarized (σ^±^) photons, that carry either +1 or −1 spin projection, the angular momentum is transferred to the electrons. The selection rule Δ*m*_J_ = ±1 applies if the magnetic field direction that defines the quantization axis of this process is parallel or antiparallel to the light propagation. The sign in the selection rule depends on the handedness of the σ^±^ photons and the beam direction in the magnetic field (i.e., parallel or antiparallel).

The optical pumping procedure is illustrated in Figure 110 for the Δ*m*_J_ = +1 scenario that allows only for electrons in the *m*_S_ = −1/2 sublevel of ^2^S_1/2_ to be excited into the *m*_S_ = +1/2 sublevel of ^2^P_1/2_. Note that the spin orientation of the electrons pumped into the ^2^P_1/2_ level is only transient due to (1) rapid collisional mixing and (2) relaxation into both sublevels of ^2^S_1/2_. Under continuous laser irradiation, an ongoing selective population depletion of one of the two ^2^S_1/2_ sublevels (i.e., the *m*_S_ = −1/2 sublevel in Figure 110) through the selective pumping process causes the population of the other sublevel (i.e., *m*_S_ = +1/2) to accumulate. As a consequence, a steady-state electron spin polarization occurs, described by^921^
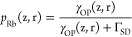
11where
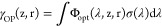
12is the optical pumping rate that depends on the power density Φ_opt_(λ,z,r) of the laser light within the width of the D_1_ transition wavelength, but also on the radial profile (r dependence) of the laser beam and the position z along the axis of propagation of laser light through the light-absorbing rubidium vapor and its pressure and gas composition dependent absorption cross section σ(λ)^922^

The maximum value of *p*_Rb_(z,r) is unity but the “spin destruction” rate Γ_SD_ counteracts the pumping process and may therefore reduce this value. Spin destruction describes all processes that lead to a reduced population accumulation at the *m*_S_ = +1/2 sublevel in Figure 110. Note that, unlike the ^2^P_1/2_ sublevels, the sublevels of ^2^S_1/2_ usually do not exhibit rapid collisional mixing. This crucial effect that makes optical pumping feasible is due to the *l* = 0 orbital angular momentum of the S-term (s orbital) and hence there is no possibility for spin–orbit coupling with the electron spin. Atomic collisions are therefore much less effective in causing electron relaxation in s orbitals compared to p orbitals. Nevertheless, strong atomic deformation through collisions with heavy noble gas atoms still causes significant Rb electron spin depolarization with an overall rate constant
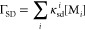
13that is the product of the number density of the corresponding gas species [M_i_] in the collision and their specific spin-destruction rate constants κ_sd_^*i*^, summed up for all species present in the SEOP mixture. The Rb spin-destruction rate constant caused by xenon is κ_sd_^Xe^ = 5.2 × 10^–21^ m^3^ s^–1^, a value that is about 3 orders of magnitude larger than that of helium and still 500 times larger than that of molecular nitrogen. Therefore, it is important to keep the xenon number density low during SEOP either through low total gas pressure or at least through low xenon partial pressures in mixtures diluted with other buffer gases such as helium, molecular nitrogen, or other gases.

The diminished Rb electron spin polarization reduces the polarization that can be passed to the noble gas nuclei, as described further below. However, it may have a further detrimental effect as it also increases the likelihood of the “rubidium runaway” effect. A high alkali metal density in the gas phase, in particular a high density of dark, nonpolarized rubidium, leads to an increased heating effect that, in turn, increases the Rb gas phase density even further. This is detrimental to the overall SEOP as the laser light will no longer be able to penetrate along the entire length of the SEOP cell.^923^ Cooling the front of the cell may mitigate this effect but a high xenon density makes this more challenging.^924^

Rubidium–rubidium atomic collisions can also lead to spin destruction as the specific rate constant is κ_sd_^Rb–Rb^ ≈ 8.1 × 10^–19^ m^3^ s^–1^; however, this effect can usually be ignored at the low number density [Rb] at typical ^129^Xe SEOP temperatures (see eq 10). This is different for the production of hyperpolarized ^83^Kr with high SEOP temperatures around 433 K (160 °C) where Rb–Rb collisions can contribute up to 20% to the electron spin destruction, in particular since the spin destruction rate from collisions with krypton atoms is about five times lower than that caused by xenon collisions.^925^

At lower pressures, a significant Rb polarization loss is also induced by spin-rotation interactions^926^ but at typical SEOP pressures this contribution can be neglected.^925^ Lastly, a strong but avoidable contribution to Rb electron spin depolarization originates from radiation trapping. Fluorescence upon relaxation of pumped Rb electrons back into the S-levels (5 ^2^P_1/2_ → 5 ^2^S_1/2_) may be detrimental to the Rb spin polarization because it can lead to radiation trapping where a single incident circularly polarized photon gives rise to multiple scattered photons that are arbitrarily polarized. Unlike monatomic noble gases, molecular nitrogen can quench the fluorescence by dissipating the energy from excited rubidium electronic states into vibrational modes.^927,928^ Relatively small number densities [N_2_] present in SEOP mixtures with about 5% N_2_ at ambient pressure can largely eliminate this effect.^925,928^ Molecular hydrogen can also serve as a radiation-quenching agent although it is less effective and higher densities are required.^929^

**Figure 111:**
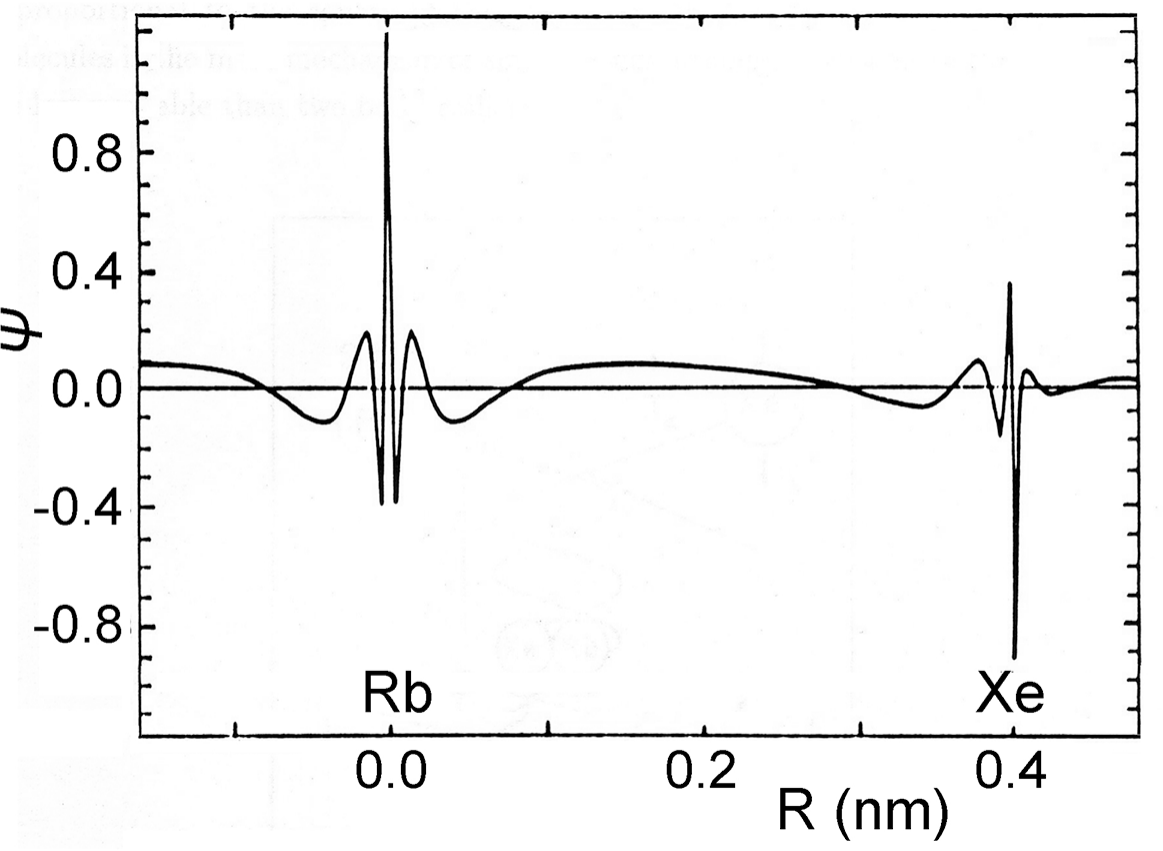
5s rubidium electron wave function ψ(*R*) during interaction with a xenon atom. Note the large nonzero value at the location of the xenon nucleus. Adapted with permission from ref (930). Copyright 1997 American Physical Society.

Another essential part of the SEOP process is spin exchange. The optical pumping process described above causes Rb electron spin hyperpolarization. In order to produce hyperpolarized noble gas, Rb electron spin polarization needs to be transferred to the nuclear spin of the noble gas, a process that is driven by Fermi contact interaction upon a close contact of a noble gas atom with a rubidium atom in the gas phase. Direct dipolar interactions between an electron spin and a nuclear spin, i.e., dipolar interactions through space, average to zero due to fast motion in the gas phase. However, the Fermi contact interaction is a scalar interaction between the electron spin *Ŝ* and nuclear spin *Î* and the corresponding Hamiltonian is
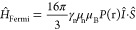
14where μ_n_ and μ_B_ are the nuclear and Bohr magneton, γ_n_ is the gyromagnetic ratio of the noble gas (NG) isotope involved in the spin exchange, and *P*(r) is proportional to the probability of finding the alkali metal outer electron at the location of the noble gas nucleus, i.e., the square of the electron wave function at the location *R*_0_^NG^ of the noble gas atomic nucleus. A plot of the wave function during close proximity interaction between Rb and Xe is shown in Figure 111 that illustrates the high value expected for |ψ(*R* = *R*_0_^Xe^)|^2^, crucial for the Fermi contact interactions to take place.^930^

The scalar interaction, written as the dot product of the electron spin and nuclear spin operators *Ŝ* and *Î*, respectively, can be rewritten using the raising (*Î*^+^ = *Î*_*x*_ + *iÎ*_*y*_) and lowering (*Î*^–^ = *Î*_*x*_ – *iÎ*_*y*_) operators:
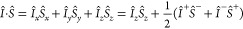
15

**Figure 112:**
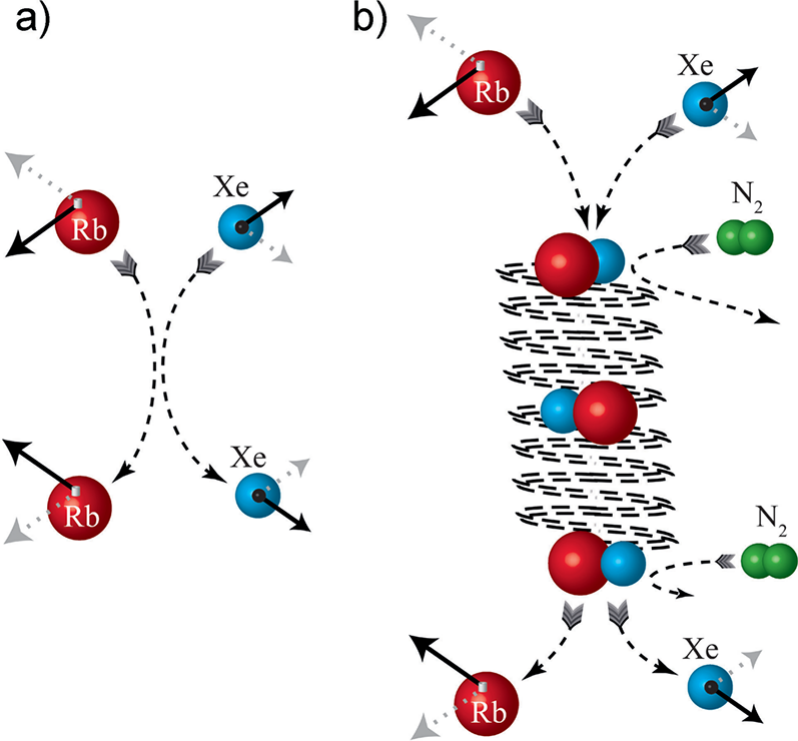
Processes that lead to Fermi contact interaction (see Figure 111) and therefore may result in spin exchange. (a) Binary collisions are not very effective but there are many such events in the gas phase. Furthermore, binary collisions may cause spin exchange even in the presence of a strong magnetic field as the short duration of the coupling leads to a relaxation-like transfer process rather than coherent coupling. (b) Three-body collisions may lead to a short-lived van der Waals complex with high probability of spin exchange during the lifetime τ of the complex.

The terms *Î*^+^*Ŝ*^–^ and *Î*^–^*Ŝ*^+^ are sometimes called flip-flop operators that describe spin exchange. Spin exchange requires that |ψ(*R* = *R*_0_^NG^)|^2^ > 0 and this is the case during binary collisions (Figure 112a) between alkali metal and noble gas atoms in the gas phase. The Fermi contact interaction is particularly effective in three-body collisions, where a third atom or molecule dissipates the energy from the collisions, that can lead to the creation of van der Waals complexes that exist temporarily until a collision with another body causes them to break up again. Figure 112b shows the spin exchange within a van der Waals complex that is typically much more efficient than spin exchange driven by binary collisions. Not all binary collisions and, similarly, not all van der Waals complex interactions result in spin exchange. However, transfer of the electron spin polarization to the nuclear spins will eventually take place as long as there is a sufficient number of successful events. Spin exchange driven by the flip-flop operator does not only cause nuclear spin polarization, but likewise leads to nuclear spin depolarization if depolarized Rb electrons are present. However, as the process takes place under continuous laser irradiation, depolarized Rb electrons are continuously pumped back into their hyperpolarized state and high electron spin polarization can be maintained. For this reason, it is important to avoid areas within the SEOP cell that are obstructed from laser irradiation as this leads to “dark” (i.e., depolarized) rubidium.

Note that the Fermi contact Hamiltonian does not contain any double-quantum terms, i.e., *Î*^+^*Ŝ*^+^ or *Î*^–^*Ŝ*^–^, that would enable a competing two-spin flip-flip process to cause nuclear spin depolarization. Therefore, as long as optical pumping keeps the electron spin polarization at high levels and “dark rubidium” is largely avoided, the Fermi contact interaction effectively leads to a directed transfer of hyperpolarization from the electrons to the noble gas nuclear spins until a steady-state nuclear spin polarization level is reached.

In addition to the fundamental mechanisms of SEOP described above, the time dependence of the process and a quantitative description of the spin polarization deserve further consideration. The Fermi contact interaction drives the spin exchange process that is quantified by the spin exchange rate γ_SE_^931^
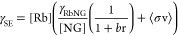
16

The process is driven by two contributions: (1) spin exchange that takes place within short-lived rubidium–noble gas van der Waals complexes in the gas (often termed a “three-body” process, see Figure 112b) that are characterized by the rate constant, γ_RbNG_; and (2) spin exchange during short-duration binary collisions, represented here by the velocity-averaged binary spin exchange cross-section ⟨σv⟩.^931^ Spin exchange from “three-body” process within van der Waals complexes is far more efficient for xenon than Rb–Xe spin exchange through binary collisions. Unfortunately, high noble gas density [NG] reduces the three-body spin exchange rate due to increased collision rates between the van der Waals complexes and the heavy noble gas atoms that reduce the lifetime of these fragile complexes. In eq 16 this effect is expressed through γ_RbNG_/[NG] that provides an important reason to keep the noble gas concentration low. This is in addition to the detrimental effect of high [NG] on Rb spin polarization described by eq 11 and eq 13.

The situation is somewhat different for krypton where a large proportion of spin exchange takes place through binary collisions as three-body events are less effective. However, a high krypton density is still detrimental for the achievable rubidium electron spin polarization, but also because of the ^83^Kr *T*_1n_ relaxation time dependence on [Kr] (see below). Short *T*_1n_ relaxation time is even more of a concern for SEOP of ^131^Xe.

The N_2_ molecules in the SEOP mixture also contribute to the Rb–NG van der Waals dimer breakup and this contribution is quantified by the specific or characteristic pressure ratio *b = P*_0_(NG)/*P*_0_(N_2_) of the gases used^93,931,932^ and by the actual partial pressure ratio r = *P*(N_2_)/*P*(NG) of those gases in a mixture. Note, however, that some nitrogen is always needed for SEOP because of its radiation-quenching effect, unless replaced by other quenching agents such as molecular hydrogen. Lastly, eq 16 suggests that increasing the SEOP temperature increases the exchange rate due to increased rubidium density [Rb]; however, this is offset by detrimental effects on the optical pumping, radiation quenching, and laser light penetration at higher temperatures.

The time-dependent noble gas spin polarization *p*_NG_(t) build-up is described by^921,922^
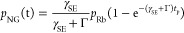
17where *p*_Rb_ is the average value of rubidium polarization within the SEOP cell that replaces *p*_Rb_(z,r) from eq 11 due to gas flow and convection in the pump cell during the buildup of *p*_NG_(t) that can take many minutes.^933^ The achievable polarization *p*_NG_(t) can be affected by Γ, the longitudinal relaxation rate 1/*T*_1n_ of the noble gas atoms. The gas-phase relaxation time *T*_1n_ of the monatomic noble gases can be long: depending on the isotope and specific conditions, it may range from days (in the case of ^3^He) to many hours (^129^Xe, ^21^Ne). However, fast relaxation can be a problem for SEOP with ^83^Kr (*T*_1n_ on the order of minutes at typical noble gas partial pressures around 3–15 kPa) and even more so for ^131^Xe (*T*_1n_ on the order of tens of seconds).

**Figure 113:**
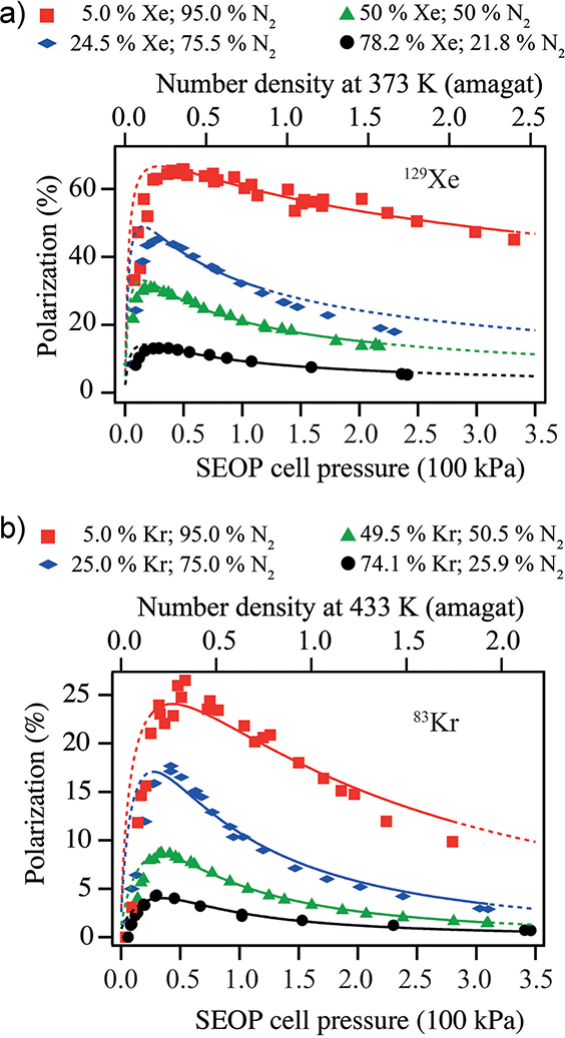
Nuclear spin polarization of ^129^Xe (a) and ^83^Kr (b) as a function of SEOP cell pressure for various mixtures. The polarization values are the maximum polarization at a given noble gas density after the steady state has been reached. Reproduced from ref (925). Copyright 2011 The Authors. Published by PLOS.

The polarization *p*_NG_(t) in eq 17 increases in time during the SEOP process until a steady-state value *p*_NG_ is reached. Operational SEOP conditions that optimize rubidium density [Rb], laser power, the effectiveness of van der Waals complexes, and other factors for a maximum spin exchange rate γ_SE_ will also shorten the pumping process time to reach the steady state. Note, however, that high temperature and even high laser power may have an adverse effect on the rubidium (or other alkali metal) electron spin polarization *p*_Rb_ and, although steady state may be reached faster due to a high γ_SE_, the obtained nuclear spin polarization *p*_NG_ may be lower than the polarization achievable at a lower temperature. The ideal SEOP conditions depend on the alkali metal used for optical pumping, the noble gas isotope, the hyperpolarized noble gas volume that needs to be generated, and practical considerations such as available laser power, total gas pressure, and limitations by the intended applications. However, common to all SEOP is that the presence of the noble gas itself (other than helium and perhaps neon) causes electron spin depolarization of the alkali metal atom that is detrimental to the achievable polarization *p*_NG_. Therefore, the noble gas is usually kept at a low partial pressure around 3–15 kPa. In some SEOP experiments, overall gas pressures below ambient can be utilized, however in the production of hyperpolarized noble gas as NMR probing agent or as MRI contrast agent, SEOP typically takes place at ambient pressures or above. Therefore, the noble gas intended for hyperpolarization is usually highly diluted in buffer gases. For example, the best results for the hyperpolarized ^129^Xe production in clinical pulmonary MRI are currently obtained with SEOP using 1–5% Xe, 5–10% N_2_ balanced by helium.^921,934^Figure 113 shows the dependence of ^129^Xe hyperpolarization on gas pressure and mixture composition in a stopped-flow SEOP experiment.

**Figure 114:**
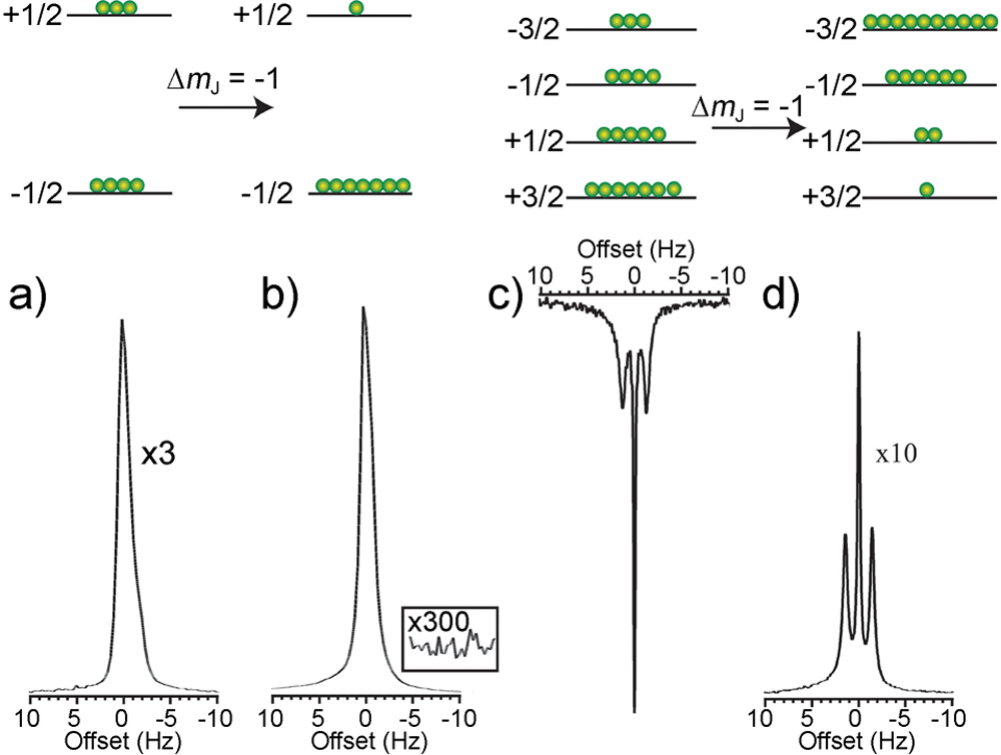
Consequences of the sign of the gyromagnetic ratio. NMR spectra of thermally polarized ^129^Xe (a) and ^131^Xe (c) are shown after individual zero-order phase correction. NMR spectra of hyperpolarized ^129^Xe (b), with the inset showing noise level after 300× magnification, and ^131^Xe (d) after SEOP of the transition Δ*m*_J_ = −1 and application of the same zero-order phase correction as for the thermal spectrum of the respective isotope. For visualization of the underlying process, the associated energy levels and their populations at high-temperature thermal equilibrium (a and c) and after optical pumping using the transition Δ*m*_J_ = −1 (b and d) are also shown. Note that the triplet observed for ^131^Xe in the gas phase is a consequence of quadrupolar couplings. Adapted from ref (918). Copyright 2011 The Authors. Published by Elsevier under CC BY 3.0 license.

It is important to note that SEOP within a high magnetic field region of *B*_0_ ≫ 10 mT effectively suppresses spin exchange in van der Waals complexes. In this case, the less effective binary collisions provide the dominant spin-exchange contribution and thereby reduce the speed of the process for ^129^Xe and the achievable polarization.^935^ A further consideration is the spin state that is populated by the SEOP process (|α⟩ or |β⟩, i.e., parallel or antiparallel with respect to the thermally polarized state) that depends on the choice of the circularly polarized light (i.e., σ^+^ or σ^–^), the direction of the magnetic field in the SEOP process (i.e., parallel or antiparallel, see discussion above), but also on the absolute sign of the gyromagnetic ratio of the noble gas isotope. Note that ^131^Xe is the only stable NMR-active noble gas isotope with a positive gyromagnetic ratio. The effect of the pump direction is illustrated in Figure 114 showing a side-by-side comparison of SEOP with ^129^Xe and ^131^Xe. The spin orientation in typical MRI and most NMR spectroscopy experiments does not affect the experiments substantially. However, in rare and specific experiments using coils with high *Q* factors and condensed hyperpolarized ^129^Xe, radiation-damping effects can trigger depolarization if the spin orientation of the hyperpolarized state is opposite to that of the Boltzmann polarization.^936,937^

The alternative method to hyperpolarize noble gases, namely metastability-exchange optical pumping (MEOP), has been developed since the 1960s,^938^ but the feasibility of this process is mostly limited to ^3^He with its fairly simple electronic structure. However, spin-exchange optical pumping with ^3^He is an intrinsically slow process that typically takes in excess of 12 h to provide ^3^He with *p* = 40% at a 1 L quantity at atmospheric pressure.^93^ In contrast, MEOP enables a more rapid production of ^3^He with *p* = 50–60% at rates of 0.4–1 L/h at atmospheric pressure.^939,940^ Note, the process takes place at a total pressure of a few mbar and the hyperpolarized ^3^He needs to be recompressed to the required pressure for various applications.^941^ If the intended usage is for inhalation as pulmonary MRI contrast agent for example, compression to slightly above ambient pressure is required. Although supply problems may impede widespread medical usage of hyperpolarized ^3^He in the future, the gas is also important as a nuclear target for fundamental physics applications^940^ and the MEOP process shall therefore briefly be described here.

Like SEOP, MEOP is based on transferring angular momentum from circularly polarized photons to electron spins. However, unlike SEOP where the single electron of the outer s-shell in gaseous alkali metal atoms is used, MEOP utilizes the metastable 2 ^3^S_1_ electronic state of helium (^3^He*) generated through plasma discharge induced by radiofrequency irradiation. The 2 ^3^S_1_ state is metastable because relaxation into the 1 ^1^S_0_ ground state is forbidden by the selection rules as a change from a triplet to a singlet state would violate the multiplicity preservation constraint and, furthermore, transition between S terms would violate the requirement for parity change in electronic dipole transitions of centrosymmetric systems. Hence the 2 ^3^S_1_ state can serve as a temporary “ground” state for the optical pumping process. Circularly polarized laser light at a wavelength of 1083 nm pumps the electrons into the various 2 ^3^P_J_ states (*J* = 0, 1, 2). The details of this process are beyond the scope of this review but it is conceptually analogous to the one in SEOP. MEOP takes place in a magnetic field (aligned with the laser beam) that breaks the 2-fold degeneracy of electron *m*_S_ = ±1/2 states of the metastable ^3^He*, separating the corresponding energy levels. Like in SEOP, relaxation processes during optical pumping cause depletion of some of the states and population accumulation in the other states, ultimately leading to high electron spin polarization in metastable ^3^He*. The hyperfine interaction affords a large intra-atomic coupling between the ^3^He* nucleus and its electrons that leads to high nuclear spin polarization. Metastability exchange collisions with ^3^He in the 1 ^1^S ground state transfer this nuclear polarization at a rate that is 4 orders of magnitude higher than the fastest spin exchange between ^3^He and an alkali metal. The process described in eq 18 denotes the nuclear spin states with |α⟩ and |β⟩:
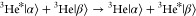
18

Due to its relatively large gyromagnetic ratio γ, ^3^He is more susceptible than all other noble gases to paramagnetic relaxation that exhibits a γ^2^ dependence. Therefore, great care needs to be taken to avoid contact of ^3^He with all paramagnetic surfaces to prevent depolarization during recompression, and specialized containers should be used to transport it. Similarly, ^3^He is more susceptible than other noble gases to relaxation caused by diffusion in strong magnetic field gradients.^942^

#### 3.13.2 Practical Aspects

We now briefly discuss practical implementation of SEOP and concepts for polarizer systems. A continuous wave, circularly polarized (section 2.2.4) laser beam that illuminates the entire region of optical pumping with sufficient power for the specific pumping process is needed. SEOP pressures far below the ambient pressure were utilized in many of the pioneering studies.^927,930,943−945^ However, with the usage of high-power solid-state laser diodes and their inherent broad line width of about 2 nm, pressure broadening of the rubidium D_1_ transition at elevated SEOP gas pressures became advantageous. With further improved laser technology, narrowing of the solid-state laser emission to typically a 0.2 nm line width with an output power of 100 W and above has increasingly become available and affordable.^944,946,947^ Nevertheless, this line width still benefits from some pressure broadening and many of the notable polarizer systems perform SEOP at around 1–2 bar. This pressure range enables straightforward continuous-flow operation but also comes with the additional benefit of a reduced risk of contamination with the ambient air that oxidizes the highly reactive alkali metal within the SEOP cell, effectively reducing, if not eliminating, its functionality.

Some applications require generation of high volumes of up to liter quantities of purified hyperpolarized noble gas at high polarization levels of *p* > 10%, and a number of innovations and studies with continuous-flow SEOP have led to very high spin polarization values of up to *p* = 50% at high production rates, generating a liter quantity within 30 min or less.^921,948−952^ For pulmonary MRI, the production of concentrated hyperpolarized ^129^Xe at ambient pressure at *p*(^129^Xe)>10% polarization and a production rate of several liters per hour at ambient pressure is desirable. Technological improvements have led to systems that significantly exceed these requirements, thus enabling more demanding applications and reducing the need of isotopically enriched ^129^Xe. Most systems utilize SEOP in a continuous-flow mode,^921^ where a lean ^129^Xe gas mixture of typically 1% to 5% ^129^Xe in a buffer gas mixture of 5–10% N_2_, balanced by ^4^He constantly flows through the SEOP cell. The lean mixture maximizes spin exchange efficiency, but the gas flow needs to be sufficiently slow to allow for the desired polarization build-up during the passage of ^129^Xe gas through the SEOP cell. Typically, the passage times are somewhere between 30 s to several minutes. Conflicting with the slow flow rate are two further requirements: (1) the demand for a production rate of several liters at ambient pressure of concentrated hyperpolarized ^129^Xe per hour, and (2) the concentration process that typically utilizes cryogenic capturing of hyperpolarized ^129^Xe in the frozen state as polycrystalline “Xe snow” until the desired amount of hyperpolarized ^129^Xe has been accumulated.^921,948,952,953^ The cryogenic accumulation process is limited by the longitudinal relaxation times of *T*_1n_ = 150 min for ^129^Xe in the polycrystalline “snow” phase that have been reported for 77 K at magnetic field strength of 2.08 T;^45^ however, these field strengths are difficult to achieve, in particular with permanent magnets that are typically used for this purpose, and a more realistic *T*_1n_ = 84 min was reported for a polarizer with hyperpolarized ^129^Xe accumulation at 0.3 T.^934^

**Figure 115:**
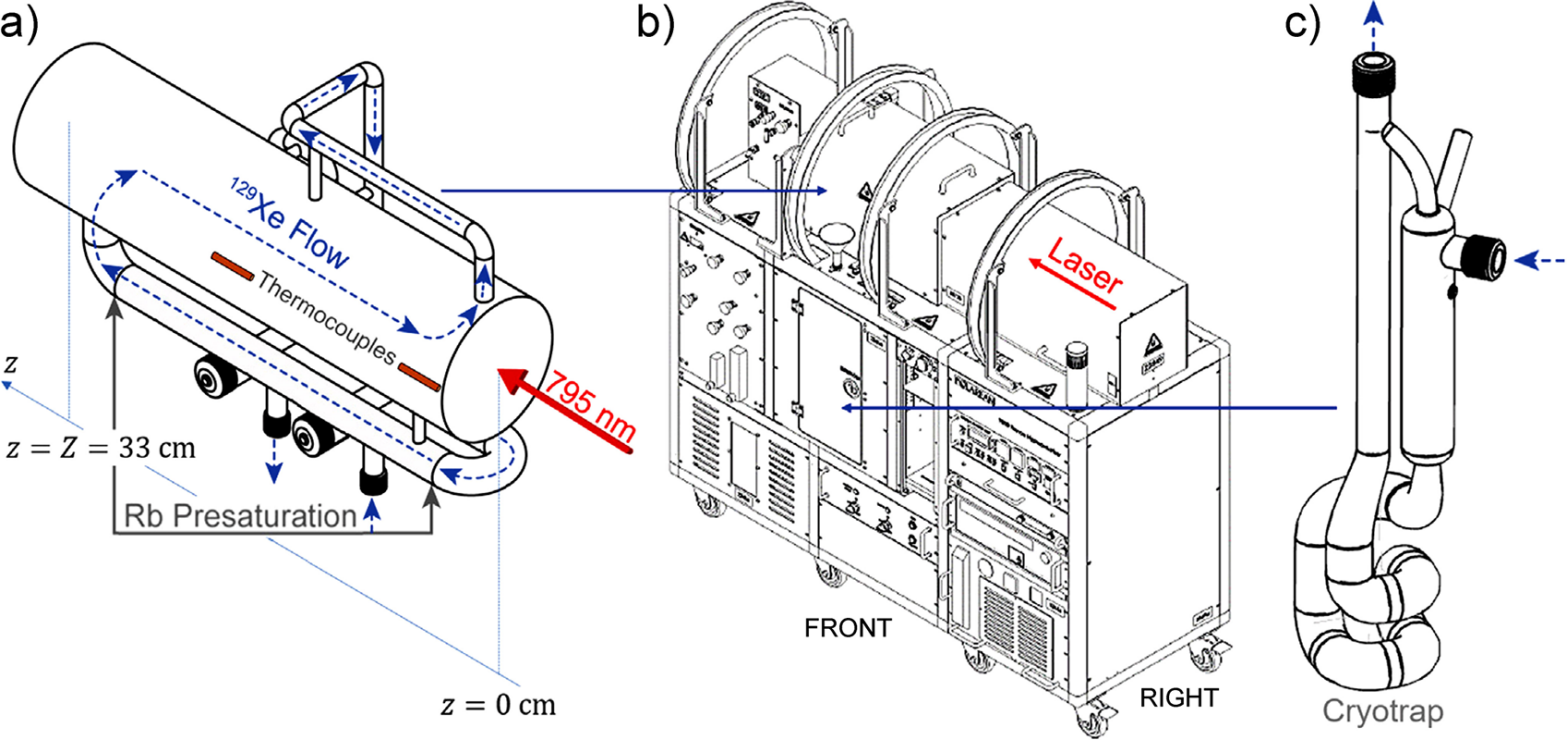
Commercial Polarean 9820-A polarizer as an example of a SEOP system operating in a continuous-flow mode. (a) SEOP cell visualizing the continuous-flow concept. Lean Xe SEOP gas mixture flows through a heated presaturation chamber to take on Rb vapor before entering the actual SEOP cell that is irradiated by the 795 nm laser beam. (b) The location of the SEOP cell within magnetic field produced by four coils. (c) After SEOP in the cell, the hyperpolarized ^129^Xe enters a borosilicate cryotrap kept at the liquid nitrogen temperature of 77 K, where it is cryo-separated from helium and molecular nitrogen buffer gases that pass through the trap without condensation. Reprinted with permission from ref (934). Copyright 2020 Elsevier.

A remarkable, fully automated SEOP system that, including cryogenic separation, generates several liters of hyperpolarized ^129^Xe per hour at a spin polarization *p*_hyp_ = 50% was developed by Hersman and co-workers.^948^ Further promising results were reported by Wild and co-workers who designed a SEOP system with optimized photon efficiency that is capable of producing, for example, 300 mL of cryogenically accumulated hyperpolarized ^129^Xe at *p*(^129^Xe) = 30% within 5 min and one liter of hyperpolarized ^129^Xe at *p*(^129^Xe) = 25% within about 15 min.^954^ Utilizing a commercial polarizer from Polarean, generation of one-liter volumes of hyperpolarized ^129^Xe *p*(^129^Xe) = 30% within 15 min was recently reported.^934^ The authors predict that substantial further improvements are possible if the *T*_1n_ times of cryo-accumulated ^129^Xe can be increased through improving the surface chemistry, better design of the cold trap to maximize its efficiency, and an increase in the applied magnetic field strength. Commercial polarizers (Polarean 9820-A, Durham NC; Figure 115) based on a design by Driehuys and co-workers^955^ are used at various sites for clinical pulmonary MRI research.^934^ Usually, ^129^Xe with isotopic enrichment levels of >90% is being used as it improves the signal intensity about 4-fold over the xenon gas at natural isotopic abundance.

**Figure 116:**
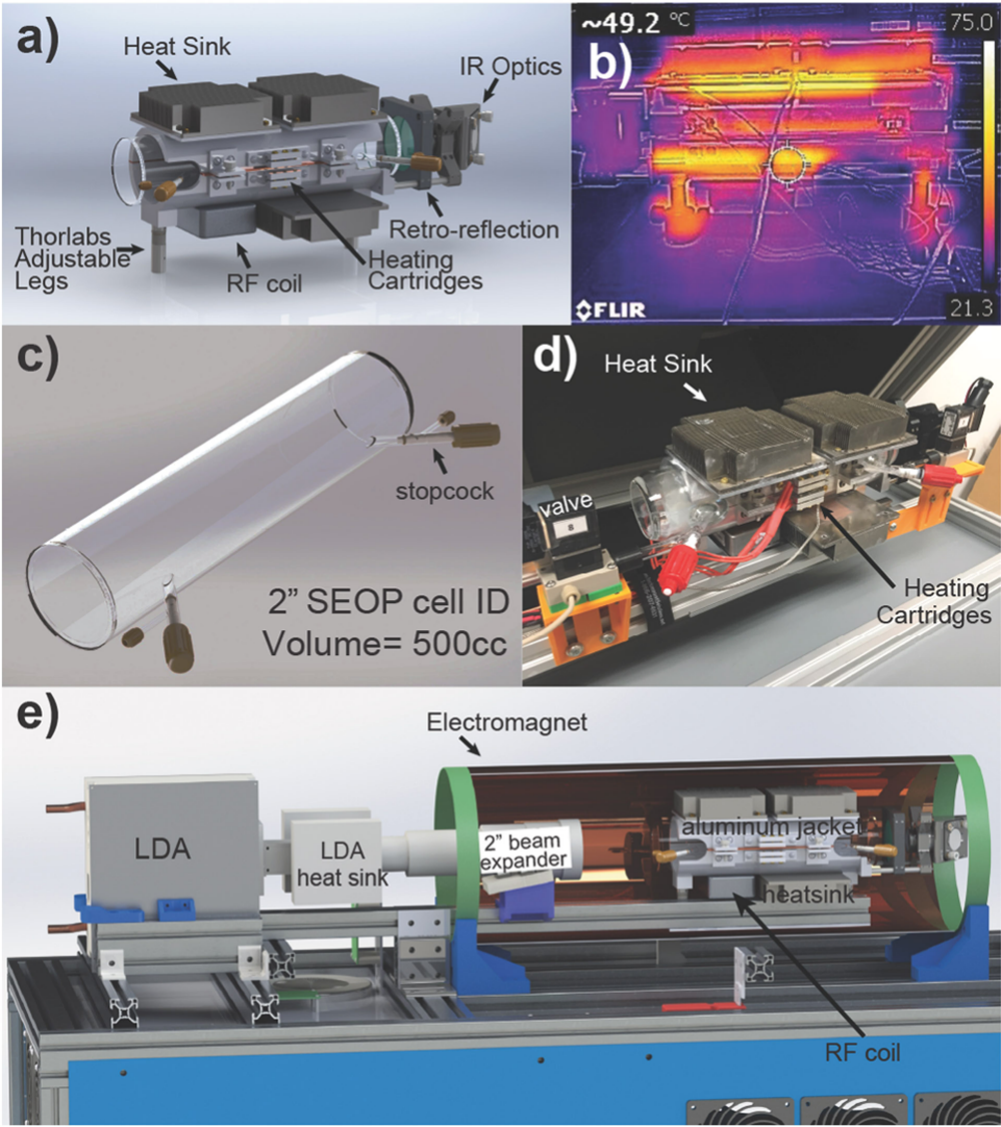
Thermal management system containing the SEOP cell (a) and plain SEOP cell (c) of the commercial XeUS GEN-3 polarizer as an example of a batch mode SEOP system. The aluminum heating jacket and heat sink shown in (a) result in localized temperature distribution as depicted in the thermal image (b). (e) Overall assembly of laser diode array (LDA), solenoid coil for the magnetic field and SEOP cell and thermal management system. Reproduced with permission from ref (958). Copyright 2020 American Chemical Society.

Another approach toward high-volume production of highly polarized ^129^Xe is the stopped-flow polarizer concept, where the SEOP cell is filled with a xenon-rich buffer gas mixture, containing typically 50% of xenon gas. After a set time for SEOP, the cell is cooled to precipitate the alkali metal and the mixture of hyperpolarized ^129^Xe and buffer gas is dispensed in a single batch per SEOP cycle (note, however, that the production is without further concentration of xenon, cryogenic or otherwise, and this simplifies the process, but not without consequences, as discussed below). Remarkably high polarization values of up to *p*(^129^Xe) = 90% have been achieved with this technology^956^ at the cost of reduced ^129^Xe concentration of 17% Xe in N_2_; however, high values of *p*(^129^Xe) = 74% were also achieved in a much more concentrated 50% Xe–N_2_ mixture.^957^ Further advances with the same concept were implemented in a commercial polarizer XeUS (Figure 116) that recently produced *p*(^129^Xe) = 89% in a 50% xenon–buffer gas mixture at a 1 L quantity with a monoexponential build-up rate of γ_SE_ + Γ = 0.045 min^–1^.^958^ According to eq 17, a process with a build-up rate of 0.045 min^–1^ and a steady-state polarization of *p*(^129^Xe) = 89% results in *p*(^129^Xe) = 62% after 30 min of SEOP.

At this point, it is useful to consider the effect of the hyperpolarized ^129^Xe dilution. The signal in NMR is proportional to the product of polarization and the dilution factor *f*; this product has been used to define the apparent polarization *p*_app_ which provides the same signal as a 100% concentrated hyperpolarized ^129^Xe gas with the polarization *p* that equals the *p*_app_ of the dilute mixture:^925^
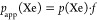
19

For example, *p*(^129^Xe) = 62% obtained after 30 min of SEOP in a 50% xenon–buffer gas mixture equals *p*_app_ = 31% at the same total pressure and temperature which is thus straightforwardly compared with the polarization after cryogenic separation following continuous-flow SEOP.

Another concept incorporates the production volume of hyperpolarized ^129^Xe and is defined as the dose equivalent volumes, DE, to 100% polarized and 100% isotopically enriched and concentrated xenon:^959^
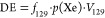
20where *f*_129_ is the isotopic fraction of ^129^Xe and *V*_129_ is the production volume. Essentially, eq 20 informs about the amount of pure ^129^Xe at 100% polarization that provides the same signal intensity as the actual mixture administered. A further approach introduced for hyperpolarized protons is the fully polarized spin concept.^960^ The detailed discussion is beyond the scope of this review. However, whatever concept is used, there is a need for standardization such as the definition of eq 20, in particular, with respect to gas dilution.

Separation of hyperpolarized noble gas is often required after SEOP, and there are alternative processes that achieve this goal. Unlike batch mode SEOP that can utilize high xenon density, SEOP in a continuous-flow mode requires lean mixtures and, therefore, subsequent concentration of the hyperpolarized gas is essential. In practice, the hyperpolarized ^129^Xe–buffer gas mixture is flown through a coldfinger at liquid nitrogen temperature (77 K, at a magnetic field of typically 0.3 T) causing the xenon to solidify while the buffer gases are exhausted. Rapid warming of the coldfinger in a water bath, for example, leads to sublimation of the concentrated ^129^Xe gas with high spin polarization, usually into a Tedlar bag at atmospheric pressure. However, cryogenic separation is inherently a “batch mode” production process that disrupts on-demand continuous flow needed for a number of applications.^961−971^ Indeed, some applications, such as HyperCEST molecular imaging, require very stable polarization conditions and, therefore, the SEOP process should be maintained in a continuous-flow mode by diverting the hyperpolarized ^129^Xe into a bypass line when the delivery to the actual sample is temporarily stopped during data acquisition.^924,972^ Furthermore, cryogenic separation is not an option at all for the production of hyperpolarized ^83^Kr and ^131^Xe due to the fast quadrupolar relaxation of these isotopes with nuclear spin *I* > 1/2. Note that ^3^He and ^21^Ne can be hyperpolarized in highly concentrated form through SEOP in the presence of a small amount of molecular nitrogen for radiation quenching. However, as discussed above, ^83^Kr, ^129^Xe, and ^131^Xe need to be at a low density in order to achieve high spin polarization. An innovative alternative approach is to use an organic compound to serve as a buffer gas that exhibits the following properties: (1) it may not react with the alkali metal, (2) it contains double or triple bonds leading to vibrational frequencies in the molecule that enable for radiation quenching, and (3) it condenses at temperatures far above xenon liquefaction.^964,966,973^ This method produces a constant stream of 70% concentrated hyperpolarized ^129^Xe but the polarization is typically in the *p* = 10% regime. This method, however, is unlikely to be applicable to *I* > 1/2 isotopes. A chemical separation methodology was reported recently that uses molecular hydrogen as a buffer gas and radiation quenching agent.^917,974,975^ Despite having only a single bond, the low mass of the hydrogen molecule leads to a sufficient cross-section for radiation quenching, although about an order of magnitude below that of molecular nitrogen. However, hydrogen has been shown to work well as long as it is not mixed with any other buffer gases.^929^ Following SEOP, the buffer gas is reactively removed through catalytic combustion and the resulting water vapor is condensed at room temperature. This chemical, rather than physical separation process is explored for the production of purified hyperpolarized ^83^Kr as an MRI contrast agent.

#### 3.13.3 Applications

Applications of hyperpolarized noble gases are briefly overviewed below, for hyperpolarized ^129^Xe in particular, and to a lesser account for ^3^He, ^131^Xe, and ^83^Kr, and references to various reviews on the subject are provided as well.

In clinical applications, hyperpolarized ^3^He pulmonary MRI has been increasingly replaced by hyperpolarized ^129^Xe MRI and a number of excellent protocols were developed or adapted from ^3^He MRI to probe different structural and functional parameters of lungs in health and disease.^966,976−982^ Perhaps the most straightforward application of hyperpolarized ^3^He and ^129^Xe are static pulmonary ventilation images that reveal ventilation defects^983^ or the response to airway-hyperresponsiveness challenges in asthma.^984^ MR velocimetry measurements^985^ can be utilized to study gas-phase flow and dynamics in the lung. However, stochastic terms from Brownian motion may be on the same order of magnitude as the coherent term arising from the flow and this can lead to a strong interplay that may lead to averaging of a velocity distribution, for example. Velocimetry experiments in lungs are experimentally demanding since they cannot be performed in a continuous-flow mode; despite that, such measurements were reported with hyperpolarized ^3^He.^986^

Gas diffusion in the respiratory zone of the lungs is restricted by alveolar walls and measurements of the apparent diffusion coefficient (ADC) with hyperpolarized noble gas can provide valuable insights into lung morphometry.^987,988^ Although the ^129^Xe self-diffusion coefficient in air, D(^129^Xe-air) = 0.14 cm^2^ s^–1^, is six times smaller than that of ^3^He in air, D(^3^He-air) = 0.86 cm^2^ s^–1^, the ADC data for both gases in lungs usually correlate well and ADC value is elevated in patients with chronic obstructive pulmonary disease (COPD).^989,990^

Two interesting properties of ^129^Xe, that are almost completely absent in ^3^He, are significant solubility in tissue and large chemical shift range that enables one to distinguish between gas and various dissolved phases. The total range of the ^129^Xe chemical shift, responding to different chemical environments (excluding xenon compounds) is almost 300 ppm depending on the materials and solvents.^991−993^ The chemical shift of ^129^Xe NMR signal in the bulk gas phase increases by about 0.6 ppm/bar in pure xenon and is usually referenced as 0 ppm at the zero pressure limit. The chemical shift of ^129^Xe is useful for pulmonary MRI as chemical-shift-selective MRI of dissolved xenon in lungs is possible due to the significant frequency shift between ^129^Xe in the gas phase (set to 0 ppm), the tissue-dissolved phase (TP) around 195 ppm and xenon interacting with red blood cells (RBC) at around 215 ppm.^980,994^ Although dissolved-phase xenon amounts to only about 1–2% of the total inhaled xenon, such xenon is constantly replenished from the alveolar gas phase through rapid diffusive exchange. This allows for rapid signal averaging in the millisecond regime if chemical-shift-selective excitation of the dissolved phase is utilized.^995,996^

**Figure 117:**
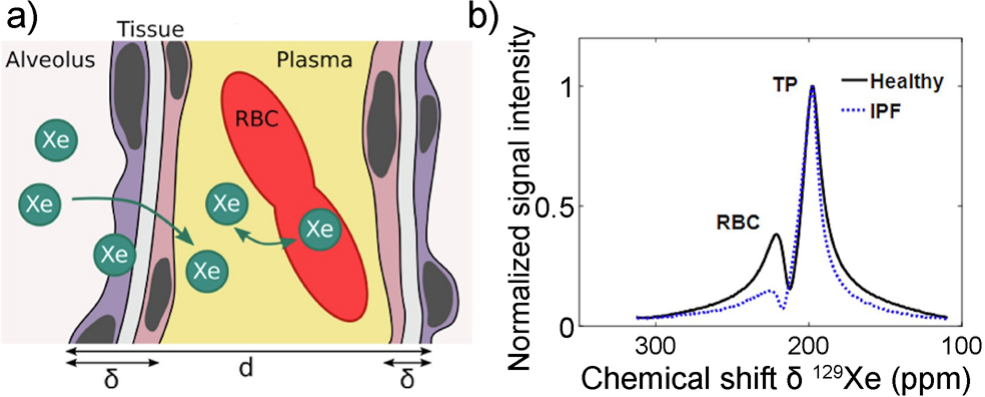
^129^Xe as a biomarker for gas exchange in the lung. (a) Xenon gas in the alveolus resonates at 0 ppm and, after dissolving into the parenchymal tissue barrier (TP, 195 ppm resonance), will transfer to the red blood cells (RBC), where it resonates around 215 ppm. (b) Example of ^129^Xe NMR spectra obtained from a healthy volunteer (black line) and a patient with IPF (blue line) where a reduction of the RBC peak compared to the TP peak is visible. Adapted from ref (980). Copyright 2021 The authors. Published by Elsevier under CC BY 4.0 license.

**Figure 118:**
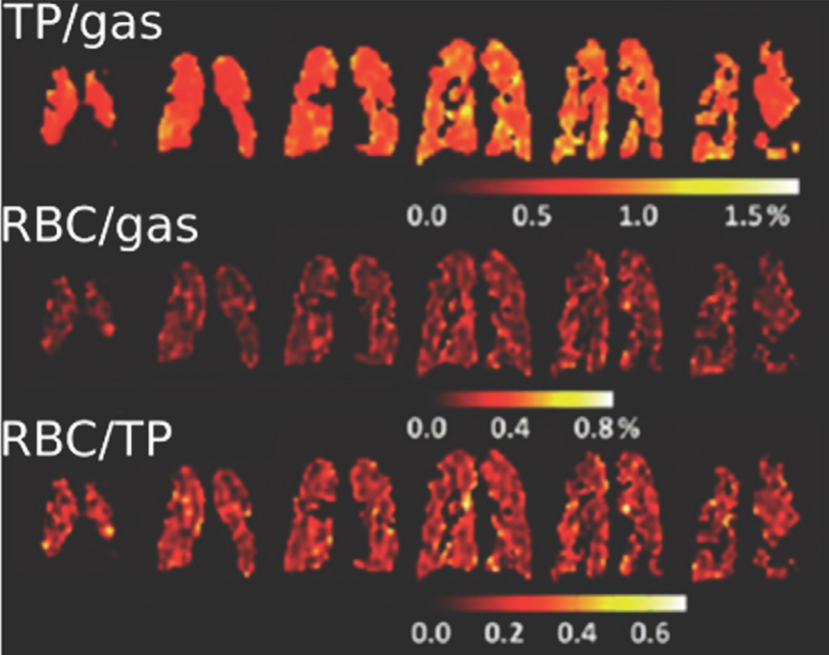
Early example of ratio maps from chemical-shift-selective hyperpolarized ^129^Xe MRI of a patient with moderate COPD. Reproduced with permission from ref (997). Copyright 2013 Wiley Periodicals, Inc.

This concept can be used to probe gas transfer through the lung parenchyma for early diagnosis of interstitial lung diseases such as idiopathic pulmonary fibrosis (IPF) or the monitoring of the scarring of lung tissue related to the long-term effects of the corona virus disease (long-COVID). Generally, scarring will slow oxygen uptake into the blood as the lung parenchyma, i.e., the pulmonary gas–blood barrier, thickens and therefore the breathing process is impeded. The xenon uptake into the blood is therefore a promising biomarker for interstitial lung diseases. Figure 117 shows pulmonary ^129^Xe NMR spectra that demonstrate the concept of ^129^Xe as a biomarker for gas exchange in the lung.^980^Figure 118 shows ratio maps of the various phases in the lung.^997^ Dissolved-phase hyperpolarized ^129^Xe imaging can also be applied as an investigative tool for neuroimaging with initial work performed on rodents.^998−1000^ In clinical work, the NMR spectrum of hyperpolarized ^129^Xe in the human head shows a peak at 189 ppm associated with soft muscular tissue, at 193 ppm from xenon dissolved in white matter, at 196 ppm from xenon dissolved in gray matter, at 200 ppm from xenon dissolved in interstitial and cerebrospinal fluids, and at 217 ppm in red blood cells.^980^

Of particular importance for biomedical applications, including molecular imaging, is the concept of xenon biosensor. Molecular imaging enables the in vivo detection of the spatial distribution of specific target molecules that serve as “biomarkers” in an organism. Molecular imaging seeks to enable early detection of disease, allows for better monitoring of treatment efficacy in personalized or stratified medicine, and is useful for drug development in preclinical and clinical studies. The challenge for molecular MRI comes from low signal intensity typically associated with dilute concentrations of the target molecules and by the complexity of the associated NMR spectra. The ^129^Xe biosensor concept, pioneered by Pines, Wemmer, and co-workers,^968,969,1001,1002^ utilizes an encapsulating agent, such as cryptophane cages, that can reversibly bind Xe atoms with fast rates of exchange. The large chemical shift range of ^129^Xe leads to a distinguishable signal separation between encapsulated xenon atoms in the hydrophobic cavity and xenon in the solvent and is an elegant path to enable molecular MRI^1003^ due to the simplicity of the corresponding NMR spectra. To utilize the biosensor for molecular imaging, biosensor molecules have cages that are linked to bioactive ligands for specific binding affinity for a particular biomarker, i.e., for a specific biomolecule that is upregulated in a particular disease.

The concept is translated from optical imaging with fluorescence markers and the initial work utilized biotin as a ligand for the protein avidin, but the concept was soon extended to peptide–antigen recognition,^1004^ to specific binding to nucleotide targets in the study that demonstrated in vitro recognition of a DNA strand,^1005^ and to cancer biomarkers.^1006^ Since these early studies, a wealth of hyperpolarized ^129^Xe biosensor molecules and concepts were developed, as reviewed elsewhere.^1007−1010^

**Figure 119:**
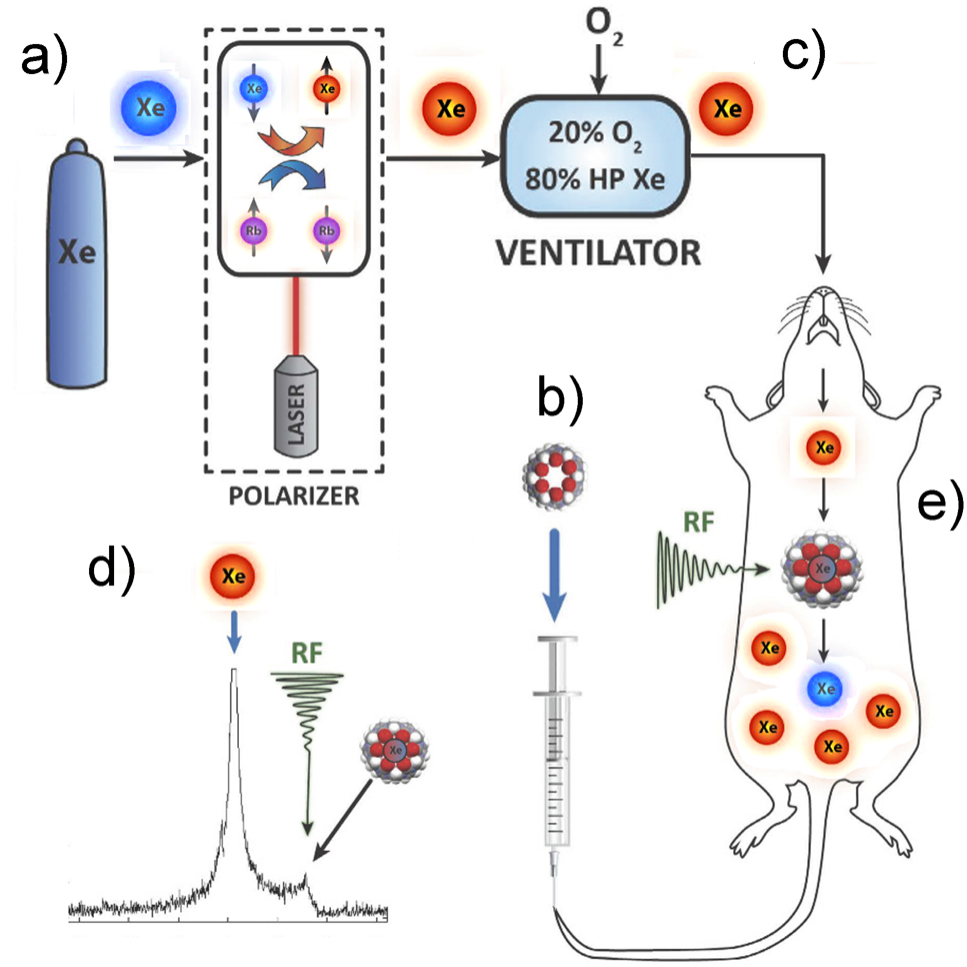
Concept of in vivo molecular imaging with a nonfunctionalized molecular cage used for HyperCEST. (a) Hyperpolarized ^129^Xe is generated through SEOP. (b) A solution containing the molecular cage, i.e., cucurbit[6]uril (CB6), is injected intravenously into the tail vein. (c) After injection of the CB6 and its distribution throughout the bloodstream, a mixture of 80% xenon/20% oxygen is used for mechanical ventilation of a rat. (d) Due to rapid exchange of xenon in the dissolved phase (for example in the blood plasma) with the molecular cage system, the interaction of the hyperpolarized ^129^Xe with CB6 is detected indirectly by the decay of the signal of freely dissolved hyperpolarized ^129^Xe at 0 ppm when the HyperCEST presaturation pulse is applied at the chemical shift frequency of the Xe–CB6 complex at about −70 ppm. (e) Molecular imaging of the CB6 presence is enabled by the presaturation pulse at −70 ppm that reduces the pool of hyperpolarized xenon (depicted in blue, but only in the presence of CB6 cages. This is compared to a control experiment with a presaturation pulse applied at +70 ppm, for example, that does not cause depolarization. Adapted from ref (1012). Copyright 2017 The Authors. Published by Springer Nature under CC BY license.

The sensitivity of the biosensor concept can be significantly amplified further through an indirect detection scheme.^968^ This is achieved via the combination of chemical exchange saturation transfer (CEST) with hyperpolarized ^129^Xe (HyperCEST) that is in rapid exchange between two sites. A sensitivity of tens of nanomolar concentration with antibody-based HyperCEST biosensors for in vitro molecular imaging of cells has been reported.^1011^ Some of the biosensors require a substantial effort in organic synthesis, but a fairly accessible cage system for the development of in vivo HyperCEST molecular imaging protocols is cucurbit[6]uril (CB6).^970^Figure 119 illustrates the concept of HyperCEST molecular imaging where nonfunctionalized CB6 was injected intravenously into a rat to demonstrate a proof of concept that HyperCEST signals can be obtained in a living organism.^1012^ More recently, the concept was optimized further,^1013^ in particular with respect to one of the key requirements of HyperCEST, i.e., high reproducibility of ^129^Xe hyperpolarization and constant concentration during the measurements. Note that a fully working biosensor concept would require functionalization of the HyperCEST agent to serve as a sensor for biomarker molecules, for example, thereby enabling molecular imaging of the biomarker presence. However, at present there is no published study with an actual functionalized cage system that would utilize the HyperCEST biosensor concept in an animal model in vivo.

HyperCEST utilizes exchange between cage-bound xenon atoms with “free” or “pool” xenon atoms of the bulk solution within the vasculature or tissues. A presaturation pulse, applied to the resonance of the xenon-cage complex, depolarizes the bound xenon, but it is the exchange, that is fast compared to the time scale of the presaturation pulse, which causes depletion of the signal of hyperpolarized ^129^Xe in solution. The depletion can be “accumulated” over time as long as irradiation is applied. HyperCEST allows for nanomolar sensitivity because the ^129^Xe signal arising from the bulk solution is much stronger than that from xenon bound to the small number of sensor molecules. Usually, the measured signal depletion is relative to that of a control experiment where the presaturation pulse is applied at a resonance frequency at the same spectral distance to the solution peak but at the opposite side. The important difference to methodology with hyperpolarized molecules is that the biosensor molecules can be injected before the hyperpolarized ^129^Xe is inhaled. Therefore, no relaxation takes place until xenon is inhaled.

**Figure 120:**
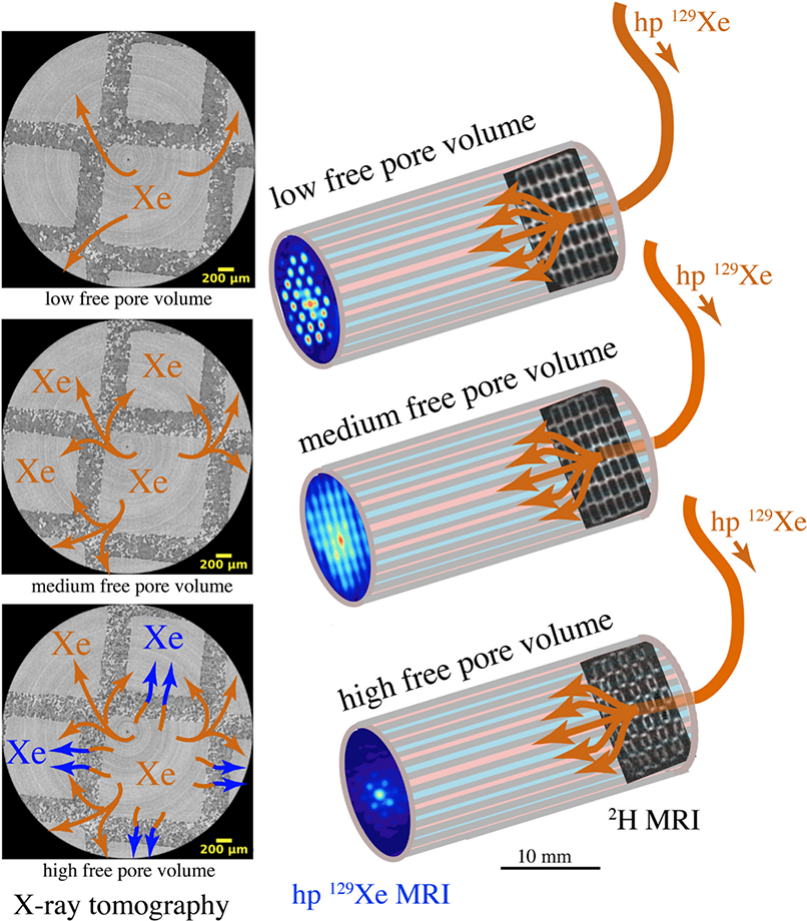
Hyperpolarized ^129^Xe MRI, ^2^H MRI, and X-ray tomography incorporated in a schematic representation of a diesel particulate filter (DPF) catalytic converter. Grayscale images (right side) inform about water (D_2_O) distribution across the whole monolith cross-section using ^2^H MRI. Hyperpolarized (hp) ^129^Xe MRI (color maps) reveals the spatial extent of permeation of xenon away from the central channel at different water saturations (free pore volumes) but also depict hyperpolarized ^129^Xe depolarization at the highest free pore volume that indicates accessibility to paramagnetic catalytic sites. Arrows drawn on the synchrotron-based X-ray tomography images inform about the noble gas pathways at various free pore volume levels that lead to depolarization (blue arrows) when the smallest pores of the catalytically active washcoat, located in the central regions of the monolith walls, become accessible. Adapted with permission from ref (1017). Copyright 2020 Elsevier B.V.

^129^Xe NMR is a useful tool in materials science and engineering applications as well. NMR spectroscopy with thermally polarized ^129^Xe has been extensively applied to the study of porous materials but the strength of hyperpolarized ^129^Xe is in the detection of dynamic processes; see the reviews published on this subject.^966,1014,1015^ A recent example of the study of a dynamic process is the utilization of xenon chemical shift for the observation of the temperature dynamics during the first 100 s of the start-up of a catalytic hydrogenation reaction.^1016^ Another engineering application, investigating the structure-transport relationships in the hierarchical porous structure of a catalytic diesel particulate filter (DPF) monolith, illustrates the (back) translation of MRI technology from medical application to engineering sciences.^1017^ Using transport-weighted MR images of hyperpolarized ^129^Xe, the authors where able to show the locations of high porosity in the regions that are largely void of the actual washcoat catalyst causing most of the gas to bypass the active sites. Similarly, using continuous-flow conditions at reduced water content, the hyperpolarized ^129^Xe MRI signal largely, but not completely, disappeared indicating that the active catalytic sites have become accessible (Figure 120). This observation suggests that paramagnetic (catalytic) centers are mostly located within the smallest pores that become accessible for gases only once almost all of the water in the material has evaporated.

**Figure 121:**
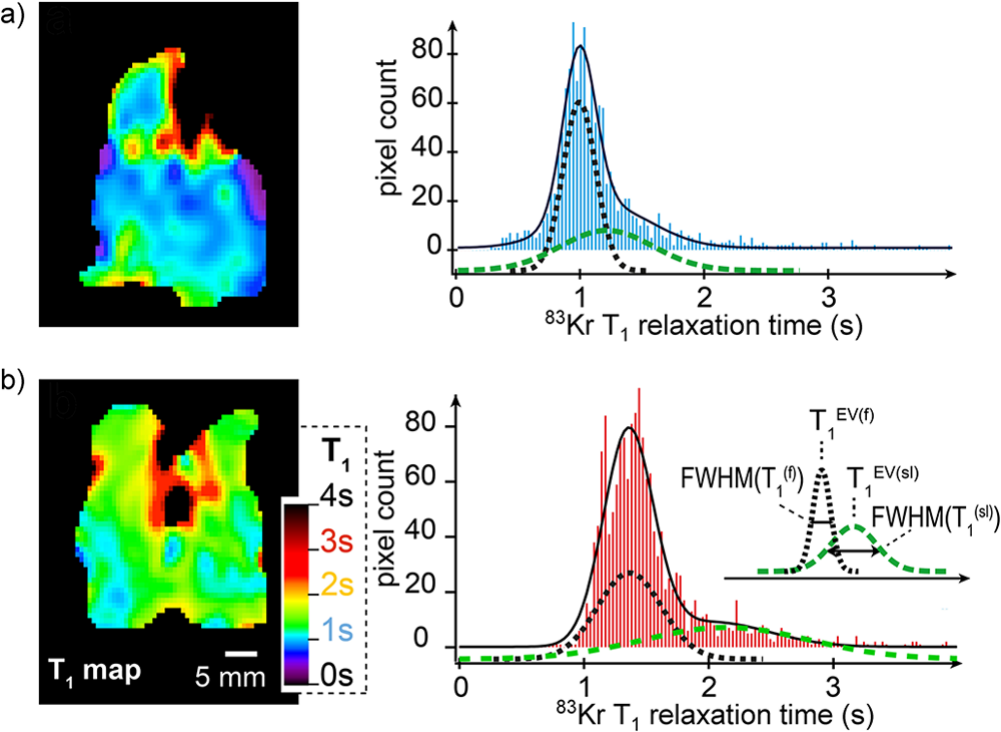
(a) Hyperpolarized ^83^Kr SQUARE *T*_1n_ map of control lung and (b) of emphysema model lung. The frequency of the *T*_1n_, i.e., the pixel (voxel) count with a particular *T*_1n_ value, is shown as the histograms next to the SQUARE maps (labels ’f’ and “sl” refer to the fast and slow components of the bimodal fitting of the distributions, respectively; EV = expected value). There is a clear shift of the *T*_1n_ relaxation times distribution in the histogram of the disease model toward longer times. The inset shows the four parameters that can be extracted from bimodal Gaussian fitting of the histograms: the most probable (or expected) value *T*_1_^EV(f)^ of the fast Gaussian component in the bimodal distribution; the expected value of the slow component *T*_1_^EV(sl)^; and the full width at half-maximum of the fast and slow components, FWHM (*T*_1_^(f)^) and FWHM (*T*_1_^(sl)^), respectively. The two parameters, *T*_1_^EV(f)^ and *T*_1_^EV(sl)^, enabled a statistically significant distinction between the emphysema model and the control lungs. Adapted from ref (1018). Copyright 2015 The Authors. Published by the Royal Society.

Applications of the quadrupolar noble gas isotopes are much less numerous. The high hyperpolarization achievable for ^83^Kr makes this isotope a potential candidate for pulmonary MRI contrast. The ^83^Kr *T*_1n_ times in the pure gas are on the order of minutes and therefore sufficiently long for clinical applications. For tissues with high surface-to-volume ratios S/V, quadrupolar relaxation during surface adsorption of ^83^Kr becomes the dominating relaxation mechanism. A large number of adsorption and desorption events depletes polarization in the gas phase, and this effect is the dominating cause for relaxation observed in the lung. Since the ^83^Kr *T*_1n_ relaxation predominantly occurs at the surfaces, the effect has been dubbed “surface quadrupolar relaxation” or SQUARE. SQUARE causes a S/V-dependent *T*_1n_ contrast leading to ^83^Kr depolarization. Furthermore, SQUARE *T*_1n_ times are also dependent on the chemical composition of the surface. Figure 121 shows hyperpolarized ^83^Kr *T*_1n_ SQUARE maps in an animal model of emphysema where the S/V is reduced and in a control lung. The work suggests that SQUARE of hyperpolarized ^83^Kr is promising as a potential biomarker for the condition.^1018^

Hyperpolarization of electron spins of atomic systems in the gas phase produced by optical pumping is the essence of another important technology which is utilized in optically pumped magnetometers (OPMs). While this application does not involve hyperpolarization of nuclear spins, OPMs are of interest in a range of magnetic resonance applications, including their use as highly sensitive NMR signal detectors. It is, therefore, briefly outlined below.

There are many different types of OPM devices, including ^3^He- and ^4^He-based magnetometers, that have been extensively studied.^1019^ For alkali-metal-based magnetometers, the principle of operation is similar to that shown in Figure 110 where the D_1_ transition between the ^2^S_1/2_ and ^2^P_1/2_ levels is pumped with σ^+^ photons from a powerful light source, typically a laser. Potassium, rubidium, or cesium and their D_1_ transition are common choices. For a full description; however, one needs to consider the hyperfine interaction with the nuclear spins. For instance, for an OPM with the vapor of the rubidium isotope ^87^Rb (*I* = 3/2), the hyperfine coupling between nuclear spin *I* and the electron total angular momentum *J* gives the total angular momentum *F* = 1 or *F* = 2.^1020^ Because of the section rule Δ*m*_F_ = +1, optical pumping in combination with relaxation processes (see discussion of Figure 110) eventually produces an overpopulation of the *m*_F_ = 2 sublevel of the ^2^S_1/2_ state only, which can reach the ultimate limit of 100% in tens of milliseconds. The light transmission depends on the optical pumping rate; for the 100% population of the *m*_F_ = 2 sublevel, no further pumping can take place and the medium becomes optically transparent. In the most straightforward systems, the pump beam is also used as the detection beam, and the intensity of transmitted light is detected with an optical detection system, e.g., a photodiode.

In the simplest case of a single beam, zero-field OPM with no additional magnetic field applied, the magnetometer detects the response of polarized electron spins to an external magnetic field. In the presence of a weak magnetic field that is not aligned with the laser beam, the magnetization vector rotates away from the laser beam direction, and laser pumping recommences. This can be observed through the weakening of the beam transmission. For very weak magnetic fields, the response is linear and the smallest detectable change in the field is
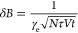
21where *N* is the number density of rubidium atoms, *V* is the cell volume, *t* is the integration time, γ_e_ is the electron gyromagnetic ratio, and the time constant τ is a measure of the dephasing time, i.e., the *T*_2e_ time, but also includes the pumping rate. Depending on the volume of the cell, the alkali metal vapor density, and the relaxation time, experimental sensitivity values in the 10^–16^ T Hz^–1/2^ have been reported, with a predicted theoretical sensitivity as high as 10^–18^ T Hz^–1/2^.^1021^

In an alternative detection scheme, a second (probe) beam perpendicular to the pump beam can be applied. This probe beam can be linearly polarized and the induced rotation of its polarization plane by a magnetic field is used for detection.

OPM devices exhibit high sensitivity to measure weak magnetic fields that is similar to that of superconducting quantum interference device (SQUID)-based magnetometers.^1020,1022^ However, unlike SQUID-based magnetometers, OPM devices do not require cryogenic cooling and the associated cryogenic hardware. OPM technology therefore allows for miniaturization, cost reduction, portability, and enables applications where, at noncryogenic temperatures, close proximity of the magnetometer to the source of the magnetic field is required. OPMs are used in a wide range of magnetic field measurements in geology, archeology, space exploration, magnetic microscopy, and a particularly exciting application is the usage of OPM for magnetoencephalography (MEG) that enables direct imaging of human brain electrophysiology.^1023^

Importantly, OPM-based technology has been explored for the detection of faint NMR signals at zero and ultralow magnetic fields (ZULF) where scalar couplings dominate over Zeeman interactions.^3,1024^ OPM can also be used for higher field strengths, including the Earth magnetic field range. These measurements enable a number of experiments that are otherwise not feasible, for example the NMR study of porous metals or even samples inside a metal container^4^ that would be impermeable for electromagnetic fields at standard NMR frequencies.

#### 3.13.4 Frontiers and Challenges

Future extensions of SEOP and its applications are certainly envisioned, and further progress in this field will be facilitated by overcoming a number of unsolved issues. If utilization of the unique properties of the highly hyperpolarized noble gases during the past decades provides any indication, a wealth of stunning and “never before thought of” applications will continue to emerge. However, the main thrust of applications will likely be in the biomedical field, in particular for pulmonary MRI, the most impactful area of hyperpolarized noble gas utilization to-date. Indeed, pulmonary MRI with hyperpolarized ^129^Xe has already received clinical approval status for a varying degree of usages in at least three countries (in the UK in 2016; in the PR China and the USA in 2022). The study of lungs affected by long-COVID is a timely and prominent example of stunning research enabled by this methodology. However, a successful future for hyperpolarized noble gas MRI in everyday clinical settings demands resolving several outstanding issues. One issue is the standardization of the technology to improve the value of the generated data for radiologists. Furthermore, MRI in general, and hyperpolarized noble gas MRI in particular, are diagnostic methodologies that are not cheap, a problem that is even more pronounced by the fact that simple lung function tests often provide sufficient information at a low cost. Nevertheless, the ability to observe lung function in health and disease with high spatial resolution provides convincing arguments for specialized researchers. However, for more general applications, the polarizer technology must become more user-friendly and more cost-efficient. It is also instructive to look at the rise of general MRI: since the early magnetic resonance experiments that utilized magnetic field gradients for spatial encoding, the success story of MRI was not only due the marvelous and crucial work of visionary physicists, chemists, engineers, and medical researchers in academia and industry. It also relied on one key development outside the world of magnetic resonance and superconductors, namely the availability of ever-increasing computational power at affordable costs. Similarly, the increasing output power of line-narrowed, semiconductor diode array lasers has enabled and improved practical hyperpolarized noble gas MRI over the years. High power of 200 W and more has become available but affordability and reliability of the lasers can still be improved.

Beyond pulmonary MRI, the development of hyperpolarized Xe biosensors is promising but the methodology has only recently made the first steps outside test tubes into in vivo research on animals. Human subject usage will not happen in the near future but will keep generations of researchers busy as potential benefits may be significant. There are only a few engineering applications of hyperpolarized noble gas MRI but this field may benefit from back-translation of medical technology into the physical sciences. Clearly, hyperpolarized noble gas NMR spectroscopy and MRI will continue to benefit materials science and chemistry research for the probing of the structure and transport properties of porous systems. Future work will also seek further advancement of the detection of hyperpolarized noble gases and a broad range of hyperpolarized molecules at low fields with OPM sensors.

### 3.14 Nuclear Spintronics

Beginning in the late 20th century with the discovery of giant magnetoresistance,^1025,1026^ conventional solid-state electronic devices began to exploit the spin of electrons to control currents and interconvert electrical and magnetic signals for novel computational and information storage devices. The era of spintronics was born and is at present the subject of many reviews^1027^ and texts.^1028^ Emerging more recently is the examination of nuclear spintronics^31^ wherein extremely long-lived nuclear spin states, their couplings to electrons and (weakly) other nuclei, are considered as schema for computation, memory, and other informational technology processes.

For three reasons, the development of solid-state nuclear spintronics is closely related to hyperpolarization of nuclear spins. First, inductive signal detection is fraught with poor sensitivity: a robust spintronics device should not require minutes-to-hours of signal averaging. Hence hyperpolarization affords more rapid interrogation of nuclear spins. Second, computation with nuclear spins requires “initial state preparation,” the quantum equivalent of setting all the registers to a specified value in conventional computing: some hyperpolarization schemes may be seen as a way to initiate a specified nuclear polarization. Third, the quantum fluctuations of transverse magnetization, and the incoherent RF excitations of longitudinal magnetization associated with the transverse fluctuations, produce spin noise that decoheres those nuclei or electrons of interest^1029^ that are to be used for computation and information storage and readout.

Further criteria need to be met to imagine nuclear spintronics devices. For example, the DiVincenzo criteria^1030^ for quantum computation requires: (1) qubits (e.g., nuclear and electron spin quantum states); (2) initial state preparation; (3) coherence times long enough for thousands-to-millions of operations; (4) logic gates; and (5) high-fidelity readout. Memory and control spintronic devices pose similar restrictions on the properties of the nuclear spins. All these criteria are common in NMR spectroscopy employing hyperpolarization (though they are given different names, for example, a CNOT gate may be a selective π-pulse in a two-spin system), and thus the NMR community has joined the quantum information processing (QIP) and spintronics communities in discerning solid-state platforms where nuclear hyperpolarization can be generated and controlled with these criteria in mind. NMR, as deployed by chemists for structural determination, was presented for quantum computing in a landmark work,^1031^ and benchmarked against other quantum computing technologies.^1032,1033^ By the end of the 20th century, however, it became clear that liquid-state NMR would not scale to be a useful quantum computing technology.^1034^ On the other hand, there are other ways where nuclear spin states are deployed for quantum technologies.

As seen in section 3.10, hyperpolarization of electrons and ^13^C nuclei at the NV defect in diamond is extensively studied toward these ends owing to the ability to optically prepare high levels of spin polarization and optically read out electron and nuclear spin states where the electrons exhibit millisecond coherence times. Similar progress toward this end is being made with *t*DNP (section 3.9). Here we examine the progress toward nuclear spintronics in two other platforms: compound semiconductors and dopants in silicon.

Laser light of an appropriate wavelength and polarization (section 2.2.4) impinging on compound semiconductors yields nuclear spin polarizations of tens of percent. Dubbed “optical pumping” or “OPNMR,” it has been the subject of many studies.^1035^ At energies close to the band gap (i.e., the electron Bloch wavevector *k* equal to zero), the conduction band wave functions have orbital angular momentum *L* = 0 and electron spin angular momentum *S* = 1/2, for a total electron angular momentum *J* = 1/2. The valence band wave functions have orbital angular momentum *L* = 1 and spin angular momentum *S* = 1/2, for a total angular momentum *J* = 3/2 or *J* = 1/2. The *J* = 1/2 states are “split-off” to lower energy (and are ignored here) so that circularly polarized light with helicity σ^+^ tuned to the band gap (1.52 eV in GaAs) drives transitions from the *m*_J_ = −3/2 valence band state to the *m*_S_ = −1/2 conduction band state and from the *m*_J_ = −1/2 state to the *m*_S_ = +1/2 conduction band state, with relative probabilities of 3:1. Thus, an excess of conduction electrons with *m*_S_ = −1/2 is created. These photoexcited electrons may then be captured at defects, and nuclear spins proximate to the defect are polarized via an Overhauser cross-relaxation between nuclei and electrons. The rate constants associated with this Overhauser process^81^ are affected by the detailed nature of the electron–nuclear couplings (Fermi contact, dipolar, indirect), which in turn may also be affected by the experimental design.^1036^ Subsequent nuclear spin diffusion may carry this local polarization to bulk nuclei. The literature reports that this scheme for nuclear hyperpolarization may also be realized by manipulating the ensemble-averaged expectation value of the electron spin polarization via light modulation,^1036^ application of fields,^97,99^ injection of electrons from magnetic materials,^99,101,1037^ and dipolar order.^1038^ In short, there are several ways to hyperpolarize electron and nuclear spins in these materials, setting the stage for potential quantum devices. GaAs has been a particularly fruitful venue for such demonstrations.

**Figure 122:**
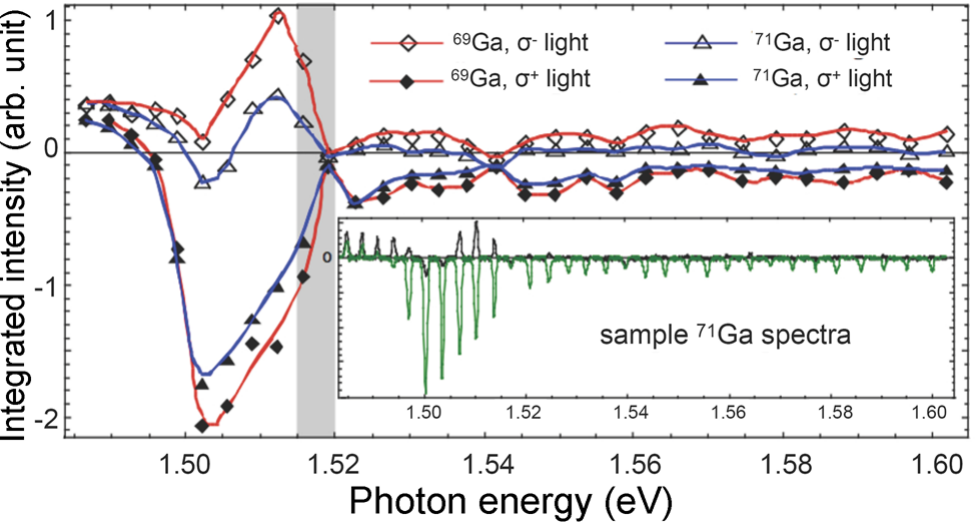
Optical pumping in bulk GaAs as a precursor to the use of nuclei to control local fields. NMR signal intensity is plotted versus the wavelength of light used to excite the bulk semiconductor. The gray band represents the optical band gap of GaAs at the sample temperature, 10 K. The inset at lower right shows a sample data set for ^71^Ga NMR as a function of σ^+^ (green) and σ^–^ (black) photon energy. Here the NMR lines are arranged in order of optical excitation energies. Reprinted with permission from ref (31). Copyright 2010 Elsevier.

Optical pumping in GaAs semiconductors is best illustrated by the “OPNMR profile” where the bulk NMR induction signal from, for example, ^69^Ga, is monitored as a function of optical excitation frequency and light helicity.^31^ “Spectra”, or OPNMR profiles, represent the compilation of bulk NMR signal intensities on the ordinate and laser photon energy on the abscissa, as shown in Figure 122. The magnitude of these ^69,71^Ga NMR signals (as well as those of ^75^As) approach tens of percent polarization, a significant hyperpolarization. These spectra are parsed qualitatively into three regimes based upon the energy of excitation photons relative to the optical band gap of the material: “sub-gap” (<1.505 eV for GaAs), “mid-gap” (1.5–1.52 eV), and “super-gap” (>1.52 eV). Subgap excitation leads to direct pumping to defect sites; midgap pumping yields *m*_S_ = ±1/2 conduction band electrons, depending on the polarization of light, where the electrons are subsequently captured at defects; supergap pumping leads to free electron carriers with spatial trajectories determined by the applied magnetic fields of the NMR instrument. These features appear in the OPNMR profiles of many bulk compound semiconductors besides GaAs.^1035^ Quantitative models for some aspects of these regimes are described in the literature; for example, elegant data^1039,1040^ and a sophisticated model^1041^ describe the supergap OPNMR profiles as variations in optical absorption due to the presence of Landau energy levels for the initial and final photoabsorption electron states. Midgap excitation, however, has been studied most frequently, as described below.

OPNMR in the midgap regime exhibits a maximum in the OPNMR signal (Figure 122). Here the bulk ^69,71^Ga nuclear spin polarization is the result of a competition between two different mechanisms: the so-called “hyperfine” mechanism, producing large polarizations via Overhauser enhancement by captured spin-polarized photoexcited electrons at defect sites,^1042^ and a “quadrupolar” relaxation mechanism that drives nuclear spins to thermal polarizations.^1043^ The rates of both relaxation mechanisms are determined by the concentration of free electrons and shallow donor occupation fraction, which varies throughout the depth of the sample. The resulting competition between these two mechanisms produces some interesting effects.

**Figure 123:**
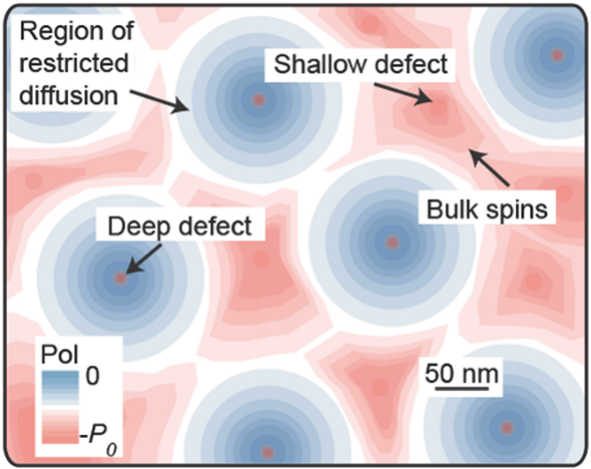
Map of the distribution of nuclear polarization, and thus local fields, in GaAs. Nuclear magnetization diffuses from “shallow defects” to bulk spins while nuclei near “deep defects” remain virtually unpolarized (blue areas). The red dots at the center of the blue circles represent the small fraction of nuclear spins near deep defects directly polarized via OPNMR. Reprinted with permission from ref (1044). Copyright 2013 American Physical Society.

In the regime of low illumination intensities, quadrupolar-driven nuclear spin relaxation^1043^ gives rise to unusual phenomenology by probing the kinetics of the pumping process of the nuclei using a train of light pulses of variable repetition rate.^1044^ Owing to the spectral differences in the NMR response of near-defect and bulk nuclear spins, two classes of recombination centers influencing nuclear spin relaxation were identified. One class comprises shallow defects (i.e., defect characterized by energy levels near in energy to the band edges) preferentially inducing hyperfine-driven nuclear spin hyperpolarization, whereas the other class is formed by deep defect centers (i.e., those characterized by energy levels deep into the band gap) and are mostly responsible for quadrupolar relaxation that diminishes the NMR signal rapidly with time. Fractioning of the illumination time into low duty cycles of light-on/light-off intervals afforded the evolution of the spin polarization over mesoscale distances, where it was found that the defect-centered electric field gradient that induces relaxation has an effective range exceeding several tens of nanometers (Figure 123). Articulating cycles of optical pumping with radio frequency control pulses timed to shape the resulting nuclear polarization as it spreads away from the defect may create “onion-like” patterns in which the polarization successively changes sign or amplitude over concentric layers of predefined thickness. Because the “pitch” in these structures is ultimately influenced by the nuclear spin relaxation, one could articulate experiments that detect trapped charges, discern the spatial profile of hyperfine interactions, or use internal fields to direct electrons in a device.

**Figure 124:**
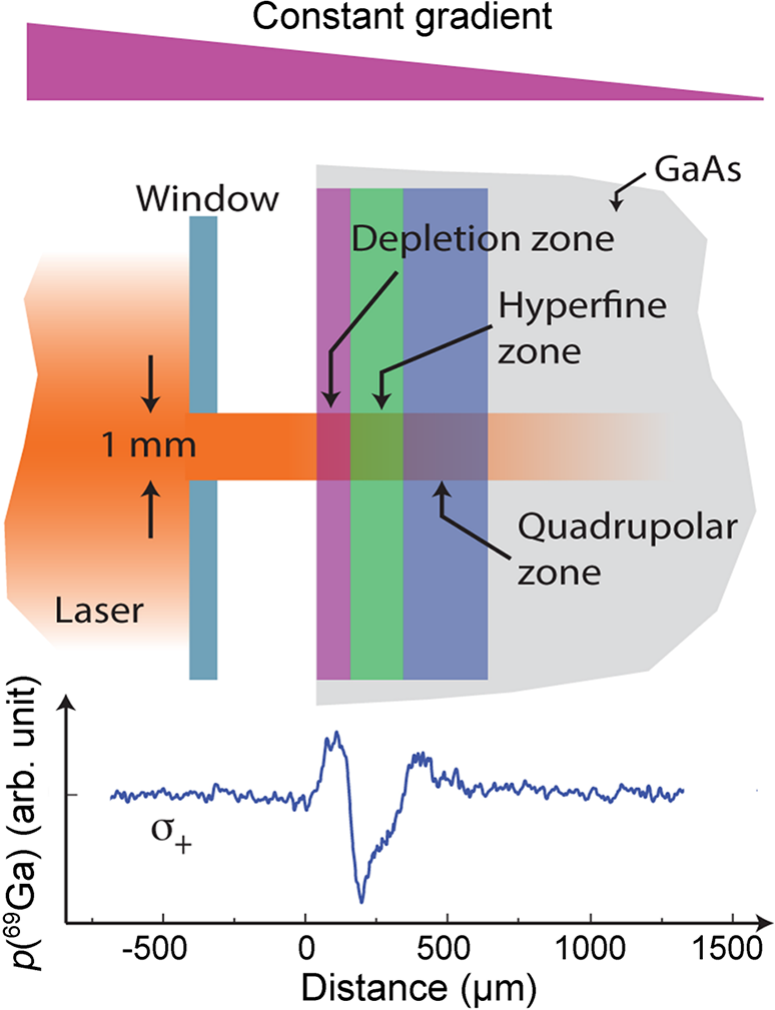
Schematic depicting a 1D NMR image of the spatially dependent nuclear polarization in GaAs. The “depletion zone” represents a region in the wafer where the occupancy of shallow donor sites is limited due to their ionization from surface electric fields. In the “hyperfine zone” the resulting signal is “negative” via hyperfine cross-relaxation with photoexcited electrons captured at defect sites, and the “quadrupolar” zone represents the region in the wafer where optical absorption has reduced the light intensity to the point where shallow donor occupancy is once again limited, leading to nuclear spin thermalization (positive) owing to relaxation from fluctuating electric fields. This spatial patterning of nuclear magnetization emerges with no lithographic techniques nor magnetic materials. Adapted by permission from ref (96), Springer Nature Customer Service Centre GmbH. Copyright 2012 Springer Nature.

A second demonstration of nuclear spin patterning also exploits the two nuclear spin relaxation mechanisms in optically pumped GaAs^96^ where ∼50 μm patterns of ^69^Ga polarization are created with no ferromagnets, no lithographic patterning techniques, nor quantum-confined structures. Here a wafer of GaAs is placed in a high-field NMR instrument with application of a constant gradient for a 1D image of the NMR-active nuclei from front-to-back. After saturating ^69^Ga magnetization with a string of RF pulses, illumination of the wafer with midgap light results in a positive NMR signal near the surface where the quadrupolar relaxation mechanism dominates (defects are all ionized due to surface-derived electric fields, thus no hyperpolarization signal); negative hyperpolarization-enhanced NMR signals appear a few hundred micrometers into the wafer owing to Overhauser cross-relaxation from polarized, defect-bound electrons produced by σ^+^ illumination; and further into the wafer, the light intensity falls so low that once again the quadrupolar relaxation mechanism dominates (Figure 124), resulting in an up-then-down-then-up NMR signal. One goal for such research is to construct patterned nuclear polarization in GaAs where Zeeman fields are spatially controlled for electron-spin-based devices. These Zeeman fields may be used, for example, to selectively tune the resonance frequency of confined electrons. Coherent electron spin rotations have already been achieved^1045^ by causing electrons to drift through a region of magnetized nuclei. The authors surmise that micron-level, three-dimensional patterning of nuclear magnetism would provide a new degree of freedom in semiconductor spintronics that will be easily integrated into existing device architectures with optical and electrical control.

**Figure 125:**
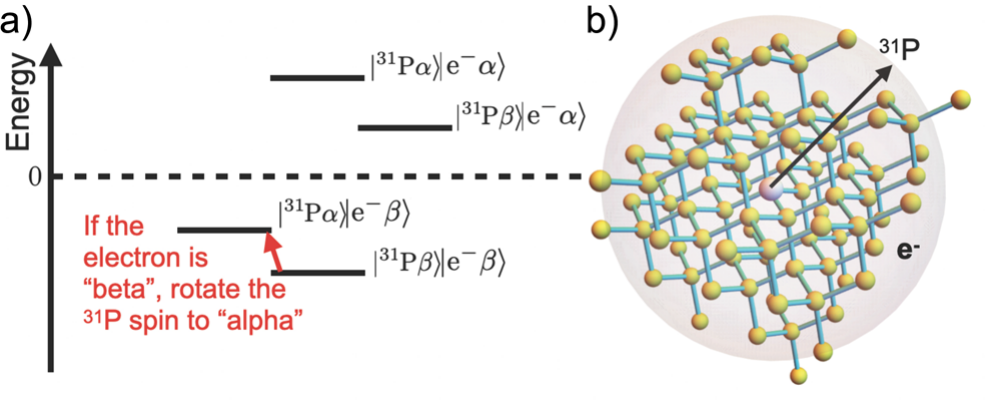
(a) Energy level diagram for the two spin-1/2 particles (an electron and a ^31^P nucleus) at the phosphorus dopant site in crystalline silicon at a given orientation of the crystal in the field. The energy levels are not drawn to scale as the gyromagnetic ratio of the ^31^P nucleus is ∼1600 times smaller than that of the electron. The conditional NOT gate is shown in red: the ^31^P spin is “flipped” only when the electron spin is in the β state. (b) The phosphorus dopant in crystalline silicon (in pink). The Bohr radius of the donor electron associated with this defect is stylized with the shaded sphere; this Bohr radius is about 12 times the carbon–carbon bond length.

At the turn of the 20th century, a novel form of quantum computer was proposed,^1046^ namely the pairing of an electron and a phosphorus atom in crystalline silicon. Here the ^31^P phosphorus dopant in silicon is 4-fold coordinated and is associated with a single unpaired electron that, at cryogenic temperatures, is localized at the phosphorus atom with polarizations on the order of tens of percent. The low temperature and presence of an applied Zeeman field *B*_0_ provide high electron polarization. This structure forms an AX spin system, a classic two-spin system that can be understood and manipulated with NMR/EPR methods. To understand how this works as a quantum computer, we must take a brief aside and consider how the “up or down” spin states of a four-level spin system composed of two coupled spins (Figure 125) may be viewed as a logic gate.^1047^

A logic gate is imagined by considering an operation wherein one of the two spins is flipped conditional on the state of the other spin. The red arrow in Figure 125a demonstrates this operation, namely a selective RF pulse on the ^31^P nucleus. The coupling Hamiltonian for the two-spin system, J*I*_*z*_*S*_*z*_ leads to oscillation of magnetization between the two spins.^1048^ In its simplest form, the controlled NOT (CNOT) gate is just a selective 180°-pulse applied to the ^31^P spin.

**Figure 126:**
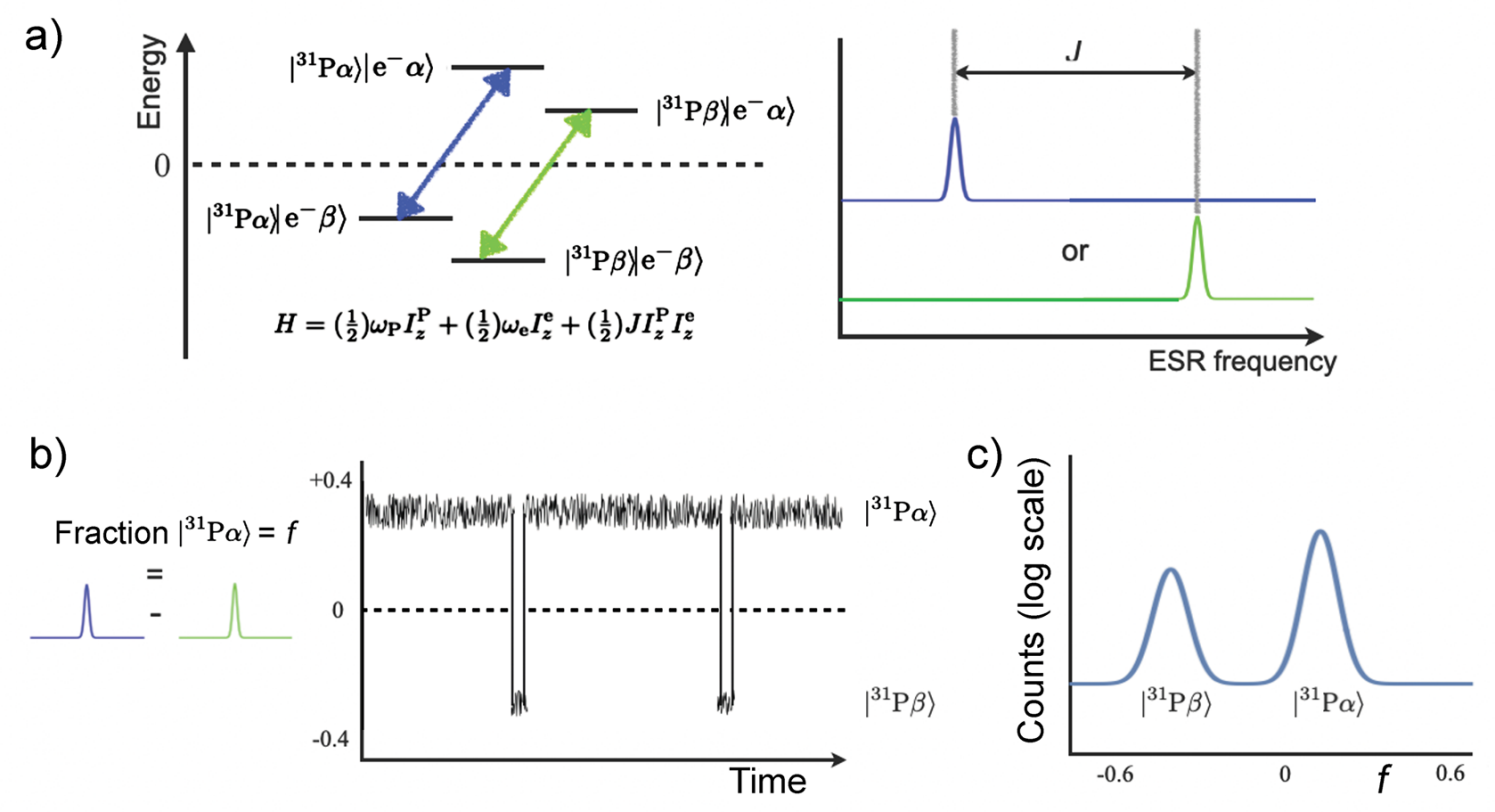
(a) Manifestation of EPR-detected single phosphorus spin resonance. The coupling J splits the EPR transition, yet because the single-spin transistor detection of the EPR signal occurs faster that the nuclear spin flip time, either the blue or green spectrum is detected, but not both. (b) Rapid jumps between the blue and green frequencies yields the fraction of electron spins in one state. Measurements over long periods of time show episodic “jumps” to the other EPR frequency, indicating the nuclear spin has flipped. (c) Binning these flips yields the ^31^P NMR spectrum. Adapted with permission from ref (1051), Springer Nature Customer Service Centre GmbH. Copyright 2013 Springer Nature.

The realization of this structure for quantum computing has been reviewed^1049^ and is the subject of active research, beginning with the construction of a silicon transistor that enables read-out and control of a single electron spin^1050^ provided the construct is held at low temperature (<1 K) and ∼1.7 T magnetic field. A powerful graphic (see Supplementary Movie 1 in ref (1050)) shows how the transistor both initializes and reads out the electron spin via application of dc and microwave fields. A fascinating attribute of this detection scheme is that the electron spin is detected in a time short relative to the nuclear spin flip. Whereas the AX spin system with some coupling between the spins produces a doublet in the ^31^P NMR spectrum and a doublet in the EPR spectrum at a specified orientation of the crystal in a magnetic field, in these experiments only a single EPR peak is observed, with the phosphorus spin being either “up” or “down” (Figure 126), owing to the fact that single spin transistors rely of differing tunneling rates of spin-up and spin-down electrons with applied voltage.^1051^ This strategy remains an active area of research.^1049,1052^

**Figure 127:**
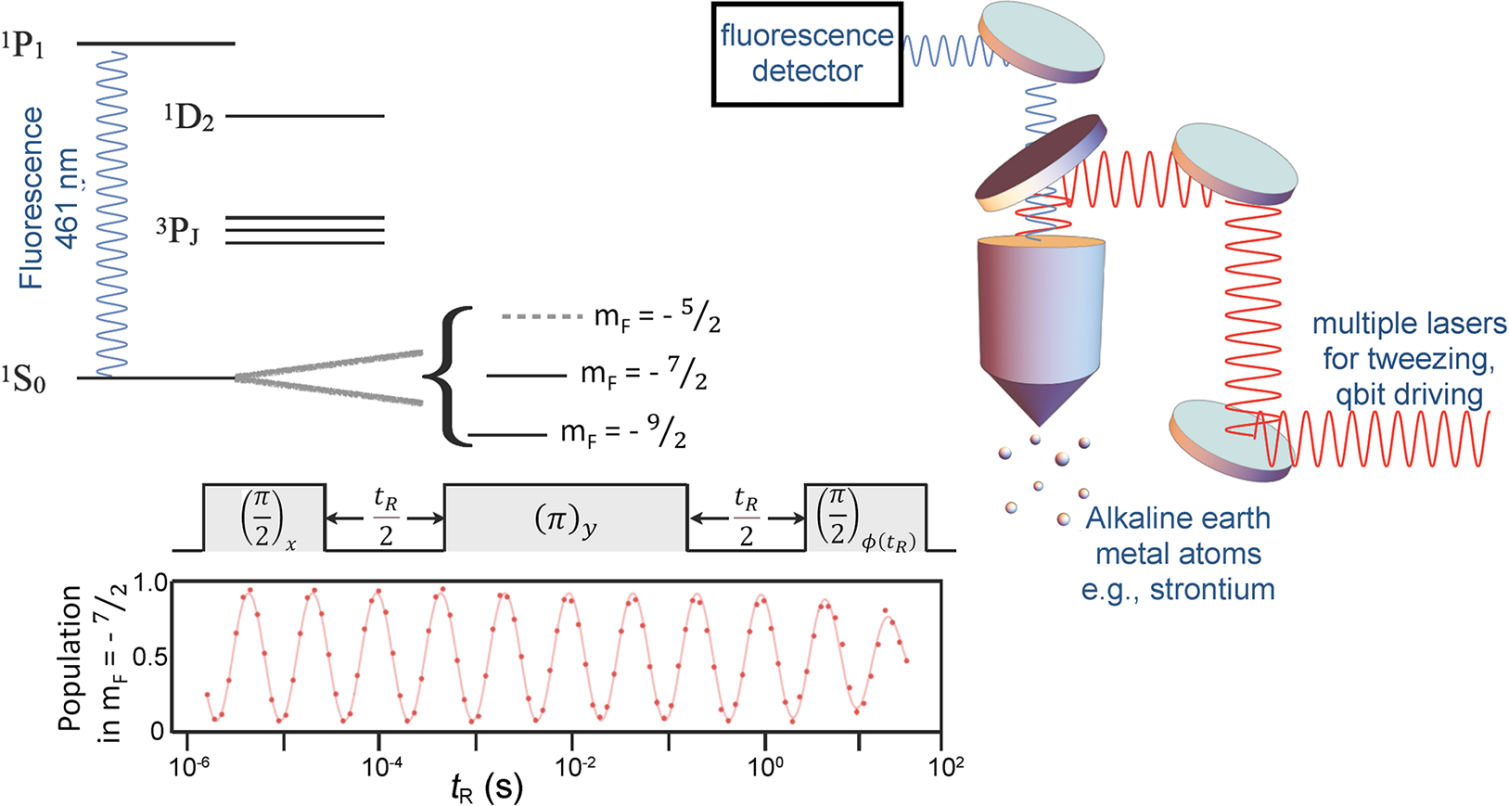
NMR spectroscopy as manifest in ultracold arrays of alkaline earth metal atoms arranged in space with optical tweezers. (a) Jablonski diagram for the ground and excited states of a typical metal atom where the total angular momentum *F⃗* = *I⃗* + *L⃗* + *S⃗*, with *I*, *L*, and *S* being the nuclear spin, orbital, and electron spin angular momenta. (b) Stylized arrangement of lenses, objective, detector, and spatial array of atoms. (c) Echo coherence (“magnetization”) between the nuclear spin sublevels as a function of delay time showing effective spin relaxation times of ∼40 s. See text for details. Adapted from ref (1053). Copyright 2022 The Authors. Published by Springer Nature under CC BY license.

It is important to note that hyperpolarized metal atoms in the gas phase form the basis for quantum information processes, following from Alfred Kastler’s original work.^92^ Here, as with NV defects in diamond (section 3.10), the initialization and readout take place with optical methods, thereby affording great sensitivity. Atoms are arranged in space via optical tweezers, subject to significant cooling (cf. optical pumping of rubidium, section 3.13) then interrogated using fluorescence methods.^1054^Figure 127 shows the Jablonski diagram for a metal atom such as strontium where the levels are labeled according to the total angular momentum *F⃗* = *I⃗* + *L⃗* + *S⃗*, with *I*, *L*, and *S* being the nuclear spin, orbital, and electron spin angular momenta, respectively. In this ingenious scheme,^1053^ Sr atoms are loaded into the optical chamber, imaged to locate them in space, “tweezed” to a specified spatial arrangement, laser-cooled (for example, using light at ∼690 nm) at the ^3^P_J_ to ^1^S_0_ transition, Figure 127a, and then individually manipulated. The readout of the spin state of each qubit (atom) is done via fluorescence at 461 nm. Detection of the nuclear spin is accomplished by driving with a π-pulse the |^1^S_0_, *F* = 9/2, *m*_F_ = −9/2⟩ nuclear spin ground state to the |^3^P_0_, *F* = 9/2, *m*_F_ = −9/2⟩ upper state, thereby placing all the polarization into the upper state; the subsequent fluorescence image will appear darker, yielding selective spin readout. The nuclear spin states of individual atoms are thereby encoded, the prerequisite for nuclear spin quantum computing. This contemporary example demonstrates the synergism between methods to develop and understand optical paths to hyperpolarization (e.g., NV centers) and the emergence of qubits for quantum computing.

## 4 SUMMARY AND OUTLOOK

Nuclear magnetic resonance spectroscopy and imaging are handicapped by low sensitivity, and this is a barrier to many applications. The signal-to-noise ratio achievable in NMR experiments is constantly improving as we employ higher-field NMR magnets, design more sensitive detectors (e.g., cryoprobes), and invent more efficient excitation and acquisition strategies. However, these three streams of development are drying up, and it is still difficult to imagine carrying out NMR spectroscopy on dilute gases in the atmosphere, studying the dynamics of a single protein molecule, or imaging cell cycles or enzyme function in the body.

Hyperpolarization can enhance the signal in an NMR experiment by orders of magnitude, and is already radically shifting the boundaries of what is possible with NMR. Hyperpolarization is almost as old as NMR itself, and the past decades have seen a multitude of hyperpolarization techniques develop and mature at different rates, opening new possibilities along the way. In addition to a quest for a better understanding of hyperpolarization mechanisms and developing more powerful hyperpolarization approaches, some of the major directions in this field are an enhanced analysis of composition, structure and function of complex samples, production and application of useful hyperpolarized agents and tracers, development of ultrasensitive detectors and signal detection schemes for magnetic resonance and beyond, and exploration of new physics and chemistry. For instance, it is now possible to undergo MRI with hyperpolarized tracers in a hospital, whether it be lung imaging with hyperpolarized ^129^Xe, or metabolic imaging with hyperpolarized [1-^13^C]pyruvate. Biomedical applications clearly provide a major impetus for the development of hyperpolarization techniques, and a broader translation of hyperpolarization technology to clinical research and adaptation to clinical practice and environment are certainly warranted. On the spectroscopy side, hyperpolarization has become a widely used tool for protein structure elucidation, surface characterization, materials analysis, and studies of chemical reactions. One exciting current direction is sample miniaturization, from microliter-volume to single-cell, and even single-spin detection schemes. Beyond the realm of applications, new physical phenomena have emerged from the orders-of-magnitude enhanced spin sensitivity, such as the surprising many-body physics of arrays of coupled spins, detectors that probe fundamental cosmological questions, and the complex role symmetry and quantum statistics play in chemical reactions.

Despite all this, there are still fundamental and practical limitations to applications of hyperpolarization. Because of the specificity of the physical and chemical methods employed to create hyperpolarization, there is not a single hyperpolarization technique which has the potential to become a universal solution for each and every need in the near future. The existing techniques differ in many aspects, including the range of hyperpolarizable substances, required instrumentation and experimental conditions, implemented protocols and procedures for preparation and utilization of hyperpolarized substances, applications they target, etc. At the same time, opportunities for cross-fertilization across the field certainly exist: there are numerous common aspects, from fundamental phenomena to key objectives and challenges and experimental implementations. Identifying them can be useful for the entire field to move forward faster.

One of the key challenging goals common to all hyperpolarization techniques is maximizing achievable polarization levels toward the ultimate 100% polarization, with the highest NMR signal enhancement providing much more flexibility in experimental design and information content of such experiments. By now, several techniques have demonstrated the capability of providing polarizations well above 50%, albeit for a select few suitable targets, while other techniques must still overcome various specific challenges and limitations to achieve comparable polarization levels. While there is no common recipe for the entire hyperpolarization field to achieve this goal, there are some general approaches that can be shared for attaining higher polarization levels without compromising utility in the intended applications. In particular, proper design of molecular topology and sample morphology, optimization of sample formulation (e.g., concentrations and distribution of polarizing agents/centers and target spins), and isotopic substitution can be the effective tools. Development of reliable theoretical models is also essential in this respect. While applications such as complex mixture analysis or detection of low-concentrated species aim at achieving the largest NMR signal enhancement, other applications such as production of hyperpolarized tracers may require maximizing molar polarization (the product of polarization and concentration of the target compound) or the production volume.

A common theme for essentially all hyperpolarization techniques is to find ways to polarize a broader range of substances. Once again, while universal solutions elude researchers, examples of approaches that can be used more broadly across the entire field are an introduction of polarizable functionalities by chemical modification, polarization transfer by means of physical (e.g., spin diffusion or NOE) or chemical (e.g., proton exchange) mechanisms from polarized spins to those that cannot be polarized directly, and polarization transfer across interfaces between different materials and different phases.

Another common multifaceted aspect is the transient nature of the hyperpolarized states of spins due to relaxation processes. Relaxation times of spins significantly affect polarization buildup rates as well as the steady-state polarization levels that can be achieved. In particular, long relaxation times may be a disadvantage because the time to achieve maximum polarization levels may become impractically long. At the same time, very short relaxation times during polarization buildup often result in reduced steady-state polarization levels. Sometimes, achieving higher polarization levels at accelerated buildup rates is possible by transferring polarization between spins of different types. Alternatively, adjustment of relaxation times may be desirable to rapidly achieve high levels of hyperpolarization. Similarly, while reduced relaxation times may be beneficial for hyperpolarization production, they are often detrimental for the subsequent use of hyperpolarized substance. To “decouple” the incompatible relaxation requirements of the hyperpolarization preparation and utilization stages, some techniques efficiently modify spin relaxation rates by dilution/trapping/immobilization/filtration/quenching/precipitation of a relaxation agent or by adjusting experimental conditions such as magnetic field, temperature, aggregation state, etc.

Obviously, long relaxation times are advantageous once a hyperpolarized system is produced, to have ample time to observe the enhanced NMR signals of the hyperpolarized substances or the products of their transformation. Furthermore, in many cases hyperpolarization is not produced in situ, and the hyperpolarized substance may need to travel a certain distance before detection, be it a simple sample transfer from a polarizer to an NMR or MRI probe, delivery to a specific tissue/organ in a living organism after administration, or a long-distance transportation of a prepolarized material to an NMR/MRI facility. Relaxation losses during transport can be substantial, and to preserve as much polarization as possible, careful planning and design of the sample composition and conditions (e.g., temperature, magnetic fields) along the delivery path is required.

In many cases, low-γ heteronuclei (e.g., ^13^C, ^15^N) have much longer relaxation times compared to ^1^H, and therefore polarization transfer to such nuclei is a common approach for extending hyperpolarization lifetime in a condensed phase. We note, however, that for gases the situation may be quite different. In contrast to ^3^He and ^129^Xe characterized by very long relaxation times, for polyatomic molecules in the gas phase the relaxation is in general faster than in condensed phase, and relaxation of heteronuclei may be much faster than that of protons.

There is also a remarkable possibility to extend polarization lifetimes beyond the conventional times of spin relaxation through the use of the unique properties of long-lived spin states, in which a correlated spin state is often protected by molecular symmetry and thus preserved for times far exceeding the conventional spin relaxation times. While this approach is not universal, it is certainly worth exploring when one expects long hyperpolarized sample delivery times and/or intends to monitor spin dynamics or sample evolution over extended periods of time.

Another common challenge is the most efficient use of hyperpolarization after it is created. In multishot experiments such as multidimensional NMR or kinetic/imaging studies, small flip angles are often used to keep sufficient polarization for subsequent excitations. An alternative approach is based on sample repolarization (in situ or with sample recirculation or shuttling) or a continuous hyperpolarization production (with or without refreshing the sample), often requiring a high degree of polarization reproducibility. Some rapid MR imaging sequences rely on the formation of a steady-state for sample magnetization; they are designed for spin systems which are initially at thermal equilibrium. For a hyperpolarized initial state, formation of a true steady-state is not possible unless hyperpolarization can be generated continuously during NMR signal detection. Such sequences may need to be redesigned for their efficient application to hyperpolarized samples. An antiphase NMR signal shape produced by some of the techniques results in a partial signal cancelation upon its broadening and, if not accounted for, can be particularly devastating in the presence of strong magnetic field gradients used in MRI.

As the majority of techniques cannot be applied efficiently to pure chemicals, it is often desirable or even mandatory to purify hyperpolarized substances after they are produced. In this respect, applications in vivo are likely the most demanding in terms of sample purity and biocompatibility. Free (bi) radicals and transition metal complexes are the examples of the sample components essential for some hyperpolarization processes but undesirable in the final sample. Various strategies to remove or neutralize them are being developed, including immobilization on porous solids with subsequent filtration, phase separation, quenching, scavenging with properly functionalized sorbents, etc. In addition, organic solvents, which are potentially toxic, often yield substantially higher polarization levels than obtained in an aqueous medium. Thus, extraction of the hyperpolarized compound accompanied by phase separation or purification by precipitation may need to be implemented. The presence of other impurities may need to be carefully considered, including side products and unreacted material in the chemistry-based hyperpolarization process.

Going further, a broader range of accessible experimental conditions is desired, both for production and for utilization/observation of hyperpolarization. Often, an increase in polarization levels may be achieved by going to more challenging conditions (e.g., cryogenic temperatures, elevated pressures, stronger magnetic fields, larger sample irradiation power), and this is certainly warranted for state-of-the-art experiments or for operation of specialized hyperpolarization facilities that may replace on-site production of hyperpolarized agents that sustain long hyperpolarization lifetimes. In addition, while hyperpolarization may not require higher magnetic fields to achieve stronger spin polarization, advanced NMR and MRI instrumentation is nevertheless attractive for achieving the increased spectral and spatial resolution. At the same time, to promote accessibility of the hyperpolarization techniques for everyday practice, they need to be made more affordable. Thus, further efforts are required to reduce the cost of equipment and make it compact, reduce consumption of expensive consumables, implement milder (e.g., ambient) conditions for delicate samples, provide higher turnover rates, etc. Conjugation with benchtop NMR spectrometers and other compact and/or portable NMR and MRI devices for signal detection is becoming increasingly popular. Furthermore, hyperpolarization provides the required sensitivity enhancement for performing NMR experiments at zero-to-ultralow magnetic fields (ZULF NMR), a rapidly emerging magnetic resonance modality with a broad range of new and exciting possibilities.

One of the more challenging aspects of hyperpolarization when applied to sample analysis is in retaining the quantitative aspect of NMR. This means creating equal polarization levels of different components in a hyperpolarized sample, and even perhaps achieving the same polarization levels between samples polarized on different days. This challenge is further complicated by relaxation: even if quantitative polarization levels on all spins in an ensemble can be achieved, they will relax at different rates prior to signal acquisition, leading again to unequal polarizations. It may be expedient to first target a pseudoquantitative approach, in which the polarization levels of all spins in a mixture are not equal, but the proportional polarization on each species is known in advance and can be factored in.

There is thus plenty of room for further optimization of the existing hyperpolarization techniques and making them more universal, including development of more efficient polarizing agents, refinement of the hyperpolarization equipment and protocols, and minimization of losses at all stages, i.e., during production, delivery, sample purification, quality control and utilization. Yet one of the objectives of this review is to demonstrate that, while the number of known sources of hyperpolarization remains quite limited (section 2.2; Figure 3), introduction of new hyperpolarization techniques is inevitable through the broader and more sophisticated uses of available hyperpolarization sources. Such new possibilities, overlooked so far, will certainly emerge. In particular, polarization sources like circularly polarized light and nuclear spin isomers of polyatomic molecules currently appear underutilized.

Given the remarkable fundamental and practical developments outlined in this review, it is clear that this field will continue expanding, contributing substantive new scholarship in spin fundamentals, as well as new technologies for implementation and applications. New applications and discoveries remind us that NMR, though an old methodology, remains young in its potential for new science.

## Supporting Information Available

Abbreviations and notation used (PDF)
