# Supplementary material for: Spin Hyperpolarization in Modern Magnetic Resonance
Source: Chem Rev. 2023 Jan 26;123(4):1417–551. doi: 10.1021/acs.chemrev.2c00534 (PMC9951229; doi:10.1021/acs.chemrev.2c00534)
Supplement: Supplementary file 1 — cr2c00534_si_001.pdf [file cr2c00534_si_001.pdf]

Spin Hyperpolarization in Modern Magnetic Resonance

James Eills[a*], Dmitry Budker[b,c,d], Silvia Cavagnero[e], Eduard Y. Chekmenev[f,g], Stuart J. Elliott[h], Sami Jannin[i], Anne Lesage[i], Jörg Matysik[j], Thomas Meersmann[k], Thomas Prisner[l], Jeffrey A. Reimer[m], Hanming Yang[e], and Igor V. Koptyug[n*]

[a]Institute for Bioengineering of Catalonia, Barcelona Institute of Science and Technology, 08028 Barcelona, Spain

[b]Johannes Gutenberg-Universität Mainz, 55128 Mainz, Germany

[c]Helmholtz-Institut, GSI Helmholtzzentrum für Schwerionenforschung, 55128 Mainz, Germany

[d]Department of Physics, UC Berkeley, Berkeley, California 94720, United States

[e]Department of Chemistry, University of Wisconsin, Madison, Madison, Wisconsin 53706, United States

[f]Department of Chemistry, Integrative Biosciences (IBio), Karmanos Cancer Institute (KCI), Wayne State University, Detroit, Michigan 48202, United States

[g]Russian Academy of Sciences, Moscow 119991, Russia

[h]Molecular Sciences Research Hub, Imperial College London, London W12 0BZ, United Kingdom

[i]Université de Lyon, CNRS, ENS Lyon, Université Lyon 1, Centre de RMN à Hauts Champs de Lyon, 69100 Villeurbanne, France

[j]Institut für Analytische Chemie, Universität Leipzig, 04103 Leipzig, Germany

[k]Sir Peter Mansfield Imaging Centre, University Park, School of Medicine, University of Nottingham, Nottingham, NG7 2RD, United Kingdom

[l]Institute of Physical and Theoretical Chemistry and Center of Biomolecular Magnetic Resonance, Goethe University Frankfurt, 60438 Frankfurt am Main, Germany

[m]Department of Chemical and Biomolecular Engineering, UC Berkeley, and Materials Science Division, Lawrence Berkeley National Laboratory, Berkeley, California 94720, United States

[n]International Tomography Center, Siberian Branch of the Russian Academy of Sciences, 630090 Novosibirsk, Russia

*Email: jeills@ibecbarcelona.eu

*Email: koptyug@tomo.nsc.ru

CONTENTS

ABBREVIATIONS S2

NOTATION S8

ABBREVIATIONS

2D - two-dimensional

ACQ - signal acquisition

ADC - apparent diffusion coefficient

ALTADENA - adiabatic longitudinal transport after dissociation engenders nuclear alignment

API - active pharmaceutical ingredient

APSOC - adiabatic passage spin order conversion

ASD - amorphous solid dispersion

aSLIC - adiabatic spin-lock induced crossing

BDPA - 1,3-bis(diphenylene)-2-phenylallyl (stable free radical)

BQ - *para*-benzoquinone

bTbK - bis-TEMPO-bisketal

CAT - catalase enzyme

CB6 - cucurbit[6]uril

CE - cross effect

CEST - chemical exchange saturation transfer

CIDEP - chemically induced dynamic electron polarization

CIDNP - chemically induced dynamic nuclear polarization

CISS - chirality-induced spin selectivity

CNOT - controlled NOT

COD - 1,5-cyclooctadiene

COPD - chronic obstructive pulmonary disease

COSY - correlation spectroscopy

COVID - coronavirus disease 2019

CP - cross-polarization

CPMG - Carr-Purcell-Meiboom-Gill

CSA - chemical shift anisotropy

CTAB - cetyltrimethylammonium bromide

CT-SE - constant-time spin-echo

CT-SE-HSQC - constant-time sensitivity-enhanced HSQC

CVD - chemical vapor deposition

CW - continuous-wave (irradiation)

CW-TR-EPR - continuous-wave time-resolved EPR

DC - direct current

DD - differential decay

*d*DNP - dissolution dynamic nuclear polarization

DE - diethyl ether

DE - dose equivalent volume

DEPT - distortionless enhancement by polarization transfer

DMA - dimethylaniline

DMPC - dimyristoylphosphatidylcholine

DMSO - dimethyl sulfoxide

DNP - dynamic nuclear polarization

DPF - diesel particulate filter

DPP - 6,13-diphenylpentacene

dppb - 1,4-bis(diphenylphosphino)butane

DQ - double-quantum (transition or coherence)

D-QRIP - dissolution quantum rotor induced polarization

DTPA - diethylenetriaminepentaacetic acid

DXA - molecules encompassing covalently linked electron-donor-bridge-acceptor

ELDOR - electron-electron double resonance

EPR - electron paramagnetic resonance (a.k.a., electron spin resonance, ESR)

ESLAC - excited-state avoided energy levels crossing (level anticrossing)

ESPT - electron spin polarization transfer

EVE - ethyl vinyl ether

FID - free induction decay

FLASH - fast low-angle shot (MRI pulse sequence)

FMISO - fluoromisonidazole

FMN - flavin mononucleotide

$FMNH_2$ - 1,5-dihydroriboflavin 5'-(dihydrogen phosphate)

FOV - field of view

FTS - Fischer-Tropsch synthesis

FT-TR-EPR pulsed Fourier-transform time-resolved EPR

GFP - green fluorescent protein

GMP - guanosine monophosphate

GO - glucose oxidase enzyme

gS2hM - generalized singlet-to-heteronuclear-magnetization

gS2M - generalized singlet-to-magnetization

GSLAC ground-state avoided energy levels crossing (level anticrossing)

HCP- hexagonal close-packed

HEP - hydroxyethyl propionate

HET - heterogeneous

HET-PHIP - parahydrogen-induced polarization in heterogeneous reactions

HET-PHIP-SAH - heterogeneous parahydrogen-induced polarization via side-arm hydrogenation

HFC - hyperfine coupling

HFI - hyperfine interaction

HH-ONP - Hartmann-Hahn optical nuclear polarization

His - L-histidine

HMQC - heteronuclear multi-quantum correlation/coherence

HPHT - high-pressure high-temperature (synthetic diamond)

HPLC - high-performance liquid chromatography

HSQC - heteronuclear single-quantum correlation/coherence

HyperCEST - chemical exchange saturation transfer with hyperpolarization

HYPOP - hyperpolarizing polymers

HYPSO - hybrid polarizing solids

IC - internal conversion

IMes - 1,3-bis(2,4,6-trimethylphenyl)imidazol-2-ylidene

INEPT - insensitive nuclei enhanced by polarization transfer

IPF - idiopathic pulmonary fibrosis

IR - infrared (spectroscopy)

ISC - intersystem crossing

ISE - integrated solid effect; integrated cross-polarization

IWI - incipient wetness impregnation

LAC - avoided energy levels crossing; level anticrossing

LC-photo-CIDNP - low-concentration photochemically induced dynamic nuclear polarization

LDA - laser diode array

LED - light-emitting diode

LFTM - low-field thermal mixing

LID - light-induced drift (of molecules)

LIGHT-SABRE - low-irradiation generation of high-tesla SABRE

LLSS - long-lived spin states

LOV - light-oxygen-voltage-sensing

MAS - magic-angle spinning

MDB - α-methyldeoxybenzoin

MeIM - 2-methylimidazolate

MEOP - metastability-exchange optical pumping

MFC - mass-flow controller

MI-ONP - microwave-induced optical nuclear polarization

MNZ - metronidazole

MOF - metal-organic framework

MR - magnetic resonance

MRI - magnetic resonance imaging

MRS - magnetic resonance spectroscopy (in vivo)

MRSI - magnetic resonance spectroscopic imaging

MSN - mesoporous silica nanoparticle

MTZ - 1-methyl-1,2,3-triazole

MW - microwave (irradiation)

MWCNT - multi-walled carbon nanotube

NC - nanocrystal

NG - noble gas

NMR - nuclear magnetic resonance

NOE - nuclear Overhauser effect

NOESY - nuclear Overhauser effect spectroscopy

NOVEL - nuclear orientation via electron spin locking

NQR - nuclear quadrupole resonance

NR - nitrate reductase enzyme

NSC - nuclear spin conversion

NSIM - nuclear spin isomers of molecules

NV - (negatively charged) nitrogen-vacancy color center

NV-DNP - nuclear polarization using NV centers

ODIP - orthodeuterium-induced polarization

OE - Overhauser effect

OE-DNP - Overhauser-enhanced DNP

ONP - optical nuclear polarization

OP - optical pumping

OPM - optically pumped magnetometer

OPNMR - optical pumping NMR

OPR - ortho-para ratio

OPSY - only parahydrogen spectroscopy

OTP - ortho-terphenyl

PASADENA - parahydrogen and synthesis allow dramatically enhanced nuclear alignment

PASS-PIETA - phase-adjusted spinning sidebands - phase-incremented echo-train acquisition

PDMS - polydimethylsiloxane

PDSD - proton-driven spin diffusion

PEDRI - proton-electron double resonance imaging

PET - positron emission tomography

PHIP - parahydrogen-induced polarization

PHIP-SAH - parahydrogen-induced polarization via side-arm hydrogenation

photo-CIDNP - photochemically induced dynamic nuclear polarization

PMMA - polymethyl methacrylate

PNZ - phenazine

PREPRINT - perturbation-recovered photo-CIDNP-enhanced constant time reverse INEPT RF pulse sequence

PRESPRINT - perturbation-recovered selective-pulse photo-CINDP enhanced reverse INEPT RF pulse sequence

PRESTO-QCPMG - phase-shifted recoupling effects a smooth transfer of order-quadrupolar Carr-Purcell-Meiboom-Gill

PRINOE - parahydrogen and RASER-induced NOE

PRINT - photo-CIDNP-enhanced constant time reverse INEPT RF pulse sequence

PS - pairwise selectivity of H_2 addition

PSI - Photosystem I

PTFE - polytetrafluoroethylene

PTSC - perfluoro(*p*-tolylsulfenyl) chloride

Py - pyridine

QIP - quantum information processing

QRIP - quantum rotor induced polarization

QUASR-SABRE - quasiresonance SABRE

RASER - radio amplification by stimulated emission of radiation

RASPRINT - rapid-acquisition selective pulse photo-CIDNP enhanced reverse INEPT RF pulse sequence

RBC - red blood cells

RC - reaction center

RF - radiofrequency

RF-ONP - radiofrequency-induced optical nuclear polarization

RNA - ribonucleic acid

RP - radical pair

RPM - radical pair mechanism (of CIDEP, CIDNP)

RQM - reverse quartet mechanism (of CIDEP)

RT - room temperature

RTPM - radical-triplet pair mechanism (of CIDEP)

S2hM - singlet-to-heteronuclear-magnetization

S2M - singlet-to-magnetization

SA-BDPA - sulfonated derivative of 1,3-bis(diphenylene)-2-phenylallyl (stable free radical)

SABRE - signal amplification by reversible exchange

SABRE-SHEATH - SABRE in shield enables alignment transfer to heteronuclei

SABRE-ZULF - SABRE at zero- to ultralow-field

SAC - single-atom catalyst

SAH - side-arm hydrogenation

SAM - self-assembled monolayer

SCRP - spin-correlated radical pair

SCRPM - spin-correlated radical pair mechanism

SE - solid effect

SE-HSQC - sensitivity-enhanced HSQC

SENS - surface-enhanced NMR spectroscopy

SEOP - spin-exchange optical pumping

SIDNP - spin-injected DNP

SLIC - spin-lock induced crossing

SLM - standard liters per minute

SNP - stimulated nuclear polarization

SNR - signal-to-noise ratio

SNR_t - $SNR/(time)^{1/2}$

SOFAST-HMQC - band-selective optimized flip-angle short-transit heteronuclear multi-quantum correlation

SPINOE - spin-polarization-induced nuclear Overhauser effect

SPY-MR - spin polarimetry magnetic resonance

SQUARE - surface quadrupolar relaxation

SQUID - superconducting quantum interference device

TBE - 1,1,1,2-tetrabromoethane

TCE - 1,1,2,2-tetrachloroethane

TCNB - 1,2,4,5-tetracyanobenzene

TCPPNa - tetrakis(4-carboxyphenyl)porphyrin sodium salt

*t*DNP - triplet dynamic nuclear polarization

TEM - transmission electron microscopy

TEMPO - 2,2,6,6-tetramethylpiperidin-1-oxyl (stable free radical)

TEMPOL - 4-hydroxy-2,2,6,6-tetramethylpiperidin-1-oxyl (stable free radical)

TEMPONE - 4-oxo-2,2,6,6-tetramethylpiperidine-N-oxyl (stable free radical)

TM - thermal mixing

TM - triplet mechanism of CIDEP

TOAC - 4-amino-1-oxyl-2,2,6,6-tetramethylpiperidine-4-carboxylic acid (stable free radical)

TOF-MS - time-of-flight mass spectrometer

TOP DNP - Time-optimized dynamic nuclear polarization

TOTAPOL - 1-(TEMPO-4-oxy)-3-(TEMPO-4-amino)propan-2-ol (nitroxide biradical)

TP - tissue-dissolved phase (of ^{129}Xe)

tpatcnH$_3$ - 1,4,7-tris[(6-carboxypyridin-2-yl)methyl]-1,4,7- triazacyclononane

TPP - tetraphenyl porphyrin

TR-EPR - time-resolved EPR

Trp - (L-)tryptophan

TSM - three-spin mixing

Tyr - L-tyrosine

UV - ultraviolet

UV-vis - ultraviolet-visible (spectroscopy)

VR - vibrational relaxation

WOLF - weak oscillating low field

ZFS - zero-field splitting

ZQ - zero-quantum (transition or coherence)

ZULF - zero- to (and) ultralow-field

NOTATION

A - hyperfine coupling constant (value)

A/E - absorption/emission (low field / high field) pattern in a spectrum

A, E_a, E_b - irreducible representations of the C_3 molecular symmetry group

AB - strongly coupled nuclear spin system

AX - weakly coupled nuclear spin system

B_0 - external (applied) permanent magnetic field

B_1 - oscillating (radiofrequency or microwave) magnetic field

B_{1e} - oscillating magnetic field applied to electron spins

b - characteristic pressure ratio of the gases in SEOP

C_3 - molecular symmetry group of a -CH_3 moiety

C_{3v} - spatial point symmetry group of a -CH_3 moiety

D - photosensitizer dye molecule

D - diffusion coefficient, diffusivity

^{S0}D - photosensitizer dye molecule in its singlet ground state

^{S1}D - photosensitizer dye molecule in its first excited singlet state

^{T}D - photosensitizer dye molecule in a triplet state

D_1, D_2 - spectral transition lines of alkali metal atoms (e.g., ^{87}Rb)

E/A -emission/absorption (low field / high field) pattern in a spectrum

E*/A - combination of net emissive (E) and E/A type polarization pattern

F - total angular momentum

F-pairs - radical pairs formed upon random collisions of transient radicals

f - parahydrogen fraction in H_2

f - leakage factor

f - dilution factor

f_{129} - isotopic fraction of ^{129}Xe

^{3}He* - metastable 2 $^3S_1$ electronic state of helium-3

H_2@C_{60} - endofullerene, with an H_2 molecule enclosed within the C_{60} cage

H_2O@C_{60}, $H_2{}^{17}O$@C_{60} - endofullerene, with an H_2O ($H_2{}^{17}O$) molecule enclosed within the C_{60} cage

I - magnetic resonance signal intensity

I_0 - magnetic resonance signal intensity under conditions of thermal equilibrium

I - nuclear spin angular momentum

I_z - nuclear spin angular momentum component along an applied magnetic field B_0

J - indirect coupling (constant) between nuclear spins

J - exchange coupling between two unpaired electrons

J - rotational quantum number of a molecule

J - total electron angular momentum

$J(\omega,\tau_c)$ - spectral density function

k - wavevector

k_B - Boltzmann constant

L - orbital angular momentum (operator)

l - orbital angular momentum quantum number

M - photo-CIDNP substrate molecule

M - nuclear magnetization of an object or sample

MCM-41 - mesoporous silica

m_F - total angular momentum quantum number

m_I - magnetic sublevels of a nucleus with spin *I*

m_J - total electron angular momentum quantum number

MNP - metal nanoparticle

mol% - molar fraction

m_S - magnetic sublevels of an electron spin *S*

n_i - number of species in state i

n-H_2 - normal hydrogen with o-H_2:p-H_2 = 3:1

o-D_2 - orthodeuterium

$o\text{-}H_2$ - orthohydrogen

P - pressure

P_+, P_0, P_- - populations of T_+, T_0, T_- states, respectively

^{S0}P, ^{Sn}P, ^{T1}P - (precursor) molecule in a ground singlet electronic state S_0, one of the excited singlet states, or lowest triplet state T_1, respectively

p - spin polarization (general, or nuclear)

p(X) - spin polarization of nucleus X

p_{app} - apparent polarization

p_e - electron spin polarization

$p_{e,therm}$ - electron spin polarization at thermal equilibrium

$p\text{-}H_2$ - parahydrogen

p_{hyp} - spin hyperpolarization

P_{MW} - microwave power

$P\text{-}SiO_2$ - silica gel modified with a phosphine-containing linker

p_{therm} - spin polarization at thermal equilibrium

$PtSn@mSiO_2$ - Pt-Sn nanoparticles confined within a mesoporous silica shell

P_x, P_y, P_z - populations of T_x, T_y, T_z states, respectively

Q_1 - a quartet spin state of a pair comprising a triplet chromophore and a doublet radical

$^{D}R^{\bullet}$ - a doublet-state free radical with an unpaired electron

$^{D}[R^{\bullet}..X]$ - radical-triplet pair in a doublet electron spin state

$^{Q}[R^{\bullet}..X]$ - radical-triplet pair in a quartet electron spin state

r - actual partial pressure ratio of the gases in SEOP

S - singlet spin state (I=0 or S=0)

S - electron spin angular momentum

s - saturation factor

S_n - singlet electronic states of molecules and materials (n=0 - ground state; n=1,2,…- excited states)

S_z - electron spin angular momentum component along an applied magnetic field B_0

T - triplet spin state (I=1 or S=1)

T - temperature

T_+, T_-, T_0 - electron spin sublevels of a triplet molecule in the lab frame of reference, or of a radical pair or biradical

T_{1e} - longitudinal (spin-lattice) relaxation time of electron spin

T_{1n} - longitudinal (spin-lattice) relaxation time of nuclear spin

T_1(X) - T_{1n} of nucleus X

$T_{1\rho}$ - longitudinal relaxation time in the rotating frame

T_{2e} (T_{2e}^*) - transverse (spin-spin) relaxation time of electron spin

T_{2n} (T_{2n}^*) - transverse (spin-spin) relaxation time of nuclear spin

T_n - triplet electronic states of molecules and materials (n=1,2,3…)

T_x, T_y, T_z - electron spin sublevels of a triplet molecule in the molecular frame of reference

V_{129} - production volume of ^{129}Xe

vol% - percentage by volume

W_0 - zero-quantum electron-nuclear cross relaxation rate

W_{1n} - single-quantum nuclear spin relaxation rate

W_2 - double-quantum electron-nuclear cross relaxation rate

wt% - percentage by weight

^{T}X - molecule (chromophore) in a triplet electronic state

[X] - concentration of species X

X - conversion of reactants to products

α - flip angle of a (radiofrequency) pulse

α, β - Zeeman states of spin-1/2 particles

Γ_{SD} - 'spin destruction' rate

Γ - longitudinal relaxation rate (=1/T_{1n}) of a noble gas atom

γ - gyromagnetic ratio of a particle (nucleus, electron)

γ(X) - gyromagnetic ratio of nucleus X (X=^{1}H, ^{13}C, ^{15}N, etc.)

γ_e - gyromagnetic ratio of an electron

γ_n - gyromagnetic ratio of a nucleus

γ_{OP} - optical pumping rate

γ_{SE} - spin exchange rate

Δg - difference in g-factors for a pair of free radicals or a biradical

$\Delta\omega_e$ - linewidth or frequency spread in an EPR spectrum

δ - chemical shift

ε - NMR signal enhancement

θ - contribution factor (MAS NMR)

κ_{sd}^{i} - spin-destruction rate constant for species i

λ - wavelength of light

λ_{MW} - wavelength of microwave radiation

μ - magnetic moment (of a nucleus or electron)

μ_B - Bohr magneton

μ_n - nuclear magneton

ξ - coupling factor between the electron and nuclear spins

σ - chemical shielding of nuclear spin (chemical shift)

σ - binary spin exchange cross-section in SEOP

$\sigma(\lambda)$ - Rb absorption cross-section in SEOP

$\sigma^{\pm}$ - circularly polarized photons

τ_c - correlation time of molecular motion

τ_{DNP} - DNP build-up time constant

τ_R - rotational correlation time of a methyl group

$\Phi_{opt}(\lambda,z,r)$ - power density of the laser light as function of wavelength and spatial coordinates

ω_e - the Larmor frequency of an electron

ω_{MW} - microwave irradiation frequency

ω_n - the Larmor frequency of a nucleus

ω_r - sample spinning frequency (MAS NMR)

ω_t - tunnel frequency
